# Computational Exploration of Stereoelectronic Relationships in Manganese‐Catalyzed Hydrogenation Reactions

**DOI:** 10.1002/chem.202501063

**Published:** 2025-05-19

**Authors:** Alister S. Goodfellow, Matthew L. Clarke, Michael Bühl

**Affiliations:** ^1^ EaStCHEM School of Chemistry University of St Andrews Purdie Building St Andrews Fife KY16 9ST UK

**Keywords:** catalyst design, density functional theory, manganese, noncovalent interactions, stereoelectronic effects

## Abstract

Stereoelectronic effects governing Mn‐catalyzed hydrogenation reactions have been deconvoluted through the analysis of a series of in silico catalyst modifications using DFT (PBE0‐D3_PCM(EtOH)_/def2‐TZVP//RI‐BP86_PCM(EtOH)_/def2‐SVP level of theory). Computations were performed on the Mn‐catalyzed reduction of indanone based on a catalyst from the Clarke group, consisting of a tridentate ligand with pyridine, amine, and phosphine donors and a ferrocenyl linker in the backbone. Enantioselectivity enhancements were found through two pathways; first, with the stabilization of aromatic substrates by means of an extended π‐system, enhancing π‐stacking noncovalent interactions; second, by the introduction of steric bulk around the active site to destabilize one of the diastereomeric hydride transfer transition states. Electronic effects were differentiated from sterics by modification of the phenyl groups at the phosphine, *trans‐* to the metal‐hydride bond. While electron‐withdrawing groups increased the thermodynamic driving force, the highest activity is predicted with electron‐donating groups due to the improved basicity of the nitrogen lone pair, required for the initiation of hydrogen activation. Based on these observations, promising routes for synthetic catalyst design may involve donating groups which improve activity, coupled with enantiodiscrimination *via* steric bulk as a more general strategy than being limited to π‐containing substrates.

## Graphical abstract

A detailed computational investigation of stereoelectronic effects in enantioselective manganese‐catalyzed hydrogenation reactions uncovered two general routes to improved selectivity; namely extension of π‐system and increased steric bulk (quantified through occupied volume in quadrants). Electronic effects were evaluated through Hammett correlations, revealing opposing electronic demands on the thermodynamics and kinetics of the key catalytic step of H_2_ activation.

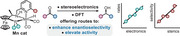

## 1 Introduction

With rising awareness of long‐term sustainability, homogeneous catalysis using earth abundant metals represents a shift away from traditional 4d and 5d metal catalysis. Platinum group metals have been used widely in the last 50 years, with the notable example of Noyori catalysis^[^ ^1^ ^]^ in the field of asymmetric hydrogenations.^[^ ^2^ ^]^ These metals are increasingly scarce and if used in the pharmaceutical industry, necessitate additional treatment steps to remove traces of these metals due to toxicity concerns.^[^ ^3^ ^]^ First‐row transition metals present an alternative approach, avoiding these 4d and 5d metals in order to perform similar or novel transformations. Hydrogenations are widely utilized with both homogeneous and heterogeneous catalysis,^[^ ^4^ ^]^ and 3d transition metals have seen widespread research, with manganese,^[^ ^5^, ^6^ ^]^ iron,^[^ ^7^, ^8^ ^]^ and cobalt^[^ ^9^, ^10^ ^]^ all explored for this purpose in the past decade.

**Figure 1:**
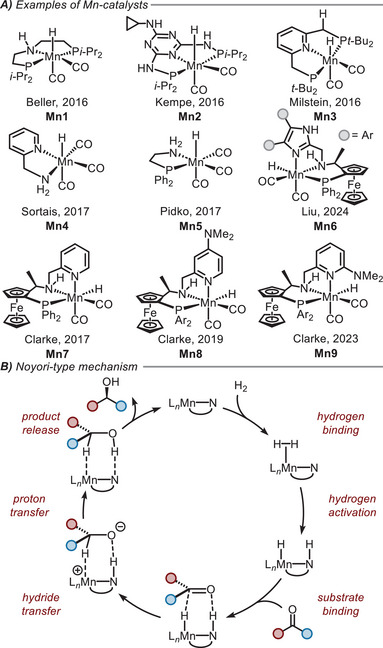
**A)** Development of manganese hydrogenation catalysts. Mn‐hydride species are drawn to compare the reducing species (corresponding to species **v** in the reaction profile in Figure 2A). Ar = 3,5‐Me,4‐OMeC_6_H_2_. **B)** Proposed catalytic cycle of Noyori‐type hydrogenation.^[^ ^25^, ^26^ ^]^

Manganese has been used for the hydrogenation of aldehydes, ketones, and esters from 2016 and more recently of C═N bonds (Figure 1A).^[^ ^11^, ^12^, ^13^, ^14^, ^15^, ^16^, ^17^, ^18^ ^]^ Initial developments saw catalysts capable of reducing esters and ketones, with subsequent improvements enabling enantioselective reductions and the reduction of more challenging substrates. For example, **Mn7** was developed with a facially‐coordinating *N,N,P*‐ligand by the Clarke group with use for the reduction of esters and ketones.^[^ ^19^ ^]^ Work involving our group led to the rational design of a catalyst with improved selectivity for hydrogenation of cyclic ketones.^[^ ^20^ ^]^

Structurally similar catalysts based on phosphino‐ferrocenyl‐aminomethyl‐heterocycles have been studied by other groups including; cycloalkane fused derivatives for improved selectivity towards the reduction of aromatic ketones,^[^ ^21^, ^22^ ^]^ use of strongly donating 4,5‐disubstituted imidazole units (**Mn6**) for highly selective reduction of dialkyl ketimines,^[^ ^18^ ^]^ for asymmetric hydrophosphination using **Mn7** ^[^ ^23^ ^]^ and for atroposelective dynamic kinetic resolutions.^[^ ^24^ ^]^

**Figure 2:**
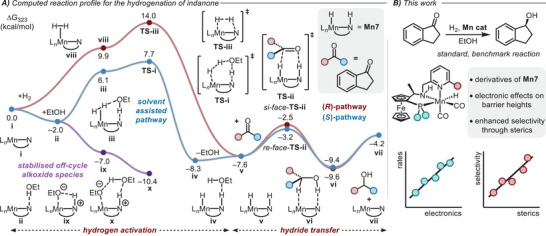
A) Computed reaction profile for the reduction of indanone using **Mn1**.^[^ ^20^ ^]^ Species **v** corresponds to the hydrogenated catalyst (containing a Mn─H bond) shown in Figure 1A. B) This work, investigation of stereoelectronic effects in Mn‐catalyzed hydrogenation reactions (PBE0‐D3_PCM(EtOH)_/def2‐TZVP//RI‐BP86_PCM(EtOH)_/def2‐SVP level of theory).

Hydrogenations using Mn‐catalysts are proposed to proceed through a Noyori‐type bifunctional mechanism (Figure 1B)^[^ ^26^ ^]^ with two main steps: hydrogen activation to form the active metal hydride species and hydride transfer to the electrophilic substrate site to reduce the substrate (see Figure 2A).^[^ ^27^ ^]^ The interplay of these two barrier heights is critical to the ability of the Mn catalyst to reduce challenging substrates. *P*,*N*,*P*‐ and *N*,*N*,*P*‐type pincer ligands have been the most popular and with each system comes a different set of electronic demands across the two main steps of the reaction. For *P*,*N*,*P*‐ligand systems, the kinetic barrier for H_2_ activation is generally lower than the barrier for hydride transfer to the substrate.^[^ ^27^, ^28^ ^]^ This is in contrast to *N*,*N*,*P*‐ligand systems, where it is the other way around.^[^ ^20^, ^29^ ^]^ The interplay of barrier heights has been explored further by Kumar et al.^[^ ^30^ ^]^ across *N*,*N*,*P*‐, *P*,*N*,*P*‐, and *C*,*N*,*C*‐ligand systems and the inclusion of strongly σ‐donating NHC‐containing ligand backbones was shown to balance the barrier heights across the two main steps of the reaction. Due to the comparatively lower barrier for hydride transfer compared to H_2_ activation, *N*,*N*,*P*‐ligand systems have been successfully used to reduce more challenging substrates such as imines.^[^ ^17^, ^31^ ^]^

Computational studies have been performed on asymmetric hydrogenations using Mn with successful rationalization and prediction of enantioselectivities^[^ ^17^, ^20^, ^21^, ^27^, ^28^ ^]^ and to design novel ligand backbones.^[^ ^18^, ^30^, ^32^ ^]^ Assessing effects from different ligand backbones across these works is challenging due to different computational methodologies, different off‐cycle resting states,^[^ ^33^, ^34^, ^35^, ^36^ ^]^ and different substrates or reaction conditions.

In this work we aim to build upon our previous studies, using the PBE0‐D3_PCM(EtOH)_/def2‐TZVP//RI‐BP86_PCM(EtOH)_/def2‐SVP level of theory, chosen from a benchmarking study against 3d transition metal‐hydride heterolytic bond strengths.^[^ ^37^ ^]^ We will apply this methodology to study ketone hydrogenation using indanone as a model substrate across a series of catalysts derived from the *N*,*N*,*P*‐ligand system from the Clarke group (Figure 2B). Through this series we explore routes to improve stereocontrol with π‐systems and with steric bulk, and improvements to activity by identifying the electronic demands of the reactivity while considering some standardized off‐cycle species.

## 2 Results and Discussion

Building upon our previous work, we consider the reduction of indanone as our model reaction and the reaction profile for the reaction catalyzed by **Mn7** ^[^ ^38^ ^]^ is shown in Figure 2A. As we have shown,^[^ ^17^, ^20^ ^]^ the barrier for hydrogen activation (**TS‐i**) is higher than hydride transfer to the substrate (**TS‐ii**) and including representative off‐cycle species lead to a more reasonable, albeit underestimated, overall barrier for the reaction (Δ^‡^ *G*_ES_  =  18.1 kcal/mol, energy span^[^ ^39^ ^]^ from **x** to **TS‐i**). Alkoxide species (**ix** and **x**) are computed to be heavily stabilized and related alkoxides have been observed experimentally^[^ ^33^ ^]^ though the true nature of the global minimum remains system dependent and challenging to accurately identify. After catalyst activation, the first step in the catalytic cycle begins with the binding of H_2_ to a vacant coordination site of the catalyst via a nonclassical η^2^‐binding mode (**iii**, **viii**). Heterolytic splitting of the σ‐bond can then proceed with initiation from the nitrogen lone pair and assistance from a protic relay molecule, modelled with EtOH solvent (**TS‐i**). This protic relay reduces ring strain through formation of a 6‐membered ring, which is 6.3 kcal/mol more stable than the 4‐membered transition state, **TS‐iii**. Following formation of the metal hydride species (**v**), the system is primed to reduce a variety of substrates. For carbonyl‐containing substrates, the reactant molecule approaches from “above” the active site, with alignment of the δ^−^ site of the hydride and δ^+^ carbon centre of the carbonyl, and the δ^+^ amine proton and δ^−^ carbonyl oxygen. Hydrogenation proceeds through an asynchronous concerted process, where the hydride is transferred first to the carbonyl, followed by a barrierless proton transfer to form the alcohol product.^[^ ^20^, ^40^, ^41^ ^]^

**Figure 3:**
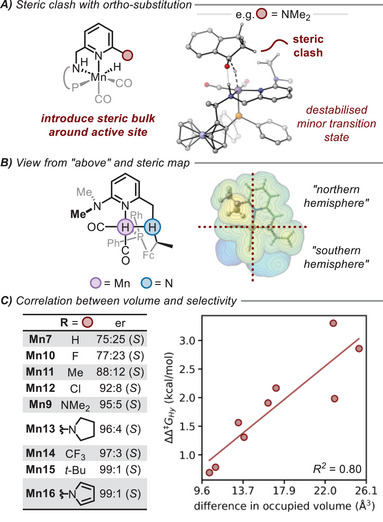
Observed enantioselectivity enhancement for the reduction of indanone through increased steric bulk. **A)** Steric clash in the minor, *pro*‐*R*, **TS‐ii** of **Mn9**. **B)** *Top‐down* view of the hydrogenated catalyst, **v**, with the *ortho*‐NMe_2_ substitution orientated in the north‐west quadrant. Steric map constructed using SambVca 2.1.^[^ ^42^ ^]^ **C)** Correlation of steric bulk measure to computed selectivity. Calculation of buried volume performed using SambVca 2.1.^[^ ^42^ ^]^

First, we consider the enantioselectivity of the reaction which arises from the energetic difference in the two diastereomeric hydride transfer transition states, **TS‐ii**. After the significant improvement in selectivity found upon the addition of an *ortho*‐NMe_2_ group (Figure 3A),^[^ ^20^ ^]^ here we explore the generality of introducing steric bulk with substituents of varying sizes, ranging for example, from R═H (**Mn7**) to R═*t*‐Bu (**Mn15**). Using a steric map of the hydrogenated catalyst (**v**), we can observe the protrusion of the *ortho*‐substituent into the binding site of the substrate with the steric bulk placed in the “*northern hemisphere*” (**Mn9**, Figure 3B). Qualitatively, this would lead to the bulky side of the substrate (*sp* ^3^‐hybridised) preferentially orientated in the “*southern hemisphere*” to minimize steric repulsion. As drawn in Figure 3B, the carbonyl of the substrate upon binding is orientated horizontally, east‐west. With more catalyst steric bulk in the northern hemisphere, the bulkiest side of the substrate (sp^3^‐hybridised) will be orientated to the south, minimizing steric repulsion with the catalyst in the north by orientating the planar sp^2^‐hybridised component of the substrate above the steric bulk introduced on the catalyst. This can be quantified using the steric maps^[^ ^42^ ^]^ and the difference in occupied volume between the northern and southern hemispheres can be calculated (Figure 3C). The larger difference between the north and south hemisphere correlates well (*R* ^2^  =  0.80) to an increase in selectivity (ΔΔ^‡^ *G* between *pro‐R*/*S* **TS‐ii**). While the bulkiest catalyst substituents are straightforward to model computationally, such derivatives may not be synthetically feasible since very bulky pyridines may not fully coordinate to the manganese centre.

The introduction of steric bulk close to the active site generally serves to raise the barrier of hydride transfer, disproportionally destabilizing the minor transition state compared to the major transition state. For the parent system, **Mn7**, the barrier for hydride transfer towards the *pro*‐*S* face of the substrate is 4.4 kcal mol^−1^ and towards the *pro*‐*R* face is 5.1 kcal mol^−1^ (Figure 2A).^[^ ^17^ ^]^ Introducing the *ortho*‐dimethylamino group (**Mn9**) leads to an increase in the barrier height of both transition states, of +0.3 kcal mol^−1^ and +1.6 kcal mol^−1^ respectively, primarily involving a destabilization of the minor transition state.^[^ ^20^ ^]^ Similarly, introducing an *ortho*‐*tert*‐butyl group (**Mn15**) leads to a destabilization of +0.3 kcal mol^−1^ and +2.5 kcal mol^−1^ to each of the diastereomeric transition states. To simply say that the steric bulk destabilizes the minor transition state is an oversimplification. For example, the barriers of *ortho*‐pyrrolidyl (**Mn13**) are altered by −0.4 kcal mol^−1^ and +0.9 kcal mol^−1^ and *ortho*‐pyrrolyl (**Mn16**) by −0.3 kcal mol^−1^ and +2.1 kcal mol^−1^. The substitution does however have a disproportionate effect upon the minor, *pro*‐*R*, diastereomeric transition state and the predicted enhancement in stereocontrol is a combination of weak non‐covalent interactions that are challenging to deconstruct and isolate from one another.

**Figure 4:**
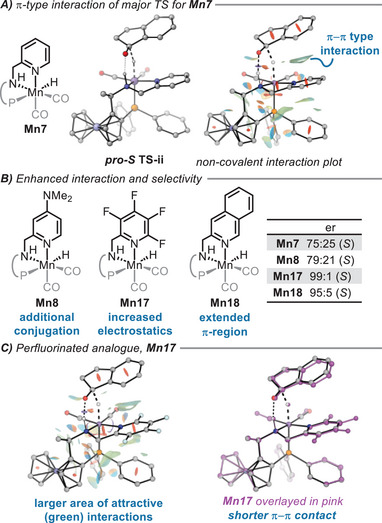
Enhanced enantioselectivity by strengthening non‐covalent interactions. **A)** Weak stabilization of major, *pro*‐*S* transition state through π‐type interactions. Visualization of non‐covalent interactions by NCIPlot 4.0.^[^ ^43^ ^]^ **B)** Increased enantioselectivity with extended conjugation and enhanced electrostatics. **C)** Increased non‐covalent interactions of **Mn17** leading to a shorter π‐π contact between the substrate and catalyst. Major, *pro*‐*S*, **TS‐ii** of **Mn17** overlayed in pink on top of **Mn7**.

While modelling the reactivity of the *para*‐NMe_2_ substituted **Mn8**, we found a very slight increase in selectivity compared to **Mn7** (0.1 kcal mol^−1^). This is likely to be attributable to numerical noise and is not reflected experimentally^[^ ^19^ ^]^ but **Mn8** does also contain a slightly larger π‐region compared to **Mn7** due to the added conjugated dimethylamino unit. From this perspective, there may be an alternative route to improve stereocontrol; while the introduction of steric bulk led to the disparate destabilization of diastereomeric transition states, a larger π‐region may be a route to stabilize one of the diasteromeric transition states through attractive noncovalent interactions (Figure 4A). By manipulation of the π‐system through perfluoronation (**Mn17**), we calculated an enhanced stereocontrol of 99:1 (*S*), significantly increasing ΔΔ^‡^ *G* by stabilizing the favored diastereomeric transition state through increased noncovalent interactions in the major, *pro*‐*S* transition state (Figure 4B). The stronger interaction is emphasized by a shortened π─π contact between the aromatic rings of the catalyst and the substrate (Figure 4C). This also contributes towards a lower barrier height of **TS‐ii** for **Mn17**, 2.2 kcal mol^−1^ lower in energy than **Mn7**. The change in barrier height is likely to be predominantly due to the stronger noncovalent interactions rather than the electronics of the system, as the barrier height of the minor diastereomeric transition state remains similar for both **Mn7** and **Mn17**, Δ^‡^ *G*  =  5.1 kcal mol^−1^ and 5.2 kcal mol^−1^ respectively (from **v** to **TS‐ii**). Alternatively, extension of the π‐region through benzannulation (**Mn18**) led to an improvement in the computed enantioselectivity to 95:5 (*S*). This catalyst derivative features a slightly more electron‐donating ring than pyridine (of **Mn7**), based on pKa values of pyridine and isoquinoline and no significant change to sterics. Neither should hinder coordination to the manganese centre and as a result, may represent an interesting candidate for catalysis studies in the future.

**Figure 5:**
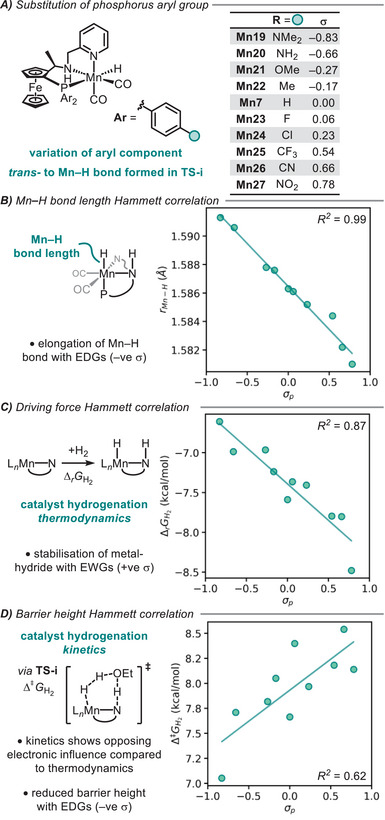
**A)** Variation of the electronics of **Mn7** by phosphorus aryl substitution. **B)** Hammett analysis of bond lengths. **C)** Hammett analysis of driving force of metal‐hydride formation. **D)** Hammett analysis of barrier height of metal‐hydride formation. EDG = electron‐donating group. EWG = electron‐withdrawing group.

Of the three experimentally synthesized catalysts **Mn7–9**, the *ortho*‐dimethylamino substituted catalyst (**Mn9**) was the most selective and the *para*‐dimethylamino substituted catalyst (**Mn8**) was the most active.^[^ ^20^ ^]^ Both experimental iterations included an electron‐rich phosphorus aryl group and a donating dimethylamino group on the pyridine. The strength of the metal‐hydride bond is central to this catalysis and intuitively should be stabilized with an electron‐poor metal centre. By varying the para‐aryl phosphine groups, *trans*‐ to the hydride, we can probe the electronic influence of this step‐in isolation from any steric effects on this reactivity (Figure 5A). Correlation of the Mn─H bond length with Hammett parameters^[^ ^44^ ^]^ (*R* ^2^  =  0.99, Figure 5B) reveals that the metal‐hydride bond is shortened with electron‐withdrawing substituents. The introduction of these electron‐withdrawing groups indicates an enhanced stability of the metal‐hydride species and correspondingly the thermodynamic driving force for the formation of the hydrogenated catalyst is strengthened (*i.e*., becomes more negative, *R* ^2^  =  0.87, Figure 5C). From the perspective of the Hammond postulate, this would imply that the barrier is likely to be lower, with a more reactant‐like transition state; however, this is not the case and the barrier for catalysts hydrogenation (from **i** to **v**, via **TS‐i**, Figure 2) generally appears to be increased with electron‐withdrawing groups (*R* ^2^  =  0.62, Figure 5D). This implies that, although the quality of the correlation is reduced, the reaction is sped up with additional electron density in the system and the transition state is lowered in energy by electron‐donating groups. This may be related to an increased basicity of the nitrogen lone pair, which is involved in heterolytic splitting of the σ_H─H_ bond.

According to the energy span model from Kozuch and Shaik,^[^ ^39^ ^]^ the rate of reaction will be dependent not only on the barrier height of **TS‐i**, but on the overall energetic span of the catalytic cycle between the highest‐lying transition state (**TS‐i**) and the lowest‐lying minimum (**x**) (Δ^‡^ *G*_ES_  =  18.1 kcal mol^−1^, for **Mn7** in Figure 2A). The stability of the off‐cycle alkoxide species, an unproductive energetic well on the potential energy surface is key in dictating the overall rate. For **Mn8**, with a *para*‐substituted NMe_2_ group on the pyridine ring, **TS‐i** is reduced to Δ*G*  =  6.5 kcal mol^−1^ and there is a minimal change to the energy of **x** (Δ*G*  =  −10.6 kcal mol^−1^) resulting a reduction of energy span to Δ^‡^ *G*_ES_  =  17.1 kcal mol^−1[^ ^17^ ^]^ in good qualitative agreement with the increased activity of **Mn8**. Computations of **Mn9** revealed a destabilization of **TS‐i** to Δ*G*  =  8.1 kcal mol^−1^ and a stabilization of **x** (Δ*G*  =  −12.2 kcal mol^−1^), increasing the energy span to Δ^‡^ *G*_ES_  =  20.3 kcal mol^−1^.^[^ ^20^ ^]^ The importance of these off‐cycle alkoxide species is evident on the overall activity of catalysts, though simplistic Hammett analysis on the phosphine series of catalysts (**Mn7** and **Mn19–Mn27**) was unable to identify key electronic demands relating to the stability of this species (Figure S1). These relatively long distance electronic effects appear to be general across different parts of the ligand. For example, the presence of the methyl substituent on the ferrocenyl linker arm was computed to be beneficial compared to a theoretical unsubstituted example which showed a slightly higher barrier for H_2_ activation by 0.6 kcal mol^−1^ (**Mn28**, see SI).

A number of design principles based on the **Mn7** catalyst emerge from this work. Enantioselectivity can be enhanced by increasing the strength of interactions in the active site through two routes; first, by increasing repulsive steric interactions that further disfavor the minor diastereomeric transition state; and second, by enhancing noncovalent interactions that further stabilize the major diastereomeric transition state through increased π‐type interactions. These strategies are exemplified by **Mn15–16** and **Mn17–18**, respectively, each with noticeable enhancements of selectivities predicted compared to the known catalysts **Mn7–9**. It is worth noting however, that these effects may not necessarily be additive; for example, the combination of extended conjugation in **Mn8** and **Mn18** with perfluorination of the aromatic N‐donor (*c.f*. **Mn17**) was not predicted to lead to further enhancements of selectivities (**Mn29** and **Mn30** in Figure S2 of the Supporting Information).

Computed trends in activity (in terms of H_2_ activation energy) can be influenced by varying electronic properties of the phosphine of the pincer ligand. In addition to the known enhancement of activity through a strongly electron‐donating *para*‐substituent at the pyridine donor (**Mn8**), we calculate that a similar modification of the aryl substituents at the phosphine donor atom could also produce a noticeable speedup (*c.f*. **Mn19**), despite an apparent reduction in thermodynamic driving force for H_2_ activation. Taken together, these principles rationalize previous catalyst derivations of **Mn7** and present routes to target enhanced selectivity and activity. Importantly however, the relevance of such catalysts designed computationally may be influenced by other factors beyond the scope of classical DFT. For instance, the stability of the complex during turnover (*e.g*., too much steric bulk could hinder stable coordination to the metal or induce hemilability), or a change in conformational preferences (*e.g*., *meridional*‐ or *facial*‐coordination modes) could affect the desired performance. Overall, we are convinced that there is much room for further improvements in chemical space around the *N*,*N*,*P*‐based manganese catalyst introduced by the Clarke group, and that theory and computation can have a key role in this design process.

## 3 Conclusion

In summary, we have applied DFT calculations to explore catalyst derivations surrounding the *N*,*N*,*P*‐ligand system introduced by the Clarke group. Inspired by the development of catalysts **Mn7–9**, we have found a number of general routes to improve both selectivity and activity. Enantioselectivity can be enhanced by increasing the strength of interactions in the active site; first, by increasing repulsive steric interactions that disproportionately disfavor the minor diastereomeric transition state; and second, by enhancing stabilizing noncovalent interactions towards the major diastereomeric transition state through increased π‐type interactions. The barrier of the rate‐limiting H_2_ activation step (*i.e*., ultimately, catalytic activity) could be modulated by electronic effects of the aromatic N‐ and P‐donor ligand fragments. Hammett analysis showed some correlation between activation barriers for this step and electronic parameters: the barrier height is indicated to decrease with increasing electron‐donating capability of the aromatic donor sites, presumably related to enhanced basicity of the amido nitrogen that can facilitate the heterolytic cleavage of H_2_ in the transition state. In this case, the thermodynamic driving force for the formation of the metal‐hydride species shows an opposing electronic trend. Bringing together these factors, additional electron‐donating groups on the ligand backbone are attractive routes for improving catalytic activity, which could potentially extend the substrate scope to classes that are more difficult to reduce than simple ketones, such as imines, urea derivatives or, ultimately, olefins. Introducing appropriate groups around the active site at the pyridine donor, either with extended nitrogen‐containing heterocycles or as steric groups, should also serve to maintain or further improve enantioselectivity of the system.

## 4 Experimental Section

The DFT methodology was chosen following a benchmarking study of heterolytic metal‐hydride bond strengths of first‐row transition metal complexes^[^ ^37^ ^]^ and has been validated in previous work on ketone reduction.^[^ ^20^ ^]^ Computations were performed at the PBE0‐D3_PCM(EtOH)_/def2‐TZVP//RI‐BP86_PCM(EtOH)_/def2‐SVP level of theory using Gaussian16, C.01 and Gibbs free energies are reported for a typical reaction temperature of 323.15 K.^[^ ^45^, ^46^, ^47^, ^48^, ^49^, ^50^, ^51^, ^52^, ^53^, ^54^, ^55^, ^56^, ^57^, ^58^, ^59^ ^]^ See Supporting Information for further computational details.

## Supporting Information

The authors have cited additional references within the Supporting Information.^[^ ^60^, ^61^, ^62^ ^]^

## Author Contributions

Alister S. Goodfellow carried out all computational studies and wrote the initial version of the manuscript. All authors agreed on the finalised version of the manuscript.

## Conflict of Interests

The authors declare no conflicts of interest.

## Supporting information



Supporting Information

## Data Availability

Details of the computational methodology can be found in the ESI alongside computational raw data and cartesian coordinates. The research data supporting this publication can be accessed from Data underpinning “Computational Exploration of Stereoelectronic Relationships in Manganese‐Catalysed Hydrogenation Reactions”, *University of St Andrews Research Portal*, ^https://doi.org/10.17630/215201f4‐e0ef‐41ef‐9926‐b8229dab5676.[^ ^63^ ^]^

## References

[1] R. Noyori , T. Ohkuma , M. Kitamura , H. Takaya , N. Sayo , H. Kumobayashi , S. Akutagawa , J. Am. Chem. Soc. 1987, 109, 5856.

[2] A. E. Cotman , Chem. – A Eur. J. 2021, 27, 39.

[3] P. Chirik , R. Morris , Acc. Chem. Res. 2015, 48, 2495. 26370392 10.1021/acs.accounts.5b00385

[4] M. A. Stoffels , F. J. R. Klauck , T. Hamadi , F. Glorius , J. Leker , Adv. Synth. Catal. 2020, 362, 1258. 32322184 10.1002/adsc.201901292 PMC7161914

[5] K. Das , S. Waiba , A. Jana , B. Maji , Chem. Soc. Rev. 2022, 51, 4386. 35583150 10.1039/d2cs00093h

[6] Y. Wang , M. Wang , Y. Li , Q. Liu , Chem 2021, 7, 1180.

[7] Q. Liang , D. Song , Chem. Soc. Rev. 2020, 49, 1209. 31984394 10.1039/c9cs00508k

[8] D. Wei , C. Darcel , Chem. Rev. 2019, 119, 2550. 30548065 10.1021/acs.chemrev.8b00372

[9] W. Liu , B. Sahoo , K. Junge , M. Beller , Acc. Chem. Res. 2018, 51, 1858. 30091891 10.1021/acs.accounts.8b00262

[10] H. Pellissier , H. Clavier , Chem. Rev. 2014, 114, 2775. 24428605 10.1021/cr4004055

[11] S. Elangovan , C. Topf , S. Fischer , H. Jiao , A. Spannenberg , W. Baumann , R. Ludwig , K. Junge , M. Beller , J. Am. Chem. Soc. 2016, 138, 8809. 27219853 10.1021/jacs.6b03709

[12] F. Kallmeier , T. Irrgang , T. Dietel , R. Kempe , Angew. Chemie – Int. Ed. 2016, 55, 11806. 10.1002/anie.201606218 27571701

[13] A. Mukherjee , A. Nerush , G. Leitus , L. J. W. Shimon , Y. Ben David , N. A. Espinosa Jalapa , D. Milstein , J. Am. Chem. Soc. 2016, 138, 4298. 26924231 10.1021/jacs.5b13519

[14] K. Z. Demmans , M. E. Olson , R. H. Morris , Organometallics. 2018, 37, 4608.

[15] A. Bruneau‐Voisine , D. Wang , T. Roisnel , C. Darcel , J. B. Sortais , Catal. Commun. 2017, 92, 1. 10.1021/acs.orglett.7b01657 28632391

[16] R. van Putten , E. A. Uslamin , M. Garbe , C. Liu , A. Gonzalez‐de‐Castro , M. Lutz , K. Junge , E. J. M. Hensen , M. Beller , L. Lefort , E. A. Pidko , Angew. Chemie – Int. Ed. 2017, 56, 7531. 10.1002/anie.201701365 PMC5485043 28429449

[17] C. L. Oates , A. S. Goodfellow , M. Bühl , M. L. Clarke , Green Chem. 2023, 25, 3864.

[18] M. Wang , S. Liu , H. Liu , Y. Wang , Y. Lan , Q. Liu , Nature. 2024, 631, 556. 38806060 10.1038/s41586-024-07581-z

[19] M. B. Widegren , M. L. Clarke , Catal. Sci. Technol. 2019, 9, 6047.

[20] C. L. Oates , A. S. Goodfellow , M. Bühl , M. L. Clarke , Angew. Chem., Int. Ed. 2023, 62, e202212479. 10.1002/anie.202212479 PMC10107995 36341982

[21] S. Zhang , Z. Ma , Y. Li , Y. Su , N. Ma , X. Guo , L. Li , Q. Liu , Z. Wang , J. Catal. 2024, 437, 115682.

[22] J. Yang , L. Yao , Z. Wang , Z. Zuo , S. Liu , P. Gao , M. Han , Q. Liu , G. A. Solan , W. H. Sun , J. Catal. 2023, 418, 40.

[23] R. Postolache , J. M. Pérez , M. C. Reis , L. Ge , E. G. Sinnema , S. R. Harutyunyan , Org. Lett. 2023, 25, 1611. 36892214 10.1021/acs.orglett.2c04256 PMC10028696

[24] Y. B. Wan , X. P. Hu , ACS Catal. 2024, 17633.

[25] P. A. Dub , J. C. Gordon , ACS Catal. 2017, 7, 6635.

[26] P. A. Dub , J. C. Gordon , Nat. Rev. Chem. 2018, 2, 396.

[27] M. Garbe , K. Junge , S. Walker , Z. Wei , H. Jiao , A. Spannenberg , S. Bachmann , M. Scalone , M. Beller , Angew. Chemie. 2017, 129, 11389. 10.1002/anie.201705471 28730716

[28] C. S. G. Seo , B. T. H. Tsui , M. V. Gradiski , S. A. M. Smith , R. H. Morris , Catal. Sci. Technol. 2021, 11, 3153.

[29] Y. Wang , L. Zhu , Z. Shao , G. Li , Y. Lan , Q. Liu , J. Am. Chem. Soc. 2019, 141, 17337. 31633346 10.1021/jacs.9b09038

[30] R. Kumar , M. K. Pandey , A. Bhandari , J. Choudhury , ACS Catal. 2023, 13, 4824.

[31] C. S. G. Seo , T. Tannoux , S. A. M. Smith , A. J. Lough , R. H. Morris , J. Org. Chem. 2019, 84, 12040. 31448605 10.1021/acs.joc.9b01964

[32] S. V. Parmar , P. Deshmukh , R. Sankpal , S. Watharkar , V. Avasare , J. Phys. Chem. A 2023, 127, 8338. 37756223 10.1021/acs.jpca.3c04494

[33] A. Passera , A. Mezzetti , Adv. Synth. Catal. 2019, 361, 4691.

[34] D. H. Nguyen , X. Trivelli , F. Capet , J. F. Paul , F. Dumeignil , R. M. Gauvin , ACS Catal. 2017, 7, 2022.

[35] R. J. Hamilton , S. H. Bergens , J. Am. Chem. Soc. 2008, 130, 11979. 18702465 10.1021/ja8034812

[36] K. S. Rawat , S. C. Mandal , P. Bhauriyal , P. Garg , B. Pathak , Catal. Sci. Technol. 2019, 9, 2794.

[37] A. S. Goodfellow , M. Bühl , Molecules. 2021, 26, 4072. 34279412 10.3390/molecules26134072 PMC8271472

[38] M. B. Widegren , G. J. Harkness , A. M. Z. Slawin , D. B. Cordes , M. L. Clarke , Angew. Chemie – Int. Ed. 2017, 56, 5825. 10.1002/anie.201702406 28425169

[39] S. Kozuch , S. Shaik , Acc. Chem. Res. 2011, 44, 101. 21067215 10.1021/ar1000956

[40] Y. Zhao , L. Zhang , M. Pu , M. Lei , Dalt. Trans. 2021, 50, 14738. 10.1039/d1dt02410h 34590102

[41] V. Papa , J. Fessler , F. Zaccaria , J. Hervochon , P. Dam , C. Kubis , A. Spannenberg , Z. Wei , H. Jiao , C. Zuccaccia , A. Macchioni , K. Junge , M. Beller , Chem. – A Eur. J. 2022, 22, e202202774. 10.1002/chem.202202774 PMC10100126 36193859

[42] L. Falivene , Z. Cao , A. Petta , L. Serra , A. Poater , R. Oliva , V. Scarano , L. Cavallo , Nat. Chem. 2019, 11, 872. 31477851 10.1038/s41557-019-0319-5

[43] R. A. Boto , F. Peccati , R. Laplaza , C. Quan , A. Carbone , J. P. Piquemal , Y. Maday , J. Contreras‐Garcĺa , J. Chem. Theory Comput. 2020, 16, 4150. 32470306 10.1021/acs.jctc.0c00063

[44] C. Hansch , A. Leo , R. W. Taft , Chem. Rev. 1991, 91, 165.

[45] A. D. Becke , Phys. Rev. A. 1988, 38, 3098. 10.1103/physreva.38.3098 9900728

[46] J. P. Perdew , Phys. Rev. B. 1986, 33, 8822. 10.1103/physrevb.33.8822 9938299

[47] J. Tomasi , B. Mennucci , E. Cancès , J. Mol. Struct. 1999, 464, 211.

[48] J. Tomasi , B. Mennucci , R. Cammi , Chem. Rev. 2005, 105, 2999. 16092826 10.1021/cr9904009

[49] S. Grimme , J. Antony , S. Ehrlich , H. Krieg , J. Chem. Phys. 2010, 132, 154104. 20423165 10.1063/1.3382344

[50] S. Grimme , S. Ehrlich , L. Goerigk , J. Comput. Chem. 2011, 32, 1456. 21370243 10.1002/jcc.21759

[51] M. J. Frisch , G. W. Trucks , H. B. Schlegel , G. E. Scuseria , M. A. Robb , J. R. Cheeseman , G. Scalmani , V. Barone , G. A. Petersson , H. Nakatsuji , X. Li , M. Caricato , A. V Marenich , J. Bloino , B. G. Janesko , R. Gomperts , B. Mennucci , H. P. Hratchian , J. V Ortiz , A. F. Izmaylov , J. L. Sonnenberg , D. Williams‐Young , F. Ding , F. Lipparini , F. Egidi , J. Goings , B. Peng , A. Petrone , T. Henderson , D. Ranasinghe , et al. Gaussian 16, Revision C.01, Gaussian Inc., Wallingford CT 2019.

[52] J. P. Perdew , K. Burke , M. Ernzerhof , Phys. Rev. Lett. 1996, 77, 3865. 10062328 10.1103/PhysRevLett.77.3865

[53] J. P. Perdew , K. Burke , M. Ernzerhof , Phys. Rev. Lett. 1997, 78, 1396. 10.1103/PhysRevLett.77.3865 10062328

[54] C. Adamo , V. Barone , J. Chem. Phys. 1999, 110, 6158.

[55] A. Schäfer , H. Horn , R. Ahlrichs , J. Chem. Phys. 1992, 97, 2571.

[56] A. Schäfer , C. Huber , R. Ahlrichs , J. Chem. Phys. 1994, 100, 5829.

[57] F. Weigend , R. Ahlrichs , Phys. Chem. Chem. Phys. 2005, 7, 3297. 16240044 10.1039/b508541a

[58] F. Weigend , Phys. Chem. Chem. Phys. 2006, 8, 1057. 16633586 10.1039/b515623h

[59] B. Mennucci , J. Tomasi , J. Chem. Phys. 1997, 106, 5151.

[60] R. L. Martin , P. J. Hay , L. R. Pratt , J. Phys. Chem. A 1998, 102, 3565.

[61] *The PyMOL Molecular Graphics System, Version 2.4*, Schrödinger, LLC 2020.

[62] C. Y. Legault , Univ. Sherbrooke 2020, Accessed April 1, 2025. www.cylview.org

[63] A. S. Goodfellow , M. L. Clarke , M. Bühl , Dataset, University of St Andrews Research Portal 2025, Accessed April 1, 2025. 10.17630/215201f4-e0ef-41ef-99

