# Supplementary material for: Computational Exploration of Stereoelectronic Relationships in Manganese‐Catalyzed Hydrogenation Reactions
Source: Chemistry. 2025 May 19;31(34):e202501063. doi: 10.1002/chem.202501063 (PMC12172583; doi:10.1002/chem.202501063)
Supplement: Supplementary file 1 — Supporting Information [file CHEM-31-e202501063-s001.pdf]

Supporting Information for

Computational Exploration of Stereoelectronic Relationships in Manganese-Catalysed Hydrogenation Reactions

Alister S. Goodfellow,[a] Matthew L. Clarke[a] and Michael Bühl*[a]

----

[a] Dr. Alister S. Goodfellow, Prof. Dr. Matthew L. Clarke, Prof. Dr. Michael Bühl
EaStCHEM School of Chemistry
University of St Andrews
Purdie Building, St Andrews, Fife, KY16 9ST, UK.
E-mail: buehl@st-andrews.ac.uk

Contents

Computational Details 2
Additional Hammett Analysis 2
Additional Catalyst Selectivities 2
Catalyst Derivative Structures 3
Summary of Computed Catalyst Data 4
References 5
Computational Data 6

Computational Details

The DFT methodology was chosen following a benchmarking study of heterolytic metal-hydride bond strengths of first-row transition metal complexes[1] and has been validated in previous work on ketone reduction.[2] Computations were performed at the PBE0-D3$_{PCM(EtOH)}$/def2-TZVP//RI-BP86$_{PCM(EtOH)}$/def2-SVP[3–11] level of theory, with both optimisations and single-points performed with implicit solvation (IEF-PCM) employing the parameters of ethanol (ε = 24.85).[12–14] Dispersion was included at the level of single-point with DFT-D3(BJ) empirical correction from Grimme,[15] including Becke-Johnson dampening.[16] Thermochemistry was evaluated at 1 atm and 298.15 K, at the level of optimisation with computation of harmonic frequencies to verify the nature of the stationary point. Gibbs free energy was evaluated to the elevated temperature of 323.15 K by the scaling of entropies, with further empirical entropy corrections by Martin, Hay and Pratt (S_{MHP} = 3.929 kcal mol^{-1} per particle) included to limit the overestimation of translational freedom in the solvent phase.[17] Computations were performed with an ultrafine integration grid (99,590), using the Gaussian16, C.01 programme.[18] Topographic steric-maps and quantification of buried volume were performed with SambVca 2.1,[19] analysis of non-covalent interactions were performed using NCIPlot 4.0[20] and structures were visualised using PyMOL 2.4.1[21] and CYLview20.[22]

Additional Hammett Analysis

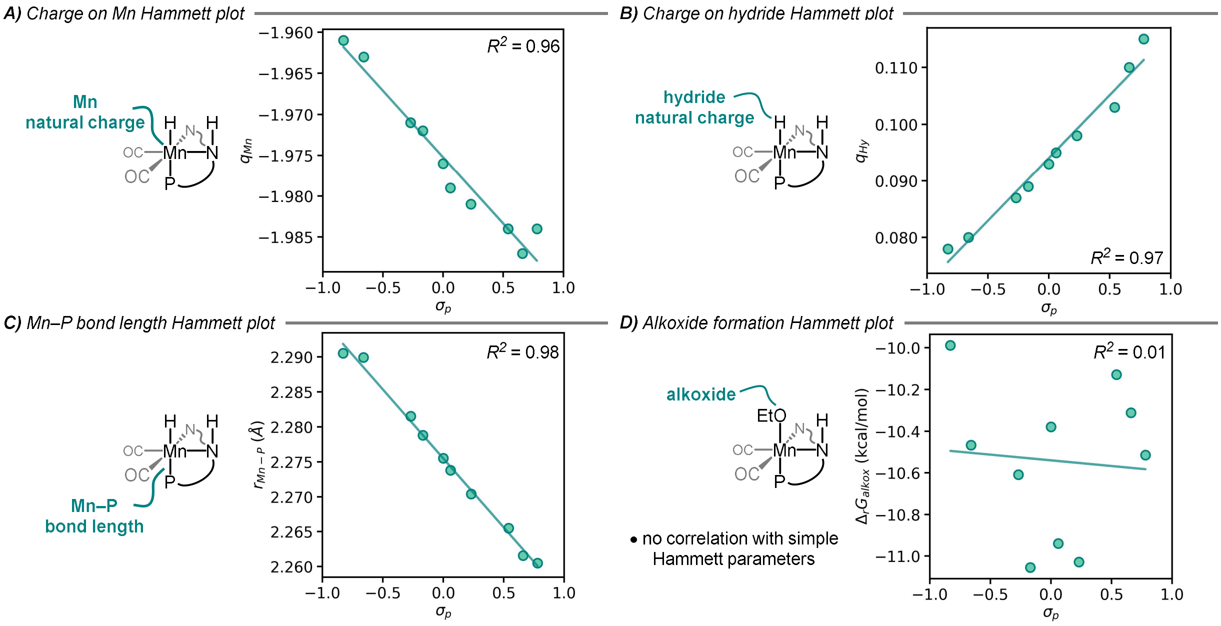

Figure S1. Additional Hammett analysis. **A)** Natural charge on Mn centre. **B)** Natural charge on hydride. **C)** Mn–P bond length. **D)** Thermodynamic driving force for alkoxide formation.

Additional Catalyst Selectivities

Additional catalyst derivatives

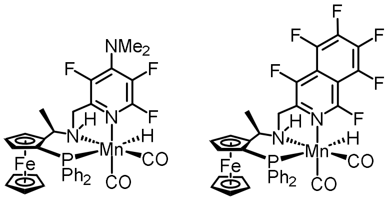

	**Mn29**	**Mn30**
$\Delta\Delta^{\ddagger}G$	0.9 kcal/mol	< 0.1 kcal/mol
e.r.	80:20 (*S*)	racemic

Figure S2. Computed selectivities for two additional catalyst derivatives.

Catalyst Derivative Structures

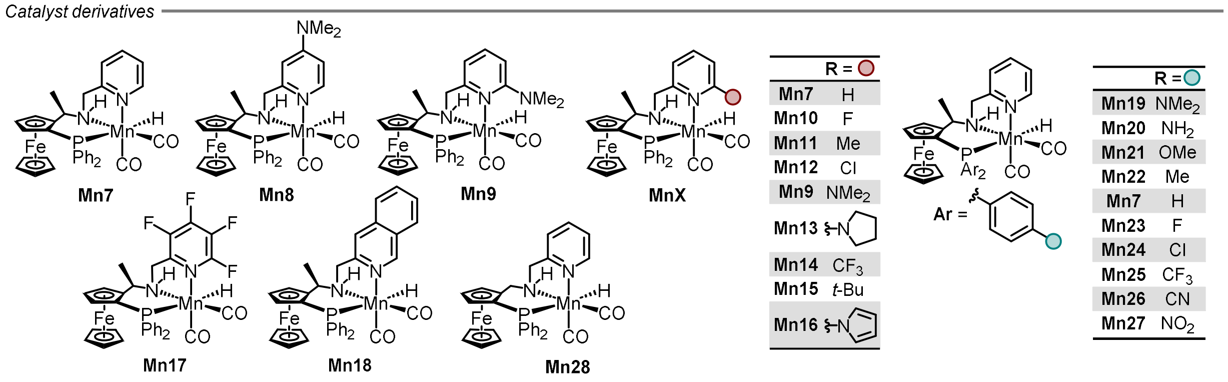

Figure S3. Labelling of the catalyst derivatives modelled in this work.

Summary of Computed Catalyst Data

Table S1. Relative free energies for each catalyst derivative **MnX**. Energies reported as free energies ($\Delta G_{323.15}$) in kcal mol^{-1}.

MnX	i	ii	iii	iv	v	vi_S	vi_R	vii	viii	ix	x	TS-i	TS-ii_re	TS-ii_si	TS-iii	ES	ΔΔG
**Mn7**	0.00	-2.03	6.05	-8.31	-7.59	-9.37	-9.58	-4.16	9.92	-7.03	-10.38	7.66	-3.20	-2.51	14.00	18.04	0.69
**Mn8**	0.00	-2.52	5.60	-8.19	-6.75	-10.12	-9.48	-4.16	10.40	-6.12	-10.57	6.55	-3.37	-2.53	14.38	17.12	0.84
**Mn9**	0.00	-2.68	6.70	-8.55	-6.75	-8.04	-7.62	-4.16	10.75	-7.03	-12.23	8.09	-2.03	-0.12	14.16	20.31	1.91
**Mn10**	0.00	-2.35	5.69	-8.78	-8.06	-9.39	-9.54	-4.16	9.38	-5.95	-10.95	7.41	-3.65	-2.87	13.47	18.36	0.78
**Mn11**	0.00	-2.42	5.93	-9.25	-8.18	-9.72	-9.95	-4.16	9.71	-5.45	-12.47	7.67	-4.20	-2.89	14.19	20.15	1.31
**Mn12**	0.00	-2.87	4.96	-8.79	-8.10	-9.84	-9.83	-4.16	8.93	-7.58	-12.76	6.67	-4.88	-3.32	13.32	19.43	1.57
**Mn13**	0.00	-3.14	7.51	-7.14	-5.62	-8.93	-9.12	-4.16	12.12	-6.08	-11.10	8.45	-1.63	0.35	14.47	19.54	1.98
**Mn14**	0.00	-2.10	5.28	-8.14	-6.98	-9.09	-9.04	-4.16	8.69	-7.46	-13.05	7.88	-2.93	-0.76	13.81	20.93	2.17
**Mn15**	0.00	-3.92	7.52	-11.96	-10.56	-8.51	-7.94	-4.16	11.13	-10.60	-14.41	8.94	-5.85	-2.99	13.72	23.35	2.86
**Mn16**	0.00	-2.49	5.01	-8.15	-6.76	-9.83	-8.16	-4.16	10.14	-7.77	-12.51	6.84	-2.72	0.39	14.17	19.34	3.11
**Mn17**	0.00	-1.49	5.35	-8.52	-7.53	-8.83	-8.67	-4.16	8.95	-6.77	-11.23	7.95	-5.25	-2.32	13.25	19.18	2.93
**Mn18**	0.00	-2.52	5.52	-9.22	-8.50	-10.15	-9.60	-4.16	9.89	-6.03	-11.64	7.18	-5.24	-3.35	13.54	18.82	1.89
**Mn19**	0.00	-2.47	6.18	-7.84	-6.61	-9.31	-9.94	-4.16	9.84	-5.20	-9.99	7.05	-3.50	-2.17	13.86	17.04	1.33
**Mn20**	0.00	-2.10	5.63	-7.59	-6.99	-9.86	-9.71	-4.16	9.80	-5.52	-10.47	7.71	-3.25	-2.21	13.85	18.18	1.04
**Mn21**	0.00	-1.60	5.91	-7.65	-6.96	-9.55	-9.44	-4.16	10.62	-5.13	-10.61	7.82	-2.86	-2.56	14.05	18.43	0.30
**Mn22**	0.00	-2.06	5.70	-8.25	-7.24	-10.23	-9.46	-4.16	9.93	-5.33	-11.06	8.05	-3.52	-2.66	13.75	19.11	0.87
**Mn23**	0.00	-2.03	6.04	-8.28	-7.36	-9.98	-9.36	-4.16	9.90	-5.43	-10.94	8.40	-3.55	-2.52	14.00	19.34	1.03
**Mn24**	0.00	-2.18	6.20	-8.04	-7.41	-9.54	-9.11	-4.16	9.92	-5.56	-11.03	7.97	-3.66	-2.75	13.80	19.00	0.91
**Mn25**	0.00	-1.20	6.73	-8.61	-7.80	-9.06	-9.06	-4.16	9.99	-5.15	-10.13	8.18	-3.77	-2.73	14.29	18.31	1.04
**Mn26**	0.00	-1.91	6.57	-8.44	-7.80	-9.20	-8.82	-4.16	10.09	-5.34	-10.31	8.54	-4.02	-2.97	13.80	18.85	1.05
**Mn27**	0.00	-1.78	6.35	-8.99	-8.48	-8.85	-9.21	-4.16	9.82	-5.77	-10.51	8.14	-4.23	-3.55	13.79	18.65	0.68
**Mn28**	0.00	-2.47	5.47	-7.80	-6.73	-9.16	-9.20	-4.16	10.16	-4.40	-9.96	8.32	-3.33	-2.18	14.15	18.29	1.16

References

[1] A. S. Goodfellow, M. Bühl, *Molecules* **2021**, *26*, 4072.

[2] C. L. Oates, A. S. Goodfellow, M. Bühl, M. L. Clarke, *Angew. Chemie Int. Ed.* **2023**, *62*, e202212479.

[3] A. D. Becke, *Phys. Rev. A* **1988**, *38*, 3098–3100.

[4] J. P. Perdew, *Phys. Rev. B* **1986**, *33*, 8822–8824.

[5] J. P. Perdew, K. Burke, Y. Wang, *Phys. Rev. B* **1996**, *54*, 16533–16539.

[6] J. P. Perdew, K. Burke, M. Ernzerhof, *Phys. Rev. Lett.* **1997**, *78*, 1396.

[7] C. Adamo, V. Barone, *J. Chem. Phys.* **1999**, *110*, 6158–6170.

[8] A. Schäfer, H. Horn, R. Ahlrichs, *J. Chem. Phys.* **1992**, *97*, 2571–2577.

[9] A. Schäfer, C. Huber, R. Ahlrichs, *J. Chem. Phys.* **1994**, *100*, 5829–5835.

[10] F. Weigend, R. Ahlrichs, *Phys. Chem. Chem. Phys.* **2005**, *7*, 3297–3305.

[11] F. Weigend, *Phys. Chem. Chem. Phys.* **2006**, *8*, 1057–1065.

[12] B. Mennucci, J. Tomasi, *J. Chem. Phys.* **1997**, *106*, 5151–5158.

[13] J. Tomasi, B. Mennucci, E. Cancès, *J. Mol. Struct.* **1999**, *464*, 211–226.

[14] J. Tomasi, B. Mennucci, R. Cammi, *Chem. Rev.* **2005**, *105*, 2999–3093.

[15] S. Grimme, J. Antony, S. Ehrlich, H. Krieg, *J. Chem. Phys.* **2010**, *132*, 154104.

[16] S. Grimme, S. Ehrlich, L. Goerigk, *J. Comput. Chem.* **2011**, *32*, 1457–1465.

[17] R. L. Martin, P. J. Hay, L. R. Pratt, *J. Phys. Chem. A* **1998**, *102*, 3565–3573.

[18] M. J. Frisch, G. W. Trucks, H. B. Schlegel, G. E. Scuseria, M. A. Robb, J. R. Cheeseman, G. Scalmani, V. Barone, G. A. Petersson, H. Nakatsuji, X. Li, M. Caricato, A. V Marenich, J. Bloino, B. G. Janesko, R. Gomperts, B. Mennucci, H. P. Hratchian, J. V Ortiz, A. F. Izmaylov, J. L. Sonnenberg, D. Williams-Young, F. Ding, F. Lipparini, F. Egidi, J. Goings, B. Peng, A. Petrone, T. Henderson, D. Ranasinghe, V. G. Zakrzewski, J. Gao, N. Rega, G. Zheng, W. Liang, M. Hada, M. Ehara, K. Toyota, R. Fukuda, J. Hasegawa, M. Ishida, T. Nakajima, Y. Honda, O. Kitao, H. Nakai, T. Vreven, K. Throssell, J. A. Montgomery, Jr., J. E. Peralta, F. Ogliaro, M. J. Bearpark, J. J. Heyd, E. N. Brothers, K. N. Kudin, V. N. Staroverov, T. A. Keith, R. Kobayashi, J. Normand, K. Raghavachari, A. P. Rendell, J. C. Burant, S. S. Iyengar, J. Tomasi, M. Cossi, J. M. Millam, M. Klene, C. Adamo, R. Cammi, J. W. Ochterski, R. L. Martin, K. Morokuma, O. Farkas, J. B. Foresman, D. J. Fox, *Gaussian 16, Revision C.01*, Gaussian Inc., Wallingford CT, **2019**.

[19] L. Falivene, Z. Cao, A. Petta, L. Serra, A. Poater, R. Oliva, V. Scarano, L. Cavallo, *Nat. Chem.* **2019**, *11*, 872–879.

[20] R. A. Boto, F. Peccati, R. Laplaza, C. Quan, A. Carbone, J. P. Piquemal, Y. Maday, J. Contreras-García, *J. Chem. Theory Comput.* **2020**, *16*, 4150–4158.

[21] *The PyMOL Molecular Graphics System, Version 2.4*, Schrödinger, LLC, **2020**.

[22] C. Y. Legault, *Univ. Sherbrooke* **2020**, www.cylview.org.

Computational Data

```
EtOH
Frequencies, energies and thermodynamic properties:
 Lowest Vibrational Mode (1/cm) =                          245.6449
 2nd Lowest Vibrational Mode (1/cm) =                      279.5446
 E(RB-P86) (a.u.) =                                       -154.922969451
 Thermal correction to Enthalpy (a.u.) =                   0.082426
 Thermal correction to Gibbs Free Energy (a.u.) =          0.051666
 Total Entropy (cal/Kmol) =                                64.739
 E(RPBE1PBE) (a.u.) =                                     -154.927492887
Optimised cartesian coordinates (Angstrom):
 C      1.228762  -0.220241   0.000005
 C     -0.089034   0.542785   0.000003
 H      2.089062   0.479844   0.000002
 H      1.307054  -0.868131   0.898094
 H      1.307052  -0.868130  -0.898087
 H     -0.134881   1.211056   0.896116
 H     -0.134861   1.211081  -0.896093
 O     -1.159836  -0.401017  -0.000021
 H     -1.993111   0.107152   0.000088
---------------------------------------
H2
Frequencies, energies and thermodynamic properties:
 Lowest Vibrational Mode (1/cm) =                          4268.9553
 2nd Lowest Vibrational Mode (1/cm) =
 E(RB-P86) (a.u.) =                                       -1.17256159454
 Thermal correction to Enthalpy (a.u.) =                   0.013030
 Thermal correction to Gibbs Free Energy (a.u.) =         -0.001825
 Total Entropy (cal/Kmol) =                                31.264
 E(RPBE1PBE) (a.u.) =                                     -1.16813917680
Optimised cartesian coordinates (Angstrom):
 H      0.000000   0.000000   0.383930
 H      0.000000   0.000000  -0.383930
---------------------------------------
indanol
Frequencies, energies and thermodynamic properties:
 Lowest Vibrational Mode (1/cm) =                          107.4633
 2nd Lowest Vibrational Mode (1/cm) =                      147.1930
 E(RB-P86) (a.u.) =                                       -423.896842178
 Thermal correction to Enthalpy (a.u.) =                   0.172666
 Thermal correction to Gibbs Free Energy (a.u.) =          0.130691
 Total Entropy (cal/Kmol) =                                88.346
 E(RPBE1PBE) (a.u.) =                                     -423.881563844
Optimised cartesian coordinates (Angstrom):
 C     -0.344837   0.933846   0.044309
 C      0.073518  -0.407502   0.184895
 C     -0.852349  -1.462018   0.162888
 C     -2.219337  -1.161945  -0.000618
 C     -2.642963   0.176253  -0.134683
 C     -1.709090   1.232424  -0.112662
 C      0.854637   1.864671   0.099607
 C      2.049117   0.913127  -0.165103
 C      1.586979  -0.479091   0.352063
 H     -0.509083  -2.505073   0.259590
 H     -2.961814  -1.975796  -0.028043
 H     -3.714424   0.398241  -0.265944
 H     -2.048109   2.275207  -0.228313
 H      0.789478   2.705687  -0.621872
 H      3.000320   1.249818   0.293340
 O      2.216197  -1.598281  -0.259470
 H      0.934943   2.323860   1.111741
 H      2.218420   0.823100  -1.261759
 H      1.830539  -0.579002   1.436039
 H      1.956109  -1.588373  -1.203195
---------------------------------------
indanone
Frequencies, energies and thermodynamic properties:
 Lowest Vibrational Mode (1/cm) =                          77.6374
 2nd Lowest Vibrational Mode (1/cm) =                      128.5655
 E(RB-P86) (a.u.) =                                       -422.707662993
 Thermal correction to Enthalpy (a.u.) =                   0.149568
 Thermal correction to Gibbs Free Energy (a.u.) =          0.108052
 Total Entropy (cal/Kmol) =                                87.378
 E(RPBE1PBE) (a.u.) =                                     -422.687390854
Optimised cartesian coordinates (Angstrom):
 C     -0.239280   0.904308  -0.000033
 C      0.093984  -0.468025  -0.000001
 C     -0.893069  -1.473639  -0.000112
 C     -2.239981  -1.084556  -0.000256
 C     -2.582051   0.288723  -0.000289
 C     -1.592237   1.289152  -0.000182
 C      1.002911   1.777816   0.000105
 C      2.186726   0.781022   0.000280
```

```
 C      1.573223  -0.631466   0.000153
 H     -0.597728  -2.535223  -0.000082
 H     -3.037466  -1.844286  -0.000343
 H     -3.645072   0.579156  -0.000406
 H     -1.877964   2.353473  -0.000212
 H      1.014217   2.446255  -0.887331
 H      2.846492   0.885009   0.887722
 O      2.196924  -1.689468   0.000262
 H      1.013989   2.446304   0.887506
 H      2.846793   0.885041  -0.886934
---------------------------------------
Mn7/i
Frequencies, energies and thermodynamic properties:
 Lowest Vibrational Mode (1/cm) =                        23.9444
 2nd Lowest Vibrational Mode (1/cm) =                    29.3476
 E(RB-P86) (a.u.) =                                    -4251.39884820
 Thermal correction to Enthalpy (a.u.) =                 0.542229
 Thermal correction to Gibbs Free Energy (a.u.) =        0.438928
 Total Entropy (cal/Kmol) =                              217.415
 E(RPBE1PBE) (a.u.) =                                  -4250.71884842
Optimised cartesian coordinates (Angstrom):
 Fe2.917887  -0.911218   0.714619
 Mn-1.205675  -0.381228  -1.607893
 P     -0.214139   0.693098   0.019465
 O     -2.232962   1.969720  -3.101141
 O      1.069091  -0.405194  -3.468128
 N     -0.881739  -2.095930  -0.861035
 N     -3.069846  -0.733089  -0.795709
 C      0.928851  -0.443878   0.912034
 C      1.090828  -1.864165   0.610934
 C      1.906876  -2.432854   1.656813
 H      2.234781  -3.478475   1.718254
 C      2.255113  -1.395064   2.591133
 H      2.886086  -1.514044   3.482459
 C      1.662672  -0.167814   2.134191
 H      1.745363   0.807274   2.631163
 C      3.666455  -0.382513  -1.132297
 H      3.068647  -0.193348  -2.033553
 C      4.189991  -1.658820  -0.723089
 H      4.067147  -2.609149  -1.259911
 C      4.883590  -1.477719   0.526943
 H      5.377100  -2.265577   1.111718
 C      4.791084  -0.086132   0.888999
 H      5.202412   0.372177   1.798444
 C      4.036737   0.590584  -0.134082
 H      3.772150   1.655895  -0.145372
 C     -1.419603   1.202395   1.341268
 C     -2.235375   2.335982   1.109901
 H     -2.090116   2.940690   0.199769
 C     -3.227395   2.702884   2.033765
 H     -3.846495   3.594425   1.844439
 C     -3.432185   1.933917   3.195988
 H     -4.211185   2.221420   3.919848
 C     -2.638174   0.797148   3.425484
 H     -2.791785   0.189051   4.331394
 C     -1.637368   0.431734   2.504749
 H     -1.013545  -0.453140   2.704021
 C      0.754204   2.261805  -0.225313
 C      1.216808   2.618052  -1.510380
 H      0.981430   1.981835  -2.375464
 C      1.983535   3.783192  -1.696094
 H      2.335996   4.047520  -2.705780
 C      2.294422   4.608630  -0.601062
 H      2.892870   5.521815  -0.747619
 C      1.828605   4.267228   0.682332
 H      2.059058   4.912824   1.544818
 C      1.060446   3.105005   0.868922
 H      0.685831   2.862555   1.875736
 C      0.482495  -2.589755  -0.600257
 H      1.078973  -2.298271  -1.487596
 C     -1.881235  -2.655604   0.028862
 H     -2.076299  -3.737820  -0.169409
 H     -1.557142  -2.619540   1.103903
 C     -3.166642  -1.897764  -0.092111
 C     -4.190626   0.030918  -0.928338
 H     -4.078568   0.964875  -1.497373
 C     -5.425286  -0.336513  -0.390672
 H     -6.295721   0.320279  -0.535591
 C     -5.528703  -1.546097   0.324417
 C     -4.380416  -2.333313   0.471111
 H     -4.408547  -3.286121   1.021488
 C      0.592559  -4.120965  -0.476446
 H      1.657393  -4.429947  -0.475866
 H      0.130631  -4.512357   0.452963
 H      0.101909  -4.607731  -1.344028
```

```
 C     -1.814880   1.034858  -2.504092
 C      0.192848  -0.383443  -2.667582
 H     -6.488796  -1.865812   0.757583
---------------------------------------
Mn7/ii
Frequencies, energies and thermodynamic properties:
 Lowest Vibrational Mode (1/cm) =                        20.8357
 2nd Lowest Vibrational Mode (1/cm) =                    20.9183
 E(RB-P86) (a.u.) =                                    -4406.33359366
 Thermal correction to Enthalpy (a.u.) =                 0.626987
 Thermal correction to Gibbs Free Energy (a.u.) =        0.508376
 Total Entropy (cal/Kmol) =                              249.638
 E(RPBE1PBE) (a.u.) =                                  -4405.66239607
Optimised cartesian coordinates (Angstrom):
 Fe-2.856548  -1.323029  -0.988718
 Mn1.268364  -0.169377   1.099869
 P     -0.363611   0.964167   0.178129
 O      1.713232   1.641078   3.405533
 O     -0.412574  -1.792409   2.881842
 N      1.380856  -1.460424  -0.324588
 N      2.899562   0.597989   0.092882
 C     -1.228601  -0.078561  -1.066898
 C     -0.844559  -1.443057  -1.422700
 C     -1.637539  -1.824615  -2.566736
 H     -1.608003  -2.799271  -3.070388
 C     -2.504274  -0.731366  -2.918474
 H     -3.241385  -0.728192  -3.732851
 C     -2.263111   0.343173  -1.995063
 H     -2.768653   1.317299  -1.999302
 C     -3.325071  -1.849239   0.948249
 H     -2.653915  -1.766231   1.812757
 C     -3.435489  -2.988667   0.076626
 H     -2.868523  -3.925299   0.163737
 C     -4.401429  -2.676884  -0.946015
 H     -4.696667  -3.331978  -1.776611
 C     -4.890515  -1.343296  -0.704010
 H     -5.624504  -0.804541  -1.318235
 C     -4.223748  -0.830047   0.464690
 H     -4.359845   0.167686   0.901789
 C      0.283816   2.389993  -0.824507
 C      0.729591   3.543825  -0.135903
 H      0.619376   3.607039   0.959058
 C      1.308000   4.614352  -0.836738
 H      1.640559   5.509887  -0.287897
 C      1.464667   4.543161  -2.234721
 H      1.919369   5.382782  -2.783978
 C      1.038839   3.395092  -2.924372
 H      1.157672   3.330735  -4.017811
 C      0.451385   2.323112  -2.225160
 H      0.109137   1.436331  -2.780267
 C     -1.715026   1.785800   1.155899
 C     -1.978764   1.392462   2.485513
 H     -1.366657   0.614295   2.962653
 C     -3.026486   1.987879   3.211922
 H     -3.217977   1.670271   4.249170
 C     -3.821326   2.985419   2.620489
 H     -4.639913   3.452439   3.190795
 C     -3.559165   3.391609   1.298842
 H     -4.170089   4.179425   0.830024
 C     -2.510955   2.800288   0.573002
 H     -2.305039   3.144393  -0.452571
 C      0.214243  -2.289066  -0.703422
 H     -0.229154  -2.623550   0.255851
 C      2.232386  -1.121701  -1.456870
 H      2.798230  -1.999584  -1.851706
 H      1.630834  -0.753743  -2.329941
 C      3.197447  -0.042419  -1.073884
 C      3.715470   1.610135   0.499225
 C      4.842437   2.008276  -0.222259
 C      5.156663   1.341602  -1.422429
 C      4.321195   0.302237  -1.847894
 C      0.584647  -3.563367  -1.485365
 H     -0.291391  -4.238823  -1.560411
 H      0.933577  -3.349982  -2.516515
 H      1.386634  -4.098902  -0.938117
 C      1.531357   0.919250   2.484683
 C      0.211132  -1.127736   2.123793
 H      2.372711  -2.677641   0.706195
 O      2.827874  -3.442541   1.164827
 C      4.225198  -3.346016   0.922724
 H      4.625698  -2.347096   1.229922
 H      4.461691  -3.456026  -0.166839
 H      4.526267  -0.249506  -2.777841
 H      5.466729   2.830220   0.157748
 H      3.445586   2.105266   1.442930
```

```
 C      4.947352  -4.438578   1.703490
 H      6.042643  -4.392849   1.531476
 H      4.761109  -4.330045   2.792708
 H      4.589144  -5.443434   1.395083
 H      6.040286   1.629018  -2.012644
---------------------------------------
Mn7/iii
Frequencies, energies and thermodynamic properties:
 Lowest Vibrational Mode (1/cm) =                         21.7161
 2nd Lowest Vibrational Mode (1/cm) =                     26.8413
 E(RB-P86) (a.u.) =                                     -4407.50616762
 Thermal correction to Enthalpy (a.u.) =                  0.642993
 Thermal correction to Gibbs Free Energy (a.u.) =         0.525677
 Total Entropy (cal/Kmol) =                               246.912
 E(RPBE1PBE) (a.u.) =                                   -4406.83187240
Optimised cartesian coordinates (Angstrom):
 Fe-2.862066  -1.353924  -0.952208
 Mn1.299511  -0.156411   1.294935
 P     -0.330133   0.919681   0.130548
 O      1.331545   1.997381   3.325272
 O     -0.519061  -1.737243   2.987459
 N      1.410440  -1.588008  -0.222582
 N      2.842751   0.629656   0.161584
 C     -1.201192  -0.153812  -1.068192
 C     -0.843884  -1.542567  -1.341349
 C     -1.628320  -1.961320  -2.479450
 H     -1.614947  -2.960318  -2.933312
 C     -2.465317  -0.869697  -2.900785
 H     -3.190628  -0.892743  -3.725351
 C     -2.214792   0.245091  -2.029537
 H     -2.697867   1.228694  -2.089770
 C     -3.390420  -1.769932   0.997108
 H     -2.742577  -1.652907   1.874957
 C     -3.496014  -2.952096   0.184318
 H     -2.946762  -3.891012   0.336003
 C     -4.428683  -2.679780  -0.879680
 H     -4.712076  -3.373065  -1.682976
 C     -4.902269  -1.327926  -0.721140
 H     -5.610804  -0.811521  -1.382661
 C     -4.258424  -0.763618   0.436655
 H     -4.390484   0.257148   0.818235
 C      0.330292   2.311246  -0.915335
 C      0.804631   3.470251  -0.255062
 H      0.728684   3.549568   0.841597
 C      1.363301   4.531121  -0.986418
 H      1.719800   5.429527  -0.457803
 C      1.467993   4.446841  -2.388004
 H      1.906102   5.279034  -2.961481
 C      1.010184   3.295167  -3.050552
 H      1.086560   3.220155  -4.147045
 C      0.444537   2.232449  -2.319950
 H      0.078590   1.342822  -2.855080
 C     -1.696802   1.799904   1.044304
 C     -2.034193   1.443390   2.367756
 H     -1.468552   0.661825   2.893079
 C     -3.099346   2.079413   3.031713
 H     -3.345854   1.788550   4.065161
 C     -3.841554   3.081933   2.383645
 H     -4.673953   3.580602   2.905034
 C     -3.508237   3.450928   1.067371
 H     -4.077341   4.241275   0.552455
 C     -2.441637   2.819387   0.404976
 H     -2.181587   3.135724  -0.617035
 C      0.202093  -2.362560  -0.580188
 H     -0.252102  -2.663532   0.386676
 C      2.100514  -1.065594  -1.398848
 H      2.616205  -1.860112  -1.991535
 H      1.398716  -0.577767  -2.130561
 C      3.101238  -0.016879  -1.009519
 C      3.672340   1.632625   0.549609
 C      4.788442   2.024211  -0.196146
 C      5.069310   1.351462  -1.398644
 C      4.213599   0.319777  -1.804703
 C      0.542019  -3.672053  -1.326093
 H     -0.353745  -4.320501  -1.414252
 H      0.924105  -3.488122  -2.351761
 H      1.313715  -4.226556  -0.755355
 C      1.334712   1.132945   2.520940
 C      0.161794  -1.087860   2.273328
 H      2.635925  -0.668567   2.266855
 H      2.489391  -1.365099   1.838775
 H      2.379748  -2.668078   0.495263
 O      2.955656  -3.324140   1.074415
 C      4.295208  -3.310321   0.616444
 H      4.365955  -3.626687  -0.456820
```

```
 H      4.737337  -2.280536   0.661549
 H      4.395186  -0.235161  -2.737381
 H      5.426856   2.841291   0.170255
 H      3.427947   2.130181   1.499435
 C      5.143454  -4.252201   1.467706
 H      4.745551  -5.287879   1.417684
 H      6.197778  -4.268164   1.120255
 H      5.134012  -3.936718   2.532538
 H      5.944126   1.626645  -2.007685
---------------------------------------
Mn7/iv
Frequencies, energies and thermodynamic properties:
 Lowest Vibrational Mode (1/cm) =                           15.2660
 2nd Lowest Vibrational Mode (1/cm) =                       25.4959
 E(RB-P86) (a.u.) =                                        -4407.53470683
 Thermal correction to Enthalpy (a.u.) =                    0.647685
 Thermal correction to Gibbs Free Energy (a.u.) =           0.529622
 Total Entropy (cal/Kmol) =                                 248.485
 E(RPBE1PBE) (a.u.) =                                      -4406.85863799
Optimised cartesian coordinates (Angstrom):
 Fe-2.946279  -1.235552  -0.957429
 Mn1.366866  -0.282407   1.100895
 P     -0.333967   0.927399   0.183527
 O      1.734598   1.519841   3.394307
 O     -0.214203  -2.092446   2.798750
 N      1.349918  -1.586238  -0.611211
 N      2.854413   0.562477  -0.039897
 C     -1.304956  -0.009695  -1.069337
 C     -0.977829  -1.361391  -1.524833
 C     -1.848826  -1.659856  -2.639450
 H     -1.875325  -2.601764  -3.201395
 C     -2.711287  -0.533256  -2.869332
 H     -3.499840  -0.471556  -3.631274
 C     -2.386279   0.480051  -1.904849
 H     -2.870644   1.460931  -1.816808
 C     -3.286228  -1.882899   0.967683
 H     -2.549180  -1.874013   1.781339
 C     -3.492084  -2.957142   0.032189
 H     -2.947119  -3.910628   0.013798
 C     -4.522179  -2.554328  -0.890954
 H     -4.895255  -3.143610  -1.739344
 C     -4.956021  -1.230777  -0.523060
 H     -5.718255  -0.634645  -1.042528
 C     -4.191105  -0.813976   0.623358
 H     -4.266757   0.154273   1.134702
 C      0.230770   2.430350  -0.764260
 C      0.787498   3.498982  -0.019509
 H      0.803411   3.448330   1.081740
 C      1.313451   4.628440  -0.667726
 H      1.732297   5.454414  -0.070685
 C      1.308883   4.704436  -2.074045
 H      1.722621   5.589542  -2.582864
 C      0.774940   3.641998  -2.823105
 H      0.766959   3.690480  -3.923929
 C      0.239179   2.512471  -2.173991
 H     -0.189204   1.697294  -2.777775
 C     -1.639562   1.690924   1.276247
 C     -1.855739   1.186222   2.576470
 H     -1.235723   0.359749   2.951974
 C     -2.862830   1.726995   3.397190
 H     -3.017104   1.320237   4.409382
 C     -3.665240   2.783383   2.930997
 H     -4.452157   3.208104   3.574458
 C     -3.450680   3.301317   1.640079
 H     -4.068078   4.134909   1.268627
 C     -2.442797   2.762876   0.821131
 H     -2.274194   3.192802  -0.178690
 C      0.071895  -2.302005  -0.946431
 H     -0.293314  -2.669778   0.033194
 C      1.982466  -0.910161  -1.763939
 H      2.405535  -1.623937  -2.503755
 H      1.198260  -0.327809  -2.298291
 C      3.042726   0.048300  -1.290113
 C      3.737147   1.497111   0.401903
 C      4.829189   1.934748  -0.356104
 C      5.038155   1.386621  -1.632511
 C      4.126113   0.427886  -2.099757
 C      0.323284  -3.531611  -1.835766
 H     -0.596883  -4.143464  -1.919027
 H      0.640232  -3.256444  -2.862448
 H      1.107876  -4.170546  -1.381933
 C      1.576608   0.799075   2.465495
 C      0.372843  -1.344298   2.088928
 H      2.587292  -1.151762   1.667652
 H      2.875976  -2.554327   1.365309
```

```
 H      2.006593  -2.322943  -0.274526
 O      3.069937  -3.414317   0.876101
 C      4.487067  -3.501105   0.697132
 H      4.660811  -4.286577  -0.070325
 H      4.896250  -2.549717   0.279390
 H      4.242754  -0.029937  -3.093703
 H      5.507985   2.692472   0.062496
 H      3.557649   1.895181   1.410940
 C      5.218895  -3.861919   1.989541
 H      4.832418  -4.815229   2.406520
 H      6.308401  -3.978224   1.807686
 H      5.085812  -3.071295   2.758319
 H      5.893222   1.698126  -2.251491
---------------------------------------
Mn7/v
Frequencies, energies and thermodynamic properties:
 Lowest Vibrational Mode (1/cm) =                          22.8984
 2nd Lowest Vibrational Mode (1/cm) =                      31.4806
 E(RB-P86) (a.u.) =                                      -4252.59625634
 Thermal correction to Enthalpy (a.u.) =                   0.563059
 Thermal correction to Gibbs Free Energy (a.u.) =          0.459114
 Total Entropy (cal/Kmol) =                                218.770
 E(RPBE1PBE) (a.u.) =                                    -4251.91602182
Optimised cartesian coordinates (Angstrom):
 Fe2.929780  -0.835431   0.735619
 Mn-1.259583  -0.397702  -1.708544
 P     -0.249528   0.662580   0.033109
 O     -2.200394   2.042618  -3.048429
 O      0.985879  -0.554665  -3.601720
 N     -0.831721  -2.300219  -0.800680
 N     -3.033888  -0.770169  -0.750561
 C      0.932578  -0.414412   0.949965
 C      1.140486  -1.837558   0.675462
 C      1.981102  -2.359052   1.730262
 H      2.342747  -3.391331   1.815027
 C      2.306289  -1.290883   2.635117
 H      2.952234  -1.371596   3.519659
 C      1.670495  -0.094932   2.158021
 H      1.729195   0.894732   2.628739
 C      3.634866  -0.315107  -1.128848
 H      3.017684  -0.151088  -2.022155
 C      4.193522  -1.574637  -0.712249
 H      4.083199  -2.534655  -1.234400
 C      4.905286  -1.361270   0.522043
 H      5.425375  -2.130390   1.108654
 C      4.789485   0.032633   0.866618
 H      5.206195   0.512036   1.762579
 C      4.002720   0.679126  -0.151080
 H      3.714543   1.738015  -0.171196
 C     -1.421347   1.220902   1.372471
 C     -2.314719   2.274376   1.057372
 H     -2.239286   2.781171   0.081087
 C     -3.292844   2.686568   1.977111
 H     -3.971089   3.515310   1.717463
 C     -3.409759   2.041992   3.223939
 H     -4.178268   2.363555   3.944723
 C     -2.540595   0.983784   3.539888
 H     -2.623525   0.471467   4.512011
 C     -1.553281   0.575553   2.621791
 H     -0.871504  -0.245286   2.893974
 C      0.714660   2.234486  -0.250642
 C      1.198531   2.534818  -1.542104
 H      0.984231   1.850811  -2.376021
 C      1.956852   3.697841  -1.771029
 H      2.326595   3.916374  -2.785644
 C      2.237571   4.579934  -0.712361
 H      2.828905   5.491946  -0.892060
 C      1.750305   4.295394   0.576995
 H      1.957348   4.984725   1.411315
 C      0.990911   3.134306   0.805215
 H      0.599846   2.935780   1.815526
 C      0.600004  -2.645227  -0.498601
 H      1.145158  -2.340148  -1.413943
 C     -1.767415  -2.547101   0.318269
 H     -1.914128  -3.628267   0.529026
 H     -1.321150  -2.100071   1.234697
 C     -3.088201  -1.873376   0.052978
 C     -4.187959  -0.078627  -0.950877
 H     -4.115619   0.806885  -1.598476
 C     -5.413964  -0.457874  -0.392431
 H     -6.310022   0.146057  -0.599203
 C     -5.473467  -1.607702   0.412337
 C     -4.285333  -2.322029   0.633140
 H     -4.275256  -3.228139   1.257832
 C      0.802256  -4.158995  -0.319487
```

```
 H      1.882884  -4.401258  -0.283354
 H      0.338170  -4.543240   0.611558
 H      0.371092  -4.710081  -1.180640
 C     -1.810415   1.066637  -2.495556
 C      0.119100  -0.468315  -2.794717
 H     -1.114473  -2.891829  -1.593884
 H     -1.921183  -1.272907  -2.854240
 H     -6.422646  -1.941937   0.858045
---------------------------------------
Mn7/vi_R
Frequencies, energies and thermodynamic properties:
 Lowest Vibrational Mode (1/cm) =                        15.2183
 2nd Lowest Vibrational Mode (1/cm) =                    18.8342
 E(RB-P86) (a.u.) =                                    -4675.30871562
 Thermal correction to Enthalpy (a.u.) =                 0.715634
 Thermal correction to Gibbs Free Energy (a.u.) =        0.589126
 Total Entropy (cal/Kmol) =                              266.259
 E(RPBE1PBE) (a.u.) =                                  -4674.62386669
Optimised cartesian coordinates (Angstrom):
 Fe-3.073077  -1.902532  -1.083056
 Mn0.911686   0.336551   0.334860
 P     -1.247979   0.715513   0.533073
 O      1.613420   1.118498   3.105501
 O      0.817289  -2.451444   1.269270
 N      0.796272  -0.080936  -1.577426
 N      1.484613   2.159836  -0.435942
 C     -2.171330  -0.076324  -0.844775
 C     -1.554636  -0.809763  -1.948095
 C     -2.596262  -1.087031  -2.908669
 H     -2.472229  -1.642215  -3.847038
 C     -3.837747  -0.550403  -2.418782
 H     -4.813806  -0.623490  -2.917194
 C     -3.585136   0.065992  -1.145655
 H     -4.332266   0.565735  -0.516003
 C     -2.436565  -3.263012   0.328686
 H     -1.528591  -3.174143   0.938657
 C     -2.520340  -3.884433  -0.966235
 H     -1.690895  -4.354745  -1.511375
 C     -3.877268  -3.760038  -1.434271
 H     -4.262344  -4.114310  -2.399879
 C     -4.633727  -3.063435  -0.424870
 H     -5.696750  -2.794443  -0.487136
 C     -3.743423  -2.753239   0.663721
 H     -4.006391  -2.208629   1.579854
 C     -1.649927   2.521371   0.347493
 C     -1.364554   3.383863   1.433230
 H     -0.979773   2.968522   2.378970
 C     -1.573543   4.767755   1.319271
 H     -1.356213   5.424826   2.176445
 C     -2.057078   5.313707   0.114439
 H     -2.220475   6.399397   0.025308
 C     -2.329232   4.466266  -0.973121
 H     -2.708078   4.884705  -1.919255
 C     -2.127416   3.077236  -0.859465
 H     -2.359724   2.424197  -1.714659
 C     -2.205234   0.289206   2.070160
 C     -1.700078  -0.645295   2.999301
 H     -0.717653  -1.109858   2.837673
 C     -2.446752  -0.994565   4.139679
 H     -2.036320  -1.724562   4.855222
 C     -3.706062  -0.412846   4.367739
 H     -4.288977  -0.685527   5.261753
 C     -4.213771   0.527748   3.452359
 H     -5.195128   0.996848   3.627018
 C     -3.467494   0.880493   2.314952
 H     -3.869349   1.634589   1.620472
 C     -0.071582  -1.182823  -2.057123
 H      0.114446  -2.022039  -1.357172
 C      0.799492   1.071353  -2.477191
 H      1.349576   0.874378  -3.427920
 H     -0.240184   1.348672  -2.792572
 C      1.404760   2.259266  -1.793608
 C      1.988009   3.215970   0.260608
 H      2.034854   3.102364   1.352972
 C      2.433896   4.386111  -0.357642
 H      2.833995   5.205408   0.257357
 C      2.364723   4.485623  -1.760352
 C      1.845521   3.404882  -2.482589
 H      1.772514   3.435977  -3.580195
 C      0.294525  -1.691063  -3.465213
 H     -0.262452  -2.621832  -3.694689
 H      0.058358  -0.955135  -4.260927
 H      1.376684  -1.927451  -3.513267
 C      1.334204   0.807981   1.998072
 C      0.795942  -1.333316   0.878428
```

```
 H      2.343741  -0.607821  -1.768615
 H      3.044934  -0.195553   0.118767
 C      3.892188  -0.512542  -0.593114
 C      4.646041  -1.636418   0.105025
 C      4.941972   0.635182  -0.612901
 C      5.763481  -1.124151   0.800688
 C      4.358657  -3.009689   0.111325
 C      5.849310   0.382024   0.616047
 H      5.525998   0.515347  -1.551865
 C      6.601936  -1.989896   1.523096
 C      5.201382  -3.877955   0.833449
 H      3.491712  -3.396149  -0.448156
 H      6.889721   0.746363   0.483620
 C      6.314027  -3.370438   1.535636
 H      7.480651  -1.601385   2.064462
 H      4.995234  -4.960619   0.845640
 H      6.969427  -4.060148   2.091950
 O      3.345815  -0.841487  -1.833544
 H      4.480482   1.643004  -0.628534
 H      5.446173   0.900221   1.516665
 H      2.712808   5.391877  -2.279118
---------------------------------------
Mn7/vi_S
Frequencies, energies and thermodynamic properties:
 Lowest Vibrational Mode (1/cm) =                        17.2286
 2nd Lowest Vibrational Mode (1/cm) =                    20.8103
 E(RB-P86) (a.u.) =                                     -4675.30900007
 Thermal correction to Enthalpy (a.u.) =                 0.715699
 Thermal correction to Gibbs Free Energy (a.u.) =        0.589832
 Total Entropy (cal/Kmol) =                              264.910
 E(RPBE1PBE) (a.u.) =                                   -4674.62429576
Optimised cartesian coordinates (Angstrom):
 Fe-3.562356  -1.423598  -0.952473
 Mn0.805875  -0.097966   0.488698
 P     -1.168611   0.871477   0.394643
 O      1.528780   0.970759   3.156051
 O     -0.110733  -2.539694   1.847172
 N      0.680910  -0.807012  -1.336748
 N      1.923942   1.325287  -0.499789
 C     -2.202537   0.111326  -0.920577
 C     -1.755895  -0.947037  -1.823470
 C     -2.780706  -1.109525  -2.827362
 H     -2.765973  -1.839238  -3.646820
 C     -3.848719  -0.184237  -2.558015
 H     -4.778738  -0.086589  -3.134204
 C     -3.503582   0.564262  -1.380968
 H     -4.115434   1.348976  -0.917939
 C     -3.402382  -2.634994   0.708282
 H     -2.536333  -2.689415   1.380232
 C     -3.598243  -3.428267  -0.475940
 H     -2.911269  -4.193854  -0.861000
 C     -4.841560  -3.025587  -1.082514
 H     -5.265820  -3.426359  -2.012867
 C     -5.416108  -1.983833  -0.269522
 H     -6.355613  -1.452305  -0.472158
 C     -4.525766  -1.739615   0.835629
 H     -4.666104  -0.991915   1.627101
 C     -1.036377   2.651561  -0.126388
 C     -0.563238   3.589711   0.822446
 H     -0.348692   3.267122   1.854386
 C     -0.369576   4.933043   0.462464
 H     -0.010272   5.653259   1.214710
 C     -0.631291   5.357563  -0.854730
 H     -0.478478   6.411288  -1.136998
 C     -1.087607   4.429904  -1.806602
 H     -1.295161   4.753292  -2.839132
 C     -1.289973   3.083558  -1.446557
 H     -1.662912   2.371774  -2.198825
 C     -2.287475   1.010880   1.874219
 C     -2.119903   0.161500   2.988827
 H     -1.303286  -0.573342   3.006366
 C     -2.995001   0.243237   4.087633
 H     -2.848311  -0.426269   4.949965
 C     -4.046893   1.175920   4.088091
 H     -4.730244   1.240383   4.949651
 C     -4.216005   2.034069   2.985683
 H     -5.031157   2.775102   2.980455
 C     -3.340380   1.956370   1.889110
 H     -3.473372   2.649957   1.044275
 C     -0.430302  -1.710162  -1.720219
 H     -0.521938  -2.427880  -0.880292
 C      1.044111   0.128342  -2.400406
 H      1.551632  -0.370420  -3.259812
 H      0.137841   0.619944  -2.841667
 C      1.933533   1.204855  -1.857880
```

```
 C      2.682677   2.302802   0.067481
 H      2.651945   2.369793   1.164282
 C      3.469077   3.180405  -0.681912
 H      4.063090   3.949764  -0.167412
 C      3.486189   3.053457  -2.083750
 C      2.710323   2.048682  -2.673821
 H      2.693111   1.908997  -3.765221
 C     -0.141395  -2.543178  -2.984157
 H     -0.918374  -3.322843  -3.117244
 H     -0.122583  -1.928503  -3.907373
 H      0.835775  -3.057228  -2.882557
 C      1.244142   0.544381   2.089360
 C      0.196412  -1.544872   1.280270
 H      2.041759  -1.733839  -1.298710
 H      2.730846  -1.163275   0.554846
 C      3.424246  -1.934132   0.053206
 C      3.492018  -3.169720   0.996532
 C      4.577053  -2.821374   2.045041
 O      2.966966  -2.185755  -1.239595
 H      4.098377   3.726407  -2.703459
 C      4.835638  -1.372906   0.142730
 C      5.493821  -1.874604   1.287737
 C      5.481820  -0.486765  -0.732436
 C      6.811924  -1.479778   1.572015
 C      6.805156  -0.094009  -0.448558
 H      4.961384  -0.120129  -1.632098
 C      7.463761  -0.585680   0.697235
 H      7.337974  -1.870395   2.459037
 H      7.332116   0.595451  -1.128043
 H      8.500849  -0.276308   0.905728
 H      5.106504  -3.709187   2.450189
 H      4.126082  -2.297662   2.919473
 H      3.825700  -4.027816   0.372383
 H      2.508623  -3.429903   1.435456
---------------------------------------
Mn7/viii
Frequencies, energies and thermodynamic properties:
 Lowest Vibrational Mode (1/cm) =                         23.3751
 2nd Lowest Vibrational Mode (1/cm) =                     30.1029
 E(RB-P86) (a.u.) =                                      -4252.56229993
 Thermal correction to Enthalpy (a.u.) =                  0.558242
 Thermal correction to Gibbs Free Energy (a.u.) =         0.453904
 Total Entropy (cal/Kmol) =                               219.598
 E(RPBE1PBE) (a.u.) =                                    -4251.88288022
Optimised cartesian coordinates (Angstrom):
 Fe2.939013  -0.831559   0.741325
 Mn-1.230056  -0.409562  -1.752354
 P     -0.252497   0.605071   0.013776
 O     -2.095658   2.181874  -2.902556
 O      1.156377  -0.380474  -3.475237
 N     -0.818149  -2.223105  -0.887235
 N     -3.021026  -0.755964  -0.769082
 C      0.924212  -0.471531   0.916272
 C      1.160246  -1.876683   0.601900
 C      1.990866  -2.409285   1.655754
 H      2.370401  -3.437554   1.713793
 C      2.277725  -1.363399   2.602334
 H      2.903395  -1.457240   3.500436
 C      1.630744  -0.163079   2.147716
 H      1.658285   0.811266   2.652085
 C      3.685846  -0.197198  -1.073821
 H      3.090153   0.022633  -1.968832
 C      4.233354  -1.480890  -0.725182
 H      4.132984  -2.405618  -1.309278
 C      4.915505  -1.347215   0.537279
 H      5.422847  -2.152364   1.085605
 C      4.791925   0.023076   0.966627
 H      5.189694   0.444498   1.899680
 C      4.029083   0.733703  -0.026868
 H      3.743202   1.792907   0.011510
 C     -1.476477   1.109913   1.323369
 C     -2.334757   2.201230   1.047385
 H     -2.231459   2.756715   0.101022
 C     -3.314142   2.592748   1.974837
 H     -3.968020   3.450076   1.748481
 C     -3.460440   1.892498   3.187655
 H     -4.228304   2.199828   3.915266
 C     -2.621469   0.799345   3.463920
 H     -2.728131   0.245288   4.410269
 C     -1.635018   0.408683   2.537868
 H     -0.977688  -0.442489   2.772903
 C      0.672734   2.208632  -0.217572
 C      1.173884   2.579701  -1.483810
 H      0.993346   1.940652  -2.358913
 C      1.912046   3.767132  -1.643073
```

H   2.293081   4.040162 -2.639975
C   2.158588   4.602233 -0.539578
H   2.735060   5.532468 -0.665386
C   1.655791   4.246638   0.725698
H   1.835178   4.897819   1.596083
C   0.914636   3.063089   0.884488
H   0.510687   2.811987   1.877505
C   0.562003 -2.615054 -0.605726
H   1.154667 -2.315416 -1.495602
C   -1.737768 -2.583088   0.168436
H   -1.938501 -3.684619   0.227902
H   -1.372124 -2.320371   1.206999
C   -3.051102 -1.874812   0.008029
C   -4.164622 -0.038050 -0.923318
C   -5.374416 -0.401532 -0.325593
C   -5.415389 -1.561967   0.469967
C   -4.238872 -2.302627   0.633530
C   0.738411 -4.144214 -0.465254
H   1.812554 -4.422398 -0.445405
H   0.274333 -4.540894   0.461835
H   0.267947 -4.652014 -1.331837
C   -1.761857   1.136417 -2.461871
C   0.234651 -0.376290 -2.733953
H   -1.795542 -1.726741 -2.785656
H   -2.148453 -1.085000 -3.148820
H   -4.097319   0.857659 -1.557908
H   -6.269076   0.216789 -0.489696
H   -4.223680 -3.218069   1.244338
H   -6.352069 -1.882891   0.951237

Mn7/ix
Frequencies, energies and thermodynamic properties:
Lowest Vibrational Mode (1/cm) =   19.9938
2nd Lowest Vibrational Mode (1/cm) =   25.1970
E(RB-P86) (a.u.) =   -4406.35116511
Thermal correction to Enthalpy (a.u.) =   0.627531
Thermal correction to Gibbs Free Energy (a.u.) =   0.513013
Total Entropy (cal/Kmol) =   241.023
E(RPBE1PBE) (a.u.) =   -4405.67534164
Optimised cartesian coordinates (Angstrom):
Fe-3.100952 -0.728405 -0.960445
Mn1.366527 -0.719122   0.966888
P   -0.080862   0.834927   0.193085
O   2.143069   0.888206   3.307495
O   -0.621269 -2.124038   2.618368
N   1.037156 -1.883899 -0.782021
N   2.930718 -0.047869 -0.236450
C   -1.246831   0.148441 -1.061688
C   -1.208034 -1.213544 -1.596249
C   -2.145519 -1.270559 -2.694370
H   -2.371401 -2.156408 -3.301152
C   -2.768811   0.016462 -2.839830
H   -3.545018   0.277310 -3.571705
C   -2.226994   0.888964 -1.835870
H   -2.503119   1.940194 -1.684921
C   -3.545659 -1.362816   0.949307
H   -2.817170 -1.515681   1.755864
C   -3.949162 -2.350626 -0.016305
H   -3.589193 -3.387005 -0.069053
C   -4.890764 -1.736243 -0.917071
H   -5.369116 -2.219525 -1.779505
C   -5.071540 -0.367385 -0.505329
H   -5.712306   0.375030 -0.999751
C   -4.238729 -0.135191   0.646172
H   -4.133746   0.813600   1.188246
C   0.735535   2.234966 -0.731126
C   1.472404   3.179580   0.023638
H   1.487057   3.110739   1.123864
C   2.182059   4.210191 -0.613909
H   2.741678   4.942090 -0.009624
C   2.181251   4.306696 -2.018976
H   2.739115   5.114076 -2.519355
C   1.464763   3.365593 -2.777739
H   1.457067   3.432018 -3.877574
C   0.745611   2.336460 -2.139132
H   0.176695   1.617949 -2.749288
C   -1.182223   1.800164   1.349313
C   -1.452768   1.318309   2.648305
H   -0.994292   0.382163   2.995235
C   -2.311420   2.024468   3.511098
H   -2.509013   1.632634   4.521578
C   -2.909752   3.224774   3.089617
H   -3.580193   3.778239   3.766193
C   -2.638422   3.720150   1.800696
H   -3.093993   4.664866   1.463218

```
 C     -1.778047   3.016867   0.939990
 H     -1.559444   3.430921  -0.056596
 C     -0.334379  -2.364896  -1.107904
 H     -0.736225  -2.721308  -0.138006
 C      1.808675  -1.386808  -1.933897
 H      2.121556  -2.199652  -2.624972
 H      1.155678  -0.706339  -2.526884
 C      3.013278  -0.605944  -1.473384
 C      3.961148   0.718882   0.195455
 C      5.106431   0.956869  -0.573100
 C      5.201573   0.370972  -1.846704
 C      4.138224  -0.422812  -2.298249
 C     -0.331611  -3.562083  -2.073767
 H     -1.346635  -4.001190  -2.146429
 H     -0.011296  -3.280874  -3.097666
 H      0.348262  -4.353767  -1.698004
 C      1.835586   0.232547   2.369512
 C      0.148247  -1.535813   1.934782
 H      1.596334  -2.616501  -0.273471
 H      4.169171  -0.904324  -3.287404
 H      5.911724   1.587089  -0.167866
 H      6.091288   0.526871  -2.475938
 O      2.641155  -2.286083   1.140158
 C      2.868459  -2.954004   2.343113
 H      1.928080  -3.384140   2.789775
 H      3.273477  -2.263566   3.134273
 C      3.870747  -4.100263   2.145294
 H      4.837080  -3.714651   1.755053
 H      4.070090  -4.632834   3.100497
 H      3.482371  -4.840266   1.412497
 H      3.861203   1.148416   1.202910
---------------------------------------
Mn7/x
Frequencies, energies and thermodynamic properties:
 Lowest Vibrational Mode (1/cm) =                         15.2679
 2nd Lowest Vibrational Mode (1/cm) =                     24.0086
 E(RB-P86) (a.u.) =                                      -4561.29174882
 Thermal correction to Enthalpy (a.u.) =                  0.711699
 Thermal correction to Gibbs Free Energy (a.u.) =         0.585348
 Total Entropy (cal/Kmol) =                               265.928
 E(RPBE1PBE) (a.u.) =                                    -4560.62416478
Optimised cartesian coordinates (Angstrom):
 Fe-3.107117  -1.523989  -0.789165
 Mn1.226354  -0.010541   0.908054
 P     -0.659890   0.936847   0.085223
 O      1.347180   2.056443   2.997000
 O     -0.128228  -1.858097   2.752196
 N      1.217384  -1.397843  -0.742167
 N      2.472916   0.970527  -0.434083
 C     -1.606429  -0.151205  -1.056854
 C     -1.174114  -1.480617  -1.484197
 C     -2.079494  -1.911884  -2.524467
 H     -2.044826  -2.875497  -3.047861
 C     -3.064143  -0.886307  -2.736483
 H     -3.901966  -0.934735  -3.444889
 C     -2.783792   0.194638  -1.833081
 H     -3.358050   1.125997  -1.749405
 C     -3.278579  -2.102438   1.180778
 H     -2.509625  -1.974082   1.953360
 C     -3.413280  -3.239839   0.309568
 H     -2.770422  -4.130365   0.305912
 C     -4.525161  -2.996368  -0.573464
 H     -4.873950  -3.664984  -1.371873
 C     -5.080119  -1.708039  -0.245117
 H     -5.926405  -1.223221  -0.750014
 C     -4.308634  -1.153583   0.836760
 H     -4.462204  -0.172991   1.305421
 C     -0.329145   2.448506  -0.956297
 C      0.119485   3.618506  -0.296704
 H      0.196064   3.634030   0.802695
 C      0.461250   4.767308  -1.028654
 H      0.799081   5.671615  -0.497616
 C      0.374649   4.763628  -2.434228
 H      0.643716   5.664345  -3.008432
 C     -0.057480   3.603408  -3.098872
 H     -0.130483   3.590006  -4.198172
 C     -0.408164   2.452965  -2.365822
 H     -0.761693   1.559579  -2.903361
 C     -1.961244   1.607199   1.243008
 C     -2.048948   1.146801   2.574511
 H     -1.328856   0.408394   2.952728
 C     -3.058577   1.620982   3.432375
 H     -3.109328   1.250139   4.468433
 C     -3.994177   2.564357   2.973077
 H     -4.783258   2.936278   3.645764
```

```
 C     -3.910208   3.037480   1.650528
 H     -4.632480   3.783389   1.282097
 C     -2.899249   2.567231   0.794634
 H     -2.835858   2.964515  -0.230289
 C      0.011512  -2.269800  -0.948657
 H     -0.239588  -2.628517   0.069402
 C      1.689862  -0.736034  -1.977068
 H      2.160279  -1.447603  -2.689733
 H      0.812478  -0.298094  -2.504596
 C      2.646158   0.379309  -1.646336
 C      3.260465   2.024894  -0.111731
 C      4.244722   2.528953  -0.970156
 C      4.437156   1.911885  -2.217292
 C      3.625308   0.819740  -2.555096
 C      0.339534  -3.508135  -1.800664
 H     -0.504944  -4.225644  -1.779993
 H      0.540390  -3.252691  -2.861257
 H      1.230300  -4.017504  -1.381432
 C      1.311634   1.217257   2.161930
 C      0.367281  -1.096042   1.994341
 H      3.215622  -2.244830   0.800103
 H      1.980238  -2.056686  -0.439939
 O      3.270685  -3.128817   0.220068
 C      4.515570  -3.147413  -0.463752
 H      4.386170  -3.724520  -1.409543
 H      4.825912  -2.115934  -0.765681
 H      3.740186   0.303228  -3.519932
 H      4.852586   3.389414  -0.654130
 H      3.090589   2.477443   0.875619
 C      5.630557  -3.790764   0.365991
 H      5.348987  -4.823260   0.662557
 H      6.580587  -3.842490  -0.208289
 H      5.823501  -3.211025   1.293745
 H      5.208889   2.273286  -2.914118
 O      3.000577  -0.947824   1.407386
 C      3.489449  -0.996412   2.728001
 C      4.477522   0.130504   3.058185
 H      4.014771  -1.976015   2.881323
 H      2.662835  -0.983162   3.483713
 H      4.895417   0.002451   4.081011
 H      5.323476   0.133843   2.338201
 H      3.985530   1.123976   3.015453
---------------------------------------
Mn7/TS-i
Frequencies, energies and thermodynamic properties:
 Lowest Vibrational Mode (1/cm) =-748.4830
 2nd Lowest Vibrational Mode (1/cm) =                     20.2612
 E(RB-P86) (a.u.) =                                      -4407.50399496
 Thermal correction to Enthalpy (a.u.) =                  0.639186
 Thermal correction to Gibbs Free Energy (a.u.) =         0.523276
 Total Entropy (cal/Kmol) =                               243.952
 E(RPBE1PBE) (a.u.) =                                    -4406.82702017
Optimised cartesian coordinates (Angstrom):
 Fe-2.902525  -1.261186  -0.959042
 Mn1.315847  -0.227590   1.257345
 P     -0.295405   0.921578   0.138927
 O      1.473818   1.859894   3.342092
 O     -0.522519  -1.777306   2.957743
 N      1.362388  -1.644520  -0.297274
 N      2.869177   0.522864   0.115577
 C     -1.210944  -0.105627  -1.071908
 C     -0.899287  -1.499522  -1.378380
 C     -1.708042  -1.873051  -2.515160
 H     -1.728945  -2.862498  -2.989118
 C     -2.516772  -0.748957  -2.903789
 H     -3.252248  -0.734518  -3.719392
 C     -2.222783   0.340127  -2.014046
 H     -2.677675   1.338234  -2.048597
 C     -3.415367  -1.701376   0.988486
 H     -2.751470  -1.622150   1.858637
 C     -3.569260  -2.862630   0.153305
 H     -3.047280  -3.820979   0.277374
 C     -4.508534  -2.539953  -0.890468
 H     -4.824584  -3.207210  -1.703535
 C     -4.937985  -1.178088  -0.697178
 H     -5.639444  -0.626480  -1.337439
 C     -4.260336  -0.657898   0.461881
 H     -4.355385   0.358330   0.865899
 C      0.391322   2.311457  -0.892202
 C      0.913378   3.440588  -0.216155
 H      0.853455   3.500540   0.882754
 C      1.499783   4.495697  -0.933913
 H      1.893060   5.370968  -0.392707
 C      1.585912   4.434866  -2.338030
 H      2.046163   5.262336  -2.900884
```

```
 C      1.081228   3.312513  -3.016550
 H      1.142678   3.255952  -4.115089
 C      0.487137   2.256070  -2.299405
 H      0.083513   1.391040  -2.847679
 C     -1.626524   1.822938   1.083127
 C     -1.952920   1.455425   2.406334
 H     -1.400473   0.650750   2.910469
 C     -2.990110   2.109791   3.096307
 H     -3.228774   1.809976   4.129042
 C     -3.714397   3.141869   2.474882
 H     -4.524835   3.654836   3.016607
 C     -3.391182   3.522093   1.159232
 H     -3.946219   4.335538   0.665363
 C     -2.352579   2.872047   0.470771
 H     -2.099224   3.197040  -0.550260
 C      0.117659  -2.375283  -0.648965
 H     -0.326819  -2.668092   0.324194
 C      2.063757  -1.120427  -1.474368
 H      2.541867  -1.921549  -2.085096
 H      1.356582  -0.602080  -2.171386
 C      3.101202  -0.112870  -1.067066
 C      3.734651   1.491234   0.512916
 C      4.859477   1.856053  -0.233718
 C      5.112689   1.192127  -1.446584
 C      4.220202   0.195773  -1.862942
 C      0.408973  -3.684272  -1.410900
 H     -0.507314  -4.303599  -1.492421
 H      0.784985  -3.503029  -2.438991
 H      1.168009  -4.268688  -0.852988
 C      1.421132   1.022510   2.511070
 C      0.168695  -1.139182   2.242693
 H      2.632630  -0.858350   2.145486
 H      2.507429  -1.616642   1.760723
 H      2.143302  -2.502094   0.331444
 O      2.783120  -3.171048   1.099649
 C      4.144129  -3.265306   0.752665
 H      4.276991  -3.477606  -0.341694
 H      4.688899  -2.297518   0.935107
 H      4.378483  -0.351356  -2.804408
 H      5.526631   2.645896   0.141194
 H      3.513458   1.981283   1.472073
 C      4.839370  -4.369880   1.553656
 H      4.353505  -5.351175   1.366058
 H      5.913411  -4.455578   1.281567
 H      4.774324  -4.164613   2.643601
 H      5.993476   1.446170  -2.055944
---------------------------------------
Mn7/TS-ii_si
Frequencies, energies and thermodynamic properties:
 Lowest Vibrational Mode (1/cm) =-245.8578
 2nd Lowest Vibrational Mode (1/cm) =                     12.7831
 E(RB-P86) (a.u.) =                                     -4675.30344377
 Thermal correction to Enthalpy (a.u.) =                 0.712351
 Thermal correction to Gibbs Free Energy (a.u.) =        0.589178
 Total Entropy (cal/Kmol) =                              259.239
 E(RPBE1PBE) (a.u.) =                                   -4674.61294299
Optimised cartesian coordinates (Angstrom):
 Fe-3.109463  -1.736164  -1.058360
 Mn1.087275   0.255866   0.320028
 P     -1.105335   0.773625   0.523708
 O      1.855422   0.939248   3.076933
 O      0.911214  -2.566229   1.128373
 N      0.842909  -0.124664  -1.737341
 N      1.659039   2.066156  -0.455748
 C     -2.124173   0.048810  -0.827744
 C     -1.585677  -0.698124  -1.965306
 C     -2.676196  -0.914114  -2.889094
 H     -2.615700  -1.459809  -3.838969
 C     -3.871114  -0.331677  -2.342264
 H     -4.868270  -0.357400  -2.801811
 C     -3.539923   0.255064  -1.073901
 H     -4.237846   0.776036  -0.406176
 C     -2.463089  -3.151651   0.291745
 H     -1.518831  -3.115623   0.850281
 C     -2.648599  -3.743142  -1.007083
 H     -1.874661  -4.241616  -1.606229
 C     -4.021987  -3.546705  -1.395232
 H     -4.476539  -3.862984  -2.343667
 C     -4.686894  -2.836007  -0.332942
 H     -5.737494  -2.515967  -0.330516
 C     -3.723891  -2.589270   0.708615
 H     -3.908925  -2.050374   1.646854
 C     -1.464373   2.597366   0.391259
 C     -1.045320   3.425362   1.461246
 H     -0.593914   2.972913   2.359616
```

```
 C     -1.205928   4.818940   1.395141
 H     -0.883827   5.446012   2.241928
 C     -1.773150   5.413045   0.251108
 H     -1.897992   6.506241   0.198444
 C     -2.177118   4.601786  -0.822922
 H     -2.621940   5.056288  -1.722740
 C     -2.024489   3.203172  -0.754714
 H     -2.362220   2.582515  -1.599154
 C     -2.046710   0.353693   2.077380
 C     -1.562900  -0.642201   2.952682
 H     -0.615778  -1.153742   2.730661
 C     -2.285033  -0.992895   4.108416
 H     -1.891621  -1.772364   4.780222
 C     -3.498974  -0.349612   4.407545
 H     -4.062902  -0.622561   5.313684
 C     -3.984836   0.652374   3.546960
 H     -4.930361   1.169063   3.777092
 C     -3.262395   1.004786   2.393690
 H     -3.645906   1.805429   1.742013
 C     -0.148520  -1.167267  -2.165100
 H      0.040330  -2.016708  -1.478781
 C      0.741734   1.137641  -2.500456
 H      1.090854   1.029688  -3.549960
 H     -0.329402   1.438547  -2.550539
 C      1.508690   2.226583  -1.802965
 C      2.268094   3.065563   0.238809
 H      2.370617   2.911561   1.322444
 C      2.755084   4.228966  -0.366731
 H      3.238892   4.998103   0.253165
 C      2.621302   4.382602  -1.757188
 C      1.988750   3.360593  -2.479874
 H      1.856164   3.433238  -3.569910
 C      0.119243  -1.665831  -3.595751
 H     -0.496798  -2.560888  -3.813423
 H     -0.113863  -0.900682  -4.364109
 H      1.183579  -1.958494  -3.700487
 C      1.525897   0.669695   1.970021
 C      0.925945  -1.428343   0.796127
 H      1.806877  -0.510369  -1.948081
 H      2.654951  -0.198344   0.056717
 C      3.933481  -0.654008  -0.950660
 C      4.317420  -1.829752  -0.091097
 C      4.848092   0.502272  -0.476976
 C      5.134665  -1.397354   0.977066
 C      4.021627  -3.188619  -0.285944
 C      5.292249   0.113121   0.950036
 H      5.721042   0.498298  -1.169913
 C      5.663008  -2.337872   1.876660
 C      4.554742  -4.128373   0.614231
 H      3.392437  -3.500420  -1.135037
 H      6.321683   0.442140   1.201770
 C      5.367604  -3.703692   1.687974
 H      6.306511  -2.019438   2.713398
 H      4.344728  -5.201891   0.481064
 H      5.782727  -4.451185   2.383325
 O      3.448360  -0.745655  -2.113663
 H      4.368617   1.495791  -0.559372
 H      4.614802   0.569610   1.707264
 H      3.002552   5.279740  -2.268172
---------------------------------------
Mn7/TS-ii_re
Frequencies, energies and thermodynamic properties:
 Lowest Vibrational Mode (1/cm) =-246.9177
 2nd Lowest Vibrational Mode (1/cm) =                     14.5925
 E(RB-P86) (a.u.) =                                      -4675.30396284
 Thermal correction to Enthalpy (a.u.) =                  0.712461
 Thermal correction to Gibbs Free Energy (a.u.) =         0.589480
 Total Entropy (cal/Kmol) =                               258.836
 E(RPBE1PBE) (a.u.) =                                    -4674.61436103
Optimised cartesian coordinates (Angstrom):
 Fe-3.642701  -1.202629  -0.855907
 Mn0.923649  -0.272107   0.489268
 P     -1.005852   0.902787   0.354360
 O      1.742051   0.772806   3.116173
 O     -0.145069  -2.652645   1.842700
 N      0.636066  -1.031042  -1.453503
 N      2.055107   1.055236  -0.593148
 C     -2.159464   0.214858  -0.904808
 C     -1.842578  -0.899049  -1.799413
 C     -2.914803  -0.990632  -2.764157
 H     -2.995155  -1.733634  -3.567442
 C     -3.887211   0.028152  -2.476213
 H     -4.827430   0.192483  -3.019512
 C     -3.432375   0.766966  -1.331579
 H     -3.955535   1.610005  -0.862453
```

```
 C     -3.513929  -2.407541   0.811458
 H     -2.623192  -2.535971   1.440140
 C     -3.842000  -3.189705  -0.351166
 H     -3.250037  -4.020024  -0.759257
 C     -5.071688  -2.678583  -0.900845
 H     -5.577131  -3.046022  -1.803989
 C     -5.505812  -1.581159  -0.074575
 H     -6.400598  -0.965698  -0.238165
 C     -4.542489  -1.411158   0.981838
 H     -4.572702  -0.646289   1.768566
 C     -0.778427   2.665910  -0.204111
 C     -0.154503   3.563052   0.696914
 H      0.109208   3.224603   1.712494
 C      0.126029   4.884759   0.313849
 H      0.602118   5.571503   1.031984
 C     -0.196234   5.329943  -0.982804
 H      0.025199   6.366141  -1.283995
 C     -0.800680   4.442933  -1.889825
 H     -1.056879   4.780969  -2.906786
 C     -1.090588   3.119564  -1.504456
 H     -1.579224   2.443677  -2.223241
 C     -2.074701   1.155909   1.860864
 C     -1.954407   0.290429   2.969316
 H     -1.211266  -0.519270   2.954066
 C     -2.781768   0.450365   4.096232
 H     -2.673161  -0.234147   4.952588
 C     -3.738141   1.480448   4.132382
 H     -4.383826   1.607117   5.015925
 C     -3.859397   2.354866   3.036381
 H     -4.599760   3.170537   3.058328
 C     -3.031272   2.197115   1.911419
 H     -3.125107   2.902043   1.070550
 C     -0.617385  -1.804283  -1.744330
 H     -0.723778  -2.480803  -0.873328
 C      0.954171  -0.011208  -2.475531
 H      1.295481  -0.460456  -3.433242
 H      0.027710   0.562073  -2.706540
 C      1.986596   0.948940  -1.951400
 C      2.917074   1.966735  -0.068753
 H      2.954278   2.024690   1.028320
 C      3.724725   2.793935  -0.856541
 H      4.397543   3.514447  -0.368814
 C      3.663227   2.676238  -2.255153
 C      2.783743   1.731701  -2.803999
 H      2.698741   1.599293  -3.893197
 C     -0.472293  -2.688359  -2.994862
 H     -1.329522  -3.386142  -3.074850
 H     -0.429053  -2.097222  -3.932366
 H      0.450574  -3.298172  -2.917951
 C      1.403272   0.365893   2.055586
 C      0.229261  -1.671363   1.288222
 H      1.433365  -1.729939  -1.475670
 H      2.298291  -1.193689   0.472013
 C      3.325365  -2.332998  -0.236769
 C      4.564131  -1.535947   0.072122
 C      3.194915  -3.338696   0.933916
 C      4.978893  -1.788252   1.399556
 C      5.309139  -0.710314  -0.785840
 C      3.998885  -2.716241   2.096123
 C      6.157017  -1.198617   1.886231
 C      6.490150  -0.123879  -0.296079
 H      4.971137  -0.544354  -1.821418
 C      6.907965  -0.365964   1.030781
 H      6.499261  -1.388510   2.916972
 H      7.098483   0.521528  -0.949962
 H      7.838347   0.095377   1.399775
 O      2.879298  -2.534239  -1.402008
 H      4.501863  -3.464844   2.742535
 H      3.328447  -2.120238   2.756585
 H      2.146264  -3.590247   1.177167
 H      3.690359  -4.271528   0.578024
 H      4.290530   3.304637  -2.905597
---------------------------------------
Mn7/TS-iii
Frequencies, energies and thermodynamic properties:
 Lowest Vibrational Mode (1/cm) =-635.0178
 2nd Lowest Vibrational Mode (1/cm) =                       26.0262
 E(RB-P86) (a.u.) =                                       -4252.55916938
 Thermal correction to Enthalpy (a.u.) =                    0.557285
 Thermal correction to Gibbs Free Energy (a.u.) =           0.454058
 Total Entropy (cal/Kmol) =                                 217.259
 E(RPBE1PBE) (a.u.) =                                     -4251.87662437
Optimised cartesian coordinates (Angstrom):
 Fe2.925876  -0.824346   0.742419
 Mn-1.213095  -0.449467  -1.735452
```

```
 P     -0.257116   0.639994   0.015126
 O     -2.167456   1.975664  -3.131911
 O      1.173626  -0.416996  -3.455349
 N     -0.835999  -2.294650  -0.829359
 N     -3.015332  -0.767010  -0.755152
 C      0.913227  -0.451154   0.916556
 C      1.146541  -1.865866   0.621734
 C      1.976297  -2.382932   1.685228
 H      2.353104  -3.410937   1.759730
 C      2.268258  -1.324551   2.614426
 H      2.896773  -1.406880   3.511520
 C      1.623597  -0.130717   2.142759
 H      1.656232   0.851506   2.631290
 C      3.678592  -0.210795  -1.077818
 H      3.085396   0.003595  -1.975630
 C      4.218856  -1.494181  -0.716203
 H      4.114979  -2.423745  -1.291941
 C      4.900293  -1.352076   0.545480
 H      5.402396  -2.154641   1.102327
 C      4.783207   0.022753   0.961719
 H      5.181334   0.450676   1.891635
 C      4.025463   0.727926  -0.039427
 H      3.744486   1.788758  -0.010940
 C     -1.466043   1.170238   1.328934
 C     -2.333388   2.248087   1.028297
 H     -2.238636   2.777604   0.065994
 C     -3.310725   2.658924   1.949514
 H     -3.971375   3.505467   1.702978
 C     -3.446982   1.991006   3.181643
 H     -4.213613   2.312800   3.904274
 C     -2.599996   0.910574   3.482682
 H     -2.698998   0.381163   4.443898
 C     -1.615828   0.500889   2.562401
 H     -0.953453  -0.340872   2.816991
 C      0.682834   2.230186  -0.237465
 C      1.162055   2.583582  -1.517238
 H      0.960776   1.933901  -2.380208
 C      1.903811   3.764822  -1.704031
 H      2.268299   4.024271  -2.710720
 C      2.174199   4.611482  -0.614929
 H      2.753229   5.537012  -0.762018
 C      1.691549   4.274286   0.663345
 H      1.889385   4.935514   1.522107
 C      0.947468   3.096613   0.849770
 H      0.558815   2.859610   1.852397
 C      0.556377  -2.656339  -0.556811
 H      1.126950  -2.375728  -1.466696
 C     -1.754848  -2.579004   0.254499
 H     -1.957985  -3.671519   0.386851
 H     -1.364170  -2.240261   1.256665
 C     -3.063270  -1.865780   0.049572
 C     -4.153403  -0.049629  -0.942395
 C     -5.372646  -0.390878  -0.348686
 C     -5.430724  -1.529710   0.474032
 C     -4.260087  -2.273596   0.668859
 C      0.750352  -4.177218  -0.369589
 H      1.827238  -4.442279  -0.338864
 H      0.287190  -4.549889   0.567704
 H      0.289471  -4.716603  -1.221865
 C     -1.785554   1.006456  -2.573872
 C      0.254007  -0.418251  -2.710589
 H     -1.498201  -2.021978  -2.253729
 H     -1.888947  -1.492427  -2.903250
 H     -4.076194   0.826877  -1.601758
 H     -6.261137   0.228848  -0.538995
 H     -4.258153  -3.174929   1.300323
 H     -6.374840  -1.833776   0.951735
---------------------------------------
Mn8/i
Frequencies, energies and thermodynamic properties:
 Lowest Vibrational Mode (1/cm) =                         20.5339
 2nd Lowest Vibrational Mode (1/cm) =                     23.3104
 E(RB-P86) (a.u.) =                                    -4385.28012296
 Thermal correction to Enthalpy (a.u.) =                  0.617252
 Thermal correction to Gibbs Free Energy (a.u.) =         0.503162
 Total Entropy (cal/Kmol) =                               240.122
 E(RPBE1PBE) (a.u.) =                                  -4384.59363489
Optimised cartesian coordinates (Angstrom):
 Fe-3.232121  -1.347123  -0.745581
 Mn0.616084   0.007320   1.711939
 P     -0.443506   0.788501  -0.028301
 O      1.165544   2.602093   3.042726
 O     -1.721402  -0.279605   3.467927
 N      0.621290  -1.787697   1.069655
 N      2.564314  -0.097972   1.034741
```

```
 C     -1.337247  -0.569982  -0.896771
 C     -1.281451  -1.975838  -0.505567
 C     -1.941505  -2.734373  -1.541308
 H     -2.091220  -3.821734  -1.544199
 C     -2.407490  -1.827207  -2.557185
 H     -2.965191  -2.102976  -3.462607
 C     -2.045140  -0.493226  -2.162099
 H     -2.259836   0.423265  -2.726663
 C     -4.139597  -0.802287   1.022120
 H     -3.620020  -0.431470   1.915227
 C     -4.410748  -2.183091   0.721230
 H     -4.142653  -3.044829   1.347328
 C     -5.071966  -2.240591  -0.557987
 H     -5.393608  -3.152826  -1.078306
 C     -5.212474  -0.892207  -1.046631
 H     -5.660368  -0.597233  -2.005165
 C     -4.634206  -0.003305  -0.072084
 H     -4.564266   1.089205  -0.153391
 C      0.736862   1.409288  -1.326940
 C      1.358949   2.664552  -1.123489
 H      1.079814   3.279851  -0.252458
 C      2.327808   3.136913  -2.023521
 H      2.794375   4.121073  -1.856652
 C      2.704466   2.355248  -3.133318
 H      3.465744   2.725263  -3.838385
 C      2.104850   1.100134  -3.333959
 H      2.394082   0.481025  -4.198392
 C      1.126466   0.628386  -2.437409
 H      0.655227  -0.350628  -2.614297
 C     -1.665206   2.189675   0.055163
 C     -2.259912   2.540137   1.286361
 H     -1.981048   2.001456   2.203316
 C     -3.212105   3.574108   1.351534
 H     -3.666058   3.835835   2.320610
 C     -3.579350   4.273487   0.188199
 H     -4.323097   5.084489   0.240471
 C     -2.984304   3.938224  -1.042274
 H     -3.258747   4.486680  -1.957585
 C     -2.031251   2.907040  -1.108211
 H     -1.559843   2.669607  -2.074792
 C     -0.628184  -2.511439   0.779302
 H     -1.314139  -2.267985   1.615242
 C      1.739242  -2.212312   0.243196
 H      2.073613  -3.252066   0.475583
 H      1.467767  -2.236546  -0.847110
 C      2.894650  -1.268935   0.415076
 C      3.563502   0.815354   1.191521
 H      3.287648   1.757338   1.687864
 C      4.872634   0.612641   0.776432
 H      5.604906   1.410626   0.953768
 C      5.240650  -0.616441   0.139503
 C      4.187390  -1.563066  -0.026976
 H      4.364430  -2.536644  -0.503142
 C     -0.485400  -4.045535   0.758553
 H     -1.485218  -4.524487   0.732844
 H      0.083682  -4.413348  -0.119773
 H      0.029466  -4.390256   1.678718
 C      0.937343   1.565222   2.512601
 C     -0.817235  -0.161863   2.705135
 N      6.520504  -0.863941  -0.277160
 C      6.845900  -2.131650  -0.922982
 H      6.258288  -2.281983  -1.855443
 H      6.653375  -2.998639  -0.252745
 H      7.917406  -2.138963  -1.190963
 C      7.566259   0.133672  -0.069266
 H      7.709441   0.363916   1.009510
 H      7.340811   1.086308  -0.597412
 H      8.521774  -0.255661  -0.463448
---------------------------------------
Mn8/ii
Frequencies, energies and thermodynamic properties:
 Lowest Vibrational Mode (1/cm) =                       19.1749
 2nd Lowest Vibrational Mode (1/cm) =                   20.9382
 E(RB-P86) (a.u.) =                                    -4540.21595592
 Thermal correction to Enthalpy (a.u.) =                0.701926
 Thermal correction to Gibbs Free Energy (a.u.) =       0.572893
 Total Entropy (cal/Kmol) =                             271.573
 E(RPBE1PBE) (a.u.) =                                  -4539.53827654
Optimised cartesian coordinates (Angstrom):
 Fe3.337222   0.902883  -1.336994
 Mn-0.582581   0.431397   1.346026
 P      0.725931  -0.945262   0.266798
 O     -0.942136  -1.276168   3.742041
 O      1.541018   1.845634   2.802750
 N     -0.705899   1.693465  -0.117698
```

N	-2.442421	-0.074983	0.620411
C	1.551701	-0.086782	-1.135738
C	1.321848	1.308447	-1.503038
C	1.994694	1.534832	-2.759791
H	2.035622	2.488032	-3.302323
C	2.638543	0.315019	-3.170208
H	3.246925	0.178601	-4.074606
C	2.376185	-0.684670	-2.171167
H	2.731730	-1.722794	-2.194355
C	4.137539	1.404508	0.494570
H	3.584717	1.443384	1.442054
C	4.287759	2.492492	-0.434955
H	3.877529	3.504368	-0.315770
C	5.049191	2.015839	-1.561641
H	5.317626	2.598969	-2.452792
C	5.372768	0.631553	-1.325892
H	5.931628	-0.024795	-2.006503
C	4.807088	0.252136	-0.056892
H	4.858652	-0.743269	0.403140
C	-0.253898	-2.288161	-0.569132
C	-0.773760	-3.332378	0.233055
H	-0.535711	-3.368319	1.308712
C	-1.589446	-4.327143	-0.329688
H	-1.977653	-5.139020	0.305993
C	-1.912602	-4.286180	-1.700176
H	-2.553420	-5.066127	-2.141240
C	-1.414605	-3.243968	-2.500617
H	-1.663186	-3.203184	-3.573258
C	-0.589382	-2.249679	-1.940301
H	-0.194961	-1.447010	-2.582017
C	2.069791	-1.932581	1.091595
C	2.576848	-1.539908	2.349000
H	2.160372	-0.661071	2.860946
C	3.619130	-2.263760	2.956885
H	4.002308	-1.944169	3.939029
C	4.165688	-3.390784	2.318181
H	4.980256	-3.958017	2.795941
C	3.659565	-3.796301	1.069230
H	4.074572	-4.683786	0.565318
C	2.616226	-3.076066	0.462237
H	2.216880	-3.418816	-0.505152
C	0.506708	2.320018	-0.687195
H	1.124513	2.606081	0.187783
C	-1.734856	1.430287	-1.118791
H	-2.190374	2.364091	-1.526016
H	-1.309092	0.901813	-2.012880
C	-2.809113	0.559905	-0.532446
C	-3.364278	-0.905447	1.184736
C	-4.630727	-1.127138	0.662412
C	-5.036230	-0.462724	-0.540420
C	-4.065582	0.405373	-1.122836
C	0.225584	3.618085	-1.468427
H	1.172924	4.149284	-1.692052
H	-0.293524	3.437279	-2.432078
H	-0.402868	4.282954	-0.841837
C	-0.796136	-0.591156	2.785986
C	0.730154	1.253383	2.169595
H	-1.359258	3.033138	0.961967
O	-1.643792	3.854223	1.467821
C	-3.056107	3.972228	1.379488
H	-3.568120	3.035754	1.717214
H	-3.389553	4.145217	0.323593
H	-4.279550	0.965238	-2.042866
H	-5.301846	-1.812536	1.195494
H	-3.059196	-1.412460	2.111844
N	-6.274602	-0.647437	-1.089206
C	-6.644759	0.058117	-2.312689
H	-5.972163	-0.204194	-3.158710
H	-7.674805	-0.221843	-2.596220
H	-6.613400	1.161767	-2.177643
C	-7.235663	-1.542658	-0.449964
H	-7.496358	-1.204243	0.576977
H	-8.163951	-1.563576	-1.047963
H	-6.848227	-2.582800	-0.382747
C	-3.521187	5.138112	2.245825
H	-4.622794	5.260971	2.191200
H	-3.241476	4.973418	3.307769
H	-3.049301	6.086794	1.913102

Mn8/iii

Frequencies, energies and thermodynamic properties:

Lowest Vibrational Mode (1/cm) =	19.8927
2nd Lowest Vibrational Mode (1/cm) =	21.3459
E(RB-P86) (a.u.) =	-4541.38834165
Thermal correction to Enthalpy (a.u.) =	0.717942

```
 Thermal correction to Gibbs Free Energy (a.u.) =         0.589739
 Total Entropy (cal/Kmol) =                               269.825
 E(RPBE1PBE) (a.u.) =                                    -4540.70719891
Optimised cartesian coordinates (Angstrom):
 Fe3.338503   0.923884  -1.304210
 Mn-0.588151   0.442993   1.546737
 P      0.676200  -0.905404   0.231377
 O     -0.655963  -1.619493   3.668328
 O      1.664709   1.779779   2.889908
 N     -0.711851   1.821602  -0.022747
 N     -2.378652  -0.133714   0.680703
 C      1.522644  -0.018984  -1.128378
 C      1.332515   1.396036  -1.430406
 C      2.001485   1.650898  -2.684852
 H      2.067492   2.623259  -3.189232
 C      2.607363   0.431842  -3.150785
 H      3.206080   0.315995  -4.064420
 C      2.324878  -0.599816  -2.191118
 H      2.649553  -1.646089  -2.257924
 C      4.188303   1.328822   0.530858
 H      3.658600   1.341342   1.491827
 C      4.345043   2.451354  -0.354983
 H      3.961393   3.466338  -0.184931
 C      5.071096   2.004771  -1.516793
 H      5.334500   2.618291  -2.388851
 C      5.366376   0.604508  -1.345841
 H      5.895353  -0.035171  -2.065111
 C      4.818155   0.185225  -0.082007
 H      4.856510  -0.828942   0.336320
 C     -0.320289  -2.212189  -0.646283
 C     -0.873953  -3.256884   0.132067
 H     -0.669294  -3.304798   1.213948
 C     -1.677040  -4.243617  -0.462803
 H     -2.093355  -5.054179   0.156446
 C     -1.949975  -4.196763  -1.843363
 H     -2.579861  -4.970707  -2.309981
 C     -1.414270  -3.156582  -2.621762
 H     -1.621207  -3.111246  -3.702980
 C     -0.604186  -2.169320  -2.028113
 H     -0.182202  -1.368188  -2.653985
 C      2.015131  -1.957894   0.993333
 C      2.588789  -1.614269   2.236485
 H      2.227101  -0.736306   2.789043
 C      3.630841  -2.386104   2.782734
 H      4.064003  -2.102874   3.755189
 C      4.113697  -3.513555   2.095791
 H      4.927963  -4.118471   2.525360
 C      3.543528  -3.869734   0.859725
 H      3.908149  -4.756002   0.316265
 C      2.499676  -3.101774   0.315493
 H      2.051267  -3.407099  -0.642662
 C      0.532898   2.390550  -0.583205
 H      1.158565   2.653295   0.295024
 C     -1.626033   1.357465  -1.065663
 H     -2.081437   2.195739  -1.646659
 H     -1.108831   0.722431  -1.836243
 C     -2.718747   0.509871  -0.473308
 C     -3.307035  -0.963610   1.225221
 C     -4.567257  -1.183878   0.681835
 C     -4.951007  -0.510218  -0.520953
 C     -3.967682   0.358642  -1.082138
 C      0.285033   3.709555  -1.348952
 H      1.243612   4.211305  -1.594309
 H     -0.262524   3.550167  -2.301180
 H     -0.308689   4.393563  -0.709986
 C     -0.641797  -0.786950   2.830000
 C      0.799688   1.220131   2.308650
 H     -1.687533   1.186545   2.659945
 H     -1.501043   1.836127   2.179040
 H     -1.399314   3.052010   0.749018
 O     -1.781609   3.820694   1.356651
 C     -3.158913   3.998905   1.088147
 H     -3.338836   4.258697   0.012081
 H     -3.743014   3.059798   1.276091
 H     -4.165716   0.923468  -2.002495
 H     -5.246434  -1.872491   1.200202
 H     -3.018635  -1.475387   2.155629
 N     -6.181652  -0.685359  -1.091409
 C     -7.157008  -1.577888  -0.471263
 H     -7.431399  -1.243414   0.553606
 H     -8.076447  -1.591131  -1.083081
 H     -6.776268  -2.620546  -0.402943
 C     -6.530886   0.033388  -2.313166
 H     -6.502918   1.135777  -2.166473
 H     -5.843685  -0.219254  -3.150366
```

```
 H     -7.555551  -0.244358  -2.617907
 C     -3.719221   5.115565   1.966609
 H     -3.180168   6.068244   1.777997
 H     -4.798634   5.281926   1.766622
 H     -3.600494   4.867034   3.042645
---------------------------------------
Mn8/iv
Frequencies, energies and thermodynamic properties:
 Lowest Vibrational Mode (1/cm) =                         16.5154
 2nd Lowest Vibrational Mode (1/cm) =                     21.4322
 E(RB-P86) (a.u.) =                                     -4541.41561080
 Thermal correction to Enthalpy (a.u.) =                  0.722542
 Thermal correction to Gibbs Free Energy (a.u.) =         0.593728
 Total Entropy (cal/Kmol) =                               271.112
 E(RPBE1PBE) (a.u.) =                                   -4540.73310564
Optimised cartesian coordinates (Angstrom):
 Fe3.385337   0.747555  -1.377321
 Mn-0.625040   0.631294   1.379784
 P      0.683275  -0.893060   0.311623
 O     -0.888596  -0.970631   3.830483
 O      1.483786   2.232226   2.657632
 N     -0.699060   1.805851  -0.426153
 N     -2.403386  -0.028331   0.542238
 C      1.579459  -0.201286  -1.142399
 C      1.391152   1.155705  -1.656121
 C      2.110199   1.243334  -2.907349
 H      2.188703   2.133917  -3.543322
 C      2.746299  -0.019394  -3.166943
 H      3.385845  -0.252720  -4.028818
 C      2.428745  -0.907774  -2.083855
 H      2.767895  -1.946951  -1.985729
 C      4.124758   1.445279   0.414437
 H      3.535719   1.605134   1.327130
 C      4.335944   2.413857  -0.629092
 H      3.944745   3.440078  -0.646453
 C      5.130870   1.794990  -1.659105
 H      5.446393   2.264147  -2.600689
 C      5.414215   0.442996  -1.249716
 H      5.984098  -0.298860  -1.825169
 C      4.791175   0.225510   0.030021
 H      4.801686  -0.709676   0.604555
 C     -0.251607  -2.330113  -0.425702
 C     -0.864266  -3.235172   0.475346
 H     -0.714157  -3.110794   1.560512
 C     -1.657253  -4.293628   0.003040
 H     -2.116998  -4.993185   0.719645
 C     -1.867874  -4.458628  -1.379791
 H     -2.491383  -5.287593  -1.750823
 C     -1.278887  -3.556404  -2.282025
 H     -1.437674  -3.675282  -3.366025
 C     -0.476139  -2.499559  -1.809397
 H     -0.011098  -1.811621  -2.532570
 C      2.012052  -1.810788   1.248838
 C      2.520506  -1.278111   2.453192
 H      2.116582  -0.334008   2.845926
 C      3.545230  -1.940594   3.154151
 H      3.929467  -1.509857   4.092651
 C      4.073699  -3.147872   2.663546
 H      4.874124  -3.667633   3.213929
 C      3.567039  -3.692819   1.469037
 H      3.968145  -4.642629   1.080253
 C      2.541443  -3.032178   0.770078
 H      2.141982  -3.481978  -0.152471
 C      0.597707   2.286204  -1.011870
 H      1.167617   2.654593  -0.135506
 C     -1.606856   1.172923  -1.408138
 H     -2.020784   1.900182  -2.139926
 H     -1.018946   0.431546  -1.994021
 C     -2.719241   0.442642  -0.697923
 C     -3.356836  -0.760869   1.174949
 C     -4.611709  -1.039699   0.641480
 C     -4.966557  -0.537683  -0.648717
 C     -3.957926   0.229377  -1.307275
 C      0.389511   3.480087  -1.959783
 H      1.363550   3.933194  -2.232019
 H     -0.124028   3.192373  -2.899913
 H     -0.211471   4.262617  -1.453410
 C     -0.774130  -0.328245   2.838555
 C      0.672850   1.557338   2.109225
 H     -1.598980   1.720598   2.045818
 H     -1.719482   3.110656   1.666137
 H     -1.173684   2.656541  -0.055390
 O     -1.864638   3.969512   1.152344
 C     -3.267268   4.248305   1.164042
 H     -3.446000   5.010569   0.374283
```

```
 H     -3.857353   3.345033   0.874902
 H     -4.128535   0.659158  -2.303089
 H     -5.309344  -1.641376   1.238007
 H     -3.094461  -1.136320   2.175232
 N     -6.190632  -0.773341  -1.214619
 C     -7.190215  -1.556822  -0.495147
 H     -7.472894  -1.081386   0.470222
 H     -8.101298  -1.638705  -1.114255
 H     -6.829898  -2.586618  -0.278233
 C     -6.503520  -0.232437  -2.533345
 H     -6.457094   0.879178  -2.546904
 H     -5.807115  -0.614397  -3.312300
 H     -7.527981  -0.533676  -2.815826
 C     -3.748996   4.775087   2.515895
 H     -3.182074   5.683680   2.807843
 H     -4.827991   5.035996   2.477461
 H     -3.611991   4.013726   3.312981
---------------------------------------
Mn8/v
Frequencies, energies and thermodynamic properties:
 Lowest Vibrational Mode (1/cm) =                         20.2581
 2nd Lowest Vibrational Mode (1/cm) =                     22.8006
 E(RB-P86) (a.u.) =                                      -4386.47666671
 Thermal correction to Enthalpy (a.u.) =                  0.638122
 Thermal correction to Gibbs Free Energy (a.u.) =         0.523989
 Total Entropy (cal/Kmol) =                               240.213
 E(RPBE1PBE) (a.u.) =                                    -4385.79016859
Optimised cartesian coordinates (Angstrom):
 Fe-3.224849  -1.302099  -0.777978
 Mn0.632465   0.035074   1.852175
 P     -0.390810   0.769377  -0.039514
 O      1.070799   2.692583   3.026837
 O     -1.707996  -0.343088   3.587718
 N      0.614308  -1.972763   1.074519
 N      2.529526  -0.096111   1.026457
 C     -1.315084  -0.560054  -0.925571
 C     -1.299438  -1.973313  -0.544111
 C     -1.972427  -2.708222  -1.592286
 H     -2.149753  -3.790828  -1.608501
 C     -2.414011  -1.781570  -2.598442
 H     -2.979462  -2.037219  -3.504630
 C     -2.018585  -0.462261  -2.191102
 H     -2.211849   0.465511  -2.744745
 C     -4.119982  -0.743915   0.990620
 H     -3.592043  -0.384793   1.883855
 C     -4.417633  -2.119189   0.686980
 H     -4.166136  -2.987452   1.310975
 C     -5.082478  -2.161114  -0.590550
 H     -5.421258  -3.065999  -1.112731
 C     -5.198923  -0.809441  -1.075394
 H     -5.642090  -0.503898  -2.032749
 C     -4.602451   0.066272  -0.100399
 H     -4.510899   1.157191  -0.179972
 C      0.761466   1.408851  -1.362263
 C      1.455885   2.613235  -1.089670
 H      1.241404   3.168437  -0.161504
 C      2.411160   3.112823  -1.989795
 H      2.932379   4.057212  -1.764271
 C      2.705800   2.408667  -3.173600
 H      3.456677   2.798789  -3.879036
 C      2.036635   1.203170  -3.445799
 H      2.259883   0.642850  -4.368159
 C      1.071631   0.706479  -2.547619
 H      0.547649  -0.232282  -2.785925
 C     -1.619015   2.174482   0.035601
 C     -2.245688   2.490208   1.260477
 H     -1.989832   1.918597   2.164500
 C     -3.197326   3.524238   1.333703
 H     -3.676360   3.756786   2.298341
 C     -3.532550   4.261469   0.183866
 H     -4.275496   5.072952   0.241888
 C     -2.905838   3.962047  -1.040141
 H     -3.155030   4.539309  -1.945110
 C     -1.953735   2.929826  -1.112636
 H     -1.458088   2.720013  -2.073601
 C     -0.709619  -2.587205   0.720424
 H     -1.362770  -2.319067   1.574714
 C      1.660124  -2.134351   0.040012
 H      1.991912  -3.188750  -0.074696
 H      1.216863  -1.829085  -0.933888
 C      2.833375  -1.233229   0.337370
 C      3.551736   0.773369   1.239203
 H      3.300256   1.688458   1.795729
 C      4.860417   0.563294   0.813532
 H      5.611149   1.331049   1.040619
```

```
 C      5.199489  -0.632739   0.109294
 C      4.118826  -1.539084  -0.114783
 H      4.271558  -2.486805  -0.647909
 C     -0.634738  -4.121902   0.653889
 H     -1.652002  -4.555058   0.580712
 H     -0.050527  -4.482125  -0.217283
 H     -0.171589  -4.524928   1.578222
 C      0.880007   1.624840   2.540523
 C     -0.799138  -0.178699   2.837030
 H      0.935312  -2.439039   1.933785
 H      1.351649  -0.635955   3.100932
 N      6.473730  -0.894431  -0.319047
 C      6.767381  -2.136046  -1.026827
 H      6.178392  -2.224987  -1.966391
 H      6.552437  -3.031101  -0.401487
 H      7.838856  -2.156862  -1.294521
 C      7.545038   0.059624  -0.050542
 H      7.696605   0.216537   1.040578
 H      7.342124   1.050213  -0.514360
 H      8.489429  -0.326789  -0.473536
---------------------------------------
Mn8/vi_R
Frequencies, energies and thermodynamic properties:
 Lowest Vibrational Mode (1/cm) =                        16.3705
 2nd Lowest Vibrational Mode (1/cm) =                    18.8245
 E(RB-P86) (a.u.) =                                     -4809.19132127
 Thermal correction to Enthalpy (a.u.) =                 0.790741
 Thermal correction to Gibbs Free Energy (a.u.) =        0.654633
 Total Entropy (cal/Kmol) =                              286.464
 E(RPBE1PBE) (a.u.) =                                   -4808.49986898
Optimised cartesian coordinates (Angstrom):
 Fe3.741321   0.536138  -1.486735
 Mn-0.598177   0.383864   0.524884
 P      1.239513  -0.805169   0.692929
 O     -1.198516   0.394707   3.425841
 O      0.694558   3.002779   0.873518
 N     -0.564373   0.391301  -1.442644
 N     -1.954364  -1.118872   0.153600
 C      2.232707  -0.700621  -0.850692
 C      1.824606   0.025235  -2.051045
 C      2.770210  -0.306938  -3.090380
 H      2.761632   0.086464  -4.114801
 C      3.753418  -1.210828  -2.555608
 H      4.614200  -1.623529  -3.099035
 C      3.433133  -1.450796  -1.175338
 H      3.995261  -2.097808  -0.489831
 C      3.856510   2.249595  -0.346753
 H      3.064298   2.634940   0.307752
 C      4.018771   2.561853  -1.741851
 H      3.377824   3.230187  -2.332527
 C      5.154530   1.825159  -2.235294
 H      5.528284   1.830089  -3.268027
 C      5.696699   1.058950  -1.141844
 H      6.556572   0.377785  -1.196062
 C      4.893365   1.318643   0.024921
 H      5.032274   0.873799   1.018868
 C      0.872334  -2.622741   0.849765
 C      0.378836  -3.096808   2.089017
 H      0.282565  -2.405713   2.942348
 C      0.013814  -4.443731   2.244338
 H     -0.359297  -4.799383   3.218076
 C      0.122149  -5.336092   1.159983
 H     -0.164621  -6.392670   1.281960
 C      0.596869  -4.870747  -0.078115
 H      0.684594  -5.561437  -0.932035
 C      0.970526  -3.521847  -0.234412
 H      1.354740  -3.175134  -1.205927
 C      2.466427  -0.569493   2.072621
 C      2.493533   0.630732   2.814725
 H      1.767985   1.427839   2.601051
 C      3.447834   0.821440   3.831065
 H      3.453557   1.763886   4.401366
 C      4.385475  -0.185238   4.120944
 H      5.130982  -0.035697   4.918030
 C      4.359873  -1.389653   3.393563
 H      5.083926  -2.188671   3.619520
 C      3.404723  -1.583021   2.380765
 H      3.384135  -2.539694   1.835867
 C      0.602711   0.944024  -2.168688
 H      0.846592   1.890139  -1.644918
 C     -1.115132  -0.805052  -2.082819
 H     -1.617087  -0.579450  -3.052975
 H     -0.307098  -1.541349  -2.331634
 C     -2.088966  -1.477361  -1.157625
 C     -2.783142  -1.729564   1.046360
```

```
 H     -2.665385  -1.435868   2.099651
 C     -3.742346  -2.670226   0.696908
 H     -4.362855  -3.102064   1.492333
 C     -3.906123  -3.050080  -0.674536
 C     -3.034770  -2.404414  -1.601388
 H     -3.083136  -2.622409  -2.676335
 C      0.300492   1.316324  -3.633795
 H      1.144480   1.889812  -4.067759
 H      0.134583   0.427332  -4.276304
 H     -0.601886   1.958807  -3.680889
 C     -0.961002   0.392407   2.265391
 C      0.228636   1.923289   0.715229
 H     -1.782369   1.452429  -1.669909
 H     -2.345076   1.735827   0.288752
 N     -4.839035  -3.968610  -1.067148
 C     -4.969892  -4.319995  -2.478333
 H     -4.026922  -4.747276  -2.884558
 H     -5.244030  -3.438744  -3.099310
 H     -5.764705  -5.078335  -2.591805
 C     -5.716584  -4.592864  -0.080250
 H     -6.344932  -3.842020   0.447353
 H     -5.140491  -5.159061   0.683993
 H     -6.390765  -5.302083  -0.592383
 C     -3.072867   2.244537  -0.441844
 C     -3.211183   3.688846   0.021853
 C     -4.490956   1.666658  -0.165099
 C     -4.338946   3.833217   0.860144
 C     -2.395689   4.788278  -0.285914
 C     -5.048311   2.498019   1.016289
 H     -5.094601   1.850771  -1.081133
 C     -4.654436   5.087766   1.408709
 C     -2.714124   6.047086   0.261653
 H     -1.527142   4.663515  -0.952297
 H     -6.154914   2.589356   1.014398
 C     -3.834899   6.194590   1.104611
 H     -5.536257   5.212478   2.059204
 H     -2.088505   6.923534   0.026409
 H     -4.077495   7.185290   1.522295
 O     -2.604268   2.077768  -1.744490
 H     -4.481743   0.573843   0.020949
 H     -4.772454   2.031658   1.990348
---------------------------------------
Mn8/vi_S
Frequencies, energies and thermodynamic properties:
 Lowest Vibrational Mode (1/cm) =                        14.5432
 2nd Lowest Vibrational Mode (1/cm) =                    17.9315
 E(RB-P86) (a.u.) =                                     -4809.19152735
 Thermal correction to Enthalpy (a.u.) =                 0.790789
 Thermal correction to Gibbs Free Energy (a.u.) =        0.653987
 Total Entropy (cal/Kmol) =                              287.924
 E(RPBE1PBE) (a.u.) =                                   -4808.50018919
Optimised cartesian coordinates (Angstrom):
 Fe-3.944582  -0.618217  -1.445189
 Mn0.383545  -0.577802   0.591433
 P     -1.379250   0.727200   0.658132
 O      0.958890  -0.422625   3.493480
 O     -1.061435  -3.084984   1.111000
 N      0.361178  -0.715635  -1.369876
 N      1.844834   0.798105   0.133438
 C     -2.370120   0.571980  -0.882399
 C     -1.998560  -0.256526  -2.026681
 C     -2.918991   0.055982  -3.094234
 H     -2.928377  -0.407100  -4.089132
 C     -3.851426   1.048860  -2.630978
 H     -4.684815   1.471164  -3.208659
 C     -3.524578   1.364607  -1.267575
 H     -4.052560   2.087685  -0.632762
 C     -4.158602  -2.251920  -0.206109
 H     -3.388572  -2.647020   0.468840
 C     -4.347219  -2.634454  -1.580222
 H     -3.751316  -3.373844  -2.132090
 C     -5.438410  -1.859454  -2.113900
 H     -5.817346  -1.901523  -3.143897
 C     -5.926662  -0.998808  -1.066271
 H     -6.743564  -0.270487  -1.158698
 C     -5.134343  -1.238721   0.112131
 H     -5.239673  -0.728282   1.078301
 C     -0.903381   2.526002   0.688943
 C     -0.380459   3.053857   1.893994
 H     -0.323131   2.418045   2.792609
 C      0.063820   4.384287   1.957407
 H      0.459026   4.783195   2.905320
 C      0.007021   5.204820   0.813963
 H      0.356407   6.248334   0.863738
 C     -0.496380   4.684535  -0.390443
```

```
 H     -0.544281   5.318929  -1.289984
 C     -0.950125   3.352757  -0.454639
 H     -1.356092   2.963485  -1.400823
 C     -2.624623   0.663813   2.039864
 C     -2.727156  -0.476302   2.865455
 H     -2.049797  -1.328451   2.715822
 C     -3.696226  -0.536768   3.883862
 H     -3.760929  -1.433761   4.520024
 C     -4.573842   0.541683   4.092085
 H     -5.330966   0.494116   4.890829
 C     -4.473090   1.686862   3.280473
 H     -5.149743   2.541197   3.441659
 C     -3.502802   1.750256   2.265580
 H     -3.422745   2.662268   1.653667
 C     -0.830445  -1.248975  -2.069901
 H     -1.131216  -2.142847  -1.487026
 C      0.985741   0.401307  -2.081915
 H      1.471246   0.085312  -3.034943
 H      0.225714   1.170591  -2.378119
 C      2.000833   1.066173  -1.196698
 C      2.712274   1.407140   0.989822
 H      2.578292   1.184579   2.058498
 C      3.726836   2.264313   0.587001
 H      4.374679   2.702931   1.356458
 C      3.912017   2.550207  -0.804250
 C      3.002159   1.904017  -1.693143
 H      3.062128   2.054339  -2.779006
 C     -0.539018  -1.736583  -3.503035
 H     -1.410079  -2.291796  -3.906128
 H     -0.319410  -0.904489  -4.203039
 H      0.326998  -2.428947  -3.494957
 C      0.733212  -0.487277   2.332623
 C     -0.535749  -2.045415   0.881747
 H      1.528394  -1.857919  -1.523832
 H      2.102325  -2.032135   0.447818
 N      4.898892   3.384690  -1.249169
 C      5.047114   3.644800  -2.678279
 H      4.134172   4.112053  -3.108677
 H      5.257173   2.712443  -3.247333
 H      5.892015   4.338391  -2.835668
 C      5.810325   4.017149  -0.298823
 H      6.388566   3.264957   0.281264
 H      5.268368   4.667248   0.422532
 H      6.529452   4.647426  -0.851465
 C      2.681743  -2.752285  -0.233413
 C      2.398800  -4.196934   0.272009
 C      3.406909  -4.431041   1.423945
 O      2.335626  -2.502139  -1.561964
 C      4.158156  -2.574196   0.093014
 C      4.571979  -3.530314   1.047323
 C      5.061390  -1.633417  -0.424698
 C      5.901661  -3.543657   1.501281
 C      6.395920  -1.649798   0.028111
 H      4.727637  -0.905247  -1.181876
 C      6.811749  -2.597103   0.986296
 H      6.236702  -4.289766   2.241108
 H      7.121816  -0.923532  -0.372491
 H      7.859427  -2.604050   1.328547
 H      1.340567  -4.350314   0.561883
 H      2.623847  -4.880027  -0.576579
 H      2.973866  -4.107103   2.398607
 H      3.701333  -5.494224   1.549954
---------------------------------------
Mn8/viii
Frequencies, energies and thermodynamic properties:
 Lowest Vibrational Mode (1/cm) =                        21.7884
 2nd Lowest Vibrational Mode (1/cm) =                    23.8710
 E(RB-P86) (a.u.) =                                    -4386.44371816
 Thermal correction to Enthalpy (a.u.) =                 0.633353
 Thermal correction to Gibbs Free Energy (a.u.) =        0.518674
 Total Entropy (cal/Kmol) =                              241.364
 E(RPBE1PBE) (a.u.) =                                  -4385.75746288
Optimised cartesian coordinates (Angstrom):
 Fe-3.234467  -1.289358  -0.789272
 Mn0.613751   0.020975   1.890283
 P     -0.373368   0.712723  -0.016104
 O      0.970229   2.804218   2.845440
 O     -1.866839  -0.214724   3.456336
 N      0.579216  -1.904052   1.164146
 N      2.509438  -0.097234   1.060787
 C     -1.296043  -0.611866  -0.885913
 C     -1.322768  -2.007488  -0.460926
 C     -1.982440  -2.752245  -1.507378
 H     -2.185397  -3.830911  -1.494746
 C     -2.371148  -1.846564  -2.556713
```

```
 H     -2.911347  -2.113807  -3.475252
 C     -1.959439  -0.523015  -2.175346
 H     -2.110603   0.390935  -2.764085
 C     -4.195262  -0.630626   0.912806
 H     -3.703864  -0.230457   1.808571
 C     -4.495284  -2.016317   0.670083
 H     -4.279991  -2.851837   1.349745
 C     -5.105118  -2.119585  -0.631646
 H     -5.433355  -3.047422  -1.119287
 C     -5.185167  -0.793779  -1.191712
 H     -5.586058  -0.535250  -2.181120
 C     -4.620020   0.126605  -0.238908
 H     -4.513243   1.211281  -0.370407
 C      0.842385   1.311263  -1.295547
 C      1.506863   2.537608  -1.054427
 H      1.264186   3.126023  -0.154638
 C      2.468580   3.020095  -1.957057
 H      2.970082   3.980777  -1.758275
 C      2.791337   2.279189  -3.110201
 H      3.545329   2.657605  -3.818639
 C      2.146257   1.054048  -3.351036
 H      2.391846   0.466835  -4.250443
 C      1.177705   0.571412  -2.449543
 H      0.673247  -0.384745  -2.656702
 C     -1.559582   2.154165   0.009667
 C     -2.211927   2.533071   1.202701
 H     -2.001705   2.000157   2.140203
 C     -3.139397   3.591435   1.208174
 H     -3.636697   3.873028   2.150011
 C     -3.427285   4.288478   0.021838
 H     -4.152051   5.118091   0.027415
 C     -2.776125   3.925149  -1.171504
 H     -2.987227   4.469811  -2.105678
 C     -1.846547   2.870883  -1.176528
 H     -1.331233   2.613774  -2.114991
 C     -0.696226  -2.539647   0.838112
 H     -1.391284  -2.275988   1.663050
 C      1.608217  -2.158577   0.175893
 H      1.964876  -3.220604   0.175363
 H      1.271842  -1.982522  -0.890278
 C      2.791094  -1.253671   0.393473
 C      3.529909   0.782691   1.237909
 C      4.827636   0.569815   0.788011
 C      5.150189  -0.640334   0.095571
 C      4.070939  -1.555765  -0.082593
 C     -0.614572  -4.083689   0.821683
 H     -1.625447  -4.537919   0.761084
 H     -0.024765  -4.468142  -0.036612
 H     -0.134544  -4.437639   1.756901
 C      0.838603   1.684848   2.484777
 C     -0.904795  -0.116378   2.772331
 H      1.307276  -1.103682   3.054345
 H      1.512872  -0.387224   3.393073
 H      3.286284   1.710026   1.777826
 H      5.581759   1.342775   0.983094
 H      4.217026  -2.514475  -0.597670
 N      6.413925  -0.901606  -0.360880
 C      6.694036  -2.150176  -1.062673
 H      6.078205  -2.252573  -1.983320
 H      7.757466  -2.168969  -1.360772
 H      6.501139  -3.038352  -0.420864
 C      7.487790   0.062258  -0.138956
 H      7.664539   0.243045   0.944382
 H      8.423431  -0.330642  -0.575137
 H      7.270761   1.041638  -0.619727
---------------------------------------
Mn8/ix
Frequencies, energies and thermodynamic properties:
 Lowest Vibrational Mode (1/cm) =                         20.4054
 2nd Lowest Vibrational Mode (1/cm) =                     25.2657
 E(RB-P86) (a.u.) =                                      -4540.23278122
 Thermal correction to Enthalpy (a.u.) =                  0.702544
 Thermal correction to Gibbs Free Energy (a.u.) =         0.578677
 Total Entropy (cal/Kmol) =                               260.699
 E(RPBE1PBE) (a.u.) =                                    -4539.55022034
Optimised cartesian coordinates (Angstrom):
 Fe-3.366121  -0.566847  -1.446890
 Mn0.640739  -0.830331   1.294747
 P     -0.554249   0.810105   0.309652
 O      1.039959   0.688436   3.784403
 O     -1.691796  -2.164502   2.490000
 N      0.606834  -1.935492  -0.525438
 N      2.433009  -0.204590   0.420868
 C     -1.485278   0.213412  -1.169895
 C     -1.410697  -1.138344  -1.725943
```

```
 C     -2.123919  -1.125871  -2.982534
 H     -2.271905  -1.987055  -3.646175
 C     -2.645887   0.194428  -3.207114
 H     -3.254888   0.509239  -4.065194
 C     -2.263370   1.018328  -2.094478
 H     -2.512670   2.079541  -1.969000
 C     -4.193662  -1.220571   0.323806
 H     -3.637235  -1.434789   1.245409
 C     -4.460196  -2.159435  -0.733921
 H     -4.151298  -3.213291  -0.754852
 C     -5.182650  -1.469658  -1.772183
 H     -5.515837  -1.903265  -2.724654
 C     -5.365326  -0.103017  -1.353770
 H     -5.862276   0.687225  -1.932415
 C     -4.751948   0.052260  -0.060221
 H     -4.698528   0.980360   0.523561
 C      0.477290   2.198366  -0.393264
 C      1.085389   3.092060   0.521100
 H      0.884298   2.991833   1.600425
 C      1.939733   4.110692   0.068624
 H      2.395872   4.802955   0.794485
 C      2.215214   4.245432  -1.306004
 H      2.886397   5.043438  -1.661341
 C      1.628881   3.353869  -2.220419
 H      1.837667   3.449949  -3.298124
 C      0.764931   2.337350  -1.768191
 H      0.302561   1.658020  -2.500946
 C     -1.820294   1.798385   1.261966
 C     -2.347819   1.304673   2.474637
 H     -1.997357   0.343950   2.875963
 C     -3.326809   2.029335   3.179698
 H     -3.725194   1.627123   4.124831
 C     -3.790953   3.260700   2.685124
 H     -4.555994   3.828442   3.238306
 C     -3.263836   3.768269   1.483008
 H     -3.612750   4.737015   1.090826
 C     -2.283457   3.046167   0.780744
 H     -1.866146   3.469534  -0.146127
 C     -0.697418  -2.341924  -1.117087
 H     -1.291674  -2.699372  -0.252021
 C      1.602008  -1.447464  -1.496796
 H      1.995512  -2.257554  -2.148877
 H      1.100721  -0.716731  -2.171304
 C      2.737347  -0.746928  -0.789311
 C      3.417682   0.484755   1.049293
 C      4.696132   0.666798   0.531570
 C      5.037972   0.098724  -0.735283
 C      3.996652  -0.630758  -1.384140
 C     -0.566531  -3.516751  -2.101984
 H     -1.568202  -3.903322  -2.377270
 H     -0.046243  -3.230133  -3.038576
 H     -0.005842  -4.349282  -1.629921
 C      0.881447   0.067410   2.786776
 C     -0.775961  -1.602551   1.984848
 H      1.028155  -2.703952   0.057104
 O      1.789426  -2.462191   1.660822
 C      1.732957  -3.176452   2.855991
 H      0.703908  -3.577133   3.080487
 H      1.988502  -2.530666   3.742368
 H      4.159428  -1.109460  -2.358743
 H      5.422057   1.242058   1.120240
 C      2.709354  -4.361582   2.828351
 H      2.682212  -4.931877   3.782327
 H      2.456311  -5.060412   2.001753
 H      3.750514  -4.009411   2.664407
 H      3.163038   0.910234   2.031506
 N      6.282351   0.240522  -1.286809
 C      6.586298  -0.375000  -2.574779
 C      7.317842   0.984948  -0.576122
 H      5.923977   0.009229  -3.381626
 H      7.629885  -0.141854  -2.851457
 H      6.479192  -1.482007  -2.540836
 H      8.236728   1.002555  -1.188827
 H      7.012151   2.037517  -0.387794
 H      7.566562   0.518751   0.403052
---------------------------------------
Mn8/x
Frequencies, energies and thermodynamic properties:
 Lowest Vibrational Mode (1/cm) =                          18.0245
 2nd Lowest Vibrational Mode (1/cm) =                      20.8570
 E(RB-P86) (a.u.) =                                      -4695.17372696
 Thermal correction to Enthalpy (a.u.) =                   0.786636
 Thermal correction to Gibbs Free Energy (a.u.) =          0.649715
 Total Entropy (cal/Kmol) =                                288.174
 E(RPBE1PBE) (a.u.) =                                    -4694.49941110
```

Optimised cartesian coordinates (Angstrom):

Fe	-3.539822	-0.779894	-1.427011
Mn	0.584437	-0.523054	1.186936
P	-0.898805	0.927421	0.289126
O	0.690162	1.107100	3.632603
O	-1.413347	-2.308644	2.397042
N	0.646536	-1.583753	-0.690591
N	2.222447	0.378752	0.269378
C	-1.793702	0.270267	-1.179606
C	-1.533739	-1.030794	-1.792915
C	-2.286858	-1.082438	-3.025086
H	-2.325713	-1.931090	-3.719398
C	-3.013590	0.148728	-3.176007
H	-3.696341	0.396688	-3.999765
C	-2.720961	0.980577	-2.041961
H	-3.124415	1.985209	-1.862630
C	-4.180364	-1.631353	0.336346
H	-3.558777	-1.798660	1.225383
C	-4.348199	-2.551911	-0.756991
H	-3.884772	-3.543980	-0.842900
C	-5.211772	-1.935114	-1.731350
H	-5.517084	-2.371651	-2.691770
C	-5.580829	-0.632936	-1.237408
H	-6.216841	0.096834	-1.756193
C	-4.941636	-0.443243	0.038758
H	-5.004901	0.454174	0.667650
C	-0.113226	2.487159	-0.368888
C	0.403955	3.411727	0.570533
H	0.274248	3.227961	1.649517
C	1.076872	4.567900	0.143061
H	1.464149	5.280659	0.888743
C	1.258388	4.814581	-1.231501
H	1.787234	5.720818	-1.567014
C	0.760259	3.896618	-2.171684
H	0.895521	4.080180	-3.249708
C	0.078030	2.740805	-1.744298
H	-0.319839	2.041959	-2.496202
C	-2.257427	1.660330	1.339899
C	-2.679198	1.017169	2.523599
H	-2.190121	0.089963	2.852066
C	-3.730356	1.548354	3.293733
H	-4.043519	1.031819	4.215018
C	-4.374625	2.732154	2.893944
H	-5.196130	3.148470	3.498406
C	-3.956481	3.386818	1.720647
H	-4.448085	4.319889	1.401854
C	-2.903689	2.858548	0.953462
H	-2.575704	3.395914	0.050104
C	-0.634778	-2.131855	-1.249064
H	-1.137300	-2.589758	-0.373644
C	1.459372	-0.844135	-1.681031
H	1.907386	-1.512897	-2.447475
H	0.793472	-0.139520	-2.227734
C	2.532114	-0.042281	-0.987446
C	3.132405	1.167199	0.893844
C	4.342378	1.559593	0.330368
C	4.693499	1.115173	-0.982534
C	3.729608	0.283346	-1.629420
C	-0.381642	-3.256044	-2.268763
H	-1.330410	-3.770684	-2.520850
H	0.060847	-2.880516	-3.214143
H	0.305850	-4.004512	-1.825859
C	0.656532	0.439111	2.654233
C	-0.648927	-1.558231	1.889466
H	2.113545	-3.077704	0.952134
H	1.204296	-2.429934	-0.409489
O	2.117333	-3.852109	0.224217
C	3.444462	-4.024032	-0.249219
H	3.396726	-4.402593	-1.297766
H	3.987491	-3.046862	-0.294045
H	3.903627	-0.110142	-2.639463
H	5.008768	2.203094	0.918767
H	2.870919	1.502262	1.908388
C	4.247985	-5.012587	0.601442
H	3.733648	-5.995952	0.645650
H	5.264469	-5.172216	0.181318
H	4.362946	-4.642178	1.642450
O	2.030794	-1.891325	1.761857
C	2.232196	-2.261140	3.105380
C	3.336460	-1.455149	3.803271
H	2.516061	-3.346471	3.144314
H	1.292403	-2.179877	3.709675
H	3.510632	-1.832135	4.835241
H	4.292231	-1.531923	3.242124
H	3.066080	-0.381485	3.874604

```
 N      5.875039   1.460759  -1.578750
 C      6.834437   2.302643  -0.869633
 C      6.190159   0.971633  -2.917532
 H      7.714063   2.472954  -1.515558
 H      6.401273   3.294147  -0.612389
 H      7.186664   1.826122   0.071858
 H      7.174433   1.366476  -3.226081
 H      6.239537  -0.139313  -2.951582
 H      5.437592   1.305130  -3.665503
---------------------------------------
Mn8/TS-i
Frequencies, energies and thermodynamic properties:
 Lowest Vibrational Mode (1/cm) =-734.7335
 2nd Lowest Vibrational Mode (1/cm) =                      17.4018
 E(RB-P86) (a.u.) =                                       -4541.38635464
 Thermal correction to Enthalpy (a.u.) =                   0.714216
 Thermal correction to Gibbs Free Energy (a.u.) =          0.586973
 Total Entropy (cal/Kmol) =                                267.805
 E(RPBE1PBE) (a.u.) =                                     -4540.70299857
Optimised cartesian coordinates (Angstrom):
 Fe3.345539   0.879107  -1.318381
 Mn-0.595565   0.484762   1.518856
 P      0.662086  -0.904498   0.239512
 O     -0.723142  -1.506199   3.697612
 O      1.661010   1.816485   2.860827
 N     -0.696428   1.841787  -0.088087
 N     -2.388902  -0.083645   0.651102
 C      1.523053  -0.047763  -1.134013
 C      1.347371   1.363261  -1.467187
 C      2.024613   1.588559  -2.722798
 H      2.101018   2.550063  -3.246069
 C      2.622130   0.355389  -3.160252
 H      3.225165   0.216728  -4.067744
 C      2.325396  -0.654365  -2.182022
 H      2.642107  -1.704230  -2.226203
 C      4.186968   1.311930   0.513717
 H      3.651252   1.347172   1.470821
 C      4.358988   2.415985  -0.392477
 H      3.983089   3.437312  -0.244439
 C      5.089052   1.941358  -1.540417
 H      5.362969   2.535754  -2.422396
 C      5.371350   0.542374  -1.340867
 H      5.899079  -0.115430  -2.044491
 C      4.811303   0.151825  -0.073092
 H      4.838231  -0.854577   0.364405
 C     -0.340399  -2.215934  -0.624046
 C     -0.913233  -3.238530   0.169813
 H     -0.716531  -3.268451   1.253876
 C     -1.724902  -4.225910  -0.411994
 H     -2.155561  -5.019448   0.219374
 C     -1.988594  -4.201229  -1.794979
 H     -2.625496  -4.975547  -2.251306
 C     -1.434668  -3.182608  -2.588868
 H     -1.634259  -3.154563  -3.672048
 C     -0.615185  -2.195232  -2.008302
 H     -0.177776  -1.412745  -2.647092
 C      1.988495  -1.955222   1.024464
 C      2.556723  -1.595489   2.265569
 H      2.199022  -0.704897   2.800256
 C      3.588338  -2.366982   2.831759
 H      4.017637  -2.071110   3.802157
 C      4.065702  -3.510060   2.167130
 H      4.871836  -4.114614   2.612267
 C      3.500440  -3.882393   0.933542
 H      3.860721  -4.781031   0.407794
 C      2.467003  -3.114740   0.369354
 H      2.021661  -3.432627  -0.586168
 C      0.564424   2.389752  -0.650649
 H      1.179967   2.656456   0.232820
 C     -1.622314   1.369150  -1.125703
 H     -2.061640   2.203354  -1.720388
 H     -1.094482   0.719048  -1.868716
 C     -2.723293   0.545744  -0.512285
 C     -3.328215  -0.892974   1.207949
 C     -4.591409  -1.105055   0.668180
 C     -4.968505  -0.443756  -0.543284
 C     -3.974101   0.403793  -1.118211
 C      0.330142   3.697774  -1.434155
 H      1.294208   4.189663  -1.676011
 H     -0.211003   3.530755  -2.388388
 H     -0.262194   4.394108  -0.807159
 C     -0.680154  -0.704009   2.831215
 C      0.792734   1.258783   2.281943
 H     -1.680582   1.310945   2.555898
 H     -1.508710   2.020803   2.113276
```

```
 H     -1.259261   2.810513   0.588310
 O     -1.691680   3.608114   1.393667
 C     -3.067422   3.852646   1.249417
 H     -3.353744   4.003846   0.173375
 H     -3.687825   2.979236   1.597389
 H     -4.165231   0.957250  -2.046851
 H     -5.278892  -1.777028   1.197300
 H     -3.046565  -1.393374   2.146416
 N     -6.201804  -0.610243  -1.109646
 C     -7.187420  -1.482534  -0.476779
 H     -7.457745  -1.130265   0.543170
 H     -8.106910  -1.494008  -1.088518
 H     -6.818361  -2.528250  -0.393752
 C     -6.544530   0.098708  -2.339089
 H     -6.500849   1.202183  -2.205381
 H     -5.863431  -0.173658  -3.175044
 H     -7.573833  -0.168330  -2.637561
 C     -3.493533   5.091163   2.044523
 H     -2.933029   5.986396   1.699715
 H     -4.580199   5.295766   1.931854
 H     -3.277690   4.955517   3.125998
---------------------------------------
Mn8/TS-ii_si
Frequencies, energies and thermodynamic properties:
 Lowest Vibrational Mode (1/cm) =-206.8904
 2nd Lowest Vibrational Mode (1/cm) =                     14.2275
 E(RB-P86) (a.u.) =                                     -4809.18468291
 Thermal correction to Enthalpy (a.u.) =                  0.787453
 Thermal correction to Gibbs Free Energy (a.u.) =         0.653368
 Total Entropy (cal/Kmol) =                               282.205
 E(RPBE1PBE) (a.u.) =                                   -4808.48770068
Optimised cartesian coordinates (Angstrom):
 Fe3.739210  -0.019851  -1.459587
 Mn-0.645597   0.608405   0.498492
 P      1.017005  -0.907419   0.703003
 O     -1.268304   0.838771   3.364293
 O      0.989441   3.042394   0.691337
 N     -0.552991   0.472788  -1.605605
 N     -2.137277  -0.773655   0.136812
 C      2.061996  -1.010839  -0.811687
 C      1.791248  -0.290398  -2.056163
 C      2.706105  -0.799312  -3.052914
 H      2.778840  -0.457928  -4.093086
 C      3.540135  -1.802605  -2.449313
 H      4.350053  -2.351807  -2.948025
 C      3.153643  -1.931653  -1.071941
 H      3.604389  -2.614632  -0.340586
 C      4.052595   1.728245  -0.414033
 H      3.296355   2.259125   0.178919
 C      4.311821   1.926280  -1.815772
 H      3.796057   2.639031  -2.473384
 C      5.350404   1.009711  -2.212035
 H      5.760431   0.896965  -3.224648
 C      5.735784   0.246449  -1.052364
 H      6.491562  -0.550047  -1.026923
 C      4.932351   0.687750   0.058158
 H      4.966707   0.290000   1.080644
 C      0.436820  -2.666733   0.923605
 C     -0.180939  -3.001198   2.153465
 H     -0.236966  -2.250855   2.959408
 C     -0.718346  -4.281707   2.362569
 H     -1.185412  -4.526320   3.330151
 C     -0.664132  -5.248342   1.339615
 H     -1.086922  -6.252472   1.502566
 C     -0.068817  -4.921679   0.109242
 H     -0.021571  -5.669900  -0.698385
 C      0.478454  -3.640408  -0.098266
 H      0.955015  -3.408122  -1.063285
 C      2.256830  -0.787115   2.092468
 C      2.448993   0.440158   2.762735
 H      1.851299   1.317452   2.477248
 C      3.404153   0.554388   3.789803
 H      3.539924   1.520362   4.301968
 C      4.178010  -0.558270   4.164648
 H      4.923748  -0.469398   4.970678
 C      3.987590  -1.789113   3.509405
 H      4.582770  -2.669321   3.801127
 C      3.031730  -1.903891   2.484836
 H      2.880912  -2.879186   1.996015
 C      0.745003   0.795211  -2.285659
 H      1.088266   1.721743  -1.783776
 C     -1.183350  -0.775211  -2.089596
 H     -1.570255  -0.679446  -3.127037
 H     -0.409881  -1.575606  -2.108894
 C     -2.284023  -1.197821  -1.151890
```

```
 C     -3.066866  -1.210537   1.028784
 H     -2.941095  -0.868169   2.066564
 C     -4.137989  -2.035997   0.701372
 H     -4.831520  -2.329860   1.499564
 C     -4.317768  -2.471826  -0.648387
 C     -3.336144  -2.011767  -1.577199
 H     -3.380423  -2.291728  -2.637862
 C      0.550733   1.127230  -3.775477
 H      1.483158   1.547689  -4.202206
 H      0.274437   0.237866  -4.377875
 H     -0.244352   1.891635  -3.889546
 C     -0.998298   0.733266   2.213141
 C      0.376762   2.028130   0.605454
 H     -1.225349   1.250879  -1.848618
 H     -1.816924   1.731289   0.223685
 N     -5.360766  -3.273713  -1.025189
 C     -5.500361  -3.686203  -2.418259
 H     -4.620154  -4.270850  -2.766207
 H     -5.623251  -2.813779  -3.097631
 H     -6.395153  -4.325740  -2.519749
 C     -6.341254  -3.711863  -0.036343
 H     -6.869563  -2.851915   0.431652
 H     -5.870735  -4.309255   0.775465
 H     -7.097051  -4.346873  -0.531832
 C     -2.859865   2.638755  -0.847521
 C     -2.508550   3.950693  -0.202679
 C     -4.142198   2.161347  -0.126371
 C     -3.292182   4.140025   0.957796
 C     -1.612909   4.934907  -0.652256
 C     -4.164019   2.923548   1.216752
 H     -4.991083   2.486092  -0.771936
 C     -3.176984   5.332433   1.691083
 C     -1.501793   6.128716   0.082292
 H     -1.021475   4.767059  -1.566643
 H     -5.184271   3.192021   1.560991
 C     -2.278057   6.323497   1.245868
 H     -3.783120   5.499181   2.596892
 H     -0.811533   6.920414  -0.251003
 H     -2.184323   7.265879   1.809567
 O     -2.530214   2.299348  -2.015803
 H     -4.199191   1.060231  -0.036683
 H     -3.704985   2.306041   2.022110
---------------------------------------
Mn8/TS-ii_re
Frequencies, energies and thermodynamic properties:
 Lowest Vibrational Mode (1/cm) =-220.2283
 2nd Lowest Vibrational Mode (1/cm) =                     13.3412
 E(RB-P86) (a.u.) =                                      -4809.18492612
 Thermal correction to Enthalpy (a.u.) =                  0.787478
 Thermal correction to Gibbs Free Energy (a.u.) =         0.653352
 Total Entropy (cal/Kmol) =                               282.292
 E(RPBE1PBE) (a.u.) =                                    -4808.48902094
Optimised cartesian coordinates (Angstrom):
 Fe-3.978862  -0.276425  -1.370705
 Mn0.448474  -0.815889   0.517726
 P     -1.209380   0.711714   0.679553
 O      1.092065  -0.902498   3.387366
 O     -1.183822  -3.233817   0.859553
 N      0.309323  -0.787776  -1.586611
 N      1.931836   0.544817   0.052770
 C     -2.284676   0.743341  -0.816744
 C     -2.044233  -0.044750  -2.025912
 C     -2.981012   0.412092  -3.027459
 H     -3.078163   0.015197  -4.045708
 C     -3.798793   1.449482  -2.460349
 H     -4.618700   1.973689  -2.969510
 C     -3.380085   1.652382  -1.101401
 H     -3.811927   2.376241  -0.398494
 C     -4.269645  -1.962764  -0.223534
 H     -3.500061  -2.461514   0.379890
 C     -4.561453  -2.237509  -1.605843
 H     -4.061488  -2.986364  -2.234903
 C     -5.608253  -1.342078  -2.027993
 H     -6.041726  -1.284737  -3.035489
 C     -5.965933  -0.515024  -0.903729
 H     -6.720141   0.283365  -0.904857
 C     -5.137470  -0.896070   0.210647
 H     -5.148102  -0.442412   1.210138
 C     -0.624758   2.478374   0.806848
 C      0.011632   2.870438   2.009834
 H      0.081523   2.158514   2.848794
 C      0.550155   4.159794   2.150284
 H      1.031923   4.449864   3.097872
 C      0.478467   5.077187   1.084039
 H      0.901849   6.088329   1.193244
```

```
 C     -0.134925   4.692565  -0.120462
 H     -0.195490   5.401989  -0.961505
 C     -0.683290   3.402508  -0.259185
 H     -1.174011   3.124548  -1.204833
 C     -2.419495   0.658509   2.099425
 C     -2.595477  -0.533711   2.834235
 H     -2.001164  -1.423292   2.581918
 C     -3.528869  -0.597273   3.885414
 H     -3.651900  -1.536452   4.448127
 C     -4.297223   0.531873   4.219889
 H     -5.026061   0.482706   5.044556
 C     -4.122558   1.728591   3.500224
 H     -4.713254   2.621763   3.759836
 C     -3.188183   1.792893   2.451724
 H     -3.049385   2.742819   1.912074
 C     -1.004457  -1.143400  -2.219245
 H     -1.337447  -2.040786  -1.660825
 C      0.927678   0.432287  -2.150578
 H      1.303973   0.276239  -3.184783
 H      0.148298   1.224842  -2.211756
 C      2.035532   0.922021  -1.253641
 C      2.870874   1.037018   0.904087
 H      2.785395   0.721639   1.954344
 C      3.900921   1.890386   0.522849
 H      4.604621   2.233100   1.292019
 C      4.030643   2.286932  -0.844150
 C      3.049351   1.754715  -1.733645
 H      3.059871   1.997617  -2.804342
 C     -0.842894  -1.557160  -3.692356
 H     -1.786067  -1.994606  -4.076237
 H     -0.574604  -0.703763  -4.348110
 H     -0.054198  -2.331689  -3.778909
 C      0.822673  -0.853298   2.232900
 C     -0.574869  -2.221307   0.717203
 H      0.969991  -1.584983  -1.802922
 H      1.636292  -1.933463   0.271476
 N      5.030381   3.118289  -1.272545
 C      5.122865   3.485409  -2.681852
 H      4.212996   4.022885  -3.029881
 H      5.264432   2.594361  -3.332870
 H      5.989940   4.154120  -2.827115
 C      6.008217   3.637141  -0.321084
 H      6.588977   2.820933   0.162889
 H      5.525301   4.237157   0.481486
 H      6.720328   4.292277  -0.853756
 C      2.538018  -3.003860  -0.764937
 C      3.842036  -2.645693  -0.108317
 C      2.055664  -4.280959  -0.036640
 C      4.015530  -3.413581   1.065671
 C      4.835249  -1.761419  -0.560915
 C      2.795399  -4.281664   1.318913
 C      5.200835  -3.293276   1.809345
 C      6.022243  -1.645879   0.184450
 H      4.678671  -1.184496  -1.486557
 C      6.201125  -2.405779   1.361258
 H      5.355852  -3.888028   2.724736
 H      6.821814  -0.966337  -0.151933
 H      7.138601  -2.309398   1.932665
 O      2.219506  -2.689931  -1.944167
 H      3.058504  -5.296042   1.683898
 H      2.162981  -3.810841   2.105705
 H      0.954722  -4.349304   0.036189
 H      2.404167  -5.131370  -0.667635
---------------------------------------
Mn8/TS-iii
Frequencies, energies and thermodynamic properties:
 Lowest Vibrational Mode (1/cm) =-605.8970
 2nd Lowest Vibrational Mode (1/cm) =                     21.8128
 E(RB-P86) (a.u.) =                                    -4386.44093988
 Thermal correction to Enthalpy (a.u.) =                 0.632356
 Thermal correction to Gibbs Free Energy (a.u.) =        0.518932
 Total Entropy (cal/Kmol) =                              238.722
 E(RPBE1PBE) (a.u.) =                                  -4385.75149918
Optimised cartesian coordinates (Angstrom):
 Fe-3.225653  -1.274064  -0.796836
 Mn0.597538  -0.025901   1.880650
 P     -0.373180   0.744566  -0.017182
 O      1.059104   2.633008   3.088013
 O     -1.886016  -0.250171   3.440258
 N      0.598285  -1.975793   1.119532
 N      2.503229  -0.122971   1.054107
 C     -1.288948  -0.590199  -0.887843
 C     -1.316183  -1.995795  -0.481034
 C     -1.975877  -2.724559  -1.539826
 H     -2.178554  -3.803157  -1.543408
```

```
 C     -2.366096  -1.805215  -2.575125
 H     -2.908607  -2.060212  -3.495635
 C     -1.953692  -0.488225  -2.175670
 H     -2.107277   0.433985  -2.750718
 C     -4.191199  -0.635405   0.910264
 H     -3.701941  -0.243726   1.810808
 C     -4.487381  -2.019362   0.652083
 H     -4.271515  -2.861657   1.323137
 C     -5.095078  -2.110040  -0.651383
 H     -5.420004  -3.033328  -1.149737
 C     -5.177189  -0.778570  -1.197321
 H     -5.576351  -0.510474  -2.184856
 C     -4.616003   0.132851  -0.233776
 H     -4.511304   1.219062  -0.353714
 C      0.830606   1.359325  -1.300455
 C      1.503122   2.577003  -1.037538
 H      1.266786   3.148290  -0.124743
 C      2.465070   3.072262  -1.933006
 H      2.972391   4.026112  -1.716677
 C      2.781544   2.352095  -3.100998
 H      3.536171   2.739916  -3.803639
 C      2.130035   1.134649  -3.363085
 H      2.371277   0.562953  -4.273646
 C      1.161198   0.639798  -2.468586
 H      0.653141  -0.310852  -2.692453
 C     -1.569150   2.175909   0.024298
 C     -2.203293   2.543513   1.230687
 H     -1.976568   2.001216   2.159168
 C     -3.132115   3.600373   1.259634
 H     -3.615705   3.873226   2.211157
 C     -3.438136   4.307566   0.083789
 H     -4.163703   5.136177   0.107493
 C     -2.803922   3.956327  -1.122395
 H     -3.029323   4.509766  -2.048058
 C     -1.873492   2.903164  -1.150971
 H     -1.370838   2.655432  -2.098882
 C     -0.694105  -2.582122   0.796604
 H     -1.368551  -2.328455   1.641096
 C      1.624959  -2.175667   0.113880
 H      1.989102  -3.232116   0.062937
 H      1.259335  -1.947780  -0.928084
 C      2.798359  -1.262798   0.364683
 C      3.518509   0.756987   1.257063
 C      4.819771   0.562468   0.807768
 C      5.154058  -0.629183   0.090540
 C      4.081480  -1.548704  -0.110253
 C     -0.630180  -4.124543   0.734804
 H     -1.645906  -4.565291   0.663053
 H     -0.045252  -4.488685  -0.135400
 H     -0.153794  -4.511607   1.658438
 C      0.872194   1.568539   2.607649
 C     -0.923444  -0.159859   2.754690
 H      1.097801  -1.485189   2.555354
 H      1.339511  -0.852473   3.180405
 H      3.268923   1.668925   1.819531
 H      5.567743   1.335696   1.024289
 H      4.236315  -2.496125  -0.643178
 N      6.420404  -0.872181  -0.367248
 C      6.712657  -2.105765  -1.090627
 H      6.101217  -2.195751  -2.015435
 H      7.777351  -2.110907  -1.384612
 H      6.524200  -3.006763  -0.465720
 C      7.486187   0.095638  -0.123795
 H      7.662440   0.252732   0.963305
 H      8.424583  -0.278718  -0.570176
 H      7.259405   1.083665  -0.581588
---------------------------------------
Mn9/i
Frequencies, energies and thermodynamic properties:
 Lowest Vibrational Mode (1/cm) =                        13.8385
 2nd Lowest Vibrational Mode (1/cm) =                    24.6535
 E(RB-P86) (a.u.) =                                     -4385.26260425
 Thermal correction to Enthalpy (a.u.) =                 0.616418
 Thermal correction to Gibbs Free Energy (a.u.) =        0.503822
 Total Entropy (cal/Kmol) =                              236.980
 E(RPBE1PBE) (a.u.) =                                   -4384.58040491
Optimised cartesian coordinates (Angstrom):
 Fe3.437364  -0.806867   0.248761
 Mn-1.068071  -0.316046  -1.289731
 P      0.136370   0.596981   0.282491
 O     -2.119549   2.319875  -2.164068
 O      0.869448  -0.007146  -3.460630
 N     -0.530827  -2.064560  -0.720910
 N     -2.906636  -1.035209  -0.458788
 C      1.497370  -0.498831   0.851510
```

```
 C      1.674111  -1.871880   0.395048
 C      2.703172  -2.467053   1.213178
 H      3.090788  -3.489407   1.115806
 C      3.167447  -1.486007   2.159692
 H      3.962062  -1.632808   2.903923
 C      2.435267  -0.268415   1.935564
 H      2.558863   0.668843   2.493687
 C      3.794220  -0.061709  -1.639562
 H      3.024532   0.172776  -2.386484
 C      4.439279  -1.336715  -1.471281
 H      4.254432  -2.237899  -2.071420
 C      5.351680  -1.233310  -0.360576
 H      5.980736  -2.041602   0.036004
 C      5.272685   0.109614   0.155990
 H      5.831668   0.503738   1.015387
 C      4.308484   0.833159  -0.631838
 H      4.002824   1.876890  -0.482985
 C     -0.894569   0.823251   1.818817
 C     -1.939103   1.779040   1.775396
 H     -2.080925   2.394175   0.871872
 C     -2.793572   1.954256   2.875277
 H     -3.593681   2.710352   2.830064
 C     -2.632154   1.163913   4.030570
 H     -3.305447   1.299090   4.891936
 C     -1.608787   0.202500   4.077117
 H     -1.475463  -0.420381   4.976179
 C     -0.742609   0.032196   2.979213
 H      0.064374  -0.713857   3.035799
 C      0.924185   2.273473   0.112264
 C      1.248647   2.780286  -1.165194
 H      1.009711   2.197303  -2.066095
 C      1.881259   4.029638  -1.297907
 H      2.125873   4.411606  -2.301868
 C      2.195570   4.790231  -0.157308
 H      2.688274   5.769853  -0.262959
 C      1.869686   4.296656   1.119184
 H      2.105271   4.888198   2.018316
 C      1.234866   3.049223   1.253637
 H      0.969851   2.683606   2.258152
 C      0.870741  -2.515790  -0.744043
 H      1.291166  -2.133979  -1.695905
 C     -1.310809  -2.666993   0.338782
 H     -1.468055  -3.766935   0.196933
 H     -0.799940  -2.586988   1.335714
 C     -2.664207  -2.025322   0.444048
 C     -4.178465  -0.507663  -0.533449
 C     -5.143421  -0.826627   0.457524
 H     -6.145640  -0.381540   0.412814
 C     -4.841322  -1.774210   1.441692
 C     -3.599281  -2.424176   1.412353
 H     -3.341931  -3.215706   2.131274
 C      1.036782  -4.047689  -0.772897
 H      2.090899  -4.316590  -0.988121
 H      0.759483  -4.527543   0.188177
 H      0.406571  -4.481754  -1.575965
 C     -1.756708   1.245361  -1.820102
 C      0.133043  -0.114385  -2.531452
 H     -5.595868  -2.038221   2.199320
 N     -4.488493   0.321255  -1.601014
 C     -4.168617  -0.113895  -2.962932
 H     -5.018758  -0.687463  -3.404471
 H     -3.263791  -0.747230  -2.960909
 H     -3.964659   0.767348  -3.603549
 C     -5.672250   1.165149  -1.521723
 H     -5.590249   1.960499  -2.289707
 H     -5.737263   1.654411  -0.530626
 H     -6.624177   0.610284  -1.709152
---------------------------------------
Mn9/ii
Frequencies, energies and thermodynamic properties:
 Lowest Vibrational Mode (1/cm) =                        21.2411
 2nd Lowest Vibrational Mode (1/cm) =                    22.5613
 E(RB-P86) (a.u.) =                                    -4540.20006328
 Thermal correction to Enthalpy (a.u.) =                 0.701182
 Thermal correction to Gibbs Free Energy (a.u.) =        0.574539
 Total Entropy (cal/Kmol) =                              266.543
 E(RPBE1PBE) (a.u.) =                                  -4539.52636272
Optimised cartesian coordinates (Angstrom):
 Fe-3.457553   1.124679   0.556150
 Mn1.034886   0.253070  -0.866676
 P     -0.575626  -0.932374   0.011252
 O      1.656027  -1.898151  -2.809647
 O     -0.561695   1.485986  -2.983901
 N      0.800259   1.646503   0.481513
 N      2.843088   0.072207   0.240975
```

C	-1.746678	0.078433	0.995810
C	-1.557921	1.497892	1.272703
C	-2.556150	1.879891	2.242403
H	-2.701575	2.888303	2.650669
C	-3.358872	0.728072	2.561426
H	-4.213170	0.708089	3.251673
C	-2.871630	-0.383219	1.789791
H	-3.275220	-1.404045	1.805001
C	-3.670142	1.457551	-1.468059
H	-2.867483	1.391754	-2.213983
C	-4.028469	2.635288	-0.723753
H	-3.552080	3.621413	-0.807570
C	-5.104395	2.288986	0.170119
H	-5.589289	2.963237	0.888812
C	-5.413521	0.895129	-0.025188
H	-6.175896	0.321465	0.518887
C	-4.525366	0.379841	-1.034899
H	-4.491895	-0.654514	-1.400840
C	0.150741	-2.100798	1.267847
C	0.995082	-3.134680	0.793568
H	1.160611	-3.255103	-0.289203
C	1.619057	-4.015250	1.691288
H	2.264217	-4.820846	1.305971
C	1.424135	-3.867727	3.078685
H	1.916746	-4.556797	3.782951
C	0.598837	-2.837215	3.559085
H	0.440406	-2.714887	4.642518
C	-0.036711	-1.957969	2.660757
H	-0.693597	-1.165900	3.050794
C	-1.652130	-2.073313	-0.985464
C	-1.879123	-1.824994	-2.356825
H	-1.395681	-0.969939	-2.850329
C	-2.725853	-2.664461	-3.102996
H	-2.891113	-2.458289	-4.172426
C	-3.354012	-3.763580	-2.490299
H	-4.014492	-4.421873	-3.076733
C	-3.127452	-4.023147	-1.126323
H	-3.608926	-4.886134	-0.639318
C	-2.279133	-3.187064	-0.379244
H	-2.096894	-3.413007	0.683133
C	-0.474967	2.375820	0.635567
H	-0.805075	2.604722	-0.398116
C	1.461421	1.363262	1.747492
H	1.812475	2.284420	2.277067
H	0.770216	0.865546	2.477144
C	2.651628	0.469179	1.529707
C	3.997419	-0.602331	-0.089461
C	4.847605	-1.096936	0.937692
C	4.579422	-0.767873	2.269355
C	3.495025	0.070912	2.576737
C	-0.323544	3.730380	1.354811
H	-1.259201	4.320157	1.274409
H	-0.088222	3.620070	2.433426
H	0.487647	4.310300	0.870177
C	1.458752	-1.026178	-2.033456
C	0.020251	0.956622	-2.092505
H	1.881972	2.780983	-0.447655
O	2.420684	3.455472	-0.969587
C	3.538963	3.853192	-0.188761
H	4.204647	2.983540	0.045689
H	3.216137	4.274750	0.797112
H	3.291753	0.401051	3.605709
H	5.747934	-1.670256	0.681966
C	4.336799	4.909968	-0.945765
H	5.211952	5.247996	-0.352921
H	4.707872	4.508838	-1.912447
H	3.703008	5.794860	-1.165702
N	4.304658	-0.759669	-1.424654
C	5.298081	-1.744981	-1.827354
H	5.154434	-1.972775	-2.902591
H	6.347400	-1.387958	-1.691741
H	5.166812	-2.687597	-1.261772
C	4.140491	0.350743	-2.366679
H	5.139139	0.778769	-2.618858
H	3.662582	0.004988	-3.306128
H	3.517057	1.151152	-1.928547
H	5.251383	-1.125706	3.065354

Mn9/iii

Frequencies, energies and thermodynamic properties:

Lowest Vibrational Mode (1/cm) =	22.1081
2nd Lowest Vibrational Mode (1/cm) =	29.6201
E(RB-P86) (a.u.) =	-4541.36832638
Thermal correction to Enthalpy (a.u.) =	0.717356
Thermal correction to Gibbs Free Energy (a.u.) =	0.591853

```
 Total Entropy (cal/Kmol) =                             264.143
 E(RPBE1PBE) (a.u.) =                                  -4540.69377140
Optimised cartesian coordinates (Angstrom):
 Fe-3.458882   0.921764   0.622173
 Mn1.075950   0.457442  -1.112495
 P     -0.400063  -0.854243   0.024294
 O      1.546981  -1.760037  -3.022246
 O     -0.890847   1.475447  -3.035935
 N      0.746496   1.959623   0.297554
 N      2.714474   0.119535   0.217172
 C     -1.611139   0.105259   1.009118
 C     -1.572433   1.553896   1.181126
 C     -2.548854   1.882915   2.193267
 H     -2.786367   2.893428   2.549232
 C     -3.194030   0.674964   2.634456
 H     -3.998765   0.607094   3.379043
 C     -2.629185  -0.423832   1.900353
 H     -2.910407  -1.479563   2.005393
 C     -3.856874   1.067667  -1.397308
 H     -3.111565   1.019462  -2.200871
 C     -4.274885   2.262502  -0.714308
 H     -3.909102   3.279429  -0.910152
 C     -5.238834   1.888395   0.289221
 H     -5.733137   2.568987   0.995439
 C     -5.418346   0.459849   0.223016
 H     -6.074613  -0.137811   0.869904
 C     -4.561710  -0.048526  -0.816851
 H     -4.450498  -1.101124  -1.107873
 C      0.388252  -1.990510   1.274324
 C      1.183879  -3.053827   0.783809
 H      1.295811  -3.200191  -0.302818
 C      1.820700  -3.938125   1.669898
 H      2.424350  -4.769246   1.271600
 C      1.687238  -3.764670   3.060944
 H      2.186940  -4.458084   3.755827
 C      0.912526  -2.702361   3.556749
 H      0.801931  -2.557968   4.643366
 C      0.265439  -1.820167   2.669967
 H     -0.350083  -1.002288   3.074509
 C     -1.461249  -2.076750  -0.906407
 C     -1.754934  -1.892232  -2.274842
 H     -1.325259  -1.044261  -2.824620
 C     -2.601840  -2.787463  -2.953653
 H     -2.815909  -2.627124  -4.022279
 C     -3.168149  -3.881225  -2.276012
 H     -3.829544  -4.582557  -2.809075
 C     -2.877397  -4.078343  -0.913745
 H     -3.309567  -4.935542  -0.373189
 C     -2.027355  -3.187182  -0.236101
 H     -1.796486  -3.368292   0.824990
 C     -0.616570   2.512433   0.463905
 H     -1.005370   2.664707  -0.564155
 C      1.350501   1.619101   1.578293
 H      1.649344   2.518646   2.173284
 H      0.642246   1.066788   2.254285
 C      2.556748   0.736513   1.421998
 C      3.801193  -0.707037   0.043644
 C      4.662789  -1.012939   1.121931
 C      4.466701  -0.405760   2.367070
 C      3.417016   0.508067   2.511295
 C     -0.607969   3.904035   1.135761
 H     -1.610670   4.375815   1.082593
 H     -0.317920   3.855295   2.206108
 H      0.110868   4.559880   0.604767
 C      1.434041  -0.884164  -2.239904
 C     -0.155711   1.035314  -2.219040
 H      2.231937   1.215325  -2.120611
 H      2.019481   1.854622  -1.631251
 H      1.587029   3.163828  -0.365183
 O      2.103779   3.897491  -0.910104
 C      3.353297   4.147594  -0.294213
 H      3.227157   4.503296   0.761765
 H      3.977353   3.217997  -0.230508
 H      3.235129   1.030765   3.461870
 H      5.517263  -1.683151   0.956840
 C      4.119283   5.206631  -1.083919
 H      3.536915   6.150962  -1.136220
 H      5.099613   5.429078  -0.612580
 H      4.304470   4.865146  -2.124482
 N      4.034410  -1.239949  -1.231658
 C      4.671153  -2.547660  -1.322366
 H      4.458673  -2.974404  -2.324905
 H      5.782824  -2.512771  -1.202933
 H      4.252620  -3.233981  -0.561551
 C      4.496544  -0.314860  -2.270224
```

```
 H      5.612505  -0.259621  -2.288327
 H      4.145845  -0.646962  -3.269661
 H      4.112895   0.702915  -2.083313
 H      5.149195  -0.619476   3.204501
---------------------------------------
Mn9/iv
Frequencies, energies and thermodynamic properties:
 Lowest Vibrational Mode (1/cm) =                        15.8860
 2nd Lowest Vibrational Mode (1/cm) =                    19.7578
 E(RB-P86) (a.u.) =                                     -4541.39672019
 Thermal correction to Enthalpy (a.u.) =                 0.721663
 Thermal correction to Gibbs Free Energy (a.u.) =        0.594879
 Total Entropy (cal/Kmol) =                              266.839
 E(RPBE1PBE) (a.u.) =                                   -4540.72099008
Optimised cartesian coordinates (Angstrom):
 Fe-3.490062   0.841109   0.668352
 Mn1.109034   0.583261  -0.897082
 P     -0.421345  -0.887084  -0.061635
 O      1.700419  -1.182659  -3.176822
 O     -0.631945   2.004396  -2.777343
 N      0.705773   1.877017   0.763257
 N      2.696511   0.059720   0.431238
 C     -1.685236  -0.082439   1.013950
 C     -1.644228   1.318604   1.432200
 C     -2.672985   1.498268   2.431878
 H     -2.918558   2.438825   2.940725
 C     -3.356046   0.247982   2.624329
 H     -4.205083   0.075750   3.299431
 C     -2.759076  -0.723603   1.750590
 H     -3.059980  -1.775129   1.658332
 C     -3.731353   1.361903  -1.310913
 H     -2.921589   1.453998  -2.046328
 C     -4.207435   2.413498  -0.451422
 H     -3.832454   3.445570  -0.425158
 C     -5.246005   1.870046   0.386321
 H     -5.796961   2.412870   1.166093
 C     -5.414258   0.481356   0.041270
 H     -6.116439  -0.219386   0.512633
 C     -4.476747   0.165938  -1.004930
 H     -4.336530  -0.816014  -1.474741
 C      0.268235  -2.226793   1.042375
 C      1.116461  -3.187335   0.438372
 H      1.288835  -3.155496  -0.650041
 C      1.733827  -4.186384   1.208418
 H      2.378605  -4.933222   0.717834
 C      1.532721  -4.232683   2.601881
 H      2.019368  -5.014227   3.206782
 C      0.707493  -3.274283   3.214091
 H      0.543086  -3.300581   4.303394
 C      0.078028  -2.279133   2.440687
 H     -0.578771  -1.547953   2.936992
 C     -1.457448  -1.919414  -1.224350
 C     -1.673020  -1.490865  -2.552244
 H     -1.206927  -0.562612  -2.910933
 C     -2.486199  -2.238046  -3.424055
 H     -2.641498  -1.887711  -4.457043
 C     -3.094531  -3.426789  -2.983100
 H     -3.729117  -4.012607  -3.667095
 C     -2.880661  -3.866974  -1.663947
 H     -3.346870  -4.800220  -1.309160
 C     -2.065478  -3.121945  -0.793533
 H     -1.894252  -3.489999   0.230150
 C     -0.693787   2.399648   0.933628
 H     -1.001568   2.691099  -0.090059
 C      1.271515   1.300151   1.995043
 H      1.500666   2.070890   2.764348
 H      0.507398   0.630663   2.448258
 C      2.505768   0.481336   1.714618
 C      3.811871  -0.707666   0.172160
 C      4.652839  -1.153021   1.223330
 C      4.415672  -0.744956   2.537552
 C      3.339289   0.117970   2.783961
 C     -0.725257   3.668448   1.802899
 H     -1.729138   4.136773   1.768341
 H     -0.484475   3.462652   2.865975
 H      0.001752   4.408341   1.410796
 C      1.520054  -0.492186  -2.229929
 C      0.013494   1.398927  -1.982145
 H      2.124783   1.710367  -1.423005
 H      2.163236   3.138751  -1.095869
 H      1.281303   2.702333   0.492809
 O      2.178384   4.007896  -0.585876
 C      3.547445   4.330529  -0.322097
 H      3.542947   5.091504   0.488531
 H      4.095677   3.442725   0.075147
```

```
 H      3.121271   0.493770   3.794481
 H      5.527124  -1.774077   0.987093
 C      4.266778   4.882770  -1.552436
 H      3.738703   5.775311  -1.948048
 H      5.308060   5.177497  -1.302415
 H      4.313851   4.124331  -2.362521
 N      4.105512  -1.043521  -1.154084
 C      4.862997  -2.263542  -1.398738
 H      4.723914  -2.555671  -2.460297
 H      5.962918  -2.151942  -1.225841
 H      4.486481  -3.088561  -0.763220
 C      4.485386   0.052156  -2.049834
 H      5.564200   0.317845  -1.925713
 H      4.313203  -0.250786  -3.102491
 H      3.855752   0.938310  -1.843878
 H      5.082851  -1.066478   3.352330
---------------------------------------
Mn9/v
Frequencies, energies and thermodynamic properties:
 Lowest Vibrational Mode (1/cm) =                        19.6732
 2nd Lowest Vibrational Mode (1/cm) =                    34.0279
 E(RB-P86) (a.u.) =                                    -4386.45866100
 Thermal correction to Enthalpy (a.u.) =                 0.637322
 Thermal correction to Gibbs Free Energy (a.u.) =        0.525625
 Total Entropy (cal/Kmol) =                              235.085
 E(RPBE1PBE) (a.u.) =                                  -4385.77799462
Optimised cartesian coordinates (Angstrom):
 Fe3.406489  -0.682557   0.369539
 Mn-1.057548  -0.393765  -1.510524
 P      0.026007   0.551591   0.256095
 O     -2.017305   2.214967  -2.477957
 O      1.014455  -0.113726  -3.561606
 N     -0.403773  -2.315405  -0.825931
 N     -2.776853  -1.068136  -0.440365
 C      1.422131  -0.485954   0.875882
 C      1.714801  -1.844213   0.416200
 C      2.730702  -2.384942   1.291909
 H      3.186488  -3.379866   1.212781
 C      3.082346  -1.387218   2.265124
 H      3.846293  -1.492442   3.047185
 C      2.288125  -0.217942   2.009281
 H      2.325473   0.720543   2.577412
 C      3.815817   0.124540  -1.481129
 H      3.070527   0.345289  -2.256237
 C      4.504999  -1.127869  -1.313269
 H      4.385090  -2.020402  -1.942158
 C      5.363221  -1.014874  -0.161572
 H      6.006441  -1.806943   0.244813
 C      5.206365   0.310518   0.381091
 H      5.709786   0.705185   1.273885
 C      4.248596   1.013936  -0.431882
 H      3.893030   2.039946  -0.272218
 C     -0.995009   0.824542   1.797523
 C     -2.051642   1.764199   1.707190
 H     -2.192374   2.338007   0.776209
 C     -2.918833   1.978573   2.790683
 H     -3.725872   2.723955   2.704720
 C     -2.761095   1.241912   3.981122
 H     -3.443182   1.407096   4.830275
 C     -1.728427   0.293702   4.075672
 H     -1.596106  -0.289370   5.001410
 C     -0.850831   0.086735   2.993160
 H     -0.038123  -0.649350   3.093558
 C      0.808943   2.239317   0.079100
 C      1.163184   2.720577  -1.200228
 H      0.951496   2.111672  -2.090442
 C      1.789404   3.971747  -1.347985
 H      2.057211   4.330748  -2.354687
 C      2.068483   4.763215  -0.219389
 H      2.556460   5.744064  -0.336040
 C      1.712648   4.296923   1.059533
 H      1.920256   4.911447   1.950324
 C      1.084308   3.047442   1.206920
 H      0.796430   2.704503   2.213102
 C      1.074053  -2.583061  -0.752685
 H      1.472056  -2.161701  -1.697133
 C     -1.137425  -2.687173   0.397680
 H     -1.189577  -3.787867   0.550436
 H     -0.575466  -2.278955   1.265961
 C     -2.528140  -2.103963   0.411829
 C     -4.047337  -0.531492  -0.433324
 C     -5.006897  -0.939041   0.529161
 H     -6.005473  -0.482402   0.521966
 C     -4.712523  -1.966168   1.427904
 C     -3.458790  -2.587991   1.343461
```

```
 H     -3.183737  -3.424363   2.002919
 C      1.386009  -4.088874  -0.743165
 H      2.473409  -4.257522  -0.873952
 H      1.078642  -4.581090   0.201986
 H      0.871444  -4.594692  -1.586252
 C     -1.687036   1.152683  -2.063738
 C      0.225627  -0.210212  -2.674892
 H     -0.767472  -2.874899  -1.609339
 H     -1.658082  -1.221278  -2.727724
 H     -5.464682  -2.302887   2.158124
 N     -4.378686   0.425152  -1.396448
 C     -4.366440  -0.004499  -2.797267
 H     -5.311346  -0.540001  -3.062127
 H     -3.493832  -0.663382  -2.974018
 H     -4.263066   0.881219  -3.456142
 C     -5.451646   1.362211  -1.094824
 H     -5.366992   2.226278  -1.785897
 H     -5.357879   1.741240  -0.058448
 H     -6.475568   0.930055  -1.225343
---------------------------------------
Mn9/vi_R
Frequencies, energies and thermodynamic properties:
 Lowest Vibrational Mode (1/cm) =                          9.2591
 2nd Lowest Vibrational Mode (1/cm) =                      15.9839
 E(RB-P86) (a.u.) =                                       -4809.17061051
 Thermal correction to Enthalpy (a.u.) =                   0.791265
 Thermal correction to Gibbs Free Energy (a.u.) =          0.656055
 Total Entropy (cal/Kmol) =                                284.574
 E(RPBE1PBE) (a.u.) =                                     -4808.48439393
Optimised cartesian coordinates (Angstrom):
 Fe-2.216030  -2.982401  -0.845969
 Mn0.499359   0.754949   0.410567
 P     -1.649650   0.354420   0.329772
 O      0.252829   1.915262   3.128240
 O      1.219543  -1.692169   1.847758
 N      0.780833   0.053619  -1.386283
 N      0.794148   2.581316  -0.664573
 C     -2.057734  -0.936238  -0.907736
 C     -1.083193  -1.547927  -1.804522
 C     -1.822559  -2.340735  -2.758295
 H     -1.387761  -2.948311  -3.562081
 C     -3.226911  -2.236749  -2.461541
 H     -4.039169  -2.745604  -2.998094
 C     -3.378314  -1.380871  -1.316541
 H     -4.328165  -1.103571  -0.841221
 C     -1.314393  -3.787307   0.824114
 H     -0.569141  -3.282010   1.451794
 C     -1.028647  -4.587261  -0.337035
 H     -0.031032  -4.803576  -0.742383
 C     -2.281433  -5.034402  -0.890909
 H     -2.406562  -5.646707  -1.794046
 C     -3.342922  -4.512493  -0.067908
 H     -4.418713  -4.658300  -0.234413
 C     -2.746321  -3.739225   0.990267
 H     -3.284436  -3.193442   1.776105
 C     -2.565166   1.853735  -0.287275
 C     -2.628628   2.983697   0.564075
 H     -2.182422   2.937854   1.570779
 C     -3.261942   4.162414   0.139262
 H     -3.314134   5.028901   0.817619
 C     -3.826398   4.238085  -1.149264
 H     -4.321285   5.163971  -1.482828
 C     -3.753834   3.127322  -2.006515
 H     -4.191659   3.178068  -3.016251
 C     -3.127899   1.939908  -1.579788
 H     -3.092846   1.072610  -2.256261
 C     -2.651769  -0.097075   1.830542
 C     -2.041564  -0.716475   2.943054
 H     -0.959805  -0.909659   2.944492
 C     -2.809607  -1.096615   4.058549
 H     -2.317147  -1.576712   4.919042
 C     -4.196062  -0.862178   4.078636
 H     -4.795611  -1.158116   4.954091
 C     -4.811894  -0.239928   2.977420
 H     -5.896224  -0.045332   2.985935
 C     -4.045488   0.144283   1.863190
 H     -4.539300   0.646978   1.016975
 C      0.435696  -1.345167  -1.737205
 H      0.816595  -1.958400  -0.895642
 C      0.483194   0.984136  -2.465296
 H      1.168448   0.869082  -3.341880
 H     -0.542148   0.809339  -2.883358
 C      0.559109   2.407300  -1.995381
 C      0.965869   3.866157  -0.191318
 C      0.714199   4.980086  -1.031251
```

```
 C      0.399892   4.780480  -2.379652
 C      0.362506   3.475160  -2.886175
 H      0.163337   3.271103  -3.948300
 C      1.134130  -1.858210  -3.012947
 H      0.992956  -2.953558  -3.112344
 H      0.737841  -1.385635  -3.935215
 H      2.221466  -1.656345  -2.946073
 C      0.385049   1.515945   2.022105
 C      0.864265  -0.730499   1.246677
 H      2.557931  -0.010400  -1.411098
 H      3.480243   0.158757   0.477957
 C      4.220478  -0.076372  -0.335566
 C      4.938418  -1.368598   0.051168
 C      5.375635   0.962642  -0.294813
 C      6.172609  -1.081615   0.675326
 C      4.517566  -2.694502  -0.132585
 C      6.387216   0.421031   0.744282
 H      5.834825   0.962749  -1.307796
 C      6.996134  -2.126251   1.128851
 C      5.344517  -3.742638   0.319187
 H      3.558402  -2.911002  -0.630255
 H      7.436712   0.721990   0.542307
 C      6.574916  -3.459485   0.946686
 H      7.964167  -1.911156   1.611437
 H      5.031706  -4.789956   0.177518
 H      7.215904  -4.287386   1.290891
 O      3.551692  -0.112614  -1.577603
 H      5.024573   1.992813  -0.084576
 H      6.143451   0.795137   1.765589
 H      0.229211   5.645611  -3.039374
 H      0.833128   5.998249  -0.639104
 N      1.396992   4.040302   1.118498
 C      1.172923   5.326281   1.765572
 C      2.641434   3.391779   1.540608
 H      0.149157   5.692845   1.558364
 H      1.272537   5.189999   2.861571
 H      1.905033   6.111846   1.455429
 H      2.609489   3.179131   2.627665
 H      2.772850   2.432599   1.010164
 H      3.520393   4.047734   1.330345
---------------------------------------
Mn9/vi_S
Frequencies, energies and thermodynamic properties:
 Lowest Vibrational Mode (1/cm) =                         10.8159
 2nd Lowest Vibrational Mode (1/cm) =                     17.6357
 E(RB-P86) (a.u.) =                                      -4809.17182546
 Thermal correction to Enthalpy (a.u.) =                  0.791239
 Thermal correction to Gibbs Free Energy (a.u.) =         0.655489
 Total Entropy (cal/Kmol) =                               285.709
 E(RPBE1PBE) (a.u.) =                                    -4808.48444891
Optimised cartesian coordinates (Angstrom):
 Fe-3.203352  -2.312645  -0.802621
 Mn0.558595   0.382040   0.429732
 P     -1.603165   0.698298   0.314105
 O      0.690082   1.651016   3.106955
 O      0.416948  -2.125504   1.933739
 N      0.612401  -0.414686  -1.347937
 N      1.462691   1.972409  -0.676217
 C     -2.395562  -0.426239  -0.899422
 C     -1.662376  -1.339349  -1.769619
 C     -2.609040  -1.873283  -2.720012
 H     -2.385841  -2.606580  -3.505429
 C     -3.907928  -1.317173  -2.446109
 H     -4.836314  -1.550517  -2.984849
 C     -3.785704  -0.432955  -1.319219
 H     -4.599935   0.144668  -0.862723
 C     -2.616514  -3.333695   0.889720
 H     -1.751319  -3.082535   1.516605
 C     -2.597508  -4.203949  -0.255730
 H     -1.720160  -4.735502  -0.648206
 C     -3.924968  -4.235068  -0.815337
 H     -4.235787  -4.790903  -1.710137
 C     -4.766334  -3.384974  -0.011702
 H     -5.831113  -3.180480  -0.187032
 C     -3.957836  -2.825096   1.040138
 H     -4.296351  -2.122003   1.812216
 C     -1.971300   2.395050  -0.359958
 C     -1.663096   3.509241   0.458239
 H     -1.259306   3.351024   1.471464
 C     -1.874899   4.816694  -0.007273
 H     -1.641799   5.672971   0.645516
 C     -2.380637   5.033221  -1.304168
 H     -2.544292   6.059356  -1.669848
 C     -2.674384   3.934090  -2.128500
 H     -3.069598   4.094275  -3.144367
```

```
 C     -2.472527   2.620905  -1.660892
 H     -2.722319   1.769584  -2.311936
 C     -2.716224   0.645025   1.803202
 C     -2.354365  -0.103121   2.944648
 H     -1.394273  -0.636884   2.974291
 C     -3.217711  -0.176956   4.052767
 H     -2.918867  -0.762981   4.936353
 C     -4.452291   0.496244   4.036370
 H     -5.125951   0.439768   4.906110
 C     -4.818762   1.249039   2.905829
 H     -5.780686   1.785694   2.885473
 C     -3.955793   1.327044   1.798754
 H     -4.249083   1.934762   0.928540
 C     -0.160049  -1.635058  -1.678854
 H     -0.007214  -2.319536  -0.820134
 C      0.637660   0.538642  -2.448318
 H      1.258129   0.191430  -3.312150
 H     -0.385522   0.691695  -2.879524
 C      1.164744   1.871278  -2.001554
 C      2.061042   3.129494  -0.222195
 C      2.162886   4.262967  -1.068846
 C      1.771877   4.166530  -2.408239
 C      1.308380   2.939187  -2.901639
 H      1.038243   2.804790  -3.959153
 C      0.355104  -2.372855  -2.930995
 H     -0.119373  -3.371815  -3.011753
 H      0.138151  -1.822535  -3.869584
 H      1.450628  -2.517048  -2.847089
 C      0.688449   1.188551   2.017724
 C      0.404834  -1.115590   1.305850
 H      2.292578  -1.040141  -1.299450
 H      3.197753  -1.182136   0.649622
 C      3.700438  -1.804801  -0.141210
 C      3.511956  -3.305331   0.229710
 C      4.655471  -3.621838   1.226138
 O      3.221337  -1.427042  -1.413887
 H      1.875161   5.037092  -3.074839
 H      2.613688   5.189973  -0.692033
 N      2.569524   3.146605   1.067836
 C      2.835349   4.427113   1.708587
 C      3.490282   2.083548   1.478885
 H      1.999802   5.131682   1.532798
 H      2.918963   4.262797   2.801938
 H      3.785540   4.903863   1.364194
 H      3.362096   1.856486   2.556162
 H      3.286969   1.163227   0.904661
 H      4.548359   2.391685   1.302122
 C      5.205003  -1.623448   0.009312
 C      5.750553  -2.657207   0.802862
 C      6.022295  -0.612269  -0.518417
 C      7.126430  -2.677314   1.087238
 C      7.403479  -0.635818  -0.237209
 H      5.587844   0.176625  -1.153510
 C      7.950349  -1.659923   0.562578
 H      7.562632  -3.482755   1.701361
 H      8.062010   0.145865  -0.649681
 H      9.032537  -1.671207   0.771144
 H      3.652070  -3.890619  -0.705547
 H      2.501420  -3.523829   0.628514
 H      4.978871  -4.683917   1.212099
 H      4.337514  -3.403483   2.272040
---------------------------------------
Mn9/viii
Frequencies, energies and thermodynamic properties:
 Lowest Vibrational Mode (1/cm) =                        21.3167
 2nd Lowest Vibrational Mode (1/cm) =                    29.7817
 E(RB-P86) (a.u.) =                                     -4386.42413055
 Thermal correction to Enthalpy (a.u.) =                 0.632601
 Thermal correction to Gibbs Free Energy (a.u.) =        0.519997
 Total Entropy (cal/Kmol) =                              236.994
 E(RPBE1PBE) (a.u.) =                                   -4385.74439566
Optimised cartesian coordinates (Angstrom):
 Fe3.416965  -0.659279   0.400294
 Mn-1.038766  -0.427986  -1.560406
 P      0.024956   0.497495   0.210701
 O     -2.033281   2.231719  -2.420633
 O      1.162124   0.018897  -3.446054
 N     -0.379834  -2.261485  -0.913926
 N     -2.753074  -1.097138  -0.454634
 C      1.410122  -0.528228   0.840956
 C      1.736457  -1.863401   0.350730
 C      2.735864  -2.405550   1.240749
 H      3.212003  -3.389913   1.144469
 C      3.040908  -1.430808   2.255157
 H      3.779621  -1.544136   3.060397
```

```
 C      2.235268  -0.266080   2.007756
 H      2.237526   0.654249   2.605916
 C      3.882081   0.231564  -1.401031
 H      3.163091   0.484883  -2.190035
 C      4.570161  -1.024547  -1.267858
 H      4.472702  -1.888285  -1.939231
 C      5.388419  -0.961661  -0.083129
 H      6.022258  -1.769026   0.307756
 C      5.207707   0.337918   0.513716
 H      5.681050   0.693565   1.438881
 C      4.274133   1.074603  -0.298477
 H      3.909248   2.091807  -0.105442
 C     -1.059963   0.739357   1.707786
 C     -2.070657   1.727763   1.632505
 H     -2.175226   2.337176   0.720154
 C     -2.934203   1.951837   2.717301
 H     -3.706077   2.734996   2.647814
 C     -2.813321   1.180450   3.889260
 H     -3.490555   1.356162   4.740130
 C     -1.823618   0.185861   3.966350
 H     -1.721441  -0.423515   4.878602
 C     -0.950857  -0.034118   2.883009
 H     -0.172301  -0.807966   2.963266
 C      0.770018   2.205559   0.073627
 C      1.123077   2.746131  -1.181770
 H      0.925040   2.181876  -2.102916
 C      1.734257   4.010359  -1.272422
 H      1.999419   4.415237  -2.262067
 C      2.001724   4.754605  -0.110201
 H      2.478730   5.744950  -0.182506
 C      1.647813   4.228851   1.145904
 H      1.845345   4.805566   2.063656
 C      1.032596   2.968240   1.236558
 H      0.744782   2.581852   2.226502
 C      1.053747  -2.545800  -0.845499
 H      1.501666  -2.117106  -1.766336
 C     -1.094729  -2.748301   0.239782
 H     -1.206359  -3.865860   0.257591
 H     -0.582484  -2.521533   1.222055
 C     -2.471229  -2.157257   0.353583
 C     -4.007499  -0.534107  -0.379546
 C     -4.937118  -0.954287   0.598308
 C     -4.619117  -2.015296   1.455248
 C     -3.380821  -2.647976   1.309237
 C      1.369051  -4.058923  -0.877244
 H      2.456583  -4.239463  -1.006252
 H      1.053132  -4.576818   0.052472
 H      0.838974  -4.528391  -1.731029
 C     -1.714834   1.149223  -2.069788
 C      0.320324  -0.144742  -2.627659
 H     -1.547932  -1.713967  -2.625303
 H     -1.893256  -1.063298  -2.989176
 H     -5.928200  -0.482801   0.640573
 H     -3.091962  -3.502470   1.939115
 N     -4.343454   0.466064  -1.302856
 C     -4.517045   0.052362  -2.697860
 H     -5.567071  -0.278905  -2.890937
 H     -4.282539   0.895087  -3.380541
 H     -3.844510  -0.789013  -2.937940
 C     -5.309297   1.477882  -0.895477
 H     -5.206230   2.354966  -1.567987
 H     -6.372040   1.133343  -0.957449
 H     -5.104242   1.812468   0.139577
 H     -5.347762  -2.363178   2.204243
---------------------------------------
Mn9/ix
Frequencies, energies and thermodynamic properties:
 Lowest Vibrational Mode (1/cm) =                         21.2526
 2nd Lowest Vibrational Mode (1/cm) =                     30.9617
 E(RB-P86) (a.u.) =                                     -4540.21614648
 Thermal correction to Enthalpy (a.u.) =                  0.701896
 Thermal correction to Gibbs Free Energy (a.u.) =         0.581268
 Total Entropy (cal/Kmol) =                               253.883
 E(RPBE1PBE) (a.u.) =                                   -4539.54052681
Optimised cartesian coordinates (Angstrom):
 Fe3.517607   0.372781  -0.745438
 Mn-1.088094   1.005414   0.694948
 P      0.221052  -0.766086   0.166233
 O     -1.962803  -0.315150   3.179163
 O      0.953426   2.246715   2.388631
 N     -0.476578   1.933002  -1.113201
 N     -2.683576   0.406788  -0.667101
 C      1.587503  -0.303101  -0.989387
 C      1.763306   1.010940  -1.608435
 C      2.805780   0.883456  -2.600781
```

```
 H      3.191140   1.691332  -3.235510
 C      3.287626  -0.470853  -2.595525
 H      4.098470  -0.868885  -3.220291
 C      2.550765  -1.200799  -1.601531
 H      2.688705  -2.260751  -1.353291
 C      3.862730   1.090394   1.157360
 H      3.087690   1.381402   1.877458
 C      4.462301   1.955159   0.175974
 H      4.232144   3.018569   0.025568
 C      5.401201   1.176761  -0.590873
 H      6.007131   1.540305  -1.431650
 C      5.383272  -0.170576  -0.080204
 H      5.973621  -1.013192  -0.464591
 C      4.430328  -0.225184   0.997847
 H      4.165758  -1.114970   1.583770
 C     -0.629281  -2.161744  -0.738833
 C     -1.549303  -2.947018  -0.002542
 H     -1.709769  -2.741159   1.068437
 C     -2.251766  -3.994929  -0.619187
 H     -2.952405  -4.605083  -0.026855
 C     -2.062626  -4.264750  -1.988661
 H     -2.613836  -5.086469  -2.472889
 C     -1.167249  -3.478147  -2.732607
 H     -1.013475  -3.679038  -3.805038
 C     -0.453969  -2.433482  -2.112628
 H      0.255970  -1.839188  -2.708030
 C      1.102272  -1.747290   1.491937
 C      1.336517  -1.192634   2.768961
 H      0.973943  -0.184753   3.009440
 C      2.037766  -1.918144   3.749882
 H      2.207241  -1.466909   4.740532
 C      2.515393  -3.210606   3.471635
 H      3.062673  -3.778312   4.240911
 C      2.280705  -3.777309   2.205368
 H      2.642460  -4.792674   1.977022
 C      1.576270  -3.054526   1.226990
 H      1.384091  -3.523533   0.249698
 C      0.962316   2.273604  -1.307106
 H      1.290509   2.674704  -0.327413
 C     -1.117453   1.361532  -2.304880
 H     -1.330235   2.130625  -3.081535
 H     -0.409253   0.643932  -2.775414
 C     -2.393173   0.623758  -1.976386
 C     -3.858393  -0.244158  -0.364452
 C     -4.652517  -0.823319  -1.387981
 C     -4.308425  -0.634937  -2.728557
 C     -3.180838   0.137686  -3.034354
 C      1.175794   3.379399  -2.354786
 H      2.231223   3.717979  -2.348924
 H      0.935270   3.041769  -3.383480
 H      0.542298   4.258156  -2.116655
 C     -1.680579   0.218441   2.160194
 C      0.165602   1.709866   1.679543
 H     -1.024630   2.757603  -0.757241
 H     -2.887833   0.345353  -4.074079
 H     -5.573842  -1.359203  -1.124988
 N     -4.260435  -0.304239   0.968147
 C     -5.193463  -1.349502   1.363852
 H     -5.162425  -1.446716   2.468495
 H     -6.253072  -1.132525   1.077622
 H     -4.899963  -2.323200   0.925475
 C     -4.500368   0.978589   1.643081
 H     -5.545257   1.327806   1.450765
 H     -4.368905   0.856118   2.737072
 H     -3.775711   1.733265   1.269066
 H     -4.938934  -1.054708  -3.528023
 O     -2.128341   2.761442   0.661277
 C     -2.127500   3.680678   1.716761
 H     -1.091353   3.980791   2.028610
 H     -2.596813   3.243527   2.642150
 C     -2.901176   4.949838   1.333463
 H     -3.955837   4.712539   1.076472
 H     -2.907391   5.684975   2.167407
 H     -2.440939   5.438689   0.447741
---------------------------------------
Mn9/x
Frequencies, energies and thermodynamic properties:
 Lowest Vibrational Mode (1/cm) =                         8.8855
 2nd Lowest Vibrational Mode (1/cm) =                     20.1028
 E(RB-P86) (a.u.) =                                      -4695.15277805
 Thermal correction to Enthalpy (a.u.) =                  0.786792
 Thermal correction to Gibbs Free Energy (a.u.) =         0.651569
 Total Entropy (cal/Kmol) =                               284.601
 E(RPBE1PBE) (a.u.) =                                    -4694.49002942
Optimised cartesian coordinates (Angstrom):
```

```
Fe-3.574342   1.136540   0.742642
Mn0.967886   0.405229  -0.895863
P     -0.688920  -0.881691  -0.023842
O      1.276779  -1.534890  -3.089394
O     -0.861604   1.791890  -2.715503
N      0.701844   1.747980   0.746103
N      2.452958  -0.340138   0.510495
C     -1.841415   0.072263   1.056738
C     -1.669737   1.473301   1.440152
C     -2.648518   1.762158   2.463272
H     -2.797147   2.731547   2.955282
C     -3.427914   0.579098   2.706223
H     -4.267703   0.494085   3.408839
C     -2.943846  -0.458562   1.838850
H     -3.336666  -1.481661   1.782992
C     -3.870252   1.592750  -1.246902
H     -3.097981   1.568079  -2.025793
C     -4.188751   2.724634  -0.417603
H     -3.710228   3.712275  -0.461242
C     -5.228945   2.328908   0.497068
H     -5.677866   2.958957   1.276624
C     -5.555103   0.950977   0.230220
H     -6.296593   0.347962   0.771335
C     -4.713613   0.494717  -0.845236
H     -4.700157  -0.516232  -1.272860
C     -0.123511  -2.270240   1.089125
C      0.545183  -3.359366   0.479415
H      0.686040  -3.373078  -0.613531
C      1.019722  -4.431628   1.252033
H      1.522528  -5.279019   0.758962
C      0.852826  -4.425671   2.650449
H      1.225062  -5.266832   3.256527
C      0.209159  -3.339603   3.266836
H      0.074914  -3.323667   4.360300
C     -0.277534  -2.268531   2.492034
H     -0.797219  -1.436531   2.991513
C     -1.851637  -1.830665  -1.139612
C     -2.048495  -1.447316  -2.484062
H     -1.491080  -0.601683  -2.906845
C     -2.961219  -2.138876  -3.301894
H     -3.096993  -1.824290  -4.348913
C     -3.691873  -3.225367  -2.790434
H     -4.404710  -3.767017  -3.432310
C     -3.499013  -3.621112  -1.454245
H     -4.059598  -4.475700  -1.042766
C     -2.583865  -2.933580  -0.638417
H     -2.432331  -3.271695   0.398169
C     -0.618418   2.440945   0.912372
H     -0.901995   2.750915  -0.113210
C      1.220764   1.180299   2.002214
H      1.603211   1.966938   2.688772
H      0.382378   0.683444   2.537558
C      2.299639   0.153360   1.769007
C      3.409072  -1.313113   0.313105
C      4.124195  -1.863695   1.408542
C      3.932261  -1.361419   2.696697
C      3.025270  -0.310533   2.879467
C     -0.499091   3.715367   1.765262
H     -1.436743   4.303885   1.712882
H     -0.298351   3.493131   2.833110
H      0.321922   4.354476   1.381286
C      1.222267  -0.768441  -2.189307
C     -0.162064   1.214490  -1.949425
H      3.081925   2.660001  -0.258351
H      1.396582   2.457133   0.440477
O      3.242237   3.369633   0.473107
C      4.458054   3.116782   1.156751
H      4.443794   3.713369   2.098362
H      4.540523   2.045731   1.472476
H      2.849659   0.135341   3.869384
H      4.874714  -2.644081   1.227872
C      5.694845   3.498926   0.336681
H      5.649889   4.566969   0.035524
H      6.628375   3.341693   0.918910
H      5.763918   2.886648  -0.587705
O      2.464265   1.762847  -1.366938
C      2.395621   2.646339  -2.467060
C      2.664238   2.005384  -3.834269
H      3.151486   3.456795  -2.304415
H      1.408212   3.173969  -2.515798
H      2.591534   2.768617  -4.639623
H      3.679525   1.558539  -3.881479
H      1.929491   1.204384  -4.057222
H      4.509180  -1.765025   3.543456
N      3.666374  -1.754409  -0.984551
```

```
 C      4.251118  -3.078012  -1.155416
 C      4.190434  -0.768477  -1.937671
 H      5.352101  -3.107468  -0.958960
 H      3.752483  -3.815011  -0.496472
 H      4.095144  -3.394862  -2.207100
 H      3.959913  -1.093694  -2.972262
 H      3.716524   0.220164  -1.762647
 H      5.301092  -0.681705  -1.835894
---------------------------------------
Mn9/TS-i
Frequencies, energies and thermodynamic properties:
 Lowest Vibrational Mode (1/cm) =-737.0869
 2nd Lowest Vibrational Mode (1/cm) =                      20.3958
 E(RB-P86) (a.u.) =                                     -4541.36619420
 Thermal correction to Enthalpy (a.u.) =                 0.713567
 Thermal correction to Gibbs Free Energy (a.u.) =        0.589582
 Total Entropy (cal/Kmol) =                              260.948
 E(RPBE1PBE) (a.u.) =                                   -4540.68941830
Optimised cartesian coordinates (Angstrom):
 Fe-3.475144   0.847778   0.635999
 Mn1.078789   0.524487  -1.071556
 P     -0.377742  -0.854764   0.007512
 O      1.616208  -1.599437  -3.059967
 O     -0.880611   1.558319  -2.994324
 N      0.700694   1.982655   0.389287
 N      2.710436   0.182712   0.264609
 C     -1.617215   0.051539   1.012261
 C     -1.609936   1.494816   1.234136
 C     -2.600245   1.772127   2.248190
 H     -2.859959   2.765435   2.635598
 C     -3.224728   0.537674   2.642266
 H     -4.034508   0.429696   3.376453
 C     -2.632027  -0.524650   1.877307
 H     -2.893905  -1.588408   1.943905
 C     -3.855847   1.045511  -1.381955
 H     -3.101899   1.031360  -2.178841
 C     -4.299004   2.213825  -0.669331
 H     -3.947280   3.241447  -0.832335
 C     -5.266978   1.796567   0.312916
 H     -5.778429   2.448999   1.033321
 C     -5.423764   0.368086   0.204171
 H     -6.076698  -0.257759   0.827292
 C     -4.549221  -0.097246  -0.840854
 H     -4.418568  -1.139356  -1.160165
 C      0.420889  -2.009375   1.234041
 C      1.245625  -3.039620   0.721280
 H      1.369514  -3.152771  -0.368035
 C      1.896636  -3.932680   1.587919
 H      2.522293  -4.738464   1.171926
 C      1.749453  -3.800600   2.982187
 H      2.260533  -4.500853   3.661750
 C      0.946167  -2.770916   3.500546
 H      0.824392  -2.658841   4.589777
 C      0.284237  -1.880538   2.632957
 H     -0.354715  -1.090191   3.055587
 C     -1.407992  -2.069524  -0.966416
 C     -1.696270  -1.847025  -2.330411
 H     -1.281644  -0.972160  -2.848857
 C     -2.518605  -2.738015  -3.044070
 H     -2.729092  -2.547823  -4.108506
 C     -3.064980  -3.865420  -2.406309
 H     -3.707003  -4.563440  -2.966705
 C     -2.779290  -4.100273  -1.048959
 H     -3.196132  -4.983852  -0.539738
 C     -1.954048  -3.213037  -0.336342
 H     -1.726771  -3.423127   0.720220
 C     -0.682437   2.501992   0.556581
 H     -1.057123   2.668684  -0.473894
 C      1.313511   1.619951   1.667199
 H      1.587558   2.510845   2.282027
 H      0.601782   1.039759   2.306999
 C      2.537882   0.765058   1.484895
 C      3.813756  -0.618947   0.076718
 C      4.676854  -0.933665   1.152267
 C      4.466066  -0.360410   2.410085
 C      3.397416   0.528838   2.571996
 C     -0.703565   3.874757   1.260027
 H     -1.714182   4.328366   1.207649
 H     -0.420214   3.807854   2.330880
 H      0.004948   4.555845   0.747081
 C      1.474641  -0.760493  -2.242181
 C     -0.146596   1.111490  -2.179449
 H      2.209917   1.390973  -1.993310
 H      2.011055   2.094597  -1.548228
 H      1.393587   2.940492  -0.180406
```

```
 O      1.997256   3.701395  -0.899532
 C      3.269947   4.031471  -0.400085
 H      3.225507   4.320850   0.684461
 H      3.978734   3.158557  -0.442743
 H      3.201857   1.025010   3.533943
 H      5.544245  -1.583790   0.975109
 H      5.150110  -0.580204   3.244572
 N      4.064211  -1.119564  -1.207891
 C      4.734163  -2.409084  -1.320935
 H      4.537175  -2.820211  -2.333028
 H      5.844000  -2.348597  -1.195291
 H      4.329680  -3.121061  -0.576281
 C      4.511257  -0.162958  -2.224578
 H      5.623857  -0.059038  -2.214036
 H      4.198937  -0.501003  -3.234361
 H      4.072560   0.832528  -2.039270
 C      3.885646   5.191749  -1.187957
 H      3.229841   6.087011  -1.135776
 H      4.885996   5.469372  -0.791228
 H      4.001602   4.921638  -2.259403
----------------------------------------
Mn9/TS-ii_si
Frequencies, energies and thermodynamic properties:
 Lowest Vibrational Mode (1/cm) =-431.8929
 2nd Lowest Vibrational Mode (1/cm) =                      8.7398
 E(RB-P86) (a.u.) =                                     -4809.15893389
 Thermal correction to Enthalpy (a.u.) =                   0.786166
 Thermal correction to Gibbs Free Energy (a.u.) =          0.654949
 Total Entropy (cal/Kmol) =                                276.170
 E(RPBE1PBE) (a.u.) =                                   -4808.47166755
Optimised cartesian coordinates (Angstrom):
 Fe-3.340718  -1.744644  -0.818791
 Mn1.031875   0.111430   0.210640
 P     -1.115169   0.809103   0.394900
 O      1.590342   0.818654   3.015316
 O      0.568007  -2.537261   1.371230
 N      0.674522  -0.454441  -1.772608
 N      1.706382   1.877098  -0.817083
 C     -2.228599  -0.015318  -0.820151
 C     -1.780546  -0.922154  -1.874969
 C     -2.911679  -1.166043  -2.740621
 H     -2.917532  -1.818595  -3.622706
 C     -4.046996  -0.443919  -2.233992
 H     -5.058669  -0.452461  -2.661439
 C     -3.635975   0.257365  -1.049463
 H     -4.275414   0.897504  -0.428386
 C     -2.756137  -3.054519   0.661855
 H     -1.793554  -3.029130   1.188795
 C     -3.024991  -3.763799  -0.561023
 H     -2.308499  -4.379301  -1.121552
 C     -4.391939  -3.505744  -0.935822
 H     -4.897954  -3.884499  -1.833993
 C     -4.969529  -2.638469   0.059277
 H     -5.993412  -2.241162   0.051891
 C     -3.958677  -2.357102   1.045104
 H     -4.073957  -1.709805   1.924120
 C     -1.396715   2.620092   0.045245
 C     -0.932389   3.552746   1.004125
 H     -0.464844   3.192660   1.934865
 C     -1.071711   4.933228   0.786632
 H     -0.724194   5.644666   1.552965
 C     -1.654752   5.408115  -0.404338
 H     -1.762136   6.490986  -0.575471
 C     -2.098785   4.490900  -1.371579
 H     -2.555862   4.851079  -2.307212
 C     -1.973625   3.105828  -1.148392
 H     -2.348173   2.402679  -1.908124
 C     -2.043524   0.639644   2.006939
 C     -1.637708  -0.303789   2.975678
 H     -0.748591  -0.924743   2.803371
 C     -2.366494  -0.465810   4.168452
 H     -2.031809  -1.206045   4.912600
 C     -3.511539   0.312552   4.412368
 H     -4.080137   0.186148   5.347420
 C     -3.921799   1.259920   3.456439
 H     -4.813919   1.880311   3.638677
 C     -3.191788   1.425260   2.266504
 H     -3.517378   2.184412   1.538578
 C     -0.378187  -1.489051  -2.052699
 H     -0.213127  -2.263564  -1.277909
 C      0.583475   0.711872  -2.663550
 H      0.914807   0.478927  -3.699905
 H     -0.483723   1.019120  -2.744131
 C      1.359592   1.889370  -2.137394
 C      2.315495   3.008347  -0.313366
```

```
 C      2.515388   4.155517  -1.120267
 H      3.008255   5.036500  -0.688709
 C      2.153610   4.144943  -2.469076
 C      1.582615   2.980151  -2.993889
 H      1.278119   2.911055  -4.048557
 C     -0.176721  -2.160381  -3.422278
 H     -0.843453  -3.040210  -3.521140
 H     -0.393208  -1.476016  -4.267939
 H      0.869637  -2.515047  -3.511216
 C      1.409797   0.600093   1.865145
 C      0.705693  -1.465945   0.877747
 H      1.607246  -0.926239  -1.990467
 H      2.569674  -0.621182  -0.083655
 C      3.606236  -1.476043  -1.049465
 C      3.727058  -2.697139  -0.168194
 C      4.883080  -0.648450  -0.744752
 C      4.768824  -2.519170   0.768044
 C      3.034414  -3.914882  -0.264331
 C      5.378058  -1.134176   0.635415
 H      5.611818  -0.936598  -1.536245
 C      5.114372  -3.564415   1.640934
 C      3.384369  -4.962352   0.606464
 H      2.240912  -4.042473  -1.017781
 C      4.414376  -4.785076   1.555334
 H      5.929559  -3.441526   2.373133
 H      2.858360  -5.928822   0.546670
 H      4.681673  -5.614868   2.229595
 O      3.065802  -1.499870  -2.200306
 H      4.727536   0.443290  -0.831238
 H      2.335990   5.025276  -3.104737
 N      2.738063   3.016739   1.023609
 C      2.828220   4.306758   1.697471
 H      2.861979   4.127225   2.791898
 H      3.743712   4.890683   1.427745
 H      1.935849   4.922907   1.475486
 C      3.858097   2.134833   1.354380
 H      4.833261   2.578485   1.033856
 H      3.887550   1.958145   2.447519
 H      3.713010   1.162002   0.853994
 H      6.483839  -1.144144   0.729373
 H      4.998338  -0.479870   1.452058
---------------------------------------
Mn9/TS-ii_re
Frequencies, energies and thermodynamic properties:
 Lowest Vibrational Mode (1/cm) =-387.3417
 2nd Lowest Vibrational Mode (1/cm) =                     18.3157
 E(RB-P86) (a.u.) =                                      -4809.16286296
 Thermal correction to Enthalpy (a.u.) =                  0.786244
 Thermal correction to Gibbs Free Energy (a.u.) =         0.655709
 Total Entropy (cal/Kmol) =                               274.734
 E(RPBE1PBE) (a.u.) =                                    -4808.47552664
Optimised cartesian coordinates (Angstrom):
 Fe-3.860402  -1.290206  -0.720076
 Mn0.766939  -0.331027   0.440580
 P     -1.152314   0.858683   0.254307
 O      1.313121   0.785135   3.111262
 O     -0.374747  -2.545160   1.981465
 N      0.406478  -1.217397  -1.424086
 N      1.916917   0.960056  -0.816046
 C     -2.349702   0.088757  -0.915406
 C     -2.075445  -1.100195  -1.719989
 C     -3.169594  -1.249073  -2.652326
 H     -3.280766  -2.053669  -3.389955
 C     -4.116076  -0.190236  -2.429032
 H     -5.065074  -0.051331  -2.964151
 C     -3.622109   0.629790  -1.357911
 H     -4.118917   1.519008  -0.949350
 C     -3.722561  -2.354488   1.039144
 H     -2.821394  -2.444404   1.659483
 C     -4.082065  -3.224970  -0.049001
 H     -3.509481  -4.095886  -0.395476
 C     -5.312456  -2.740403  -0.620766
 H     -5.838477  -3.172790  -1.482482
 C     -5.715761  -1.570978   0.117925
 H     -6.603615  -0.956359  -0.082556
 C     -4.732522  -1.330200   1.141728
 H     -4.737632  -0.501863   1.861949
 C     -0.947940   2.578354  -0.438659
 C     -0.351864   3.557371   0.392763
 H     -0.076339   3.301304   1.428614
 C     -0.118739   4.857422  -0.084842
 H      0.327982   5.612118   0.582258
 C     -0.455121   5.196934  -1.409806
 H     -0.270652   6.216196  -1.784669
 C     -1.028594   4.226812  -2.248958
```

```
 H     -1.295745   4.481245  -3.287191
 C     -1.275782   2.926585  -1.767167
 H     -1.746270   2.186627  -2.432804
 C     -2.196778   1.228542   1.757585
 C     -2.093819   0.430057   2.917135
 H     -1.371024  -0.395982   2.954549
 C     -2.914318   0.676871   4.033331
 H     -2.817307   0.043331   4.929384
 C     -3.849431   1.726420   4.008505
 H     -4.489982   1.920218   4.883561
 C     -3.956111   2.532726   2.860317
 H     -4.680718   3.362219   2.831261
 C     -3.134011   2.288778   1.746414
 H     -3.218608   2.941470   0.863730
 C     -0.856929  -2.007605  -1.618242
 H     -0.944738  -2.612273  -0.694137
 C      0.678695  -0.286730  -2.530596
 H      1.033017  -0.810726  -3.446154
 H     -0.272149   0.217947  -2.814739
 C      1.669062   0.780729  -2.145438
 C      2.762240   1.990146  -0.458910
 C      3.278694   2.882015  -1.431224
 H      3.946481   3.694145  -1.114932
 C      3.000591   2.689492  -2.786349
 C      2.200919   1.601036  -3.154173
 H      1.955927   1.394193  -4.206373
 C     -0.746334  -2.989852  -2.797076
 H     -1.614298  -3.678981  -2.806066
 H     -0.714128  -2.474985  -3.779069
 H      0.170662  -3.601931  -2.684800
 C      1.152250   0.388906   2.006509
 C      0.033728  -1.631356   1.337824
 H      1.189884  -1.940714  -1.423386
 H      2.125068  -1.374742   0.439769
 C      2.961346  -2.671606  -0.178253
 C      4.335379  -2.061479  -0.074347
 C      2.828356  -3.559590   1.086782
 C      4.872338  -2.293038   1.211746
 C      5.084773  -1.428667  -1.079962
 C      3.854094  -2.994833   2.093695
 C      6.183446  -1.882124   1.504779
 C      6.396772  -1.017616  -0.783200
 H      4.648041  -1.278922  -2.080596
 C      6.940195  -1.243933   0.500456
 H      6.622327  -2.062875   2.499966
 H      7.009922  -0.526207  -1.555808
 H      7.972616  -0.924999   0.717141
 O      2.410802  -2.948066  -1.288929
 H      4.311032  -3.767816   2.745773
 H      3.371239  -2.252942   2.770502
 H      1.793023  -3.627737   1.466995
 H      3.133906  -4.580206   0.760228
 H      3.425019   3.364423  -3.545799
 N      3.103514   2.149746   0.889772
 C      3.468498   3.486099   1.343201
 H      2.767794   4.239718   0.934893
 H      3.396716   3.510792   2.450283
 H      4.512903   3.782063   1.071651
 C      3.950822   1.107912   1.478132
 H      3.632999   0.116746   1.111033
 H      5.024398   1.266313   1.213447
 H      3.847451   1.120251   2.581012
---------------------------------------
Mn9/TS-iii
Frequencies, energies and thermodynamic properties:
 Lowest Vibrational Mode (1/cm) =-572.0868
 2nd Lowest Vibrational Mode (1/cm) =                     20.2039
 E(RB-P86) (a.u.) =                                     -4386.42188423
 Thermal correction to Enthalpy (a.u.) =                 0.631455
 Thermal correction to Gibbs Free Energy (a.u.) =        0.519964
 Total Entropy (cal/Kmol) =                              234.654
 E(RPBE1PBE) (a.u.) =                                   -4385.73901825
Optimised cartesian coordinates (Angstrom):
 Fe3.408353  -0.664479   0.379796
 Mn-1.031824  -0.464315  -1.519819
 P      0.021776   0.537518   0.225612
 O     -2.044639   2.095201  -2.608281
 O      1.149727  -0.061808  -3.434241
 N     -0.414409  -2.322155  -0.805716
 N     -2.764736  -1.087271  -0.409154
 C      1.409222  -0.496259   0.847871
 C      1.717395  -1.852836   0.395236
 C      2.725471  -2.373907   1.289318
 H      3.191494  -3.365124   1.219309
 C      3.055681  -1.370063   2.265430
```

```
 H      3.807539  -1.463985   3.060808
 C      2.256555  -0.207505   1.992296
 H      2.277935   0.732916   2.558100
 C      3.865870   0.174221  -1.449591
 H      3.142473   0.417828  -2.237381
 C      4.536897  -1.088354  -1.291563
 H      4.420627  -1.966845  -1.940452
 C      5.369505  -1.007292  -0.118205
 H      5.995867  -1.813628   0.286580
 C      5.213996   0.309444   0.447237
 H      5.702196   0.681232   1.358209
 C      4.282012   1.038766  -0.373307
 H      3.933844   2.065737  -0.202393
 C     -1.034415   0.815070   1.738206
 C     -2.075312   1.769218   1.634715
 H     -2.214031   2.329474   0.695464
 C     -2.927996   2.020068   2.722193
 H     -3.725131   2.774801   2.627993
 C     -2.766036   1.309195   3.926980
 H     -3.435201   1.504920   4.779877
 C     -1.746882   0.347433   4.033213
 H     -1.613193  -0.215715   4.970801
 C     -0.885824   0.100833   2.946318
 H     -0.085945  -0.648253   3.049350
 C      0.778543   2.234760   0.043688
 C      1.120355   2.736694  -1.230976
 H      0.913354   2.143389  -2.131908
 C      1.731112   3.997152  -1.365371
 H      1.987826   4.371955  -2.369020
 C      2.008334   4.776251  -0.228367
 H      2.484594   5.763885  -0.334818
 C      1.665053   4.289248   1.046205
 H      1.870442   4.893941   1.944036
 C      1.050876   3.031900   1.180750
 H      0.771236   2.675155   2.184184
 C      1.023523  -2.598517  -0.755980
 H      1.444923  -2.212679  -1.707581
 C     -1.124200  -2.712141   0.392348
 H     -1.241377  -3.822345   0.494858
 H     -0.586732  -2.404681   1.333468
 C     -2.495421  -2.095488   0.465736
 C     -4.022147  -0.527633  -0.394882
 C     -4.959052  -0.873085   0.608228
 C     -4.646760  -1.867370   1.541533
 C     -3.411136  -2.517330   1.446446
 C      1.337732  -4.110541  -0.718202
 H      2.423558  -4.297505  -0.849541
 H      1.030420  -4.581796   0.238644
 H      0.800337  -4.620437  -1.543438
 C     -1.712519   1.055799  -2.155607
 C      0.322358  -0.211175  -2.597633
 H     -1.245698  -2.014027  -2.149064
 H     -1.670902  -1.472611  -2.752910
 H     -5.951178  -0.402456   0.610183
 H     -3.133076  -3.331133   2.132341
 H     -5.380315  -2.157843   2.309953
 N     -4.355290   0.382977  -1.402909
 C     -5.384202   1.375170  -1.121916
 H     -5.293400   2.195653  -1.863512
 H     -6.425465   0.972803  -1.194534
 H     -5.240141   1.807163  -0.112792
 C     -4.425316  -0.136792  -2.772171
 H     -5.426125  -0.589159  -2.979121
 H     -4.251047   0.683936  -3.497303
 H     -3.647202  -0.904607  -2.928217
---------------------------------------
Mn10/i
Frequencies, energies and thermodynamic properties:
 Lowest Vibrational Mode (1/cm) =                          24.1624
 2nd Lowest Vibrational Mode (1/cm) =                      26.7651
 E(RB-P86) (a.u.) =                                     -4350.56236425
 Thermal correction to Enthalpy (a.u.) =                   0.534949
 Thermal correction to Gibbs Free Energy (a.u.) =          0.429638
 Total Entropy (cal/Kmol) =                                221.646
 E(RPBE1PBE) (a.u.) =                                   -4349.91411983
Optimised cartesian coordinates (Angstrom):
 Fe3.039623  -0.885414   0.666466
 Mn-1.129601  -0.382233  -1.573414
 P     -0.138218   0.665245   0.074301
 O     -2.002222   1.976391  -3.156708
 O      1.125221  -0.304656  -3.445260
 N     -0.752800  -2.114883  -0.894723
 N     -2.980239  -0.808627  -0.695335
 C      1.042550  -0.479955   0.907825
 C      1.237671  -1.883184   0.549880
```

```
 C      2.083338  -2.469233   1.562234
 H      2.439283  -3.507328   1.579030
 C      2.417266  -1.459085   2.531360
 H      3.063531  -1.595122   3.409177
 C      1.786064  -0.231626   2.130186
 H      1.849950   0.725573   2.663516
 C      3.740404  -0.223893  -1.155314
 H      3.118709   0.017226  -2.027282
 C      4.286884  -1.518540  -0.845871
 H      4.162270  -2.431437  -1.443806
 C      5.006429  -1.414356   0.398208
 H      5.522017  -2.233844   0.916721
 C      4.906954  -0.051798   0.856392
 H      5.333833   0.348609   1.785785
 C      4.122336   0.683680  -0.101433
 H      3.845673   1.743828  -0.034008
 C     -1.331784   1.103277   1.432316
 C     -2.175910   2.225213   1.251652
 H     -2.061485   2.860857   0.358871
 C     -3.159804   2.538642   2.203334
 H     -3.801863   3.421441   2.053699
 C     -3.327899   1.727494   3.342584
 H     -4.101127   1.973127   4.087734
 C     -2.504615   0.602701   3.522042
 H     -2.629192  -0.038026   4.409712
 C     -1.511388   0.290823   2.573796
 H     -0.864224  -0.585108   2.734028
 C      0.797015   2.259069  -0.131394
 C      1.243374   2.661177  -1.408800
 H      1.012759   2.048014  -2.291474
 C      1.986620   3.845565  -1.565486
 H      2.326372   4.145744  -2.569482
 C      2.290146   4.644695  -0.449094
 H      2.870137   5.572977  -0.573218
 C      1.840372   4.257405   0.826923
 H      2.065045   4.881886   1.706306
 C      1.095795   3.075720   0.984808
 H      0.733960   2.796671   1.986803
 C      0.630096  -2.579772  -0.678580
 H      1.201838  -2.236833  -1.563464
 C     -1.712720  -2.740624  -0.009271
 H     -1.884252  -3.817784  -0.251424
 H     -1.363589  -2.744164   1.058724
 C     -3.022471  -2.019005  -0.052713
 C     -4.122503  -0.092142  -0.701484
 C     -5.333624  -0.505047  -0.142702
 H     -6.210077   0.154031  -0.210751
 C     -5.369548  -1.759742   0.486665
 C     -4.194775  -2.523530   0.531736
 H     -4.172482  -3.507569   1.022549
 C      0.786758  -4.110536  -0.617575
 H      1.860110  -4.386861  -0.648348
 H      0.354370  -4.552446   0.303376
 H      0.294528  -4.577147  -1.495297
 C     -1.706232   1.044066  -2.492005
 C      0.253358  -0.331087  -2.639362
 H     -6.302631  -2.129145   0.937792
 F     -4.079334   1.104801  -1.296840
----------------------------------------
Mn10/ii
Frequencies, energies and thermodynamic properties:
 Lowest Vibrational Mode (1/cm) =                         14.7951
 2nd Lowest Vibrational Mode (1/cm) =                     20.2804
 E(RB-P86) (a.u.) =                                     -4505.49699605
 Thermal correction to Enthalpy (a.u.) =                  0.619585
 Thermal correction to Gibbs Free Energy (a.u.) =         0.498450
 Total Entropy (cal/Kmol) =                               254.952
 E(RPBE1PBE) (a.u.) =                                   -4504.85749581
Optimised cartesian coordinates (Angstrom):
 Fe-3.032888  -1.186447  -0.949015
 Mn1.194571  -0.283713   1.056839
 P     -0.371360   0.946136   0.140107
 O      1.622552   1.371591   3.483048
 O     -0.573988  -1.763687   2.866643
 N      1.195202  -1.605168  -0.343062
 N      2.858501   0.374231  -0.034250
 C     -1.323495  -0.060565  -1.071898
 C     -1.040719  -1.455635  -1.404457
 C     -1.873483  -1.802818  -2.531078
 H     -1.918668  -2.786626  -3.015417
 C     -2.666416  -0.658449  -2.894369
 H     -3.412340  -0.619455  -3.699741
 C     -2.338534   0.414268  -1.995791
 H     -2.775082   1.421119  -2.012876
 C     -3.512283  -1.637517   1.003824
```

```
 H     -2.825106  -1.583155   1.857955
 C     -3.712338  -2.785025   0.159205
 H     -3.210684  -3.756795   0.261129
 C     -4.666679  -2.428785  -0.859769
 H     -5.016822  -3.079404  -1.672356
 C     -5.059479  -1.059617  -0.641994
 H     -5.761903  -0.484436  -1.260092
 C     -4.344246  -0.568905   0.507591
 H     -4.406133   0.444801   0.924303
 C      0.361690   2.299323  -0.903724
 C      0.900298   3.433296  -0.249442
 H      0.812401   3.530265   0.844629
 C      1.547147   4.439841  -0.984383
 H      1.952854   5.321175  -0.462446
 C      1.679702   4.322869  -2.381812
 H      2.188799   5.112264  -2.957193
 C      1.160168   3.193602  -3.037378
 H      1.259371   3.093726  -4.130084
 C      0.504006   2.186091  -2.304368
 H      0.089663   1.313724  -2.832570
 C     -1.650896   1.875827   1.115963
 C     -1.937046   1.514173   2.450188
 H     -1.382618   0.697365   2.933148
 C     -2.934137   2.192465   3.175071
 H     -3.144145   1.898953   4.215810
 C     -3.655030   3.241573   2.577982
 H     -4.433747   3.773253   3.147496
 C     -3.369841   3.615468   1.251665
 H     -3.922800   4.442288   0.778106
 C     -2.372213   2.941152   0.526830
 H     -2.147005   3.258894  -0.503214
 C     -0.033619  -2.359073  -0.681017
 H     -0.482733  -2.636757   0.293235
 C      2.043385  -1.362236  -1.498840
 H      2.538697  -2.288467  -1.877208
 H      1.451054  -0.978922  -2.372009
 C      3.089070  -0.340105  -1.181419
 C      3.741859   1.349611   0.256437
 C      4.874769   1.658831  -0.499543
 C      5.112513   0.902803  -1.657747
 C      4.205152  -0.109251  -2.000870
 C      0.231183  -3.675585  -1.435014
 H     -0.691414  -4.289104  -1.475474
 H      0.572794  -3.514548  -2.478009
 H      1.004964  -4.252005  -0.888686
 C      1.494884   0.748055   2.487756
 C      0.091689  -1.158043   2.094110
 H      2.124167  -2.855860   0.711339
 O      2.542510  -3.635512   1.179104
 C      3.943444  -3.603402   0.939909
 H      4.385725  -2.617889   1.232792
 H      4.177556  -3.740733  -0.147082
 H      4.348275  -0.721942  -2.902810
 H      5.537044   2.471105  -0.169883
 C      4.616109  -4.713439   1.739919
 H      5.712499  -4.718533   1.569001
 H      4.433823  -4.578918   2.826893
 H      4.214570  -5.706601   1.447437
 H      5.994915   1.106568  -2.282696
 F      3.505448   2.067296   1.358886
---------------------------------------
Mn10/iii
Frequencies, energies and thermodynamic properties:
 Lowest Vibrational Mode (1/cm) =                          20.1304
 2nd Lowest Vibrational Mode (1/cm) =                      27.0282
 E(RB-P86) (a.u.) =                                      -4506.67049912
 Thermal correction to Enthalpy (a.u.) =                   0.635757
 Thermal correction to Gibbs Free Energy (a.u.) =          0.516434
 Total Entropy (cal/Kmol) =                                251.136
 E(RPBE1PBE) (a.u.) =                                    -4506.02777089
Optimised cartesian coordinates (Angstrom):
 Fe-3.046757  -1.228275  -0.894450
 Mn1.227514  -0.243999   1.253699
 P     -0.348431   0.904643   0.082103
 O      1.265282   1.855980   3.340198
 O     -0.664514  -1.672625   2.986328
 N      1.215138  -1.718454  -0.231838
 N      2.826661   0.404421   0.039955
 C     -1.313713  -0.140689  -1.067698
 C     -1.052684  -1.556396  -1.309705
 C     -1.881422  -1.950905  -2.424883
 H     -1.940732  -2.959515  -2.853124
 C     -2.653909  -0.817848  -2.860339
 H     -3.393609  -0.814169  -3.672331
 C     -2.317454   0.299332  -2.021455
```

```
 H     -2.737212   1.310642  -2.097260
 C     -3.567396  -1.565981   1.071924
 H     -2.897706  -1.471719   1.935948
 C     -3.761738  -2.756809   0.288894
 H     -3.270830  -3.725304   0.454023
 C     -4.692874  -2.449270  -0.766749
 H     -5.033022  -3.140664  -1.549371
 C     -5.077258  -1.066900  -0.632324
 H     -5.762828  -0.521286  -1.294599
 C     -4.379518  -0.519181   0.501831
 H     -4.441123   0.515912   0.861976
 C      0.400556   2.210384  -1.013892
 C      1.006882   3.327038  -0.389353
 H      0.984032   3.424815   0.707950
 C      1.635460   4.320300  -1.157753
 H      2.096128   5.186682  -0.656983
 C      1.678225   4.209029  -2.560881
 H      2.171939   4.988119  -3.163029
 C      1.087918   3.098699  -3.187931
 H      1.115379   3.003140  -4.285120
 C      0.452098   2.103708  -2.420290
 H     -0.016443   1.246297  -2.927173
 C     -1.636362   1.906333   0.982960
 C     -2.007962   1.587073   2.306977
 H     -1.512055   0.764390   2.839858
 C     -3.018889   2.313898   2.961874
 H     -3.293225   2.051280   3.995858
 C     -3.672150   3.370765   2.304100
 H     -4.462127   3.940456   2.818668
 C     -3.304410   3.701753   0.987063
 H     -3.804316   4.532792   0.464445
 C     -2.291382   2.979264   0.333136
 H     -2.003494   3.264368  -0.690661
 C     -0.049445  -2.422101  -0.541887
 H     -0.503435  -2.662822   0.441612
 C      1.900914  -1.277279  -1.440141
 H      2.332985  -2.120629  -2.031890
 H      1.214336  -0.751288  -2.160548
 C      2.993503  -0.302153  -1.121206
 C      3.753763   1.332298   0.329678
 C      4.872419   1.626454  -0.456130
 C      5.041993   0.891145  -1.637172
 C      4.092176  -0.085778  -1.969432
 C      0.187270  -3.774060  -1.250875
 H     -0.751980  -4.361995  -1.303185
 H      0.561573  -3.649967  -2.288385
 H      0.929854  -4.361873  -0.674912
 C      1.301444   1.035849   2.494747
 C      0.051063  -1.083213   2.253285
 H      2.549628  -0.803157   2.212933
 H      2.345589  -1.505269   1.814873
 H      2.127147  -2.838262   0.504929
 O      2.664887  -3.513460   1.097115
 C      3.993553  -3.611112   0.617952
 H      4.021139  -3.947057  -0.451214
 H      4.515510  -2.618502   0.640604
 H      4.190781  -0.686041  -2.885340
 H      5.575496   2.403772  -0.127065
 C      4.779479  -4.604366   1.470613
 H      4.301853  -5.606639   1.441486
 H      5.824021  -4.706481   1.108627
 H      4.810540  -4.274425   2.530668
 H      5.909781   1.078320  -2.287630
 F      3.586887   2.016103   1.467941
--------------------------------------
Mn10/iv
Frequencies, energies and thermodynamic properties:
 Lowest Vibrational Mode (1/cm) =                        19.4067
 2nd Lowest Vibrational Mode (1/cm) =                    22.5889
 E(RB-P86) (a.u.) =                                    -4506.69853514
 Thermal correction to Enthalpy (a.u.) =                 0.640378
 Thermal correction to Gibbs Free Energy (a.u.) =        0.520070
 Total Entropy (cal/Kmol) =                              253.211
 E(RPBE1PBE) (a.u.) =                                  -4506.05438462
Optimised cartesian coordinates (Angstrom):
 Fe-3.114748  -1.130334  -0.900037
 Mn1.282932  -0.362770   1.064377
 P     -0.360772   0.915441   0.139183
 O      1.584312   1.344378   3.439422
 O     -0.382515  -2.058893   2.785653
 N      1.163740  -1.703311  -0.621526
 N      2.819085   0.374738  -0.156328
 C     -1.411473   0.002412  -1.066100
 C     -1.166112  -1.374732  -1.495884
 C     -2.071026  -1.649365  -2.589524
```

```
 H     -2.157850  -2.600250  -3.129960
 C     -2.875890  -0.482810  -2.829962
 H     -3.673799  -0.395210  -3.579533
 C     -2.480454   0.531727  -1.893214
 H     -2.910239   1.538804  -1.817327
 C     -3.461684  -1.719199   1.043185
 H     -2.713757  -1.734623   1.846737
 C     -3.738444  -2.799159   0.133031
 H     -3.246373  -3.781088   0.127406
 C     -4.757947  -2.359513  -0.784999
 H     -5.174051  -2.944404  -1.616213
 C     -5.114531  -1.007269  -0.439054
 H     -5.850581  -0.381078  -0.960921
 C     -4.312045  -0.609731   0.688252
 H     -4.328264   0.370967   1.180810
 C      0.285052   2.347409  -0.867535
 C      0.968511   3.373546  -0.169917
 H      1.039134   3.334879   0.929423
 C      1.558183   4.442663  -0.863793
 H      2.076914   5.236812  -0.303135
 C      1.491824   4.498071  -2.269702
 H      1.956870   5.335234  -2.814086
 C      0.831464   3.476115  -2.972407
 H      0.775140   3.508163  -4.072404
 C      0.230931   2.407930  -2.277277
 H     -0.294507   1.624120  -2.844629
 C     -1.597664   1.791259   1.226053
 C     -1.852606   1.315649   2.530657
 H     -1.303033   0.444092   2.913796
 C     -2.809704   1.944820   3.347848
 H     -2.995117   1.560468   4.363560
 C     -3.522425   3.060851   2.874483
 H     -4.269423   3.554871   3.516039
 C     -3.269080   3.548430   1.579030
 H     -3.816312   4.427043   1.201382
 C     -2.311092   2.921533   0.762903
 H     -2.110965   3.326362  -0.241614
 C     -0.158753  -2.356823  -0.911258
 H     -0.524031  -2.677222   0.084511
 C      1.795154  -1.090101  -1.805899
 H      2.141691  -1.839313  -2.550140
 H      1.033405  -0.461661  -2.320339
 C      2.935717  -0.200373  -1.395904
 C      3.785485   1.241568   0.190008
 C      4.891830   1.580535  -0.598536
 C      5.010703   0.968064  -1.851564
 C      4.014470   0.061372  -2.251454
 C      0.006918  -3.622531  -1.769344
 H     -0.947196  -4.184039  -1.817619
 H      0.318177  -3.394981  -2.809352
 H      0.763704  -4.291285  -1.311248
 C      1.494497   0.689021   2.457751
 C      0.245318  -1.356392   2.062497
 H      2.472897  -1.274843   1.634727
 H      2.669283  -2.698311   1.352266
 H      1.789370  -2.466299  -0.283041
 O      2.804231  -3.579508   0.880848
 C      4.211213  -3.760998   0.694240
 H      4.328414  -4.569326  -0.060118
 H      4.678008  -2.845492   0.257106
 H      4.063871  -0.443007  -3.227344
 H      5.626316   2.300807  -0.212410
 C      4.927723  -4.145696   1.988345
 H      4.483377  -5.064400   2.424906
 H      6.006011  -4.335735   1.801806
 H      4.851724  -3.334704   2.743529
 H      5.866487   1.193296  -2.505269
 F      3.680454   1.814463   1.394484
---------------------------------------
Mn10/v
Frequencies, energies and thermodynamic properties:
 Lowest Vibrational Mode (1/cm) =                         18.8247
 2nd Lowest Vibrational Mode (1/cm) =                     31.0468
 E(RB-P86) (a.u.) =                                     -4351.75993026
 Thermal correction to Enthalpy (a.u.) =                  0.555797
 Thermal correction to Gibbs Free Energy (a.u.) =         0.449631
 Total Entropy (cal/Kmol) =                               223.445
 E(RPBE1PBE) (a.u.) =                                   -4351.11183917
Optimised cartesian coordinates (Angstrom):
 Fe3.064095  -0.820963   0.658264
 Mn-1.194106  -0.376098  -1.655308
 P     -0.163308   0.639039   0.100207
 O     -1.956337   2.106127  -3.033364
 O      1.026610  -0.469609  -3.569542
 N     -0.716886  -2.301169  -0.813010
```

```
 N     -2.963717  -0.823084  -0.638543
 C      1.066743  -0.443725   0.944899
 C      1.291433  -1.852986   0.617933
 C      2.172355  -2.392922   1.629788
 H      2.553921  -3.420717   1.670211
 C      2.506534  -1.348427   2.558792
 H      3.180394  -1.445680   3.420542
 C      1.836247  -0.149305   2.139778
 H      1.892745   0.826172   2.639796
 C      3.700908  -0.199864  -1.199202
 H      3.049865   0.003536  -2.059503
 C      4.280937  -1.475054  -0.868805
 H      4.158100  -2.407190  -1.436721
 C      5.033797  -1.321160   0.349769
 H      5.578587  -2.115895   0.876819
 C      4.922132   0.052011   0.770784
 H      5.367095   0.486935   1.675808
 C      4.096644   0.744855  -0.183922
 H      3.801562   1.801012  -0.138655
 C     -1.316202   1.117747   1.487833
 C     -2.276975   2.120885   1.209053
 H     -2.270582   2.625927   0.229235
 C     -3.239435   2.479511   2.166705
 H     -3.971624   3.269696   1.934907
 C     -3.273364   1.829000   3.415694
 H     -4.030370   2.107885   4.165819
 C     -2.337360   0.819038   3.695415
 H     -2.356166   0.301447   4.668085
 C     -1.364551   0.465581   2.739453
 H     -0.630779  -0.318495   2.982628
 C      0.761130   2.240746  -0.144011
 C      1.236972   2.584853  -1.427850
 H      1.038534   1.918917  -2.279989
 C      1.966707   3.771177  -1.627094
 H      2.330385   4.024050  -2.635904
 C      2.226724   4.633106  -0.546611
 H      2.795568   5.563424  -0.703587
 C      1.747663   4.304484   0.735129
 H      1.938894   4.977154   1.586644
 C      1.016791   3.119804   0.934127
 H      0.632832   2.885315   1.939478
 C      0.729729  -2.630845  -0.566191
 H      1.241866  -2.285853  -1.486187
 C     -1.605524  -2.595355   0.328789
 H     -1.713159  -3.683173   0.527683
 H     -1.145217  -2.147801   1.238267
 C     -2.955776  -1.964791   0.123360
 C     -4.148344  -0.197836  -0.752069
 C     -5.352838  -0.629336  -0.183069
 H     -6.265118  -0.039196  -0.347319
 C     -5.334072  -1.810052   0.568529
 C     -4.111017  -2.486180   0.719732
 H     -4.044408  -3.415562   1.303820
 C      0.962694  -4.145774  -0.444551
 H      2.047993  -4.369035  -0.448699
 H      0.533956  -4.569631   0.486245
 H      0.515698  -4.675563  -1.310959
 C     -1.688928   1.113738  -2.443522
 C      0.165589  -0.415777  -2.752065
 H     -1.016741  -2.875347  -1.612737
 H     -1.874762  -1.225921  -2.811907
 H     -6.255794  -2.196179   1.028479
 F     -4.172166   0.924042  -1.481451
---------------------------------------
Mn10/vi_R
Frequencies, energies and thermodynamic properties:
 Lowest Vibrational Mode (1/cm) =                         10.9307
 2nd Lowest Vibrational Mode (1/cm) =                     20.2058
 E(RB-P86) (a.u.) =                                     -4774.47218674
 Thermal correction to Enthalpy (a.u.) =                  0.708329
 Thermal correction to Gibbs Free Energy (a.u.) =         0.579652
 Total Entropy (cal/Kmol) =                               270.822
 E(RPBE1PBE) (a.u.) =                                   -4773.81888758
Optimised cartesian coordinates (Angstrom):
 Fe-3.221873  -1.830161  -1.041731
 Mn0.894550   0.225885   0.286991
 P     -1.241500   0.712665   0.512227
 O      1.615883   0.754185   3.113757
 O      0.665098  -2.544569   1.226079
 N      0.726464  -0.211804  -1.620355
 N      1.536381   2.038887  -0.546510
 C     -2.225304  -0.049045  -0.839727
 C     -1.664364  -0.825869  -1.943014
 C     -2.733941  -1.060439  -2.884231
 H     -2.653274  -1.631895  -3.817594
```

```
 C     -3.938712  -0.456195  -2.381477
 H     -4.925149  -0.485737  -2.863654
 C     -3.634964   0.160688  -1.119689
 H     -4.345117   0.704607  -0.483798
 C     -2.633430  -3.205235   0.376462
 H     -1.713072  -3.154742   0.972029
 C     -2.767223  -3.835657  -0.909897
 H     -1.970438  -4.352553  -1.461489
 C     -4.123070  -3.648575  -1.359911
 H     -4.539494  -3.993267  -2.315919
 C     -4.828809  -2.904077  -0.348013
 H     -5.877822  -2.582713  -0.398331
 C     -3.908146  -2.627101   0.724091
 H     -4.129802  -2.059953   1.637497
 C     -1.545344   2.535612   0.305377
 C     -1.172317   3.396155   1.365829
 H     -0.776661   2.973689   2.303443
 C     -1.303447   4.787879   1.233716
 H     -1.017666   5.444581   2.070825
 C     -1.795527   5.342396   0.036119
 H     -1.896895   6.434373  -0.067063
 C     -2.155472   4.495697  -1.026082
 H     -2.541369   4.920910  -1.966306
 C     -2.032189   3.099028  -0.894387
 H     -2.331679   2.447430  -1.729473
 C     -2.197821   0.356321   2.066894
 C     -1.741938  -0.610031   2.989276
 H     -0.796112  -1.140760   2.814031
 C     -2.492062  -0.906619   4.142093
 H     -2.120528  -1.662203   4.852293
 C     -3.705228  -0.240658   4.389479
 H     -4.290429  -0.472498   5.293459
 C     -4.163118   0.731133   3.480601
 H     -5.107731   1.265428   3.669949
 C     -3.413341   1.031449   2.330420
 H     -3.775128   1.809355   1.640162
 C     -0.204336  -1.276621  -2.067884
 H     -0.050802  -2.112687  -1.356838
 C      0.769770   0.915486  -2.545629
 H      1.292846   0.671122  -3.500532
 H     -0.260520   1.236117  -2.851855
 C      1.438598   2.095466  -1.912600
 C      2.076819   3.110421   0.065658
 C      2.559740   4.248910  -0.584159
 H      2.988179   5.067604   0.009984
 C      2.473009   4.285204  -1.983993
 C      1.906040   3.192307  -2.654090
 H      1.815257   3.180749  -3.749908
 C      0.112707  -1.826950  -3.471946
 H     -0.493425  -2.732873  -3.674661
 H     -0.100545  -1.095058  -4.277748
 H      1.180794  -2.117922  -3.533309
 C      1.347856   0.601986   1.973730
 C      0.701516  -1.428131   0.829719
 H      2.236616  -0.832028  -1.817200
 H      2.987466  -0.367838   0.040494
 C      3.806026  -0.761315  -0.668917
 C      4.515835  -1.882029   0.078419
 C      4.909524   0.330958  -0.763092
 C      5.665319  -1.387438   0.733562
 C      4.163686  -3.237545   0.161893
 C      5.819545   0.101121   0.468097
 H      5.474076   0.130595  -1.700198
 C      6.471547  -2.252639   1.492334
 C      4.974211  -4.105419   0.920370
 H      3.271546  -3.611031  -0.365689
 H      6.874043   0.408068   0.305403
 C      6.118870  -3.615133   1.582177
 H      7.374570  -1.878298   2.002760
 H      4.717218  -5.174735   0.993229
 H      6.748226  -4.304839   2.167786
 O      3.223379  -1.125316  -1.881849
 H      4.498501   1.358216  -0.831709
 H      5.452439   0.685050   1.343504
 H      2.844240   5.158469  -2.541023
 F      2.156358   3.066377   1.399220
---------------------------------------
Mn10/vi_S
Frequencies, energies and thermodynamic properties:
 Lowest Vibrational Mode (1/cm) =                          16.6281
 2nd Lowest Vibrational Mode (1/cm) =                      19.3097
 E(RB-P86) (a.u.) =                                       -4774.47262837
 Thermal correction to Enthalpy (a.u.) =                   0.708283
 Thermal correction to Gibbs Free Energy (a.u.) =          0.580608
 Total Entropy (cal/Kmol) =                                268.716
```

E(RPBE1PBE) (a.u.) = -4773.81968364
Optimised cartesian coordinates (Angstrom):

```
Fe-3.680707  -1.360584  -0.908862
Mn0.758019  -0.189171   0.468553
P     -1.175332   0.862025   0.360742
O      1.418901   0.701967   3.217405
O     -0.266573  -2.557688   1.863053
N      0.585187  -0.941569  -1.342808
N      1.923579   1.187827  -0.602190
C     -2.253497   0.112970  -0.924291
C     -1.861021  -0.987453  -1.801941
C     -2.898977  -1.129113  -2.795532
H     -2.922804  -1.879509  -3.595874
C     -3.922815  -0.150483  -2.543537
H     -4.851754  -0.026371  -3.116356
C     -3.536561   0.611462  -1.388050
H     -4.109220   1.434490  -0.941465
C     -3.565224  -2.542048   0.777298
H     -2.697201  -2.623039   1.444064
C     -3.807202  -3.350449  -0.387983
H     -3.159801  -4.155437  -0.761076
C     -5.034770  -2.902735  -0.994962
H     -5.484241  -3.302757  -1.913721
C     -5.553569  -1.817906  -0.201117
H     -6.468395  -1.246957  -0.409308
C     -4.644420  -1.592270   0.892495
H     -4.744130  -0.822798   1.669031
C     -0.959446   2.617915  -0.212915
C     -0.410041   3.551177   0.699028
H     -0.184200   3.242573   1.732446
C     -0.150162   4.871328   0.297614
H      0.268982   5.588898   1.020844
C     -0.421085   5.276098  -1.024046
H     -0.215482   6.311570  -1.338483
C     -0.953848   4.352273  -1.939021
H     -1.168846   4.660218  -2.974723
C     -1.223029   3.029465  -1.537666
H     -1.654125   2.320779  -2.261149
C     -2.276848   1.092908   1.841175
C     -2.161358   0.247720   2.965865
H     -1.393360  -0.537453   2.992676
C     -3.027176   0.398977   4.064497
H     -2.921769  -0.267800   4.934932
C     -4.017457   1.396924   4.054822
H     -4.693263   1.515515   4.916579
C     -4.134385   2.250034   2.941957
H     -4.901137   3.040939   2.928259
C     -3.267854   2.103040   1.845168
H     -3.359316   2.791595   0.990748
C     -0.570216  -1.806036  -1.685660
H     -0.685287  -2.491090  -0.822184
C      0.961321  -0.054277  -2.439148
H      1.427260  -0.596087  -3.295882
H      0.066241   0.463391  -2.874289
C      1.904475   1.004499  -1.960178
C      2.717057   2.170833  -0.135643
C      3.522101   2.996722  -0.924213
H      4.137109   3.769054  -0.442494
C      3.505283   2.788980  -2.311285
C      2.687387   1.776828  -2.832986
H      2.642025   1.581065  -3.914078
C     -0.332664  -2.692908  -2.923340
H     -1.145970  -3.439858  -3.022658
H     -0.295408  -2.111775  -3.867492
H      0.620858  -3.248072  -2.813379
C      1.191779   0.394501   2.100001
C      0.088997  -1.590101   1.276461
H      1.906616  -1.902998  -1.290444
H      2.617750  -1.263022   0.534217
C      3.293538  -2.071305   0.060496
C      3.337832  -3.261466   1.061071
C      4.422266  -2.877647   2.097642
O      2.827395  -2.368318  -1.216891
H      4.125564   3.411263  -2.973710
C      4.713992  -1.530810   0.126767
C      5.378864  -0.701423  -0.789010
C      5.358505  -1.985562   1.298886
C      6.707546  -0.317365  -0.518449
H      4.869474  -0.373907  -1.709836
C      6.681959  -1.599352   1.569490
C      7.352550  -0.761518   0.654172
H      7.249525   0.327233  -1.229389
H      7.197490  -1.953683   2.477643
H      8.393858  -0.459502   0.852037
H      2.348516  -3.486643   1.505946
```

```
 H      3.974181  -2.302182   2.940427
 H      3.662433  -4.152914   0.480322
 H      4.934685  -3.751786   2.551312
 F      2.726769   2.360565   1.187203
---------------------------------------
Mn10/viii
Frequencies, energies and thermodynamic properties:
 Lowest Vibrational Mode (1/cm) =                          19.4491
 2nd Lowest Vibrational Mode (1/cm) =                      35.2686
 E(RB-P86) (a.u.) =                                       -4351.72666977
 Thermal correction to Enthalpy (a.u.) =                   0.550962
 Thermal correction to Gibbs Free Energy (a.u.) =          0.444694
 Total Entropy (cal/Kmol) =                                223.660
 E(RPBE1PBE) (a.u.) =                                     -4351.07909889
Optimised cartesian coordinates (Angstrom):
 Fe3.070982  -0.820388   0.673358
 Mn-1.165279  -0.371715  -1.710325
 P     -0.161469   0.584316   0.076018
 O     -1.893450   2.265901  -2.852940
 O      1.181886  -0.248159  -3.468459
 N     -0.693143  -2.213103  -0.932551
 N     -2.945869  -0.817466  -0.664933
 C      1.054091  -0.506110   0.906745
 C      1.310453  -1.891104   0.525890
 C      2.172204  -2.452820   1.539025
 H      2.571882  -3.475084   1.544689
 C      2.460350  -1.442718   2.523512
 H      3.107064  -1.562864   3.403367
 C      1.782056  -0.236392   2.134736
 H      1.802985   0.716368   2.679309
 C      3.763847  -0.082316  -1.123002
 H      3.141886   0.177580  -1.988858
 C      4.332590  -1.376677  -0.857927
 H      4.224719  -2.270281  -1.487325
 C      5.046318  -1.303023   0.391924
 H      5.575306  -2.130795   0.883136
 C      4.921123   0.041031   0.897082
 H      5.339191   0.416423   1.840853
 C      4.125804   0.795269  -0.036991
 H      3.831459   1.847864   0.064998
 C     -1.377839   1.000448   1.422990
 C     -2.279495   2.068115   1.196630
 H     -2.212967   2.657418   0.267954
 C     -3.259277   2.389091   2.150268
 H     -3.948430   3.227974   1.962885
 C     -3.361747   1.641755   3.339409
 H     -4.130690   1.893656   4.086880
 C     -2.478055   0.572944   3.566504
 H     -2.550346  -0.017395   4.493942
 C     -1.490853   0.252590   2.614507
 H     -0.798676  -0.580632   2.810363
 C      0.728422   2.214027  -0.097870
 C      1.230048   2.635373  -1.348343
 H      1.068640   2.022216  -2.245424
 C      1.944595   3.841971  -1.463892
 H      2.326227   4.154829  -2.448766
 C      2.166994   4.645912  -0.332339
 H      2.724710   5.591401  -0.424403
 C      1.664728   4.239183   0.917486
 H      1.826170   4.864933   1.809750
 C      0.947254   3.036061   1.033128
 H      0.544590   2.743193   2.015180
 C      0.703295  -2.583660  -0.703792
 H      1.267370  -2.224024  -1.589683
 C     -1.568787  -2.650738   0.126650
 H     -1.735571  -3.759480   0.140318
 H     -1.180783  -2.427119   1.166884
 C     -2.908678  -1.983588   0.052357
 C     -4.118023  -0.160772  -0.703561
 C     -5.297058  -0.578556  -0.080665
 C     -5.254440  -1.780869   0.641467
 C     -4.046810  -2.489238   0.704315
 C      0.922096  -4.112494  -0.647639
 H      2.003424  -4.361456  -0.665934
 H      0.490379  -4.570178   0.266885
 H      0.445288  -4.586388  -1.529833
 C     -1.662262   1.204469  -2.391973
 C      0.271861  -0.287702  -2.712873
 H     -1.742847  -1.642861  -2.786965
 H     -2.126793  -0.988759  -3.095574
 H     -6.206113   0.030502  -0.178334
 H     -3.971078  -3.436082   1.258633
 H     -6.157021  -2.157495   1.146118
 F     -4.147193   0.980002  -1.404386
---------------------------------------
```

```
Mn10/ix
Frequencies, energies and thermodynamic properties:
 Lowest Vibrational Mode (1/cm) =                         24.5299
 2nd Lowest Vibrational Mode (1/cm) =                     33.5095
 E(RB-P86) (a.u.) =                                     -4505.51467445
 Thermal correction to Enthalpy (a.u.) =                  0.620319
 Thermal correction to Gibbs Free Energy (a.u.) =         0.505420
 Total Entropy (cal/Kmol) =                               241.825
 E(RPBE1PBE) (a.u.) =                                   -4504.87072193
Optimised cartesian coordinates (Angstrom):
 Fe-3.278551  -0.635755  -0.825811
 Mn1.256248  -0.790962   0.949209
 P     -0.110454   0.786598   0.083231
 O      2.024795   0.904363   3.228369
 O     -0.747195  -1.928347   2.760248
 N      0.769014  -2.081353  -0.684810
 N      2.833330  -0.342531  -0.422654
 C     -1.369570   0.083097  -1.068425
 C     -1.453444  -1.320232  -1.474679
 C     -2.434732  -1.403496  -2.532085
 H     -2.749461  -2.320277  -3.046284
 C     -2.966098  -0.090333  -2.775960
 H     -3.748964   0.162868  -3.503429
 C     -2.322081   0.824631  -1.875296
 H     -2.513328   1.903469  -1.813277
 C     -3.691758  -1.078503   1.144046
 H     -2.944139  -1.229305   1.933404
 C     -4.215686  -2.100939   0.277362
 H     -3.943825  -3.164977   0.295854
 C     -5.141684  -1.481007  -0.635715
 H     -5.694416  -1.987739  -1.438133
 C     -5.192131  -0.073831  -0.330515
 H     -5.790682   0.679489  -0.860081
 C     -4.294511   0.176102   0.767190
 H     -4.088356   1.151922   1.225655
 C      0.772094   2.033938  -0.988116
 C      1.610639   2.976690  -0.345489
 H      1.668058   2.999860   0.754723
 C      2.371776   3.886832  -1.096716
 H      3.012019   4.619449  -0.579650
 C      2.320354   3.862148  -2.504046
 H      2.919208   4.574864  -3.092981
 C      1.500714   2.921968  -3.151192
 H      1.452610   2.894199  -4.251633
 C      0.730179   2.013872  -2.399114
 H      0.081741   1.294624  -2.922803
 C     -1.099665   1.923374   1.182579
 C     -1.380345   1.561716   2.518151
 H     -0.994645   0.618120   2.927466
 C     -2.158026   2.398985   3.339215
 H     -2.365103   2.099833   4.379045
 C     -2.663888   3.611719   2.839204
 H     -3.270633   4.267630   3.483555
 C     -2.381791   3.986784   1.512641
 H     -2.765506   4.939023   1.112764
 C     -1.602351   3.152231   0.692941
 H     -1.373857   3.471166  -0.335921
 C     -0.647367  -2.484607  -0.908307
 H     -1.030184  -2.713861   0.106546
 C      1.514559  -1.758027  -1.909388
 H      1.720350  -2.651117  -2.538809
 H      0.891664  -1.074704  -2.531482
 C      2.805456  -1.050743  -1.589721
 C      3.942965   0.360399  -0.165273
 C      5.072369   0.418485  -0.991210
 C      5.040885  -0.323232  -2.178437
 C      3.890723  -1.069583  -2.479806
 C     -0.778084  -3.765490  -1.749990
 H     -1.825764  -4.126973  -1.740391
 H     -0.484679  -3.610988  -2.808340
 H     -0.143832  -4.569109  -1.323320
 C      1.763461   0.227907   2.293312
 C      0.028916  -1.443484   2.004941
 H      1.300985  -2.800298  -0.128235
 H      3.826118  -1.664655  -3.402270
 H      5.936228   1.025465  -0.687081
 H      5.905046  -0.320093  -2.859734
 O      2.428480  -2.428270   1.189108
 C      2.705365  -3.059552   2.403562
 C      3.843546  -2.406304   3.205230
 H      3.003513  -4.122582   2.194545
 H      1.803698  -3.136681   3.070960
 H      4.066396  -2.983120   4.130101
 H      4.772600  -2.360451   2.596710
 H      3.580049  -1.370222   3.502597
```

F   3.965580   1.058447   0.976379

Mn10/x

Frequencies, energies and thermodynamic properties:

Lowest Vibrational Mode (1/cm) =	15.6142
2nd Lowest Vibrational Mode (1/cm) =	23.4254
E(RB-P86) (a.u.) =	-4660.45688290
Thermal correction to Enthalpy (a.u.) =	0.704619
Thermal correction to Gibbs Free Energy (a.u.) =	0.576430
Total Entropy (cal/Kmol) =	269.795
E(RPBE1PBE) (a.u.) =	-4659.82072775

Optimised cartesian coordinates (Angstrom):

```
Fe-3.257171  -1.416783  -0.761641
Mn1.161707  -0.095849   0.893534
P     -0.681376   0.927867   0.064923
O      1.292416   1.877322   3.072466
O     -0.259160  -1.842490   2.770674
N      1.068665  -1.530659  -0.716745
N      2.456894   0.802988  -0.540960
C     -1.685977  -0.131299  -1.054760
C     -1.326024  -1.490638  -1.453378
C     -2.251857  -1.893865  -2.487139
H     -2.268842  -2.868498  -2.990614
C     -3.179909  -0.821315  -2.721474
H     -4.018587  -0.839112  -3.430302
C     -2.842600   0.261319  -1.839634
H     -3.365602   1.223983  -1.775618
C     -3.455678  -1.945019   1.219264
H     -2.678963  -1.839995   1.987740
C     -3.649219  -3.091946   0.371973
H     -3.052704  -4.014044   0.387171
C     -4.747969  -2.810414  -0.516148
H     -5.130881  -3.476706  -1.300712
C     -5.236689  -1.489262  -0.214184
H     -6.057896  -0.972547  -0.728758
C     -4.436963  -0.952502   0.855990
H     -4.540742   0.043740   1.304914
C     -0.270912   2.395824  -1.010915
C      0.236430   3.557430  -0.380098
H      0.309049   3.598776   0.718511
C      0.646030   4.664157  -1.141473
H      1.030546   5.562886  -0.633228
C      0.568411   4.625677  -2.547085
H      0.891461   5.493382  -3.143840
C      0.075415   3.473497  -3.182760
H      0.008327   3.433354  -4.281772
C     -0.342895   2.365431  -2.420676
H     -0.741982   1.477943  -2.935654
C     -1.945100   1.684444   1.209993
C     -2.066999   1.238373   2.543876
H     -1.394971   0.460290   2.930919
C     -3.050210   1.778460   3.393232
H     -3.128504   1.418152   4.431279
C     -3.924512   2.774033   2.923224
H     -4.692506   3.197739   3.589663
C     -3.806299   3.231968   1.598034
H     -4.480781   4.017323   1.221083
C     -2.821772   2.695537   0.750164
H     -2.730906   3.079722  -0.277695
C     -0.190650  -2.332690  -0.891242
H     -0.461050  -2.638138   0.139081
C      1.573635  -0.951898  -1.976981
H      1.989256  -1.718832  -2.665255
H      0.722975  -0.475512  -2.515658
C      2.603732   0.111345  -1.709455
C      3.301904   1.818688  -0.327421
C      4.328986   2.211749  -1.194576
C      4.486335   1.482452  -2.379029
C      3.609978   0.416260  -2.638852
C      0.056130  -3.619298  -1.697745
H     -0.835125  -4.276692  -1.655826
H      0.275655  -3.416538  -2.766047
H      0.909319  -4.172295  -1.256188
C      1.277771   1.106010   2.177289
C      0.268685  -1.120088   1.994066
H      2.994862  -2.446808   0.868266
H      1.786610  -2.226359  -0.387704
O      2.969366  -3.367549   0.347674
C      4.202898  -3.530415  -0.338099
H      4.016770  -4.140708  -1.253093
H      4.598538  -2.545756  -0.691719
H      3.696693  -0.178940  -3.559325
H      4.971442   3.061146  -0.924632
C      5.263233  -4.226277   0.520203
H      4.895125  -5.214265   0.869041
```

```
 H      6.201093  -4.389298  -0.053258
 H      5.512343  -3.620177   1.417244
 H      5.283412   1.743172  -3.091483
 O      2.905200  -1.087693   1.366523
 C      3.481214  -1.042185   2.652443
 C      4.572272   0.027684   2.786952
 H      3.937270  -2.041335   2.881743
 H      2.712540  -0.877345   3.449566
 H      5.028012   0.007529   3.801266
 H      5.380268  -0.142031   2.043364
 H      4.157833   1.041457   2.614286
 F      3.140958   2.512837   0.806385
---------------------------------------
Mn10/TS-i
Frequencies, energies and thermodynamic properties:
 Lowest Vibrational Mode (1/cm) =-761.3870
 2nd Lowest Vibrational Mode (1/cm) =                     20.1489
 E(RB-P86) (a.u.) =                                     -4506.66818174
 Thermal correction to Enthalpy (a.u.) =                 0.631934
 Thermal correction to Gibbs Free Energy (a.u.) =        0.513966
 Total Entropy (cal/Kmol) =                              248.287
 E(RPBE1PBE) (a.u.) =                                   -4506.02266657
Optimised cartesian coordinates (Angstrom):
 Fe-3.083332  -1.145363  -0.893878
 Mn1.236110  -0.310109   1.217219
 P     -0.319695   0.905574   0.088755
 O      1.379412   1.721323   3.358341
 O     -0.669933  -1.715616   2.953975
 N      1.165393  -1.765363  -0.306897
 N      2.842156   0.307177  -0.002452
 C     -1.327475  -0.095939  -1.068381
 C     -1.106892  -1.513588  -1.343779
 C     -1.959183  -1.865435  -2.455459
 H     -2.049544  -2.863302  -2.902886
 C     -2.707331  -0.704540  -2.857357
 H     -3.457526  -0.666211  -3.658651
 C     -2.331208   0.386415  -2.001260
 H     -2.726438   1.409138  -2.051261
 C     -3.587485  -1.508945   1.071950
 H     -2.904193  -1.449488   1.928478
 C     -3.823487  -2.678290   0.268048
 H     -3.356932  -3.662724   0.407685
 C     -4.759775  -2.325365  -0.768513
 H     -5.127755  -2.991572  -1.560304
 C     -5.105677  -0.936538  -0.601666
 H     -5.784518  -0.359751  -1.244108
 C     -4.379174  -0.430236   0.533607
 H     -4.408167   0.598895   0.914298
 C      0.448889   2.207304  -0.998900
 C      1.103401   3.290775  -0.364341
 H      1.102952   3.367336   0.734934
 C      1.752042   4.277299  -1.124618
 H      2.249700   5.118083  -0.615651
 C      1.768421   4.191636  -2.530154
 H      2.278268   4.965193  -3.125946
 C      1.131287   3.113633  -3.167524
 H      1.137910   3.037944  -4.266594
 C      0.474340   2.126204  -2.407803
 H     -0.032146   1.295984  -2.923414
 C     -1.570926   1.925492   1.019670
 C     -1.933627   1.593171   2.342980
 H     -1.452939   0.748123   2.854503
 C     -2.916339   2.335179   3.023366
 H     -3.184601   2.062122   4.056249
 C     -3.549253   3.420405   2.392302
 H     -4.317128   4.001795   2.926861
 C     -3.189448   3.764634   1.076439
 H     -3.673337   4.617859   0.574912
 C     -2.204625   3.026830   0.396981
 H     -1.921565   3.321916  -0.625365
 C     -0.128855  -2.430050  -0.610864
 H     -0.568494  -2.670299   0.378592
 C      1.857328  -1.314086  -1.516314
 H      2.247837  -2.156169  -2.133643
 H      1.165945  -0.749827  -2.193810
 C      2.985789  -0.386247  -1.174699
 C      3.802322   1.196025   0.300777
 C      4.932635   1.459535  -0.480150
 C      5.078313   0.735229  -1.670247
 C      4.092777  -0.200924  -2.017933
 C      0.066838  -3.774183  -1.341598
 H     -0.886783  -4.338211  -1.387464
 H      0.430581  -3.644988  -2.381933
 H      0.800835  -4.389273  -0.783160
 C      1.369810   0.928172   2.486548
```

```
 C      0.053424  -1.134659   2.220936
 H      2.528246  -0.987997   2.099950
 H      2.354405  -1.749778   1.733603
 H      1.912072  -2.645469   0.329193
 O      2.531938  -3.321971   1.109518
 C      3.878535  -3.503624   0.741584
 H      3.977032  -3.761713  -0.346485
 H      4.478788  -2.561446   0.878483
 H      4.171094  -0.791659  -2.941898
 H      5.662939   2.206097  -0.139312
 C      4.528325  -4.615470   1.570044
 H      3.985270  -5.574221   1.428232
 H      5.590126  -4.770771   1.281299
 H      4.497335  -4.367430   2.652534
 H      5.953974   0.898566  -2.316395
 F      3.659955   1.870033   1.447364
--------------------------------------
Mn10/TS-ii_si
Frequencies, energies and thermodynamic properties:
 Lowest Vibrational Mode (1/cm) =-245.8096
 2nd Lowest Vibrational Mode (1/cm) =                     12.1472
 E(RB-P86) (a.u.) =                                     -4774.46715070
 Thermal correction to Enthalpy (a.u.) =                  0.705050
 Thermal correction to Gibbs Free Energy (a.u.) =         0.579671
 Total Entropy (cal/Kmol) =                               263.882
 E(RPBE1PBE) (a.u.) =                                   -4773.80855163
Optimised cartesian coordinates (Angstrom):
 Fe-3.324875  -1.572816  -1.004495
 Mn1.051434   0.096509   0.254058
 P     -1.088811   0.778988   0.515327
 O      1.851616   0.460120   3.063473
 O      0.665860  -2.707263   1.025690
 N      0.722198  -0.268510  -1.796753
 N      1.729638   1.890073  -0.567792
 C     -2.198243   0.130954  -0.803274
 C     -1.749405  -0.656016  -1.952503
 C     -2.877111  -0.786671  -2.847365
 H     -2.884089  -1.335166  -3.797523
 C     -4.008619  -0.113550  -2.270417
 H     -5.016625  -0.062571  -2.703419
 C     -3.600052   0.445454  -1.012008
 H     -4.237929   1.018019  -0.326513
 C     -2.760281  -3.033956   0.334220
 H     -1.803901  -3.071229   0.871566
 C     -3.019071  -3.609373  -0.959225
 H     -2.299053  -4.166076  -1.574093
 C     -4.381318  -3.307719  -1.317865
 H     -4.879350  -3.588058  -2.255712
 C     -4.966099  -2.547564  -0.242776
 H     -5.988473  -2.147133  -0.218295
 C     -3.964392  -2.375520   0.777092
 H     -4.087041  -1.824100   1.718203
 C     -1.303912   2.625670   0.377860
 C     -0.769475   3.423928   1.418676
 H     -0.311398   2.943020   2.298357
 C     -0.818450   4.825367   1.343777
 H     -0.406773   5.430190   2.167610
 C     -1.387377   5.455687   0.219982
 H     -1.423701   6.555030   0.160512
 C     -1.905949   4.672658  -0.825129
 H     -2.352538   5.155405  -1.709200
 C     -1.865867   3.266650  -0.747849
 H     -2.291580   2.669503  -1.568998
 C     -2.018302   0.438503   2.094371
 C     -1.606917  -0.609120   2.946420
 H     -0.720680  -1.207486   2.692567
 C     -2.324522  -0.900110   4.121190
 H     -1.988464  -1.720874   4.774679
 C     -3.460944  -0.145672   4.462637
 H     -4.020894  -0.372339   5.383868
 C     -3.873675   0.906988   3.624855
 H     -4.757870   1.509539   3.887641
 C     -3.155605   1.200016   2.452273
 H     -3.480223   2.039330   1.817432
 C     -0.358995  -1.236287  -2.185126
 H     -0.216787  -2.090162  -1.493729
 C      0.688251   0.987621  -2.570639
 H      0.994335   0.847153  -3.629700
 H     -0.359685   1.364928  -2.590931
 C      1.549180   2.032862  -1.920380
 C      2.421263   2.866867   0.046570
 C      2.975754   3.986871  -0.583122
 H      3.526050   4.723677   0.018010
 C      2.802961   4.107468  -1.967728
 C      2.080103   3.110597  -2.642963
```

```
 H      1.916567   3.163890  -3.728995
 C     -0.168555  -1.769934  -3.615280
 H     -0.860059  -2.614718  -3.805766
 H     -0.358777  -0.997385  -4.388101
 H      0.865752  -2.149060  -3.740574
 C      1.533178   0.372279   1.927428
 C      0.768931  -1.569540   0.705287
 H      1.644701  -0.737732  -2.029232
 H      2.583113  -0.448139  -0.056188
 C      3.771915  -1.054927  -1.089658
 C      4.166161  -2.140403  -0.123319
 C      4.763154   0.102056  -0.812081
 C      5.062587  -1.623109   0.838963
 C      3.807488  -3.497997  -0.138174
 C      5.274918  -0.134254   0.624994
 H      5.590961  -0.033223  -1.546014
 C      5.608615  -2.475026   1.813042
 C      4.358506  -4.348959   0.836497
 H      3.115366  -3.878105  -0.906343
 H      6.328671   0.176560   0.780454
 C      5.250570  -3.838872   1.805039
 H      6.312881  -2.090168   2.569008
 H      4.100008  -5.420128   0.844307
 H      5.678136  -4.518017   2.560369
 O      3.207559  -1.251144  -2.203081
 H      4.326988   1.101834  -0.996186
 H      4.658716   0.440039   1.353645
 H      3.226589   4.965268  -2.510948
 F      2.596068   2.744000   1.367901
---------------------------------------
```

Mn10/TS-ii_re

```
Frequencies, energies and thermodynamic properties:
 Lowest Vibrational Mode (1/cm) =-237.4754
 2nd Lowest Vibrational Mode (1/cm) =                     17.9991
 E(RB-P86) (a.u.) =                                      -4774.46799490
 Thermal correction to Enthalpy (a.u.) =                  0.705194
 Thermal correction to Gibbs Free Energy (a.u.) =         0.580248
 Total Entropy (cal/Kmol) =                               262.970
 E(RPBE1PBE) (a.u.) =                                    -4773.81041600
Optimised cartesian coordinates (Angstrom):
 Fe-3.745974  -1.156553  -0.820330
 Mn0.856068  -0.334693   0.477719
 P     -1.035237   0.896988   0.324079
 O      1.587459   0.535084   3.192883
 O     -0.289502  -2.660370   1.846738
 N      0.532626  -1.126623  -1.450716
 N      2.034144   0.951240  -0.673061
 C     -2.220541   0.214884  -0.908338
 C     -1.943874  -0.926870  -1.781009
 C     -3.025075  -1.005962  -2.736909
 H     -3.133538  -1.762711  -3.523898
 C     -3.964532   0.047340  -2.463903
 H     -4.903381   0.228263  -3.004289
 C     -3.479869   0.795498  -1.337606
 H     -3.974264   1.662848  -0.881577
 C     -3.645436  -2.332339   0.869942
 H     -2.755038  -2.476702   1.495589
 C     -4.004388  -3.126314  -0.275330
 H     -3.440974  -3.982636  -0.669844
 C     -5.220494  -2.587601  -0.828830
 H     -5.742189  -2.956370  -1.722136
 C     -5.615435  -1.461181  -0.022132
 H     -6.491401  -0.821201  -0.193306
 C     -4.641275  -1.300941   1.025764
 H     -4.642612  -0.519994   1.797126
 C     -0.745909   2.635009  -0.285111
 C     -0.047459   3.518209   0.574176
 H      0.242832   3.185510   1.584127
 C      0.280220   4.817261   0.153396
 H      0.814988   5.493969   0.839004
 C     -0.068684   5.252479  -1.139955
 H      0.190646   6.270697  -1.470721
 C     -0.747172   4.378249  -2.005757
 H     -1.023723   4.708056  -3.020069
 C     -1.085022   3.077864  -1.582356
 H     -1.630239   2.412139  -2.269108
 C     -2.087403   1.225164   1.827008
 C     -2.012342   0.367744   2.946028
 H     -1.312634  -0.479903   2.942903
 C     -2.830030   0.585186   4.070189
 H     -2.757453  -0.093347   4.935058
 C     -3.731011   1.664599   4.093285
 H     -4.368679   1.835922   4.975105
 C     -3.806729   2.530356   2.986567
 H     -4.503484   3.383779   2.997875
```

```
 C     -2.988432   2.315159   1.863814
 H     -3.046023   3.012511   1.013341
 C     -0.746781  -1.868282  -1.713421
 H     -0.867139  -2.521224  -0.826685
 C      0.863590  -0.143802  -2.500486
 H      1.163244  -0.623240  -3.457259
 H     -0.045344   0.461504  -2.720279
 C      1.943403   0.789119  -2.031424
 C      2.932995   1.852033  -0.239459
 C      3.768103   2.621658  -1.057592
 H      4.465762   3.333076  -0.594978
 C      3.675299   2.434734  -2.441878
 C      2.751821   1.497088  -2.931919
 H      2.642616   1.316554  -4.011086
 C     -0.638861  -2.785051  -2.944035
 H     -1.516820  -3.458939  -3.000018
 H     -0.587554  -2.218140  -3.895968
 H      0.266280  -3.420128  -2.861034
 C      1.314529   0.240013   2.080132
 C      0.118996  -1.699048   1.279799
 H      1.309826  -1.847689  -1.463939
 H      2.207859  -1.283643   0.471750
 C      3.205313  -2.458379  -0.223746
 C      4.457843  -1.673874   0.060602
 C      3.062241  -3.431563   0.972295
 C      4.871844  -1.897740   1.393396
 C      5.211763  -0.880762  -0.820063
 C      3.879277  -2.791818   2.115964
 C      6.057924  -1.310363   1.862869
 C      6.399808  -0.294891  -0.346471
 H      4.875840  -0.739623  -1.860099
 C      6.816831  -0.508190   0.985572
 H      6.399612  -1.477642   2.897679
 H      7.015168   0.325731  -1.017499
 H      7.752870  -0.048146   1.341572
 O      2.746564  -2.675835  -1.380596
 H      4.371824  -3.531013   2.780920
 H      3.220005  -2.167473   2.761268
 H      2.010069  -3.659260   1.224137
 H      3.541431  -4.381312   0.639536
 H      4.315528   3.009438  -3.127686
 F      3.027163   2.022424   1.083861
---------------------------------------
Mn10/TS-iii
Frequencies, energies and thermodynamic properties:
 Lowest Vibrational Mode (1/cm) =-647.2720
 2nd Lowest Vibrational Mode (1/cm) =                     20.3576
 E(RB-P86) (a.u.) =                                      -4351.72329017
 Thermal correction to Enthalpy (a.u.) =                  0.549957
 Thermal correction to Gibbs Free Energy (a.u.) =         0.444587
 Total Entropy (cal/Kmol) =                               221.771
 E(RPBE1PBE) (a.u.) =                                    -4351.07253798
Optimised cartesian coordinates (Angstrom):
 Fe3.056779  -0.810496   0.674444
 Mn-1.145019  -0.417662  -1.693217
 P     -0.170949   0.619537   0.079870
 O     -1.943275   2.044382  -3.125839
 O      1.209970  -0.297442  -3.438660
 N     -0.711257  -2.292955  -0.870407
 N     -2.937435  -0.822055  -0.647079
 C      1.042071  -0.482908   0.907925
 C      1.295234  -1.880013   0.551820
 C      2.158082  -2.422358   1.575851
 H      2.555409  -3.445003   1.601411
 C      2.451836  -1.395476   2.539314
 H      3.102272  -1.500737   3.418181
 C      1.774892  -0.196585   2.129449
 H      1.801145   0.766825   2.654716
 C      3.753854  -0.093569  -1.129359
 H      3.133332   0.160367  -1.997762
 C      4.315962  -1.388295  -0.851242
 H      4.204788  -2.287238  -1.472418
 C      5.029992  -1.305783   0.397616
 H      5.554739  -2.131162   0.897307
 C      4.911182   0.043745   0.889486
 H      5.330343   0.426101   1.829954
 C      4.119830   0.792649  -0.052173
 H      3.829922   1.847406   0.039861
 C     -1.368733   1.059224   1.436918
 C     -2.285235   2.109222   1.187880
 H     -2.236660   2.667606   0.238986
 C     -3.258044   2.450266   2.141718
 H     -3.958474   3.275327   1.935489
 C     -3.340101   1.739598   3.354707
 H     -4.103882   2.006511   4.102261
```

```
 C     -2.443290   0.686791   3.604326
 H     -2.500052   0.123999   4.549848
 C     -1.463092   0.347078   2.651757
 H     -0.761871  -0.474189   2.865566
 C      0.729732   2.238837  -0.119478
 C      1.205409   2.641478  -1.386246
 H      1.023049   2.014507  -2.269964
 C      1.919565   3.844913  -1.533720
 H      2.281549   4.143333  -2.530450
 C      2.165735   4.664493  -0.418325
 H      2.722850   5.607617  -0.535119
 C      1.687395   4.277232   0.847152
 H      1.867014   4.916282   1.726455
 C      0.970995   3.076972   0.994836
 H      0.586067   2.799309   1.988467
 C      0.695722  -2.633625  -0.645963
 H      1.238218  -2.301170  -1.555253
 C     -1.591095  -2.651172   0.220094
 H     -1.757877  -3.753734   0.312424
 H     -1.185341  -2.341807   1.226340
 C     -2.926641  -1.975426   0.092107
 C     -4.100664  -0.154458  -0.716191
 C     -5.296785  -0.551904  -0.109576
 C     -5.281584  -1.743078   0.628369
 C     -4.081263  -2.462083   0.726549
 C      0.928764  -4.155897  -0.530436
 H      2.011976  -4.395542  -0.537905
 H      0.498657  -4.580584   0.400425
 H      0.459055  -4.668235  -1.394482
 C     -1.676582   1.063847  -2.527403
 C      0.299739  -0.341361  -2.682523
 H     -1.414895  -1.974695  -2.257965
 H     -1.840588  -1.427603  -2.875261
 H     -6.196340   0.066744  -0.231855
 H     -4.026404  -3.400916   1.296431
 H     -6.197694  -2.104240   1.119707
 F     -4.106317   0.977974  -1.429070
---------------------------------------
Mn11/i
Frequencies, energies and thermodynamic properties:
 Lowest Vibrational Mode (1/cm) =                          20.8326
 2nd Lowest Vibrational Mode (1/cm) =                      24.0822
 E(RB-P86) (a.u.) =                                       -4290.67947201
 Thermal correction to Enthalpy (a.u.) =                   0.570446
 Thermal correction to Gibbs Free Energy (a.u.) =          0.464061
 Total Entropy (cal/Kmol) =                                223.904
 E(RPBE1PBE) (a.u.) =                                     -4290.00038929
Optimised cartesian coordinates (Angstrom):
 Fe3.008922  -0.931301   0.676211
 Mn-1.159899  -0.357551  -1.570705
 P     -0.141942   0.684193   0.074241
 O     -1.862458   1.927298  -3.329479
 O      1.113144  -0.329600  -3.420492
 N     -0.794317  -2.092865  -0.910299
 N     -3.052330  -0.769081  -0.683943
 C      1.016260  -0.495206   0.902661
 C      1.193447  -1.898411   0.535378
 C      2.020153  -2.506507   1.550380
 H      2.359323  -3.550283   1.561391
 C      2.359754  -1.510065   2.531311
 H      2.994823  -1.663199   3.414478
 C      1.750206  -0.270487   2.135036
 H      1.822313   0.679238   2.680172
 C      3.733785  -0.268204  -1.135433
 H      3.121755  -0.010752  -2.009589
 C      4.256670  -1.573907  -0.831273
 H      4.121327  -2.480434  -1.436527
 C      4.969006  -1.490207   0.418511
 H      5.467409  -2.321690   0.934744
 C      4.888555  -0.129408   0.885559
 H      5.315577   0.257461   1.820609
 C      4.123291   0.625661  -0.072751
 H      3.863508   1.689702   0.000960
 C     -1.281757   1.158626   1.467152
 C     -1.958717   2.400682   1.434428
 H     -1.737053   3.127687   0.636483
 C     -2.902533   2.728086   2.422799
 H     -3.411312   3.704765   2.388089
 C     -3.195766   1.814444   3.452835
 H     -3.935542   2.071587   4.227436
 C     -2.536366   0.573052   3.487913
 H     -2.756825  -0.147694   4.291558
 C     -1.585957   0.246380   2.503091
 H     -1.065949  -0.722906   2.552510
 C      0.847531   2.242959  -0.156146
```

```
 C      1.241954   2.649937  -1.448908
 H      0.933438   2.067915  -2.328431
 C      2.030894   3.801569  -1.627265
 H      2.328674   4.104390  -2.643705
 C      2.432940   4.564142  -0.516899
 H      3.048906   5.466532  -0.657512
 C      2.035850   4.173126   0.775500
 H      2.337808   4.768887   1.651637
 C      1.247042   3.023917   0.954696
 H      0.930316   2.743623   1.971186
 C      0.582703  -2.577183  -0.700375
 H      1.160938  -2.233726  -1.580533
 C     -1.760133  -2.720110  -0.038927
 H     -1.938149  -3.792181  -0.299473
 H     -1.411885  -2.746735   1.029207
 C     -3.067154  -1.992008  -0.072595
 C     -4.218230  -0.036101  -0.670748
 C     -5.390466  -0.546499  -0.082281
 H     -6.299530   0.072795  -0.097643
 C     -5.404404  -1.817042   0.511543
 C     -4.214421  -2.548948   0.517688
 H     -4.152845  -3.546318   0.978770
 C      0.721397  -4.110091  -0.653763
 H      1.791734  -4.397791  -0.685363
 H      0.282327  -4.556271   0.261847
 H      0.225881  -4.562815  -1.536921
 C     -1.656574   1.038927  -2.571562
 C      0.236923  -0.338612  -2.619232
 H     -6.323305  -2.218942   0.965290
 C     -4.258580   1.332051  -1.288217
 H     -3.405913   1.946385  -0.940879
 H     -4.192575   1.280705  -2.393769
 H     -5.203332   1.844433  -1.024677
---------------------------------------
Mn11/ii
Frequencies, energies and thermodynamic properties:
 Lowest Vibrational Mode (1/cm) =                         15.6588
 2nd Lowest Vibrational Mode (1/cm) =                     21.8319
 E(RB-P86) (a.u.) =                                     -4445.61418996
 Thermal correction to Enthalpy (a.u.) =                  0.655098
 Thermal correction to Gibbs Free Energy (a.u.) =         0.533020
 Total Entropy (cal/Kmol) =                               256.936
 E(RPBE1PBE) (a.u.) =                                   -4444.94403611
Optimised cartesian coordinates (Angstrom):
 Fe-3.012394  -1.211180  -0.964924
 Mn1.211846  -0.259414   1.055406
 P     -0.379511   0.951240   0.144212
 O      1.486382   1.242861   3.595593
 O     -0.549600  -1.791196   2.826128
 N      1.216885  -1.579898  -0.340108
 N      2.920606   0.417690  -0.035960
 C     -1.311615  -0.070416  -1.075726
 C     -1.015285  -1.463271  -1.404209
 C     -1.835886  -1.819441  -2.536822
 H     -1.868502  -2.804340  -3.019975
 C     -2.634800  -0.681955  -2.908302
 H     -3.374320  -0.649777  -3.719870
 C     -2.321820   0.394708  -2.009405
 H     -2.765124   1.398322  -2.034078
 C     -3.499894  -1.665991   0.984777
 H     -2.817754  -1.604369   1.842449
 C     -3.683733  -2.815558   0.139122
 H     -3.173268  -3.782389   0.244173
 C     -4.635306  -2.468605  -0.885676
 H     -4.974301  -3.122663  -1.700240
 C     -5.042427  -1.103213  -0.670528
 H     -5.746731  -0.534937  -1.292872
 C     -4.339206  -0.605622   0.483496
 H     -4.413378   0.407583   0.899462
 C      0.296226   2.330373  -0.905494
 C      0.671650   3.549043  -0.291609
 H      0.474270   3.708815   0.780851
 C      1.278670   4.570239  -1.041422
 H      1.554635   5.516914  -0.550075
 C      1.532307   4.385784  -2.414052
 H      2.009092   5.186481  -3.001330
 C      1.170048   3.175740  -3.031001
 H      1.360207   3.023941  -4.105503
 C      0.555197   2.154059  -2.283303
 H      0.260677   1.218957  -2.784027
 C     -1.686407   1.832410   1.131388
 C     -1.941696   1.468620   2.471236
 H     -1.344287   0.684106   2.955683
 C     -2.962496   2.103967   3.202322
 H     -3.146660   1.808409   4.247359
```

```
 C     -3.739766   3.112140   2.606005
 H     -4.537189   3.610118   3.180068
 C     -3.487252   3.488096   1.273600
 H     -4.084824   4.282828   0.799318
 C     -2.466547   2.856347   0.542880
 H     -2.270323   3.176294  -0.492162
 C     -0.000738  -2.352458  -0.675580
 H     -0.448987  -2.634624   0.297541
 C      2.052220  -1.321257  -1.496698
 H      2.546800  -2.243571  -1.886567
 H      1.450151  -0.936872  -2.362818
 C      3.102912  -0.302251  -1.183886
 C      3.843865   1.395182   0.258964
 C      4.944604   1.632654  -0.585548
 C      5.132723   0.877444  -1.751863
 C      4.191037  -0.109002  -2.052577
 C      0.282916  -3.667835  -1.424735
 H     -0.632092  -4.292671  -1.465356
 H      0.625267  -3.506406  -2.467350
 H      1.062192  -4.233166  -0.874514
 C      1.440326   0.705310   2.541275
 C      0.109776  -1.165092   2.064506
 H      2.173686  -2.806955   0.711712
 O      2.605354  -3.571448   1.192832
 C      4.001358  -3.540720   0.926192
 H      4.446506  -2.548611   1.190997
 H      4.214449  -3.698806  -0.162197
 H      4.278743  -0.734559  -2.953543
 H      5.658725   2.422666  -0.310412
 C      4.691934  -4.633624   1.734544
 H      5.784896  -4.639767   1.542890
 H      4.530188  -4.478603   2.822067
 H      4.287253  -5.633081   1.469048
 H      5.996677   1.059462  -2.409317
 C      3.695966   2.228502   1.499010
 H      2.691685   2.691154   1.549756
 H      3.819692   1.615087   2.413913
 H      4.458009   3.030345   1.515390
---------------------------------------
Mn11/iii
Frequencies, energies and thermodynamic properties:
 Lowest Vibrational Mode (1/cm) =                          21.7893
 2nd Lowest Vibrational Mode (1/cm) =                      25.7522
 E(RB-P86) (a.u.) =                                      -4446.78761150
 Thermal correction to Enthalpy (a.u.) =                   0.671254
 Thermal correction to Gibbs Free Energy (a.u.) =          0.550963
 Total Entropy (cal/Kmol) =                                253.174
 E(RPBE1PBE) (a.u.) =                                    -4446.11376729
Optimised cartesian coordinates (Angstrom):
 Fe-3.061235  -1.240887  -0.869997
 Mn1.229422  -0.224306   1.253135
 P     -0.354580   0.910312   0.067961
 O      1.127590   1.845984   3.362272
 O     -0.662433  -1.665741   2.973034
 N      1.205664  -1.697351  -0.224572
 N      2.903328   0.397871   0.053427
 C     -1.328065  -0.152220  -1.060960
 C     -1.068243  -1.570322  -1.286980
 C     -1.899573  -1.977973  -2.395340
 H     -1.959184  -2.991395  -2.812136
 C     -2.674499  -0.850351  -2.840729
 H     -3.417291  -0.856270  -3.649903
 C     -2.335925   0.276299  -2.015310
 H     -2.756274   1.286646  -2.100849
 C     -3.580165  -1.565843   1.099012
 H     -2.909648  -1.464493   1.961521
 C     -3.771989  -2.762404   0.324179
 H     -3.278273  -3.728421   0.495275
 C     -4.704374  -2.464172  -0.733071
 H     -5.042892  -3.161481  -1.511150
 C     -5.092035  -1.081845  -0.607716
 H     -5.779213  -0.542325  -1.273319
 C     -4.395143  -0.524864   0.522348
 H     -4.459258   0.512378   0.875687
 C      0.373968   2.211108  -1.047249
 C      0.959210   3.347211  -0.438590
 H      0.916964   3.466903   0.656363
 C      1.576733   4.338598  -1.218187
 H      2.019919   5.220257  -0.728461
 C      1.627974   4.206444  -2.619152
 H      2.113284   4.983229  -3.231025
 C      1.053987   3.079548  -3.231834
 H      1.084895   2.969362  -4.327558
 C      0.429722   2.086823  -2.452012
 H     -0.027376   1.217251  -2.948594
```

```
 C     -1.650288   1.909499   0.963375
 C     -2.036877   1.582292   2.281260
 H     -1.545400   0.758367   2.815989
 C     -3.057359   2.302572   2.928313
 H     -3.342398   2.033572   3.957761
 C     -3.707111   3.360436   2.268561
 H     -4.504763   3.924895   2.777025
 C     -3.326495   3.697973   0.957009
 H     -3.824322   4.528694   0.431887
 C     -2.303526   2.982052   0.311104
 H     -2.007379   3.271513  -0.709034
 C     -0.052425  -2.420429  -0.519739
 H     -0.497676  -2.663219   0.467080
 C      1.847905  -1.217771  -1.439755
 H      2.241700  -2.046839  -2.077091
 H      1.141213  -0.659981  -2.114107
 C      2.975493  -0.278478  -1.130453
 C      3.918514   1.265609   0.368750
 C      4.989555   1.483976  -0.520730
 C      5.050133   0.804614  -1.744113
 C      4.029423  -0.100369  -2.044971
 C      0.198359  -3.772701  -1.223714
 H     -0.733746  -4.372605  -1.269022
 H      0.566418  -3.648749  -2.263447
 H      0.951240  -4.348121  -0.648644
 C      1.237015   1.043642   2.502861
 C      0.047447  -1.069897   2.239165
 H      2.526328  -0.786144   2.238994
 H      2.345037  -1.482368   1.818983
 H      2.151171  -2.798869   0.488520
 O      2.707205  -3.467367   1.073722
 C      4.036526  -3.526552   0.590794
 H      4.069037  -3.838077  -0.485609
 H      4.537054  -2.523734   0.634204
 H      4.032567  -0.679372  -2.980393
 H      5.781831   2.189677  -0.231085
 C      4.845454  -4.521102   1.420197
 H      4.389916  -5.532773   1.369742
 H      5.891422  -4.592277   1.054851
 H      4.871223  -4.214062   2.487293
 H      5.887031   0.969516  -2.440195
 C      3.923590   1.989273   1.687082
 H      3.058501   2.673997   1.780262
 H      3.872669   1.281643   2.538866
 H      4.847612   2.589013   1.788826
---------------------------------------
Mn11/iv
Frequencies, energies and thermodynamic properties:
 Lowest Vibrational Mode (1/cm) =                        15.4849
 2nd Lowest Vibrational Mode (1/cm) =                    20.8253
 E(RB-P86) (a.u.) =                                     -4446.81603650
 Thermal correction to Enthalpy (a.u.) =                 0.675759
 Thermal correction to Gibbs Free Energy (a.u.) =        0.554036
 Total Entropy (cal/Kmol) =                              256.187
 E(RPBE1PBE) (a.u.) =                                   -4446.14090611
Optimised cartesian coordinates (Angstrom):
 Fe-3.116435  -1.152994  -0.892005
 Mn1.290675  -0.337098   1.062931
 P     -0.370484   0.922987   0.129298
 O      1.408164   1.332409   3.477271
 O     -0.360945  -2.066268   2.761175
 N      1.167514  -1.672240  -0.619093
 N      2.898337   0.383125  -0.149889
 C     -1.419081  -0.009118  -1.065771
 C     -1.166628  -1.387478  -1.485301
 C     -2.069032  -1.675409  -2.577295
 H     -2.149407  -2.630496  -3.111363
 C     -2.881248  -0.515393  -2.825413
 H     -3.679763  -0.437638  -3.575454
 C     -2.491851   0.507840  -1.895481
 H     -2.926986   1.513189  -1.826905
 C     -3.460675  -1.735416   1.053433
 H     -2.712986  -1.740648   1.857271
 C     -3.726599  -2.822019   0.147994
 H     -3.225451  -3.799362   0.147258
 C     -4.748865  -2.395691  -0.773302
 H     -5.158561  -2.988007  -1.602452
 C     -5.117973  -1.045124  -0.433945
 H     -5.859063  -0.428018  -0.959506
 C     -4.320488  -0.635217   0.692420
 H     -4.346403   0.347396   1.180621
 C      0.236225   2.366354  -0.886356
 C      0.858781   3.434592  -0.195357
 H      0.886911   3.430045   0.906997
 C      1.427136   4.510586  -0.896275
```

```
 H      1.897284   5.337006  -0.339439
 C      1.398349   4.532930  -2.304311
 H      1.847083   5.374899  -2.854907
 C      0.792280   3.473683  -3.000775
 H      0.761065   3.481819  -4.102201
 C      0.213499   2.398670  -2.297959
 H     -0.272954   1.587653  -2.861977
 C     -1.623065   1.775492   1.219357
 C     -1.886354   1.281438   2.515517
 H     -1.331400   0.411965   2.894681
 C     -2.859601   1.888808   3.330055
 H     -3.050241   1.489856   4.339146
 C     -3.582558   3.000623   2.862408
 H     -4.342538   3.477312   3.501826
 C     -3.323446   3.505107   1.574690
 H     -3.879655   4.379468   1.200282
 C     -2.348853   2.900203   0.761529
 H     -2.146400   3.317752  -0.237188
 C     -0.143889  -2.351897  -0.899459
 H     -0.502173  -2.676609   0.097393
 C      1.757586  -1.017450  -1.799094
 H      2.062440  -1.740824  -2.586642
 H      0.984692  -0.361267  -2.257274
 C      2.932047  -0.166225  -1.402067
 C      3.943832   1.194125   0.213615
 C      5.002375   1.469747  -0.677934
 C      5.027780   0.904313  -1.957135
 C      3.971622   0.059343  -2.317624
 C      0.042160  -3.616534  -1.754964
 H     -0.903361  -4.192447  -1.803563
 H      0.351481  -3.386330  -2.794949
 H      0.808386  -4.272710  -1.294381
 C      1.410517   0.698376   2.474427
 C      0.255548  -1.349672   2.041339
 H      2.474779  -1.240969   1.657592
 H      2.703936  -2.648352   1.333510
 H      1.818858  -2.423968  -0.305326
 O      2.862251  -3.522610   0.855656
 C      4.273765  -3.668467   0.674336
 H      4.414290  -4.466100  -0.087467
 H      4.720406  -2.737639   0.248788
 H      3.937741  -0.428415  -3.303216
 H      5.815012   2.130221  -0.341083
 C      4.993185  -4.049096   1.968150
 H      4.568931  -4.982634   2.393143
 H      6.076684  -4.211311   1.785422
 H      4.893860  -3.247994   2.731141
 H      5.856102   1.109349  -2.652411
 C      3.995346   1.784689   1.593113
 H      3.118506   2.430840   1.790720
 H      3.986739   0.987414   2.363525
 H      4.912156   2.391301   1.718473
---------------------------------------
Mn11/v
Frequencies, energies and thermodynamic properties:
 Lowest Vibrational Mode (1/cm) =                         15.7337
 2nd Lowest Vibrational Mode (1/cm) =                     28.8091
 E(RB-P86) (a.u.) =                                     -4291.87731277
 Thermal correction to Enthalpy (a.u.) =                  0.591156
 Thermal correction to Gibbs Free Energy (a.u.) =         0.483819
 Total Entropy (cal/Kmol) =                               225.910
 E(RPBE1PBE) (a.u.) =                                   -4291.19805023
Optimised cartesian coordinates (Angstrom):
 Fe3.055855  -0.868435   0.635143
 Mn-1.231945  -0.338446  -1.635515
 P     -0.158338   0.649215   0.117005
 O     -1.773941   2.192162  -3.023284
 O      0.978960  -0.495231  -3.553810
 N     -0.769063  -2.264536  -0.802136
 N     -3.061802  -0.796431  -0.638138
 C      1.064874  -0.461713   0.939346
 C      1.267066  -1.870302   0.597577
 C      2.145516  -2.433578   1.598610
 H      2.511122  -3.467608   1.627030
 C      2.501843  -1.402703   2.534746
 H      3.179876  -1.517927   3.391019
 C      1.847062  -0.189801   2.131059
 H      1.920807   0.779726   2.640324
 C      3.692442  -0.252168  -1.223789
 H      3.039303  -0.036336  -2.079458
 C      4.253360  -1.537579  -0.900007
 H      4.111577  -2.466205  -1.469232
 C      5.015428  -1.399181   0.314740
 H      5.549875  -2.204147   0.836853
 C      4.928633  -0.025454   0.739826
```

H 5.385870 0.399809 1.643337
C 4.109413 0.683310 -0.208434
H 3.832358 1.744108 -0.158922
C -1.261558 1.161509 1.533815
C -2.144447 2.247281 1.314364
H -2.091502 2.811591 0.368400
C -3.077003 2.626691 2.293407
H -3.746663 3.481664 2.106808
C -3.157810 1.917092 3.507491
H -3.891138 2.212021 4.274774
C -2.295673 0.830347 3.731653
H -2.347993 0.269012 4.678486
C -1.353512 0.455577 2.753617
H -0.674479 -0.387212 2.957203
C 0.810759 2.223502 -0.144655
C 1.287280 2.549374 -1.433024
H 1.060216 1.890022 -2.282584
C 2.055758 3.709437 -1.641298
H 2.418306 3.947567 -2.654092
C 2.356890 4.563114 -0.565110
H 2.956564 5.472630 -0.729092
C 1.880322 4.251897 0.721858
H 2.104840 4.917123 1.571100
C 1.110282 3.093866 0.929548
H 0.730701 2.873514 1.939600
C 0.674065 -2.627780 -0.583624
H 1.181427 -2.289343 -1.508565
C -1.630591 -2.506000 0.368565
H -1.719072 -3.583560 0.626273
H -1.158119 -2.007846 1.243794
C -2.995486 -1.911094 0.153638
C -4.293796 -0.213056 -0.799992
C -5.442914 -0.735395 -0.168628
H -6.406032 -0.228317 -0.328288
C -5.365189 -1.879518 0.632053
C -4.110128 -2.481277 0.786022
H -3.979564 -3.388768 1.394328
C 0.873774 -4.148391 -0.473915
H 1.953737 -4.395840 -0.489633
H 0.444334 -4.569104 0.458021
H 0.407081 -4.662170 -1.339573
C -1.615667 1.178784 -2.424085
C 0.125856 -0.417472 -2.729249
H -1.106190 -2.848654 -1.579865
H -1.926109 -1.162576 -2.802563
H -6.260748 -2.295653 1.118092
C -4.446373 0.991514 -1.683105
H -3.807406 1.827874 -1.339674
H -4.139635 0.759970 -2.723145
H -5.498823 1.333191 -1.691117

Mn11/vi_R
Frequencies, energies and thermodynamic properties:
Lowest Vibrational Mode (1/cm) = 10.3506
2nd Lowest Vibrational Mode (1/cm) = 18.9851
E(RB-P86) (a.u.) = -4714.58917947
Thermal correction to Enthalpy (a.u.) = 0.743912
Thermal correction to Gibbs Free Energy (a.u.) = 0.614207
Total Entropy (cal/Kmol) = 272.986
E(RPBE1PBE) (a.u.) = -4713.90593675
Optimised cartesian coordinates (Angstrom):
Fe-3.213708 -1.819433 -1.075347
Mn0.891169 0.250866 0.308310
P -1.254734 0.715760 0.523929
O 1.557344 0.538491 3.179316
O 0.665142 -2.540395 1.178985
N 0.730884 -0.177699 -1.595262
N 1.580373 2.099128 -0.515249
C -2.220469 -0.038593 -0.848392
C -1.646802 -0.807830 -1.949736
C -2.704384 -1.036379 -2.905725
H -2.611882 -1.601234 -3.842021
C -3.915135 -0.435516 -2.413323
H -4.895784 -0.461673 -2.907388
C -3.626278 0.173322 -1.144247
H -4.344208 0.714018 -0.514502
C -2.638330 -3.205679 0.336845
H -1.723824 -3.158503 0.941608
C -2.758137 -3.825159 -0.956252
H -1.955013 -4.336032 -1.504255
C -4.109646 -3.635994 -1.418349
H -4.515836 -3.972835 -2.381542
C -4.826633 -2.901312 -0.407189
H -5.875608 -2.581107 -0.465290
C -3.917392 -2.632562 0.676666

```
 H     -4.149426  -2.074045   1.592821
 C     -1.598617   2.533762   0.335508
 C     -1.377188   3.389129   1.441173
 H     -1.093085   2.963717   2.417504
 C     -1.535828   4.778817   1.313053
 H     -1.369726   5.429451   2.186416
 C     -1.906783   5.337578   0.074984
 H     -2.030322   6.427507  -0.025433
 C     -2.122944   4.496522  -1.030230
 H     -2.418919   4.924199  -2.001403
 C     -1.971250   3.102830  -0.902425
 H     -2.162729   2.455632  -1.772061
 C     -2.222992   0.313091   2.060966
 C     -1.760275  -0.661360   2.971353
 H     -0.799911  -1.166661   2.802078
 C     -2.521491  -0.999291   4.105466
 H     -2.143304  -1.760628   4.805928
 C     -3.754148  -0.367860   4.346150
 H     -4.348290  -0.632085   5.235283
 C     -4.220710   0.610420   3.448709
 H     -5.181487   1.117341   3.631858
 C     -3.459959   0.951634   2.317384
 H     -3.831161   1.733158   1.636542
 C     -0.183545  -1.248729  -2.060745
 H     -0.032150  -2.090197  -1.355963
 C      0.761046   0.957585  -2.504659
 H      1.276506   0.722516  -3.466535
 H     -0.272924   1.273985  -2.802399
 C      1.436746   2.135572  -1.874080
 C      2.170685   3.181869   0.095810
 C      2.609818   4.289090  -0.654711
 H      3.075718   5.133062  -0.125294
 C      2.463972   4.315514  -2.048764
 C      1.870305   3.213115  -2.666540
 H      1.731064   3.169804  -3.757116
 C      0.154083  -1.785678  -3.465344
 H     -0.442986  -2.694659  -3.681020
 H     -0.055717  -1.049800  -4.268337
 H      1.225013  -2.067992  -3.516178
 C      1.317932   0.515145   2.020229
 C      0.695365  -1.415318   0.806546
 H      2.253072  -0.782737  -1.795894
 H      3.020437  -0.341460   0.061170
 C      3.830284  -0.734103  -0.654842
 C      4.535947  -1.872995   0.069179
 C      4.944378   0.347930  -0.746751
 C      5.702817  -1.403385   0.711708
 C      4.166207  -3.224562   0.141371
 C      5.874178   0.086144   0.463423
 H      5.490548   0.159243  -1.697068
 C      6.508834  -2.289643   1.446225
 C      4.976104  -4.113503   0.875702
 H      3.260077  -3.578507  -0.375672
 H      6.930051   0.379335   0.284570
 C      6.138418  -3.648085   1.524618
 H      7.425317  -1.934620   1.946412
 H      4.704812  -5.179882   0.939436
 H      6.767294  -4.354079   2.091039
 O      3.237092  -1.077779  -1.870234
 H      4.541542   1.379825  -0.788536
 H      5.534612   0.662793   1.354711
 H      2.811332   5.178910  -2.636677
 C      2.371078   3.199548   1.583843
 H      1.413081   3.058525   2.120005
 H      3.049912   2.385623   1.907807
 H      2.811103   4.164263   1.899131
---------------------------------------
Mn11/vi_S
Frequencies, energies and thermodynamic properties:
 Lowest Vibrational Mode (1/cm) =                       16.6750
 2nd Lowest Vibrational Mode (1/cm) =                   20.4217
 E(RB-P86) (a.u.) =                                    -4714.58969943
 Thermal correction to Enthalpy (a.u.) =                0.743915
 Thermal correction to Gibbs Free Energy (a.u.) =       0.615272
 Total Entropy (cal/Kmol) =                             270.751
 E(RPBE1PBE) (a.u.) =                                  -4713.90672580
Optimised cartesian coordinates (Angstrom):
 Fe-3.667599  -1.369532  -0.940726
 Mn0.760836  -0.156623   0.474685
 P     -1.192939   0.864369   0.374180
 O      1.277203   0.535355   3.306628
 O     -0.243270  -2.576049   1.790272
 N      0.591386  -0.879891  -1.343091
 N      1.983177   1.251535  -0.577086
 C     -2.255524   0.119945  -0.928579
```

C	-1.847426	-0.964890	-1.817278
C	-2.877636	-1.104727	-2.818977
H	-2.888508	-1.844724	-3.629265
C	-3.912936	-0.139870	-2.560302
H	-4.839816	-0.017684	-3.136883
C	-3.540935	0.611084	-1.392943
H	-4.124383	1.422729	-0.939582
C	-3.547749	-2.572104	0.729488
H	-2.682018	-2.650963	1.399392
C	-3.773113	-3.367463	-0.448078
H	-3.113702	-4.159251	-0.828322
C	-5.002583	-2.926375	-1.056126
H	-5.441893	-3.319191	-1.982887
C	-5.539139	-1.858917	-0.250597
H	-6.459766	-1.296439	-0.456308
C	-4.639176	-1.637280	0.851357
H	-4.752789	-0.879787	1.637701
C	-1.020432	2.634598	-0.169125
C	-0.572228	3.589092	0.775276
H	-0.416205	3.291930	1.825287
C	-0.341898	4.920267	0.392020
H	-0.003097	5.652998	1.141655
C	-0.543976	5.316953	-0.944096
H	-0.361943	6.360837	-1.244883
C	-0.982863	4.374910	-1.890028
H	-1.148854	4.677329	-2.936294
C	-1.221544	3.041259	-1.506560
H	-1.583942	2.319764	-2.254659
C	-2.310544	1.039325	1.851427
C	-2.193802	0.165286	2.953775
H	-1.412968	-0.607006	2.969131
C	-3.075326	0.269784	4.045362
H	-2.967629	-0.419286	4.897961
C	-4.084518	1.248523	4.050967
H	-4.772841	1.330358	4.907069
C	-4.204948	2.128787	2.959868
H	-4.987503	2.904174	2.957161
C	-3.322637	2.028468	1.870413
H	-3.419284	2.737288	1.033458
C	-0.544215	-1.762481	-1.705817
H	-0.651690	-2.465534	-0.856045
C	0.937069	0.043535	-2.414339
H	1.386139	-0.470244	-3.297502
H	0.029139	0.565221	-2.816089
C	1.891963	1.090618	-1.930935
C	2.843447	2.214202	-0.101950
C	3.588915	3.020292	-0.983257
H	4.262712	3.779853	-0.560659
C	3.484374	2.853683	-2.371061
C	2.625612	1.862008	-2.849546
H	2.507199	1.673266	-3.926961
C	-0.282049	-2.623220	-2.957104
H	-1.080191	-3.383981	-3.073530
H	-0.251086	-2.025582	-3.891056
H	0.681606	-3.161120	-2.850246
C	1.127320	0.332328	2.150006
C	0.098847	-1.587116	1.231239
H	1.929495	-1.823614	-1.317771
H	2.650779	-1.239863	0.521989
C	3.315694	-2.042038	0.027672
C	3.348126	-3.262325	0.992146
C	4.440072	-2.923364	2.036344
O	2.845825	-2.298144	-1.258150
H	4.069413	3.481141	-3.060882
C	4.743998	-1.523249	0.104977
C	5.417151	-0.676507	-0.788651
C	5.386510	-2.021810	1.260361
C	6.752711	-0.320764	-0.513064
H	4.907866	-0.312861	-1.695868
C	6.716814	-1.663596	1.536173
C	7.396126	-0.809271	0.642702
H	7.301138	0.336935	-1.206888
H	7.230828	-2.052339	2.431037
H	8.442801	-0.529056	0.844391
H	2.357202	-3.489172	1.432551
H	4.001832	-2.366053	2.896404
H	3.660884	-4.139652	0.384046
H	4.941462	-3.816965	2.463728
C	3.018343	2.415885	1.375836
H	2.061372	2.683513	1.863920
H	3.387720	1.492539	1.864475
H	3.745940	3.226776	1.567562

Mn11/viii

Frequencies, energies and thermodynamic properties:

```
 Lowest Vibrational Mode (1/cm) =                          18.8619
 2nd Lowest Vibrational Mode (1/cm) =                      31.2598
 E(RB-P86) (a.u.) =                                       -4291.84331657
 Thermal correction to Enthalpy (a.u.) =                   0.586453
 Thermal correction to Gibbs Free Energy (a.u.) =          0.479015
 Total Entropy (cal/Kmol) =                                226.123
 E(RPBE1PBE) (a.u.) =                                     -4291.16473138
Optimised cartesian coordinates (Angstrom):
 Fe3.070639  -0.853985   0.645914
 Mn-1.194552  -0.337571  -1.696184
 P     -0.157941   0.588587   0.092886
 O     -1.734019   2.339269  -2.843219
 O      1.150574  -0.257006  -3.457549
 N     -0.728396  -2.182864  -0.934285
 N     -3.031734  -0.801746  -0.670240
 C      1.056102  -0.524912   0.897643
 C      1.299718  -1.905642   0.494532
 C      2.162325  -2.489399   1.494353
 H      2.552765  -3.515223   1.483071
 C      2.465579  -1.495816   2.491019
 H      3.117043  -1.633666   3.364777
 C      1.794999  -0.278455   2.123664
 H      1.827438   0.666199   2.681696
 C      3.762862  -0.103376  -1.145467
 H      3.139476   0.171338  -2.005646
 C      4.320047  -1.405957  -0.896682
 H      4.200620  -2.291808  -1.534882
 C      5.039877  -1.352413   0.350685
 H      5.562723  -2.190455   0.830966
 C      4.930066  -0.012583   0.870479
 H      5.355950   0.348693   1.816271
 C      4.138169   0.759198  -0.051994
 H      3.854633   1.813457   0.062339
 C     -1.342840   1.020216   1.462579
 C     -2.201860   2.129601   1.275550
 H     -2.107503   2.751122   0.370025
 C     -3.161970   2.463055   2.244892
 H     -3.816690   3.334914   2.086878
 C     -3.285586   1.687718   3.413762
 H     -4.038813   1.948550   4.174056
 C     -2.440082   0.581681   3.605892
 H     -2.525924  -0.028221   4.519409
 C     -1.473950   0.248625   2.636961
 H     -0.810875  -0.613512   2.806851
 C      0.765206   2.201418  -0.081162
 C      1.276059   2.614466  -1.330712
 H      1.098897   2.009427  -2.230002
 C      2.020586   3.803042  -1.443495
 H      2.408275   4.109194  -2.428118
 C      2.265880   4.597203  -0.309799
 H      2.847338   5.528488  -0.399723
 C      1.756737   4.198100   0.939622
 H      1.936845   4.815356   1.834236
 C      1.009057   3.013181   1.052282
 H      0.603187   2.726385   2.034720
 C      0.666863  -2.576806  -0.733163
 H      1.224228  -2.220329  -1.624228
 C     -1.573912  -2.574530   0.164668
 H     -1.728239  -3.683417   0.228650
 H     -1.160973  -2.306901   1.184426
 C     -2.926405  -1.931328   0.089160
 C     -4.258904  -0.191620  -0.758945
 C     -5.373495  -0.695225  -0.061022
 C     -5.258086  -1.844549   0.733397
 C     -4.014139  -2.474994   0.798305
 C      0.860735  -4.109852  -0.692711
 H      1.937508  -4.376588  -0.726963
 H      0.433108  -4.568595   0.223149
 H      0.364610  -4.567782  -1.572659
 C     -1.601444   1.260487  -2.377058
 C      0.245425  -0.280139  -2.695175
 H     -1.790820  -1.587753  -2.782733
 H     -2.157392  -0.922773  -3.089501
 H     -6.338028  -0.175813  -0.159145
 H     -3.863888  -3.390803   1.389545
 H     -6.127578  -2.243374   1.278566
 C     -4.445225   1.021906  -1.627146
 H     -3.827043   1.871139  -1.276985
 H     -4.151625   0.817113  -2.676148
 H     -5.504878   1.340089  -1.617028
---------------------------------------
Mn11/ix
Frequencies, energies and thermodynamic properties:
 Lowest Vibrational Mode (1/cm) =                          23.3000
 2nd Lowest Vibrational Mode (1/cm) =                      32.1058
```

```
 E(RB-P86) (a.u.) =                                          -4445.63119472
 Thermal correction to Enthalpy (a.u.) =                      0.655844
 Thermal correction to Gibbs Free Energy (a.u.) =             0.540398
 Total Entropy (cal/Kmol) =                                   242.978
 E(RPBE1PBE) (a.u.) =                                        -4444.95680240
Optimised cartesian coordinates (Angstrom):
 Fe-3.256766  -0.603081  -0.880789
 Mn1.282818  -0.825084   0.912214
 P     -0.098811   0.791596   0.122424
 O      1.880991   0.659444   3.378739
 O     -0.755279  -2.077888   2.602998
 N      0.802593  -2.017529  -0.785327
 N      2.919757  -0.296949  -0.432803
 C     -1.346183   0.131932  -1.071252
 C     -1.423359  -1.251033  -1.542035
 C     -2.393688  -1.287940  -2.611902
 H     -2.699581  -2.180752  -3.171672
 C     -2.926334   0.033867  -2.799865
 H     -3.702991   0.318816  -3.522255
 C     -2.293116   0.907513  -1.851836
 H     -2.486950   1.982144  -1.743232
 C     -3.693188  -1.130654   1.063128
 H     -2.954637  -1.309527   1.855062
 C     -4.197628  -2.119592   0.147379
 H     -3.918059  -3.181566   0.125368
 C     -5.115753  -1.468311  -0.751746
 H     -5.653704  -1.944758  -1.582292
 C     -5.180803  -0.075473  -0.388723
 H     -5.777540   0.695197  -0.894877
 C     -4.300008   0.134487   0.730782
 H     -4.106898   1.091823   1.231951
 C      0.745964   2.119199  -0.882086
 C      1.475004   3.120576  -0.196874
 H      1.454030   3.157108   0.904731
 C      2.210004   4.086118  -0.904498
 H      2.762691   4.863160  -0.352508
 C      2.239479   4.062121  -2.312031
 H      2.816883   4.818031  -2.867524
 C      1.522822   3.069796  -3.002663
 H      1.533468   3.045008  -4.104146
 C      0.780188   2.106167  -2.293749
 H      0.208686   1.349673  -2.853632
 C     -1.122621   1.840296   1.278749
 C     -1.406079   1.396447   2.588757
 H     -0.998217   0.442985   2.949869
 C     -2.215750   2.163122   3.447193
 H     -2.423402   1.799304   4.466074
 C     -2.753325   3.386565   3.010908
 H     -3.385363   3.987075   3.684291
 C     -2.471408   3.842734   1.709996
 H     -2.880978   4.803361   1.358789
 C     -1.660072   3.078775   0.853428
 H     -1.435023   3.461771  -0.153861
 C     -0.606746  -2.432381  -1.030782
 H     -0.990967  -2.719371  -0.031580
 C      1.527491  -1.594185  -1.987361
 H      1.720063  -2.434669  -2.689932
 H      0.892709  -0.867077  -2.543024
 C      2.829306  -0.918422  -1.641913
 C      4.074834   0.375823  -0.141525
 C      5.139768   0.434707  -1.065390
 C      5.045788  -0.214095  -2.301601
 C      3.867213  -0.908815  -2.590236
 C     -0.715964  -3.670890  -1.936694
 H     -1.758628  -4.046636  -1.949604
 H     -0.420783  -3.460181  -2.984706
 H     -0.072260  -4.486058  -1.547654
 C      1.707462   0.080748   2.358526
 C      0.033641  -1.546355   1.893146
 H      1.354011  -2.759443  -0.278944
 H      3.735259  -1.442706  -3.543196
 H      6.048393   0.990821  -0.791378
 H      5.877210  -0.182046  -3.022515
 O      2.460078  -2.474888   1.076014
 C      2.632512  -3.238429   2.233136
 C      3.646429  -2.657000   3.232289
 H      2.997973  -4.258228   1.934975
 H      1.668353  -3.424325   2.781216
 H      3.797743  -3.342348   4.095532
 H      4.631579  -2.500206   2.741774
 H      3.302784  -1.679183   3.628830
 C      4.238578   1.058236   1.187278
 H      3.398439   1.748085   1.391675
 H      4.261023   0.317548   2.011432
 H      5.182827   1.634509   1.211068
```

```
---------------------------------------
Mn11/x
Frequencies, energies and thermodynamic properties:
 Lowest Vibrational Mode (1/cm) =                          17.7555
 2nd Lowest Vibrational Mode (1/cm) =                      27.1346
 E(RB-P86) (a.u.) =                                      -4600.57222871
 Thermal correction to Enthalpy (a.u.) =                   0.740056
 Thermal correction to Gibbs Free Energy (a.u.) =          0.611495
 Total Entropy (cal/Kmol) =                                270.579
 E(RPBE1PBE) (a.u.) =                                    -4599.91013041
Optimised cartesian coordinates (Angstrom):
 Fe-3.264945  -1.425348  -0.775531
 Mn1.145719  -0.100231   0.941281
 P     -0.672289   0.928286   0.037762
 O      1.087971   1.828586   3.159071
 O     -0.429593  -1.814397   2.721194
 N      1.060215  -1.523496  -0.682542
 N      2.565246   0.760105  -0.470010
 C     -1.675256  -0.155235  -1.064399
 C     -1.321832  -1.525348  -1.430956
 C     -2.233023  -1.939526  -2.472977
 H     -2.249270  -2.922966  -2.959202
 C     -3.148686  -0.864166  -2.741622
 H     -3.976673  -0.888637  -3.462741
 C     -2.817135   0.231356  -1.873390
 H     -3.333592   1.198918  -1.834191
 C     -3.523832  -1.917220   1.209211
 H     -2.769252  -1.800785   1.997408
 C     -3.693530  -3.078477   0.376678
 H     -3.098899  -4.000624   0.425535
 C     -4.764528  -2.811140  -0.548961
 H     -5.124658  -3.490297  -1.333309
 C     -5.259442  -1.483988  -0.285226
 H     -6.063498  -0.975225  -0.833726
 C     -4.491179  -0.929684   0.798858
 H     -4.606843   0.074202   1.227482
 C     -0.246161   2.371228  -1.065921
 C      0.255332   3.543637  -0.450631
 H      0.304884   3.606686   0.648885
 C      0.672979   4.638266  -1.224752
 H      1.051365   5.545459  -0.727148
 C      0.608684   4.576657  -2.630235
 H      0.938528   5.433962  -3.238146
 C      0.117320   3.415948  -3.251207
 H      0.057247   3.359112  -4.349852
 C     -0.309616   2.320645  -2.475310
 H     -0.710958   1.428558  -2.980613
 C     -1.959053   1.715360   1.138563
 C     -2.143079   1.274824   2.467287
 H     -1.502317   0.486377   2.883821
 C     -3.149691   1.833883   3.275750
 H     -3.274839   1.477316   4.310496
 C     -3.988088   2.842638   2.769795
 H     -4.774544   3.281079   3.404411
 C     -3.810304   3.293392   1.449057
 H     -4.456935   4.087506   1.042779
 C     -2.801986   2.738060   0.642120
 H     -2.666983   3.115926  -0.383117
 C     -0.181271  -2.351919  -0.855561
 H     -0.454958  -2.661664   0.172431
 C      1.511773  -0.892679  -1.935408
 H      1.868996  -1.635816  -2.681280
 H      0.648202  -0.372837  -2.405829
 C      2.591593   0.120784  -1.672651
 C      3.519844   1.709403  -0.225892
 C      4.486921   2.039666  -1.199074
 C      4.505702   1.384517  -2.435072
 C      3.543653   0.396733  -2.669349
 C      0.092503  -3.634581  -1.660076
 H     -0.784784  -4.310577  -1.617579
 H      0.308417  -3.428963  -2.728462
 H      0.957960  -4.171667  -1.222519
 C      1.168052   1.077919   2.246224
 C      0.164652  -1.105518   1.979187
 H      3.195347  -2.269852   0.722325
 H      1.821313  -2.193158  -0.401402
 O      3.270886  -3.128515   0.108415
 C      4.470090  -3.075061  -0.649231
 H      4.343899  -3.733297  -1.540468
 H      4.656684  -2.045863  -1.047593
 H      3.516524  -0.165465  -3.614543
 H      5.231161   2.814554  -0.963400
 C      5.692699  -3.538384   0.148530
 H      5.538824  -4.567673   0.536049
 H      6.607684  -3.538469  -0.482166
```

```
 H      5.877378  -2.871055   1.017084
 H      5.262652   1.631516  -3.195314
 O      2.865811  -1.092075   1.510572
 C      3.129752  -1.480497   2.842114
 C      3.951272  -0.462323   3.643299
 H      3.701467  -2.444960   2.819633
 H      2.193043  -1.709480   3.410140
 H      4.194039  -0.861433   4.652532
 H      4.907861  -0.233560   3.126777
 H      3.395960   0.488046   3.782624
 C      3.560830   2.410235   1.100732
 H      2.627224   2.975430   1.287312
 H      3.667288   1.670976   1.916942
 H      4.410421   3.117757   1.140089
---------------------------------------
Mn11/TS-i
Frequencies, energies and thermodynamic properties:
 Lowest Vibrational Mode (1/cm) =-748.3503
 2nd Lowest Vibrational Mode (1/cm) =                      21.9247
 E(RB-P86) (a.u.) =                                      -4446.78548001
 Thermal correction to Enthalpy (a.u.) =                   0.667461
 Thermal correction to Gibbs Free Energy (a.u.) =          0.548934
 Total Entropy (cal/Kmol) =                                249.463
 E(RPBE1PBE) (a.u.) =                                    -4446.10910451
Optimised cartesian coordinates (Angstrom):
 Fe-3.087835  -1.171287  -0.874028
 Mn1.236993  -0.279745   1.226327
 P     -0.328456   0.911893   0.074144
 O      1.216117   1.731809   3.386533
 O     -0.668844  -1.702141   2.947111
 N      1.166316  -1.738293  -0.284303
 N      2.916898   0.314284   0.022417
 C     -1.335419  -0.114726  -1.063411
 C     -1.109443  -1.535021  -1.316922
 C     -1.959048  -1.907194  -2.423904
 H     -2.044176  -2.911955  -2.856790
 C     -2.712720  -0.755741  -2.842728
 H     -3.463211  -0.732666  -3.644355
 C     -2.341750   0.349288  -2.002443
 H     -2.741202   1.369570  -2.067194
 C     -3.594947  -1.514340   1.094685
 H     -2.913764  -1.440532   1.951678
 C     -3.820789  -2.694714   0.304121
 H     -3.347514  -3.674056   0.456274
 C     -4.756661  -2.360389  -0.738999
 H     -5.117781  -3.038289  -1.524004
 C     -5.112410  -0.972054  -0.589457
 H     -5.793437  -0.407534  -1.240441
 C     -4.392459  -0.447460   0.541584
 H     -4.429699   0.585740   0.910216
 C      0.417418   2.208667  -1.034876
 C      1.036879   3.322573  -0.418857
 H      1.006568   3.430698   0.677743
 C      1.673768   4.306296  -1.192457
 H      2.143059   5.170928  -0.696712
 C      1.711816   4.188053  -2.595156
 H      2.212667   4.958637  -3.202312
 C      1.104841   3.082853  -3.215340
 H      1.125262   2.983460  -4.312345
 C      0.460229   2.098478  -2.441377
 H     -0.023951   1.247618  -2.944779
 C     -1.595072   1.927498   0.991654
 C     -1.976095   1.591072   2.308863
 H     -1.497876   0.748897   2.827036
 C     -2.973883   2.325274   2.975443
 H     -3.255253   2.048902   4.003949
 C     -3.605707   3.406406   2.336246
 H     -4.385548   3.981677   2.859997
 C     -3.229967   3.753345   1.025703
 H     -3.713758   4.602287   0.516846
 C     -2.229718   3.023378   0.360237
 H     -1.936268   3.320041  -0.658639
 C     -0.117013  -2.429580  -0.577298
 H     -0.551616  -2.671699   0.413832
 C      1.819963  -1.251610  -1.498237
 H      2.181632  -2.078764  -2.152904
 H      1.110728  -0.665692  -2.136580
 C      2.974124  -0.349813  -1.169427
 C      3.954773   1.150799   0.347249
 C      5.032555   1.349881  -0.539249
 C      5.078772   0.680546  -1.768086
 C      4.034481  -0.194231  -2.079447
 C      0.101796  -3.775009  -1.299485
 H     -0.841549  -4.356665  -1.339113
 H      0.460663  -3.646204  -2.341538
```

```
 H      0.848631  -4.372131  -0.738715
 C      1.289906   0.952482   2.502172
 C      0.048966  -1.113628   2.214169
 H      2.507855  -0.948948   2.145137
 H      2.364151  -1.702137   1.751859
 H      1.943819  -2.604906   0.339709
 O      2.582712  -3.271417   1.109509
 C      3.931387  -3.410755   0.731432
 H      4.035287  -3.569002  -0.375137
 H      4.523175  -2.479822   0.953841
 H      4.024475  -0.764687  -3.020011
 H      5.842138   2.032183  -0.241492
 C      4.588555  -4.586642   1.459755
 H      4.057626  -5.534818   1.228679
 H      5.653838  -4.703432   1.165608
 H      4.548421  -4.437552   2.559978
 H      5.921429   0.829031  -2.460775
 C      3.978292   1.857584   1.673827
 H      3.127965   2.559071   1.777787
 H      3.912809   1.138267   2.514710
 H      4.915224   2.435828   1.781425
---------------------------------------
Mn11/TS-ii_si
Frequencies, energies and thermodynamic properties:
 Lowest Vibrational Mode (1/cm) =-260.4888
 2nd Lowest Vibrational Mode (1/cm) =                     13.4991
 E(RB-P86) (a.u.) =                                     -4714.58355022
 Thermal correction to Enthalpy (a.u.) =                 0.740386
 Thermal correction to Gibbs Free Energy (a.u.) =        0.614082
 Total Entropy (cal/Kmol) =                              265.830
 E(RPBE1PBE) (a.u.) =                                   -4713.89485687
Optimised cartesian coordinates (Angstrom):
 Fe-3.243155  -1.636277  -1.049563
 Mn1.064795   0.175482   0.299988
 P     -1.108551   0.788584   0.510123
 O      1.743253   0.393878   3.152473
 O      0.719057  -2.636973   1.051409
 N      0.783518  -0.222860  -1.746653
 N      1.752673   2.013814  -0.535065
 C     -2.162130   0.096358  -0.835239
 C     -1.664761  -0.691489  -1.963193
 C     -2.763981  -0.857450  -2.887046
 H     -2.731819  -1.415172  -3.831355
 C     -3.925933  -0.205128  -2.347994
 H     -4.922768  -0.181759  -2.808399
 C     -3.564271   0.376150  -1.085453
 H     -4.233121   0.942523  -0.424975
 C     -2.679801  -3.070906   0.317290
 H     -1.737287  -3.078263   0.879773
 C     -2.889112  -3.663828  -0.977284
 H     -2.139082  -4.207462  -1.567476
 C     -4.248182  -3.399187  -1.375229
 H     -4.713544  -3.699759  -2.323539
 C     -4.880427  -2.644592  -0.323307
 H     -5.912704  -2.269658  -0.329973
 C     -3.911420  -2.439105   0.721597
 H     -4.073133  -1.882559   1.653775
 C     -1.420178   2.622242   0.367695
 C     -1.078437   3.446619   1.466973
 H     -0.731839   2.988887   2.408196
 C     -1.195841   4.843754   1.382165
 H     -0.934649   5.466683   2.252638
 C     -1.645934   5.445545   0.191329
 H     -1.736790   6.541230   0.123690
 C     -1.981695   4.637647  -0.908456
 H     -2.340186   5.097071  -1.843568
 C     -1.871113   3.236417  -0.821547
 H     -2.159632   2.620327  -1.687379
 C     -2.065002   0.396413   2.063218
 C     -1.629172  -0.632513   2.925825
 H     -0.706413  -1.184624   2.701105
 C     -2.368952  -0.966116   4.075427
 H     -2.011703  -1.771412   4.736861
 C     -3.554266  -0.274674   4.381210
 H     -4.131788  -0.534702   5.282557
 C     -3.994279   0.757545   3.531932
 H     -4.918165   1.310658   3.765240
 C     -3.254076   1.092997   2.384909
 H     -3.603780   1.915440   1.741699
 C     -0.252173  -1.230064  -2.156178
 H     -0.101645  -2.077430  -1.458679
 C      0.712307   1.029178  -2.517265
 H      1.002450   0.890979  -3.581349
 H     -0.342564   1.385064  -2.522198
 C      1.566318   2.093062  -1.888238
```

```
 C      2.444421   3.035852   0.068827
 C      2.959122   4.115249  -0.679616
 H      3.505220   4.909444  -0.149607
 C      2.786872   4.172514  -2.067001
 C      2.075210   3.135390  -2.679154
 H      1.900051   3.122553  -3.765134
 C     -0.008093  -1.762449  -3.578918
 H     -0.663756  -2.632671  -3.781305
 H     -0.207706  -1.000997  -4.360230
 H      1.042106  -2.103668  -3.678811
 C      1.477567   0.389861   1.996788
 C      0.804758  -1.496846   0.733162
 H      1.730295  -0.646085  -1.966101
 H      2.619071  -0.337551   0.026440
 C      3.847188  -0.897209  -0.985064
 C      4.161539  -2.092605  -0.124108
 C      4.853099   0.193211  -0.538474
 C      5.038035  -1.718160   0.918435
 C      3.759546  -3.426545  -0.300632
 C      5.310544  -0.224899   0.876188
 H      5.703900   0.127455  -1.255201
 C      5.517792  -2.690656   1.811501
 C      4.243407  -4.398590   0.592877
 H      3.085773  -3.695159  -1.129884
 H      6.369350   0.025264   1.094308
 C      5.114569  -4.030908   1.641715
 H      6.206042  -2.416827   2.628153
 H      3.948781  -5.453727   0.473856
 H      5.490001  -4.803711   2.331926
 O      3.346723  -0.964721  -2.143849
 H      4.441124   1.217241  -0.612737
 H      4.696230   0.283214   1.654533
 H      3.197190   5.006941  -2.655967
 C      2.673474   3.022571   1.553022
 H      1.719530   2.920555   2.104301
 H      3.313045   2.168961   1.853956
 H      3.169526   3.958017   1.873568
---------------------------------------
Mn11/TS-ii_re
Frequencies, energies and thermodynamic properties:
 Lowest Vibrational Mode (1/cm) =-245.6557
 2nd Lowest Vibrational Mode (1/cm) =                    14.7359
 E(RB-P86) (a.u.) =                                     -4714.58502795
 Thermal correction to Enthalpy (a.u.) =                 0.740679
 Thermal correction to Gibbs Free Energy (a.u.) =        0.614860
 Total Entropy (cal/Kmol) =                              264.809
 E(RPBE1PBE) (a.u.) =                                   -4713.89776102
Optimised cartesian coordinates (Angstrom):
 Fe-3.737272  -1.152592  -0.849162
 Mn0.870289  -0.320386   0.464606
 P     -1.040748   0.897376   0.346695
 O      1.479686   0.322165   3.267941
 O     -0.271178  -2.702689   1.732541
 N      0.539316  -1.055665  -1.478087
 N      2.086418   1.021303  -0.661662
 C     -2.219457   0.230721  -0.903956
 C     -1.935969  -0.890007  -1.800747
 C     -3.015040  -0.953956  -2.760025
 H     -3.117604  -1.693474  -3.564074
 C     -3.960976   0.087600  -2.464661
 H     -4.900292   0.275288  -3.001957
 C     -3.481468   0.813350  -1.321635
 H     -3.980340   1.668542  -0.847904
 C     -3.634917  -2.357190   0.819977
 H     -2.747068  -2.499797   1.449423
 C     -3.972686  -3.134963  -0.342762
 H     -3.393570  -3.975583  -0.748169
 C     -5.191566  -2.603239  -0.896957
 H     -5.700322  -2.962766  -1.801442
 C     -5.609246  -1.497455  -0.073262
 H     -6.492804  -0.866912  -0.240433
 C     -4.646627  -1.343050   0.986126
 H     -4.666167  -0.577089   1.772131
 C     -0.800360   2.660670  -0.210890
 C     -0.201030   3.565671   0.698429
 H      0.023461   3.239564   1.727443
 C      0.095342   4.882805   0.311730
 H      0.551743   5.574856   1.037484
 C     -0.188763   5.317112  -0.997495
 H      0.045566   6.349396  -1.302238
 C     -0.775086   4.424851  -1.911055
 H     -1.005260   4.754917  -2.936782
 C     -1.080408   3.106230  -1.521396
 H     -1.557631   2.428388  -2.245993
 C     -2.103687   1.152326   1.857800
```

```
 C     -2.023282   0.251297   2.941659
 H     -1.310497  -0.584288   2.909790
 C     -2.852834   0.408346   4.067224
 H     -2.774632  -0.304004   4.903946
 C     -3.773419   1.469522   4.126879
 H     -4.420812   1.593469   5.009547
 C     -3.857183   2.377016   3.054723
 H     -4.570652   3.215699   3.093691
 C     -3.026577   2.222344   1.931037
 H     -3.093237   2.951383   1.108444
 C     -0.725852  -1.815064  -1.758417
 H     -0.835582  -2.495215  -0.891133
 C      0.828193  -0.020128  -2.483817
 H      1.097316  -0.450703  -3.472929
 H     -0.092112   0.584865  -2.645940
 C      1.922125   0.896359  -2.013114
 C      3.043482   1.899731  -0.218104
 C      3.805855   2.669122  -1.121735
 H      4.552150   3.368379  -0.717070
 C      3.630087   2.535350  -2.503080
 C      2.676149   1.616698  -2.953477
 H      2.496038   1.454995  -4.026723
 C     -0.602409  -2.695847  -3.013703
 H     -1.469971  -3.381310  -3.089272
 H     -0.558183  -2.102899  -3.949990
 H      0.312422  -3.318708  -2.945686
 C      1.269999   0.139681   2.114903
 C      0.132361  -1.718309   1.201552
 H      1.333229  -1.756222  -1.529803
 H      2.231713  -1.259364   0.424071
 C      3.222747  -2.415382  -0.307768
 C      4.482490  -1.659859   0.021834
 C      3.060024  -3.441565   0.840882
 C      4.884856  -1.952163   1.344972
 C      5.252654  -0.835764  -0.815032
 C      3.876073  -2.866131   2.018742
 C      6.076287  -1.405332   1.848784
 C      6.446748  -0.291737  -0.308014
 H      4.923704  -0.637595  -1.847891
 C      6.852577  -0.573937   1.014597
 H      6.409158  -1.627014   2.876244
 H      7.074594   0.351649  -0.945221
 H      7.793359  -0.145798   1.396993
 O      2.775095  -2.579365  -1.477814
 H      4.354756  -3.641658   2.651735
 H      3.220148  -2.264288   2.688316
 H      2.003672  -3.669074   1.074373
 H      3.531297  -4.379838   0.466856
 H      4.229856   3.126889  -3.211595
 C      3.310172   2.049794   1.251856
 H      2.403425   2.375751   1.797007
 H      3.628768   1.085778   1.695960
 H      4.108193   2.796765   1.422727
---------------------------------------
Mn11/TS-iii
Frequencies, energies and thermodynamic properties:
 Lowest Vibrational Mode (1/cm) =-643.7692
 2nd Lowest Vibrational Mode (1/cm) =                      23.8030
 E(RB-P86) (a.u.) =                                      -4291.83988757
 Thermal correction to Enthalpy (a.u.) =                   0.585491
 Thermal correction to Gibbs Free Energy (a.u.) =          0.479470
 Total Entropy (cal/Kmol) =                                223.141
 E(RPBE1PBE) (a.u.) =                                    -4291.15815963
Optimised cartesian coordinates (Angstrom):
 Fe3.044327  -0.852002   0.660911
 Mn-1.175818  -0.380662  -1.685520
 P     -0.169845   0.628389   0.092102
 O     -1.759428   2.107725  -3.167679
 O      1.186142  -0.313348  -3.421974
 N     -0.751187  -2.261565  -0.886387
 N     -3.024127  -0.796886  -0.651301
 C      1.031934  -0.504608   0.899811
 C      1.271090  -1.897810   0.520193
 C      2.126595  -2.466927   1.535841
 H      2.512176  -3.494372   1.544693
 C      2.431127  -1.459367   2.516114
 H      3.079931  -1.586021   3.393388
 C      1.767327  -0.246777   2.125778
 H      1.803369   0.706999   2.667732
 C      3.752537  -0.123141  -1.133482
 H      3.135514   0.146282  -1.999715
 C      4.300307  -1.426721  -0.868166
 H      4.180514  -2.317904  -1.498843
 C      5.012784  -1.364976   0.382796
 H      5.527567  -2.201183   0.874818
```

```
 C      4.907434  -0.019509   0.888666
 H      5.328928   0.348390   1.833854
 C      4.126090   0.747768  -0.046448
 H      3.847308   1.804516   0.056310
 C     -1.329674   1.092071   1.474533
 C     -2.163211   2.222107   1.295324
 H     -2.057996   2.845372   0.392047
 C     -3.112700   2.574784   2.268592
 H     -3.746714   3.462720   2.115410
 C     -3.252394   1.798315   3.434736
 H     -3.997251   2.073989   4.198022
 C     -2.433451   0.671063   3.619309
 H     -2.531745   0.058966   4.530145
 C     -1.478573   0.319114   2.646161
 H     -0.837254  -0.560430   2.811376
 C      0.775071   2.223652  -0.111114
 C      1.250619   2.619946  -1.379954
 H      1.038408   2.007429  -2.266695
 C      2.003832   3.799458  -1.527186
 H      2.364244   4.092458  -2.526088
 C      2.291644   4.601464  -0.409155
 H      2.879731   5.525625  -0.525641
 C      1.815887   4.220141   0.859094
 H      2.028844   4.844678   1.741381
 C      1.060425   3.044102   1.006283
 H      0.680076   2.771792   2.003015
 C      0.653680  -2.626315  -0.682932
 H      1.194500  -2.292044  -1.592165
 C     -1.608473  -2.589475   0.228061
 H     -1.766679  -3.690644   0.350031
 H     -1.182895  -2.257270   1.218236
 C     -2.954031  -1.930987   0.106947
 C     -4.234619  -0.158332  -0.747426
 C     -5.371525  -0.645289  -0.071388
 C     -5.295528  -1.807636   0.705868
 C     -4.064324  -2.462913   0.787109
 C      0.863863  -4.153550  -0.588551
 H      1.943324  -4.409362  -0.606074
 H      0.433158  -4.584201   0.339229
 H      0.380934  -4.647124  -1.456204
 C     -1.603901   1.119033  -2.538882
 C      0.276120  -0.337629  -2.664348
 H     -1.471393  -1.926713  -2.264474
 H     -1.889301  -1.368325  -2.875282
 H     -6.321100  -0.099869  -0.173815
 H     -3.943007  -3.386389   1.372782
 H     -6.182403  -2.195941   1.230094
 C     -4.378957   1.074128  -1.594801
 H     -3.683508   1.871372  -1.269015
 H     -4.153238   0.857970  -2.658149
 H     -5.411960   1.465032  -1.530885
---------------------------------------
Mn12/i
Frequencies, energies and thermodynamic properties:
 Lowest Vibrational Mode (1/cm) =                         23.0082
 2nd Lowest Vibrational Mode (1/cm) =                     27.4507
 E(RB-P86) (a.u.) =                                     -4710.89052578
 Thermal correction to Enthalpy (a.u.) =                  0.533763
 Thermal correction to Gibbs Free Energy (a.u.) =         0.427730
 Total Entropy (cal/Kmol) =                               223.166
 E(RPBE1PBE) (a.u.) =                                   -4710.18072241
Optimised cartesian coordinates (Angstrom):
 Fe3.197812  -0.841080   0.559818
 Mn-1.058413  -0.367103  -1.527828
 P     -0.056108   0.627779   0.151693
 O     -1.840308   2.021907  -3.107694
 O      1.154290  -0.145987  -3.432503
 N     -0.603216  -2.110651  -0.940509
 N     -2.914820  -0.900596  -0.628824
 C      1.197329  -0.512006   0.881666
 C      1.424474  -1.889568   0.452112
 C      2.320182  -2.494469   1.408931
 H      2.708463  -3.520175   1.365653
 C      2.653634  -1.519712   2.413879
 H      3.332285  -1.674921   3.263613
 C      1.971855  -0.296326   2.090803
 H      2.022874   0.636832   2.666444
 C      3.822561  -0.079425  -1.250547
 H      3.167643   0.178407  -2.092770
 C      4.417757  -1.367952  -1.014197
 H      4.302616  -2.258165  -1.647196
 C      5.171058  -1.294207   0.211882
 H      5.727025  -2.118254   0.678908
 C      5.043445   0.043507   0.732180
 H      5.485607   0.417015   1.665627
```

```
 C      4.208134   0.793932  -0.169536
 H      3.901305   1.840920  -0.047905
 C     -1.209410   0.929572   1.579816
 C     -2.077430   2.046235   1.533693
 H     -2.008390   2.762019   0.699206
 C     -3.028641   2.253535   2.545944
 H     -3.690541   3.133100   2.499978
 C     -3.139886   1.340182   3.611995
 H     -3.887464   1.502765   4.404572
 C     -2.291879   0.220142   3.658459
 H     -2.371144  -0.499869   4.488678
 C     -1.331678   0.014535   2.649690
 H     -0.663700  -0.858582   2.707282
 C      0.814363   2.263949   0.007913
 C      1.216507   2.745108  -1.256992
 H      0.982705   2.172033  -2.165175
 C      1.917524   3.959861  -1.369056
 H      2.222736   4.321821  -2.363712
 C      2.222671   4.710846  -0.220126
 H      2.769368   5.663028  -0.309411
 C      1.817578   4.244086   1.044115
 H      2.044306   4.829625   1.949373
 C      1.115752   3.031535   1.157825
 H      0.789864   2.688777   2.152262
 C      0.797987  -2.546281  -0.788146
 H      1.332997  -2.149041  -1.673125
 C     -1.514106  -2.799787  -0.057555
 H     -1.646635  -3.875342  -0.329800
 H     -1.141896  -2.823246   1.002852
 C     -2.857648  -2.142529  -0.045408
 C     -4.120427  -0.272710  -0.568099
 C     -5.273573  -0.826821   0.006172
 H     -6.207557  -0.248905   0.001520
 C     -5.198453  -2.107655   0.571839
 C     -3.966163  -2.769329   0.545535
 H     -3.844874  -3.771936   0.981728
 C      0.996862  -4.073223  -0.802456
 H      2.075828  -4.316324  -0.880168
 H      0.608069  -4.568438   0.110727
 H      0.488925  -4.513964  -1.684593
 C     -1.624345   1.080970  -2.423081
 C      0.302415  -0.235621  -2.610032
 H     -6.087198  -2.570868   1.025913
 Cl-4.290040   1.330575  -1.233755
---------------------------------------
Mn12/ii
Frequencies, energies and thermodynamic properties:
 Lowest Vibrational Mode (1/cm) =                        10.6644
 2nd Lowest Vibrational Mode (1/cm) =                    19.9413
 E(RB-P86) (a.u.) =                                     -4865.82498379
 Thermal correction to Enthalpy (a.u.) =                 0.618415
 Thermal correction to Gibbs Free Energy (a.u.) =        0.495800
 Total Entropy (cal/Kmol) =                              258.065
 E(RPBE1PBE) (a.u.) =                                   -4865.12412715
Optimised cartesian coordinates (Angstrom):
 Fe3.232328   1.020744  -0.866771
 Mn-1.120151   0.398134   0.990636
 P      0.376972  -0.929928   0.086555
 O     -1.585311  -1.129649   3.489053
 O      0.718265   1.715378   2.849506
 N     -0.970042   1.740413  -0.376036
 N     -2.838696  -0.116141  -0.171200
 C      1.445385   0.032939  -1.063918
 C      1.280579   1.452350  -1.370783
 C      2.170761   1.759161  -2.464709
 H      2.305822   2.747173  -2.923102
 C      2.883903   0.565078  -2.833301
 H      3.648462   0.486199  -3.618017
 C      2.448031  -0.498420  -1.970460
 H      2.805679  -1.535707  -2.000363
 C      3.690753   1.395757   1.107212
 H      2.976511   1.380247   1.940400
 C      4.004401   2.539353   0.292386
 H      3.578751   3.545944   0.401591
 C      4.955844   2.127554  -0.708135
 H      5.378431   2.763208  -1.497785
 C      5.233205   0.727736  -0.508556
 H      5.904992   0.110222  -1.119826
 C      4.449744   0.274023   0.611355
 H      4.419088  -0.749278   1.007601
 C     -0.411497  -2.193229  -1.026376
 C     -1.028737  -3.321614  -0.435952
 H     -0.971895  -3.471885   0.653855
 C     -1.714112  -4.256077  -1.228931
 H     -2.182146  -5.133777  -0.755373
```

```
 C     -1.807337  -4.070360  -2.621768
 H     -2.346742  -4.803016  -3.242812
 C     -1.208409  -2.945707  -3.215013
 H     -1.275110  -2.792958  -4.304063
 C     -0.513406  -2.011404  -2.423704
 H     -0.034629  -1.144290  -2.903643
 C      1.553448  -1.974689   1.075836
 C      1.836029  -1.656333   2.421957
 H      1.334758  -0.807692   2.907726
 C      2.761785  -2.419800   3.156529
 H      2.969493  -2.159092   4.206414
 C      3.414667  -3.511481   2.557469
 H      4.137513  -4.109638   3.134634
 C      3.132957  -3.841549   1.218911
 H      3.632987  -4.700101   0.742965
 C      2.206452  -3.081898   0.484077
 H      1.982971  -3.364398  -0.556453
 C      0.319638   2.412126  -0.658169
 H      0.756535   2.639710   0.334360
 C     -1.784800   1.575739  -1.563564
 H     -2.189316   2.542380  -1.949767
 H     -1.190415   1.156069  -2.418734
 C     -2.926563   0.642100  -1.312278
 C     -3.850328  -1.000955   0.035778
 C     -4.951873  -1.156183  -0.818590
 C     -5.030687  -0.359207  -1.968964
 C     -3.998871   0.552333  -2.214546
 C      0.171270   3.758356  -1.391231
 H      1.135771   4.305000  -1.392108
 H     -0.149339   3.642720  -2.446833
 H     -0.575497   4.378635  -0.855288
 C     -1.481415  -0.581841   2.446586
 C      0.024942   1.173715   2.054019
 H     -1.833585   3.024310   0.697324
 O     -2.200349   3.810049   1.196473
 C     -3.600097   3.879103   0.957336
 H     -4.103861   2.909436   1.198893
 H     -3.822386   4.087524  -0.120729
 H     -4.007428   1.200205  -3.103311
 H     -5.726739  -1.893476  -0.569453
 C     -4.202990   4.985895   1.815308
 H     -5.296122   5.070771   1.644471
 H     -4.033868   4.782217   2.893640
 H     -3.737298   5.965283   1.576397
 H     -5.884362  -0.454396  -2.656561
 Cl-3.819651  -2.031345   1.441253
---------------------------------------
Mn12/iii
Frequencies, energies and thermodynamic properties:
 Lowest Vibrational Mode (1/cm) =                         17.5788
 2nd Lowest Vibrational Mode (1/cm) =                     26.0929
 E(RB-P86) (a.u.) =                                     -4866.99921193
 Thermal correction to Enthalpy (a.u.) =                  0.634645
 Thermal correction to Gibbs Free Energy (a.u.) =         0.514045
 Total Entropy (cal/Kmol) =                               253.824
 E(RPBE1PBE) (a.u.) =                                   -4866.29500378
Optimised cartesian coordinates (Angstrom):
 Fe-3.257374   1.101400   0.769846
 Mn1.150005   0.316529  -1.190418
 P     -0.381280  -0.889367  -0.008337
 O      1.222540  -1.803968  -3.252032
 O     -0.787711   1.564602  -3.003856
 N      0.989131   1.827645   0.240324
 N      2.817565  -0.163139   0.082806
 C     -1.463516   0.137619   1.050316
 C     -1.304963   1.575907   1.240452
 C     -2.199571   1.958958   2.307680
 H     -2.340286   2.977659   2.690726
 C     -2.913606   0.795468   2.762566
 H     -3.682965   0.775565   3.546275
 C     -2.474013  -0.329530   1.983650
 H     -2.830680  -1.362691   2.085166
 C     -3.741026   1.308848  -1.224600
 H     -3.044130   1.208623  -2.065914
 C     -4.020523   2.525458  -0.510102
 H     -3.579002   3.510101  -0.714418
 C     -4.962154   2.220922   0.537082
 H     -5.360279   2.931230   1.274095
 C     -5.267247   0.814175   0.466107
 H     -5.939551   0.265802   1.139583
 C     -4.510024   0.248889  -0.620285
 H     -4.504589  -0.805085  -0.926867
 C      0.420617  -2.081487   1.176162
 C      1.167663  -3.150093   0.624603
 H      1.225570  -3.275896  -0.468560
```

```
 C      1.835755  -4.060071   1.459792
 H      2.407566  -4.889632   1.014395
 C      1.778536  -3.910629   2.858773
 H      2.304065  -4.623648   3.513584
 C      1.048002  -2.846579   3.414361
 H      0.996573  -2.721525   4.507715
 C      0.371871  -1.936091   2.579340
 H     -0.205727  -1.115016   3.030598
 C     -1.567441  -2.021075  -0.895241
 C     -1.959388  -1.760142  -2.226433
 H     -1.534012  -0.908335  -2.774359
 C     -2.900282  -2.584169  -2.869977
 H     -3.191150  -2.366183  -3.909801
 C     -3.463101  -3.680986  -2.193805
 H     -4.198047  -4.326819  -2.699913
 C     -3.075831  -3.952516  -0.869069
 H     -3.505748  -4.812407  -0.331090
 C     -2.132296  -3.132303  -0.226014
 H     -1.828605  -3.368937   0.805454
 C     -0.323287   2.471000   0.479056
 H     -0.752493   2.655752  -0.527166
 C      1.629732   1.437977   1.487067
 H      1.951830   2.312054   2.104149
 H      0.946756   0.855072   2.165660
 C      2.823351   0.564897   1.244493
 C      3.884499  -0.968773  -0.133960
 C      4.959150  -1.117137   0.757421
 C      4.945810  -0.375648   1.945071
 C      3.866748   0.482610   2.182450
 C     -0.184799   3.855729   1.149871
 H     -1.155508   4.392662   1.150440
 H      0.158113   3.785358   2.203112
 H      0.545571   4.464341   0.579999
 C      1.276797  -0.984765  -2.406079
 C     -0.055944   1.045839  -2.234054
 H      2.449900   0.918325  -2.147535
 H      2.195761   1.620842  -1.777172
 H      1.875565   2.975156  -0.493126
 O      2.393356   3.662704  -1.086873
 C      3.703279   3.835399  -0.578346
 H      3.687677   4.194566   0.483486
 H      4.273079   2.869351  -0.566563
 H      3.817953   1.098498   3.092159
 H      5.784526  -1.795188   0.502676
 C      4.461265   4.847428  -1.434108
 H      3.934983   5.825454  -1.439637
 H      5.490068   5.008586  -1.049272
 H      4.535345   4.496472  -2.485243
 H      5.773874  -0.461708   2.664792
 Cl3.986005  -1.880385  -1.619597
---------------------------------------
Mn12/iv
Frequencies, energies and thermodynamic properties:
 Lowest Vibrational Mode (1/cm) =                        19.8944
 2nd Lowest Vibrational Mode (1/cm) =                    22.7773
 E(RB-P86) (a.u.) =                                    -4867.02695270
 Thermal correction to Enthalpy (a.u.) =                 0.639192
 Thermal correction to Gibbs Free Energy (a.u.) =        0.518394
 Total Entropy (cal/Kmol) =                              254.239
 E(RPBE1PBE) (a.u.) =                                  -4866.32124552
Optimised cartesian coordinates (Angstrom):
 Fe-3.299624  -1.036737  -0.789561
 Mn1.198331  -0.419308   1.010681
 P     -0.402114   0.904908   0.065757
 O      1.474962   1.302863   3.375510
 O     -0.511919  -1.970567   2.816463
 N      0.951647  -1.813403  -0.609523
 N      2.797171   0.170789  -0.275903
 C     -1.543298  -0.005396  -1.056650
 C     -1.385448  -1.411206  -1.429389
 C     -2.337499  -1.684561  -2.482374
 H     -2.491690  -2.652284  -2.975860
 C     -3.087047  -0.487731  -2.752072
 H     -3.902835  -0.390340  -3.480904
 C     -2.609376   0.544162  -1.874253
 H     -2.982398   1.575614  -1.830894
 C     -3.623781  -1.520515   1.187009
 H     -2.856960  -1.538489   1.972354
 C     -3.977011  -2.624371   0.333878
 H     -3.533663  -3.628904   0.360438
 C     -4.997616  -2.175030  -0.578253
 H     -5.463388  -2.774262  -1.372076
 C     -5.278117  -0.792624  -0.285632
 H     -5.996017  -0.153925  -0.817527
 C     -4.427795  -0.386558   0.802740
```

```
 H     -4.383332   0.614378   1.250818
 C      0.286301   2.248807  -1.030663
 C      1.071276   3.249473  -0.407309
 H      1.198465   3.242318   0.687511
 C      1.693047   4.251879  -1.169102
 H      2.293097   5.026337  -0.664939
 C      1.557326   4.263522  -2.571015
 H      2.048159   5.047698  -3.169055
 C      0.794481   3.265803  -3.201026
 H      0.682850   3.264063  -4.297253
 C      0.161627   2.265319  -2.437225
 H     -0.443736   1.500698  -2.948159
 C     -1.558388   1.895560   1.142928
 C     -1.830506   1.473264   2.462619
 H     -1.338678   0.576604   2.865317
 C     -2.732220   2.188595   3.271594
 H     -2.931492   1.845022   4.299225
 C     -3.372435   3.338110   2.775068
 H     -4.075903   3.899413   3.410456
 C     -3.102628   3.771443   1.463960
 H     -3.593756   4.674394   1.067080
 C     -2.199704   3.058319   0.655637
 H     -1.986571   3.419804  -0.362597
 C     -0.408168  -2.415566  -0.832449
 H     -0.757802  -2.678488   0.185323
 C      1.556787  -1.254048  -1.829266
 H      1.799782  -2.029097  -2.588279
 H      0.819095  -0.568334  -2.303333
 C      2.788972  -0.460311  -1.496896
 C      3.879615   0.945303  -0.021410
 C      4.959578   1.123794  -0.905159
 C      4.939315   0.458030  -2.133852
 C      3.832021  -0.351267  -2.426621
 C     -0.325784  -3.721346  -1.641146
 H     -1.305607  -4.239028  -1.638761
 H     -0.035957  -3.551386  -2.698212
 H      0.413066  -4.405544  -1.176631
 C      1.427458   0.653644   2.386184
 C      0.132857  -1.325986   2.055023
 H      2.348193  -1.368133   1.603073
 H      2.479541  -2.806735   1.344641
 H      1.557897  -2.593882  -0.274606
 O      2.558478  -3.706276   0.896045
 C      3.948624  -3.961620   0.672850
 H      4.002755  -4.795923  -0.060075
 H      4.444798  -3.082950   0.194636
 H      3.763509  -0.900223  -3.377094
 H      5.795075   1.772051  -0.608524
 C      4.686032  -4.343970   1.955759
 H      4.212224  -5.227307   2.432449
 H      5.747633  -4.591423   1.742743
 H      4.672479  -3.509214   2.688385
 H      5.771532   0.566687  -2.845157
 Cl3.998144   1.786920   1.501976
---------------------------------------
Mn12/v
Frequencies, energies and thermodynamic properties:
 Lowest Vibrational Mode (1/cm) =                         19.3232
 2nd Lowest Vibrational Mode (1/cm) =                     29.7810
 E(RB-P86) (a.u.) =                                     -4712.08834221
 Thermal correction to Enthalpy (a.u.) =                  0.554601
 Thermal correction to Gibbs Free Energy (a.u.) =         0.447661
 Total Entropy (cal/Kmol) =                               225.075
 E(RPBE1PBE) (a.u.) =                                   -4711.37843014
Optimised cartesian coordinates (Angstrom):
 Fe3.226610  -0.790352   0.530059
 Mn-1.141691  -0.347210  -1.581481
 P     -0.068225   0.606112   0.188435
 O     -1.811385   2.204269  -2.873500
 O      1.016900  -0.321777  -3.562105
 N     -0.578916  -2.288709  -0.851618
 N     -2.913230  -0.906223  -0.543372
 C      1.232949  -0.477552   0.917801
 C      1.478369  -1.863090   0.514641
 C      2.414003  -2.426698   1.462449
 H      2.821769  -3.444965   1.437973
 C      2.762786  -1.417725   2.424912
 H      3.475632  -1.537158   3.251810
 C      2.047231  -0.217930   2.090893
 H      2.102218   0.735118   2.632866
 C      3.778810  -0.086659  -1.325830
 H      3.092042   0.128999  -2.154817
 C      4.402820  -1.356562  -1.062426
 H      4.283055  -2.271255  -1.658628
 C      5.195512  -1.225294   0.133339
```

```
 H      5.778841  -2.023272   0.612015
 C      5.064482   0.128822   0.607108
 H      5.531032   0.543670   1.510676
 C      4.187220   0.832106  -0.291911
 H      3.868227   1.878105  -0.199097
 C     -1.168756   0.971860   1.651545
 C     -2.183863   1.942002   1.465261
 H     -2.251849   2.484694   0.508290
 C     -3.109443   2.218243   2.484422
 H     -3.886179   2.982908   2.322834
 C     -3.051415   1.516061   3.704121
 H     -3.779838   1.729734   4.502422
 C     -2.060432   0.537912   3.892691
 H     -2.006998  -0.019760   4.841617
 C     -1.124327   0.267628   2.875034
 H     -0.346737  -0.492457   3.047959
 C      0.801811   2.244137  -0.010005
 C      1.253064   2.647571  -1.285794
 H      1.066877   2.007485  -2.160055
 C      1.944907   3.861518  -1.449573
 H      2.289300   4.160787  -2.452429
 C      2.192107   4.691956  -0.341639
 H      2.731115   5.643982  -0.471320
 C      1.739496   4.302860   0.932527
 H      1.922187   4.949190   1.806028
 C      1.046333   3.090309   1.096543
 H      0.683891   2.807016   2.097264
 C      0.883472  -2.598148  -0.680144
 H      1.351010  -2.202593  -1.603406
 C     -1.403500  -2.626518   0.320979
 H     -1.445190  -3.718130   0.524646
 H     -0.934156  -2.153024   1.212231
 C     -2.794779  -2.077141   0.169475
 C     -4.160647  -0.376763  -0.591379
 C     -5.297729  -0.951370   0.005958
 H     -6.268144  -0.446329  -0.092692
 C     -5.157772  -2.155311   0.701301
 C     -3.877436  -2.724116   0.778272
 H     -3.705604  -3.669011   1.314250
 C      1.149246  -4.111936  -0.637028
 H      2.237463  -4.311635  -0.696691
 H      0.769384  -4.586004   0.290802
 H      0.675677  -4.611876  -1.507003
 C     -1.616465   1.182220  -2.305231
 C      0.183389  -0.320232  -2.714688
 H     -0.905525  -2.848253  -1.651494
 H     -1.834466  -1.150228  -2.764205
 H     -6.027355  -2.637742   1.171787
 Cl-4.430685   1.116551  -1.452745
---------------------------------------
Mn12/vi_R
Frequencies, energies and thermodynamic properties:
 Lowest Vibrational Mode (1/cm) =                         12.6956
 2nd Lowest Vibrational Mode (1/cm) =                     21.2000
 E(RB-P86) (a.u.) =                                     -5134.80024346
 Thermal correction to Enthalpy (a.u.) =                  0.706981
 Thermal correction to Gibbs Free Energy (a.u.) =         0.577941
 Total Entropy (cal/Kmol) =                               271.587
 E(RPBE1PBE) (a.u.) =                                   -5134.08617046
Optimised cartesian coordinates (Angstrom):
 Fe-3.409702  -1.716160  -0.952907
 Mn0.867250   0.109225   0.231539
 P     -1.230671   0.746519   0.477257
 O      1.581256   0.440381   3.086601
 O      0.453573  -2.621067   1.210418
 N      0.612654  -0.351965  -1.662483
 N      1.663393   1.880626  -0.669024
 C     -2.298746   0.004395  -0.819978
 C     -1.817354  -0.831457  -1.916934
 C     -2.924218  -1.020399  -2.824790
 H     -2.904298  -1.616842  -3.745753
 C     -4.075352  -0.330776  -2.306068
 H     -5.074654  -0.310600  -2.761519
 C     -3.700209   0.294124  -1.067507
 H     -4.357509   0.894998  -0.425901
 C     -2.874981  -3.099628   0.478719
 H     -1.937287  -3.098209   1.048690
 C     -3.083632  -3.744200  -0.790468
 H     -2.337120  -4.322605  -1.351243
 C     -4.435796  -3.476866  -1.209932
 H     -4.898988  -3.811238  -2.147923
 C     -5.064331  -2.668371  -0.196217
 H     -6.090979  -2.279420  -0.226909
 C     -4.099410  -2.432024   0.846164
 H     -4.259574  -1.835003   1.753310
```

```
 C     -1.419333   2.576134   0.203127
 C     -0.947691   3.451123   1.211041
 H     -0.540606   3.040762   2.149016
 C     -0.995058   4.842205   1.027006
 H     -0.631395   5.510260   1.823887
 C     -1.500679   5.381097  -0.172000
 H     -1.535842   6.472551  -0.316463
 C     -1.959969   4.519419  -1.182577
 H     -2.358427   4.932188  -2.123102
 C     -1.921213   3.123862  -0.998002
 H     -2.300635   2.461975  -1.791328
 C     -2.175610   0.508229   2.061122
 C     -1.795039  -0.489260   2.984855
 H     -0.910370  -1.112339   2.795410
 C     -2.543045  -0.698710   4.157768
 H     -2.230483  -1.479727   4.868852
 C     -3.679274   0.085533   4.423989
 H     -4.262590  -0.078274   5.343938
 C     -4.062268   1.086765   3.512712
 H     -4.946586   1.711675   3.715437
 C     -3.314233   1.300161   2.341955
 H     -3.616762   2.099646   1.647794
 C     -0.389856  -1.368482  -2.067435
 H     -0.266677  -2.201601  -1.347470
 C      0.665081   0.767716  -2.590807
 H      1.098697   0.488990  -3.580734
 H     -0.360668   1.157570  -2.824342
 C      1.459166   1.903558  -2.025888
 C      2.362966   2.924391  -0.151862
 C      2.865671   3.992763  -0.909415
 H      3.421847   4.795317  -0.406935
 C      2.647561   3.996589  -2.293615
 C      1.938653   2.930541  -2.855930
 H      1.745601   2.879990  -3.937505
 C     -0.139404  -1.958570  -3.469024
 H     -0.801805  -2.830808  -3.641179
 H     -0.329747  -1.229090  -4.282705
 H      0.908097  -2.312636  -3.550150
 C      1.342663   0.406646   1.929669
 C      0.560472  -1.516202   0.794358
 H      2.073202  -1.052448  -1.888796
 H      2.875389  -0.591477  -0.048818
 C      3.656775  -1.062209  -0.757549
 C      4.289265  -2.208497   0.019480
 C      4.832807  -0.052076  -0.880622
 C      5.466061  -1.772500   0.668017
 C      3.849546  -3.535629   0.134085
 C      5.717067  -0.304475   0.364410
 H      5.386778  -0.321971  -1.806610
 C      6.211786  -2.668783   1.451992
 C      4.599909  -4.434985   0.917536
 H      2.936347  -3.862792  -0.388068
 H      6.790103  -0.068943   0.204405
 C      5.771455  -4.003172   1.573017
 H      7.135276  -2.341057   1.957845
 H      4.274325  -5.483430   1.015189
 H      6.352852  -4.717349   2.178536
 O      3.040403  -1.411902  -1.955359
 H      4.493874   0.997953  -0.985082
 H      5.377911   0.326897   1.217819
 H      3.032499   4.817785  -2.916667
 Cl2.695639   2.976075   1.559069
---------------------------------------
Mn12/vi_S
Frequencies, energies and thermodynamic properties:
 Lowest Vibrational Mode (1/cm) =                         16.1167
 2nd Lowest Vibrational Mode (1/cm) =                     18.7112
 E(RB-P86) (a.u.) =                                     -5134.80052437
 Thermal correction to Enthalpy (a.u.) =                  0.707106
 Thermal correction to Gibbs Free Energy (a.u.) =         0.578387
 Total Entropy (cal/Kmol) =                               270.912
 E(RPBE1PBE) (a.u.) =                                   -5134.08667151
Optimised cartesian coordinates (Angstrom):
 Fe-3.786168  -1.366261  -0.838790
 Mn0.694696  -0.223513   0.448889
 P     -1.219784   0.864955   0.308898
 O      1.264726   0.578673   3.241889
 O     -0.383534  -2.522390   1.909127
 N      0.480732  -1.033371  -1.329139
 N      1.935343   1.099065  -0.690917
 C     -2.331184   0.079032  -0.924526
 C     -1.969629  -1.060155  -1.764207
 C     -3.021147  -1.219521  -2.740661
 H     -3.067627  -1.998431  -3.512269
 C     -4.024015  -0.213205  -2.514041
```

```
 H     -4.957482  -0.093631  -3.080426
 C     -3.610496   0.583581  -1.391857
 H     -4.162699   1.432708  -0.968840
 C     -3.678821  -2.495428   0.883933
 H     -2.804741  -2.577313   1.542583
 C     -3.954983  -3.334604  -0.251520
 H     -3.332446  -4.167311  -0.605556
 C     -5.178440  -2.875935  -0.858560
 H     -5.648749  -3.293584  -1.758857
 C     -5.660603  -1.753553  -0.094354
 H     -6.563413  -1.166700  -0.310594
 C     -4.732768  -1.515642   0.980696
 H     -4.802998  -0.718571   1.732153
 C     -0.975536   2.584008  -0.356466
 C     -0.387573   3.548081   0.497198
 H     -0.145006   3.284513   1.539113
 C     -0.109373   4.841429   0.027163
 H      0.341488   5.582906   0.705759
 C     -0.400438   5.188016  -1.306592
 H     -0.180024   6.202366  -1.675245
 C     -0.973340   4.233537  -2.164130
 H     -1.205630   4.496289  -3.208507
 C     -1.261114   2.937814  -1.693593
 H     -1.725871   2.206216  -2.371884
 C     -2.302681   1.187021   1.785514
 C     -2.220751   0.367578   2.932238
 H     -1.486598  -0.448218   2.979882
 C     -3.077033   0.584618   4.027129
 H     -2.998002  -0.062517   4.914998
 C     -4.024416   1.622999   3.991848
 H     -4.692497   1.793113   4.850987
 C     -4.108512   2.449375   2.856270
 H     -4.842116   3.270499   2.821560
 C     -3.251632   2.236441   1.762596
 H     -3.317464   2.903209   0.888744
 C     -0.690094  -1.892988  -1.632284
 H     -0.806080  -2.548857  -0.746774
 C      0.842844  -0.178926  -2.450700
 H      1.255751  -0.750355  -3.315510
 H     -0.050194   0.362165  -2.861437
 C      1.842120   0.855887  -2.038205
 C      2.813199   2.065778  -0.316397
 C      3.604321   2.804446  -1.209169
 H      4.289669   3.567826  -0.817464
 C      3.498827   2.536886  -2.580337
 C      2.606721   1.543072  -2.995467
 H      2.487327   1.290399  -4.059132
 C     -0.476698  -2.823091  -2.842499
 H     -1.301365  -3.561195  -2.908513
 H     -0.441765  -2.274411  -3.805946
 H      0.470018  -3.387852  -2.723501
 C      1.104671   0.340992   2.095338
 C     -0.008020  -1.582112   1.290974
 H      1.785619  -2.019483  -1.263627
 H      2.549133  -1.325765   0.523111
 C      3.184714  -2.180195   0.076291
 C      3.194835  -3.329520   1.124409
 C      4.304987  -2.945303   2.133177
 O      2.692281  -2.509323  -1.183407
 H      4.108815   3.095306  -3.306161
 C      4.627322  -1.697755   0.103024
 C      5.315036  -0.940438  -0.857108
 C      5.267632  -2.128795   1.286357
 C      6.662753  -0.603690  -0.619678
 H      4.807624  -0.632363  -1.785658
 C      6.610085  -1.789411   1.523972
 C      7.303736  -1.023056   0.564199
 H      7.222546  -0.016040  -1.365334
 H      7.122289  -2.125807   2.440788
 H      8.359755  -0.758571   0.736151
 H      2.202974  -3.498966   1.587787
 H      3.889439  -2.315269   2.953330
 H      3.479437  -4.255402   0.577538
 H      4.786416  -3.818830   2.620667
 Cl3.006873   2.446181   1.373448
---------------------------------------
Mn12/viii
Frequencies, energies and thermodynamic properties:
 Lowest Vibrational Mode (1/cm) =                          17.3371
 2nd Lowest Vibrational Mode (1/cm) =                      30.0101
 E(RB-P86) (a.u.) =                                     -4712.05521973
 Thermal correction to Enthalpy (a.u.) =                   0.549810
 Thermal correction to Gibbs Free Energy (a.u.) =          0.442265
 Total Entropy (cal/Kmol) =                                226.348
 E(RPBE1PBE) (a.u.) =                                   -4711.34585202
```

```
Optimised cartesian coordinates (Angstrom):
 Fe3.247006  -0.785143   0.522376
 Mn-1.108796  -0.327398  -1.641347
 P     -0.064797   0.547934   0.170289
 O     -1.746554   2.377412  -2.667881
 O      1.179175  -0.070667  -3.455300
 N     -0.562851  -2.185562  -0.965789
 N     -2.900872  -0.899531  -0.586051
 C      1.233692  -0.538359   0.869014
 C      1.506573  -1.894282   0.405819
 C      2.431571  -2.481445   1.346133
 H      2.856732  -3.491335   1.280528
 C      2.743524  -1.513288   2.365084
 H      3.437552  -1.658438   3.204179
 C      2.017296  -0.307983   2.070552
 H      2.043097   0.618878   2.658329
 C      3.849961   0.037861  -1.270966
 H      3.189558   0.308682  -2.104328
 C      4.465336  -1.247185  -1.074380
 H      4.359362  -2.120998  -1.731252
 C      5.223136  -1.196226   0.150331
 H      5.793367  -2.024637   0.591800
 C      5.078398   0.124703   0.708416
 H      5.520259   0.478726   1.649597
 C      4.227048   0.887007  -0.167572
 H      3.906236   1.925852  -0.016532
 C     -1.233713   0.818118   1.594821
 C     -2.230431   1.812500   1.448094
 H     -2.269862   2.421242   0.530472
 C     -3.172551   2.035097   2.465420
 H     -3.938097   2.816945   2.337789
 C     -3.141523   1.260149   3.640778
 H     -3.881373   1.434101   4.438168
 C     -2.162060   0.263595   3.790107
 H     -2.129434  -0.347589   4.706206
 C     -1.212587   0.042228   2.773703
 H     -0.444972  -0.734718   2.909394
 C      0.756523   2.219063   0.071756
 C      1.249082   2.709535  -1.157326
 H      1.117080   2.128025  -2.079963
 C      1.917428   3.945858  -1.218477
 H      2.292288   4.313038  -2.187057
 C      2.102675   4.710915  -0.053418
 H      2.623896   5.680100  -0.103275
 C      1.611082   4.233970   1.175303
 H      1.745037   4.827500   2.093755
 C      0.939529   3.000402   1.237065
 H      0.546307   2.649826   2.203882
 C      0.849545  -2.542587  -0.822255
 H      1.364345  -2.138252  -1.718587
 C     -1.364475  -2.653489   0.134713
 H     -1.474045  -3.768989   0.161672
 H     -0.942438  -2.405195   1.156322
 C     -2.742149  -2.064858   0.118813
 C     -4.142811  -0.356571  -0.587096
 C     -5.245315  -0.896806   0.091818
 C     -5.065694  -2.083463   0.815902
 C     -3.798309  -2.673862   0.819805
 C      1.091252  -4.069203  -0.842963
 H      2.173592  -4.299604  -0.926771
 H      0.714149  -4.572560   0.071702
 H      0.576362  -4.512788  -1.719476
 C     -1.588870   1.287196  -2.243416
 C      0.297347  -0.167635  -2.671734
 H     -1.688278  -1.535447  -2.779992
 H     -2.101917  -0.870001  -3.022529
 H     -6.218074  -0.390306   0.035394
 H     -3.607684  -3.611994   1.361499
 H     -5.908649  -2.538556   1.357562
 Cl-4.443685   1.102006  -1.500921
--------------------------------------
Mn12/ix
Frequencies, energies and thermodynamic properties:
 Lowest Vibrational Mode (1/cm) =                        23.7217
 2nd Lowest Vibrational Mode (1/cm) =                    31.3294
 E(RB-P86) (a.u.) =                                     -4865.84387044
 Thermal correction to Enthalpy (a.u.) =                 0.619098
 Thermal correction to Gibbs Free Energy (a.u.) =        0.502107
 Total Entropy (cal/Kmol) =                              246.228
 E(RPBE1PBE) (a.u.) =                                   -4865.13841207
Optimised cartesian coordinates (Angstrom):
 Fe-3.368163  -0.652994  -0.824975
 Mn1.176105  -0.769920   0.934890
 P     -0.209002   0.806735   0.082771
 O      1.798007   0.796813   3.346967
```

```
 O     -0.842787  -1.949891   2.694147
 N      0.703593  -2.041635  -0.704482
 N      2.811901  -0.285802  -0.458165
 C     -1.467305   0.090953  -1.064132
 C     -1.534610  -1.310749  -1.478702
 C     -2.515648  -1.399795  -2.535985
 H     -2.819106  -2.317147  -3.055939
 C     -3.063256  -0.091756  -2.771377
 H     -3.850110   0.156113  -3.496384
 C     -2.429699   0.825376  -1.865500
 H     -2.633566   1.901524  -1.796531
 C     -3.780568  -1.110689   1.141866
 H     -3.031924  -1.257309   1.930926
 C     -4.290598  -2.134649   0.268729
 H     -4.006480  -3.195554   0.282437
 C     -5.221624  -1.520773  -0.643297
 H     -5.766607  -2.029612  -1.449682
 C     -5.289028  -0.115836  -0.330970
 H     -5.894819   0.633256  -0.858302
 C     -4.396815   0.138756   0.770068
 H     -4.202447   1.114545   1.233760
 C      0.640321   2.072669  -0.994161
 C      1.453280   3.041150  -0.357962
 H      1.511558   3.070796   0.741877
 C      2.187774   3.969030  -1.114010
 H      2.809173   4.720190  -0.600653
 C      2.135259   3.936768  -2.520861
 H      2.713295   4.663149  -3.113939
 C      1.340061   2.971988  -3.162664
 H      1.290176   2.938707  -4.262875
 C      0.596072   2.046554  -2.405413
 H     -0.035265   1.309963  -2.925938
 C     -1.213290   1.920176   1.193034
 C     -1.499712   1.535732   2.521213
 H     -1.107641   0.590482   2.920116
 C     -2.291505   2.352702   3.349149
 H     -2.502358   2.035836   4.382966
 C     -2.806897   3.567400   2.863846
 H     -3.424571   4.207396   3.513798
 C     -2.520902   3.964288   1.544561
 H     -2.912920   4.917678   1.155613
 C     -1.727522   3.149973   0.717837
 H     -1.497779   3.485438  -0.305421
 C     -0.708987  -2.467056  -0.925209
 H     -1.084355  -2.712906   0.088165
 C      1.420087  -1.670182  -1.929968
 H      1.599804  -2.536345  -2.603364
 H      0.786139  -0.956242  -2.503793
 C      2.729496  -0.991581  -1.625560
 C      3.962919   0.380299  -0.236471
 C      5.059550   0.389245  -1.117030
 C      4.965316  -0.349528  -2.300859
 C      3.780972  -1.053156  -2.554610
 C     -0.820521  -3.742087  -1.778254
 H     -1.861792  -4.121443  -1.766427
 H     -0.535583  -3.573799  -2.836723
 H     -0.170713  -4.538587  -1.361751
 C      1.622757   0.194110   2.344215
 C     -0.058810  -1.450342   1.955345
 H      1.251641  -2.768849  -0.179927
 H      3.659540  -1.650913  -3.469739
 H      5.960174   0.961731  -0.857437
 H      5.805777  -0.376394  -3.010734
 O      2.387906  -2.356526   1.172809
 C      2.641999  -2.942695   2.413845
 C      3.602691  -4.130810   2.266009
 H      1.705761  -3.308916   2.921023
 H      3.094776  -2.209038   3.136351
 H      3.820900  -4.598841   3.250335
 H      3.167038  -4.909370   1.603157
 H      4.565162  -3.804933   1.816359
 Cl4.149456   1.309421   1.230648
---------------------------------------
Mn12/x
Frequencies, energies and thermodynamic properties:
 Lowest Vibrational Mode (1/cm) =                         15.7770
 2nd Lowest Vibrational Mode (1/cm) =                     24.3616
 E(RB-P86) (a.u.) =                                     -5020.78503232
 Thermal correction to Enthalpy (a.u.) =                  0.703336
 Thermal correction to Gibbs Free Energy (a.u.) =         0.573627
 Total Entropy (cal/Kmol) =                               272.995
 E(RPBE1PBE) (a.u.) =                                   -5020.08925169
Optimised cartesian coordinates (Angstrom):
 Fe-3.367043  -1.416061  -0.710766
 Mn1.085548  -0.097971   0.884176
```

```
 P     -0.750349   0.932492   0.027050
 O      1.038958   1.868601   3.072841
 O     -0.384749  -1.814015   2.747471
 N      0.958315  -1.528844  -0.722174
 N      2.476462   0.754991  -0.565930
 C     -1.790103  -0.141907  -1.044155
 C     -1.444912  -1.509460  -1.424857
 C     -2.385846  -1.922803  -2.440620
 H     -2.415191  -2.904881  -2.928965
 C     -3.311472  -0.848465  -2.678207
 H     -4.160386  -0.872666  -3.374578
 C     -2.956879   0.245163  -1.816480
 H     -3.473708   1.211647  -1.757476
 C     -3.558589  -1.918808   1.278648
 H     -2.779137  -1.804629   2.042856
 C     -3.752813  -3.076292   0.446288
 H     -3.154479  -3.997013   0.470759
 C     -4.854536  -2.807296  -0.442068
 H     -5.238265  -3.483383  -1.217804
 C     -5.344353  -1.483190  -0.154973
 H     -6.167754  -0.974081  -0.673612
 C     -4.542321  -0.932200   0.906062
 H     -4.647947   0.068351   1.344909
 C     -0.318913   2.359972  -1.094652
 C      0.282083   3.496646  -0.501522
 H      0.420165   3.537759   0.590849
 C      0.703143   4.578195  -1.291724
 H      1.162538   5.457077  -0.811827
 C      0.544543   4.538067  -2.690542
 H      0.877815   5.385382  -3.310546
 C     -0.041708   3.410373  -3.289637
 H     -0.172522   3.369283  -4.382855
 C     -0.472952   2.328133  -2.497768
 H     -0.946299   1.461254  -2.984019
 C     -1.998630   1.743108   1.151783
 C     -2.172255   1.290850   2.478085
 H     -1.545028   0.480349   2.873425
 C     -3.150266   1.866768   3.309133
 H     -3.268947   1.501523   4.341624
 C     -3.968587   2.904314   2.828437
 H     -4.732446   3.356141   3.481030
 C     -3.799841   3.366713   1.510623
 H     -4.430927   4.183283   1.124906
 C     -2.820043   2.794197   0.680524
 H     -2.690137   3.179838  -0.342612
 C     -0.298678  -2.341174  -0.869707
 H     -0.554393  -2.640721   0.165888
 C      1.401570  -0.916970  -1.987250
 H      1.729051  -1.669481  -2.736758
 H      0.541799  -0.376891  -2.443540
 C      2.506671   0.074531  -1.750969
 C      3.447620   1.670023  -0.370426
 C      4.453737   1.972044  -1.305871
 C      4.470667   1.268220  -2.514127
 C      3.484371   0.297650  -2.733768
 C     -0.054884  -3.632500  -1.669535
 H     -0.942510  -4.293648  -1.612036
 H      0.151249  -3.437755  -2.741947
 H      0.805839  -4.178157  -1.233868
 C      1.132949   1.107638   2.172513
 C      0.165749  -1.104444   1.973384
 H      2.945189  -2.402387   0.837551
 H      1.698455  -2.219271  -0.424983
 O      2.905987  -3.302780   0.284827
 C      4.113738  -3.452580  -0.453056
 H      3.879192  -3.541271  -1.542560
 H      4.752133  -2.543453  -0.343603
 H      3.462280  -0.292004  -3.661643
 H      5.202993   2.739161  -1.068458
 C      4.897785  -4.691222  -0.014888
 H      4.274560  -5.604693  -0.118171
 H      5.810288  -4.826390  -0.634381
 H      5.210884  -4.613426   1.047792
 H      5.247697   1.469613  -3.266881
 O      2.835325  -1.050550   1.393062
 C      3.269789  -1.021775   2.733057
 C      4.684927  -1.593511   2.878173
 H      2.580635  -1.592768   3.413454
 H      3.271155   0.027020   3.120934
 H      5.024266  -1.540604   3.935052
 H      4.724987  -2.657221   2.562168
 H      5.406646  -1.023096   2.255493
 Cl3.498103   2.567092   1.126229
--------------------------------------
Mn12/TS-i
```

```
Frequencies, energies and thermodynamic properties:
 Lowest Vibrational Mode (1/cm) =-773.0377
 2nd Lowest Vibrational Mode (1/cm) =                     18.0879
 E(RB-P86) (a.u.) =                                      -4866.99672177
 Thermal correction to Enthalpy (a.u.) =                  0.630790
 Thermal correction to Gibbs Free Energy (a.u.) =         0.511421
 Total Entropy (cal/Kmol) =                               251.233
 E(RPBE1PBE) (a.u.) =                                    -4866.28975334
Optimised cartesian coordinates (Angstrom):
 Fe-3.276601   1.030899   0.781853
 Mn1.157434   0.373040  -1.157586
 P     -0.358037  -0.889736  -0.017788
 O      1.313001  -1.672412  -3.281289
 O     -0.783624   1.611717  -2.974251
 N      0.948302   1.867931   0.306789
 N      2.821964  -0.088136   0.125648
 C     -1.467262   0.097291   1.056257
 C     -1.341608   1.535518   1.277238
 C     -2.250632   1.879780   2.345560
 H     -2.415013   2.887554   2.747475
 C     -2.942059   0.692672   2.772128
 H     -3.716187   0.641472   3.549589
 C     -2.472894  -0.407135   1.975090
 H     -2.808512  -1.449211   2.054316
 C     -3.745984   1.263956  -1.212518
 H     -3.038761   1.193452  -2.048311
 C     -4.058488   2.461308  -0.479091
 H     -3.636521   3.458774  -0.661420
 C     -5.003478   2.118082   0.552834
 H     -5.423593   2.806324   1.298493
 C     -5.277522   0.706812   0.453831
 H     -5.943903   0.132107   1.111001
 C     -4.497955   0.177357  -0.634799
 H     -4.466912  -0.870694  -0.959641
 C      0.453868  -2.088396   1.153465
 C      1.220933  -3.136018   0.589025
 H      1.282354  -3.245861  -0.505682
 C      1.904846  -4.044474   1.412862
 H      2.491779  -4.857766   0.957215
 C      1.844779  -3.914001   2.813675
 H      2.383040  -4.625655   3.459559
 C      1.095076  -2.870455   3.382322
 H      1.041196  -2.760107   4.477145
 C      0.402329  -1.962122   2.558520
 H     -0.191279  -1.158656   3.020756
 C     -1.517856  -2.023635  -0.935132
 C     -1.894240  -1.747464  -2.267744
 H     -1.474238  -0.880377  -2.795449
 C     -2.812956  -2.575440  -2.937876
 H     -3.092325  -2.345385  -3.978259
 C     -3.368141  -3.691494  -2.287404
 H     -4.085527  -4.340399  -2.814357
 C     -2.995627  -3.978478  -0.961606
 H     -3.419557  -4.853647  -0.443882
 C     -2.074535  -3.154170  -0.291884
 H     -1.781357  -3.402977   0.739818
 C     -0.388891   2.475690   0.541687
 H     -0.806085   2.657412  -0.469472
 C      1.600525   1.472123   1.553155
 H      1.891311   2.342970   2.185891
 H      0.916292   0.860947   2.195935
 C      2.817733   0.635457   1.290495
 C      3.905665  -0.869793  -0.095915
 C      4.989323  -0.991838   0.789645
 C      4.967811  -0.249617   1.975733
 C      3.868522   0.581267   2.220928
 C     -0.286483   3.853399   1.227453
 H     -1.269318   4.366887   1.223587
 H      0.049758   3.780962   2.282374
 H      0.431749   4.486443   0.668670
 C      1.331274  -0.883838  -2.404858
 C     -0.047365   1.096863  -2.205140
 H      2.425388   1.090273  -2.039205
 H      2.205609   1.853704  -1.697163
 H      1.675269   2.761810  -0.327147
 O      2.285054   3.443361  -1.115041
 C      3.609562   3.706820  -0.718002
 H      3.666617   3.981105   0.369147
 H      4.265986   2.799538  -0.829748
 H      3.809930   1.194756   3.131617
 H      5.827903  -1.651673   0.530222
 C      4.214405   4.845332  -1.544367
 H      3.614117   5.772708  -1.427190
 H      5.258399   5.064460  -1.232926
 H      4.223554   4.583924  -2.624106
```

```
 H      5.802927  -0.314505   2.689406
 Cl4.019346  -1.784238  -1.577397
---------------------------------------
Mn12/TS-ii_si
Frequencies, energies and thermodynamic properties:
 Lowest Vibrational Mode (1/cm) =-260.4266
 2nd Lowest Vibrational Mode (1/cm) =                     14.1193
 E(RB-P86) (a.u.) =                                      -5134.79534404
 Thermal correction to Enthalpy (a.u.) =                  0.703817
 Thermal correction to Gibbs Free Energy (a.u.) =         0.577572
 Total Entropy (cal/Kmol) =                               265.704
 E(RPBE1PBE) (a.u.) =                                    -5134.07566068
Optimised cartesian coordinates (Angstrom):
 Fe-3.478130  -1.480316  -0.925483
 Mn1.004472   0.006016   0.208723
 P     -1.096819   0.813766   0.475004
 O      1.776945   0.213520   3.039369
 O      0.457608  -2.750620   1.037295
 N      0.614599  -0.406174  -1.815908
 N      1.805865   1.760038  -0.686608
 C     -2.261677   0.168882  -0.797461
 C     -1.874087  -0.676691  -1.926625
 C     -3.021975  -0.777047  -2.799310
 H     -3.073505  -1.354654  -3.730690
 C     -4.107462  -0.028264  -2.226832
 H     -5.118982   0.060606  -2.645286
 C     -3.649000   0.548495  -0.993831
 H     -4.243838   1.175430  -0.317190
 C     -2.968290  -2.932388   0.444996
 H     -2.005012  -3.006742   0.965873
 C     -3.281767  -3.528130  -0.826928
 H     -2.604229  -4.139279  -1.438308
 C     -4.632249  -3.162703  -1.170485
 H     -5.162136  -3.440932  -2.091352
 C     -5.154975  -2.342553  -0.107607
 H     -6.153641  -1.886551  -0.076972
 C     -4.126567  -2.197267   0.889473
 H     -4.201630  -1.614433   1.816525
 C     -1.227618   2.661462   0.262407
 C     -0.647617   3.477519   1.263752
 H     -0.202884   3.013919   2.159185
 C     -0.632746   4.875252   1.129678
 H     -0.184136   5.494043   1.923250
 C     -1.182808   5.483305  -0.015386
 H     -1.168620   6.579633  -0.121777
 C     -1.748437   4.681777  -1.021420
 H     -2.181994   5.146848  -1.921300
 C     -1.772374   3.280071  -0.884408
 H     -2.235479   2.669976  -1.675262
 C     -2.018258   0.578698   2.077767
 C     -1.668743  -0.476778   2.947866
 H     -0.831777  -1.142927   2.696350
 C     -2.385898  -0.689276   4.139499
 H     -2.098120  -1.517060   4.807012
 C     -3.460370   0.151571   4.479928
 H     -4.019525  -0.013811   5.414576
 C     -3.811900   1.210931   3.623152
 H     -4.647660   1.879895   3.883752
 C     -3.094263   1.425560   2.433389
 H     -3.370700   2.269777   1.782535
 C     -0.517938  -1.331646  -2.160590
 H     -0.408388  -2.171478  -1.446864
 C      0.612384   0.833132  -2.609559
 H      0.845628   0.656860  -3.681930
 H     -0.409425   1.275279  -2.577450
 C      1.572819   1.836742  -2.039392
 C      2.600320   2.731595  -0.170785
 C      3.188547   3.764363  -0.921692
 H      3.819227   4.506076  -0.413575
 C      2.957832   3.808159  -2.300155
 C      2.136881   2.821586  -2.862846
 H      1.919756   2.808013  -3.940807
 C     -0.374052  -1.915063  -3.576669
 H     -1.109161  -2.729540  -3.732720
 H     -0.537035  -1.157296  -4.370068
 H      0.638403  -2.348783  -3.703336
 C      1.492466   0.224724   1.890553
 C      0.625198  -1.628150   0.690895
 H      1.510643  -0.921169  -2.058748
 H      2.500481  -0.635440  -0.106542
 C      3.623337  -1.345630  -1.131561
 C      3.954995  -2.439199  -0.150050
 C      4.702797  -0.260030  -0.895051
 C      4.909039  -1.973786   0.782722
 C      3.495298  -3.765981  -0.130260
```

```
 C      5.230958  -0.510689   0.533630
 H      5.499971  -0.466420  -1.646023
 C      5.410780  -2.846706   1.762272
 C      4.002143  -4.638231   0.849529
 H      2.759983  -4.106962  -0.876338
 H      6.309769  -0.281689   0.656619
 C      4.951053  -4.179350   1.789230
 H      6.158917  -2.502454   2.495361
 H      3.663957  -5.686443   0.883995
 H      5.342850  -4.874845   2.549087
 O      3.033934  -1.525205  -2.235742
 H      4.335846   0.766112  -1.083904
 H      4.681874   0.125365   1.264650
 H      3.411005   4.596013  -2.920067
 Cl2.951507   2.742247   1.538611
---------------------------------------
Mn12/TS-ii_re
Frequencies, energies and thermodynamic properties:
 Lowest Vibrational Mode (1/cm) =-239.9312
 2nd Lowest Vibrational Mode (1/cm) =                     18.8019
 E(RB-P86) (a.u.) =                                     -5134.79596793
 Thermal correction to Enthalpy (a.u.) =                 0.703851
 Thermal correction to Gibbs Free Energy (a.u.) =        0.577732
 Total Entropy (cal/Kmol) =                              265.440
 E(RPBE1PBE) (a.u.) =                                   -5134.07832499
Optimised cartesian coordinates (Angstrom):
 Fe-3.860437  -1.122713  -0.752856
 Mn0.786113  -0.382656   0.454546
 P     -1.074149   0.900215   0.274750
 O      1.432040   0.404660   3.213558
 O     -0.424095  -2.636153   1.880348
 N      0.409065  -1.223900  -1.436637
 N      2.033342   0.842275  -0.758547
 C     -2.297319   0.200681  -0.910772
 C     -2.065309  -0.979968  -1.743247
 C     -3.161120  -1.063388  -2.681889
 H     -3.301581  -1.845344  -3.438615
 C     -4.066705   0.025886  -2.436443
 H     -5.007646   0.213296  -2.970958
 C     -3.545861   0.800671  -1.344755
 H     -4.008935   1.697985  -0.914470
 C     -3.773387  -2.245192   0.974014
 H     -2.878432  -2.398000   1.590992
 C     -4.174460  -3.064039  -0.139261
 H     -3.644709  -3.950759  -0.512676
 C     -5.380458  -2.504144  -0.693824
 H     -5.926723  -2.884461  -1.567384
 C     -5.726876  -1.339518   0.080193
 H     -6.584066  -0.677073  -0.100331
 C     -4.732749  -1.176947   1.108732
 H     -4.697891  -0.371639   1.853728
 C     -0.749134   2.602664  -0.412588
 C     -0.012132   3.500789   0.397134
 H      0.289164   3.201095   1.414040
 C      0.341518   4.772480  -0.081705
 H      0.908062   5.460620   0.565895
 C     -0.020034   5.164618  -1.385252
 H      0.260027   6.161161  -1.761986
 C     -0.738003   4.275109  -2.202316
 H     -1.025274   4.571173  -3.224028
 C     -1.101914   3.002479  -1.720315
 H     -1.678529   2.325695  -2.369572
 C     -2.098716   1.316155   1.774934
 C     -2.054835   0.485379   2.915672
 H     -1.393065  -0.391793   2.932515
 C     -2.855825   0.767215   4.037437
 H     -2.807524   0.108821   4.919375
 C     -3.709580   1.884635   4.036363
 H     -4.334067   2.106331   4.916378
 C     -3.755148   2.722867   2.907171
 H     -4.415528   3.604763   2.898469
 C     -2.953400   2.443361   1.786547
 H     -2.988011   3.118774   0.917403
 C     -0.889618  -1.946235  -1.659335
 H     -1.013530  -2.569334  -0.751983
 C      0.729727  -0.265970  -2.507191
 H      0.970086  -0.764938  -3.471075
 H     -0.164757   0.369957  -2.696427
 C      1.864905   0.631467  -2.105330
 C      3.014449   1.713300  -0.416422
 C      3.826328   2.401077  -1.335271
 H      4.591215   3.094945  -0.962015
 C      3.641268   2.167775  -2.701090
 C      2.648838   1.256468  -3.085941
 H      2.463499   1.029138  -4.145852
```

```
 C     -0.815907  -2.902532  -2.862008
 H     -1.709806  -3.556956  -2.888250
 H     -0.761829  -2.366927  -3.831742
 H      0.075029  -3.555812  -2.767830
 C      1.222734   0.170066   2.072118
 C      0.009292  -1.702979   1.285242
 H      1.172877  -1.959692  -1.450049
 H      2.110944  -1.369221   0.461668
 C      3.060281  -2.588219  -0.204842
 C      4.338144  -1.829860   0.035373
 C      2.908943  -3.515526   1.026369
 C      4.761447  -2.013952   1.371134
 C      5.101777  -1.090282  -0.882474
 C      3.755917  -2.856178   2.136782
 C      5.965721  -1.437630   1.806417
 C      6.308377  -0.515913  -0.443397
 H      4.757921  -0.980066  -1.923640
 C      6.733727  -0.687419   0.891913
 H      6.314267  -1.573526   2.843544
 H      6.931156   0.063592  -1.143709
 H      7.683722  -0.236123   1.221168
 O      2.579940  -2.833171  -1.347733
 H      4.238285  -3.583549   2.821969
 H      3.120852  -2.192728   2.767134
 H      1.854761  -3.708201   1.298264
 H      3.360785  -4.487825   0.721652
 H      4.264635   2.682246  -3.447582
 Cl3.333884   2.034192   1.267939
---------------------------------------
Mn12/TS-iii
Frequencies, energies and thermodynamic properties:
 Lowest Vibrational Mode (1/cm) =-668.4102
 2nd Lowest Vibrational Mode (1/cm) =                      19.4391
 E(RB-P86) (a.u.) =                                      -4712.05132296
 Thermal correction to Enthalpy (a.u.) =                   0.548702
 Thermal correction to Gibbs Free Energy (a.u.) =          0.442061
 Total Entropy (cal/Kmol) =                                224.445
 E(RPBE1PBE) (a.u.) =                                    -4711.33872261
Optimised cartesian coordinates (Angstrom):
 Fe3.218639  -0.778336   0.547089
 Mn-1.088770  -0.384220  -1.630145
 P     -0.079573   0.585259   0.166757
 O     -1.793293   2.135524  -3.004937
 O      1.210360  -0.142008  -3.429487
 N     -0.574614  -2.277775  -0.915663
 N     -2.890464  -0.906250  -0.559748
 C      1.205334  -0.517864   0.878065
 C      1.480451  -1.887475   0.441493
 C      2.398287  -2.455898   1.401681
 H      2.822512  -3.467209   1.359809
 C      2.705804  -1.469679   2.402650
 H      3.395733  -1.599756   3.247411
 C      1.982295  -0.271070   2.080745
 H      2.006892   0.666679   2.650879
 C      3.834638   0.022129  -1.252075
 H      3.179420   0.285185  -2.091762
 C      4.444940  -1.262629  -1.036911
 H      4.340637  -2.143549  -1.684499
 C      5.196168  -1.199552   0.191033
 H      5.761542  -2.024319   0.645371
 C      5.051885   0.128082   0.733006
 H      5.489009   0.491508   1.672800
 C      4.207884   0.882659  -0.156722
 H      3.889016   1.924028  -0.019383
 C     -1.228195   0.902896   1.598727
 C     -2.201278   1.919194   1.443968
 H     -2.227736   2.518662   0.519723
 C     -3.137861   2.173574   2.459093
 H     -3.884543   2.972352   2.324219
 C     -3.126140   1.408376   3.641067
 H     -3.861695   1.606860   4.436704
 C     -2.171695   0.388817   3.798248
 H     -2.154401  -0.215928   4.719078
 C     -1.227904   0.136215   2.784000
 H     -0.480645  -0.659400   2.926664
 C      0.766124   2.239840   0.026540
 C      1.211923   2.711020  -1.227616
 H      1.034523   2.119080  -2.135999
 C      1.889905   3.939548  -1.331038
 H      2.228461   4.291771  -2.318383
 C      2.130123   4.715815  -0.183666
 H      2.658781   5.678736  -0.266147
 C      1.683109   4.259023   1.069851
 H      1.859455   4.862702   1.974446
 C      1.002667   3.033376   1.173872
```

```
 H      0.643263   2.699302   2.159628
 C      0.848089  -2.597721  -0.765249
 H      1.346254  -2.213292  -1.679136
 C     -1.389895  -2.680118   0.205274
 H     -1.494542  -3.789897   0.298735
 H     -0.961744  -2.353425   1.196740
 C     -2.765921  -2.082199   0.136345
 C     -4.119885  -0.338124  -0.567440
 C     -5.247016  -0.874513   0.077079
 C     -5.106055  -2.080810   0.772745
 C     -3.846834  -2.690673   0.796141
 C      1.114868  -4.118474  -0.730659
 H      2.201374  -4.333376  -0.794653
 H      0.734073  -4.595313   0.196405
 H      0.618993  -4.600400  -1.597521
 C     -1.604293   1.132350  -2.413109
 C      0.326459  -0.239379  -2.647269
 H     -1.334480  -1.921078  -2.254035
 H     -1.793298  -1.355295  -2.835733
 H     -6.207296  -0.345171   0.016851
 H     -3.683897  -3.641124   1.324880
 H     -5.968889  -2.534671   1.283255
 Cl-4.373965   1.159960  -1.426138
---------------------------------------
Mn13/i
Frequencies, energies and thermodynamic properties:
 Lowest Vibrational Mode (1/cm) =                          19.6520
 2nd Lowest Vibrational Mode (1/cm) =                      25.0349
 E(RB-P86) (a.u.) =                                      -4462.63163200
 Thermal correction to Enthalpy (a.u.) =                   0.652909
 Thermal correction to Gibbs Free Energy (a.u.) =          0.537720
 Total Entropy (cal/Kmol) =                                242.436
 E(RPBE1PBE) (a.u.) =                                    -4461.94626864
Optimised cartesian coordinates (Angstrom):
 Fe3.691358  -0.802508  -0.194742
 Mn-1.022290  -0.286562  -0.923513
 P      0.452550   0.593690   0.421798
 O     -2.318379   2.354108  -1.313874
 O      0.475526   0.081986  -3.408072
 N     -0.385873  -2.042761  -0.477462
 N     -2.748891  -1.122836   0.030432
 C      1.890308  -0.500875   0.743263
 C      1.980829  -1.869658   0.252598
 C      3.137566  -2.471741   0.870156
 H      3.500549  -3.493164   0.697265
 C      3.762733  -1.498565   1.728328
 H      4.675513  -1.651839   2.320092
 C      3.003976  -0.279262   1.647995
 H      3.225221   0.652693   2.184287
 C      3.701974  -0.041977  -2.110222
 H      2.811224   0.199104  -2.705395
 C      4.365683  -1.317964  -2.071004
 H      4.074872  -2.214402  -2.635204
 C      5.463855  -1.222851  -1.142488
 H      6.153561  -2.033917  -0.872671
 C      5.480597   0.115916  -0.609596
 H      6.185846   0.503681   0.137822
 C      4.390626   0.845187  -1.204825
 H      4.118239   1.887634  -0.994982
 C     -0.308627   0.786534   2.113129
 C     -1.329128   1.755609   2.276600
 H     -1.610275   2.404041   1.431177
 C     -1.983836   1.902635   3.509717
 H     -2.767815   2.668379   3.623323
 C     -1.643402   1.072611   4.596098
 H     -2.159718   1.186385   5.562552
 C     -0.641841   0.099717   4.439909
 H     -0.368628  -0.553274   5.284308
 C      0.023737  -0.043943   3.206891
 H      0.817563  -0.799057   3.103965
 C      1.191838   2.278300   0.149755
 C      1.273963   2.815927  -1.153162
 H      0.873256   2.251158  -2.007170
 C      1.870212   4.071261  -1.371096
 H      1.925060   4.477542  -2.393624
 C      2.389227   4.806629  -0.290388
 H      2.853502   5.790802  -0.461921
 C      2.304569   4.282438   1.012528
 H      2.701251   4.854639   1.866388
 C      1.706504   3.029109   1.232506
 H      1.630672   2.639685   2.259926
 C      0.989056  -2.495314  -0.737447
 H      1.244812  -2.102658  -1.742456
 C     -1.006075  -2.662029   0.675509
 H     -1.180582  -3.761494   0.543228
```

```
 H     -0.364266  -2.590545   1.593731
 C     -2.339276  -2.025475   0.965578
 C     -4.034583  -0.628939   0.100256
 C     -4.800756  -0.808917   1.289891
 H     -5.783948  -0.332694   1.395526
 C     -4.318538  -1.661168   2.285338
 C     -3.096306  -2.335065   2.103111
 H     -2.718092  -3.057889   2.840473
 C      1.139393  -4.027272  -0.812662
 H      2.140994  -4.297850  -1.204532
 H      1.023575  -4.517249   0.176023
 H      0.380815  -4.448891  -1.503488
 C     -1.824926   1.283113  -1.186215
 C     -0.083126  -0.038904  -2.363573
 H     -4.918218  -1.830674   3.193676
 N     -4.558594  -0.015535  -1.004041
 C     -4.165519  -0.358549  -2.390682
 C     -5.864492   0.664813  -0.969206
 C     -5.436266  -0.068560  -3.207992
 H     -3.832185  -1.416358  -2.439351
 C     -6.087746   1.086591  -2.428330
 H     -5.846563   1.519060  -0.259698
 H     -6.107315  -0.954382  -3.209413
 H     -5.555191   2.039229  -2.634999
 H     -3.312090   0.263841  -2.732479
 H     -5.205804   0.183576  -4.262199
 H     -7.160053   1.235512  -2.663327
 H     -6.668963  -0.033714  -0.635239
---------------------------------------
Mn13/ii
Frequencies, energies and thermodynamic properties:
 Lowest Vibrational Mode (1/cm) =                          19.3573
 2nd Lowest Vibrational Mode (1/cm) =                      22.2222
 E(RB-P86) (a.u.) =                                      -4617.56933935
 Thermal correction to Enthalpy (a.u.) =                   0.737693
 Thermal correction to Gibbs Free Energy (a.u.) =          0.608722
 Total Entropy (cal/Kmol) =                                271.443
 E(RPBE1PBE) (a.u.) =                                    -4616.89325732
Optimised cartesian coordinates (Angstrom):
 Fe-3.698838   1.211224   0.249425
 Mn0.900905   0.165040  -0.636260
 P     -0.848972  -0.948781   0.051489
 O      1.712997  -2.113620  -2.346489
 O     -0.375278   1.411865  -2.952144
 N      0.553214   1.579740   0.668544
 N      2.626417   0.048930   0.601276
 C     -2.096875   0.099578   0.885288
 C     -1.887496   1.509418   1.191983
 C     -2.979576   1.928500   2.037232
 H     -3.134801   2.941184   2.431057
 C     -3.859020   0.808473   2.249763
 H     -4.790346   0.821004   2.832097
 C     -3.325839  -0.320004   1.535596
 H     -3.766618  -1.324668   1.496193
 C     -3.644950   1.557417  -1.782650
 H     -2.758799   1.460801  -2.423527
 C     -4.046176   2.746555  -1.079467
 H     -3.524157   3.712805  -1.095302
 C     -5.237938   2.441423  -0.328943
 H     -5.780962   3.132566   0.329588
 C     -5.575454   1.061770  -0.571950
 H     -6.421931   0.517854  -0.131559
 C     -4.589761   0.513924  -1.467559
 H     -4.551897  -0.519986  -1.834608
 C     -0.301624  -2.116668   1.397658
 C      0.567930  -3.176727   1.040680
 H      0.858074  -3.319264  -0.012484
 C      1.060087  -4.056005   2.018188
 H      1.727550  -4.881257   1.723014
 C      0.705346  -3.882508   3.370389
 H      1.094899  -4.570751   4.137243
 C     -0.147800  -2.827665   3.735573
 H     -0.431521  -2.685281   4.790612
 C     -0.650847  -1.948852   2.756544
 H     -1.331675  -1.137563   3.055166
 C     -1.830310  -2.075850  -1.053136
 C     -1.893843  -1.835947  -2.443231
 H     -1.339615  -0.995038  -2.884353
 C     -2.667623  -2.665466  -3.274928
 H     -2.705370  -2.466726  -4.357772
 C     -3.384678  -3.745642  -2.729729
 H     -3.987441  -4.396422  -3.382911
 C     -3.321556  -3.996000  -1.346853
 H     -3.873961  -4.844046  -0.911661
 C     -2.546633  -3.169909  -0.513863
```

```
 H     -2.492037  -3.388458   0.564187
 C     -0.707803   2.346501   0.684898
 H     -0.912497   2.586054  -0.378800
 C      1.073802   1.270550   1.993483
 H      1.385240   2.179829   2.567551
 H      0.306048   0.775536   2.643644
 C      2.271520   0.361735   1.880208
 C      3.819176  -0.604967   0.376566
 C      4.496672  -1.229799   1.468438
 C      4.056815  -0.982643   2.769360
 C      2.965981  -0.121011   2.995950
 C     -0.594954   3.695382   1.422047
 H     -1.499022   4.312506   1.244352
 H     -0.481081   3.575517   2.519199
 H      0.280194   4.251381   1.030136
 C      1.422233  -1.181085  -1.676529
 C      0.076823   0.874423  -1.992678
 H      1.780985   2.722576  -0.086202
 O      2.378205   3.431545  -0.483989
 C      3.353437   3.806135   0.478953
 H      3.988824   2.933985   0.778066
 H      2.872701   4.180610   1.418498
 H      2.637863   0.144159   4.011299
 H      5.380730  -1.855249   1.291977
 C      4.240065   4.904506  -0.098931
 H      5.004154   5.227527   0.638243
 H      4.766579   4.550774  -1.010344
 H      3.631951   5.790103  -0.379915
 H      4.592283  -1.436767   3.618168
 N      4.346639  -0.595148  -0.882206
 C      4.044203   0.437174  -1.901955
 C      5.607096  -1.297254  -1.194207
 C      5.382721   0.607964  -2.637162
 H      3.656529   1.357452  -1.417117
 C      5.956617  -0.819195  -2.612332
 H      5.480512  -2.398351  -1.119536
 H      6.047442   1.301402  -2.078116
 H      5.444339  -1.451303  -3.368595
 H      6.403314  -1.001146  -0.471643
 H      7.045386  -0.867967  -2.810895
 H      5.249183   1.013234  -3.659842
 H      3.260823   0.082920  -2.604496
---------------------------------------
Mn13/iii
Frequencies, energies and thermodynamic properties:
 Lowest Vibrational Mode (1/cm) =                         21.9620
 2nd Lowest Vibrational Mode (1/cm) =                     25.3721
 E(RB-P86) (a.u.) =                                     -4618.73582549
 Thermal correction to Enthalpy (a.u.) =                  0.754134
 Thermal correction to Gibbs Free Energy (a.u.) =         0.626061
 Total Entropy (cal/Kmol) =                               269.553
 E(RPBE1PBE) (a.u.) =                                   -4618.05865647
Optimised cartesian coordinates (Angstrom):
 Fe-3.783435   0.792912   0.262548
 Mn0.929822   0.505748  -0.911660
 P     -0.590359  -0.845399   0.118113
 O      1.767896  -1.804532  -2.563120
 O     -0.855283   1.331690  -3.085191
 N      0.379374   2.017901   0.405879
 N      2.481185   0.336614   0.574681
 C     -1.962045   0.080757   0.903673
 C     -2.010276   1.533896   1.026156
 C     -3.119360   1.845290   1.897323
 H     -3.444211   2.853315   2.184810
 C     -3.760087   0.621142   2.298893
 H     -4.647853   0.536654   2.940271
 C     -3.059309  -0.470534   1.680119
 H     -3.304804  -1.535087   1.786013
 C     -3.942442   0.834189  -1.794874
 H     -3.106666   0.777799  -2.502914
 C     -4.474698   2.042334  -1.223999
 H     -4.119503   3.062063  -1.424606
 C     -5.538941   1.678456  -0.323305
 H     -6.134365   2.371199   0.286507
 C     -5.665584   0.242694  -0.341294
 H     -6.375381  -0.348936   0.252447
 C     -4.676408  -0.279941  -1.248039
 H     -4.499342  -1.339685  -1.473297
 C      0.099147  -1.887521   1.503112
 C      1.047363  -2.880859   1.159324
 H      1.344637  -3.017045   0.106816
 C      1.601815  -3.712197   2.146197
 H      2.329230  -4.488325   1.859685
 C      1.230780  -3.553809   3.495105
 H      1.664968  -4.206417   4.269096
```

```
 C      0.305063  -2.557130   3.848135
 H      0.010601  -2.423113   4.901329
 C     -0.257838  -1.727628   2.859265
 H     -0.991033  -0.960467   3.150854
 C     -1.467446  -2.165778  -0.870606
 C     -1.620724  -2.048608  -2.269181
 H     -1.186241  -1.194002  -2.804960
 C     -2.332606  -3.019706  -2.996771
 H     -2.437974  -2.910721  -4.087884
 C     -2.902321  -4.123671  -2.338662
 H     -3.457839  -4.884426  -2.909733
 C     -2.750565  -4.253928  -0.946259
 H     -3.186341  -5.117969  -0.419661
 C     -2.035094  -3.286564  -0.219244
 H     -1.911080  -3.415232   0.867068
 C     -1.014576   2.515768   0.399269
 H     -1.286470   2.629826  -0.670580
 C      0.840977   1.719404   1.754930
 H      1.047636   2.641173   2.356635
 H      0.073156   1.165839   2.359559
 C      2.086454   0.873903   1.764622
 C      3.643571  -0.408337   0.541446
 C      4.276192  -0.797347   1.754652
 C      3.825171  -0.282132   2.970524
 C      2.744626   0.610390   2.976259
 C     -1.138927   3.919960   1.032298
 H     -2.146945   4.347544   0.853648
 H     -0.971564   3.904464   2.129508
 H     -0.390677   4.594592   0.570111
 C      1.483703  -0.871069  -1.899175
 C     -0.203998   0.961447  -2.168540
 H      2.114619   1.315774  -1.847613
 H      1.856292   1.939277  -1.366703
 H      1.246373   3.250435  -0.175998
 O      1.789454   4.000642  -0.668100
 C      2.942184   4.321357   0.087606
 H      2.673094   4.685918   1.113409
 H      3.596884   3.424672   0.244475
 H      2.377809   1.069263   3.905961
 H      5.160147  -1.446182   1.727983
 C      3.746101   5.405515  -0.626841
 H      3.129879   6.318776  -0.767727
 H      4.651091   5.683502  -0.046836
 H      4.070745   5.058176  -1.630658
 H      4.339450  -0.551017   3.906649
 N      4.197022  -0.760401  -0.677218
 C      4.421661   0.222840  -1.769818
 C      5.191544  -1.849077  -0.761990
 C      5.735521  -0.238119  -2.425475
 H      4.472213   1.253387  -1.361843
 C      5.703609  -1.758903  -2.203604
 H      4.726218  -2.824141  -0.506579
 H      6.609544   0.204723  -1.901422
 H      4.984345  -2.238571  -2.900788
 H      3.600637   0.193454  -2.517047
 H      5.792962   0.057676  -3.491988
 H      6.688445  -2.250156  -2.330793
 H      6.043440  -1.686536  -0.059158
---------------------------------------
Mn13/iv
Frequencies, energies and thermodynamic properties:
 Lowest Vibrational Mode (1/cm) =                          18.1187
 2nd Lowest Vibrational Mode (1/cm) =                      22.5509
 E(RB-P86) (a.u.) =                                      -4618.76268931
 Thermal correction to Enthalpy (a.u.) =                   0.758358
 Thermal correction to Gibbs Free Energy (a.u.) =          0.629375
 Total Entropy (cal/Kmol) =                                271.468
 E(RPBE1PBE) (a.u.) =                                    -4618.08524579
Optimised cartesian coordinates (Angstrom):
 Fe-3.787013   0.704524   0.332383
 Mn0.962563   0.635778  -0.704370
 P     -0.567384  -0.875698   0.059182
 O      1.864175  -1.266359  -2.760800
 O     -0.626163   1.839426  -2.850856
 N      0.311776   1.983392   0.828056
 N      2.438376   0.297545   0.808470
 C     -1.987324  -0.089018   0.934056
 C     -2.068352   1.332841   1.266411
 C     -3.213500   1.506712   2.131392
 H     -3.564967   2.457988   2.550177
 C     -3.847595   0.230758   2.323460
 H     -4.758557   0.046403   2.908653
 C     -3.104082  -0.750898   1.583425
 H     -3.336769  -1.821896   1.522352
 C     -3.829719   1.088713  -1.691399
```

```
 H     -2.948444   1.181308  -2.339225
 C     -4.449792   2.162931  -0.961541
 H     -4.130669   3.213925  -0.964013
 C     -5.548058   1.614877  -0.207082
 H     -6.208653   2.172927   0.470009
 C     -5.608644   0.200312  -0.474001
 H     -6.323821  -0.508352  -0.035168
 C     -4.545428  -0.126085  -1.388348
 H     -4.305983  -1.125773  -1.772839
 C      0.064321  -2.105070   1.315865
 C      1.047776  -3.025016   0.875770
 H      1.360449  -3.026196  -0.181520
 C      1.623934  -3.942908   1.768824
 H      2.377493  -4.659389   1.404247
 C      1.244105  -3.945736   3.125225
 H      1.698170  -4.663517   3.826776
 C      0.283331  -3.025185   3.576700
 H     -0.020574  -3.017459   4.635895
 C     -0.303842  -2.111835   2.679183
 H     -1.066429  -1.409332   3.049420
 C     -1.407556  -2.035366  -1.141470
 C     -1.509953  -1.690575  -2.506941
 H     -1.071148  -0.750629  -2.869612
 C     -2.175849  -2.536853  -3.412322
 H     -2.244033  -2.251007  -4.474189
 C     -2.747778  -3.742337  -2.967934
 H     -3.266538  -4.405761  -3.678201
 C     -2.646284  -4.098703  -1.610721
 H     -3.085414  -5.043467  -1.251904
 C     -1.978067  -3.254436  -0.706194
 H     -1.892760  -3.556689   0.349309
 C     -1.121908   2.437333   0.813776
 H     -1.326567   2.658912  -0.252521
 C      0.761180   1.492677   2.142692
 H      0.865611   2.308929   2.892156
 H     -0.017125   0.806329   2.542738
 C      2.058502   0.729572   2.045027
 C      3.629304  -0.393945   0.712453
 C      4.339040  -0.786504   1.878770
 C      3.901310  -0.378404   3.139569
 C      2.760478   0.431877   3.223329
 C     -1.312281   3.744788   1.601456
 H     -2.328393   4.153879   1.432669
 H     -1.180313   3.606137   2.694088
 H     -0.584464   4.503260   1.248475
 C      1.559098  -0.505380  -1.903425
 C     -0.047501   1.318832  -1.951086
 H      1.954238   1.801836  -1.183454
 H      1.873838   3.251070  -0.947398
 H      0.873617   2.824781   0.579862
 O      1.788971   4.139383  -0.480426
 C      3.106002   4.568093  -0.119862
 H      2.980708   5.359905   0.650524
 H      3.675777   3.738405   0.363123
 H      2.391182   0.811449   4.187331
 H      5.266282  -1.365110   1.777756
 C      3.889799   5.118159  -1.311084
 H      3.338858   5.953567  -1.791286
 H      4.883366   5.496483  -0.989637
 H      4.057580   4.331807  -2.077324
 H      4.464852  -0.658557   4.043123
 N      4.133258  -0.706082  -0.544163
 C      4.493557   0.366901  -1.498533
 C      5.153607  -1.749544  -0.697608
 C      5.065650  -0.387010  -2.722654
 H      3.609980   0.996407  -1.719855
 C      5.317588  -1.838424  -2.224611
 H      4.818921  -2.696800  -0.227660
 H      4.346006  -0.380753  -3.563945
 H      4.551809  -2.523025  -2.640522
 H      6.124811  -1.456426  -0.221555
 H      6.314208  -2.231609  -2.508047
 H      5.997085   0.099674  -3.075898
 H      5.275646   1.019299  -1.036617
---------------------------------------
Mn13/v
Frequencies, energies and thermodynamic properties:
 Lowest Vibrational Mode (1/cm) =                        21.1468
 2nd Lowest Vibrational Mode (1/cm) =                    29.7348
 E(RB-P86) (a.u.) =                                    -4463.82478807
 Thermal correction to Enthalpy (a.u.) =                 0.673934
 Thermal correction to Gibbs Free Energy (a.u.) =        0.559474
 Total Entropy (cal/Kmol) =                              240.903
 E(RPBE1PBE) (a.u.) =                                  -4463.14199423
Optimised cartesian coordinates (Angstrom):
```

```
 Fe3.695210  -0.506530  -0.122690
 Mn-0.970629  -0.047856  -1.360887
 P      0.251530   0.346956   0.522163
 O     -2.178095   2.631350  -1.191689
 O      0.810346   1.111824  -3.374876
 N     -0.129654  -2.017597  -1.421520
 N     -2.522015  -1.209442  -0.461749
 C      1.779733  -0.685104   0.611760
 C      2.095437  -1.780854  -0.305040
 C      3.243927  -2.474746   0.233829
 H      3.746439  -3.338118  -0.219975
 C      3.652867  -1.820855   1.447102
 H      4.513846  -2.099824   2.069357
 C      2.762901  -0.717128   1.678444
 H      2.812416  -0.016460   2.521973
 C      3.832676   0.870245  -1.650152
 H      2.991047   1.237160  -2.252214
 C      4.630679  -0.291155  -1.943397
 H      4.510509  -0.955641  -2.809714
 C      5.600086  -0.445297  -0.888619
 H      6.343230  -1.249851  -0.806902
 C      5.402818   0.624384   0.056056
 H      5.970036   0.777871   0.983933
 C      4.309574   1.435513  -0.412158
 H      3.895481   2.317733   0.092454
 C     -0.592722  -0.027754   2.147754
 C     -1.733864   0.749340   2.466832
 H     -2.039831   1.569470   1.796296
 C     -2.477021   0.492322   3.630212
 H     -3.353301   1.116917   3.867366
 C     -2.108636  -0.562964   4.487682
 H     -2.693993  -0.768041   5.398125
 C     -0.990875  -1.353379   4.170942
 H     -0.694455  -2.182795   4.833269
 C     -0.237131  -1.087737   3.010716
 H      0.644216  -1.709180   2.788509
 C      0.892252   2.066679   0.873919
 C      1.077954   2.979191  -0.187670
 H      0.822499   2.681703  -1.214528
 C      1.593424   4.265594   0.054384
 H      1.729951   4.964213  -0.786645
 C      1.928740   4.661476   1.361740
 H      2.329446   5.670174   1.550838
 C      1.742079   3.762392   2.427606
 H      1.996114   4.063346   3.456766
 C      1.224336   2.477236   2.186137
 H      1.068661   1.791314   3.033584
 C      1.358612  -2.154578  -1.585859
 H      1.613372  -1.412701  -2.368701
 C     -0.686522  -2.828511  -0.322707
 H     -0.656984  -3.919966  -0.536937
 H     -0.047925  -2.670579   0.573361
 C     -2.098375  -2.415657   0.012802
 C     -3.824589  -0.841141  -0.188810
 C     -4.621904  -1.598678   0.711565
 H     -5.641366  -1.264085   0.943472
 C     -4.140684  -2.798417   1.237603
 C     -2.868792  -3.241129   0.845010
 H     -2.457129  -4.199390   1.194565
 C      1.753654  -3.544791  -2.111185
 H      2.825498  -3.560031  -2.391951
 H      1.588899  -4.345243  -1.361401
 H      1.172100  -3.792127  -3.023295
 C     -1.745327   1.527015  -1.252620
 C      0.147965   0.645067  -2.502404
 H     -0.555145  -2.328318  -2.305615
 H     -1.634682  -0.475826  -2.737230
 H     -4.766285  -3.401080   1.914248
 N     -4.351970   0.281657  -0.808977
 C     -4.416131   0.370785  -2.284449
 C     -5.570215   0.919287  -0.300010
 C     -5.094194   1.734716  -2.554957
 H     -3.399930   0.265655  -2.714358
 C     -5.690182   2.171733  -1.186428
 H     -5.471968   1.146906   0.781138
 H     -4.358354   2.478766  -2.916980
 H     -5.084235   2.991439  -0.751682
 H     -5.046167  -0.467233  -2.673909
 H     -5.875186   1.631082  -3.335112
 H     -6.738100   2.525279  -1.257894
 H     -6.469263   0.262972  -0.430069
---------------------------------------
Mn13/vi_R
Frequencies, energies and thermodynamic properties:
 Lowest Vibrational Mode (1/cm) =                        9.2286
```

```
 2nd Lowest Vibrational Mode (1/cm) =                     12.2028
 E(RB-P86) (a.u.) =                                     -4886.53890482
 Thermal correction to Enthalpy (a.u.) =                  0.827665
 Thermal correction to Gibbs Free Energy (a.u.) =         0.688199
 Total Entropy (cal/Kmol) =                               293.531
 E(RPBE1PBE) (a.u.) =                                   -4885.85075825
Optimised cartesian coordinates (Angstrom):
 Fe-2.692267  -2.792471  -0.542153
 Mn0.438443   0.729892   0.324812
 P     -1.741006   0.566071   0.262770
 O      0.249664   2.397903   2.766715
 O      0.890537  -1.592496   2.045642
 N      0.607894  -0.177918  -1.396373
 N      1.136605   2.325725  -0.910334
 C     -2.321084  -0.792903  -0.818258
 C     -1.438907  -1.590683  -1.660779
 C     -2.280099  -2.393672  -2.516354
 H     -1.931451  -3.122529  -3.259045
 C     -3.658325  -2.110897  -2.212290
 H     -4.532420  -2.582133  -2.681897
 C     -3.691170  -1.131988  -1.159881
 H     -4.594235  -0.708599  -0.701297
 C     -1.835711  -3.518903   1.187416
 H     -1.023739  -3.038658   1.748753
 C     -1.669911  -4.458139   0.110404
 H     -0.712942  -4.822007  -0.287040
 C     -2.978316  -4.818752  -0.373665
 H     -3.193284  -5.501595  -1.206573
 C     -3.954475  -4.103462   0.408625
 H     -5.043919  -4.146579   0.276105
 C     -3.249099  -3.297767   1.371414
 H     -3.703678  -2.620171   2.105702
 C     -2.456129   2.087654  -0.541743
 C     -2.341885   3.318061   0.150601
 H     -1.876375   3.346082   1.148912
 C     -2.826586   4.505568  -0.420508
 H     -2.740323   5.452890   0.135195
 C     -3.417982   4.486909  -1.698801
 H     -3.796312   5.419423  -2.146876
 C     -3.522416   3.273322  -2.399045
 H     -3.983046   3.249544  -3.399592
 C     -3.045082   2.078886  -1.825784
 H     -3.147593   1.133527  -2.379445
 C     -2.784025   0.431733   1.796374
 C     -2.252148  -0.129702   2.977742
 H     -1.206095  -0.465793   3.007171
 C     -3.053492  -0.268648   4.125376
 H     -2.622375  -0.706531   5.039639
 C     -4.395032   0.153146   4.108880
 H     -5.020467   0.046611   5.009405
 C     -4.931674   0.719604   2.938355
 H     -5.979349   1.059677   2.917572
 C     -4.131510   0.862416   1.791125
 H     -4.560313   1.324550   0.888216
 C      0.094097  -1.546110  -1.628231
 H      0.420329  -2.131033  -0.743451
 C      0.417004   0.696767  -2.546983
 H      1.073165   0.426569  -3.412907
 H     -0.626496   0.626731  -2.948648
 C      0.696797   2.130479  -2.185779
 C      1.577596   3.580841  -0.543664
 C      1.281491   4.706106  -1.368355
 H      1.529830   5.721347  -1.034278
 C      0.744897   4.493119  -2.639101
 C      0.504819   3.182373  -3.090655
 H      0.137482   2.977828  -4.106592
 C      0.703412  -2.228772  -2.870265
 H      0.459931  -3.310583  -2.874958
 H      0.323829  -1.797060  -3.819148
 H      1.805885  -2.121291  -2.845355
 C      0.372132   1.743021   1.787625
 C      0.636052  -0.669684   1.340399
 H      2.408579  -0.480605  -1.344137
 H      3.467377  -0.647876   0.536462
 C      4.051199  -1.029550  -0.341724
 C      4.318464  -2.519804  -0.112862
 C      5.485601  -0.435874  -0.309100
 C      5.607257  -2.711573   0.434134
 C      3.475311  -3.616183  -0.351855
 C      6.310998  -1.376218   0.600666
 H      5.869051  -0.484554  -1.352041
 C      6.061942  -4.004346   0.745817
 C      3.931306  -4.913492  -0.042646
 H      2.473945  -3.467503  -0.786040
 H      7.389115  -1.414615   0.338069
```

C   5.216469 -5.105907   0.503385
H   7.069686 -4.159905   1.165904
H   3.281994 -5.783515 -0.232579
H   5.564813 -6.125274   0.736294
O   3.375403 -0.699392 -1.540486
H   5.509338   0.626009   0.008059
H   6.257452 -1.046130   1.663912
H   0.546321   5.354061 -3.296852
N   2.335319   3.702350   0.587894
C   3.230918   2.629262   1.079122
C   2.747352   5.019759   1.106398
C   4.403336   3.398562   1.710883
H   3.520250   1.970229   0.234647
C   3.723122   4.673152   2.239882
H   1.866364   5.605822   1.443548
H   4.913076   2.809431   2.498792
H   3.163221   4.451732   3.173287
H   3.263809   5.612047   0.314045
H   4.428461   5.500423   2.452496
H   5.157866   3.659854   0.938058
H   2.723547   1.990572   1.831577

Mn13/vi_S
Frequencies, energies and thermodynamic properties:
Lowest Vibrational Mode (1/cm) = 11.1900
2nd Lowest Vibrational Mode (1/cm) = 16.3618
E(RB-P86) (a.u.) = -4886.54065533
Thermal correction to Enthalpy (a.u.) = 0.827750
Thermal correction to Gibbs Free Energy (a.u.) = 0.689146
Total Entropy (cal/Kmol) = 291.716
E(RPBE1PBE) (a.u.) = -4885.85146619
Optimised cartesian coordinates (Angstrom):
Fe-3.416452 -2.340635 -0.563189
Mn0.395521   0.421967   0.330586
P   -1.768164   0.734738   0.254504
O   0.564965   2.083170   2.778598
O   0.307737 -1.941107   2.053538
N   0.371178 -0.494165 -1.395931
N   1.436954   1.829080 -0.898397
C   -2.621536 -0.467130 -0.831952
C   -1.927328 -1.435537 -1.671874
C   -2.916892 -2.036952 -2.533908
H   -2.729017 -2.823289 -3.276055
C   -4.203571 -1.464302 -2.236318
H   -5.156141 -1.736182 -2.710999
C   -4.030774 -0.503012 -1.181253
H   -4.824189   0.104149 -0.726261
C   -2.749946 -3.236218   1.170926
H   -1.859714 -2.941003   1.741287
C   -2.777360 -4.186731   0.091436
H   -1.915761 -4.744353 -0.299790
C   -4.128012 -4.260128 -0.405199
H   -4.475461 -4.879859 -1.242724
C   -4.937104 -3.355338   0.371481
H   -6.009536 -3.165676   0.229290
C   -4.085590 -2.719916   1.343519
H   -4.392424 -1.962602   2.076467
C   -2.130634   2.376269 -0.550019
C   -1.745077   3.549581   0.143475
H   -1.283136   3.472020   1.140799
C   -1.955625   4.815935 -0.424936
H   -1.660441   5.719651   0.131666
C   -2.538828   4.930839 -1.701953
H   -2.701839   5.924697 -2.148227
C   -2.911145   3.771718 -2.403405
H   -3.367406   3.852347 -3.402985
C   -2.709572   2.500125 -1.832635
H   -3.020430   1.602019 -2.387215
C   -2.825852   0.830420   1.781104
C   -2.439702   0.164146   2.964570
H   -1.493856 -0.394394   3.000630
C   -3.261034   0.203412   4.105826
H   -2.944011 -0.319836   5.021896
C   -4.476860   0.909838   4.080745
H   -5.117398   0.942246   4.976354
C   -4.866712   1.581816   2.907993
H   -5.813869   2.143799   2.880303
C   -4.045657   1.546665   1.767204
H   -4.355324   2.092604   0.862386
C   -0.420953 -1.721748 -1.630657
H   -0.230985 -2.362242 -0.745066
C   0.369589   0.408022 -2.540886
H   0.942472   0.004838 -3.414191
H   -0.666350   0.574451 -2.933772
C   0.963068   1.741551 -2.173678

```
 C      2.141559   2.955814  -0.526356
 C      2.102433   4.121066  -1.348137
 H      2.569239   5.054988  -1.010672
 C      1.532381   4.036747  -2.619332
 C      1.007760   2.813244  -3.074628
 H      0.603052   2.698866  -4.090558
 C      0.037764  -2.522090  -2.866980
 H     -0.429523  -3.527718  -2.871420
 H     -0.233762  -2.022717  -3.819836
 H      1.137770  -2.651216  -2.833294
 C      0.545325   1.421545   1.796799
 C      0.272131  -0.986113   1.345099
 H      2.057742  -1.146829  -1.358378
 H      3.057297  -1.411735   0.572341
 C      3.423436  -2.070847  -0.260809
 C      3.019238  -3.537499   0.070309
 C      4.128481  -4.050636   1.023020
 O      2.968845  -1.565782  -1.498658
 H      1.529684   4.922446  -3.274154
 C      4.942193  -2.132770  -0.173546
 C      5.889787  -1.254457  -0.720785
 C      5.346553  -3.258681   0.578456
 C      7.259511  -1.502881  -0.496732
 H      5.563945  -0.396019  -1.330141
 C      6.711200  -3.502642   0.806281
 C      7.666285  -2.616922   0.265031
 H      8.017803  -0.827973  -0.925966
 H      7.036498  -4.381309   1.387859
 H      8.739980  -2.804145   0.428920
 H      1.998709  -3.616054   0.494913
 H      3.879492  -3.808031   2.082209
 H      3.047443  -4.109332  -0.883090
 H      4.281327  -5.149466   0.980137
 N      2.901966   2.907604   0.608902
 C      3.541101   1.666187   1.105283
 C      3.581183   4.104890   1.137212
 C      4.839699   2.165566   1.760647
 H      3.700452   0.963497   0.261588
 C      4.440760   3.555818   2.284524
 H      2.842506   4.867751   1.462304
 H      5.197605   1.479140   2.553275
 H      3.830574   3.459244   3.207627
 H      4.226061   4.571360   0.354880
 H      5.303719   4.212070   2.512066
 H      5.645944   2.258643   1.001740
 H      2.896344   1.147543   1.844936
---------------------------------------
Mn13/viii
Frequencies, energies and thermodynamic properties:
 Lowest Vibrational Mode (1/cm) =                          21.9940
 2nd Lowest Vibrational Mode (1/cm) =                      26.9234
 E(RB-P86) (a.u.) =                                       -4463.79163350
 Thermal correction to Enthalpy (a.u.) =                   0.669390
 Thermal correction to Gibbs Free Energy (a.u.) =          0.554610
 Total Entropy (cal/Kmol) =                                241.577
 E(RPBE1PBE) (a.u.) =                                     -4463.10881869
Optimised cartesian coordinates (Angstrom):
 Fe3.720532  -0.568564  -0.075974
 Mn-0.964628  -0.281035  -1.345043
 P      0.294980   0.417657   0.402912
 O     -2.193672   2.410839  -1.514224
 O      0.943248   0.565689  -3.401458
 N     -0.186271  -2.151191  -1.045240
 N     -2.549541  -1.209313  -0.204391
 C      1.797675  -0.602105   0.663817
 C      2.086153  -1.839713  -0.052442
 C      3.224930  -2.445442   0.596312
 H      3.710722  -3.382114   0.293684
 C      3.650537  -1.602873   1.683255
 H      4.506615  -1.785702   2.347072
 C      2.782393  -0.457350   1.722403
 H      2.847619   0.373933   2.436316
 C      3.900487   0.595053  -1.772202
 H      3.071935   0.930918  -2.407894
 C      4.626577  -0.636778  -1.926649
 H      4.452426  -1.395979  -2.700993
 C      5.605846  -0.712544  -0.872025
 H      6.306394  -1.540624  -0.699026
 C      5.485674   0.477380  -0.067192
 H      6.079769   0.713815   0.825916
 C      4.429034   1.284128  -0.621033
 H      4.075042   2.246715  -0.229434
 C     -0.568808   0.365112   2.056296
 C     -1.663885   1.242125   2.244736
 H     -1.969601   1.921493   1.432419
```

```
 C     -2.355955   1.270171   3.466550
 H     -3.198558   1.967186   3.600469
 C     -1.975346   0.410876   4.514981
 H     -2.517268   0.433434   5.473837
 C     -0.902087  -0.477392   4.329787
 H     -0.599258  -1.156463   5.142929
 C     -0.202624  -0.502038   3.107843
 H      0.642306  -1.195739   2.981587
 C      0.946129   2.169652   0.444336
 C      1.120384   2.909340  -0.745625
 H      0.837774   2.475174  -1.714089
 C      1.659953   4.208269  -0.710866
 H      1.784928   4.769223  -1.650745
 C      2.033468   4.789569   0.513493
 H      2.453796   5.807504   0.539660
 C      1.858489   4.064449   1.706164
 H      2.140647   4.511316   2.672922
 C      1.314951   2.768382   1.672519
 H      1.168017   2.223804   2.618012
 C      1.247095  -2.389492  -1.217749
 H      1.548558  -1.832749  -2.129924
 C     -0.716544  -2.790830   0.135230
 H     -0.806833  -3.906332   0.032160
 H     -0.068833  -2.664847   1.051878
 C     -2.078663  -2.271334   0.509350
 C     -3.824653  -0.750033   0.060744
 C     -4.525575  -1.211399   1.208678
 C     -3.999245  -2.258355   1.968735
 C     -2.781658  -2.836611   1.586186
 C      1.580157  -3.874717  -1.489121
 H      2.641345  -3.999373  -1.789631
 H      1.407978  -4.515673  -0.599202
 H      0.943103  -4.251985  -2.315100
 C     -1.756783   1.313382  -1.465815
 C      0.233691   0.238262  -2.509980
 H     -1.563036  -1.421133  -2.532288
 H     -1.944215  -0.736538  -2.766483
 H     -5.509736  -0.794284   1.455283
 H     -2.348574  -3.688349   2.131028
 H     -4.558252  -2.642830   2.836498
 N     -4.421456   0.143899  -0.810104
 C     -4.431501  -0.061202  -2.282514
 C     -5.606025   0.919696  -0.395446
 C     -5.790130   0.506736  -2.731234
 H     -4.305097  -1.136645  -2.526148
 C     -6.056387   1.605862  -1.689752
 H     -5.345918   1.623569   0.422873
 H     -6.578746  -0.273711  -2.669784
 H     -5.424406   2.495962  -1.894315
 H     -3.605917   0.495452  -2.773553
 H     -5.760922   0.877774  -3.775189
 H     -7.113624   1.934258  -1.646425
 H     -6.425250   0.257823  -0.023608
---------------------------------------
Mn13/ix
Frequencies, energies and thermodynamic properties:
 Lowest Vibrational Mode (1/cm) =                         19.4652
 2nd Lowest Vibrational Mode (1/cm) =                     28.4142
 E(RB-P86) (a.u.) =                                     -4617.58033421
 Thermal correction to Enthalpy (a.u.) =                  0.738320
 Thermal correction to Gibbs Free Energy (a.u.) =         0.614436
 Total Entropy (cal/Kmol) =                               260.735
 E(RPBE1PBE) (a.u.) =                                   -4616.90409600
Optimised cartesian coordinates (Angstrom):
 Fe-3.787473  -0.352724  -0.443071
 Mn0.943008  -1.019823   0.481847
 P     -0.403273   0.763268   0.089127
 O      2.061008   0.317631   2.855372
 O     -0.909755  -2.238408   2.394090
 N      0.129412  -1.940207  -1.250914
 N      2.386129  -0.434468  -1.051575
 C     -1.891956   0.306624  -0.906614
 C     -2.146706  -1.007973  -1.495965
 C     -3.293763  -0.875601  -2.364521
 H     -3.754753  -1.682601  -2.947840
 C     -3.761523   0.482650  -2.310793
 H     -4.634818   0.884947  -2.841609
 C     -2.911487   1.210022  -1.409453
 H     -3.012104   2.271738  -1.150887
 C     -3.922175  -1.055880   1.491116
 H     -3.073039  -1.346702   2.122126
 C     -4.631659  -1.923460   0.588669
 H     -4.424828  -2.989024   0.420675
 C     -5.646311  -1.144539  -0.074097
 H     -6.343926  -1.509717  -0.839765
```

```
 C     -5.564553   0.205995   0.421706
 H     -6.189439   1.049650   0.099202
 C     -4.497327   0.261913   1.386623
 H     -4.163925   1.153755   1.932913
 C      0.355242   2.150551  -0.905568
 C      1.373830   2.909223  -0.279208
 H      1.663243   2.683130   0.760178
 C      2.012324   3.955362  -0.964197
 H      2.792910   4.544000  -0.456081
 C      1.658336   4.250280  -2.295235
 H      2.159308   5.070836  -2.833082
 C      0.663640   3.489693  -2.932476
 H      0.381253   3.709901  -3.974547
 C      0.015171   2.446174  -2.243019
 H     -0.773051   1.871860  -2.753382
 C     -1.128398   1.757350   1.497834
 C     -1.241135   1.205216   2.792367
 H     -0.873237   0.191028   2.995387
 C     -1.827513   1.941226   3.838635
 H     -1.903305   1.491967   4.841705
 C     -2.310035   3.241481   3.609062
 H     -2.767503   3.817188   4.429346
 C     -2.195981   3.805278   2.325085
 H     -2.563156   4.826321   2.133650
 C     -1.605774   3.072166   1.280955
 H     -1.507157   3.538294   0.288436
 C     -1.323261  -2.274687  -1.284857
 H     -1.542386  -2.676644  -0.275431
 C      0.636537  -1.368684  -2.505272
 H      0.763150  -2.137600  -3.300908
 H     -0.118633  -0.650850  -2.895472
 C      1.940674  -0.630749  -2.319491
 C      3.590916   0.212044  -0.884549
 C      4.249306   0.820572  -1.984981
 C      3.745040   0.652418  -3.276795
 C      2.594829  -0.126089  -3.457171
 C     -1.656316  -3.378141  -2.303474
 H     -2.706382  -3.711578  -2.181217
 H     -1.528930  -3.040114  -3.352081
 H     -1.004694  -4.260148  -2.137216
 C      1.686510  -0.228848   1.872917
 C     -0.202114  -1.709702   1.599668
 O      1.948505  -2.784870   0.342328
 C      2.032484  -3.719616   1.379686
 H      1.026616  -4.015519   1.782665
 H      2.590459  -3.302711   2.265114
 H      2.179172  -0.319452  -4.457065
 H      5.186630   1.368606  -1.823744
 C      2.753942  -4.990278   0.909372
 H      2.830503  -5.736980   1.729430
 H      2.207772  -5.462324   0.064179
 H      3.781885  -4.757905   0.557619
 H      4.267580   1.095897  -4.138869
 N      4.160127   0.238152   0.380833
 C      4.519949  -1.043260   1.034706
 C      5.250540   1.166441   0.695645
 C      5.139185  -0.611955   2.381500
 H      3.634216  -1.706487   1.084200
 C      5.516346   0.887461   2.188442
 H      4.951144   2.212932   0.482731
 H      4.409435  -0.724864   3.205957
 H      4.862919   1.530014   2.811762
 H      0.710517  -2.766664  -0.954590
 H      6.168838   0.944203   0.093051
 H      6.566716   1.112282   2.461331
 H      6.020823  -1.238924   2.623796
 H      5.289754  -1.557577   0.404837
---------------------------------------
Mn13/x
Frequencies, energies and thermodynamic properties:
 Lowest Vibrational Mode (1/cm) =                         19.8478
 2nd Lowest Vibrational Mode (1/cm) =                     25.2213
 E(RB-P86) (a.u.) =                                     -4772.51776703
 Thermal correction to Enthalpy (a.u.) =                  0.823193
 Thermal correction to Gibbs Free Energy (a.u.) =         0.685772
 Total Entropy (cal/Kmol) =                               289.226
 E(RPBE1PBE) (a.u.) =                                   -4771.85443221
Optimised cartesian coordinates (Angstrom):
 Fe3.910545  -0.962425   0.424598
 Mn-0.797406  -0.505570  -0.770299
 P      0.810479   0.842963   0.093910
 O     -1.472583   1.575830  -2.738709
 O      0.953028  -1.524550  -2.886375
 N     -0.265074  -1.975820   0.671397
 N     -2.197604  -0.055400   0.832978
```

C	2.123714	-0.104780	0.980900
C	2.110066	-1.547081	1.223457
C	3.192949	-1.841484	2.133661
H	3.467119	-2.836354	2.506503
C	3.883476	-0.619111	2.442076
H	4.768600	-0.523897	3.085221
C	3.238676	0.448305	1.729067
H	3.534486	1.504676	1.753322
C	4.071493	-1.189664	-1.618966
H	3.237172	-1.161523	-2.331041
C	4.556890	-2.362721	-0.941744
H	4.163690	-3.382194	-1.053396
C	5.634237	-1.962717	-0.073000
H	6.201348	-2.621604	0.598171
C	5.816256	-0.540569	-0.216163
H	6.546898	0.073432	0.327336
C	4.848824	-0.061876	-1.168940
H	4.711659	0.980514	-1.484630
C	0.233497	2.063958	1.383389
C	-0.563430	3.147700	0.941214
H	-0.803168	3.250397	-0.129544
C	-1.040793	4.103954	1.851956
H	-1.646730	4.949109	1.488013
C	-0.747024	3.983541	3.224055
H	-1.120202	4.734348	3.938582
C	0.023372	2.898746	3.675496
H	0.255461	2.792673	4.747350
C	0.510700	1.943664	2.761913
H	1.126187	1.109729	3.132151
C	1.793501	1.995603	-1.003152
C	1.900632	1.765945	-2.392027
H	1.376904	0.920433	-2.856129
C	2.680164	2.612180	-3.202272
H	2.747341	2.415738	-4.284182
C	3.365392	3.702282	-2.638427
H	3.974044	4.364936	-3.274008
C	3.260554	3.945523	-1.256678
H	3.786384	4.800826	-0.802975
C	2.477731	3.103587	-0.448003
H	2.392094	3.323191	0.627257
C	1.114084	-2.553743	0.661011
H	1.342164	-2.715072	-0.411442
C	-0.765051	-1.668694	2.021437
H	-1.116392	-2.582568	2.547626
H	0.075389	-1.257350	2.622244
C	-1.882644	-0.655590	2.010881
C	-3.229829	0.858371	0.831445
C	-3.830566	1.281162	2.045810
C	-3.459226	0.684255	3.252460
C	-2.496808	-0.332255	3.233129
C	1.185285	-3.920418	1.362027
H	2.173758	-4.390805	1.188619
H	1.034655	-3.842544	2.457968
H	0.414297	-4.603158	0.950026
C	-1.279280	0.742905	-1.919764
C	0.288840	-1.093994	-2.001506
H	-2.935867	-2.790007	-0.002592
H	-0.926399	-2.633401	0.211789
O	-3.237594	-3.408314	0.750191
C	-4.546818	-3.057719	1.155936
H	-4.736679	-3.551876	2.137376
H	-4.641593	-1.957376	1.344858
H	-2.190509	-0.854401	4.151435
H	-4.626298	2.036696	2.024002
C	-5.627464	-3.492742	0.158974
H	-5.571608	-4.586685	-0.025122
H	-6.645684	-3.255894	0.536739
H	-5.498681	-2.976695	-0.816471
O	-2.170152	-2.000348	-1.173227
C	-2.133772	-2.839750	-2.306523
C	-2.609276	-2.184910	-3.609850
H	-2.787470	-3.725327	-2.097793
H	-1.109513	-3.261819	-2.480117
H	-2.544308	-2.904468	-4.455111
H	-3.664320	-1.847627	-3.531963
H	-1.987316	-1.301754	-3.865075
H	-3.941411	0.988219	4.194766
N	-3.684540	1.350220	-0.384860
C	-4.272360	0.409155	-1.368311
C	-4.519452	2.555423	-0.437688
C	-4.704986	1.314919	-2.541993
H	-3.551691	-0.395315	-1.613846
C	-4.732592	2.755256	-1.950239
H	-4.012631	3.406954	0.060436
H	-3.982907	1.251569	-3.378721

```
 H     -3.901656   3.357260  -2.369230
 H     -5.505286   2.398337   0.071437
 H     -5.677297   3.295723  -2.159252
 H     -5.695638   1.002694  -2.929521
 H     -5.169454  -0.073482  -0.903723
---------------------------------------
Mn13/TS-i
Frequencies, energies and thermodynamic properties:
 Lowest Vibrational Mode (1/cm) =-736.6729
 2nd Lowest Vibrational Mode (1/cm) =                    16.7092
 E(RB-P86) (a.u.) =                                    -4618.73056768
 Thermal correction to Enthalpy (a.u.) =                 0.749993
 Thermal correction to Gibbs Free Energy (a.u.) =        0.621188
 Total Entropy (cal/Kmol) =                              271.094
 E(RPBE1PBE) (a.u.) =                                  -4618.05222703
Optimised cartesian coordinates (Angstrom):
 Fe-3.802475   0.702585   0.370715
 Mn0.886769   0.567224  -0.932542
 P     -0.575059  -0.851272   0.086897
 O      1.694291  -1.603143  -2.774536
 O     -0.952594   1.420752  -3.053225
 N      0.316129   2.047522   0.443505
 N      2.416384   0.368313   0.531264
 C     -1.948077   0.018239   0.938859
 C     -2.032592   1.466005   1.107737
 C     -3.123234   1.722777   2.019118
 H     -3.465842   2.712787   2.345814
 C     -3.718712   0.470168   2.400889
 H     -4.585343   0.343083   3.063661
 C     -3.007237  -0.583171   1.730406
 H     -3.220896  -1.656757   1.811430
 C     -4.011569   0.815940  -1.678491
 H     -3.190341   0.816968  -2.405877
 C     -4.573613   1.981312  -1.049821
 H     -4.259758   3.020263  -1.218276
 C     -5.602984   1.545066  -0.140976
 H     -6.208074   2.192085   0.508249
 C     -5.678624   0.107554  -0.211452
 H     -6.352320  -0.531778   0.374791
 C     -4.692767  -0.344005  -1.158905
 H     -4.482861  -1.387374  -1.427794
 C      0.183470  -1.895784   1.432383
 C      1.146090  -2.858898   1.044851
 H      1.398038  -2.985728  -0.020567
 C      1.776446  -3.666827   2.005099
 H      2.514166  -4.419730   1.684796
 C      1.468761  -3.514464   3.370747
 H      1.964209  -4.147361   4.124004
 C      0.526910  -2.549715   3.766352
 H      0.279842  -2.421719   4.832390
 C     -0.113569  -1.745137   2.804108
 H     -0.859763  -1.004760   3.130281
 C     -1.444286  -2.169187  -0.908385
 C     -1.647141  -2.011431  -2.296683
 H     -1.257584  -1.125482  -2.816078
 C     -2.351334  -2.981686  -3.032739
 H     -2.496772  -2.841497  -4.115651
 C     -2.862772  -4.124704  -2.393401
 H     -3.412133  -4.884774  -2.971314
 C     -2.660999  -4.294919  -1.011664
 H     -3.051297  -5.189528  -0.500659
 C     -1.953583  -3.328039  -0.276046
 H     -1.789869  -3.486264   0.801143
 C     -1.100187   2.499260   0.477679
 H     -1.392200   2.616038  -0.585915
 C      0.837480   1.752419   1.779384
 H      1.002999   2.672094   2.390175
 H      0.109588   1.142109   2.370753
 C      2.122610   0.972180   1.718054
 C      3.582766  -0.355129   0.433170
 C      4.393116  -0.578145   1.572764
 C      4.056806   0.006608   2.796536
 C      2.919326   0.822315   2.864778
 C     -1.248819   3.888874   1.130692
 H     -2.271695   4.288561   0.976323
 H     -1.057551   3.867369   2.223553
 H     -0.531634   4.589639   0.657983
 C      1.445019  -0.738195  -2.010703
 C     -0.270505   1.045150  -2.160762
 H      2.055545   1.451100  -1.786992
 H      1.769422   2.156140  -1.394975
 H      1.001295   3.013113  -0.099094
 O      1.623747   3.791138  -0.799008
 C      2.839191   4.195181  -0.221967
 H      2.718813   4.449616   0.866012
```

```
 H      3.610665   3.375165  -0.245990
 H      2.628980   1.328292   3.797208
 H      5.317769  -1.162791   1.472346
 C      3.415538   5.417058  -0.944932
 H      2.700768   6.266697  -0.902965
 H      4.374847   5.746433  -0.490356
 H      3.601395   5.187067  -2.015951
 H      4.699372  -0.141069   3.678483
 N      3.937068  -0.846578  -0.825087
 C      4.443534   0.098945  -1.845692
 C      4.767615  -2.052991  -0.962275
 C      5.979561  -0.101051  -1.836409
 H      4.135116   1.133169  -1.607573
 C      6.185489  -1.576809  -1.390677
 H      4.313008  -2.671845  -1.768128
 H      6.444977   0.593552  -1.107066
 H      6.582817  -2.211556  -2.208074
 H      4.013676  -0.169062  -2.835662
 H      6.424236   0.117983  -2.828137
 H      6.906738  -1.642076  -0.550655
 H      4.745293  -2.660250  -0.037521
---------------------------------------
Mn13/TS-ii_si
Frequencies, energies and thermodynamic properties:
 Lowest Vibrational Mode (1/cm) =-282.3194
 2nd Lowest Vibrational Mode (1/cm) =                     11.5012
 E(RB-P86) (a.u.) =                                      -4886.52727304
 Thermal correction to Enthalpy (a.u.) =                  0.823210
 Thermal correction to Gibbs Free Energy (a.u.) =         0.687855
 Total Entropy (cal/Kmol) =                               284.878
 E(RPBE1PBE) (a.u.) =                                    -4885.83565579
Optimised cartesian coordinates (Angstrom):
 Fe-3.907357  -1.155776  -0.627233
 Mn0.857615  -0.201704   0.031708
 P     -1.117463   0.953044   0.284955
 O      2.235252   0.281106   2.582879
 O     -0.107656  -2.574659   1.449279
 N      0.249776  -0.957566  -1.827274
 N      1.936334   1.146674  -1.295808
 C     -2.433305   0.260827  -0.821599
 C     -2.246880  -0.821936  -1.787155
 C     -3.436603  -0.880946  -2.605394
 H     -3.625703  -1.598436  -3.413491
 C     -4.354888   0.130564  -2.161100
 H     -5.356906   0.316598  -2.570398
 C     -3.746182   0.830964  -1.066189
 H     -4.196801   1.667674  -0.518492
 C     -3.545964  -2.463383   0.924052
 H     -2.566086  -2.641427   1.386485
 C     -4.082852  -3.164747  -0.212510
 H     -3.590326  -3.978932  -0.761030
 C     -5.370120  -2.595849  -0.521034
 H     -6.027199  -2.895965  -1.348321
 C     -5.630307  -1.543922   0.428315
 H     -6.521672  -0.902692   0.451730
 C     -4.503353  -1.460817   1.320165
 H     -4.382617  -0.744712   2.142991
 C     -1.207856   2.761493  -0.199213
 C     -0.981400   3.780628   0.755436
 H     -0.825516   3.519506   1.813608
 C     -0.964606   5.134647   0.376481
 H     -0.795820   5.910381   1.140535
 C     -1.166399   5.498587  -0.967185
 H     -1.154843   6.559405  -1.263664
 C     -1.389888   4.495624  -1.927418
 H     -1.558683   4.765901  -2.982123
 C     -1.412021   3.142187  -1.545911
 H     -1.619137   2.377308  -2.310536
 C     -1.928348   1.019603   1.969197
 C     -1.422118   0.246919   3.035587
 H     -0.529479  -0.374510   2.886322
 C     -2.047212   0.261373   4.297184
 H     -1.634063  -0.350743   5.114680
 C     -3.186433   1.054251   4.514488
 H     -3.674887   1.067120   5.501831
 C     -3.694065   1.839976   3.462432
 H     -4.581191   2.473461   3.622235
 C     -3.069561   1.824624   2.204081
 H     -3.472324   2.462441   1.403162
 C     -1.033735  -1.727824  -1.928155
 H     -1.006150  -2.412527  -1.057440
 C      0.421788   0.069282  -2.870204
 H      0.449562  -0.364192  -3.892700
 H     -0.447160   0.762877  -2.836961
 C      1.684830   0.842620  -2.606289
```

```
 C      3.044902   1.924920  -1.027764
 C      3.960175   2.275725  -2.062149
 H      4.844556   2.878128  -1.818860
 C      3.707226   1.905280  -3.381114
 C      2.531712   1.192235  -3.666373
 H      2.275434   0.890038  -4.691815
 C     -1.083845  -2.604964  -3.191185
 H     -1.948317  -3.296711  -3.143571
 H     -1.179524  -2.010606  -4.122610
 H     -0.164031  -3.220205  -3.256674
 C      1.673439   0.184073   1.543216
 C      0.234716  -1.596629   0.870021
 H      1.025472  -1.665635  -1.982513
 H      2.177062  -1.181988  -0.229250
 C      3.052817  -2.325393  -1.058791
 C      3.194292  -3.254189   0.119929
 C      4.359819  -1.493463  -1.064818
 C      4.281953  -2.847106   0.924688
 C      2.461820  -4.416381   0.410822
 C      4.902631  -1.573183   0.378263
 H      5.045153  -2.021962  -1.767174
 C      4.640764  -3.606543   2.050676
 C      2.825441  -5.176792   1.536560
 H      1.627821  -4.721123  -0.241107
 H      6.010702  -1.576262   0.438540
 C      3.905449  -4.771557   2.350933
 H      5.489718  -3.306221   2.686923
 H      2.271470  -6.096706   1.784051
 H      4.182464  -5.379207   3.227688
 O      2.380392  -2.588264  -2.099042
 H      4.219535  -0.467953  -1.453406
 H      4.549129  -0.701951   0.974809
 H      4.406682   2.188204  -4.183042
 N      3.273607   2.366297   0.262427
 C      4.574386   2.920298   0.657439
 C      2.233836   3.095877   1.016567
 C      4.409036   3.107276   2.173114
 H      5.394887   2.226355   0.383533
 C      2.919164   3.504148   2.344304
 H      1.914990   3.993397   0.436345
 H      4.610531   2.145732   2.686643
 H      2.464088   2.983273   3.208743
 H      1.342779   2.457111   1.149506
 H      2.804809   4.593820   2.513674
 H      5.112124   3.862395   2.577319
 H      4.776875   3.903109   0.160111
---------------------------------------
Mn13/TS-ii_re
Frequencies, energies and thermodynamic properties:
 Lowest Vibrational Mode (1/cm) =-388.8216
 2nd Lowest Vibrational Mode (1/cm) =                     17.5870
 E(RB-P86) (a.u.) =                                      -4886.52825042
 Thermal correction to Enthalpy (a.u.) =                  0.822780
 Thermal correction to Gibbs Free Energy (a.u.) =         0.689141
 Total Entropy (cal/Kmol) =                               281.267
 E(RPBE1PBE) (a.u.) =                                    -4885.84024773
Optimised cartesian coordinates (Angstrom):
 Fe-4.109901  -1.223359  -0.424710
 Mn0.615394  -0.384826   0.346887
 P     -1.247197   0.886602   0.118482
 O      1.309404   1.023780   2.838129
 O     -0.541863  -2.344745   2.188978
 N      0.097300  -1.431121  -1.392409
 N      1.786650   0.678641  -1.096867
 C     -2.559259   0.053207  -0.868846
 C     -2.392509  -1.226014  -1.554805
 C     -3.555219  -1.419036  -2.391022
 H     -3.751739  -2.290651  -3.027891
 C     -4.439454  -0.298002  -2.221170
 H     -5.417981  -0.171041  -2.703462
 C     -3.837489   0.605130  -1.279788
 H     -4.266403   1.555024  -0.935725
 C     -3.901425  -2.102490   1.428707
 H     -2.966872  -2.168528   2.000486
 C     -4.363632  -3.064551   0.463698
 H     -3.848888  -3.992070   0.178882
 C     -5.608121  -2.586567  -0.082506
 H     -6.204795  -3.082122  -0.860079
 C     -5.916789  -1.328683   0.548714
 H     -6.790750  -0.698313   0.336377
 C     -4.861197  -1.027492   1.480243
 H     -4.787123  -0.129517   2.107152
 C     -0.993661   2.492555  -0.796993
 C     -0.227120   3.494655  -0.153734
 H      0.157280   3.320199   0.864298
```

```
 C      0.041229   4.714244  -0.796064
 H      0.625412   5.488970  -0.274006
 C     -0.431845   4.946405  -2.102299
 H     -0.219533   5.902342  -2.606877
 C     -1.175126   3.950098  -2.757275
 H     -1.548632   4.120387  -3.779812
 C     -1.456492   2.731359  -2.109386
 H     -2.057146   1.970588  -2.631059
 C     -2.171652   1.501961   1.619634
 C     -2.068388   0.822015   2.852691
 H     -1.414881  -0.055891   2.944581
 C     -2.800884   1.254226   3.973426
 H     -2.704570   0.711708   4.927420
 C     -3.647910   2.372636   3.879594
 H     -4.219377   2.711518   4.758330
 C     -3.754922   3.060374   2.656956
 H     -4.411204   3.941364   2.572565
 C     -3.019626   2.631754   1.537821
 H     -3.102498   3.192425   0.593854
 C     -1.207741  -2.174974  -1.434341
 H     -1.262734  -2.683040  -0.451311
 C      0.338097  -0.620064  -2.595764
 H      0.586150  -1.243803  -3.483464
 H     -0.599160  -0.077723  -2.854379
 C      1.421394   0.404775  -2.382489
 C      2.735181   1.663653  -0.900330
 C      3.218997   2.436432  -1.988360
 H      3.956851   3.226913  -1.800792
 C      2.814564   2.151354  -3.293236
 C      1.921935   1.092472  -3.499858
 H      1.579566   0.813406  -4.507120
 C     -1.210761  -3.270811  -2.514233
 H     -2.105340  -3.915908  -2.404793
 H     -1.218029  -2.855310  -3.542730
 H     -0.314439  -3.911198  -2.393391
 C      1.100666   0.493317   1.799176
 C     -0.131197  -1.532837   1.421881
 H      0.845966  -2.189713  -1.366092
 H      1.904677  -1.512477   0.393879
 C      2.623906  -2.922216  -0.129078
 C      4.039629  -2.406305  -0.149062
 C      2.498123  -3.672184   1.222978
 C      4.626831  -2.553839   1.127167
 C      4.781105  -1.944386  -1.248924
 C      3.607829  -3.083764   2.121129
 C      5.981115  -2.230575   1.314219
 C      6.136961  -1.621025  -1.058853
 H      4.303688  -1.860019  -2.238489
 C      6.730768  -1.764112   0.214436
 H      6.458804  -2.350141   2.300706
 H      6.744186  -1.265084  -1.906746
 H      7.796448  -1.516955   0.347797
 O      1.999605  -3.264774  -1.180658
 H      4.041828  -3.816094   2.832971
 H      3.208372  -2.242688   2.733697
 H      1.481489  -3.632406   1.654028
 H      2.721623  -4.738084   0.987055
 H      3.212344   2.731626  -4.140332
 N      3.213747   1.905791   0.383348
 C      3.989051   0.861580   1.095721
 C      3.870612   3.181549   0.694662
 C      4.451202   1.547573   2.404382
 H      4.866272   0.566804   0.472681
 C      4.140453   3.056162   2.202324
 H      3.216148   4.034704   0.422903
 H      3.907851   1.146372   3.281201
 H      3.229238   3.338657   2.766770
 H      5.532813   1.367998   2.568577
 H      3.369651  -0.041338   1.245414
 H      4.837923   3.302096   0.141727
 H      4.963642   3.721337   2.531290
---------------------------------------
Mn13/TS-iii
Frequencies, energies and thermodynamic properties:
 Lowest Vibrational Mode (1/cm) =-563.5502
 2nd Lowest Vibrational Mode (1/cm) =                     21.7208
 E(RB-P86) (a.u.) =                                      -4463.78764548
 Thermal correction to Enthalpy (a.u.) =                  0.668009
 Thermal correction to Gibbs Free Energy (a.u.) =         0.552859
 Total Entropy (cal/Kmol) =                               242.353
 E(RPBE1PBE) (a.u.) =                                    -4463.10329688
Optimised cartesian coordinates (Angstrom):
 Fe3.699687  -0.505508  -0.100479
 Mn-0.940483  -0.129135  -1.390332
 P      0.252992   0.357258   0.479761
```

```
 O     -2.198514   2.548761  -1.429493
 O      0.968967   1.052508  -3.271393
 N     -0.144443  -2.053301  -1.361459
 N     -2.513288  -1.226361  -0.407253
 C      1.767305  -0.677896   0.594847
 C      2.092622  -1.793546  -0.292856
 C      3.232907  -2.467987   0.283162
 H      3.742244  -3.341510  -0.143645
 C      3.625101  -1.787355   1.488666
 H      4.474968  -2.051109   2.132774
 C      2.733344  -0.676757   1.679788
 H      2.770454   0.042841   2.507912
 C      3.885693   0.896456  -1.604485
 H      3.061163   1.305849  -2.200758
 C      4.640019  -0.286633  -1.921410
 H      4.495601  -0.927983  -2.801246
 C      5.601851  -0.497422  -0.869219
 H      6.316227  -1.329153  -0.804018
 C      5.442269   0.559767   0.097609
 H      6.014817   0.673978   1.027963
 C      4.379223   1.419400  -0.354717
 H      3.997905   2.307375   0.166007
 C     -0.630876   0.030193   2.090088
 C     -1.761462   0.831766   2.379097
 H     -2.066580   1.623786   1.675571
 C     -2.495025   0.636127   3.560642
 H     -3.365883   1.276122   3.774713
 C     -2.121564  -0.375809   4.465859
 H     -2.697834  -0.530850   5.391835
 C     -1.010693  -1.187535   4.179508
 H     -0.711533  -1.983372   4.880392
 C     -0.269229  -0.987091   2.999119
 H      0.603231  -1.625807   2.793655
 C      0.876021   2.088783   0.795395
 C      1.055787   2.993639  -0.273670
 H      0.800314   2.694884  -1.299386
 C      1.564996   4.284643  -0.042600
 H      1.695440   4.976592  -0.889872
 C      1.900984   4.692442   1.260368
 H      2.297283   5.704420   1.440440
 C      1.719533   3.801524   2.333895
 H      1.972730   4.111944   3.360294
 C      1.206885   2.512675   2.104340
 H      1.053955   1.835378   2.958897
 C      1.300987  -2.208653  -1.543669
 H      1.583824  -1.517601  -2.365135
 C     -0.684952  -2.845608  -0.279038
 H     -0.726625  -3.941115  -0.513689
 H     -0.058554  -2.788497   0.655214
 C     -2.072733  -2.407468   0.108107
 C     -3.801379  -0.828047  -0.120507
 C     -4.578456  -1.528699   0.836957
 C     -4.083597  -2.703085   1.411616
 C     -2.829653  -3.177375   1.008785
 C      1.691403  -3.628439  -2.011175
 H      2.760760  -3.674041  -2.303796
 H      1.527325  -4.392270  -1.222697
 H      1.082735  -3.907140  -2.895272
 C     -1.762928   1.450674  -1.409086
 C      0.261040   0.573066  -2.449488
 H     -1.138772  -1.420573  -2.461685
 H     -1.651880  -0.758465  -2.824293
 H     -5.589765  -1.177810   1.079371
 H     -2.415407  -4.118815   1.398634
 H     -4.692488  -3.263231   2.138605
 N     -4.332265   0.266233  -0.793200
 C     -4.465015   0.239919  -2.269467
 C     -5.526704   0.945995  -0.277361
 C     -5.218359   1.549243  -2.615604
 H     -5.062041  -0.654296  -2.572815
 C     -5.685803   2.123709  -1.250873
 H     -5.374962   1.254431   0.777071
 H     -4.559405   2.265231  -3.144324
 H     -5.020766   2.953193  -0.937997
 H     -3.471991   0.159397  -2.749280
 H     -6.074929   1.332284  -3.285129
 H     -6.725028   2.507782  -1.271143
 H     -6.432121   0.287029  -0.314077
---------------------------------------
Mn14/i
Frequencies, energies and thermodynamic properties:
 Lowest Vibrational Mode (1/cm) =                          15.4444
 2nd Lowest Vibrational Mode (1/cm) =                      23.8844
 E(RB-P86) (a.u.) =                                     -4588.17349356
 Thermal correction to Enthalpy (a.u.) =                   0.549917
```

```
 Thermal correction to Gibbs Free Energy (a.u.) =         0.438279
 Total Entropy (cal/Kmol) =                               234.961
 E(RPBE1PBE) (a.u.) =                                    -4587.58369917
Optimised cartesian coordinates (Angstrom):
 Fe3.526675  -0.773741   0.273799
 Mn-0.930671  -0.291409  -1.372377
 P      0.188716   0.558862   0.311366
 O     -1.790764   2.306454  -2.512685
 O      1.098098   0.173875  -3.425137
 N     -0.361318  -2.055651  -0.953279
 N     -2.767295  -1.046256  -0.468023
 C      1.557216  -0.562673   0.816728
 C      1.790548  -1.887124   0.250781
 C      2.799445  -2.527668   1.060240
 H      3.218632  -3.528046   0.892157
 C      3.196788  -1.622795   2.107132
 H      3.963335  -1.815684   2.869954
 C      2.442303  -0.408156   1.957052
 H      2.516504   0.478847   2.599455
 C      3.943305   0.160653  -1.515394
 H      3.199916   0.460236  -2.265200
 C      4.599125  -1.118319  -1.450505
 H      4.448645  -1.956872  -2.143722
 C      5.468356  -1.117035  -0.301264
 H      6.093269  -1.954531   0.036851
 C      5.351459   0.166524   0.343084
 H      5.872509   0.478114   1.258367
 C      4.407037   0.955443  -0.404799
 H      4.081903   1.975934  -0.164354
 C     -0.868758   0.636169   1.839657
 C     -1.863599   1.640467   1.916423
 H     -1.949605   2.389784   1.114298
 C     -2.743855   1.691056   3.008879
 H     -3.506359   2.484758   3.058166
 C     -2.656701   0.729459   4.034666
 H     -3.349670   0.768324   4.890095
 C     -1.681544  -0.279513   3.959981
 H     -1.605107  -1.035360   4.757959
 C     -0.790500  -0.327225   2.870398
 H     -0.019538  -1.111922   2.834965
 C      0.954960   2.251545   0.281277
 C      1.297621   2.854082  -0.949099
 H      1.078108   2.341333  -1.896203
 C      1.922180   4.114170  -0.976715
 H      2.180924   4.571611  -1.944838
 C      2.210242   4.789401   0.222835
 H      2.696457   5.777590   0.199357
 C      1.866750   4.199616   1.453085
 H      2.082367   4.723597   2.397911
 C      1.240655   2.941203   1.483166
 H      0.963780   2.499347   2.453132
 C      1.058567  -2.457961  -0.972538
 H      1.493911  -1.977280  -1.870505
 C     -1.145396  -2.807416  -0.007688
 H     -1.250927  -3.884205  -0.291176
 H     -0.671520  -2.836194   1.011263
 C     -2.521175  -2.243533   0.143021
 C     -4.057211  -0.580179  -0.376376
 C     -5.063332  -1.237884   0.341221
 H     -6.072057  -0.808339   0.373859
 C     -4.777213  -2.444697   1.000804
 C     -3.487467  -2.957081   0.879071
 H     -3.202674  -3.912021   1.345940
 C      1.286100  -3.972965  -1.131753
 H      2.356594  -4.177446  -1.335629
 H      1.002768  -4.549946  -0.227769
 H      0.700430  -4.356738  -1.992009
 C     -1.546587   1.261469  -2.016380
 C      0.326480  -0.016570  -2.542491
 H     -5.553781  -2.971049   1.575065
 C     -4.468456   0.687619  -1.119689
 F     -5.805794   0.901943  -1.012943
 F     -3.867920   1.799743  -0.630567
 F     -4.200592   0.604532  -2.444758
---------------------------------------
Mn14/ii
Frequencies, energies and thermodynamic properties:
 Lowest Vibrational Mode (1/cm) =                         16.0329
 2nd Lowest Vibrational Mode (1/cm) =                     19.8584
 E(RB-P86) (a.u.) =                                      -4743.10826970
 Thermal correction to Enthalpy (a.u.) =                  0.634647
 Thermal correction to Gibbs Free Energy (a.u.) =         0.507539
 Total Entropy (cal/Kmol) =                               267.521
 E(RPBE1PBE) (a.u.) =                                    -4742.52715264
Optimised cartesian coordinates (Angstrom):
```

```
 Fe-3.608598   0.906285   0.594102
 Mn0.923558   0.428555  -0.863519
 P     -0.559419  -0.913753   0.034678
 O      1.507459  -1.359969  -3.149639
 O     -0.832455   1.534495  -2.920395
 N      0.580755   1.805913   0.444023
 N      2.707796   0.216816   0.391937
 C     -1.800474   0.025042   1.009319
 C     -1.737589   1.462239   1.258647
 C     -2.743686   1.768984   2.246845
 H     -2.972121   2.767479   2.641153
 C     -3.430273   0.555008   2.602832
 H     -4.264303   0.470206   3.312577
 C     -2.861515  -0.520780   1.837195
 H     -3.169175  -1.573808   1.879080
 C     -3.901914   1.207211  -1.424892
 H     -3.114056   1.220469  -2.188933
 C     -4.360669   2.345357  -0.674132
 H     -3.989471   3.374549  -0.770349
 C     -5.374590   1.894870   0.245084
 H     -5.907492   2.518702   0.975164
 C     -5.544870   0.476240   0.059198
 H     -6.231284  -0.170005   0.622633
 C     -4.633063   0.050017  -0.970308
 H     -4.503608  -0.976961  -1.335601
 C      0.257136  -2.001510   1.302818
 C      1.143626  -3.003334   0.838269
 H      1.286908  -3.150900  -0.243587
 C      1.844331  -3.812038   1.746731
 H      2.523606  -4.593124   1.369804
 C      1.684954  -3.621982   3.133510
 H      2.237894  -4.254829   3.845669
 C      0.818275  -2.620814   3.603261
 H      0.687196  -2.465613   4.686080
 C      0.105834  -1.813931   2.694805
 H     -0.582991  -1.045570   3.077169
 C     -1.549168  -2.139211  -0.949199
 C     -1.823513  -1.903187  -2.314342
 H     -1.424948  -1.008859  -2.813440
 C     -2.609158  -2.808418  -3.050130
 H     -2.811694  -2.611445  -4.114807
 C     -3.128623  -3.960798  -2.433568
 H     -3.740778  -4.670476  -3.012384
 C     -2.855587  -4.206719  -1.075688
 H     -3.252734  -5.109848  -0.585512
 C     -2.068605  -3.304710  -0.338306
 H     -1.849811  -3.518812   0.719515
 C     -0.755380   2.429390   0.587968
 H     -1.112966   2.599624  -0.446888
 C      1.268643   1.676512   1.710555
 H      1.547467   2.662008   2.158057
 H      0.622676   1.189536   2.489311
 C      2.518162   0.866494   1.576688
 C      3.903723  -0.437694   0.243206
 C      4.861657  -0.526229   1.259675
 C      4.620530   0.093762   2.497266
 C      3.440182   0.818899   2.641923
 C     -0.720987   3.809182   1.272762
 H     -1.699629   4.318284   1.162005
 H     -0.497300   3.747067   2.357508
 H      0.051798   4.435900   0.783009
 C      1.365185  -0.676866  -2.196527
 C     -0.180149   1.070843  -2.044300
 H      1.509214   3.097253  -0.573159
 O      1.898213   3.877420  -1.063487
 C      3.292175   3.927596  -0.787152
 H      3.793482   2.961585  -1.047102
 H      3.488738   4.100526   0.302005
 H      3.210033   1.360873   3.571421
 H      5.797837  -1.067898   1.076277
 C      3.924766   5.055569  -1.594494
 H      5.014158   5.124611  -1.395044
 H      3.780464   4.887822  -2.682591
 H      3.461823   6.031203  -1.335698
 H      5.356259   0.027363   3.312334
 C      4.264616  -1.068175  -1.099104
 F      3.535904  -2.179884  -1.364055
 F      4.105653  -0.196918  -2.123809
 F      5.566356  -1.452475  -1.118420
---------------------------------------
Mn14/iii
Frequencies, energies and thermodynamic properties:
 Lowest Vibrational Mode (1/cm) =                        16.5878
 2nd Lowest Vibrational Mode (1/cm) =                    23.7029
 E(RB-P86) (a.u.) =                                    -4744.28241708
```

Thermal correction to Enthalpy (a.u.) = 0.651015
Thermal correction to Gibbs Free Energy (a.u.) = 0.524954
Total Entropy (cal/Kmol) = 265.317
E(RPBE1PBE) (a.u.) = -4743.69783897
Optimised cartesian coordinates (Angstrom):

```
Fe-3.591457   0.907842   0.571507
Mn0.983574   0.424961  -1.075282
P     -0.503916  -0.866399   0.083964
O      1.324324  -1.722530  -3.077361
O     -0.957676   1.423283  -3.028872
N      0.610139   1.958603   0.280523
N      2.669991   0.167543   0.300734
C     -1.745679   0.111457   1.007044
C     -1.718679   1.563878   1.144085
C     -2.711919   1.910944   2.133497
H     -2.961180   2.928105   2.461362
C     -3.356822   0.709480   2.592991
H     -4.173276   0.654741   3.325748
C     -2.775130  -0.403174   1.893664
H     -3.050640  -1.458491   2.018130
C     -3.966443   1.001320  -1.455712
H     -3.213612   0.933297  -2.250649
C     -4.391549   2.213463  -0.808882
H     -4.022912   3.224861  -1.026598
C     -5.366947   1.865460   0.192825
H     -5.868425   2.564085   0.875949
C     -5.546464   0.435811   0.161599
H     -6.210005  -0.144767   0.816521
C     -4.678312  -0.099508  -0.854685
H     -4.564711  -1.159197  -1.117289
C      0.303707  -1.914370   1.395544
C      1.172084  -2.946604   0.967179
H      1.318785  -3.132107  -0.107930
C      1.846606  -3.747397   1.903157
H      2.514020  -4.550323   1.551909
C      1.673382  -3.523347   3.282280
H      2.203767  -4.150602   4.016177
C      0.819374  -2.495313   3.716653
H      0.674755  -2.313360   4.793533
C      0.137345  -1.694723   2.780272
H     -0.538343  -0.903019   3.137824
C     -1.524678  -2.144718  -0.809979
C     -1.863335  -1.976419  -2.170578
H     -1.488967  -1.111126  -2.734237
C     -2.684902  -2.912034  -2.824696
H     -2.934910  -2.765106  -3.887347
C     -3.180499  -4.029473  -2.129871
H     -3.821584  -4.762850  -2.644213
C     -2.846286  -4.208373  -0.775413
H     -3.224523  -5.082579  -0.221932
C     -2.021809  -3.276185  -0.121232
H     -1.758179  -3.440076   0.934996
C     -0.759150   2.507978   0.414878
H     -1.134815   2.631155  -0.621610
C      1.191426   1.648968   1.574776
H      1.395499   2.560577   2.188682
H      0.515660   1.027070   2.225040
C      2.473822   0.884427   1.445158
C      3.862754  -0.493442   0.185795
C      4.833354  -0.508665   1.196526
C      4.600791   0.196205   2.387323
C      3.412589   0.913677   2.498064
C     -0.765485   3.917914   1.046566
H     -1.769871   4.381622   0.964829
H     -0.491733   3.903238   2.122123
H     -0.042943   4.561895   0.506351
C      1.271182  -0.890972  -2.244611
C     -0.229489   1.001345  -2.199068
H      2.260023   1.119775  -1.995467
H      1.921778   1.803235  -1.663752
H      1.452157   3.160640  -0.432712
O      1.944793   3.875636  -1.011970
C      3.223064   4.129742  -0.458011
H      3.148718   4.469247   0.607609
H      3.856999   3.204562  -0.441691
H      3.187838   1.511897   3.393380
H      5.768616  -1.060656   1.043479
C      3.936355   5.204871  -1.273952
H      3.345824   6.145449  -1.281190
H      4.939718   5.427646  -0.854356
H      4.066068   4.877826  -2.327367
H      5.347257   0.195870   3.195496
C      4.210322  -1.241663  -1.101583
F      4.092924  -0.455582  -2.200969
F      5.494543  -1.675242  -1.077781
```

```
 F      3.439253  -2.340507  -1.285716
---------------------------------------
Mn14/iv
Frequencies, energies and thermodynamic properties:
 Lowest Vibrational Mode (1/cm) =                        14.6034
 2nd Lowest Vibrational Mode (1/cm) =                    17.6049
 E(RB-P86) (a.u.) =                                    -4744.30983131
 Thermal correction to Enthalpy (a.u.) =                 0.655426
 Thermal correction to Gibbs Free Energy (a.u.) =        0.528945
 Total Entropy (cal/Kmol) =                              266.202
 E(RPBE1PBE) (a.u.) =                                  -4743.72319061
Optimised cartesian coordinates (Angstrom):
 Fe-3.611445   0.889459   0.593085
 Mn1.023740   0.515097  -0.893229
 P     -0.533102  -0.893611   0.027989
 O      1.502136  -1.237289  -3.200471
 O     -0.691311   1.877659  -2.837429
 N      0.582450   1.932390   0.651958
 N      2.645343   0.124492   0.476822
 C     -1.814045  -0.012519   1.016588
 C     -1.773138   1.412406   1.343058
 C     -2.811032   1.658779   2.318739
 H     -3.060695   2.631041   2.761739
 C     -3.499046   0.424748   2.585055
 H     -4.355567   0.298870   3.260877
 C     -2.896656  -0.602907   1.782281
 H     -3.197495  -1.658253   1.755431
 C     -3.837667   1.281465  -1.417534
 H     -3.024204   1.322014  -2.153387
 C     -4.312178   2.389570  -0.631491
 H     -3.931087   3.419006  -0.670111
 C     -5.359092   1.907795   0.232986
 H     -5.910538   2.503205   0.972957
 C     -5.534161   0.500724  -0.022203
 H     -6.243463  -0.163775   0.489212
 C     -4.592778   0.112337  -1.039772
 H     -4.458568  -0.898426  -1.445921
 C      0.148201  -2.126921   1.253310
 C      1.012266  -3.130333   0.751652
 H      1.201980  -3.196875  -0.330743
 C      1.629264  -4.046134   1.619220
 H      2.291343  -4.824875   1.207814
 C      1.408567  -3.966877   3.007871
 H      1.895227  -4.683143   3.688768
 C      0.563964  -2.966479   3.518083
 H      0.382746  -2.894991   4.602613
 C     -0.063680  -2.053551   2.648034
 H     -0.736477  -1.289050   3.066496
 C     -1.542772  -2.014247  -1.069519
 C     -1.774189  -1.659536  -2.416731
 H     -1.333231  -0.740072  -2.826605
 C     -2.570848  -2.471430  -3.244384
 H     -2.739018  -2.179375  -4.293270
 C     -3.145656  -3.651414  -2.739255
 H     -3.766376  -4.288496  -3.389233
 C     -2.916889  -4.016650  -1.400057
 H     -3.358183  -4.941489  -0.995134
 C     -2.119203  -3.206481  -0.572563
 H     -1.937648  -3.514016   0.469085
 C     -0.821549   2.457404   0.776050
 H     -1.123340   2.674321  -0.267659
 C      1.139319   1.427031   1.914631
 H      1.257306   2.219853   2.685089
 H      0.432883   0.679315   2.338572
 C      2.461631   0.751845   1.681025
 C      3.850785  -0.506390   0.312467
 C      4.848338  -0.540507   1.301018
 C      4.638582   0.102725   2.526385
 C      3.423678   0.767591   2.705576
 C     -0.863718   3.786060   1.549842
 H     -1.869876   4.244029   1.473169
 H     -0.630666   3.661367   2.627066
 H     -0.138648   4.500409   1.109804
 C      1.376995  -0.574426  -2.228347
 C     -0.051921   1.306119  -2.016123
 H      2.093906   1.544698  -1.482528
 H      2.141943   2.990461  -1.239257
 H      1.161682   2.742650   0.340181
 O      2.142286   3.899949  -0.805177
 C      3.501438   4.248488  -0.523715
 H      3.466206   5.116700   0.169766
 H      4.021286   3.424353   0.021655
 H      3.200050   1.306259   3.638284
 H      5.788957  -1.066950   1.099362
 C      4.284188   4.615951  -1.783738
```

```
 H      3.786691   5.446109  -2.327118
 H      5.316392   4.936572  -1.528249
 H      4.359351   3.748943  -2.473944
 H      5.408886   0.091270   3.311376
 C      4.187287  -1.202403  -1.006850
 F      4.180449  -0.351509  -2.058164
 F      5.434734  -1.741982  -0.963171
 F      3.345228  -2.227909  -1.281095
---------------------------------------
Mn14/v
Frequencies, energies and thermodynamic properties:
 Lowest Vibrational Mode (1/cm) =                          15.4382
 2nd Lowest Vibrational Mode (1/cm) =                      25.0592
 E(RB-P86) (a.u.) =                                      -4589.37126594
 Thermal correction to Enthalpy (a.u.) =                   0.570888
 Thermal correction to Gibbs Free Energy (a.u.) =          0.458884
 Total Entropy (cal/Kmol) =                                235.734
 E(RPBE1PBE) (a.u.) =                                    -4588.78034343
Optimised cartesian coordinates (Angstrom):
 Fe3.509055  -0.735553   0.325516
 Mn-1.031195  -0.359235  -1.426270
 P      0.153062   0.560848   0.304056
 O     -1.821273   2.228688  -2.564416
 O      0.971060  -0.196676  -3.556385
 N     -0.345370  -2.296961  -0.828823
 N     -2.749682  -1.058905  -0.336836
 C      1.541832  -0.512848   0.874271
 C      1.800671  -1.868462   0.388389
 C      2.823024  -2.441143   1.235658
 H      3.259933  -3.442173   1.131940
 C      3.210381  -1.465301   2.217363
 H      3.986982  -1.596502   2.982766
 C      2.432615  -0.278449   1.995713
 H      2.494867   0.649231   2.579088
 C      3.890837   0.087712  -1.522790
 H      3.133584   0.329051  -2.279937
 C      4.566423  -1.175717  -1.385112
 H      4.421492  -2.058604  -2.022236
 C      5.450172  -1.088236  -0.250793
 H      6.090676  -1.893955   0.132281
 C      5.323175   0.232279   0.310861
 H      5.850631   0.609090   1.197425
 C      4.358327   0.958635  -0.472740
 H      4.021230   1.987542  -0.293219
 C     -0.807356   0.840507   1.881352
 C     -1.791658   1.858676   1.876761
 H     -1.909441   2.500317   0.989700
 C     -2.618101   2.064472   2.993334
 H     -3.371318   2.868478   2.972512
 C     -2.489440   1.245833   4.132008
 H     -3.139776   1.405571   5.006691
 C     -1.525637   0.223262   4.142806
 H     -1.413976  -0.422922   5.028379
 C     -0.689506   0.022342   3.027213
 H      0.072605  -0.771593   3.064719
 C      0.968266   2.226764   0.094566
 C      1.293027   2.691686  -1.198527
 H      1.035205   2.086542  -2.078979
 C      1.948770   3.923983  -1.373681
 H      2.192910   4.270662  -2.390555
 C      2.287143   4.711894  -0.259049
 H      2.797874   5.678218  -0.397033
 C      1.962188   4.261116   1.033636
 H      2.217342   4.873021   1.913727
 C      1.304922   3.030416   1.208907
 H      1.043697   2.699486   2.226244
 C      1.134082  -2.569932  -0.787221
 H      1.516330  -2.123017  -1.726147
 C     -1.061276  -2.683669   0.396012
 H     -1.023233  -3.776126   0.596408
 H     -0.562264  -2.183962   1.255417
 C     -2.491520  -2.225143   0.338805
 C     -4.057878  -0.646905  -0.320703
 C     -5.086665  -1.352033   0.326974
 H     -6.110144  -0.959527   0.294182
 C     -4.801358  -2.544840   1.000264
 C     -3.474356  -2.983262   0.995064
 H     -3.179758  -3.915649   1.499000
 C      1.439101  -4.076225  -0.831749
 H      2.523865  -4.243174  -0.983620
 H      1.144559  -4.599623   0.100550
 H      0.911673  -4.551840  -1.684316
 C     -1.576688   1.188540  -2.053379
 C      0.208395  -0.251152  -2.647196
 H     -0.721822  -2.846740  -1.613692
```

```
 H     -1.747478  -1.128137  -2.610101
 H     -5.593827  -3.114022   1.507765
 C     -4.475463   0.630444  -1.047944
 F     -5.800755   0.879209  -0.866174
 F     -3.823306   1.724583  -0.587867
 F     -4.287667   0.550249  -2.385395
---------------------------------------
Mn14/vi_R
Frequencies, energies and thermodynamic properties:
 Lowest Vibrational Mode (1/cm) =                        14.9019
 2nd Lowest Vibrational Mode (1/cm) =                    18.3734
 E(RB-P86) (a.u.) =                                     -5012.08332652
 Thermal correction to Enthalpy (a.u.) =                 0.723625
 Thermal correction to Gibbs Free Energy (a.u.) =        0.589231
 Total Entropy (cal/Kmol) =                              282.855
 E(RPBE1PBE) (a.u.) =                                   -5011.48866050
Optimised cartesian coordinates (Angstrom):
 Fe-3.649748  -1.654239  -0.828738
 Mn0.728538   0.020507   0.197705
 P     -1.330040   0.790103   0.405492
 O      1.448417   0.471857   3.032240
 O      0.170477  -2.609043   1.354220
 N      0.421607  -0.536774  -1.649835
 N      1.713154   1.665917  -0.842805
 C     -2.451646   0.011749  -0.823495
 C     -2.026471  -0.919413  -1.864523
 C     -3.151639  -1.113926  -2.747991
 H     -3.172381  -1.772383  -3.625667
 C     -4.260051  -0.331931  -2.268292
 H     -5.262445  -0.291868  -2.715539
 C     -3.839247   0.356130  -1.078723
 H     -4.457269   1.033004  -0.474895
 C     -3.173457  -2.959781   0.694303
 H     -2.232744  -2.965335   1.259096
 C     -3.422583  -3.680221  -0.525732
 H     -2.709582  -4.333174  -1.046872
 C     -4.762757  -3.375735  -0.958062
 H     -5.248599  -3.751152  -1.868655
 C     -5.343478  -2.468331  -0.001077
 H     -6.349885  -2.031945  -0.055209
 C     -4.360755  -2.208263   1.018755
 H     -4.484436  -1.541915   1.882302
 C     -1.427098   2.598354  -0.017995
 C     -0.930479   3.533427   0.921387
 H     -0.559098   3.186287   1.897869
 C     -0.913561   4.904931   0.621646
 H     -0.532173   5.620871   1.366979
 C     -1.378909   5.363336  -0.626066
 H     -1.363821   6.439446  -0.860874
 C     -1.864291   4.441084  -1.568734
 H     -2.233838   4.790643  -2.545940
 C     -1.890384   3.065722  -1.268198
 H     -2.293024   2.358070  -2.008777
 C     -2.272228   0.725090   2.006864
 C     -1.945670  -0.226829   2.997161
 H     -1.101570  -0.914239   2.850232
 C     -2.696286  -0.308393   4.184082
 H     -2.425765  -1.055187   4.947235
 C     -3.781654   0.559386   4.398106
 H     -4.366857   0.495810   5.329166
 C     -4.111152   1.515052   3.419619
 H     -4.955500   2.204179   3.580183
 C     -3.360455   1.600670   2.234351
 H     -3.621082   2.365373   1.486264
 C     -0.627627  -1.531002  -1.988819
 H     -0.536774  -2.323780  -1.220173
 C      0.523044   0.516909  -2.642814
 H      0.926646   0.154456  -3.619202
 H     -0.481086   0.949382  -2.893759
 C      1.395797   1.636455  -2.170559
 C      2.553002   2.675065  -0.442731
 C      3.028227   3.667083  -1.308660
 H      3.694789   4.448113  -0.923290
 C      2.658338   3.644250  -2.663350
 C      1.846150   2.599954  -3.096239
 H      1.543855   2.507693  -4.150010
 C     -0.414338  -2.215903  -3.352878
 H     -1.120571  -3.062610  -3.468523
 H     -0.574387  -1.528786  -4.208863
 H      0.613754  -2.625819  -3.414602
 C      1.213790   0.381623   1.878616
 C      0.331197  -1.541417   0.864494
 H      1.850900  -1.370402  -1.865608
 H      2.792757  -0.916468  -0.090409
 C      3.472649  -1.513693  -0.794275
```

```
 C      3.988137  -2.709997  -0.004008
 C      4.767845  -0.684971  -1.032728
 C      5.265391  -2.436899   0.533248
 C      3.364824  -3.948683   0.210122
 C      5.711907  -1.038080   0.142363
 H      5.198796  -1.046055  -1.992304
 C      5.928385  -3.406915   1.304308
 C      4.031003  -4.921990   0.981035
 H      2.373370  -4.152227  -0.224701
 H      6.788996  -0.983791  -0.122016
 C      5.303500  -4.651501   1.525290
 H      6.928563  -3.205985   1.723178
 H      3.559281  -5.902646   1.155963
 H      5.817355  -5.422714   2.121902
 O      2.761996  -1.831447  -1.956055
 H      4.573155   0.400749  -1.138437
 H      5.559076  -0.335491   0.992523
 H      3.017919   4.417722  -3.357907
 C      3.053784   2.733722   0.999393
 F      3.652879   1.576123   1.374983
 F      3.981688   3.711368   1.151276
 F      2.065356   3.005457   1.883917
---------------------------------------
Mn14/vi_S
Frequencies, energies and thermodynamic properties:
 Lowest Vibrational Mode (1/cm) =                         14.7241
 2nd Lowest Vibrational Mode (1/cm) =                     19.7413
 E(RB-P86) (a.u.) =                                      -5012.08364176
 Thermal correction to Enthalpy (a.u.) =                  0.723633
 Thermal correction to Gibbs Free Energy (a.u.) =         0.589942
 Total Entropy (cal/Kmol) =                               281.377
 E(RPBE1PBE) (a.u.) =                                    -5011.48950004
Optimised cartesian coordinates (Angstrom):
 Fe-3.979713  -1.395063  -0.716933
 Mn0.545210  -0.243808   0.399125
 P     -1.358651   0.864864   0.242541
 O      1.115782   0.674742   3.152897
 O     -0.532201  -2.462195   1.972308
 N      0.267383  -1.101224  -1.340413
 N      1.879861   0.971533  -0.827943
 C     -2.518385   0.033140  -0.912707
 C     -2.191992  -1.143349  -1.712772
 C     -3.275454  -1.337433  -2.646914
 H     -3.351595  -2.148589  -3.382129
 C     -4.263864  -0.314133  -2.432059
 H     -5.214765  -0.212410  -2.972301
 C     -3.809006   0.527540  -1.359468
 H     -4.341021   1.398820  -0.955926
 C     -3.830566  -2.435887   1.056880
 H     -2.940426  -2.484724   1.696654
 C     -4.132870  -3.331331  -0.027765
 H     -3.518169  -4.181024  -0.354024
 C     -5.370749  -2.903441  -0.627895
 H     -5.862245  -3.365935  -1.494330
 C     -5.835485  -1.743624   0.089889
 H     -6.743575  -1.168007  -0.134271
 C     -4.882604  -1.452166   1.129264
 H     -4.935660  -0.618247   1.841107
 C     -1.105436   2.536881  -0.531805
 C     -0.466332   3.535733   0.241314
 H     -0.188144   3.327205   1.285859
 C     -0.187027   4.795570  -0.311504
 H      0.303432   5.564892   0.305853
 C     -0.528362   5.073666  -1.649392
 H     -0.306800   6.061717  -2.083023
 C     -1.154183   4.084854  -2.427202
 H     -1.427961   4.294376  -3.473537
 C     -1.443441   2.822826  -1.873300
 H     -1.952938   2.066112  -2.488896
 C     -2.395961   1.288690   1.726048
 C     -2.320620   0.511481   2.902446
 H     -1.616760  -0.329359   2.968221
 C     -3.144792   0.803068   4.004381
 H     -3.071078   0.188233   4.915329
 C     -4.053501   1.874606   3.946708
 H     -4.696159   2.103343   4.811596
 C     -4.131667   2.658019   2.780858
 H     -4.835655   3.503689   2.727600
 C     -3.306955   2.370319   1.679424
 H     -3.368654   3.002798   0.780276
 C     -0.909796  -1.971414  -1.586324
 H     -1.001572  -2.599687  -0.678219
 C      0.593264  -0.275580  -2.489836
 H      0.962055  -0.869870  -3.360309
 H     -0.306237   0.270260  -2.879072
```

```
 C      1.634587   0.744222  -2.151645
 C      2.874193   1.871126  -0.539963
 C      3.573844   2.581748  -1.522689
 H      4.355979   3.290468  -1.224672
 C      3.276945   2.369485  -2.878687
 C      2.306313   1.421115  -3.190122
 H      2.048093   1.186644  -4.233562
 C     -0.724656  -2.938983  -2.771617
 H     -1.549409  -3.679590  -2.792825
 H     -0.715705  -2.420988  -3.752522
 H      0.225998  -3.497797  -2.657220
 C      0.959946   0.379951   2.020201
 C     -0.162503  -1.552358   1.306612
 H      1.569078  -2.125976  -1.299593
 H      2.419267  -1.475872   0.466776
 C      2.979474  -2.372092   0.015189
 C      2.919818  -3.529400   1.053486
 C      4.084770  -3.253505   2.035675
 O      2.445810  -2.659938  -1.242381
 H      3.811890   2.922414  -3.664935
 C      4.456894  -2.009169   0.012404
 C      5.180561  -1.297359  -0.955997
 C      5.089540  -2.508622   1.172351
 C      6.557281  -1.077082  -0.751188
 H      4.677310  -0.934923  -1.866964
 C      6.461377  -2.285376   1.378021
 C      7.191628  -1.565383   0.409703
 H      7.144610  -0.526835  -1.504207
 H      6.967027  -2.676344   2.276667
 H      8.270122  -1.392025   0.556391
 H      1.929892  -3.619391   1.542504
 H      3.746395  -2.602770   2.874870
 H      3.110268  -4.469892   0.490984
 H      4.503811  -4.171265   2.499102
 C      3.295031   2.117106   0.908311
 F      4.395320   2.909527   0.964302
 F      2.334292   2.749619   1.625560
 F      3.617051   0.968703   1.548224
---------------------------------------
Mn14/viii
Frequencies, energies and thermodynamic properties:
 Lowest Vibrational Mode (1/cm) =                       15.5632
 2nd Lowest Vibrational Mode (1/cm) =                   20.2296
 E(RB-P86) (a.u.) =                                    -4589.33885495
 Thermal correction to Enthalpy (a.u.) =                0.566031
 Thermal correction to Gibbs Free Energy (a.u.) =       0.452827
 Total Entropy (cal/Kmol) =                             238.259
 E(RPBE1PBE) (a.u.) =                                  -4588.74921735
Optimised cartesian coordinates (Angstrom):
 Fe3.549441  -0.710809   0.262500
 Mn-0.972763  -0.269706  -1.507630
 P      0.155700   0.487586   0.311732
 O     -1.725234   2.478252  -2.307996
 O      1.184677   0.227281  -3.422999
 N     -0.323524  -2.139864  -1.000560
 N     -2.724268  -1.023948  -0.423400
 C      1.565103  -0.578855   0.794704
 C      1.846709  -1.884096   0.209167
 C      2.872681  -2.496581   1.019408
 H      3.327608  -3.479605   0.841657
 C      3.236453  -1.591656   2.078828
 H      4.006844  -1.766501   2.842224
 C      2.442545  -0.400982   1.938823
 H      2.483164   0.481808   2.590175
 C      3.971080   0.286425  -1.494563
 H      3.233600   0.606365  -2.240912
 C      4.632992  -0.990095  -1.468423
 H      4.491581  -1.805511  -2.190464
 C      5.494197  -1.022307  -0.313412
 H      6.121721  -1.867116   0.000959
 C      5.365825   0.238849   0.372559
 H      5.879642   0.522671   1.300906
 C      4.421638   1.046774  -0.354886
 H      4.090016   2.057521  -0.084196
 C     -0.897409   0.537108   1.848885
 C     -1.980562   1.447604   1.872891
 H     -2.156585   2.115634   1.015771
 C     -2.836319   1.511469   2.984684
 H     -3.670983   2.230460   2.989047
 C     -2.631443   0.658006   4.085661
 H     -3.303836   0.706823   4.956858
 C     -1.564409  -0.256247   4.066218
 H     -1.394903  -0.927614   4.923180
 C     -0.701191  -0.317808   2.955173
 H      0.136483  -1.031392   2.959979
```

```
 C      0.879765   2.205953   0.323565
 C      1.288736   2.819726  -0.880888
 H      1.143881   2.307680  -1.842078
 C      1.888151   4.092031  -0.868298
 H      2.197553   4.555843  -1.818366
 C      2.087405   4.770719   0.347095
 H      2.554195   5.768472   0.355131
 C      1.680298   4.169999   1.551921
 H      1.826836   4.694248   2.509771
 C      1.077881   2.899508   1.540635
 H      0.751803   2.450254   2.491403
 C      1.103064  -2.470198  -1.000535
 H      1.531442  -1.996940  -1.908468
 C     -1.020130  -2.698532   0.124706
 H     -1.106918  -3.816768   0.081856
 H     -0.520606  -2.515924   1.124248
 C     -2.412596  -2.163032   0.259435
 C     -4.022129  -0.592867  -0.340092
 C     -4.989942  -1.218304   0.455350
 C     -4.641131  -2.356783   1.200744
 C     -3.340713  -2.839260   1.080025
 C      1.371565  -3.985871  -1.140625
 H      2.447327  -4.182949  -1.328552
 H      1.083246  -4.554757  -0.232117
 H      0.794147  -4.386861  -1.998550
 C     -1.519588   1.368839  -1.963490
 C      0.364399   0.029511  -2.592980
 H     -1.561728  -1.405705  -2.722131
 H     -2.018327  -0.746524  -2.879145
 H     -6.012380  -0.821858   0.479813
 H     -3.015913  -3.747187   1.610022
 H     -5.382775  -2.859836   1.838891
 C     -4.488867   0.603627  -1.166904
 F     -5.838304   0.733907  -1.111979
 F     -3.975474   1.777269  -0.721356
 F     -4.171042   0.476772  -2.479302
---------------------------------------
Mn14/ix
Frequencies, energies and thermodynamic properties:
 Lowest Vibrational Mode (1/cm) =                        21.5893
 2nd Lowest Vibrational Mode (1/cm) =                    26.8059
 E(RB-P86) (a.u.) =                                     -4743.12597927
 Thermal correction to Enthalpy (a.u.) =                 0.635491
 Thermal correction to Gibbs Free Energy (a.u.) =        0.513645
 Total Entropy (cal/Kmol) =                              256.447
 E(RPBE1PBE) (a.u.) =                                   -4742.54223503
Optimised cartesian coordinates (Angstrom):
 Fe-3.638536  -0.592139  -0.662016
 Mn0.997122  -0.819886   0.830295
 P     -0.381454   0.796624   0.014985
 O      1.745245   0.734243   3.212053
 O     -0.973903  -1.911339   2.691863
 N      0.397970  -2.077393  -0.768900
 N      2.650205  -0.450831  -0.650595
 C     -1.735744   0.094385  -1.028935
 C     -1.867123  -1.309511  -1.417674
 C     -2.914474  -1.385557  -2.410286
 H     -3.274903  -2.301142  -2.895815
 C     -3.439852  -0.065732  -2.630812
 H     -4.264082   0.194348  -3.308464
 C     -2.726090   0.844945  -1.779695
 H     -2.895563   1.927220  -1.714302
 C     -3.948972  -0.992007   1.336446
 H     -3.160487  -1.132471   2.086528
 C     -4.522912  -2.028705   0.520017
 H     -4.255560  -3.093712   0.546353
 C     -5.492612  -1.421774  -0.355614
 H     -6.089303  -1.941044  -1.117524
 C     -5.519482  -0.008254  -0.077503
 H     -6.140982   0.737768  -0.590733
 C     -4.563785   0.258350   0.965934
 H     -4.328983   1.242015   1.392863
 C      0.410563   2.015287  -1.159529
 C      1.244664   3.022027  -0.617819
 H      1.351010   3.116308   0.473658
 C      1.933480   3.912526  -1.457371
 H      2.571248   4.694624  -1.015156
 C      1.812073   3.806537  -2.856108
 H      2.354124   4.503861  -3.514442
 C      0.990851   2.807215  -3.405578
 H      0.882342   2.718227  -4.498427
 C      0.293127   1.919525  -2.564127
 H     -0.364534   1.160054  -3.014495
 C     -1.286238   1.960750   1.159493
 C     -1.509962   1.605662   2.507786
```

```
 H     -1.121379   0.657042   2.901692
 C     -2.233775   2.456417   3.363357
 H     -2.395429   2.161624   4.412440
 C     -2.744040   3.676301   2.885847
 H     -3.308504   4.342816   3.557172
 C     -2.521638   4.043487   1.545976
 H     -2.911007   4.999840   1.161668
 C     -1.796115   3.195220   0.691483
 H     -1.617063   3.507597  -0.348887
 C     -1.033309  -2.477845  -0.905395
 H     -1.353920  -2.712978   0.129058
 C      1.040070  -1.705180  -2.032750
 H      1.124611  -2.559934  -2.738782
 H      0.403842  -0.946476  -2.541809
 C      2.406741  -1.110334  -1.815901
 C      3.893358   0.086548  -0.503167
 C      4.889916   0.015058  -1.489316
 C      4.621263  -0.655776  -2.689892
 C      3.362872  -1.235753  -2.844414
 C     -1.216270  -3.754527  -1.743685
 H     -2.261368  -4.115476  -1.667450
 H     -0.992960  -3.596029  -2.818359
 H     -0.556759  -4.560577  -1.362249
 C      1.519449   0.134918   2.218426
 C     -0.216857  -1.442387   1.906607
 H      0.959325  -2.822050  -0.283375
 H      3.100833  -1.792733  -3.756170
 H      5.869057   0.475076  -1.308556
 H      5.386349  -0.731540  -3.476835
 O      2.141227  -2.455105   1.035444
 C      2.450179  -3.025425   2.270973
 C      3.364597  -4.244831   2.089439
 H      1.534754  -3.353702   2.838845
 H      2.967516  -2.292697   2.947235
 H      3.625646  -4.703233   3.067869
 H      2.868981  -5.020602   1.466584
 H      4.308308  -3.954746   1.579661
 C      4.276802   0.779330   0.805121
 F      4.310375  -0.086777   1.844313
 F      5.519005   1.323963   0.717751
 F      3.439313   1.792364   1.123938
---------------------------------------
Mn14/x
Frequencies, energies and thermodynamic properties:
 Lowest Vibrational Mode (1/cm) =                        13.5023
 2nd Lowest Vibrational Mode (1/cm) =                    19.2502
 E(RB-P86) (a.u.) =                                     -4898.06655427
 Thermal correction to Enthalpy (a.u.) =                 0.719668
 Thermal correction to Gibbs Free Energy (a.u.) =        0.584604
 Total Entropy (cal/Kmol) =                              284.267
 E(RPBE1PBE) (a.u.) =                                   -4897.49314481
Optimised cartesian coordinates (Angstrom):
 Fe-3.666799   1.226045   0.580787
 Mn0.932298   0.234704  -0.838670
 P     -0.829181  -0.919646   0.054249
 O      1.135634  -1.801109  -2.952570
 O     -0.686181   1.685940  -2.796007
 N      0.631791   1.730152   0.671964
 N      2.438678  -0.383766   0.688292
 C     -1.985494   0.133629   1.028202
 C     -1.777904   1.549483   1.320949
 C     -2.778015   1.940369   2.287695
 H     -2.909377   2.946035   2.706259
 C     -3.604383   0.801806   2.583721
 H     -4.468417   0.792357   3.261595
 C     -3.128410  -0.308762   1.806783
 H     -3.551160  -1.321496   1.808474
 C     -3.859775   1.578343  -1.440934
 H     -3.053900   1.498815  -2.181560
 C     -4.195224   2.759356  -0.690540
 H     -3.696420   3.735040  -0.764887
 C     -5.284892   2.431997   0.192971
 H     -5.757754   3.111680   0.914498
 C     -5.625229   1.047507  -0.014640
 H     -6.403186   0.487763   0.521683
 C     -4.742995   0.518365  -1.021817
 H     -4.729295  -0.514540  -1.392843
 C     -0.355419  -2.237217   1.289989
 C      0.237266  -3.425775   0.801551
 H      0.342781  -3.579926  -0.283146
 C      0.684458  -4.420479   1.686241
 H      1.136784  -5.341695   1.285570
 C      0.557490  -4.241921   3.076970
 H      0.909977  -5.021592   3.770718
 C     -0.026471  -3.064004   3.573380
```

```
 H     -0.138214  -2.915889   4.659399
 C     -0.482428  -2.069499   2.686924
 H     -0.960506  -1.166247   3.096408
 C     -1.978928  -1.887984  -1.054214
 C     -2.124153  -1.549031  -2.417130
 H     -1.528979  -0.735918  -2.853271
 C     -3.034203  -2.242250  -3.236074
 H     -3.130125  -1.963202  -4.297396
 C     -3.813580  -3.285548  -2.706556
 H     -4.524390  -3.828915  -3.349172
 C     -3.673869  -3.635184  -1.351068
 H     -4.274824  -4.454512  -0.925217
 C     -2.761783  -2.945640  -0.533309
 H     -2.654389  -3.245413   0.520493
 C     -0.691446   2.442425   0.743956
 H     -0.942103   2.661943  -0.312515
 C      1.084765   1.211455   1.972351
 H      1.278428   2.018813   2.711294
 H      0.281773   0.577549   2.408696
 C      2.321136   0.368903   1.817703
 C      3.553861  -1.161404   0.587144
 C      4.547395  -1.217850   1.578291
 C      4.413774  -0.437744   2.733938
 C      3.282817   0.370557   2.847456
 C     -0.574012   3.791891   1.473042
 H     -1.507529   4.376723   1.351168
 H     -0.389525   3.674579   2.560504
 H      0.256536   4.378586   1.031978
 C      1.123488  -1.010445  -2.074889
 C     -0.077421   1.079180  -1.979615
 H      2.622412   2.685362  -0.803694
 H      1.326713   2.465555   0.375978
 O      2.544324   3.590254  -0.259941
 C      3.722104   3.775144   0.517279
 H      3.463921   3.781611   1.605568
 H      4.424102   2.920127   0.368325
 H      3.127686   1.014213   3.725706
 H      5.418995  -1.868600   1.438190
 C      4.427343   5.087606   0.170834
 H      3.741285   5.949419   0.312652
 H      5.315930   5.246391   0.818708
 H      4.765540   5.094345  -0.886979
 H      5.182532  -0.459625   3.520616
 O      2.561744   1.352121  -1.395690
 C      2.911537   1.455857  -2.758376
 C      4.312524   2.053972  -2.929243
 H      2.178897   2.084877  -3.332937
 H      2.892918   0.451275  -3.245021
 H      4.587335   2.114193  -4.004398
 H      4.366513   3.079671  -2.507333
 H      5.070240   1.428025  -2.412773
 C      3.771772  -2.045440  -0.643320
 F      3.953141  -1.331714  -1.776075
 F      4.882687  -2.814013  -0.492369
 F      2.742418  -2.900919  -0.843866
---------------------------------------
Mn14/TS-i
Frequencies, energies and thermodynamic properties:
 Lowest Vibrational Mode (1/cm) =-789.3013
 2nd Lowest Vibrational Mode (1/cm) =                     17.8444
 E(RB-P86) (a.u.) =                                     -4744.27962730
 Thermal correction to Enthalpy (a.u.) =                 0.647128
 Thermal correction to Gibbs Free Energy (a.u.) =        0.523213
 Total Entropy (cal/Kmol) =                              260.802
 E(RPBE1PBE) (a.u.) =                                   -4743.69213810
Optimised cartesian coordinates (Angstrom):
 Fe-3.597904   0.860864   0.586390
 Mn0.990280   0.469794  -1.045715
 P     -0.488089  -0.867238   0.074381
 O      1.392065  -1.605277  -3.103859
 O     -0.942449   1.481802  -3.001361
 N      0.579459   1.986734   0.342319
 N      2.668925   0.218019   0.337522
 C     -1.745596   0.079621   1.014819
 C     -1.740858   1.530324   1.180966
 C     -2.742308   1.846058   2.172593
 H     -3.006653   2.853280   2.518657
 C     -3.371308   0.627149   2.606127
 H     -4.190065   0.547488   3.333888
 C     -2.770626  -0.463679   1.889081
 H     -3.032233  -1.524697   1.992836
 C     -3.959536   0.984899  -1.440854
 H     -3.199249   0.942472  -2.230575
 C     -4.408724   2.178557  -0.775843
 H     -4.054632   3.199391  -0.972717
```

```
 C     -5.386184   1.797638   0.211583
 H     -5.903605   2.476031   0.903123
 C     -5.542903   0.366296   0.153631
 H     -6.201786  -0.236205   0.793232
 C     -4.658711  -0.137285  -0.865004
 H     -4.526034  -1.190321  -1.144854
 C      0.316711  -1.933982   1.372782
 C      1.185401  -2.960611   0.931449
 H      1.326915  -3.135995  -0.146024
 C      1.866459  -3.767938   1.856904
 H      2.533499  -4.566564   1.495240
 C      1.700551  -3.556005   3.238899
 H      2.236372  -4.188222   3.964529
 C      0.846528  -2.533765   3.686492
 H      0.707054  -2.361348   4.765625
 C      0.157253  -1.727230   2.760486
 H     -0.520146  -0.942094   3.129394
 C     -1.492961  -2.134143  -0.852567
 C     -1.812326  -1.944525  -2.215005
 H     -1.434179  -1.067684  -2.757917
 C     -2.619686  -2.872896  -2.896706
 H     -2.855128  -2.709210  -3.960238
 C     -3.119567  -4.004308  -2.228125
 H     -3.749475  -4.731917  -2.764027
 C     -2.803928  -4.204723  -0.872123
 H     -3.185553  -5.090182  -0.339231
 C     -1.993933  -3.279697  -0.190376
 H     -1.744064  -3.460450   0.866494
 C     -0.807648   2.509872   0.473344
 H     -1.170773   2.633834  -0.566853
 C      1.169515   1.662201   1.636772
 H      1.342782   2.563492   2.270436
 H      0.493595   1.008883   2.245004
 C      2.470884   0.931044   1.484679
 C      3.871548  -0.423430   0.215897
 C      4.851783  -0.416696   1.218350
 C      4.620555   0.291100   2.406616
 C      3.419315   0.986059   2.526581
 C     -0.837567   3.911076   1.117003
 H     -1.848856   4.358177   1.033830
 H     -0.566561   3.892743   2.192877
 H     -0.126591   4.572691   0.582739
 C      1.310603  -0.803175  -2.244577
 C     -0.215065   1.054500  -2.172711
 H      2.216278   1.268357  -1.904436
 H      1.935107   2.021772  -1.587512
 H      1.280876   2.910914  -0.269354
 O      1.887437   3.614585  -1.044415
 C      3.173483   3.963877  -0.589805
 H      3.167424   4.222340   0.502645
 H      3.894754   3.105898  -0.688031
 H      3.192240   1.583692   3.421591
 H      5.794032  -0.954819   1.059479
 C      3.729651   5.155669  -1.373969
 H      3.063143   6.037894  -1.266641
 H      4.743339   5.439549  -1.017925
 H      3.798584   4.914754  -2.456265
 H      5.375573   0.309715   3.206515
 C      4.221866  -1.172776  -1.070656
 F      4.118480  -0.385566  -2.168457
 F      5.503010  -1.616942  -1.037576
 F      3.444370  -2.266269  -1.259159
---------------------------------------
Mn14/TS-ii_si
Frequencies, energies and thermodynamic properties:
 Lowest Vibrational Mode (1/cm) =-306.1076
 2nd Lowest Vibrational Mode (1/cm) =                      14.1203
 E(RB-P86) (a.u.) =                                      -5012.07677015
 Thermal correction to Enthalpy (a.u.) =                   0.720026
 Thermal correction to Gibbs Free Energy (a.u.) =          0.589171
 Total Entropy (cal/Kmol) =                                275.409
 E(RPBE1PBE) (a.u.) =                                    -5011.47569683
Optimised cartesian coordinates (Angstrom):
 Fe-3.694990  -1.288209  -0.806923
 Mn0.920560  -0.107050   0.182198
 P     -1.108641   0.915407   0.382665
 O      1.743413   0.211473   2.986097
 O      0.090652  -2.701009   1.255831
 N      0.466718  -0.648430  -1.789601
 N      1.929276   1.489500  -0.873607
 C     -2.333818   0.248523  -0.824177
 C     -2.033741  -0.721653  -1.876141
 C     -3.193711  -0.795034  -2.735297
 H     -3.304289  -1.444392  -3.612671
 C     -4.202387   0.095652  -2.229753
```

```
 H     -5.205672   0.238555  -2.653039
 C     -3.682951   0.733566  -1.052620
 H     -4.213026   1.469513  -0.434770
 C     -3.313256  -2.653031   0.690048
 H     -2.358464  -2.767018   1.219143
 C     -3.684929  -3.328922  -0.524835
 H     -3.068480  -4.051704  -1.076178
 C     -4.997832  -2.873931  -0.905106
 H     -5.554754  -3.183613  -1.799593
 C     -5.439122  -1.917853   0.078301
 H     -6.391727  -1.371480   0.063845
 C     -4.397653  -1.778744   1.062670
 H     -4.414990  -1.110353   1.933242
 C     -1.143849   2.741281  -0.004998
 C     -0.643827   3.647301   0.960513
 H     -0.320283   3.275784   1.944595
 C     -0.566769   5.022146   0.683307
 H     -0.182749   5.711470   1.452274
 C     -0.976541   5.517718  -0.569001
 H     -0.914616   6.595835  -0.786365
 C     -1.469429   4.626879  -1.537819
 H     -1.800013   5.002973  -2.519262
 C     -1.554390   3.249676  -1.258064
 H     -1.968538   2.573258  -2.021742
 C     -2.031698   0.881531   2.003138
 C     -1.736022  -0.104932   2.968846
 H     -0.934520  -0.833141   2.785570
 C     -2.462457  -0.171319   4.172042
 H     -2.215311  -0.947581   4.913596
 C     -3.494063   0.748497   4.429245
 H     -4.060654   0.697646   5.372606
 C     -3.793086   1.739747   3.476381
 H     -4.595274   2.469825   3.669646
 C     -3.066144   1.808106   2.275180
 H     -3.302945   2.600997   1.548979
 C     -0.738951  -1.502008  -2.061436
 H     -0.692396  -2.293348  -1.287485
 C      0.562008   0.522571  -2.669967
 H      0.729212   0.245837  -3.733335
 H     -0.400940   1.080095  -2.637762
 C      1.653080   1.447833  -2.214892
 C      2.857784   2.422749  -0.486882
 C      3.520036   3.274980  -1.385003
 H      4.252999   3.995908  -1.003609
 C      3.243842   3.194750  -2.755470
 C      2.291485   2.262421  -3.167558
 H      2.023310   2.151710  -4.228531
 C     -0.653652  -2.196951  -3.431464
 H     -1.446085  -2.966312  -3.521603
 H     -0.773057  -1.489724  -4.277472
 H      0.324614  -2.708497  -3.534432
 C      1.444681   0.176316   1.842449
 C      0.370971  -1.641452   0.803129
 H      1.321120  -1.243107  -2.008728
 H      2.328213  -0.968148  -0.085839
 C      3.341250  -1.840505  -1.049605
 C      3.370625  -3.062828  -0.166031
 C      4.607103  -1.035858  -0.657085
 C      4.347487  -2.905938   0.841643
 C      2.643923  -4.256575  -0.302285
 C      4.990846  -1.533585   0.753691
 H      5.386533  -1.325989  -1.398557
 C      4.598541  -3.955576   1.741167
 C      2.899860  -5.307414   0.596473
 H      1.899322  -4.362362  -1.107512
 H      6.086120  -1.562960   0.930601
 C      3.868549  -5.154737   1.612310
 H      5.361597  -3.851926   2.530251
 H      2.348399  -6.257249   0.507233
 H      4.062458  -5.987482   2.307685
 O      2.830352  -1.822473  -2.209973
 H      4.467728   0.058027  -0.736014
 H      4.558420  -0.866736   1.531394
 H      3.760034   3.846151  -3.475833
 C      3.240612   2.578385   0.985544
 F      2.173669   2.808100   1.783160
 F      3.898739   1.493053   1.463003
 F      4.077851   3.633961   1.158061
---------------------------------------
Mn14/TS-ii_re
Frequencies, energies and thermodynamic properties:
 Lowest Vibrational Mode (1/cm) =-299.5630
 2nd Lowest Vibrational Mode (1/cm) =                    14.1983
 E(RB-P86) (a.u.) =                                    -5012.07806069
 Thermal correction to Enthalpy (a.u.) =                 0.719956
```

Thermal correction to Gibbs Free Energy (a.u.) = 0.589064
Total Entropy (cal/Kmol) = 275.485
E(RPBE1PBE) (a.u.) = -5011.47904484

Optimised cartesian coordinates (Angstrom):

```
Fe-4.037577  -1.130653  -0.652638
Mn0.657053  -0.414340   0.394224
P     -1.197201   0.896523   0.214430
O      1.340019   0.425250   3.126926
O     -0.582464  -2.580651   1.922458
N      0.207177  -1.304917  -1.447341
N      1.947671   0.736566  -0.896693
C     -2.462229   0.160660  -0.905903
C     -2.270775  -1.052869  -1.698959
C     -3.394348  -1.156803  -2.601847
H     -3.566720  -1.965065  -3.323542
C     -4.277142  -0.045295  -2.373142
H     -5.230584   0.135762  -2.887288
C     -3.713875   0.763015  -1.327849
H     -4.150949   1.683973  -0.921310
C     -3.926201  -2.144180   1.138367
H     -3.024946  -2.249758   1.755493
C     -4.326653  -3.033980   0.080703
H     -3.790098  -3.936549  -0.241619
C     -5.542326  -2.521110  -0.497592
H     -6.091160  -2.959852  -1.341658
C     -5.894926  -1.314316   0.206134
H     -6.760295  -0.672844  -0.007987
C     -4.895086  -1.079051   1.215209
H     -4.863680  -0.229600   1.909727
C     -0.910035   2.571289  -0.556315
C     -0.241485   3.551920   0.215091
H      0.024688   3.337533   1.261353
C      0.080187   4.802528  -0.336984
H      0.592929   5.555503   0.282727
C     -0.247418   5.092575  -1.675330
H      0.007173   6.072761  -2.108756
C     -0.903580   4.123357  -2.453001
H     -1.169474   4.340384  -3.499984
C     -1.234717   2.872171  -1.898076
H     -1.770058   2.135763  -2.517149
C     -2.179403   1.367532   1.727747
C     -2.104745   0.579408   2.896754
H     -1.437014  -0.292144   2.932180
C     -2.881898   0.896758   4.025830
H     -2.808560   0.270785   4.929346
C     -3.743491   2.007648   4.004209
H     -4.349603   2.257082   4.889611
C     -3.820577   2.803639   2.846545
H     -4.487600   3.680149   2.820436
C     -3.042684   2.488553   1.718718
H     -3.103487   3.131755   0.827114
C     -1.101253  -2.024387  -1.614314
H     -1.205966  -2.618168  -0.685170
C      0.504260  -0.375775  -2.545082
H      0.683041  -0.896983  -3.510774
H     -0.375308   0.287368  -2.706406
C      1.689500   0.486456  -2.217440
C      3.006996   1.567676  -0.641634
C      3.782334   2.163110  -1.649077
H      4.611477   2.824501  -1.371098
C      3.498577   1.899215  -2.994752
C      2.440422   1.033986  -3.273715
H      2.172579   0.773903  -4.308473
C     -1.063326  -3.018923  -2.787251
H     -1.962171  -3.666877  -2.770194
H     -1.029990  -2.515016  -3.774726
H     -0.174241  -3.674445  -2.692873
C      1.119435   0.167495   1.993978
C     -0.138629  -1.683882   1.281748
H      0.965371  -2.052127  -1.469772
H      1.958325  -1.461297   0.400572
C      2.809217  -2.727336  -0.245402
C      4.154951  -2.088793  -0.025638
C      2.608754  -3.658880   0.977452
C      4.598463  -2.341609   1.291549
C      4.960218  -1.408454  -0.953473
C      3.537045  -3.094123   2.074383
C      5.869579  -1.903224   1.696095
C      6.233188  -0.970969  -0.545432
H      4.598587  -1.244025  -1.981467
C      6.681915  -1.216880   0.770498
H      6.235044  -2.098012   2.717961
H      6.889879  -0.443168  -1.255716
H      7.684445  -0.874918   1.074976
O      2.313156  -2.948227  -1.390955
```

```
 H      3.963616  -3.871130   2.741952
 H      2.980886  -2.381523   2.725526
 H      1.550161  -3.768738   1.275899
 H      2.969575  -4.659587   0.645384
 H      4.097770   2.351567  -3.798608
 C      3.413081   1.887487   0.797744
 F      4.492471   2.713694   0.822146
 F      2.433523   2.529778   1.480559
 F      3.762617   0.784782   1.494895
---------------------------------------
Mn14/TS-iii
Frequencies, energies and thermodynamic properties:
 Lowest Vibrational Mode (1/cm) =-708.8647
 2nd Lowest Vibrational Mode (1/cm) =                      18.6776
 E(RB-P86) (a.u.) =                                      -4589.33433876
 Thermal correction to Enthalpy (a.u.) =                   0.564963
 Thermal correction to Gibbs Free Energy (a.u.) =          0.453343
 Total Entropy (cal/Kmol) =                                234.925
 E(RPBE1PBE) (a.u.) =                                    -4588.74169728
Optimised cartesian coordinates (Angstrom):
 Fe3.521323  -0.709366   0.277388
 Mn-0.957766  -0.335017  -1.488636
 P      0.140687   0.527572   0.317066
 O     -1.771522   2.266112  -2.623935
 O      1.205416   0.115044  -3.406466
 N     -0.342509  -2.244208  -0.928403
 N     -2.724279  -1.034466  -0.384206
 C      1.539476  -0.556835   0.810681
 C      1.820568  -1.881652   0.256809
 C      2.846550  -2.472260   1.085071
 H      3.300216  -3.460105   0.934185
 C      3.213453  -1.541207   2.118664
 H      3.985900  -1.696901   2.883937
 C      2.419943  -0.355461   1.948678
 H      2.464696   0.544767   2.575279
 C      3.940723   0.289694  -1.479108
 H      3.200261   0.629952  -2.212845
 C      4.574647  -1.001486  -1.469804
 H      4.406043  -1.808663  -2.195241
 C      5.450789  -1.059445  -0.327363
 H      6.064398  -1.919344  -0.026781
 C      5.358892   0.199810   0.368087
 H      5.891108   0.466262   1.291182
 C      4.422740   1.032767  -0.341596
 H      4.116205   2.048620  -0.060237
 C     -0.886398   0.643393   1.869690
 C     -1.959195   1.566642   1.871915
 H     -2.134786   2.205713   0.992855
 C     -2.805634   1.678421   2.987137
 H     -3.631698   2.407279   2.973258
 C     -2.603244   0.859742   4.114594
 H     -3.268574   0.945496   4.988353
 C     -1.548128  -0.068612   4.116934
 H     -1.380796  -0.714233   4.993937
 C     -0.694281  -0.177382   3.002335
 H      0.133086  -0.902902   3.025068
 C      0.891626   2.232552   0.266808
 C      1.253814   2.814788  -0.967838
 H      1.062250   2.281098  -1.909025
 C      1.863594   4.081750  -1.010188
 H      2.136769   4.520940  -1.982822
 C      2.118224   4.786504   0.179690
 H      2.592969   5.779941   0.145040
 C      1.755122   4.218324   1.414329
 H      1.944010   4.764463   2.352357
 C      1.143029   2.953344   1.457985
 H      0.850205   2.530177   2.431446
 C      1.094011  -2.541085  -0.925631
 H      1.499680  -2.099746  -1.859348
 C     -1.037242  -2.703574   0.248490
 H     -1.097494  -3.819149   0.314665
 H     -0.530036  -2.395564   1.207240
 C     -2.437763  -2.166997   0.321632
 C     -4.017244  -0.590190  -0.329102
 C     -5.008360  -1.199070   0.452930
 C     -4.689158  -2.336666   1.209345
 C     -3.390505  -2.832215   1.121228
 C      1.385592  -4.056034  -0.996955
 H      2.464504  -4.245881  -1.171928
 H      1.099915  -4.587554  -0.065467
 H      0.818963  -4.502120  -1.839302
 C     -1.536016   1.223481  -2.125912
 C      0.387783  -0.068512  -2.569707
 H     -1.184469  -1.844589  -2.185860
 H     -1.674875  -1.253426  -2.723436
```

```
 H     -6.025238  -0.788325   0.456739
 H     -3.087549  -3.739814   1.664113
 H     -5.449724  -2.829409   1.833109
 C     -4.456559   0.603349  -1.176535
 F     -3.881225   1.764454  -0.776693
 F     -4.182923   0.422845  -2.489444
 F     -5.797543   0.794321  -1.086185
---------------------------------------
Mn15/i
Frequencies, energies and thermodynamic properties:
 Lowest Vibrational Mode (1/cm) =                         21.1061
 2nd Lowest Vibrational Mode (1/cm) =                     28.6252
 E(RB-P86) (a.u.) =                                      -4408.50473128
 Thermal correction to Enthalpy (a.u.) =                  0.655558
 Thermal correction to Gibbs Free Energy (a.u.) =         0.540472
 Total Entropy (cal/Kmol) =                               242.218
 E(RPBE1PBE) (a.u.) =                                    -4407.81976494
Optimised cartesian coordinates (Angstrom):
 Fe3.551857  -0.985258   0.185621
 Mn-1.074407  -0.355064  -0.982972
 P      0.399053   0.715267   0.229277
 O     -1.967802   2.255623  -2.063631
 O      0.590075  -0.678864  -3.358979
 N     -0.588983  -1.981624  -0.075484
 N     -3.007763  -0.965503  -0.063876
 C      1.735986  -0.344881   0.893885
 C      1.743098  -1.794970   0.760680
 C      2.814184  -2.296690   1.586679
 H      3.104312  -3.349818   1.694841
 C      3.469240  -1.181550   2.221025
 H      4.335831  -1.240261   2.893580
 C      2.815747   0.025633   1.791119
 H      3.081461   1.046331   2.095881
 C      3.726310  -0.728452  -1.852216
 H      2.891495  -0.581786  -2.550332
 C      4.275769  -1.997480  -1.455685
 H      3.939182  -2.983597  -1.803126
 C      5.328542  -1.750917  -0.502919
 H      5.931216  -2.515354   0.005647
 C      5.432098  -0.326257  -0.313455
 H      6.128588   0.185113   0.364561
 C      4.440366   0.306067  -1.144265
 H      4.248144   1.384383  -1.216031
 C     -0.468748   1.311579   1.766986
 C     -1.466301   2.308340   1.625645
 H     -1.666669   2.749478   0.636182
 C     -2.198683   2.747964   2.739663
 H     -2.963271   3.531234   2.614890
 C     -1.960341   2.188259   4.010397
 H     -2.538577   2.530900   4.883154
 C     -0.980367   1.192486   4.158884
 H     -0.785004   0.751794   5.149584
 C     -0.235736   0.756144   3.045685
 H      0.543083  -0.009414   3.180544
 C      1.259503   2.253894  -0.355770
 C      1.512965   2.446751  -1.731610
 H      1.173341   1.701736  -2.465578
 C      2.203845   3.588447  -2.175742
 H      2.392021   3.725836  -3.252407
 C      2.647510   4.552546  -1.252601
 H      3.185468   5.447931  -1.602415
 C      2.393967   4.371408   0.119116
 H      2.731989   5.124254   0.848970
 C      1.701583   3.231789   0.565451
 H      1.496030   3.110756   1.640622
 C      0.755189  -2.575774  -0.114899
 H      1.095319  -2.465722  -1.164712
 C     -1.312748  -2.218683   1.151302
 H     -1.515398  -3.305336   1.341005
 H     -0.748930  -1.879902   2.062223
 C     -2.651920  -1.530226   1.123643
 C     -4.311725  -0.582705  -0.250371
 C     -5.183299  -0.527494   0.856780
 H     -6.212144  -0.167067   0.719626
 C     -4.767798  -0.966696   2.122770
 C     -3.498495  -1.539852   2.245410
 H     -3.151740  -1.986064   3.189550
 C      0.777110  -4.084857   0.197239
 H      1.770902  -4.513373  -0.044610
 H      0.570043  -4.303635   1.265095
 H      0.021547  -4.610719  -0.421469
 C     -1.666740   1.182448  -1.654674
 C     -0.045397  -0.515550  -2.366934
 H     -5.449885  -0.909416   2.985235
 C     -4.892647  -0.343308  -1.668273
```

```
 C     -5.164037   1.164246  -1.907658
 C     -3.985943  -0.926943  -2.772230
 C     -6.252000  -1.097060  -1.768991
 H     -5.837267   1.576301  -1.126972
 H     -4.232613   1.759363  -1.912144
 H     -5.664584   1.303432  -2.889524
 H     -3.874087  -2.025033  -2.653141
 H     -4.442153  -0.734914  -3.765437
 H     -2.969655  -0.486188  -2.760988
 H     -6.626911  -1.028427  -2.811412
 H     -6.141558  -2.172251  -1.516641
 H     -7.034917  -0.666368  -1.113245
---------------------------------------
Mn15/ii
Frequencies, energies and thermodynamic properties:
 Lowest Vibrational Mode (1/cm) =                          19.2294
 2nd Lowest Vibrational Mode (1/cm) =                      21.3857
 E(RB-P86) (a.u.) =                                       -4563.44119795
 Thermal correction to Enthalpy (a.u.) =                   0.740081
 Thermal correction to Gibbs Free Energy (a.u.) =          0.610460
 Total Entropy (cal/Kmol) =                                272.811
 E(RPBE1PBE) (a.u.) =                                     -4562.76691909
Optimised cartesian coordinates (Angstrom):
 Fe-3.532445   1.328030   0.425868
 Mn0.937121   0.061094  -0.826531
 P     -0.798846  -0.945834   0.057970
 O      1.364686  -2.341409  -2.502496
 O     -0.493857   1.282053  -3.058032
 N      0.754300   1.533171   0.451965
 N      2.855423   0.001242   0.245274
 C     -1.922064   0.178288   0.961934
 C     -1.633277   1.588424   1.190580
 C     -2.628212   2.082305   2.111827
 H     -2.709509   3.114122   2.476846
 C     -3.525007   1.007276   2.447813
 H     -4.398543   1.079303   3.109859
 C     -3.101715  -0.167802   1.735350
 H     -3.580776  -1.154730   1.775589
 C     -3.656757   1.586339  -1.615675
 H     -2.840237   1.424963  -2.331467
 C     -3.942915   2.820558  -0.934388
 H     -3.387841   3.761972  -1.043968
 C     -5.069386   2.599446  -0.063527
 H     -5.520575   3.340837   0.609502
 C     -5.482097   1.226695  -0.210000
 H     -6.303305   0.738992   0.332229
 C     -4.607569   0.598865  -1.166443
 H     -4.646035  -0.450127  -1.487241
 C     -0.169051  -2.092967   1.385210
 C      0.620934  -3.200186   0.987326
 H      0.802954  -3.391671  -0.082001
 C      1.167691  -4.067520   1.946462
 H      1.771378  -4.929386   1.620454
 C      0.947591  -3.836362   3.318274
 H      1.379783  -4.515731   4.069976
 C      0.172236  -2.736578   3.722826
 H     -0.008485  -2.550647   4.793551
 C     -0.385579  -1.869104   2.763870
 H     -1.007278  -1.023795   3.095063
 C     -1.917284  -2.062241  -0.917638
 C     -2.112174  -1.850869  -2.300190
 H     -1.577045  -1.042105  -2.817810
 C     -2.994557  -2.668610  -3.028671
 H     -3.133967  -2.492182  -4.106999
 C     -3.690942  -3.708434  -2.386933
 H     -4.379276  -4.349939  -2.959700
 C     -3.497710  -3.930283  -1.011373
 H     -4.033283  -4.746730  -0.501305
 C     -2.613958  -3.116078  -0.281363
 H     -2.459860  -3.312463   0.791210
 C     -0.469357   2.354666   0.553920
 H     -0.755727   2.574880  -0.495117
 C      1.354218   1.204562   1.735154
 H      1.730312   2.102705   2.289594
 H      0.627309   0.725146   2.441599
 C      2.529124   0.279823   1.538304
 C      4.058098  -0.593418  -0.030363
 C      4.812219  -1.146358   1.024898
 C      4.400899  -0.991600   2.357566
 C      3.273523  -0.208448   2.625435
 C     -0.235464   3.716858   1.236814
 H     -1.127859   4.365350   1.123895
 H     -0.027080   3.619598   2.322380
 H      0.621194   4.222680   0.748742
 C      1.250806  -1.360110  -1.847132
```

```
 C      0.028910   0.756623  -2.129917
 H      1.971528   2.688972  -0.350341
 O      2.533057   3.430539  -0.736333
 C      3.581050   3.735353   0.173279
 H      4.230033   2.842773   0.362115
 H      3.176119   4.046264   1.170183
 H      2.959726   0.023634   3.654058
 H      5.750791  -1.674113   0.804839
 C      4.430642   4.867499  -0.394841
 H      5.252323   5.134907   0.301502
 H      4.881534   4.575028  -1.366471
 H      3.812123   5.773669  -0.566030
 H      4.988935  -1.433971   3.176627
 C      4.677886  -0.537320  -1.449294
 C      4.753200  -1.953102  -2.075918
 C      3.929483   0.435908  -2.384047
 C      6.128216   0.012935  -1.302543
 H      5.330501  -2.650113  -1.433170
 H      3.747229  -2.383724  -2.238800
 H      5.267117  -1.901035  -3.059109
 H      3.821894   1.440917  -1.926967
 H      4.493779   0.537910  -3.334184
 H      2.908423   0.084875  -2.627518
 H      6.577018   0.128576  -2.311016
 H      6.134532   1.007915  -0.810678
 H      6.789311  -0.662719  -0.724071
---------------------------------------
Mn15/iii
Frequencies, energies and thermodynamic properties:
 Lowest Vibrational Mode (1/cm) =                        18.8520
 2nd Lowest Vibrational Mode (1/cm) =                    21.6694
 E(RB-P86) (a.u.) =                                     -4564.60852954
 Thermal correction to Enthalpy (a.u.) =                 0.757253
 Thermal correction to Gibbs Free Energy (a.u.) =        0.628958
 Total Entropy (cal/Kmol) =                              270.019
 E(RPBE1PBE) (a.u.) =                                   -4563.93224651
Optimised cartesian coordinates (Angstrom):
 Fe-3.546464   1.045863   0.625598
 Mn1.001941   0.326425  -1.082608
 P     -0.585153  -0.895718   0.062802
 O      1.297787  -1.793742  -3.125648
 O     -0.893870   1.417967  -3.018920
 N      0.696970   1.901914   0.232750
 N      2.862871   0.101995   0.342790
 C     -1.732672   0.165995   1.023844
 C     -1.631958   1.617570   1.134687
 C     -2.576906   2.029155   2.146376
 H     -2.766377   3.062710   2.462633
 C     -3.265070   0.868843   2.645419
 H     -4.060361   0.865233   3.403041
 C     -2.757197  -0.281797   1.951280
 H     -3.078306  -1.319164   2.108990
 C     -3.950418   1.137173  -1.394226
 H     -3.212144   1.027593  -2.198478
 C     -4.313580   2.372305  -0.752441
 H     -3.905502   3.365068  -0.985094
 C     -5.286823   2.075049   0.267614
 H     -5.747475   2.800598   0.951447
 C     -5.527524   0.654191   0.252474
 H     -6.205387   0.108284   0.922394
 C     -4.699780   0.073285  -0.772546
 H     -4.635975  -0.992847  -1.026052
 C      0.062720  -2.080788   1.351315
 C      0.569512  -3.335046   0.936753
 H      0.527915  -3.622607  -0.125432
 C      1.106653  -4.236658   1.871165
 H      1.487873  -5.211885   1.528932
 C      1.153479  -3.898118   3.236050
 H      1.574237  -4.605116   3.968448
 C      0.651674  -2.655041   3.659333
 H      0.672876  -2.383175   4.726687
 C      0.108380  -1.753848   2.725183
 H     -0.302223  -0.796007   3.079583
 C     -1.729470  -2.026911  -0.887459
 C     -1.950192  -1.854344  -2.270903
 H     -1.411004  -1.078222  -2.828833
 C     -2.866106  -2.670746  -2.960153
 H     -3.019722  -2.519795  -4.040378
 C     -3.577392  -3.672463  -2.278116
 H     -4.292863  -4.311994  -2.818883
 C     -3.363580  -3.856381  -0.899654
 H     -3.910737  -4.641158  -0.353449
 C     -2.445902  -3.044417  -0.211849
 H     -2.281803  -3.217139   0.862559
 C     -0.652152   2.502825   0.365889
```

```
 H     -1.036922   2.607913  -0.669017
 C      1.267058   1.561480   1.523913
 H      1.348364   2.449715   2.194251
 H      0.639886   0.830565   2.103986
 C      2.632380   0.947711   1.391300
 C      4.109541  -0.484534   0.252284
 C      5.107313  -0.189848   1.206280
 C      4.863334   0.680945   2.273438
 C      3.598249   1.255162   2.368350
 C     -0.601548   3.933025   0.948401
 H     -1.594273   4.422461   0.873933
 H     -0.301839   3.948854   2.016866
 H      0.124823   4.535964   0.367782
 C      1.249625  -0.988791  -2.264474
 C     -0.183196   0.953139  -2.195661
 H      2.270353   0.986828  -2.024112
 H      1.990220   1.673248  -1.646228
 H      1.573886   3.062487  -0.488559
 O      2.084203   3.742325  -1.099069
 C      3.330556   4.069575  -0.512040
 H      3.201672   4.458382   0.531288
 H      3.994512   3.170516  -0.423351
 H      3.340178   1.950122   3.180776
 H      6.095897  -0.652082   1.113288
 C      4.037526   5.128589  -1.354402
 H      3.417613   6.047104  -1.428993
 H      5.017211   5.405483  -0.911555
 H      4.216546   4.756117  -2.385324
 H      5.651455   0.904132   3.009255
 C      4.457567  -1.494396  -0.872116
 C      3.438526  -2.656150  -0.827957
 C      4.501569  -0.778079  -2.246465
 C      5.858426  -2.129136  -0.679005
 H      3.624309  -3.292479   0.061686
 H      2.403566  -2.282309  -0.754612
 H      3.522017  -3.289274  -1.734907
 H      5.274215   0.018851  -2.243101
 H      4.763612  -1.506080  -3.042226
 H      3.537904  -0.317552  -2.522532
 H      6.013230  -2.883108  -1.477901
 H      6.681025  -1.389708  -0.765252
 H      5.954770  -2.654723   0.293086
---------------------------------------
Mn15/iv
Frequencies, energies and thermodynamic properties:
 Lowest Vibrational Mode (1/cm) =                        15.0944
 2nd Lowest Vibrational Mode (1/cm) =                    21.7003
 E(RB-P86) (a.u.) =                                    -4564.63929512
 Thermal correction to Enthalpy (a.u.) =                 0.761796
 Thermal correction to Gibbs Free Energy (a.u.) =        0.633354
 Total Entropy (cal/Kmol) =                              270.328
 E(RPBE1PBE) (a.u.) =                                  -4563.96767800
Optimised cartesian coordinates (Angstrom):
 Fe-3.562580   1.002664   0.674551
 Mn1.037549   0.435537  -0.921766
 P     -0.598120  -0.919223  -0.011047
 O      1.478744  -1.257666  -3.282473
 O     -0.656930   1.872106  -2.814628
 N      0.677750   1.861750   0.628813
 N      2.831415   0.044141   0.542996
 C     -1.792353   0.024838   1.037392
 C     -1.681051   1.446362   1.360548
 C     -2.672877   1.737699   2.370886
 H     -2.864399   2.719972   2.820639
 C     -3.401825   0.533891   2.663726
 H     -4.238007   0.443332   3.369990
 C     -2.869951  -0.518374   1.843836
 H     -3.214218  -1.560278   1.835646
 C     -3.823047   1.404554  -1.328426
 H     -3.025003   1.411020  -2.082208
 C     -4.234186   2.530411  -0.531529
 H     -3.813478   3.543869  -0.579771
 C     -5.278021   2.089994   0.358440
 H     -5.788129   2.706650   1.110608
 C     -5.514773   0.691098   0.108053
 H     -6.237615   0.055084   0.636489
 C     -4.614739   0.266245  -0.932189
 H     -4.529482  -0.749558  -1.339069
 C     -0.070379  -2.279119   1.161271
 C      0.450669  -3.481322   0.625128
 H      0.458811  -3.637289  -0.465230
 C      0.942204  -4.493369   1.466832
 H      1.332532  -5.425111   1.026902
 C      0.934747  -4.320275   2.863644
 H      1.321099  -5.113105   3.523610
```

```
 C      0.424009  -3.129652   3.409113
 H      0.403955  -2.984553   4.501179
 C     -0.076680  -2.119831   2.565947
 H     -0.498404  -1.208518   3.017886
 C     -1.723859  -1.885001  -1.151525
 C     -1.859319  -1.510947  -2.505965
 H     -1.274346  -0.672473  -2.906619
 C     -2.744900  -2.198388  -3.356832
 H     -2.833597  -1.890643  -4.410948
 C     -3.509511  -3.271783  -2.868002
 H     -4.200988  -3.810801  -3.535036
 C     -3.380298  -3.656474  -1.520554
 H     -3.970575  -4.498982  -1.125893
 C     -2.493583  -2.971631  -0.671735
 H     -2.394847  -3.298803   0.374674
 C     -0.702775   2.445252   0.762926
 H     -1.012680   2.657603  -0.279262
 C      1.223892   1.339880   1.888451
 H      1.265646   2.112400   2.686795
 H      0.549164   0.538414   2.259806
 C      2.596617   0.757362   1.684893
 C      4.073384  -0.539102   0.409082
 C      5.058154  -0.377287   1.409223
 C      4.810311   0.372327   2.562542
 C      3.548388   0.951296   2.700744
 C     -0.680972   3.787980   1.512702
 H     -1.671964   4.279839   1.448782
 H     -0.429060   3.675475   2.586965
 H      0.059588   4.466597   1.043167
 C      1.350665  -0.626949  -2.286798
 C     -0.024542   1.264660  -2.010837
 H      2.116142   1.464267  -1.491906
 H      2.210696   2.933410  -1.297594
 H      1.283268   2.650047   0.314682
 O      2.239338   3.834870  -0.853440
 C      3.612259   4.166059  -0.619967
 H      3.612121   5.014796   0.097969
 H      4.146541   3.323341  -0.119308
 H      3.283940   1.553014   3.582714
 H      6.039821  -0.846275   1.277983
 C      4.344651   4.563954  -1.900715
 H      3.832220   5.413348  -2.398570
 H      5.389316   4.869393  -1.679598
 H      4.383474   3.717143  -2.618394
 H      5.586981   0.499923   3.332266
 C      4.423910  -1.385463  -0.837027
 C      3.379578  -2.518451  -0.973697
 C      4.490958  -0.450900  -2.073017
 C      5.807005  -2.072122  -0.720970
 H      3.534278  -3.275438  -0.176983
 H      2.349992  -2.132988  -0.872365
 H      3.471242  -3.021393  -1.957884
 H      5.374611   0.217262  -1.992432
 H      4.593831  -1.047541  -3.003048
 H      3.589025   0.183667  -2.154019
 H      5.966828  -2.691750  -1.627507
 H      6.644537  -1.345987  -0.672358
 H      5.873383  -2.747979   0.156867
---------------------------------------
Mn15/v
Frequencies, energies and thermodynamic properties:
 Lowest Vibrational Mode (1/cm) =                          22.0640
 2nd Lowest Vibrational Mode (1/cm) =                      27.1831
 E(RB-P86) (a.u.) =                                      -4409.70154806
 Thermal correction to Enthalpy (a.u.) =                   0.677201
 Thermal correction to Gibbs Free Energy (a.u.) =          0.563308
 Total Entropy (cal/Kmol) =                                239.708
 E(RPBE1PBE) (a.u.) =                                    -4409.02447052
Optimised cartesian coordinates (Angstrom):
 Fe3.483871  -0.863062   0.335928
 Mn-1.060079  -0.330332  -1.404858
 P      0.204345   0.630829   0.269882
 O     -1.753115   2.205232  -2.717451
 O      0.953711  -0.349040  -3.515695
 N     -0.470083  -2.262677  -0.704333
 N     -2.954055  -1.037099  -0.217676
 C      1.534298  -0.503103   0.877915
 C      1.713930  -1.891150   0.453092
 C      2.705663  -2.483795   1.322430
 H      3.083861  -3.511936   1.261825
 C      3.152036  -1.489506   2.259350
 H      3.922870  -1.630551   3.028861
 C      2.441800  -0.271650   1.986472
 H      2.558165   0.673982   2.531014
 C      3.902586  -0.166407  -1.556421
```

```
 H      3.154941   0.067545  -2.325681
 C      4.516710  -1.451039  -1.345106
 H      4.327919  -2.361378  -1.929986
 C      5.404946  -1.341159  -0.216112
 H      6.006315  -2.153383   0.213861
 C      5.342531   0.014272   0.268125
 H      5.888969   0.415876   1.132044
 C      4.413130   0.739993  -0.557807
 H      4.125964   1.792491  -0.437373
 C     -0.637318   1.100864   1.875350
 C     -1.397245   2.294650   1.924579
 H     -1.406812   2.974405   1.057747
 C     -2.128406   2.638512   3.074181
 H     -2.702806   3.578659   3.092796
 C     -2.126615   1.789279   4.196829
 H     -2.700850   2.058010   5.097645
 C     -1.381198   0.598158   4.159691
 H     -1.364386  -0.072117   5.034136
 C     -0.641293   0.258868   3.011149
 H     -0.042348  -0.665275   3.016749
 C      1.144287   2.209242  -0.085807
 C      1.416710   2.586476  -1.418657
 H      1.045435   1.971456  -2.249369
 C      2.167335   3.743268  -1.699636
 H      2.367080   4.020010  -2.747194
 C      2.657317   4.543222  -0.652596
 H      3.242944   5.449911  -0.873127
 C      2.388760   4.179667   0.680039
 H      2.763448   4.800345   1.509785
 C      1.636819   3.025365   0.960280
 H      1.424390   2.766465   2.008989
 C      0.995789  -2.604937  -0.682007
 H      1.381290  -2.213630  -1.643983
 C     -1.168215  -2.547976   0.557080
 H     -1.079400  -3.612611   0.863411
 H     -0.687752  -1.944958   1.357323
 C     -2.622567  -2.169985   0.471081
 C     -4.285464  -0.678446  -0.229944
 C     -5.252634  -1.464984   0.434661
 H     -6.304507  -1.158585   0.408446
 C     -4.899272  -2.629910   1.121350
 C     -3.550877  -2.989134   1.135673
 H     -3.201110  -3.894299   1.653661
 C      1.230386  -4.124375  -0.674384
 H      2.304888  -4.345923  -0.829602
 H      0.922519  -4.601316   0.278284
 H      0.673223  -4.605249  -1.504685
 C     -1.535228   1.195977  -2.132043
 C      0.183844  -0.328099  -2.607808
 H     -0.893223  -2.839824  -1.444850
 H     -1.795095  -1.153421  -2.536030
 H     -5.661062  -3.241363   1.628987
 C     -4.754356   0.592172  -0.975303
 C     -3.990292   1.808828  -0.400306
 C     -4.534524   0.379812  -2.495597
 C     -6.261867   0.881287  -0.774333
 H     -4.355072   2.036107   0.623051
 H     -2.905934   1.609956  -0.333834
 H     -4.144761   2.705687  -1.034233
 H     -5.255483  -0.373665  -2.878483
 H     -4.699157   1.327976  -3.048023
 H     -3.511359   0.014763  -2.706192
 H     -6.512457   1.820333  -1.309896
 H     -6.911074   0.084945  -1.193422
 H     -6.528273   1.029419   0.292928
---------------------------------------
Mn15/vi_R
Frequencies, energies and thermodynamic properties:
 Lowest Vibrational Mode (1/cm) =                         11.4372
 2nd Lowest Vibrational Mode (1/cm) =                     17.3241
 E(RB-P86) (a.u.) =                                     -4832.41187903
 Thermal correction to Enthalpy (a.u.) =                  0.830952
 Thermal correction to Gibbs Free Energy (a.u.) =         0.693855
 Total Entropy (cal/Kmol) =                               288.545
 E(RPBE1PBE) (a.u.) =                                   -4831.72545563
Optimised cartesian coordinates (Angstrom):
 Fe-3.021084  -2.428325  -0.939657
 Mn0.556159   0.470721   0.417856
 P     -1.642554   0.614811   0.354410
 O      0.797995   1.225003   3.272453
 O      0.620260  -2.135916   1.728814
 N      0.671326  -0.262851  -1.367409
 N      1.401189   2.286499  -0.747596
 C     -2.327710  -0.498394  -0.939248
 C     -1.524926  -1.327655  -1.832203
```

C	-2.418677	-1.875159	-2.824964
H	-2.136381	-2.559345	-3.635150
C	-3.752560	-1.406830	-2.557826
H	-4.654658	-1.669319	-3.126865
C	-3.705031	-0.564890	-1.394280
H	-4.562009	-0.053421	-0.937797
C	-2.414645	-3.461773	0.736300
H	-1.587891	-3.171198	1.396933
C	-2.301466	-4.291380	-0.433808
H	-1.378220	-4.749150	-0.813787
C	-3.606556	-4.395517	-1.035248
H	-3.851758	-4.942539	-1.955482
C	-4.528144	-3.631822	-0.232921
H	-5.599039	-3.495653	-0.435139
C	-3.791945	-3.052194	0.860541
H	-4.201069	-2.397892	1.641239
C	-2.215543	2.291952	-0.212659
C	-2.324309	3.346232	0.725352
H	-2.145465	3.152387	1.794746
C	-2.679659	4.639876	0.309100
H	-2.772206	5.445664	1.054551
C	-2.917977	4.905498	-1.052734
H	-3.195292	5.920541	-1.378443
C	-2.806950	3.866634	-1.993225
H	-2.999688	4.062705	-3.059990
C	-2.461182	2.567568	-1.577415
H	-2.405637	1.758918	-2.321981
C	-2.719662	0.322892	1.842542
C	-2.238976	-0.414520	2.946294
H	-1.205719	-0.786307	2.957857
C	-3.075520	-0.684907	4.044758
H	-2.682091	-1.259911	4.897898
C	-4.402916	-0.222348	4.056276
H	-5.056214	-0.433169	4.917734
C	-4.890002	0.518224	2.963525
H	-5.926584	0.891130	2.964684
C	-4.055018	0.791833	1.866682
H	-4.448637	1.387130	1.028441
C	-0.010225	-1.532399	-1.727984
H	0.177053	-2.218595	-0.878887
C	0.668434	0.682732	-2.464205
H	1.268383	0.320572	-3.334049
H	-0.363105	0.830664	-2.880158
C	1.212440	2.023483	-2.072097
C	1.998321	3.491160	-0.418348
C	2.314031	4.430276	-1.418167
C	2.057058	4.170831	-2.771776
C	1.516063	2.933224	-3.104076
H	1.325244	2.645757	-4.148954
C	0.563049	-2.223251	-2.981548
H	0.143516	-3.245452	-3.073726
H	0.325144	-1.680296	-3.919053
H	1.663577	-2.312387	-2.888674
C	0.720367	1.029437	2.108010
C	0.530908	-1.090803	1.172749
H	2.407559	-0.778077	-1.355723
H	3.494879	-0.563564	0.447243
C	4.112006	-1.023358	-0.370746
C	4.656508	-2.353155	0.145464
C	5.410032	-0.189529	-0.561737
C	5.973972	-2.195384	0.629220
C	4.019298	-3.602525	0.186915
C	6.422221	-0.752720	0.465719
H	5.765726	-0.392127	-1.595871
C	6.665459	-3.294068	1.167729
C	4.713563	-4.705142	0.724091
H	2.994199	-3.717458	-0.200954
H	7.481039	-0.658115	0.145054
C	6.027524	-4.550756	1.211729
H	7.696606	-3.181822	1.542381
H	4.230402	-5.695147	0.759594
H	6.562815	-5.421388	1.624330
O	3.335337	-1.122458	-1.546941
H	5.238405	0.901795	-0.470929
H	6.338748	-0.214892	1.438372
H	2.300390	4.916672	-3.544155
H	2.775147	5.383343	-1.135583
C	2.346167	3.825104	1.049081
C	1.022994	4.039493	1.827137
C	3.246241	2.699791	1.623063
C	3.158095	5.136990	1.184751
H	0.556808	4.997142	1.515235
H	0.294318	3.235261	1.624575
H	1.207208	4.078299	2.920139
H	4.254319	2.753375	1.161048

```
 H      3.362411   2.815131   2.720011
 H      2.834213   1.692781   1.422762
 H      3.398908   5.290878   2.256907
 H      4.119180   5.099318   0.631327
 H      2.590741   6.028941   0.848363
---------------------------------------
Mn15/vi_S
Frequencies, energies and thermodynamic properties:
 Lowest Vibrational Mode (1/cm) =                         14.8718
 2nd Lowest Vibrational Mode (1/cm) =                     17.9458
 E(RB-P86) (a.u.) =                                     -4832.41310544
 Thermal correction to Enthalpy (a.u.) =                  0.830877
 Thermal correction to Gibbs Free Energy (a.u.) =         0.693131
 Total Entropy (cal/Kmol) =                               289.910
 E(RPBE1PBE) (a.u.) =                                   -4831.72559755
Optimised cartesian coordinates (Angstrom):
 Fe-3.542694  -2.040033  -0.866663
 Mn0.469585   0.240399   0.472965
 P     -1.658414   0.777355   0.293696
 O      0.695312   1.181662   3.273028
 O      0.005512  -2.233231   1.949386
 N      0.499981  -0.623720  -1.260909
 N      1.728845   1.748737  -0.761496
 C     -2.501613  -0.274539  -0.954415
 C     -1.840138  -1.297011  -1.757982
 C     -2.788292  -1.736857  -2.753583
 H     -2.613265  -2.515308  -3.507021
 C     -4.019349  -1.013819  -2.574095
 H     -4.936292  -1.144238  -3.164625
 C     -3.852748  -0.117872  -1.462823
 H     -4.613011   0.572262  -1.075068
 C     -3.190761  -3.064972   0.886482
 H     -2.342250  -2.899844   1.562888
 C     -3.207782  -3.971149  -0.230930
 H     -2.379761  -4.618544  -0.549660
 C     -4.491935  -3.861758  -0.875006
 H     -4.812550  -4.407125  -1.772762
 C     -5.270841  -2.889080  -0.151552
 H     -6.289866  -2.564466  -0.401502
 C     -4.466590  -2.393971   0.935405
 H     -4.763445  -1.628174   1.663640
 C     -1.863216   2.490860  -0.399889
 C     -1.732912   3.603942   0.465146
 H     -1.589444   3.446767   1.545995
 C     -1.805966   4.912882  -0.038382
 H     -1.715215   5.767685   0.650507
 C     -1.995398   5.131884  -1.416390
 H     -2.050893   6.158808  -1.810881
 C     -2.120010   4.033483  -2.284432
 H     -2.276803   4.195207  -3.362774
 C     -2.057649   2.720574  -1.781077
 H     -2.185214   1.871034  -2.469114
 C     -2.821284   0.807567   1.744398
 C     -2.544922   0.054174   2.906001
 H     -1.615606  -0.525965   2.983404
 C     -3.455374   0.034059   3.978301
 H     -3.221615  -0.557421   4.877616
 C     -4.653610   0.765727   3.905353
 H     -5.364620   0.750598   4.746531
 C     -4.935870   1.523660   2.754061
 H     -5.868927   2.105632   2.688705
 C     -4.026060   1.547816   1.683050
 H     -4.255792   2.159780   0.797168
 C     -0.394348  -1.766836  -1.571938
 H     -0.366313  -2.415167  -0.674073
 C      0.689694   0.243831  -2.405722
 H      1.196469  -0.278704  -3.252862
 H     -0.289320   0.584510  -2.835852
 C      1.505140   1.457501  -2.074384
 C      2.576702   2.804947  -0.479550
 C      3.107048   3.592825  -1.518825
 C      2.812305   3.323604  -2.862680
 C      2.013601   2.219847  -3.144215
 H      1.776661   1.924195  -4.177269
 C      0.086254  -2.635403  -2.751704
 H     -0.500212  -3.575355  -2.794755
 H     -0.023579  -2.129116  -3.732528
 H      1.152142  -2.900973  -2.605002
 C      0.650000   0.900230   2.124634
 C      0.129999  -1.229522   1.326254
 H      2.132488  -1.408270  -1.162006
 H      3.107467  -1.390633   0.751731
 C      3.542307  -2.123205   0.018352
 C      3.294688  -3.563769   0.554419
 C      4.468909  -3.842427   1.526349
```

```
 O      3.027577  -1.860941  -1.270863
 H      3.224233   3.953301  -3.666366
 H      3.767335   4.432727  -1.275112
 C      5.058894  -2.009864   0.087642
 C      5.589347  -2.987110   0.958775
 C      5.898431  -1.101081  -0.574221
 C      6.974767  -3.053132   1.184478
 C      7.288607  -1.170989  -0.351328
 H      5.472343  -0.357054  -1.266512
 C      7.821779  -2.139053   0.524107
 H      7.399703  -3.815574   1.858451
 H      7.964487  -0.470476  -0.868338
 H      8.910830  -2.187935   0.686337
 H      3.356859  -4.248362  -0.319796
 H      2.294440  -3.682300   1.016646
 H      4.736665  -4.917051   1.605770
 H      4.215646  -3.504427   2.557936
 C      2.968612   3.138201   0.977096
 C      1.724988   3.730363   1.688887
 C      3.541845   1.868385   1.657039
 C      4.083448   4.210389   1.060716
 H      1.522106   4.750191   1.300719
 H      0.821207   3.122223   1.509818
 H      1.889203   3.798102   2.783811
 H      4.525718   1.611712   1.211615
 H      3.688189   2.043041   2.742632
 H      2.876521   0.993228   1.535043
 H      4.340055   4.367447   2.128700
 H      5.011203   3.896434   0.539040
 H      3.765482   5.193136   0.656319
---------------------------------------
Mn15/viii
Frequencies, energies and thermodynamic properties:
 Lowest Vibrational Mode (1/cm) =                         17.5032
 2nd Lowest Vibrational Mode (1/cm) =                     23.7096
 E(RB-P86) (a.u.) =                                     -4409.66506989
 Thermal correction to Enthalpy (a.u.) =                  0.672541
 Thermal correction to Gibbs Free Energy (a.u.) =         0.557187
 Total Entropy (cal/Kmol) =                               242.782
 E(RPBE1PBE) (a.u.) =                                   -4408.98366702
Optimised cartesian coordinates (Angstrom):
 Fe3.509570  -0.837469   0.332831
 Mn-1.014465  -0.332189  -1.463520
 P      0.206772   0.572582   0.251214
 O     -1.718741   2.300904  -2.625401
 O      1.155854  -0.120299  -3.405926
 N     -0.437544  -2.192311  -0.838449
 N     -2.927291  -1.042622  -0.280111
 C      1.531818  -0.562477   0.830376
 C      1.748610  -1.918322   0.335196
 C      2.730771  -2.529580   1.199205
 H      3.132531  -3.545432   1.092402
 C      3.132431  -1.578374   2.201578
 H      3.883757  -1.743060   2.985930
 C      2.404836  -0.360306   1.973705
 H      2.485779   0.556208   2.571826
 C      3.989056  -0.006358  -1.493123
 H      3.269720   0.278724  -2.270999
 C      4.601971  -1.300114  -1.352391
 H      4.438561  -2.166645  -2.007180
 C      5.448312  -1.268112  -0.186460
 H      6.039717  -2.106513   0.205555
 C      5.360520   0.049693   0.390800
 H      5.874991   0.390739   1.299344
 C      4.456534   0.829146  -0.414608
 H      4.160221   1.870249  -0.231874
 C     -0.729539   0.946670   1.823279
 C     -1.489382   2.137462   1.904828
 H     -1.477750   2.857028   1.071329
 C     -2.244817   2.430232   3.053073
 H     -2.822431   3.367150   3.100015
 C     -2.259686   1.534850   4.138462
 H     -2.851455   1.764673   5.038679
 C     -1.507693   0.349139   4.068721
 H     -1.503449  -0.354923   4.916094
 C     -0.747134   0.057738   2.921161
 H     -0.148430  -0.865593   2.892104
 C      1.100572   2.194792   0.000643
 C      1.389696   2.677866  -1.293850
 H      1.049871   2.128826  -2.181346
 C      2.118763   3.868620  -1.470718
 H      2.330005   4.228140  -2.490305
 C      2.571650   4.596032  -0.356943
 H      3.140820   5.528765  -0.496460
 C      2.286310   4.126813   0.938533
```

```
 H      2.630705   4.689991   1.820540
 C      1.554663   2.940223   1.115586
 H      1.327542   2.600766   2.137582
 C      0.982360  -2.558784  -0.828576
 H      1.415001  -2.158902  -1.768659
 C     -1.115373  -2.594059   0.364262
 H     -1.091581  -3.702823   0.513124
 H     -0.655461  -2.191666   1.317233
 C     -2.556979  -2.170163   0.395326
 C     -4.257866  -0.671385  -0.228392
 C     -5.185296  -1.431702   0.512658
 C     -4.792533  -2.577100   1.216010
 C     -3.454367  -2.952238   1.150466
 C      1.207995  -4.087562  -0.877367
 H      2.277232  -4.325565  -1.055227
 H      0.907107  -4.595393   0.062538
 H      0.616337  -4.520716  -1.709365
 C     -1.523264   1.254206  -2.113871
 C      0.327402  -0.195409  -2.563009
 H     -1.614654  -1.624156  -2.450949
 H     -1.971450  -0.977480  -2.817127
 H     -6.236481  -1.124722   0.539564
 H     -3.080649  -3.848686   1.667278
 H     -5.525762  -3.163596   1.791299
 C     -4.774708   0.584875  -0.973099
 C     -4.042445   1.827078  -0.413306
 C     -4.585532   0.401263  -2.500020
 C     -6.288896   0.825555  -0.752407
 H     -4.399148   2.046963   0.614078
 H     -2.953270   1.660798  -0.357195
 H     -4.231322   2.717078  -1.047988
 H     -5.211719  -0.439042  -2.866628
 H     -4.894058   1.321698  -3.037763
 H     -3.536811   0.186318  -2.765958
 H     -6.578713   1.750065  -1.293058
 H     -6.915530   0.001917  -1.152492
 H     -6.544178   0.977451   0.316424
----------------------------------------
Mn15/ix
Frequencies, energies and thermodynamic properties:
 Lowest Vibrational Mode (1/cm) =                        17.5604
 2nd Lowest Vibrational Mode (1/cm) =                    26.7591
 E(RB-P86) (a.u.) =                                     -4563.45732747
 Thermal correction to Enthalpy (a.u.) =                 0.741714
 Thermal correction to Gibbs Free Energy (a.u.) =        0.617730
 Total Entropy (cal/Kmol) =                              260.946
 E(RPBE1PBE) (a.u.) =                                   -4562.78531741
Optimised cartesian coordinates (Angstrom):
 Fe-3.600840  -0.665954  -0.769227
 Mn0.995815  -0.778928   0.893945
 P     -0.425137   0.831281   0.073989
 O      1.702475   0.691862   3.344222
 O     -1.038771  -1.923998   2.635329
 N      0.500990  -1.997875  -0.768033
 N      2.864112  -0.390633  -0.710030
 C     -1.706667   0.090374  -1.040745
 C     -1.773734  -1.310907  -1.453893
 C     -2.772141  -1.408474  -2.493318
 H     -3.078005  -2.328922  -3.006363
 C     -3.331509  -0.104388  -2.720555
 H     -4.132640   0.138526  -3.431454
 C     -2.687189   0.816996  -1.827276
 H     -2.894938   1.892149  -1.761964
 C     -3.986595  -1.151269   1.197514
 H     -3.226263  -1.314010   1.971964
 C     -4.516819  -2.157944   0.316331
 H     -4.239948  -3.220805   0.308960
 C     -5.457324  -1.523389  -0.571492
 H     -6.017872  -2.015424  -1.377660
 C     -5.510016  -0.123106  -0.236048
 H     -6.118389   0.638325  -0.742354
 C     -4.599733   0.107750   0.855226
 H     -4.391589   1.075212   1.330317
 C      0.273378   2.171282  -1.031855
 C      0.859856   3.318183  -0.446565
 H      0.842373   3.447284   0.646961
 C      1.452084   4.311540  -1.244735
 H      1.892239   5.201782  -0.767620
 C      1.482082   4.172352  -2.644739
 H      1.947147   4.950663  -3.270300
 C      0.906935   3.035331  -3.238551
 H      0.915092   2.918039  -4.334027
 C      0.304695   2.046038  -2.439361
 H     -0.168227   1.180828  -2.928966
 C     -1.440766   1.875456   1.248296
```

```
 C     -1.594139   1.502591   2.601075
 H     -1.085713   0.611998   2.991594
 C     -2.400618   2.260953   3.470491
 H     -2.503435   1.951117   4.522625
 C     -3.066877   3.406375   3.002937
 H     -3.696663   4.000320   3.684175
 C     -2.916212   3.793229   1.658333
 H     -3.426921   4.693106   1.279878
 C     -2.108545   3.037760   0.791536
 H     -1.989349   3.370867  -0.250518
 C     -0.913570  -2.452442  -0.930643
 H     -1.246144  -2.703879   0.095816
 C      1.132759  -1.580816  -2.024235
 H      1.150182  -2.403327  -2.771761
 H      0.514776  -0.772784  -2.474305
 C      2.540273  -1.065806  -1.843398
 C      4.140316   0.103855  -0.599456
 C      5.072345  -0.058362  -1.647476
 H      6.082980   0.353942  -1.544825
 C      4.728693  -0.752109  -2.813100
 C      3.440405  -1.277529  -2.906860
 H      3.115320  -1.846394  -3.790748
 C     -1.037819  -3.733193  -1.773622
 H     -2.072156  -4.127233  -1.716333
 H     -0.800211  -3.569471  -2.844158
 H     -0.360325  -4.517787  -1.379218
 C      1.474843   0.132212   2.326934
 C     -0.256761  -1.421192   1.895360
 H      1.079317  -2.738516  -0.299453
 H      5.459986  -0.886943  -3.625238
 C      4.582352   0.817122   0.697038
 C      3.615839   1.986505   0.985165
 C      4.600666  -0.236635   1.835648
 C      6.003858   1.418079   0.593265
 H      3.755373   2.794347   0.237578
 H      2.563324   1.657371   0.929764
 H      3.800159   2.407122   1.994971
 H      5.449616  -0.937926   1.688489
 H      4.729920   0.259280   2.820010
 H      3.669983  -0.834714   1.839703
 H      6.240297   1.933407   1.547229
 H      6.785002   0.645289   0.439104
 H      6.087146   2.170643  -0.218602
 C      2.300127  -3.088021   2.333693
 C      3.185663  -4.325491   2.136990
 H      2.769118  -2.436439   3.122017
 H      1.319640  -3.419524   2.769725
 H      3.323478  -4.876433   3.092396
 H      2.731145  -5.022017   1.399870
 H      4.188688  -4.038040   1.755781
 O      2.144230  -2.397880   1.126369
---------------------------------------
Mn15/x
Frequencies, energies and thermodynamic properties:
 Lowest Vibrational Mode (1/cm) =                          16.8786
 2nd Lowest Vibrational Mode (1/cm) =                      20.0354
 E(RB-P86) (a.u.) =                                       -4718.39228485
 Thermal correction to Enthalpy (a.u.) =                   0.826568
 Thermal correction to Gibbs Free Energy (a.u.) =          0.688427
 Total Entropy (cal/Kmol) =                                290.742
 E(RPBE1PBE) (a.u.) =                                     -4717.73303814
Optimised cartesian coordinates (Angstrom):
 Fe-3.704696  -1.125566  -0.786238
 Mn0.840527  -0.438258   0.909094
 P     -0.830514   0.893101   0.056134
 O      1.211559   1.102858   3.386003
 O     -1.022689  -1.866095   2.641004
 N      0.560319  -1.757404  -0.723944
 N      2.503052   0.309059  -0.633493
 C     -1.958765  -0.072550  -1.050560
 C     -1.789307  -1.471423  -1.442270
 C     -2.745222  -1.746241  -2.490185
 H     -2.889146  -2.711345  -2.991941
 C     -3.507921  -0.555069  -2.743681
 H     -4.329360  -0.457074  -3.465977
 C     -3.034936   0.472118  -1.859107
 H     -3.417466   1.499133  -1.813277
 C     -4.042452  -1.602049   1.191628
 H     -3.284577  -1.593530   1.984965
 C     -4.356803  -2.720801   0.342922
 H     -3.888970  -3.713692   0.383196
 C     -5.375998  -2.303579  -0.585677
 H     -5.816488  -2.919631  -1.381056
 C     -5.693156  -0.925695  -0.308187
 H     -6.418681  -0.308878  -0.855347
```

```
 C     -4.867621  -0.490960   0.788394
 H     -4.852494   0.514995   1.227679
 C     -0.353300   2.305316  -1.074347
 C     -0.019518   3.565575  -0.524949
 H     -0.104497   3.733826   0.559683
 C      0.401497   4.622109  -1.350577
 H      0.644915   5.598493  -0.901978
 C      0.508451   4.436628  -2.741086
 H      0.838363   5.264956  -3.387887
 C      0.182960   3.187930  -3.298944
 H      0.253013   3.031784  -4.387231
 C     -0.246792   2.133143  -2.473173
 H     -0.526743   1.174183  -2.935274
 C     -2.017751   1.768274   1.205881
 C     -2.118924   1.398904   2.564560
 H     -1.469071   0.617510   2.977585
 C     -3.053379   2.022042   3.412969
 H     -3.112220   1.717771   4.470039
 C     -3.902033   3.026676   2.918397
 H     -4.631900   3.515095   3.583350
 C     -3.806429   3.409237   1.567568
 H     -4.460744   4.200258   1.167736
 C     -2.871994   2.788791   0.721364
 H     -2.802349   3.118183  -0.326186
 C     -0.757633  -2.447146  -0.896107
 H     -1.055984  -2.743641   0.128717
 C      1.129395  -1.274190  -1.984632
 H      1.456020  -2.115149  -2.632982
 H      0.329942  -0.742460  -2.546127
 C      2.297433  -0.333578  -1.814697
 C      3.589296   1.157671  -0.550852
 C      4.400261   1.400474  -1.678498
 C      4.156087   0.760040  -2.899087
 C      3.098087  -0.141472  -2.959007
 C     -0.646111  -3.733602  -1.731115
 H     -1.594453  -4.304771  -1.679878
 H     -0.431078  -3.532222  -2.799986
 H      0.157306  -4.383495  -1.328219
 C      1.119199   0.537717   2.350950
 C     -0.310312  -1.268612   1.903027
 H      3.199145  -2.535706   0.077004
 H      1.239019  -2.409033  -0.278213
 O      3.562097  -3.109400  -0.679215
 C      4.916207  -2.776546  -0.926277
 H      5.195776  -3.230698  -1.905252
 H      5.053724  -1.672576  -1.050784
 H      2.870703  -0.701885  -3.878148
 H      5.244319   2.094563  -1.598366
 C      5.874909  -3.286218   0.155754
 H      5.781100  -4.386332   0.276582
 H      6.931407  -3.054514  -0.100202
 H      5.652356  -2.815393   1.137083
 O      2.289094  -1.858317   1.239713
 C      2.292193  -2.781615   2.308803
 C      2.809490  -2.228947   3.642848
 H      2.943695  -3.641521   2.008477
 H      1.277348  -3.223346   2.478980
 H      2.798017  -3.023733   4.420567
 H      3.852099  -1.859516   3.547772
 H      2.183306  -1.388151   4.005258
 H      4.794911   0.952331  -3.774989
 C      3.954197   1.850009   0.782987
 C      2.814671   2.821759   1.168861
 C      4.216896   0.761221   1.854187
 C      5.244352   2.699853   0.680218
 H      2.801318   3.687640   0.475657
 H      1.828247   2.330546   1.108137
 H      2.955126   3.200229   2.202148
 H      5.185612   0.258238   1.646394
 H      4.280536   1.220788   2.862427
 H      3.435330  -0.021210   1.856066
 H      5.449729   3.144767   1.675864
 H      6.131529   2.094084   0.402031
 H      5.149496   3.539656  -0.039077
---------------------------------------
Mn15/TS-i
Frequencies, energies and thermodynamic properties:
 Lowest Vibrational Mode (1/cm) =-707.0064
 2nd Lowest Vibrational Mode (1/cm) =                      19.3038
 E(RB-P86) (a.u.) =                                      -4564.60655294
 Thermal correction to Enthalpy (a.u.) =                   0.753351
 Thermal correction to Gibbs Free Energy (a.u.) =          0.626650
 Total Entropy (cal/Kmol) =                                266.663
 E(RPBE1PBE) (a.u.) =                                    -4563.92781271
Optimised cartesian coordinates (Angstrom):
```

```
Fe-3.554955   0.996818   0.640497
Mn1.011535   0.369039  -1.057814
P     -0.564463  -0.898603   0.051683
O      1.363972  -1.680264  -3.155202
O     -0.880003   1.472697  -2.991736
N      0.669043   1.923902   0.294019
N      2.864833   0.152180   0.376795
C     -1.733765   0.129586   1.029026
C     -1.653819   1.579684   1.175491
C     -2.609363   1.957258   2.190354
H     -2.813792   2.980824   2.528929
C     -3.284264   0.776720   2.658327
H     -4.083833   0.745638   3.410709
C     -2.756870  -0.350813   1.941413
H     -3.065245  -1.395489   2.073880
C     -3.943643   1.134607  -1.379338
H     -3.196295   1.058842  -2.179237
C     -4.334140   2.345764  -0.708326
H     -3.942033   3.351458  -0.911023
C     -5.310824   2.004734   0.294475
H     -5.789872   2.703739   0.993124
C     -5.526239   0.580955   0.239724
H     -6.199704   0.005659   0.889137
C     -4.679628   0.041953  -0.792665
H     -4.594639  -1.015881  -1.073195
C      0.083088  -2.096325   1.329358
C      0.597935  -3.344018   0.904702
H      0.559471  -3.622556  -0.159978
C      1.139814  -4.250415   1.831688
H      1.526449  -5.220632   1.481320
C      1.184964  -3.923193   3.199421
H      1.609685  -4.633925   3.925882
C      0.676274  -2.686507   3.632921
H      0.695969  -2.423291   4.702473
C      0.127069  -1.781187   2.706239
H     -0.291586  -0.830379   3.070177
C     -1.690345  -2.024597  -0.926266
C     -1.898400  -1.829139  -2.308685
H     -1.361454  -1.037210  -2.846228
C     -2.798876  -2.641929  -3.022184
H     -2.943076  -2.472831  -4.101031
C     -3.506810  -3.663026  -2.365855
H     -4.210151  -4.299716  -2.925569
C     -3.305205  -3.870004  -0.988790
H     -3.849822  -4.670196  -0.462771
C     -2.403122  -3.061412  -0.276756
H     -2.248136  -3.252259   0.795945
C     -0.695625   2.501369   0.429593
H     -1.070524   2.612034  -0.607914
C      1.256360   1.580063   1.582709
H      1.322037   2.459812   2.263253
H      0.633927   0.831685   2.135552
C      2.631807   0.992050   1.429487
C      4.117497  -0.419182   0.278706
C      5.118182  -0.113642   1.226583
C      4.872745   0.756610   2.293356
C      3.601115   1.315717   2.397050
C     -0.665892   3.921697   1.031187
H     -1.664384   4.398221   0.955826
H     -0.371867   3.928324   2.101033
H      0.053297   4.543233   0.461156
C      1.289563  -0.902712  -2.270504
C     -0.168622   1.002702  -2.171017
H      2.248657   1.121181  -1.941728
H      2.019917   1.878634  -1.608509
H      1.405376   2.843399  -0.343002
O      2.004976   3.505866  -1.122487
C      3.268226   3.913252  -0.648579
H      3.227864   4.183619   0.439738
H      4.024454   3.085055  -0.721424
H      3.340565   2.008165   3.210805
H      6.110775  -0.566008   1.127011
H      5.663449   0.990695   3.022894
C      3.786981   5.117309  -1.438827
H      3.086352   5.974823  -1.350378
H      4.784162   5.443521  -1.072801
H      3.880049   4.867534  -2.517297
C      4.469908  -1.419584  -0.851739
C      3.449631  -2.580430  -0.824237
C      4.520039  -0.684737  -2.216248
C      5.868471  -2.058609  -0.657198
H      3.628552  -3.224236   0.061408
H      2.414465  -2.206060  -0.755498
H      3.539118  -3.205953  -1.735797
H      5.315614   0.089481  -2.207090
```

```
 H      4.754053  -1.407204  -3.025706
 H      3.567657  -0.189281  -2.470572
 H      6.024956  -2.807274  -1.460793
 H      6.693361  -1.320898  -0.735339
 H      5.959554  -2.590860   0.311924
---------------------------------------
Mn15/TS-ii_si
Frequencies, energies and thermodynamic properties:
 Lowest Vibrational Mode (1/cm) =-397.8003
 2nd Lowest Vibrational Mode (1/cm) =                     17.8175
 E(RB-P86) (a.u.) =                                     -4832.40200481
 Thermal correction to Enthalpy (a.u.) =                  0.826183
 Thermal correction to Gibbs Free Energy (a.u.) =         0.693368
 Total Entropy (cal/Kmol) =                               279.531
 E(RPBE1PBE) (a.u.) =                                   -4831.71745124
Optimised cartesian coordinates (Angstrom):
 Fe-3.659614  -1.174935  -0.943134
 Mn0.954936  -0.077445   0.246840
 P     -1.095305   0.965850   0.415404
 O      1.709338   0.176739   3.080269
 O     -0.033085  -2.625752   1.268985
 N      0.551970  -0.571638  -1.738804
 N      2.162324   1.584280  -0.885238
 C     -2.269537   0.336186  -0.867449
 C     -1.940330  -0.619859  -1.922130
 C     -3.058796  -0.656175  -2.836250
 H     -3.138657  -1.287651  -3.729875
 C     -4.073117   0.244688  -2.362092
 H     -5.052147   0.416487  -2.829151
 C     -3.597113   0.851464  -1.151030
 H     -4.142095   1.590413  -0.550690
 C     -3.364646  -2.582214   0.533485
 H     -2.432047  -2.725748   1.094161
 C     -3.704214  -3.221125  -0.710602
 H     -3.082790  -3.943983  -1.256223
 C     -4.991938  -2.728456  -1.128465
 H     -5.521194  -3.004815  -2.050282
 C     -5.449740  -1.786245  -0.139190
 H     -6.389657  -1.219256  -0.175706
 C     -4.443835  -1.693895   0.886762
 H     -4.480175  -1.045287   1.771553
 C     -1.215768   2.810626   0.116292
 C     -1.001007   3.713781   1.184044
 H     -0.827137   3.331233   2.201681
 C     -1.023634   5.102567   0.969679
 H     -0.865193   5.786092   1.819136
 C     -1.248641   5.618105  -0.319885
 H     -1.265983   6.706610  -0.487777
 C     -1.459074   4.731927  -1.390844
 H     -1.646661   5.121628  -2.404141
 C     -1.446871   3.341945  -1.173631
 H     -1.650209   2.669814  -2.021607
 C     -2.080002   0.822987   2.000065
 C     -1.740988  -0.136208   2.978256
 H     -0.867336  -0.784747   2.837840
 C     -2.515694  -0.280522   4.144743
 H     -2.230302  -1.035356   4.894643
 C     -3.641919   0.532978   4.354250
 H     -4.246707   0.420823   5.268120
 C     -3.987127   1.497582   3.389390
 H     -4.864186   2.146347   3.543236
 C     -3.212433   1.642867   2.226205
 H     -3.491271   2.416059   1.494615
 C     -0.650772  -1.412806  -2.061240
 H     -0.643208  -2.208559  -1.290546
 C      0.686919   0.605719  -2.603760
 H      0.769895   0.329536  -3.676990
 H     -0.228078   1.231357  -2.511697
 C      1.874926   1.442378  -2.214618
 C      3.164082   2.474351  -0.550055
 C      3.898073   3.142645  -1.554635
 H      4.699172   3.831940  -1.265428
 C      3.628388   2.944494  -2.911922
 C      2.583296   2.084402  -3.245583
 H      2.301595   1.898102  -4.292302
 C     -0.519493  -2.106638  -3.428450
 H     -1.316286  -2.867428  -3.548673
 H     -0.599573  -1.398862  -4.278514
 H      0.457624  -2.626203  -3.490740
 C      1.440021   0.161091   1.926735
 C      0.317752  -1.578438   0.834474
 H      1.399461  -1.186753  -1.949283
 H      2.337018  -1.045994  -0.026800
 C      3.251038  -2.022966  -0.932609
 C      3.129909  -3.261925  -0.074750
```

```
 C      4.614601  -1.401658  -0.529364
 C      4.119832  -3.252230   0.931782
 C      2.268559  -4.358263  -0.240324
 C      4.932737  -1.971268   0.870295
 H      5.345089  -1.785927  -1.277378
 C      4.243890  -4.346689   1.804066
 C      2.396210  -5.454942   0.630950
 H      1.517462  -4.355669  -1.046346
 C      3.374729  -5.445605   1.648360
 H      5.016068  -4.356110   2.591068
 H      1.735284  -6.329442   0.517998
 H      3.467156  -6.312981   2.321981
 O      2.783707  -1.948513  -2.114109
 H      4.624786  -0.298122  -0.597278
 H      4.216986   3.459881  -3.686437
 H      6.015454  -2.143361   1.043049
 H      4.591375  -1.274778   1.668591
 C      3.513103   2.780390   0.927747
 C      2.224177   3.162000   1.687613
 C      4.226838   1.551476   1.540047
 C      4.480171   3.981910   1.072351
 H      1.859362   4.152594   1.348121
 H      1.417902   2.429524   1.509466
 H      2.417210   3.215752   2.778707
 H      5.236235   1.432273   1.093817
 H      4.345449   1.676435   2.636156
 H      3.655774   0.624991   1.354574
 H      4.632159   4.179556   2.153544
 H      5.481285   3.786092   0.636460
 H      4.072115   4.909312   0.619599
---------------------------------------
Mn15/TS-ii_re
Frequencies, energies and thermodynamic properties:
 Lowest Vibrational Mode (1/cm) =-377.8779
 2nd Lowest Vibrational Mode (1/cm) =                      19.2328
 E(RB-P86) (a.u.) =                                      -4832.40465212
 Thermal correction to Enthalpy (a.u.) =                   0.826137
 Thermal correction to Gibbs Free Energy (a.u.) =          0.693422
 Total Entropy (cal/Kmol) =                                279.322
 E(RPBE1PBE) (a.u.) =                                    -4831.72206633
Optimised cartesian coordinates (Angstrom):
 Fe-3.966217  -1.244127  -0.763901
 Mn0.686346  -0.326955   0.447574
 P     -1.254135   0.901590   0.255220
 O      1.314588   0.446799   3.215090
 O     -0.571658  -2.517542   1.909323
 N      0.316177  -1.219106  -1.402496
 N      2.078763   0.922153  -0.923577
 C     -2.433782   0.113891  -0.929877
 C     -2.159320  -1.079042  -1.727559
 C     -3.235407  -1.217026  -2.681816
 H     -3.341018  -2.022546  -3.419192
 C     -4.171495  -0.145062  -2.481355
 H     -5.107065   0.005836  -3.036431
 C     -3.688265   0.670982  -1.403229
 H     -4.180145   1.570858  -1.013576
 C     -3.876387  -2.320390   0.991561
 H     -2.985558  -2.430118   1.623462
 C     -4.238449  -3.177306  -0.106487
 H     -3.679541  -4.058287  -0.449790
 C     -5.450155  -2.663736  -0.692597
 H     -5.972894  -3.080044  -1.564163
 C     -5.839047  -1.489958   0.046927
 H     -6.710954  -0.855809  -0.162518
 C     -4.865884  -1.275941   1.086094
 H     -4.864197  -0.451307   1.810608
 C     -1.153971   2.632733  -0.449140
 C     -0.866052   3.724662   0.403470
 H     -0.771371   3.565445   1.488746
 C     -0.717351   5.023095  -0.113377
 H     -0.504817   5.859517   0.571531
 C     -0.841450   5.255502  -1.495458
 H     -0.724518   6.273240  -1.900263
 C     -1.124385   4.179002  -2.354217
 H     -1.234756   4.348061  -3.437286
 C     -1.282738   2.880902  -1.835284
 H     -1.538202   2.060330  -2.523436
 C     -2.312996   1.210484   1.765132
 C     -2.133075   0.448148   2.939291
 H     -1.339020  -0.307440   2.991674
 C     -2.967496   0.639405   4.056653
 H     -2.807140   0.033896   4.962852
 C     -3.995483   1.596698   4.019349
 H     -4.647082   1.747181   4.894711
 C     -4.181898   2.366597   2.856187
```

```
 H     -4.980748   3.124189   2.815097
 C     -3.347104   2.177528   1.741731
 H     -3.500414   2.802780   0.849224
 C     -0.954380  -1.997451  -1.603709
 H     -1.059920  -2.592781  -0.675803
 C      0.602621  -0.284670  -2.495717
 H      0.734335  -0.804896  -3.469246
 H     -0.267124   0.397294  -2.624122
 C      1.821154   0.552261  -2.214272
 C      3.141029   1.779999  -0.713653
 C      3.930398   2.224665  -1.796762
 H      4.769129   2.903179  -1.606292
 C      3.671956   1.814864  -3.107953
 C      2.592012   0.959028  -3.317909
 H      2.326786   0.601207  -4.323613
 C     -0.839968  -2.997447  -2.767309
 H     -1.715145  -3.677256  -2.773307
 H     -0.793184  -2.501265  -3.758088
 H      0.069130  -3.617220  -2.635067
 C      1.106226   0.220114   2.071060
 C     -0.113492  -1.609170   1.292864
 H      1.094234  -1.950252  -1.407676
 H      2.017859  -1.392490   0.450332
 C      2.826962  -2.688868  -0.122705
 C      4.218809  -2.132295   0.041975
 C      2.614269  -3.579007   1.131329
 C      4.690374  -2.387347   1.348856
 C      5.042673  -1.550759  -0.935123
 C      3.602626  -3.036744   2.186333
 C      6.011483  -2.055589   1.691712
 C      6.364703  -1.217588  -0.588711
 H      4.655097  -1.383280  -1.953024
 C      6.843749  -1.469816   0.715673
 H      6.400117  -2.258594   2.703345
 H      7.036188  -0.769494  -1.338806
 H      7.884927  -1.213869   0.970743
 O      2.319729  -2.947511  -1.259748
 H      3.994039  -3.814650   2.874030
 H      3.109515  -2.265360   2.821607
 H      1.561476  -3.630452   1.462392
 H      2.917640  -4.603533   0.815191
 H      4.301762   2.159080  -3.942731
 C      3.501044   2.284856   0.704548
 C      2.263616   2.970737   1.326603
 C      4.025755   1.092202   1.541257
 C      4.627784   3.347661   0.689545
 H      2.058279   3.930679   0.810083
 H      1.362384   2.340549   1.232434
 H      2.434529   3.181598   2.402220
 H      5.019760   0.773027   1.167661
 H      4.127600   1.383413   2.607134
 H      3.351161   0.220384   1.476473
 H      4.801832   3.685447   1.732062
 H      5.591431   2.946819   0.313500
 H      4.359475   4.244116   0.092707
---------------------------------------
Mn15/TS-iii
Frequencies, energies and thermodynamic properties:
 Lowest Vibrational Mode (1/cm) =-526.9008
 2nd Lowest Vibrational Mode (1/cm) =                       19.6897
 E(RB-P86) (a.u.) =                                       -4409.66354446
 Thermal correction to Enthalpy (a.u.) =                    0.671367
 Thermal correction to Gibbs Free Energy (a.u.) =          0.557220
 Total Entropy (cal/Kmol) =                                 240.243
 E(RPBE1PBE) (a.u.) =                                     -4408.97968162
Optimised cartesian coordinates (Angstrom):
 Fe3.492522  -0.833854   0.322648
 Mn-1.001467  -0.348605  -1.456973
 P      0.193056   0.594735   0.265771
 O     -1.740802   2.175314  -2.806102
 O      1.175406  -0.124502  -3.389247
 N     -0.453177  -2.240541  -0.805584
 N     -2.917235  -1.039248  -0.263330
 C      1.517164  -0.555309   0.829011
 C      1.734011  -1.915932   0.336335
 C      2.720461  -2.522175   1.200172
 H      3.122692  -3.538001   1.096390
 C      3.125963  -1.567701   2.196673
 H      3.881962  -1.729073   2.977089
 C      2.395896  -0.352132   1.967757
 H      2.479660   0.567083   2.561323
 C      3.969370   0.014345  -1.496596
 H      3.248253   0.314144  -2.266920
 C      4.571127  -1.286653  -1.374151
 H      4.396558  -2.143826  -2.038319
```

```
 C      5.424390  -1.275646  -0.213084
 H      6.010471  -2.123664   0.166020
 C      5.351432   0.036070   0.379945
 H      5.873527   0.361697   1.289782
 C      4.450081   0.832872  -0.411364
 H      4.163520   1.874205  -0.214659
 C     -0.727964   0.967605   1.847856
 C     -1.502757   2.149337   1.924322
 H     -1.503526   2.862541   1.085149
 C     -2.258244   2.440753   3.072765
 H     -2.847448   3.370714   3.115031
 C     -2.258961   1.552667   4.164403
 H     -2.851094   1.780982   5.064760
 C     -1.493315   0.375470   4.099509
 H     -1.478670  -0.323461   4.951051
 C     -0.733150   0.085541   2.951179
 H     -0.125320  -0.832093   2.926289
 C      1.091204   2.213401   0.014488
 C      1.360524   2.698257  -1.283637
 H      1.008410   2.148009  -2.165837
 C      2.084334   3.890860  -1.469712
 H      2.280750   4.251996  -2.491718
 C      2.550156   4.618594  -0.361393
 H      3.115000   5.552911  -0.507868
 C      2.282719   4.148417   0.937682
 H      2.636778   4.712767   1.815134
 C      1.556850   2.959619   1.123891
 H      1.342496   2.619210   2.148447
 C      0.972144  -2.591182  -0.811442
 H      1.383589  -2.202267  -1.765324
 C     -1.128536  -2.603762   0.412941
 H     -1.116946  -3.707338   0.591171
 H     -0.644086  -2.171714   1.335597
 C     -2.563899  -2.153951   0.440054
 C     -4.240333  -0.647347  -0.220593
 C     -5.175514  -1.359574   0.557927
 C     -4.798103  -2.488506   1.294703
 C     -3.469474  -2.897574   1.222726
 C      1.209632  -4.117541  -0.831987
 H      2.280211  -4.350378  -1.006900
 H      0.912964  -4.608750   0.117870
 H      0.621675  -4.571259  -1.655400
 C     -1.525377   1.179464  -2.208755
 C      0.347445  -0.211027  -2.545915
 H     -1.338350  -1.859770  -2.109697
 H     -1.764567  -1.281626  -2.668494
 H     -6.219658  -1.028707   0.584165
 H     -3.110468  -3.788252   1.759458
 H     -5.535744  -3.039500   1.898668
 C     -4.734812   0.574243  -1.030249
 C     -3.994944   1.835085  -0.522119
 C     -4.523889   0.294951  -2.540794
 C     -6.248300   0.842193  -0.842342
 H     -4.363731   2.107788   0.488307
 H     -2.907841   1.660193  -0.443226
 H     -4.165314   2.694805  -1.201913
 H     -5.204957  -0.515914  -2.875045
 H     -4.749125   1.203078  -3.137246
 H     -3.488537  -0.019540  -2.761663
 H     -6.523764   1.732571  -1.444441
 H     -6.878017  -0.000218  -1.195812
 H     -6.514036   1.064632   0.211645
---------------------------------------
Mn16/i
Frequencies, energies and thermodynamic properties:
 Lowest Vibrational Mode (1/cm) =                         17.9869
 2nd Lowest Vibrational Mode (1/cm) =                     25.3626
 E(RB-P86) (a.u.) =                                     -4460.21623375
 Thermal correction to Enthalpy (a.u.) =                  0.606592
 Thermal correction to Gibbs Free Energy (a.u.) =         0.493621
 Total Entropy (cal/Kmol) =                               237.768
 E(RPBE1PBE) (a.u.) =                                   -4459.52937039
Optimised cartesian coordinates (Angstrom):
 Fe3.643686  -0.640689  -0.110369
 Mn-0.986432  -0.132083  -1.207581
 P      0.274915   0.446451   0.479308
 O     -2.211979   2.569314  -1.303310
 O      0.767933   0.870060  -3.325030
 N     -0.300022  -1.919030  -1.148482
 N     -2.678591  -1.166834  -0.411243
 C      1.738183  -0.650678   0.657763
 C      1.965935  -1.840029  -0.155065
 C      3.085257  -2.545551   0.421010
 H      3.526795  -3.473851   0.036065
 C      3.555929  -1.810546   1.566304
```

```
 H      4.410219  -2.082852   2.200975
 C      2.736803  -0.637362   1.711505
 H      2.842707   0.129362   2.489978
 C      3.822331   0.605674  -1.743385
 H      2.990532   0.980503  -2.353972
 C      4.538495  -0.622052  -1.965994
 H      4.352944  -1.339615  -2.776616
 C      5.525024  -0.759373  -0.924621
 H      6.219521  -1.601009  -0.799817
 C      5.420456   0.387924  -0.059130
 H      6.022478   0.573667   0.840538
 C      4.366291   1.230096  -0.562408
 H      4.023182   2.173276  -0.117868
 C     -0.647971   0.190954   2.076889
 C     -1.739694   1.050824   2.351586
 H     -1.977313   1.873587   1.657647
 C     -2.520468   0.867914   3.503776
 H     -3.358523   1.552798   3.709733
 C     -2.236414  -0.188962   4.391403
 H     -2.851665  -0.334327   5.293523
 C     -1.164875  -1.055885   4.119412
 H     -0.935343  -1.884729   4.808125
 C     -0.372472  -0.868193   2.969918
 H      0.474268  -1.544726   2.778784
 C      0.945989   2.166214   0.703113
 C      1.143770   3.004746  -0.415643
 H      0.871016   2.655659  -1.421636
 C      1.691503   4.290414  -0.255678
 H      1.837394   4.933057  -1.138444
 C      2.046134   4.756206   1.023121
 H      2.472229   5.764473   1.147094
 C      1.845866   3.930563   2.144460
 H      2.113774   4.289119   3.151176
 C      1.296077   2.646104   1.986969
 H      1.128932   2.017846   2.875908
 C      1.122455  -2.230256  -1.376869
 H      1.438620  -1.580019  -2.216588
 C     -0.936814  -2.833369  -0.226423
 H     -1.037342  -3.871289  -0.634223
 H     -0.342317  -2.964202   0.717540
 C     -2.309933  -2.360348   0.139072
 C     -3.976753  -0.774949  -0.245598
 C     -4.871988  -1.459682   0.592192
 H     -5.906401  -1.099608   0.682095
 C     -4.443758  -2.625989   1.244981
 C     -3.156421  -3.105005   0.978962
 H     -2.793957  -4.048278   1.413888
 C      1.383539  -3.682341  -1.821459
 H      2.431929  -3.796653  -2.164236
 H      1.216675  -4.417570  -1.007610
 H      0.719903  -3.942948  -2.671207
 C     -1.791992   1.463225  -1.286882
 C      0.109151   0.469206  -2.419919
 H     -5.127915  -3.176105   1.908477
 N     -4.458029   0.315822  -1.010108
 C     -4.376072   0.397899  -2.400780
 C     -5.301027   1.320962  -0.545166
 C     -5.156423   1.466855  -2.812457
 H     -3.784911  -0.330719  -2.965395
 C     -5.735411   2.057131  -1.638322
 H     -5.485746   1.441088   0.528386
 H     -5.297847   1.790343  -3.851366
 H     -6.387930   2.938227  -1.595759
---------------------------------------
Mn16/ii
Frequencies, energies and thermodynamic properties:
 Lowest Vibrational Mode (1/cm) =                        22.1527
 2nd Lowest Vibrational Mode (1/cm) =                    22.7766
 E(RB-P86) (a.u.) =                                    -4615.15323546
 Thermal correction to Enthalpy (a.u.) =                 0.691320
 Thermal correction to Gibbs Free Energy (a.u.) =        0.564367
 Total Entropy (cal/Kmol) =                              267.195
 E(RPBE1PBE) (a.u.) =                                  -4614.47505105
Optimised cartesian coordinates (Angstrom):
 Fe-3.666854   1.094582   0.274641
 Mn0.945684   0.261193  -0.703860
 P     -0.704219  -0.915666   0.114812
 O      1.761462  -1.992980  -2.445374
 O     -0.500654   1.313740  -3.017366
 N      0.567419   1.720252   0.542153
 N      2.631635   0.169374   0.600070
 C     -1.978488   0.124527   0.924990
 C     -1.846614   1.563930   1.124220
 C     -2.932792   1.977480   1.979768
 H     -3.135873   3.004830   2.308494
```

```
 C     -3.734205   0.825961   2.302618
 H     -4.644714   0.825574   2.917077
 C     -3.157982  -0.317371   1.648085
 H     -3.538988  -1.345935   1.694076
 C     -3.714442   1.287673  -1.777748
 H     -2.851302   1.191070  -2.448819
 C     -4.154597   2.503588  -1.147516
 H     -3.689822   3.492604  -1.257893
 C     -5.295760   2.192487  -0.324531
 H     -5.850493   2.901320   0.304798
 C     -5.562799   0.781976  -0.449218
 H     -6.357557   0.228155   0.068385
 C     -4.583925   0.221556  -1.344432
 H     -4.500541  -0.833847  -1.634182
 C     -0.056098  -1.975909   1.502561
 C      0.861584  -3.002956   1.170539
 H      1.124355  -3.185058   0.115944
 C      1.436352  -3.798097   2.174556
 H      2.140206  -4.599732   1.899616
 C      1.117353  -3.570385   3.527757
 H      1.571849  -4.192163   4.315311
 C      0.216404  -2.547216   3.867425
 H     -0.040354  -2.363760   4.922988
 C     -0.369998  -1.753920   2.862003
 H     -1.087871  -0.968049   3.141652
 C     -1.662619  -2.149210  -0.891527
 C     -1.787814  -1.989774  -2.288930
 H     -1.293102  -1.149210  -2.795969
 C     -2.547488  -2.900573  -3.044937
 H     -2.633765  -2.763643  -4.134529
 C     -3.188888  -3.982746  -2.415965
 H     -3.780866  -4.696954  -3.010026
 C     -3.063621  -4.153302  -1.025222
 H     -3.556416  -5.001952  -0.524601
 C     -2.302451  -3.245794  -0.267683
 H     -2.199106  -3.401673   0.817634
 C     -0.729049   2.428630   0.530632
 H     -0.965790   2.584180  -0.541337
 C      1.094182   1.515346   1.884113
 H      1.364355   2.468013   2.404616
 H      0.343728   1.029151   2.561567
 C      2.324924   0.657577   1.834171
 C      3.841987  -0.431986   0.426324
 C      4.682889  -0.743908   1.508591
 C      4.293147  -0.357369   2.799950
 C      3.122985   0.396199   2.961033
 C     -0.668259   3.828273   1.172383
 H     -1.602373   4.390119   0.968959
 H     -0.532512   3.791277   2.272939
 H      0.174076   4.398016   0.730319
 C      1.491541  -1.070971  -1.756332
 C      0.017484   0.859291  -2.049962
 H      1.781077   2.757895  -0.375431
 O      2.420719   3.326120  -0.907094
 C      3.408321   3.849117  -0.027919
 H      4.003033   3.031757   0.454880
 H      2.938635   4.428693   0.806506
 H      2.823318   0.786953   3.944470
 H      5.651711  -1.228607   1.324767
 C      4.347489   4.764249  -0.806019
 H      5.117933   5.202281  -0.138147
 H      4.867422   4.206513  -1.613218
 H      3.781219   5.595739  -1.275880
 H      4.934478  -0.591323   3.663189
 N      4.276733  -0.651886  -0.897229
 C      4.171318   0.295850  -1.920461
 C      5.036320  -1.729261  -1.346300
 C      4.859789  -0.193504  -3.019327
 H      3.642502   1.242639  -1.746805
 C      5.399353  -1.476424  -2.660755
 H      5.213033  -2.597685  -0.701787
 H      4.971868   0.322151  -3.981289
 H      5.980125  -2.150745  -3.302532
---------------------------------------
Mn16/iii
Frequencies, energies and thermodynamic properties:
 Lowest Vibrational Mode (1/cm) =                        16.5151
 2nd Lowest Vibrational Mode (1/cm) =                    23.1270
 E(RB-P86) (a.u.) =                                    -4616.32445370
 Thermal correction to Enthalpy (a.u.) =                 0.707654
 Thermal correction to Gibbs Free Energy (a.u.) =        0.580677
 Total Entropy (cal/Kmol) =                              267.246
 E(RPBE1PBE) (a.u.) =                                  -4615.64435898
Optimised cartesian coordinates (Angstrom):
 Fe-3.706474   0.759941   0.336865
```

Mn0.984727	0.469885	-0.979486	
P	-0.498691	-0.833671	0.166125
O	1.715610	-1.902818	-2.592245
O	-0.865807	1.167988	-3.145426
N	0.432096	2.063495	0.245868
N	2.531566	0.397900	0.493775
C	-1.850884	0.119956	0.949320
C	-1.922642	1.577338	0.978979
C	-3.001309	1.926147	1.873793
H	-3.334560	2.945104	2.108201
C	-3.601464	0.720002	2.379069
H	-4.461589	0.662717	3.059832
C	-2.905271	-0.398006	1.804084
H	-3.125240	-1.457484	1.988350
C	-3.937203	0.700134	-1.712700
H	-3.124664	0.638506	-2.447180
C	-4.491945	1.915111	-1.178957
H	-4.179097	2.935727	-1.437261
C	-5.511909	1.558404	-0.225835
H	-6.109546	2.258779	0.372950
C	-5.589279	0.120212	-0.174220
H	-6.257690	-0.466422	0.470296
C	-4.613977	-0.411045	-1.090564
H	-4.408483	-1.473674	-1.273606
C	0.274524	-1.766695	1.581675
C	1.206511	-2.784018	1.263890
H	1.420464	-3.024372	0.209624
C	1.851721	-3.505444	2.281732
H	2.564334	-4.303122	2.017737
C	1.587324	-3.213046	3.633553
H	2.094114	-3.778487	4.431494
C	0.672029	-2.197453	3.958005
H	0.456569	-1.962652	5.012595
C	0.017745	-1.478165	2.939274
H	-0.707665	-0.696060	3.210526
C	-1.385557	-2.221416	-0.710422
C	-1.600462	-2.180162	-2.105205
H	-1.207175	-1.347232	-2.703463
C	-2.322603	-3.201228	-2.749224
H	-2.476915	-3.152186	-3.838786
C	-2.840507	-4.279046	-2.009849
H	-3.403905	-5.079091	-2.515724
C	-2.627436	-4.332823	-0.620432
H	-3.022848	-5.175541	-0.031232
C	-1.901979	-3.315406	0.023607
H	-1.730185	-3.383344	1.108948
C	-0.973335	2.530214	0.246526
H	-1.280537	2.555252	-0.819379
C	0.937599	1.879995	1.598389
H	1.097200	2.845047	2.141188
H	0.224430	1.311376	2.256136
C	2.229355	1.117390	1.614551
C	3.718189	-0.275603	0.482671
C	4.570814	-0.337451	1.597267
C	4.223221	0.355383	2.764786
C	3.051344	1.116544	2.757248
C	-1.111538	3.976704	0.771610
H	-2.134650	4.366193	0.591951
H	-0.911387	4.053239	1.860717
H	-0.394543	4.628866	0.233837
C	1.497466	-0.948018	-1.938076
C	-0.179761	0.858258	-2.233524
H	2.210768	1.188242	-1.938425
H	1.885190	1.865980	-1.579220
H	1.257261	3.265152	-0.473665
O	1.765721	3.975327	-1.048377
C	2.950410	4.361173	-0.375574
H	2.728845	4.767373	0.645482
H	3.637881	3.489538	-0.215744
H	2.751285	1.711867	3.632176
H	5.513164	-0.896834	1.515802
C	3.682434	5.428479	-1.185431
H	3.035871	6.319809	-1.330326
H	4.612621	5.753369	-0.673879
H	3.956237	5.041714	-2.189922
H	4.877816	0.327715	3.648902
N	4.152876	-0.910748	-0.713655
C	4.544576	-0.243411	-1.873630
C	4.528420	-2.246907	-0.812030
C	5.143321	-1.167162	-2.718270
H	4.407435	0.838537	-1.969889
C	5.126868	-2.436404	-2.050367
H	4.307183	-2.946856	0.001466
H	5.554968	-0.948699	-3.711621
H	5.498714	-3.392279	-2.439928

```
---------------------------------------
Mn16/iv
Frequencies, energies and thermodynamic properties:
 Lowest Vibrational Mode (1/cm) =                         20.5083
 2nd Lowest Vibrational Mode (1/cm) =                     22.8947
 E(RB-P86) (a.u.) =                                     -4616.35187857
 Thermal correction to Enthalpy (a.u.) =                  0.712028
 Thermal correction to Gibbs Free Energy (a.u.) =         0.585493
 Total Entropy (cal/Kmol) =                               266.316
 E(RPBE1PBE) (a.u.) =                                   -4615.67019197
Optimised cartesian coordinates (Angstrom):
 Fe-3.724555   0.750884   0.372752
 Mn1.011555   0.579255  -0.768908
 P     -0.532589  -0.871571   0.083458
 O      1.824801  -1.436877  -2.751342
 O     -0.597955   1.733554  -2.931324
 N      0.395417   2.014344   0.702279
 N      2.490578   0.301145   0.714179
 C     -1.918721  -0.033052   0.961753
 C     -1.977418   1.403650   1.229713
 C     -3.095895   1.628807   2.117453
 H     -3.427264   2.601740   2.501483
 C     -3.735729   0.369609   2.385535
 H     -4.631732   0.221955   3.003281
 C     -3.022333  -0.653060   1.672156
 H     -3.266182  -1.723279   1.667748
 C     -3.815169   1.056324  -1.662516
 H     -2.950578   1.120425  -2.335921
 C     -4.415069   2.160194  -0.960505
 H     -4.093702   3.209250  -1.011400
 C     -5.496321   1.645536  -0.159562
 H     -6.138881   2.231694   0.511071
 C     -5.567049   0.222096  -0.370054
 H     -6.273382  -0.466589   0.112678
 C     -4.526934  -0.143242  -1.295988
 H     -4.299942  -1.157908  -1.647330
 C      0.131519  -2.046468   1.372680
 C      1.085710  -3.001638   0.943031
 H      1.350775  -3.067538  -0.125220
 C      1.691891  -3.874131   1.861478
 H      2.420470  -4.620430   1.505958
 C      1.372316  -3.795451   3.231142
 H      1.850309  -4.477494   3.952089
 C      0.439919  -2.840544   3.670451
 H      0.181943  -2.770119   4.739411
 C     -0.178138  -1.972891   2.748493
 H     -0.919756  -1.243745   3.110058
 C     -1.411295  -2.070960  -1.045605
 C     -1.540681  -1.784949  -2.422197
 H     -1.097318  -0.868869  -2.836861
 C     -2.238984  -2.660750  -3.273421
 H     -2.328382  -2.421301  -4.345080
 C     -2.816050  -3.837045  -2.762505
 H     -3.360337  -4.523569  -3.430407
 C     -2.686768  -4.135222  -1.393616
 H     -3.129271  -5.057166  -0.983409
 C     -1.986285  -3.261610  -0.543130
 H     -1.879227  -3.518333   0.522402
 C     -1.036212   2.475605   0.696150
 H     -1.264007   2.639818  -0.375672
 C      0.869212   1.583131   2.028585
 H      0.960403   2.424550   2.750573
 H      0.117493   0.885347   2.459533
 C      2.184696   0.859563   1.926701
 C      3.690311  -0.345767   0.628788
 C      4.542752  -0.533092   1.733316
 C      4.189573  -0.002931   2.978515
 C      3.000621   0.732794   3.061514
 C     -1.201358   3.825452   1.414703
 H     -2.219189   4.230271   1.246595
 H     -1.045134   3.747139   2.510101
 H     -0.478644   4.559459   1.004292
 C      1.562555  -0.625055  -1.930940
 C     -0.007269   1.238146  -2.027545
 H      2.043201   1.667470  -1.336391
 H      1.989708   3.119142  -1.135071
 H      0.959600   2.839388   0.403414
 O      1.915810   4.035724  -0.721850
 C      3.236678   4.468526  -0.381403
 H      3.119633   5.300195   0.347192
 H      3.797665   3.658870   0.144299
 H      2.687562   1.207413   4.003297
 H      5.493090  -1.064051   1.581969
 C      4.026752   4.948508  -1.598508
 H      3.485049   5.764417  -2.120843
```

```
 H      5.024344   5.331755  -1.295975
 H      4.185924   4.122710  -2.324053
 H      4.844425  -0.130699   3.853238
 N      4.148062  -0.823625  -0.630050
 C      4.532168  -0.012105  -1.695751
 C      4.596059  -2.120823  -0.864014
 C      5.200550  -0.803304  -2.617751
 H      4.284298   1.053854  -1.694144
 C      5.234964  -2.140308  -2.096202
 H      4.397255  -2.916301  -0.136871
 H      5.615863  -0.456785  -3.572567
 H      5.665245  -3.027011  -2.578587
--------------------------------------
Mn16/v
Frequencies, energies and thermodynamic properties:
 Lowest Vibrational Mode (1/cm) =                         21.1164
 2nd Lowest Vibrational Mode (1/cm) =                     31.8778
 E(RB-P86) (a.u.) =                                      -4461.41348017
 Thermal correction to Enthalpy (a.u.) =                  0.627496
 Thermal correction to Gibbs Free Energy (a.u.) =         0.515225
 Total Entropy (cal/Kmol) =                               236.294
 E(RPBE1PBE) (a.u.) =                                    -4460.72675047
Optimised cartesian coordinates (Angstrom):
 Fe3.631881  -0.546933   0.012887
 Mn-1.010199  -0.202969  -1.376975
 P      0.193683   0.419315   0.455830
 O     -2.158080   2.497125  -1.586864
 O      0.820017   0.647191  -3.502919
 N     -0.176953  -2.169734  -1.193948
 N     -2.568428  -1.216497  -0.389692
 C      1.699782  -0.613531   0.714154
 C      2.022057  -1.817019  -0.053533
 C      3.146887  -2.452361   0.596258
 H      3.648374  -3.370325   0.265179
 C      3.535309  -1.659174   1.730395
 H      4.377218  -1.869042   2.403703
 C      2.656053  -0.525568   1.802299
 H      2.695932   0.272506   2.554994
 C      3.818820   0.655830  -1.650167
 H      2.994378   0.976057  -2.300381
 C      4.592521  -0.547249  -1.808880
 H      4.466867  -1.295965  -2.602687
 C      5.546267  -0.610503  -0.731038
 H      6.269297  -1.418273  -0.555128
 C      5.364054   0.557232   0.092782
 H      5.924755   0.795088   1.006707
 C      4.295131   1.338511  -0.472591
 H      3.897247   2.278849  -0.070083
 C     -0.702341   0.263099   2.087560
 C     -1.832981   1.095719   2.278709
 H     -2.097620   1.841752   1.511028
 C     -2.616741   0.988669   3.438930
 H     -3.483784   1.654973   3.574963
 C     -2.299240   0.032022   4.423140
 H     -2.916322  -0.055060   5.331501
 C     -1.190651  -0.810821   4.236221
 H     -0.932716  -1.562863   4.999277
 C     -0.396253  -0.695896   3.078239
 H      0.478255  -1.354343   2.958823
 C      0.849529   2.161558   0.596555
 C      1.073101   2.925034  -0.570140
 H      0.835560   2.500077  -1.555719
 C      1.602811   4.225405  -0.484944
 H      1.769524   4.806514  -1.406052
 C      1.913386   4.784210   0.767877
 H      2.324977   5.803996   0.833912
 C      1.687528   4.034891   1.937025
 H      1.921585   4.465044   2.924113
 C      1.156337   2.735448   1.852312
 H      0.970729   2.167496   2.777478
 C      1.314615  -2.334207  -1.300292
 H      1.595571  -1.691218  -2.157864
 C     -0.769231  -2.834681  -0.018973
 H     -0.706839  -3.943736  -0.069855
 H     -0.184587  -2.527487   0.875753
 C     -2.201287  -2.411983   0.171351
 C     -3.876032  -0.852350  -0.231858
 C     -4.789774  -1.585203   0.549419
 H     -5.825335  -1.224837   0.625858
 C     -4.380411  -2.771992   1.165857
 C     -3.066574  -3.204162   0.939670
 H     -2.696133  -4.150668   1.360635
 C      1.710010  -3.778360  -1.649905
 H      2.789160  -3.831909  -1.895726
 H      1.517148  -4.486364  -0.818280
```

```
 H      1.152749  -4.125591  -2.544447
 C     -1.757056   1.385801  -1.496469
 C      0.134958   0.311827  -2.591075
 H     -0.581389  -2.586426  -2.044145
 H     -1.695000  -0.769026  -2.692452
 H     -5.079874  -3.363432   1.775088
 N     -4.376934   0.288997  -0.915462
 C     -4.500921   0.393794  -2.299386
 C     -5.107890   1.310708  -0.315626
 C     -5.291153   1.499183  -2.575877
 H     -3.997649  -0.326603  -2.952050
 C     -5.669632   2.085337  -1.321136
 H     -5.139684   1.403707   0.775987
 H     -5.562613   1.854179  -3.578198
 H     -6.274175   2.988481  -1.169278
---------------------------------------
Mn16/vi_R
Frequencies, energies and thermodynamic properties:
 Lowest Vibrational Mode (1/cm) =                          10.3209
 2nd Lowest Vibrational Mode (1/cm) =                      15.3050
 E(RB-P86) (a.u.) =                                      -4884.12404922
 Thermal correction to Enthalpy (a.u.) =                   0.781101
 Thermal correction to Gibbs Free Energy (a.u.) =          0.644634
 Total Entropy (cal/Kmol) =                                287.220
 E(RPBE1PBE) (a.u.) =                                    -4883.43292736
Optimised cartesian coordinates (Angstrom):
 Fe-3.067321  -2.498011  -0.622403
 Mn0.599392   0.461809   0.242925
 P     -1.584708   0.635561   0.282546
 O      0.783634   1.787705   2.887898
 O      0.740646  -1.997166   1.826819
 N      0.579949  -0.402053  -1.504693
 N      1.240878   2.086979  -0.987237
 C     -2.381420  -0.575559  -0.840730
 C     -1.652725  -1.480134  -1.723947
 C     -2.625039  -2.103688  -2.590241
 H     -2.409827  -2.853691  -3.361909
 C     -3.933979  -1.611109  -2.251395
 H     -4.879551  -1.918263  -2.718371
 C     -3.792694  -0.677287  -1.167685
 H     -4.609005  -0.129099  -0.679725
 C     -2.327093  -3.405041   1.074608
 H     -1.448458  -3.075507   1.643831
 C     -2.312130  -4.322383  -0.033547
 H     -1.424247  -4.815977  -0.451079
 C     -3.662317  -4.460852  -0.516935
 H     -3.983233  -5.074026  -1.369718
 C     -4.513268  -3.629877   0.296477
 H     -5.596680  -3.499754   0.171834
 C     -3.688514  -2.974510   1.278170
 H     -4.030370  -2.259849   2.038029
 C     -2.106675   2.283509  -0.406235
 C     -1.836469   3.438456   0.367857
 H     -1.380514   3.336876   1.366143
 C     -2.151490   4.715504  -0.122979
 H     -1.946923   5.603556   0.496018
 C     -2.725223   4.861266  -1.401412
 H     -2.969811   5.863756  -1.786839
 C     -2.983977   3.721826  -2.181576
 H     -3.433288   3.826470  -3.182029
 C     -2.678516   2.438393  -1.688273
 H     -2.902890   1.553855  -2.303479
 C     -2.582370   0.551864   1.848999
 C     -2.094132  -0.143251   2.976758
 H     -1.101240  -0.613044   2.950634
 C     -2.872873  -0.246048   4.143591
 H     -2.476008  -0.789919   5.015407
 C     -4.147806   0.344620   4.199843
 H     -4.755374   0.265221   5.115250
 C     -4.639980   1.044568   3.083187
 H     -5.634727   1.516593   3.119450
 C     -3.861824   1.151982   1.917313
 H     -4.253827   1.717848   1.057789
 C     -0.133345  -1.684736  -1.724576
 H      0.120252  -2.312108  -0.846746
 C      0.460212   0.494917  -2.644026
 H      1.034770   0.145983  -3.537508
 H     -0.600404   0.567175  -3.000484
 C      0.931456   1.874059  -2.299792
 C      1.773757   3.301399  -0.658585
 C      1.880226   4.355844  -1.579686
 H      2.323368   5.307501  -1.254921
 C      1.475977   4.151021  -2.907206
 C      1.031365   2.878865  -3.278689
 H      0.751697   2.649794  -4.317623
```

```
 C      0.339350  -2.456730  -2.972619
 H     -0.095140  -3.476869  -2.976853
 H      0.039656  -1.962738  -3.919749
 H      1.442741  -2.555225  -2.953538
 C      0.777866   1.310342   1.807279
 C      0.617532  -1.015776   1.170323
 H      2.267765  -0.914869  -1.606550
 H      3.333429  -0.626502   0.178572
 C      3.958412  -1.136039  -0.607955
 C      4.464026  -2.450929  -0.017923
 C      5.279643  -0.341942  -0.812572
 C      5.774637  -2.297106   0.485510
 C      3.801538  -3.684906   0.066029
 C      6.257048  -0.873477   0.263724
 H      5.650662  -0.606332  -1.827098
 C      6.433086  -3.382987   1.087465
 C      4.462607  -4.774923   0.667069
 H      2.782875  -3.799064  -0.338514
 H      7.324174  -0.815648  -0.037262
 C      5.769164  -4.623780   1.175026
 H      7.458752  -3.273570   1.477610
 H      3.959105  -5.752893   0.736685
 H      6.278317  -5.484814   1.637769
 O      3.199372  -1.267479  -1.789196
 H      5.137387   0.757628  -0.786582
 H      6.163481  -0.287138   1.206961
 H      1.552824   4.963388  -3.645459
 N      2.302325   3.490133   0.642461
 C      3.333874   2.728729   1.192931
 C      2.090683   4.610660   1.440625
 C      3.760545   3.359328   2.350944
 H      3.676842   1.817060   0.693771
 C      2.968558   4.545465   2.513572
 H      1.309784   5.336046   1.185680
 H      4.558830   3.002963   3.013844
 H      3.023459   5.267658   3.337747
---------------------------------------
Mn16/vi_S
Frequencies, energies and thermodynamic properties:
 Lowest Vibrational Mode (1/cm) =                         11.8096
 2nd Lowest Vibrational Mode (1/cm) =                     20.7968
 E(RB-P86) (a.u.) =                                      -4884.12567391
 Thermal correction to Enthalpy (a.u.) =                  0.780484
 Thermal correction to Gibbs Free Energy (a.u.) =         0.645567
 Total Entropy (cal/Kmol) =                               283.956
 E(RPBE1PBE) (a.u.) =                                    -4883.43664698
Optimised cartesian coordinates (Angstrom):
 Fe3.970669  -1.552975   0.510134
 Mn-0.542967  -0.146892  -0.319740
 P      1.406800   0.862441  -0.169508
 O     -1.163209   1.258953  -2.853153
 O      0.362090  -2.246480  -2.150960
 N     -0.233236  -1.154989   1.331576
 N     -1.742904   0.987486   1.027294
 C      2.577107  -0.090068   0.876450
 C      2.235057  -1.313357   1.595923
 C      3.344925  -1.620102   2.466608
 H      3.416849  -2.489054   3.132991
 C      4.364706  -0.620480   2.290125
 H      5.338865  -0.596396   2.797008
 C      3.903227   0.318895   1.304981
 H      4.454719   1.198107   0.947588
 C      3.698707  -2.462336  -1.320449
 H      2.775676  -2.439981  -1.913815
 C      4.027351  -3.439578  -0.317123
 H      3.403439  -4.292375  -0.017480
 C      5.307263  -3.089901   0.244475
 H      5.826961  -3.625414   1.050269
 C      5.771532  -1.896333  -0.415799
 H      6.707797  -1.363759  -0.201271
 C      4.776613  -1.505900  -1.380600
 H      4.820308  -0.626396  -2.035999
 C      1.250893   2.489295   0.719965
 C      0.618284   3.557341   0.038480
 H      0.283872   3.422831  -1.003173
 C      0.421444   4.792902   0.675747
 H     -0.058102   5.619686   0.127785
 C      0.836215   4.976062   2.009503
 H      0.679768   5.945114   2.509235
 C      1.451689   3.917079   2.697729
 H      1.780757   4.051634   3.740460
 C      1.660711   2.679884   2.057853
 H      2.162409   1.866256   2.603315
 C      2.403596   1.355203  -1.660942
 C      2.240402   0.684527  -2.892484
```

```
 H      1.493566  -0.114561  -2.993827
 C      3.032264   1.028389  -4.003149
 H      2.889551   0.496664  -4.957211
 C      3.996281   2.046676  -3.899148
 H      4.613790   2.316445  -4.770528
 C      4.161962   2.724913  -2.677775
 H      4.909564   3.528989  -2.587347
 C      3.368897   2.385668  -1.567847
 H      3.498428   2.938614  -0.624508
 C      0.918466  -2.083258   1.455432
 H      0.951113  -2.634483   0.494542
 C     -0.485249  -0.419227   2.562158
 H     -0.893443  -1.062565   3.379682
 H      0.459820   0.011258   2.984488
 C     -1.442352   0.709414   2.331081
 C     -2.657652   1.977437   0.797566
 C     -3.200008   2.763835   1.825997
 H     -3.933407   3.540951   1.569431
 C     -2.834509   2.509201   3.155998
 C     -1.966684   1.444103   3.409889
 H     -1.675898   1.173785   4.435767
 C      0.744415  -3.140716   2.563213
 H      1.545771  -3.903731   2.493008
 H      0.785905  -2.705221   3.582680
 H     -0.227145  -3.659106   2.436809
 C     -1.000240   0.731127  -1.809692
 C      0.062392  -1.387004  -1.388668
 H     -1.543133  -2.181272   1.267425
 H     -2.473110  -1.532211  -0.451369
 C     -2.981720  -2.458961  -0.013575
 C     -2.915688  -3.580546  -1.090470
 C     -4.131222  -3.326186  -2.016066
 O     -2.385338  -2.765323   1.213643
 H     -3.251449   3.111728   3.976876
 C     -4.474495  -2.169162   0.063199
 C     -5.188812  -1.526115   1.085965
 C     -5.131708  -2.655408  -1.089198
 C     -6.581051  -1.360309   0.943124
 H     -4.665045  -1.173765   1.989272
 C     -6.519043  -2.484875  -1.232967
 C     -7.239681  -1.833478  -0.210276
 H     -7.160649  -0.864552   1.738684
 H     -7.043657  -2.865405  -2.125139
 H     -8.329521  -1.702514  -0.308756
 H     -1.944198  -3.608339  -1.621977
 H     -3.856404  -2.634921  -2.846025
 H     -3.041173  -4.546068  -0.552677
 H     -4.529092  -4.246422  -2.492966
 N     -3.128441   2.204199  -0.523322
 C     -3.935753   1.319451  -1.238280
 C     -3.131174   3.439924  -1.163621
 C     -4.426200   1.991492  -2.347869
 H     -4.111700   0.303067  -0.869113
 C     -3.911317   3.329966  -2.306661
 H     -2.553731   4.277330  -0.756239
 H     -5.087627   1.562060  -3.110693
 H     -4.080966   4.126069  -3.042449
---------------------------------------
Mn16/viii
Frequencies, energies and thermodynamic properties:
 Lowest Vibrational Mode (1/cm) =                          22.6474
 2nd Lowest Vibrational Mode (1/cm) =                      29.7181
 E(RB-P86) (a.u.) =                                      -4461.38058224
 Thermal correction to Enthalpy (a.u.) =                   0.622925
 Thermal correction to Gibbs Free Energy (a.u.) =          0.510284
 Total Entropy (cal/Kmol) =                                237.073
 E(RPBE1PBE) (a.u.) =                                    -4460.69484768
Optimised cartesian coordinates (Angstrom):
 Fe3.649196  -0.529114   0.038075
 Mn-0.981841  -0.205268  -1.449587
 P      0.191088   0.376391   0.402669
 O     -2.147619   2.517524  -1.556931
 O      1.009181   0.788677  -3.358139
 N     -0.170467  -2.080015  -1.262768
 N     -2.562221  -1.227533  -0.421620
 C      1.691440  -0.642821   0.666462
 C      2.038543  -1.817119  -0.126123
 C      3.149374  -2.458379   0.536796
 H      3.666571  -3.362492   0.190395
 C      3.500729  -1.697807   1.707558
 H      4.321659  -1.922182   2.402245
 C      2.613352  -0.569438   1.787035
 H      2.625039   0.204218   2.565771
 C      3.900140   0.737440  -1.571862
 H      3.102513   1.088863  -2.238126
```

```
 C      4.671102  -0.463509  -1.750574
 H      4.567903  -1.180032  -2.576553
 C      5.584669  -0.576369  -0.641720
 H      6.296929  -1.395127  -0.472389
 C      5.379174   0.559685   0.221327
 H      5.908824   0.757920   1.162909
 C      4.335796   1.370217  -0.351252
 H      3.930124   2.298222   0.072036
 C     -0.770531   0.165290   1.985961
 C     -1.880859   1.018079   2.196266
 H     -2.126789   1.793368   1.452197
 C     -2.665786   0.897614   3.354840
 H     -3.518453   1.577904   3.509872
 C     -2.363118  -0.086504   4.315521
 H     -2.979126  -0.181304   5.223789
 C     -1.270137  -0.945230   4.109172
 H     -1.024401  -1.717412   4.855641
 C     -0.477149  -0.821135   2.951850
 H      0.383158  -1.492836   2.810506
 C      0.804013   2.126756   0.614725
 C      1.045812   2.949407  -0.507051
 H      0.836979   2.580506  -1.520368
 C      1.559287   4.249099  -0.345387
 H      1.738312   4.876221  -1.233186
 C      1.838269   4.747361   0.939400
 H      2.237880   5.766225   1.064754
 C      1.596119   3.938297   2.064374
 H      1.805010   4.319764   3.076623
 C      1.079114   2.640776   1.904085
 H      0.879897   2.027926   2.796793
 C      1.275361  -2.279564  -1.377350
 H      1.611098  -1.641504  -2.221350
 C     -0.748398  -2.841956  -0.184838
 H     -0.779299  -3.946341  -0.384727
 H     -0.179413  -2.769750   0.790213
 C     -2.150834  -2.409786   0.123677
 C     -3.855146  -0.850741  -0.198104
 C     -4.723025  -1.563061   0.644467
 C     -4.269144  -2.740393   1.257582
 C     -2.976220  -3.180773   0.964835
 C      1.654879  -3.729408  -1.756216
 H      2.731436  -3.805689  -2.014865
 H      1.458627  -4.447937  -0.933092
 H      1.065913  -4.047395  -2.640625
 C     -1.758520   1.404411  -1.528106
 C      0.259341   0.394508  -2.530806
 H     -1.539241  -1.217301  -2.767936
 H     -1.981810  -0.536070  -2.882765
 H     -5.753690  -1.204392   0.774062
 H     -2.583531  -4.119491   1.383337
 H     -4.931096  -3.318101   1.920221
 N     -4.381314   0.280943  -0.881257
 C     -4.594827   0.350807  -2.256877
 C     -5.019477   1.354731  -0.269444
 C     -5.347343   1.487284  -2.517195
 H     -4.231780  -0.441732  -2.919530
 C     -5.610407   2.127078  -1.260653
 H     -4.968942   1.484243   0.817486
 H     -5.677533   1.819507  -3.509470
 H     -6.160227   3.062578  -1.098338
---------------------------------------
Mn16/ix
Frequencies, energies and thermodynamic properties:
 Lowest Vibrational Mode (1/cm) =                          21.0946
 2nd Lowest Vibrational Mode (1/cm) =                      29.7360
 E(RB-P86) (a.u.) =                                       -4615.17063862
 Thermal correction to Enthalpy (a.u.) =                   0.692085
 Thermal correction to Gibbs Free Energy (a.u.) =          0.570260
 Total Entropy (cal/Kmol) =                                256.401
 E(RPBE1PBE) (a.u.) =                                     -4614.48978710
Optimised cartesian coordinates (Angstrom):
 Fe-3.731953  -0.410370  -0.479903
 Mn0.995850  -0.972301   0.523720
 P     -0.378813   0.783160   0.095536
 O      2.013583   0.457478   2.889391
 O     -0.871516  -2.215446   2.406170
 N      0.234315  -1.901734  -1.227134
 N      2.467351  -0.383515  -0.966568
 C     -1.846456   0.293516  -0.911390
 C     -2.060889  -1.024618  -1.508791
 C     -3.196635  -0.915524  -2.395242
 H     -3.629845  -1.730751  -2.988313
 C     -3.696418   0.431452  -2.344477
 H     -4.570791   0.814868  -2.887291
 C     -2.878322   1.175402  -1.427225
```

```
 H     -3.007311   2.233472  -1.165881
 C     -3.886450  -1.105602   1.456317
 H     -3.043733  -1.365315   2.108860
 C     -4.547096  -2.001494   0.544694
 H     -4.302390  -3.060859   0.389038
 C     -5.571363  -1.260216  -0.145674
 H     -6.239408  -1.652693  -0.924051
 C     -5.544388   0.095411   0.342176
 H     -6.188825   0.916237  -0.000078
 C     -4.501032   0.192210   1.329693
 H     -4.210234   1.097536   1.878193
 C      0.386482   2.153014  -0.917132
 C      1.421213   2.902848  -0.306320
 H      1.711180   2.688712   0.735618
 C      2.075502   3.926728  -1.010019
 H      2.868813   4.508619  -0.514123
 C      1.721186   4.207373  -2.344083
 H      2.235552   5.009412  -2.896879
 C      0.708651   3.456901  -2.964885
 H      0.424796   3.667252  -4.008528
 C      0.043728   2.436473  -2.256796
 H     -0.758754   1.870880  -2.754620
 C     -1.131508   1.789184   1.478330
 C     -1.282905   1.241825   2.771116
 H     -0.928114   0.225536   2.987450
 C     -1.890790   1.986266   3.798609
 H     -1.996736   1.541514   4.800904
 C     -2.355795   3.289868   3.551650
 H     -2.829388   3.872467   4.357766
 C     -2.203625   3.848129   2.269379
 H     -2.557168   4.871419   2.064959
 C     -1.592587   3.106382   1.243297
 H     -1.465070   3.567092   0.251497
 C     -1.210350  -2.271612  -1.289231
 H     -1.436625  -2.684148  -0.285806
 C      0.744225  -1.294501  -2.463488
 H      0.860524  -2.034441  -3.287087
 H     -0.000913  -0.551611  -2.825423
 C      2.059152  -0.589200  -2.249564
 C      3.690551   0.190938  -0.789022
 C      4.454180   0.702785  -1.856603
 C      3.983148   0.556063  -3.165456
 C      2.785359  -0.139270  -3.366077
 C     -1.496525  -3.376969  -2.319544
 H     -2.539250  -3.738324  -2.216144
 H     -1.361735  -3.029349  -3.364038
 H     -0.825052  -4.242754  -2.147543
 C      1.686423  -0.143684   1.924558
 C     -0.158676  -1.680582   1.622133
 O      2.055044  -2.711918   0.404935
 C      2.100267  -3.655054   1.439983
 H      1.079017  -3.975728   1.777881
 H      2.592963  -3.231558   2.359703
 H      2.397561  -0.331681  -4.377368
 H      5.433495   1.155105  -1.647920
 C      2.876284  -4.905202   1.003475
 H      2.914664  -5.659226   1.819404
 H      2.395364  -5.378395   0.120310
 H      3.920881  -4.651195   0.722156
 H      4.567699   0.937033  -4.016432
 N      4.260840   0.205586   0.505311
 C      4.415155  -0.942439   1.287055
 C      5.032129   1.235563   1.040947
 C      5.273726  -0.628031   2.329006
 H      3.854064  -1.843630   0.993308
 C      5.655953   0.749963   2.181241
 H      5.042706   2.228586   0.577426
 H      5.589328  -1.315048   3.124480
 H      6.305312   1.330859   2.848609
 H      0.830727  -2.719184  -0.939656
---------------------------------------
Mn16/x
Frequencies, energies and thermodynamic properties:
 Lowest Vibrational Mode (1/cm) =                          20.0923
 2nd Lowest Vibrational Mode (1/cm) =                      25.1497
 E(RB-P86) (a.u.) =                                     -4770.10782253
 Thermal correction to Enthalpy (a.u.) =                   0.776757
 Thermal correction to Gibbs Free Energy (a.u.) =          0.641988
 Total Entropy (cal/Kmol) =                                283.645
 E(RPBE1PBE) (a.u.) =                                   -4769.44012965
Optimised cartesian coordinates (Angstrom):
 Fe3.824421  -0.989658   0.400322
 Mn-0.881580  -0.399143  -0.799103
 P      0.752533   0.885280   0.137510
 O     -1.404664   1.833123  -2.646139
```

O	0.908023	-1.397774	-2.891611
N	-0.395569	-1.911166	0.642563
N	-2.319386	0.023828	0.776179
C	2.058398	-0.106089	0.979221
C	2.009540	-1.552914	1.186501
C	3.086105	-1.894057	2.087798
H	3.337186	-2.903723	2.436206
C	3.805726	-0.696067	2.424788
H	4.694263	-0.638123	3.067641
C	3.186595	0.403732	1.738474
H	3.507207	1.452291	1.785518
C	3.996110	-1.165892	-1.648320
H	3.168490	-1.099589	-2.365152
C	4.448030	-2.367939	-0.999822
H	4.031864	-3.374320	-1.142332
C	5.527385	-2.017196	-0.112700
H	6.073234	-2.706969	0.544902
C	5.743811	-0.596364	-0.215531
H	6.483816	-0.014787	0.350468
C	4.795426	-0.069280	-1.161839
H	4.685005	0.984158	-1.450005
C	0.161407	2.038941	1.481644
C	-0.701115	3.093209	1.094112
H	-0.964175	3.225071	0.031790
C	-1.216420	3.984490	2.049145
H	-1.874536	4.807378	1.727435
C	-0.894483	3.827681	3.411187
H	-1.300206	4.525949	4.160281
C	-0.052777	2.775006	3.807645
H	0.205564	2.642790	4.870478
C	0.473876	1.886476	2.849846
H	1.147383	1.080223	3.178437
C	1.742249	2.081616	-0.902200
C	1.900463	1.882439	-2.291179
H	1.407784	1.038433	-2.791042
C	2.691176	2.759470	-3.055896
H	2.798814	2.587672	-4.138687
C	3.336012	3.849806	-2.446098
H	3.952879	4.536918	-3.046782
C	3.180533	4.061522	-1.064249
H	3.674913	4.916046	-0.575186
C	2.387118	3.188176	-0.299983
H	2.262982	3.380977	0.776755
C	0.978795	-2.518626	0.617246
H	1.194079	-2.675127	-0.458406
C	-0.818801	-1.497985	1.991997
H	-1.028259	-2.367389	2.653041
H	0.012893	-0.939146	2.474060
C	-2.026808	-0.601125	1.953596
C	-3.412659	0.840394	0.766626
C	-4.168005	1.118729	1.924258
C	-3.826038	0.506664	3.133055
C	-2.753459	-0.391574	3.138012
C	1.010852	-3.896952	1.299050
H	1.978126	-4.401154	1.102799
H	0.881755	-3.830084	2.398617
H	0.205697	-4.540404	0.890060
C	-1.277354	0.928888	-1.895859
C	0.226192	-0.979044	-2.016212
H	-2.695237	-2.860878	-0.411888
H	-1.070476	-2.656007	0.359957
O	-2.669137	-3.681619	0.226701
C	-3.825056	-3.726432	1.048241
H	-3.612029	-4.422969	1.891810
H	-4.038417	-2.731692	1.515467
H	-2.461620	-0.929331	4.051923
H	-5.044961	1.775424	1.843645
C	-5.068860	-4.208849	0.295523
H	-4.890253	-5.207930	-0.155147
H	-5.944485	-4.286308	0.975344
H	-5.335758	-3.507495	-0.523567
O	-2.341360	-1.756462	-1.397469
C	-2.258259	-2.487980	-2.606175
C	-2.569060	-1.686205	-3.875365
H	-2.983772	-3.338617	-2.543442
H	-1.254782	-2.970100	-2.731739
H	-2.479943	-2.337574	-4.771947
H	-3.600528	-1.276170	-3.857280
H	-1.867926	-0.836007	-4.002295
H	-4.410651	0.700123	4.045015
N	-3.855291	1.411698	-0.450355
C	-4.178977	0.677985	-1.593434
C	-4.347710	2.710691	-0.583056
C	-4.858668	1.522903	-2.456861
H	-3.834378	-0.366504	-1.660630

```
 C     -4.958417   2.809698  -1.824561
 H     -4.193521   3.452633   0.208491
 H     -5.236559   1.248601  -3.450080
 H     -5.413306   3.717843  -2.240266
---------------------------------------
Mn16/TS-i
Frequencies, energies and thermodynamic properties:
 Lowest Vibrational Mode (1/cm) =-772.6449
 2nd Lowest Vibrational Mode (1/cm) =                     19.1745
 E(RB-P86) (a.u.) =                                      -4616.32187382
 Thermal correction to Enthalpy (a.u.) =                  0.703699
 Thermal correction to Gibbs Free Energy (a.u.) =         0.578240
 Total Entropy (cal/Kmol) =                               264.050
 E(RPBE1PBE) (a.u.) =                                    -4615.63913946
Optimised cartesian coordinates (Angstrom):
 Fe-3.714998   0.705648   0.352555
 Mn0.986108   0.528652  -0.941432
 P     -0.481015  -0.836141   0.150376
 O      1.774233  -1.762823  -2.633077
 O     -0.847772   1.258806  -3.111627
 N      0.389059   2.088113   0.331126
 N      2.520154   0.447105   0.539811
 C     -1.854543   0.073726   0.955794
 C     -1.952486   1.528788   1.032481
 C     -3.041040   1.832637   1.931707
 H     -3.392130   2.838061   2.196403
 C     -3.623031   0.601155   2.394226
 H     -4.485947   0.508309   3.067367
 C     -2.904758  -0.486124   1.788675
 H     -3.108154  -1.554282   1.938480
 C     -3.927558   0.704453  -1.699225
 H     -3.107802   0.676893  -2.427896
 C     -4.505285   1.894382  -1.133912
 H     -4.205630   2.926939  -1.358261
 C     -5.528114   1.493883  -0.201613
 H     -6.141155   2.166817   0.412830
 C     -5.584179   0.053956  -0.194149
 H     -6.248840  -0.561845   0.426542
 C     -4.593044  -0.434786  -1.117065
 H     -4.369757  -1.488264  -1.329718
 C      0.292382  -1.797361   1.546958
 C      1.237665  -2.796007   1.208946
 H      1.457085  -3.009869   0.150038
 C      1.889426  -3.532200   2.211881
 H      2.611308  -4.315932   1.931728
 C      1.619957  -3.272585   3.569457
 H      2.132155  -3.849491   4.355645
 C      0.692454  -2.275096   3.914399
 H      0.472836  -2.065780   4.973487
 C      0.030769  -1.541850   2.910402
 H     -0.705656  -0.776096   3.198423
 C     -1.343414  -2.208820  -0.772236
 C     -1.544953  -2.129086  -2.167377
 H     -1.156584  -1.273613  -2.736395
 C     -2.247208  -3.139994  -2.848429
 H     -2.391604  -3.060862  -3.937586
 C     -2.757803  -4.245883  -2.146330
 H     -3.305572  -5.037894  -2.681194
 C     -2.557443  -4.338113  -0.756994
 H     -2.947227  -5.203093  -0.197032
 C     -1.852126  -3.330685  -0.075980
 H     -1.689781  -3.428081   1.008599
 C     -1.032435   2.526519   0.332187
 H     -1.324890   2.566172  -0.736842
 C      0.902487   1.879409   1.684379
 H      1.034251   2.832412   2.250039
 H      0.189367   1.276556   2.301524
 C      2.211398   1.143029   1.674209
 C      3.717554  -0.206922   0.516213
 C      4.575352  -0.268927   1.627828
 C      4.223355   0.402990   2.805115
 C      3.037632   1.143470   2.812654
 C     -1.195688   3.956578   0.886436
 H     -2.223537   4.332142   0.706889
 H     -1.002469   4.014814   1.977605
 H     -0.488493   4.631691   0.363950
 C      1.532244  -0.839231  -1.942832
 C     -0.166244   0.935693  -2.200320
 H      2.167962   1.367079  -1.824366
 H      1.872135   2.110000  -1.502908
 H      1.074547   3.024802  -0.279019
 O      1.695981   3.734312  -1.036513
 C      2.900565   4.206298  -0.482186
 H      2.751949   4.578882   0.566595
 H      3.671302   3.390764  -0.399474
```

```
 H      2.731174   1.721774   3.696688
 H      5.525746  -0.812760   1.535239
 C      3.486782   5.341840  -1.326045
 H      2.767726   6.185821  -1.395359
 H      4.433813   5.726394  -0.889863
 H      3.698731   4.993923  -2.359635
 H      4.882824   0.375907   3.685540
 N      4.160202  -0.823125  -0.686758
 C      4.550044  -0.138672  -1.837607
 C      4.563831  -2.151123  -0.792167
 C      5.174083  -1.043421  -2.683711
 H      4.378942   0.938341  -1.931580
 C      5.176476  -2.318620  -2.026440
 H      4.351902  -2.861177   0.014977
 H      5.587716  -0.809406  -3.672695
 H      5.570149  -3.263883  -2.420505
---------------------------------------
Mn16/TS-ii_si
Frequencies, energies and thermodynamic properties:
 Lowest Vibrational Mode (1/cm) =-292.9294
 2nd Lowest Vibrational Mode (1/cm) =                     16.2315
 E(RB-P86) (a.u.) =                                      -4884.11596463
 Thermal correction to Enthalpy (a.u.) =                  0.776718
 Thermal correction to Gibbs Free Energy (a.u.) =         0.644557
 Total Entropy (cal/Kmol) =                               278.155
 E(RPBE1PBE) (a.u.) =                                    -4883.41958105
Optimised cartesian coordinates (Angstrom):
 Fe-3.821884  -1.211317  -0.653915
 Mn0.899974  -0.130133   0.048184
 P     -1.101572   0.948343   0.318485
 O      2.143292   0.370105   2.665452
 O      0.027310  -2.579562   1.397625
 N      0.327633  -0.856791  -1.834382
 N      1.913033   1.262618  -1.207919
 C     -2.390253   0.251837  -0.815015
 C     -2.169841  -0.806530  -1.801218
 C     -3.355245  -0.882285  -2.624540
 H     -3.521886  -1.588047  -3.447780
 C     -4.303397   0.093051  -2.162383
 H     -5.309489   0.257921  -2.570692
 C     -3.718547   0.788282  -1.051206
 H     -4.195239   1.598485  -0.485856
 C     -3.430316  -2.538205   0.873486
 H     -2.449252  -2.697996   1.339824
 C     -3.939479  -3.232508  -0.280011
 H     -3.419830  -4.020620  -0.841414
 C     -5.241390  -2.696221  -0.585105
 H     -5.884265  -2.998830  -1.422564
 C     -5.538236  -1.671618   0.383113
 H     -6.448243  -1.057471   0.413324
 C     -4.419408  -1.572645   1.283398
 H     -4.325085  -0.870519   2.121662
 C     -1.206098   2.765220  -0.116594
 C     -0.967721   3.754574   0.867427
 H     -0.806842   3.459231   1.916174
 C     -0.954332   5.119930   0.529702
 H     -0.775516   5.871975   1.314739
 C     -1.171506   5.524045  -0.799864
 H     -1.161953   6.593274  -1.064086
 C     -1.409334   4.551313  -1.787183
 H     -1.591489   4.854354  -2.830638
 C     -1.427653   3.186435  -1.448060
 H     -1.645681   2.445392  -2.232987
 C     -1.935106   0.956512   1.991639
 C     -1.454522   0.137964   3.035457
 H     -0.563157  -0.483694   2.880753
 C     -2.104710   0.106293   4.283871
 H     -1.711377  -0.540507   5.084186
 C     -3.243700   0.897114   4.510141
 H     -3.751483   0.873724   5.487454
 C     -3.726270   1.727094   3.480620
 H     -4.613180   2.358872   3.647999
 C     -3.076716   1.758234   2.235320
 H     -3.460382   2.429076   1.451959
 C     -0.927357  -1.669811  -1.960843
 H     -0.879260  -2.377662  -1.109987
 C      0.450530   0.202328  -2.848997
 H      0.537653  -0.200727  -3.880696
 H     -0.469276   0.828579  -2.828544
 C      1.636219   1.069453  -2.538390
 C      2.942843   2.115237  -0.924205
 C      3.732637   2.727515  -1.916528
 H      4.535412   3.409601  -1.603427
 C      3.466514   2.479756  -3.266735
 C      2.388179   1.641497  -3.575678
```

```
 H      2.119968   1.417955  -4.618546
 C     -0.941963  -2.509910  -3.249568
 H     -1.778289  -3.236442  -3.224784
 H     -1.060271  -1.891936  -4.162825
 H      0.001811  -3.085765  -3.332755
 C      1.646859   0.278564   1.596075
 C      0.329079  -1.573718   0.846975
 H      1.136626  -1.522072  -2.013820
 H      2.250895  -1.072769  -0.209695
 C      3.182985  -2.109483  -1.090822
 C      3.271133  -3.161565  -0.014257
 C      4.474868  -1.268695  -0.926621
 C      4.303669  -2.835898   0.893111
 C      2.541707  -4.355669   0.103123
 C      4.932690  -1.502641   0.529582
 H      5.210221  -1.706514  -1.640263
 C      4.609909  -3.712421   1.947279
 C      2.852997  -5.233525   1.156763
 H      1.751216  -4.594481  -0.626191
 H      6.035242  -1.499919   0.655336
 C      3.877999  -4.910894   2.072706
 H      5.415834  -3.476300   2.661620
 H      2.300818  -6.180627   1.268012
 H      4.114442  -5.609743   2.891442
 O      2.582734  -2.270786  -2.195083
 H      4.339176  -0.207832  -1.209639
 H      4.531184  -0.707274   1.197901
 H      4.074545   2.943948  -4.057354
 N      3.247993   2.440767   0.425862
 C      4.517390   2.338921   0.990322
 C      2.444377   3.215018   1.258066
 C      4.505698   3.018136   2.201336
 H      5.310022   1.778379   0.482066
 C      3.195319   3.578018   2.366171
 H      1.414381   3.445028   0.969979
 H      5.348552   3.090751   2.900170
 H      2.836379   4.180816   3.209820
--------------------------------------
Mn16/TS-ii_re
Frequencies, energies and thermodynamic properties:
 Lowest Vibrational Mode (1/cm) =-366.5368
 2nd Lowest Vibrational Mode (1/cm) =                      18.4921
 E(RB-P86) (a.u.) =                                       -4884.11871538
 Thermal correction to Enthalpy (a.u.) =                   0.776523
 Thermal correction to Gibbs Free Energy (a.u.) =          0.645134
 Total Entropy (cal/Kmol) =                                276.532
 E(RPBE1PBE) (a.u.) =                                     -4883.42518001
Optimised cartesian coordinates (Angstrom):
 Fe4.078798  -1.216596   0.434768
 Mn-0.649525  -0.371343  -0.356859
 P      1.220324   0.892522  -0.106885
 O     -1.356299   1.037407  -2.844287
 O      0.506200  -2.323091  -2.211336
 N     -0.134766  -1.455972   1.361017
 N     -1.834189   0.635201   1.076053
 C      2.521328   0.045622   0.884068
 C      2.351882  -1.245025   1.548102
 C      3.506714  -1.448178   2.392597
 H      3.699904  -2.329692   3.016670
 C      4.387941  -0.321048   2.250968
 H      5.360926  -0.198546   2.745398
 C      3.792271   0.595918   1.319382
 H      4.220990   1.553770   0.998019
 C      3.888993  -2.061489  -1.436106
 H      2.960321  -2.119980  -2.018191
 C      4.346523  -3.040191  -0.485600
 H      3.832873  -3.974792  -0.222818
 C      5.584807  -2.568562   0.079700
 H      6.176732  -3.076710   0.852741
 C      5.894306  -1.298025  -0.525044
 H      6.764240  -0.668869  -0.293511
 C      4.845474  -0.982588  -1.459478
 H      4.773924  -0.073178  -2.070017
 C      0.966214   2.482853   0.833999
 C      0.268044   3.530113   0.185593
 H     -0.051042   3.408993  -0.862495
 C     -0.010980   4.730455   0.859804
 H     -0.537073   5.543090   0.333295
 C      0.382694   4.898663   2.201496
 H      0.161929   5.839487   2.730059
 C      1.061028   3.858812   2.859347
 H      1.375785   3.979694   3.908255
 C      1.353943   2.660041   2.180498
 H      1.908647   1.867142   2.705604
 C      2.152753   1.520691  -1.596033
```

```
 C      2.036403   0.864313  -2.840520
 H      1.366355   0.000731  -2.947553
 C      2.776242   1.302722  -3.954026
 H      2.669967   0.778767  -4.917232
 C      3.642714   2.404206  -3.841080
 H      4.219823   2.748152  -4.714109
 C      3.761977   3.069186  -2.606908
 H      4.433299   3.937179  -2.507982
 C      3.020086   2.634139  -1.494820
 H      3.112993   3.177244  -0.541590
 C      1.172152  -2.195715   1.400327
 H      1.236912  -2.684018   0.407940
 C     -0.393000  -0.663894   2.573160
 H     -0.589254  -1.298858   3.464893
 H      0.514744  -0.064976   2.812064
 C     -1.539770   0.286340   2.367805
 C     -2.838211   1.546342   0.906074
 C     -3.521498   2.145275   1.980097
 H     -4.320789   2.865930   1.757470
 C     -3.198521   1.790059   3.294222
 C     -2.200421   0.827807   3.482646
 H     -1.911818   0.494400   4.490463
 C      1.169219  -3.312631   2.458232
 H      2.064254  -3.955421   2.340224
 H      1.171463  -2.918395   3.495042
 H      0.273944  -3.951828   2.322103
 C     -1.149085   0.509562  -1.807074
 C      0.095763  -1.515317  -1.442143
 H     -0.890014  -2.208268   1.323061
 H     -1.950455  -1.460637  -0.437887
 C     -2.715995  -2.837186   0.095180
 C     -4.111353  -2.272079   0.028866
 C     -2.548459  -3.634259  -1.224637
 C     -4.643659  -2.449084  -1.268438
 C     -4.882720  -1.731414   1.071533
 C     -3.601318  -3.052968  -2.193223
 C     -5.972591  -2.077463  -1.534191
 C     -6.211387  -1.357067   0.800737
 H     -4.449553  -1.623402   2.078962
 C     -6.750455  -1.530603  -0.492854
 H     -6.407207  -2.217091  -2.537625
 H     -6.840399  -0.935817   1.601279
 H     -7.795319  -1.240759  -0.688972
 O     -2.153158  -3.161768   1.185555
 H     -4.029189  -3.804074  -2.888940
 H     -3.154280  -2.251882  -2.825300
 H     -1.513083  -3.632447  -1.610694
 H     -2.806677  -4.686016  -0.962883
 H     -3.728512   2.238411   4.147740
 N     -3.253124   1.924240  -0.403286
 C     -4.050276   1.149342  -1.242086
 C     -3.241070   3.234893  -0.874226
 C     -4.513148   1.962651  -2.265910
 H     -4.211746   0.088840  -1.027069
 C     -3.996339   3.281293  -2.038527
 H     -2.683933   4.010557  -0.337939
 H     -5.150859   1.637615  -3.097542
 H     -4.149007   4.169151  -2.665139
---------------------------------------
Mn16/TS-iii
Frequencies, energies and thermodynamic properties:
 Lowest Vibrational Mode (1/cm) =-667.0235
 2nd Lowest Vibrational Mode (1/cm) =                    21.9732
 E(RB-P86) (a.u.) =                                    -4461.37690522
 Thermal correction to Enthalpy (a.u.) =                 0.621692
 Thermal correction to Gibbs Free Energy (a.u.) =        0.509755
 Total Entropy (cal/Kmol) =                              235.592
 E(RPBE1PBE) (a.u.) =                                  -4460.68796114
Optimised cartesian coordinates (Angstrom):
 Fe3.630152  -0.544282  -0.000325
 Mn-0.977472  -0.211261  -1.415913
 P      0.188116   0.408515   0.436246
 O     -2.179027   2.476130  -1.700320
 O      0.983122   0.790556  -3.348846
 N     -0.204307  -2.139150  -1.227824
 N     -2.567995  -1.207595  -0.378078
 C      1.681770  -0.637291   0.661378
 C      2.010639  -1.820687  -0.133594
 C      3.126658  -2.465972   0.518543
 H      3.632532  -3.375459   0.170076
 C      3.501737  -1.702582   1.678706
 H      4.333167  -1.928839   2.359979
 C      2.623177  -0.568942   1.765962
 H      2.653767   0.209785   2.539174
 C      3.872639   0.723622  -1.611386
```

```
 H      3.069911   1.086390  -2.265023
 C      4.625883  -0.486510  -1.804374
 H      4.502103  -1.199194  -2.630865
 C      5.554625  -0.613183  -0.709998
 H      6.259138  -1.441059  -0.553126
 C      5.376029   0.523029   0.158671
 H      5.921746   0.711960   1.092939
 C      4.334144   1.347646  -0.396142
 H      3.945866   2.279132   0.035786
 C     -0.742033   0.207308   2.040310
 C     -1.867167   1.042736   2.242857
 H     -2.134572   1.796602   1.483987
 C     -2.641979   0.930494   3.408914
 H     -3.507365   1.596462   3.555385
 C     -2.314984  -0.029594   4.385873
 H     -2.923464  -0.119156   5.299748
 C     -1.208540  -0.872936   4.187024
 H     -0.944728  -1.627547   4.945279
 C     -0.425969  -0.756437   3.021853
 H      0.443124  -1.418099   2.885749
 C      0.825274   2.150182   0.635529
 C      1.045543   2.965853  -0.495896
 H      0.814600   2.589881  -1.501950
 C      1.565361   4.265164  -0.352057
 H      1.728064   4.887113  -1.246640
 C      1.870798   4.769925   0.924261
 H      2.275145   5.788541   1.035836
 C      1.648640   3.968215   2.058737
 H      1.877993   4.355572   3.064331
 C      1.125909   2.670876   1.916332
 H      0.941488   2.063471   2.816026
 C      1.244005  -2.319992  -1.368506
 H      1.548769  -1.692650  -2.231833
 C     -0.774016  -2.843768  -0.100690
 H     -0.808779  -3.954199  -0.245153
 H     -0.176975  -2.706629   0.844499
 C     -2.171082  -2.378409   0.201062
 C     -3.858626  -0.815848  -0.177413
 C     -4.731326  -1.486407   0.696797
 C     -4.287196  -2.642068   1.353005
 C     -3.001526  -3.111859   1.068437
 C      1.629093  -3.772947  -1.723467
 H      2.703926  -3.847801  -1.988147
 H      1.441142  -4.476487  -0.885808
 H      1.037244  -4.109804  -2.598646
 C     -1.772381   1.374430  -1.591346
 C      0.252244   0.386677  -2.508131
 H     -1.150543  -1.592414  -2.366517
 H     -1.678440  -0.951738  -2.786137
 H     -5.759768  -1.116690   0.811717
 H     -2.621545  -4.043032   1.514250
 H     -4.952816  -3.187243   2.039131
 N     -4.377369   0.275828  -0.922901
 C     -4.514494   0.289832  -2.310677
 C     -5.091015   1.343225  -0.385883
 C     -5.296134   1.382979  -2.652298
 H     -4.058261  -0.496137  -2.921079
 C     -5.654375   2.055212  -1.435822
 H     -5.106236   1.513117   0.696681
 H     -5.580080   1.669525  -3.672730
 H     -6.248067   2.972844  -1.338820
---------------------------------------
Mn17/i
Frequencies, energies and thermodynamic properties:
 Lowest Vibrational Mode (1/cm) =                        21.8809
 2nd Lowest Vibrational Mode (1/cm) =                    23.5728
 E(RB-P86) (a.u.) =                                     -4648.02165815
 Thermal correction to Enthalpy (a.u.) =                 0.514035
 Thermal correction to Gibbs Free Energy (a.u.) =        0.402208
 Total Entropy (cal/Kmol) =                              235.360
 E(RPBE1PBE) (a.u.) =                                   -4647.46362916
Optimised cartesian coordinates (Angstrom):
 Fe3.303741  -1.185866   0.646081
 Mn-0.688141  -0.022221  -1.673128
 P      0.338863   0.754175   0.101259
 O     -1.235327   2.555411  -3.047201
 O      1.637477  -0.024950  -3.455964
 N     -0.527150  -1.840075  -1.146080
 N     -2.607729  -0.309549  -0.882095
 C      1.354657  -0.583705   0.857985
 C      1.412992  -1.962392   0.376642
 C      2.144741  -2.727255   1.357823
 H      2.385544  -3.795864   1.289317
 C      2.542465  -1.851166   2.427977
 H      3.131194  -2.136341   3.310416
```

```
 C      2.066093  -0.529974   2.122714
 H      2.208947   0.362770   2.745038
 C      4.152561  -0.451000  -1.082191
 H      3.601482  -0.067181  -1.950482
 C      4.542093  -1.820307  -0.873303
 H      4.346513  -2.658329  -1.555873
 C      5.212292  -1.905185   0.399357
 H      5.612181  -2.818974   0.858989
 C      5.239910  -0.585076   0.976133
 H      5.665345  -0.316988   1.952558
 C      4.582686   0.313562   0.062724
 H      4.420030   1.388225   0.216831
 C     -0.872189   1.182643   1.445750
 C     -1.594705   2.395505   1.340688
 H     -1.372017   3.099022   0.522302
 C     -2.594993   2.712795   2.273921
 H     -3.141675   3.665166   2.184929
 C     -2.900637   1.817099   3.317244
 H     -3.686611   2.066056   4.047735
 C     -2.198085   0.604207   3.420276
 H     -2.430360  -0.102262   4.233201
 C     -1.188984   0.286559   2.490857
 H     -0.637240  -0.660736   2.591427
 C      1.438057   2.251814   0.084089
 C      1.986840   2.717182  -1.130461
 H      1.739785   2.214499  -2.076305
 C      2.853941   3.825118  -1.143406
 H      3.272886   4.176770  -2.099572
 C      3.180285   4.483608   0.055352
 H      3.857304   5.352497   0.043609
 C      2.629764   4.033021   1.269545
 H      2.872288   4.548288   2.212606
 C      1.761586   2.927738   1.284302
 H      1.323490   2.599206   2.239763
 C      0.788321  -2.473600  -0.931464
 H      1.428907  -2.116823  -1.761809
 C     -1.579445  -2.440527  -0.351237
 H     -1.873989  -3.454191  -0.717812
 H     -1.268717  -2.606197   0.715219
 C     -2.782654  -1.556560  -0.345938
 C     -3.649199   0.538328  -0.851313
 C     -4.910568   0.209900  -0.332449
 C     -5.104148  -1.076781   0.199452
 C     -4.013322  -1.961742   0.184955
 C      0.775951  -4.011102  -1.011935
 H      1.813708  -4.400750  -1.033001
 H      0.259182  -4.484547  -0.152184
 H      0.275390  -4.337915  -1.946225
 C     -1.067016   1.538263  -2.469137
 C      0.734216  -0.028090  -2.686674
 F     -3.492170   1.764487  -1.344141
 F     -5.905140   1.100647  -0.340206
 F     -6.282690  -1.435617   0.702459
 F     -4.154925  -3.193248   0.696710
---------------------------------------
Mn17/ii
Frequencies, energies and thermodynamic properties:
 Lowest Vibrational Mode (1/cm) =                       18.8790
 2nd Lowest Vibrational Mode (1/cm) =                   20.3567
 E(RB-P86) (a.u.) =                                   -4802.95587118
 Thermal correction to Enthalpy (a.u.) =                0.598751
 Thermal correction to Gibbs Free Energy (a.u.) =       0.471724
 Total Entropy (cal/Kmol) =                             267.352
 E(RPBE1PBE) (a.u.) =                                 -4802.40639170
Optimised cartesian coordinates (Angstrom):
 Fe3.395557   0.926875  -1.137031
 Mn-0.688221   0.413752   1.265314
 P      0.645148  -0.949489   0.179850
 O     -1.034464  -1.251260   3.698350
 O      1.394204   1.664952   2.904063
 N     -0.688967   1.754759  -0.117730
 N     -2.509503  -0.058915   0.335774
 C      1.576788  -0.018630  -1.103905
 C      1.407381   1.404134  -1.394354
 C      2.159246   1.686994  -2.593476
 H      2.258650   2.670311  -3.070652
 C      2.792449   0.475630  -3.042600
 H      3.449986   0.377124  -3.916903
 C      2.445100  -0.575654  -2.126390
 H      2.773493  -1.620708  -2.194891
 C      4.108447   1.293227   0.762198
 H      3.508697   1.294184   1.681374
 C      4.340545   2.429082  -0.089794
 H      3.953125   3.444556   0.068444
 C      5.148672   1.996060  -1.201178
```

```
 H      5.481441   2.621892  -2.040076
 C      5.418818   0.590671  -1.033717
 H      5.994168  -0.041765  -1.723074
 C      4.773795   0.154819   0.177751
 H      4.772624  -0.866985   0.578969
 C     -0.336980  -2.185600  -0.801139
 C     -0.940279  -3.262581  -0.107811
 H     -0.757429  -3.393922   0.970874
 C     -1.771693  -4.169214  -0.784968
 H     -2.226985  -5.007731  -0.234330
 C     -2.025128  -4.006250  -2.161021
 H     -2.678853  -4.717008  -2.690866
 C     -1.440058  -2.932571  -2.853769
 H     -1.631972  -2.798353  -3.930277
 C     -0.599680  -2.025680  -2.179611
 H     -0.136367  -1.197585  -2.737539
 C      1.900850  -2.034963   1.012692
 C      2.359889  -1.731978   2.312847
 H      1.954335  -0.866917   2.855874
 C      3.340798  -2.531768   2.927190
 H      3.687008  -2.282861   3.942820
 C      3.872421  -3.644807   2.252386
 H      4.638501  -4.271632   2.735698
 C      3.413805  -3.959894   0.959906
 H      3.817774  -4.835302   0.426963
 C      2.431865  -3.164034   0.345210
 H      2.068925  -3.435278  -0.658485
 C      0.572005   2.389745  -0.567466
 H      1.137636   2.602714   0.361263
 C     -1.662566   1.628912  -1.191124
 H     -2.113619   2.605933  -1.489063
 H     -1.198601   1.231695  -2.132613
 C     -2.749579   0.688646  -0.784876
 C     -3.438901  -0.952761   0.709153
 C     -4.652820  -1.147265   0.032397
 C     -4.915707  -0.370344  -1.108684
 C     -3.940135   0.557390  -1.510679
 C      0.368693   3.740480  -1.277976
 H      1.340714   4.257881  -1.406180
 H     -0.090276   3.635648  -2.282376
 H     -0.282426   4.381826  -0.650124
 C     -0.944459  -0.616684   2.706687
 C      0.595965   1.148162   2.196939
 H     -1.370219   3.068137   1.047446
 O     -1.651695   3.871418   1.572488
 C     -3.064706   3.998448   1.476786
 H     -3.581080   3.052683   1.778986
 H     -3.387393   4.209872   0.424967
 C     -3.529884   5.135463   2.379663
 H     -4.630269   5.265363   2.320163
 H     -3.259786   4.931985   3.437221
 H     -3.050966   6.092535   2.083454
 F     -4.151433   1.305348  -2.602457
 F     -6.051816  -0.516337  -1.783514
 F     -5.535902  -2.050933   0.460635
 F     -3.212162  -1.702902   1.783782
---------------------------------------
Mn17/iii
Frequencies, energies and thermodynamic properties:
 Lowest Vibrational Mode (1/cm) =                        15.9853
 2nd Lowest Vibrational Mode (1/cm) =                    23.7898
 E(RB-P86) (a.u.) =                                     -4804.12942491
 Thermal correction to Enthalpy (a.u.) =                 0.614868
 Thermal correction to Gibbs Free Energy (a.u.) =        0.488303
 Total Entropy (cal/Kmol) =                              266.378
 E(RPBE1PBE) (a.u.) =                                   -4803.57705833
Optimised cartesian coordinates (Angstrom):
 Fe3.402903   0.983130  -1.079850
 Mn-0.718792   0.362543   1.469462
 P      0.622037  -0.901223   0.131413
 O     -0.759062  -1.774489   3.518366
 O      1.458583   1.560662   3.031544
 N     -0.703440   1.865517   0.013476
 N     -2.476422  -0.112114   0.395008
 C      1.562083   0.075994  -1.095207
 C      1.421347   1.516698  -1.285452
 C      2.170523   1.858189  -2.471961
 H      2.287909   2.867323  -2.886675
 C      2.777395   0.666844  -3.003285
 H      3.428207   0.612570  -3.886411
 C      2.415748  -0.434522  -2.154290
 H      2.721712  -1.479758  -2.290764
 C      4.148970   1.214481   0.828267
 H      3.564719   1.162604   1.755570
 C      4.381793   2.401703   0.050225
```

```
 H      4.008618   3.408466   0.281248
 C      5.167288   2.033918  -1.100200
 H      5.494275   2.709997  -1.901559
 C      5.423348   0.617588  -1.029902
 H      5.980811   0.026008  -1.768489
 C      4.791604   0.109540   0.159976
 H      4.785995  -0.936107   0.493871
 C     -0.365075  -2.096306  -0.898653
 C     -1.004842  -3.170617  -0.234131
 H     -0.863740  -3.311472   0.849650
 C     -1.816110  -4.068536  -0.946891
 H     -2.301072  -4.903496  -0.416684
 C     -2.010035  -3.901526  -2.331673
 H     -2.647206  -4.605487  -2.889924
 C     -1.385653  -2.832787  -2.996971
 H     -1.529617  -2.695306  -4.080456
 C     -0.567196  -1.933774  -2.286050
 H     -0.074675  -1.109138  -2.823721
 C      1.885993  -2.043664   0.883614
 C      2.416034  -1.794889   2.168219
 H      2.059371  -0.941864   2.761483
 C      3.409718  -2.632752   2.706470
 H      3.809691  -2.424199   3.711339
 C      3.886355  -3.731302   1.969997
 H      4.662741  -4.387933   2.393528
 C      3.359330  -3.992089   0.691886
 H      3.719982  -4.854555   0.109093
 C      2.363621  -3.158323   0.154339
 H      1.949048  -3.387997  -0.839471
 C      0.588792   2.453976  -0.406197
 H      1.155784   2.621022   0.532572
 C     -1.541470   1.542659  -1.135244
 H     -1.972290   2.444374  -1.634267
 H     -0.979445   1.012229  -1.953221
 C     -2.659988   0.632983  -0.735448
 C     -3.433986  -0.974074   0.756722
 C     -4.629930  -1.161154   0.043676
 C     -4.835640  -0.397686  -1.115554
 C     -3.830294   0.507803  -1.497102
 C      0.418354   3.842156  -1.061198
 H      1.401668   4.337329  -1.196532
 H     -0.066773   3.785291  -2.057860
 H     -0.201434   4.482600  -0.402060
 C     -0.795416  -0.934070   2.693882
 C      0.621661   1.061071   2.364833
 H     -1.885538   1.020734   2.561325
 H     -1.659349   1.709219   2.152363
 H     -1.432525   3.055327   0.859676
 O     -1.849899   3.755450   1.510419
 C     -3.195893   3.998635   1.140918
 H     -3.271066   4.362942   0.083763
 H     -3.812712   3.062960   1.188316
 C     -3.807024   5.043547   2.071005
 H     -3.234129   5.993593   2.020103
 H     -4.860825   5.258127   1.795699
 H     -3.788594   4.692263   3.124401
 F     -3.999944   1.241878  -2.605429
 F     -5.950307  -0.529620  -1.827220
 F     -5.545056  -2.037768   0.460082
 F     -3.261598  -1.698863   1.860201
---------------------------------------
Mn17/iv
Frequencies, energies and thermodynamic properties:
 Lowest Vibrational Mode (1/cm) =                         16.1264
 2nd Lowest Vibrational Mode (1/cm) =                     19.5072
 E(RB-P86) (a.u.) =                                     -4804.15740363
 Thermal correction to Enthalpy (a.u.) =                  0.619374
 Thermal correction to Gibbs Free Energy (a.u.) =         0.492334
 Total Entropy (cal/Kmol) =                               267.376
 E(RPBE1PBE) (a.u.) =                                   -4803.60314613
Optimised cartesian coordinates (Angstrom):
 Fe-3.449816  -0.854823  -1.127665
 Mn0.766106  -0.518169   1.303070
 P     -0.631729   0.909626   0.203905
 O      0.985166   1.177507   3.694994
 O     -1.233537  -2.014270   2.843252
 N      0.689938  -1.865312  -0.382388
 N      2.479741   0.056187   0.243986
 C     -1.631206   0.093503  -1.107695
 C     -1.485089  -1.307379  -1.505763
 C     -2.288843  -1.500733  -2.692015
 H     -2.415130  -2.444627  -3.236821
 C     -2.936184  -0.260247  -3.021137
 H     -3.633939  -0.100165  -3.853973
 C     -2.541892   0.720155  -2.048389
```

```
 H     -2.869247   1.767442  -2.021836
 C     -4.067059  -1.378499   0.766110
 H     -3.417775  -1.458627   1.647721
 C     -4.351709  -2.437215  -0.166310
 H     -3.965609  -3.464180  -0.115187
 C     -5.213650  -1.908753  -1.192457
 H     -5.593415  -2.459559  -2.063386
 C     -5.465354  -0.522696  -0.891471
 H     -6.071182   0.167984  -1.493224
 C     -4.755178  -0.193376   0.316717
 H     -4.725747   0.790283   0.802717
 C      0.276387   2.246160  -0.726993
 C      0.980783   3.207219   0.039399
 H      0.913667   3.187280   1.139516
 C      1.764039   4.189987  -0.587600
 H      2.296862   4.935091   0.024672
 C      1.872430   4.220778  -1.991636
 H      2.489151   4.989824  -2.483141
 C      1.190335   3.262392  -2.760134
 H      1.268533   3.276775  -3.859108
 C      0.396633   2.281458  -2.133517
 H     -0.142418   1.548252  -2.753371
 C     -1.877372   1.925221   1.147269
 C     -2.326892   1.492106   2.413648
 H     -1.923015   0.569257   2.853795
 C     -3.294817   2.229376   3.120036
 H     -3.633806   1.876902   4.107213
 C     -3.823441   3.412070   2.572927
 H     -4.578892   3.990589   3.128078
 C     -3.375255   3.857036   1.315483
 H     -3.777506   4.786443   0.881502
 C     -2.406622   3.121867   0.609781
 H     -2.053013   3.492803  -0.365076
 C     -0.656246  -2.381433  -0.812529
 H     -1.156510  -2.648510   0.139272
 C      1.505300  -1.358005  -1.503324
 H      1.885329  -2.165125  -2.165252
 H      0.866771  -0.703758  -2.139022
 C      2.647739  -0.538239  -0.977882
 C      3.462183   0.848371   0.690921
 C      4.659272   1.093662  -0.006313
 C      4.846685   0.475268  -1.249612
 C      3.812743  -0.351209  -1.729583
 C     -0.532077  -3.668196  -1.645483
 H     -1.529045  -4.127750  -1.796009
 H     -0.089492  -3.486567  -2.646182
 H      0.097264  -4.406057  -1.107782
 C      0.933031   0.523107   2.710650
 C     -0.464771  -1.389773   2.189835
 H      1.791034  -1.537726   1.997305
 H      1.862934  -2.990124   1.749679
 H      1.188748  -2.684360   0.030501
 O      1.944488  -3.879448   1.284187
 C      3.333862  -4.222129   1.240392
 H      3.433662  -5.037908   0.491858
 H      3.944498  -3.365991   0.864712
 C      3.863499  -4.687272   2.596263
 H      3.274690  -5.549373   2.973066
 H      4.926505  -4.999524   2.518778
 H      3.802937  -3.873452   3.349785
 F      3.323172   1.439655   1.875561
 F      5.593680   1.894513   0.509422
 F      5.960453   0.666397  -1.951537
 F      3.954627  -0.946594  -2.922893
---------------------------------------
Mn17/v
Frequencies, energies and thermodynamic properties:
 Lowest Vibrational Mode (1/cm) =                          18.8975
 2nd Lowest Vibrational Mode (1/cm) =                      21.6973
 E(RB-P86) (a.u.) =                                      -4649.21902690
 Thermal correction to Enthalpy (a.u.) =                   0.535023
 Thermal correction to Gibbs Free Energy (a.u.) =          0.422559
 Total Entropy (cal/Kmol) =                                236.700
 E(RPBE1PBE) (a.u.) =                                    -4648.66087281
Optimised cartesian coordinates (Angstrom):
 Fe3.314430  -1.153656   0.630782
 Mn-0.753109   0.054758  -1.763763
 P      0.308660   0.727735   0.134408
 O     -1.171089   2.759637  -2.836306
 O      1.520995  -0.065701  -3.611957
 N     -0.523669  -2.003058  -1.161613
 N     -2.594314  -0.301600  -0.846348
 C      1.359667  -0.586584   0.883221
 C      1.445630  -1.962114   0.388464
 C      2.205626  -2.719604   1.358161
```

```
 H      2.469577  -3.781772   1.282709
 C      2.600125  -1.842001   2.425670
 H      3.210877  -2.120923   3.294723
 C      2.089226  -0.531412   2.136630
 H      2.224108   0.362437   2.759120
 C      4.113479  -0.385557  -1.104553
 H      3.536166  -0.002285  -1.956092
 C      4.531564  -1.750371  -0.919358
 H      4.337505  -2.584490  -1.607102
 C      5.231058  -1.836676   0.336971
 H      5.656038  -2.748318   0.777885
 C      5.249021  -0.522653   0.926882
 H      5.690317  -0.257522   1.896991
 C      4.556671   0.374054   0.038456
 H      4.378862   1.443788   0.208215
 C     -0.858032   1.169451   1.521023
 C     -1.668565   2.318064   1.344650
 H     -1.534630   2.950650   0.451825
 C     -2.641934   2.662590   2.296643
 H     -3.255305   3.565636   2.147023
 C     -2.837450   1.855863   3.434687
 H     -3.602990   2.124456   4.179822
 C     -2.050923   0.704794   3.610469
 H     -2.196058   0.065559   4.496211
 C     -1.067325   0.363107   2.661400
 H     -0.450965  -0.534649   2.824409
 C      1.416481   2.226439   0.117494
 C      1.984738   2.662871  -1.098889
 H      1.750039   2.132795  -2.033178
 C      2.852462   3.770032  -1.126950
 H      3.287038   4.097303  -2.084977
 C      3.159667   4.459538   0.059655
 H      3.836619   5.328367   0.036701
 C      2.589668   4.038909   1.275635
 H      2.817385   4.577860   2.209284
 C      1.721234   2.933459   1.303967
 H      1.268300   2.627589   2.260223
 C      0.867473  -2.519297  -0.906588
 H      1.457376  -2.120827  -1.755471
 C     -1.489823  -2.359686  -0.102941
 H     -1.752817  -3.438520  -0.097643
 H     -1.016096  -2.142366   0.880677
 C     -2.727868  -1.519719  -0.232883
 C     -3.676177   0.487141  -0.894923
 C     -4.937636   0.134437  -0.381621
 C     -5.086425  -1.118332   0.227893
 C     -3.949866  -1.947184   0.295723
 C      0.931998  -4.053767  -0.973547
 H      1.986554  -4.393648  -0.971048
 H      0.418493  -4.539631  -0.119078
 H      0.471635  -4.418902  -1.914705
 C     -1.042254   1.677993  -2.371296
 C      0.637208  -0.009967  -2.821173
 H     -0.838337  -2.431493  -2.042893
 H     -1.476189  -0.562479  -3.036603
 F     -3.576133   1.681001  -1.476170
 F     -5.968035   0.977649  -0.475777
 F     -6.256937  -1.506416   0.728398
 F     -4.051845  -3.146715   0.887840
---------------------------------------
Mn17/vi_R
Frequencies, energies and thermodynamic properties:
 Lowest Vibrational Mode (1/cm) =                         14.3628
 2nd Lowest Vibrational Mode (1/cm) =                     19.3692
 E(RB-P86) (a.u.) =                                     -5071.93076459
 Thermal correction to Enthalpy (a.u.) =                  0.687380
 Thermal correction to Gibbs Free Energy (a.u.) =         0.552628
 Total Entropy (cal/Kmol) =                               283.608
 E(RPBE1PBE) (a.u.) =                                   -5071.36744729
Optimised cartesian coordinates (Angstrom):
 Fe-3.698290  -1.076660  -1.286324
 Mn0.702040  -0.277723   0.425477
 P     -1.268405   0.684968   0.659482
 O      1.375426  -0.315357   3.312511
 O     -0.240560  -3.000829   0.972390
 N      0.548373  -0.408769  -1.529516
 N      1.790874   1.432202  -0.125760
 C     -2.320404   0.364877  -0.811314
 C     -1.895634  -0.376950  -1.997030
 C     -2.931627  -0.219294  -2.990246
 H     -2.933784  -0.668077  -3.991658
 C     -3.984958   0.591647  -2.440810
 H     -4.919122   0.866548  -2.948872
 C     -3.619746   0.947702  -1.097555
 H     -4.216874   1.562266  -0.411753
```

```
 C     -3.540198  -2.732854  -0.068818
 H     -2.669046  -2.994947   0.544992
 C     -3.756123  -3.131553  -1.434220
 H     -3.082387  -3.752631  -2.039732
 C     -4.999082  -2.553100  -1.876939
 H     -5.435845  -2.651063  -2.879817
 C     -5.553507  -1.798479  -0.781824
 H     -6.487483  -1.221105  -0.804514
 C     -4.650992  -1.906392   0.335035
 H     -4.775129  -1.429889   1.316151
 C     -1.105238   2.536508   0.691071
 C     -0.610944   3.144266   1.870370
 H     -0.406052   2.530292   2.762147
 C     -0.381277   4.528926   1.915322
 H     -0.004650   4.989356   2.842515
 C     -0.629051   5.325284   0.780281
 H     -0.447382   6.411062   0.816238
 C     -1.109664   4.727866  -0.397466
 H     -1.308106   5.343530  -1.289205
 C     -1.347374   3.340677  -0.444223
 H     -1.737763   2.888124  -1.368610
 C     -2.370527   0.382751   2.124663
 C     -2.214599  -0.772995   2.919936
 H     -1.415292  -1.494154   2.700803
 C     -3.080589  -1.017103   4.001573
 H     -2.943581  -1.922875   4.613183
 C     -4.111041  -0.109690   4.303507
 H     -4.786947  -0.301116   5.151951
 C     -4.268520   1.049733   3.521840
 H     -5.067153   1.771496   3.755885
 C     -3.402244   1.297547   2.443289
 H     -3.526029   2.219768   1.854419
 C     -0.580367  -1.150247  -2.145441
 H     -0.670076  -2.082744  -1.553607
 C      0.908713   0.779619  -2.296834
 H      1.440203   0.538204  -3.247874
 H      0.004306   1.364506  -2.609132
 C      1.769662   1.678740  -1.471061
 C      2.506516   2.254879   0.656364
 C      3.254316   3.340396   0.173412
 C      3.254868   3.588869  -1.209234
 C      2.498456   2.736267  -2.030724
 C     -0.318600  -1.575989  -3.602762
 H     -1.109839  -2.272374  -3.946162
 H     -0.301728  -0.717327  -4.304795
 H      0.650875  -2.109184  -3.673815
 C      1.136516  -0.248650   2.158567
 C      0.087869  -1.888793   0.731662
 H      1.887753  -1.332530  -1.776319
 H      2.588976  -1.330947   0.156089
 C      3.342668  -1.807641  -0.575685
 C      3.695125  -3.171597   0.001867
 C      4.681961  -1.028666  -0.440342
 C      4.874534  -3.090535   0.774605
 C      3.017554  -4.389629  -0.157992
 C      5.407459  -1.667310   0.769055
 H      5.254423  -1.217602  -1.374943
 C      5.382802  -4.237569   1.407314
 C      3.529284  -5.540151   0.474264
 H      2.106677  -4.439921  -0.775632
 H      6.514352  -1.616384   0.700572
 C      4.702690  -5.463215   1.252660
 H      6.306439  -4.187196   2.007658
 H      3.014780  -6.507625   0.356003
 H      5.096387  -6.371257   1.737579
 O      2.789915  -1.823568  -1.855191
 H      4.539916   0.066936  -0.349469
 H      5.125660  -1.150416   1.715297
 F      2.523245   2.038794   1.968587
 F      3.946681   4.117140   1.007543
 F      3.949319   4.601441  -1.717200
 F      2.465089   2.954413  -3.352026
---------------------------------------
Mn17/vi_S
Frequencies, energies and thermodynamic properties:
 Lowest Vibrational Mode (1/cm) =                          15.8663
 2nd Lowest Vibrational Mode (1/cm) =                      20.8862
 E(RB-P86) (a.u.) =                                      -5071.93129226
 Thermal correction to Enthalpy (a.u.) =                   0.687327
 Thermal correction to Gibbs Free Energy (a.u.) =          0.553435
 Total Entropy (cal/Kmol) =                                281.801
 E(RPBE1PBE) (a.u.) =                                    -5071.36858684
Optimised cartesian coordinates (Angstrom):
 Fe-3.981966  -0.853686  -1.214512
 Mn0.460235  -0.562669   0.550745
```

P	-1.324671	0.735608	0.575682
O	1.046016	-0.326203	3.446772
O	-0.928806	-3.002680	1.395710
N	0.320054	-0.917599	-1.382180
N	1.839216	0.851221	-0.168917
C	-2.393454	0.398033	-0.878689
C	-2.078544	-0.554329	-1.942059
C	-3.059050	-0.365461	-2.985043
H	-3.119660	-0.937411	-3.919626
C	-3.973300	0.670039	-2.583911
H	-4.841869	1.021143	-3.157179
C	-3.575210	1.138641	-1.285216
H	-4.074106	1.926902	-0.706874
C	-4.118196	-2.328983	0.217803
H	-3.313394	-2.635607	0.897803
C	-4.364895	-2.874681	-1.090381
H	-3.785692	-3.670375	-1.577929
C	-5.489086	-2.176942	-1.660283
H	-5.913407	-2.343562	-2.659436
C	-5.940047	-1.201238	-0.700737
H	-6.769093	-0.494688	-0.841013
C	-5.091577	-1.292450	0.459106
H	-5.160310	-0.671641	1.361813
C	-0.851096	2.522215	0.376023
C	-0.257131	3.179920	1.480338
H	-0.152931	2.654400	2.443327
C	0.200008	4.502161	1.360105
H	0.652136	5.004087	2.230259
C	0.083454	5.183014	0.132528
H	0.443577	6.219718	0.038914
C	-0.494409	4.533223	-0.971371
H	-0.590895	5.058776	-1.934745
C	-0.960208	3.209448	-0.852736
H	-1.424249	2.718401	-1.721786
C	-2.483816	0.818400	2.025979
C	-2.543735	-0.234783	2.963838
H	-1.878292	-1.103442	2.865739
C	-3.455015	-0.185806	4.034591
H	-3.487270	-1.015181	4.758704
C	-4.315673	0.915893	4.183154
H	-5.027219	0.953799	5.023159
C	-4.256516	1.974882	3.258468
H	-4.920184	2.846831	3.371488
C	-3.344367	1.929744	2.190198
H	-3.296486	2.775680	1.486890
C	-0.905744	-1.541170	-1.940573
H	-1.157577	-2.361058	-1.238825
C	0.868868	0.103243	-2.270453
H	1.353013	-0.324743	-3.179950
H	0.070853	0.787707	-2.660732
C	1.864712	0.938241	-1.533433
C	2.686666	1.620354	0.530571
C	3.608606	2.502116	-0.055569
C	3.650405	2.590301	-1.456687
C	2.761903	1.788374	-2.192919
C	-0.692516	-2.191337	-3.321001
H	-1.581135	-2.790780	-3.603854
H	-0.522407	-1.446360	-4.125117
H	0.177442	-2.877914	-3.286308
C	0.852198	-0.374687	2.283401
C	-0.421577	-1.995515	1.031531
H	1.504453	-2.044519	-1.465996
H	2.170034	-1.826685	0.470407
C	2.778456	-2.616809	-0.116462
C	2.632414	-3.954461	0.663071
C	3.681219	-3.872529	1.799240
O	2.355244	-2.630619	-1.441323
F	2.666368	1.553562	1.858525
F	2.768687	1.856457	-3.530933
F	4.506512	3.407455	-2.060949
F	4.424867	3.237639	0.700227
C	4.243138	-2.267626	0.099177
C	5.052915	-1.390354	-0.637674
C	4.758060	-2.978577	1.206199
C	6.396102	-1.215608	-0.248446
H	4.643899	-0.866306	-1.516763
C	6.096067	-2.801036	1.595740
C	6.911634	-1.913600	0.862965
H	7.050677	-0.537325	-0.819432
H	6.511056	-3.355305	2.453965
H	7.965141	-1.773283	1.154561
H	2.900108	-4.767853	-0.046853
H	1.598282	-4.136917	1.015823
H	4.066014	-4.862213	2.122848
H	3.242395	-3.392729	2.704441

```
---------------------------------------
Mn17/viii
Frequencies, energies and thermodynamic properties:
 Lowest Vibrational Mode (1/cm) =                        19.0614
 2nd Lowest Vibrational Mode (1/cm) =                    25.9872
 E(RB-P86) (a.u.) =                                    -4649.18629251
 Thermal correction to Enthalpy (a.u.) =                 0.530044
 Thermal correction to Gibbs Free Energy (a.u.) =        0.417115
 Total Entropy (cal/Kmol) =                              237.678
 E(RPBE1PBE) (a.u.) =                                  -4648.62913428
Optimised cartesian coordinates (Angstrom):
 Fe3.318179  -1.142975   0.660963
 Mn-0.712418   0.053219  -1.828321
 P      0.297700   0.680134   0.097290
 O     -1.099272   2.868728  -2.675009
 O      1.717458   0.114633  -3.471424
 N     -0.474347  -1.902388  -1.260020
 N     -2.579010  -0.306218  -0.894647
 C      1.338674  -0.629052   0.842251
 C      1.464669  -1.982023   0.310590
 C      2.200730  -2.750408   1.286163
 H      2.486270  -3.805611   1.187172
 C      2.539471  -1.898279   2.395958
 H      3.117614  -2.191074   3.282957
 C      2.019865  -0.585783   2.124771
 H      2.112526   0.290043   2.779725
 C      4.185671  -0.307086  -1.012344
 H      3.645651   0.104269  -1.874575
 C      4.603444  -1.674984  -0.859269
 H      4.441268  -2.484499  -1.583599
 C      5.247596  -1.803935   0.423396
 H      5.659768  -2.728817   0.848800
 C      5.231187  -0.511781   1.061316
 H      5.630005  -0.279977   2.058095
 C      4.572055   0.413241   0.176200
 H      4.381701   1.475929   0.375114
 C     -0.946792   1.066141   1.426337
 C     -1.708445   2.253232   1.303591
 H     -1.512572   2.950438   0.473109
 C     -2.712583   2.556189   2.237749
 H     -3.291747   3.487402   2.132817
 C     -2.978860   1.673655   3.302352
 H     -3.766740   1.911571   4.034436
 C     -2.233462   0.488634   3.425883
 H     -2.433614  -0.206990   4.256417
 C     -1.222824   0.184388   2.493446
 H     -0.639672  -0.742232   2.608347
 C      1.360961   2.209143   0.155756
 C      1.971600   2.710716  -1.014011
 H      1.795175   2.225442  -1.983649
 C      2.815700   3.834838  -0.955329
 H      3.281542   4.212907  -1.879140
 C      3.060196   4.474724   0.272279
 H      3.719457   5.356091   0.316690
 C      2.450335   3.987534   1.442945
 H      2.628544   4.485879   2.409157
 C      1.603729   2.867086   1.384975
 H      1.119516   2.511404   2.307657
 C      0.860325  -2.452259  -1.020958
 H      1.507078  -2.052684  -1.829616
 C     -1.452662  -2.395661  -0.322526
 H     -1.767462  -3.454914  -0.514690
 H     -1.097051  -2.416365   0.751378
 C     -2.682649  -1.544232  -0.324854
 C     -3.648673   0.499338  -0.872554
 C     -4.882698   0.148694  -0.303151
 C     -5.006944  -1.123991   0.278146
 C     -3.886092  -1.969791   0.256624
 C      0.914414  -3.990935  -1.148211
 H      1.963304  -4.353025  -1.144887
 H      0.382567  -4.504593  -0.320276
 H      0.447279  -4.301086  -2.105204
 C     -1.002409   1.743042  -2.336127
 C      0.771367   0.091847  -2.762423
 H     -1.377762  -1.011381  -3.076837
 H     -1.676763  -0.288085  -3.312145
 F     -3.553861   1.706688  -1.427775
 F     -5.911732   0.999508  -0.317400
 F     -6.153931  -1.509247   0.831480
 F     -3.973470  -3.186424   0.814437
---------------------------------------
Mn17/ix
Frequencies, energies and thermodynamic properties:
 Lowest Vibrational Mode (1/cm) =                        20.4751
 2nd Lowest Vibrational Mode (1/cm) =                    23.5803
```

```
 E(RB-P86) (a.u.) =                                     -4802.97496804
 Thermal correction to Enthalpy (a.u.) =                 0.599486
 Thermal correction to Gibbs Free Energy (a.u.) =        0.477228
 Total Entropy (cal/Kmol) =                              257.314
 E(RPBE1PBE) (a.u.) =                                   -4802.42070194
Optimised cartesian coordinates (Angstrom):
 Fe-3.463473  -0.788310  -1.102679
 Mn0.757113  -0.593157   1.317031
 P     -0.513061   0.845130   0.118814
 O      1.161732   1.298159   3.537188
 O     -1.468389  -1.636134   2.911190
 N      0.558640  -2.046772  -0.235247
 N      2.508558  -0.188929   0.133882
 C     -1.562118  -0.014961  -1.130842
 C     -1.543866  -1.452588  -1.407452
 C     -2.352123  -1.673213  -2.584807
 H     -2.559373  -2.645444  -3.049198
 C     -2.877259  -0.411625  -3.030107
 H     -3.548850  -0.259618  -3.885660
 C     -2.402343   0.608480  -2.137639
 H     -2.631309   1.679349  -2.209210
 C     -4.158984  -1.056732   0.818887
 H     -3.536724  -1.098811   1.722067
 C     -4.516648  -2.179500  -0.007220
 H     -4.222453  -3.224364   0.160446
 C     -5.309776  -1.690829  -1.105993
 H     -5.719824  -2.295836  -1.925716
 C     -5.445040  -0.264280  -0.956722
 H     -5.976205   0.408117  -1.643671
 C     -4.731862   0.128724   0.230729
 H     -4.624796   1.152373   0.612388
 C      0.492085   2.019901  -0.925116
 C      1.201255   3.049375  -0.260050
 H      1.083840   3.182722   0.827586
 C      2.054670   3.906728  -0.973441
 H      2.592114   4.707763  -0.441120
 C      2.225937   3.742069  -2.361790
 H      2.897314   4.413137  -2.920591
 C      1.534461   2.716592  -3.028854
 H      1.659742   2.580284  -4.114996
 C      0.671836   1.860832  -2.316420
 H      0.126232   1.072758  -2.858106
 C     -1.682502   2.036656   0.947738
 C     -2.144514   1.789941   2.258826
 H     -1.797267   0.905416   2.810090
 C     -3.055860   2.667960   2.874041
 H     -3.404106   2.459521   3.898159
 C     -3.515899   3.806620   2.189637
 H     -4.227498   4.494654   2.673091
 C     -3.053894   4.067247   0.886285
 H     -3.400775   4.961417   0.344128
 C     -2.141107   3.192371   0.272148
 H     -1.773460   3.422613  -0.739949
 C     -0.799784  -2.527142  -0.621131
 H     -1.320143  -2.678175   0.345593
 C      1.460743  -1.819001  -1.374253
 H      1.802598  -2.762151  -1.851931
 H      0.908834  -1.253398  -2.159723
 C      2.648952  -1.001763  -0.949967
 C      3.531296   0.589260   0.485430
 C      4.762401   0.622930  -0.195047
 C      4.926202  -0.216417  -1.305878
 C      3.844403  -1.034850  -1.679927
 C     -0.768326  -3.883667  -1.344852
 H     -1.795509  -4.285884  -1.450942
 H     -0.327244  -3.814737  -2.360083
 H     -0.181869  -4.617703  -0.755544
 C      1.043752   0.547928   2.631453
 C     -0.595485  -1.196296   2.239307
 H      1.023126  -2.693161   0.452471
 F      3.399389   1.388470   1.542271
 F      5.748005   1.425927   0.209177
 F      6.067651  -0.232569  -1.986848
 F      3.971426  -1.836933  -2.746657
 O      1.953305  -2.106836   1.881816
 C      2.051896  -2.552001   3.201799
 H      1.064781  -2.897873   3.618037
 H      2.386602  -1.730837   3.893513
 C      3.051812  -3.711480   3.308306
 H      4.056572  -3.397140   2.952883
 H      3.149884  -4.064639   4.357618
 H      2.726486  -4.571573   2.684122
---------------------------------------
Mn17/x
Frequencies, energies and thermodynamic properties:
```

```
 Lowest Vibrational Mode (1/cm) =                        13.9720
 2nd Lowest Vibrational Mode (1/cm) =                    18.6719
 E(RB-P86) (a.u.) =                                     -4957.91583842
 Thermal correction to Enthalpy (a.u.) =                 0.683714
 Thermal correction to Gibbs Free Energy (a.u.) =        0.547975
 Total Entropy (cal/Kmol) =                              285.687
 E(RPBE1PBE) (a.u.) =                                   -4957.36957426
Optimised cartesian coordinates (Angstrom):
 Fe-3.602559  -1.037608  -1.078006
 Mn0.708609  -0.338977   1.145657
 P     -0.884315   0.937269   0.155167
 O      0.806919   1.530602   3.415540
 O     -1.131290  -1.950970   2.757559
 N      0.640204  -1.695715  -0.535788
 N      2.268133   0.448005  -0.092714
 C     -1.865203   0.050555  -1.121809
 C     -1.625323  -1.328249  -1.543541
 C     -2.460235  -1.575280  -2.697104
 H     -2.532971  -2.521496  -3.247618
 C     -3.216816  -0.387125  -2.983632
 H     -3.959225  -0.274725  -3.785028
 C     -2.862031   0.612740  -2.015413
 H     -3.269423   1.630499  -1.964755
 C     -4.105910  -1.607456   0.836607
 H     -3.424620  -1.627128   1.696952
 C     -4.327223  -2.690583  -0.084582
 H     -3.850186  -3.679108  -0.045732
 C     -5.266811  -2.244049  -1.080968
 H     -5.625556  -2.829432  -1.938114
 C     -5.629314  -0.884291  -0.772810
 H     -6.313360  -0.251943  -1.354471
 C     -4.910476  -0.489046   0.410328
 H     -4.951689   0.495281   0.894218
 C     -0.157580   2.367349  -0.796131
 C      0.413457   3.432113  -0.057782
 H      0.348092   3.435641   1.042130
 C      1.060512   4.491723  -0.714337
 H      1.491838   5.316055  -0.124274
 C      1.159824   4.500215  -2.119095
 H      1.669086   5.330517  -2.633426
 C      0.603423   3.443701  -2.860020
 H      0.672352   3.442173  -3.959607
 C     -0.052422   2.383846  -2.204142
 H     -0.497297   1.573765  -2.802509
 C     -2.172416   1.815551   1.176823
 C     -2.518005   1.342840   2.461459
 H     -2.006306   0.468535   2.886555
 C     -3.523411   1.979429   3.212151
 H     -3.778134   1.596806   4.213222
 C     -4.196258   3.098634   2.691362
 H     -4.981699   3.597573   3.280911
 C     -3.853928   3.582750   1.415393
 H     -4.369145   4.463686   1.000387
 C     -2.847126   2.949652   0.666169
 H     -2.578514   3.353606  -0.322282
 C     -0.677817  -2.325880  -0.894947
 H     -1.104961  -2.628415   0.081570
 C      1.359679  -1.156221  -1.706715
 H      1.782303  -1.951259  -2.356769
 H      0.643653  -0.583005  -2.338715
 C      2.448233  -0.219298  -1.266963
 C      3.182470   1.343070   0.279487
 C      4.344069   1.633580  -0.459513
 C      4.551563   0.943173  -1.661538
 C      3.579674   0.006677  -2.061631
 C     -0.492441  -3.605221  -1.728210
 H     -1.454813  -4.147075  -1.819333
 H     -0.124670  -3.396138  -2.753594
 H      0.229094  -4.275137  -1.219192
 C      0.808754   0.795557   2.490866
 C     -0.428644  -1.276879   2.083992
 H      2.245292  -2.893202   1.208516
 H      1.222755  -2.488318  -0.158820
 O      2.171522  -3.782331   0.644489
 C      3.449869  -4.073700   0.095103
 H      3.300665  -4.617828  -0.866873
 H      4.001523  -3.133882  -0.156674
 C      4.306942  -4.932264   1.029245
 H      3.782707  -5.878807   1.278942
 H      5.279787  -5.189602   0.557864
 H      4.518787  -4.399334   1.980644
 O      2.254545  -1.544478   1.757950
 C      2.682970  -1.615316   3.100477
 C      3.881071  -0.705520   3.398922
 H      2.976506  -2.672875   3.331857
```

```
 H      1.856742  -1.373432   3.815406
 H      4.213718  -0.817793   4.454084
 H      4.738817  -0.958594   2.739567
 H      3.624226   0.360020   3.230721
 F      3.001005   2.017846   1.413883
 F      5.221019   2.541165  -0.029186
 F      5.630012   1.172635  -2.403126
 F      3.749077  -0.655168  -3.214496
---------------------------------------
Mn17/TS-i
Frequencies, energies and thermodynamic properties:
 Lowest Vibrational Mode (1/cm) =-774.1747
 2nd Lowest Vibrational Mode (1/cm) =                      16.1418
 E(RB-P86) (a.u.) =                                      -4804.12686795
 Thermal correction to Enthalpy (a.u.) =                   0.611059
 Thermal correction to Gibbs Free Energy (a.u.) =          0.486347
 Total Entropy (cal/Kmol) =                                262.478
 E(RPBE1PBE) (a.u.) =                                    -4803.57110792
Optimised cartesian coordinates (Angstrom):
 Fe3.421991   0.935620  -1.081571
 Mn-0.726621   0.407019   1.436971
 P      0.606786  -0.901384   0.136110
 O     -0.836056  -1.658348   3.547110
 O      1.448723   1.599765   3.006446
 N     -0.678341   1.894611  -0.058326
 N     -2.483494  -0.052366   0.357976
 C      1.573248   0.045253  -1.098705
 C      1.451319   1.484156  -1.320892
 C      2.216217   1.794865  -2.505953
 H      2.348613   2.794131  -2.939555
 C      2.815076   0.586668  -3.006190
 H      3.475065   0.508163  -3.880558
 C      2.432022  -0.493229  -2.139458
 H      2.728778  -1.543968  -2.251688
 C      4.149119   1.199355   0.829407
 H      3.553555   1.175430   1.750726
 C      4.406563   2.366038   0.028152
 H      4.044226   3.382483   0.232591
 C      5.200552   1.962985  -1.104359
 H      5.545324   2.616879  -1.916577
 C      5.437088   0.545499  -1.000091
 H      5.994608  -0.069487  -1.719220
 C      4.784967   0.071977   0.192975
 H      4.761232  -0.966012   0.549138
 C     -0.383730  -2.096944  -0.890936
 C     -1.057853  -3.143477  -0.215869
 H     -0.937939  -3.264168   0.872945
 C     -1.876981  -4.038256  -0.923510
 H     -2.388181  -4.851749  -0.384608
 C     -2.046063  -3.895027  -2.314268
 H     -2.689779  -4.596155  -2.868562
 C     -1.388689  -2.853225  -2.990279
 H     -1.513184  -2.734060  -4.078358
 C     -0.561394  -1.958226  -2.284352
 H     -0.042321  -1.156246  -2.831305
 C      1.849037  -2.049123   0.915234
 C      2.367996  -1.787086   2.201776
 H      2.016083  -0.919570   2.776743
 C      3.344366  -2.629567   2.764032
 H      3.736386  -2.410582   3.769834
 C      3.813930  -3.746004   2.050143
 H      4.576795  -4.406114   2.492448
 C      3.297076  -4.020274   0.770666
 H      3.652173  -4.896862   0.205785
 C      2.318777  -3.181718   0.209052
 H      1.911102  -3.421427  -0.785284
 C      0.633629   2.457727  -0.474216
 H      1.185010   2.629361   0.472395
 C     -1.518828   1.555878  -1.209301
 H     -1.924146   2.452311  -1.733305
 H     -0.942825   0.996558  -1.990745
 C     -2.655072   0.676863  -0.784965
 C     -3.459805  -0.885809   0.735328
 C     -4.662797  -1.056166   0.028550
 C     -4.855584  -0.307487  -1.141729
 C     -3.829658   0.567780  -1.541206
 C      0.485676   3.835085  -1.151540
 H      1.476381   4.315873  -1.281414
 H      0.010752   3.769555  -2.152255
 H     -0.131238   4.494667  -0.508707
 C     -0.840983  -0.847499   2.692219
 C      0.610673   1.102697   2.337948
 H     -1.865373   1.170692   2.449029
 H     -1.675948   1.937178   2.088774
 H     -1.284202   2.817350   0.675784
```

```
 O     -1.770176   3.499784   1.531202
 C     -3.123220   3.811483   1.283418
 H     -3.274333   4.156957   0.227059
 H     -3.784605   2.909374   1.401361
 C     -3.615953   4.904237   2.234899
 H     -3.004690   5.824450   2.119366
 H     -4.677920   5.164818   2.037890
 H     -3.531980   4.573237   3.291941
 F     -3.985602   1.288090  -2.660196
 F     -5.975045  -0.423869  -1.847898
 F     -5.595509  -1.904634   0.462475
 F     -3.302380  -1.596895   1.849148
--------------------------------------
Mn17/TS-ii_si
Frequencies, energies and thermodynamic properties:
 Lowest Vibrational Mode (1/cm) =-256.7921
 2nd Lowest Vibrational Mode (1/cm) =                      11.9859
 E(RB-P86) (a.u.) =                                      -5071.92574284
 Thermal correction to Enthalpy (a.u.) =                   0.684094
 Thermal correction to Gibbs Free Energy (a.u.) =          0.552086
 Total Entropy (cal/Kmol) =                                277.834
 E(RPBE1PBE) (a.u.) =                                    -5071.35702761
Optimised cartesian coordinates (Angstrom):
 Fe3.789136   0.577919  -1.239181
 Mn-0.785511   0.472355   0.379539
 P      1.080993  -0.775484   0.674985
 O     -1.459063   0.727741   3.233820
 O      0.453293   3.106886   0.734241
 N     -0.508341   0.454293  -1.711069
 N     -1.994114  -1.145826  -0.151729
 C      2.231061  -0.665094  -0.756637
 C      1.950288   0.063607  -1.994626
 C      2.997642  -0.270118  -2.933293
 H      3.096279   0.122109  -3.953158
 C      3.919860  -1.171966  -2.299063
 H      4.833598  -1.580647  -2.750889
 C      3.458739  -1.412760  -0.960386
 H      3.947882  -2.057308  -0.218838
 C      3.773727   2.307756  -0.120960
 H      2.909069   2.705093   0.426225
 C      4.105590   2.597341  -1.491064
 H      3.543873   3.256882  -2.166141
 C      5.290233   1.851521  -1.830842
 H      5.785520   1.837656  -2.810943
 C      5.692810   1.102430  -0.668026
 H      6.549343   0.417755  -0.607060
 C      4.755049   1.381579   0.388091
 H      4.770368   0.950934   1.397623
 C      0.724453  -2.600502   0.794419
 C      0.056598  -3.061357   1.955446
 H     -0.160701  -2.360389   2.777853
 C     -0.330141  -4.406351   2.072637
 H     -0.840231  -4.749275   2.986987
 C     -0.071235  -5.311964   1.025367
 H     -0.376934  -6.366260   1.116462
 C      0.578554  -4.861264  -0.136136
 H      0.786575  -5.561526  -0.960934
 C      0.974983  -3.514508  -0.252426
 H      1.496818  -3.183200  -1.163525
 C      2.174500  -0.530260   2.162247
 C      2.151346   0.693385   2.865784
 H      1.467414   1.494973   2.553515
 C      3.002089   0.901612   3.966897
 H      2.970115   1.862445   4.504783
 C      3.884322  -0.111423   4.382303
 H      4.548014   0.051331   5.246332
 C      3.908078  -1.338212   3.693390
 H      4.589689  -2.141384   4.016103
 C      3.056910  -1.548128   2.594449
 H      3.073905  -2.520638   2.077975
 C      0.778122   0.999657  -2.265666
 H      0.939311   1.937028  -1.697950
 C     -0.890336  -0.837397  -2.315129
 H     -1.257924  -0.731550  -3.358076
 H      0.007250  -1.495554  -2.361800
 C     -1.928285  -1.512533  -1.469421
 C     -2.863490  -1.797589   0.633547
 C     -3.717187  -2.821992   0.187195
 C     -3.665596  -3.192995  -1.164059
 C     -2.752059  -2.515583  -1.992968
 C      0.648298   1.379746  -3.750199
 H      1.540298   1.947898  -4.081001
 H      0.548345   0.494091  -4.410337
 H     -0.236756   2.031456  -3.895624
 C     -1.208311   0.572030   2.088839
```

```
 C     -0.000684   2.022620   0.581453
 H     -1.272433   1.142032  -1.980915
 H     -2.112684   1.402746   0.033410
 C     -3.139480   2.175438  -1.028881
 C     -3.114643   3.465823  -0.252112
 C     -4.405842   1.437948  -0.526432
 C     -4.040512   3.402343   0.813526
 C     -2.373706   4.629579  -0.513490
 C     -4.701519   2.035669   0.865695
 H     -5.212907   1.710261  -1.245056
 C     -4.227228   4.520303   1.643026
 C     -2.565543   5.748345   0.316725
 H     -1.666868   4.656212  -1.358152
 H     -5.783019   2.089706   1.107493
 C     -3.484261   5.691206   1.387594
 H     -4.948262   4.490848   2.476541
 H     -2.002742   6.677232   0.130568
 H     -3.627591   6.576778   2.027709
 O     -2.642544   2.020438  -2.181912
 H     -4.307718   0.336635  -0.552292
 H     -4.224199   1.418746   1.660730
 F     -2.943677  -1.462640   1.919919
 F     -4.555226  -3.424549   1.031994
 F     -4.452113  -4.152557  -1.642034
 F     -2.667074  -2.855036  -3.286840
---------------------------------------
Mn17/TS-ii_re
Frequencies, energies and thermodynamic properties:
 Lowest Vibrational Mode (1/cm) =-244.3663
 2nd Lowest Vibrational Mode (1/cm) =                      5.6673
 E(RB-P86) (a.u.) =                                    -5071.92699549
 Thermal correction to Enthalpy (a.u.) =                   0.684156
 Thermal correction to Gibbs Free Energy (a.u.) =          0.550682
 Total Entropy (cal/Kmol) =                                280.921
 E(RPBE1PBE) (a.u.) =                                  -5071.36016150
Optimised cartesian coordinates (Angstrom):
 Fe-4.036265  -0.604866  -1.140769
 Mn0.513571  -0.714655   0.540587
 P     -1.202315   0.762410   0.571969
 O      1.120496  -0.558887   3.416531
 O     -0.991607  -3.115675   1.289792
 N      0.250161  -1.022128  -1.530930
 N      1.917014   0.626650  -0.229089
 C     -2.354491   0.530796  -0.843129
 C     -2.150127  -0.424140  -1.933304
 C     -3.152701  -0.147305  -2.936953
 H     -3.289613  -0.695550  -3.877349
 C     -3.974465   0.941866  -2.484557
 H     -4.837665   1.361368  -3.018404
 C     -3.494055   1.357853  -1.196576
 H     -3.912871   2.167231  -0.584930
 C     -4.210612  -2.117598   0.246900
 H     -3.394986  -2.512566   0.866319
 C     -4.575657  -2.591030  -1.062191
 H     -4.092201  -3.410691  -1.610600
 C     -5.670812  -1.785662  -1.538897
 H     -6.163634  -1.879250  -2.515867
 C     -5.985034  -0.815347  -0.521530
 H     -6.759967  -0.039949  -0.587620
 C     -5.081549  -1.017695   0.580930
 H     -5.046074  -0.427192   1.505514
 C     -0.641928   2.529341   0.381483
 C      0.090335   3.099976   1.451401
 H      0.243204   2.525694   2.379735
 C      0.623923   4.394512   1.343665
 H      1.182714   4.826027   2.189463
 C      0.449879   5.136417   0.159062
 H      0.870897   6.150705   0.073845
 C     -0.262873   4.573574  -0.913063
 H     -0.405019   5.145440  -1.844030
 C     -0.806561   3.278569  -0.804125
 H     -1.375087   2.860125  -1.648993
 C     -2.319557   0.900866   2.055135
 C     -2.435643  -0.178009   2.958135
 H     -1.845493  -1.092092   2.802243
 C     -3.306156  -0.097300   4.060502
 H     -3.383825  -0.948271   4.755739
 C     -4.068751   1.063993   4.277633
 H     -4.747789   1.127574   5.142721
 C     -3.952768   2.148433   3.388414
 H     -4.539868   3.065796   3.554461
 C     -3.081580   2.069807   2.287872
 H     -2.988044   2.934102   1.611726
 C     -1.085546  -1.512018  -2.016377
 H     -1.350944  -2.317478  -1.303685
```

```
 C      0.775108   0.103179  -2.329346
 H      1.119455  -0.209749  -3.338046
 H     -0.039866   0.845607  -2.488721
 C      1.892908   0.775349  -1.589549
 C      2.870905   1.274070   0.452725
 C      3.850267   2.089443  -0.141850
 C      3.834653   2.245066  -1.534397
 C      2.835030   1.566701  -2.256236
 C     -0.990910  -2.149916  -3.412668
 H     -1.937029  -2.669983  -3.662413
 H     -0.794753  -1.404759  -4.210408
 H     -0.179623  -2.905586  -3.426937
 C      0.905494  -0.568409   2.253768
 C     -0.433978  -2.115623   0.976565
 H      0.935935  -1.821646  -1.664465
 H      1.744398  -1.803959   0.385682
 C      2.667085  -2.880691  -0.494977
 C      3.965536  -2.297581  -0.003948
 C      2.372649  -4.061767   0.462932
 C      4.275820  -2.822486   1.270847
 C      4.837013  -1.419994  -0.669792
 C      3.172306  -3.751002   1.747465
 C      5.473255  -2.451579   1.904161
 C      6.034662  -1.048761  -0.031603
 H      4.584408  -1.053380  -1.678117
 C      6.346730  -1.560576   1.246913
 H      5.735150  -2.854263   2.896475
 H      6.739973  -0.366722  -0.532977
 H      7.291184  -1.267176   1.733000
 O      2.260844  -2.809278  -1.690128
 H      3.563797  -4.654728   2.258494
 H      2.531323  -3.217279   2.485574
 H      1.291767  -4.235138   0.618794
 H      2.788122  -4.963857  -0.042806
 F      2.912515   1.148939   1.776868
 F      4.772788   2.697631   0.605566
 F      4.738321   3.001927  -2.150377
 F      2.785869   1.701002  -3.589355
---------------------------------------
Mn17/TS-iii
Frequencies, energies and thermodynamic properties:
 Lowest Vibrational Mode (1/cm) =-670.0006
 2nd Lowest Vibrational Mode (1/cm) =                      18.7098
 E(RB-P86) (a.u.) =                                       -4649.18256911
 Thermal correction to Enthalpy (a.u.) =                   0.529041
 Thermal correction to Gibbs Free Energy (a.u.) =          0.416969
 Total Entropy (cal/Kmol) =                                235.876
 E(RPBE1PBE) (a.u.) =                                     -4648.62220684
Optimised cartesian coordinates (Angstrom):
 Fe3.306107  -1.130437   0.662336
 Mn-0.696218   0.001346  -1.818493
 P      0.291710   0.713236   0.105739
 O     -1.171307   2.679586  -2.981104
 O      1.742815   0.069474  -3.445842
 N     -0.498189  -1.992998  -1.217055
 N     -2.565114  -0.320641  -0.876729
 C      1.329021  -0.608721   0.844330
 C      1.453633  -1.974375   0.331265
 C      2.192229  -2.726169   1.319351
 H      2.477119  -3.782786   1.237154
 C      2.535669  -1.858230   2.413681
 H      3.118599  -2.137834   3.301646
 C      2.014809  -0.551378   2.124004
 H      2.111817   0.334809   2.764216
 C      4.175590  -0.304563  -1.015132
 H      3.635946   0.106157  -1.877607
 C      4.586493  -1.674285  -0.857878
 H      4.419497  -2.485360  -1.579357
 C      5.232062  -1.801982   0.423956
 H      5.639954  -2.727428   0.852230
 C      5.222838  -0.507630   1.057512
 H      5.623543  -0.274693   2.053253
 C      4.567197   0.417687   0.170135
 H      4.381766   1.481829   0.366001
 C     -0.934225   1.114768   1.448108
 C     -1.704683   2.294474   1.308259
 H     -1.520338   2.975311   0.461467
 C     -2.703281   2.610847   2.243986
 H     -3.288710   3.536457   2.124687
 C     -2.956795   1.748409   3.327991
 H     -3.740799   1.996169   4.060973
 C     -2.203951   0.569972   3.468487
 H     -2.394195  -0.110725   4.313655
 C     -1.198764   0.253035   2.534371
 H     -0.610966  -0.668955   2.663198
```

```
 C      1.364965   2.234405   0.144220
 C      1.946767   2.729301  -1.043019
 H      1.746887   2.236116  -2.004355
 C      2.790567   3.854887  -1.011346
 H      3.234497   4.227783  -1.947989
 C      3.061718   4.503318   0.206202
 H      3.720369   5.385968   0.229723
 C      2.478795   4.023830   1.393868
 H      2.677610   4.529889   2.352048
 C      1.633249   2.901543   1.363001
 H      1.169067   2.552102   2.298394
 C      0.852588  -2.508593  -0.978009
 H      1.475330  -2.126131  -1.812999
 C     -1.470938  -2.414044  -0.232791
 H     -1.787039  -3.481545  -0.344643
 H     -1.083687  -2.345470   0.823737
 C     -2.693270  -1.546858  -0.285952
 C     -3.623338   0.498214  -0.880250
 C     -4.872054   0.170699  -0.325276
 C     -5.022138  -1.090184   0.271154
 C     -3.909748  -1.949530   0.282129
 C      0.923893  -4.049103  -1.049125
 H      1.976302  -4.399784  -1.039057
 H      0.400770  -4.536824  -0.200393
 H      0.455896  -4.398220  -1.991904
 C     -1.030926   1.616558  -2.491731
 C      0.795256   0.036716  -2.738165
 H     -1.093714  -1.444300  -2.574579
 H     -1.436657  -0.783805  -3.134531
 F     -3.506374   1.697971  -1.445145
 F     -5.886610   1.036645  -0.365483
 F     -6.181362  -1.454793   0.810784
 F     -4.021891  -3.154787   0.858576
---------------------------------------
Mn18/i
Frequencies, energies and thermodynamic properties:
 Lowest Vibrational Mode (1/cm) =                        18.5299
 2nd Lowest Vibrational Mode (1/cm) =                    23.7318
 E(RB-P86) (a.u.) =                                    -4404.93300793
 Thermal correction to Enthalpy (a.u.) =                 0.590492
 Thermal correction to Gibbs Free Energy (a.u.) =        0.480738
 Total Entropy (cal/Kmol) =                              230.997
 E(RPBE1PBE) (a.u.) =                                  -4404.24095739
Optimised cartesian coordinates (Angstrom):
 Fe3.429896  -1.058091   0.705281
 Mn-0.631536  -0.236395  -1.639838
 P      0.387315   0.723971   0.042697
 O     -1.517055   2.219052  -3.053498
 O      1.664397  -0.293045  -3.473899
 N     -0.402222  -1.991050  -0.958110
 N     -2.525030  -0.522474  -0.870143
 C      1.463270  -0.501776   0.899607
 C      1.560648  -1.914785   0.541576
 C      2.333514  -2.564996   1.572589
 H      2.609308  -3.627122   1.594472
 C      2.719268  -1.584416   2.552813
 H      3.331723  -1.770338   3.445599
 C      2.193608  -0.311851   2.140146
 H      2.317034   0.636917   2.677998
 C      4.227717  -0.493402  -1.109788
 H      3.652347  -0.237935  -2.009263
 C      4.681850  -1.809906  -0.747648
 H      4.518603  -2.730518  -1.324014
 C      5.367339  -1.714541   0.516247
 H      5.813183  -2.549201   1.073750
 C      5.339562  -0.335840   0.934070
 H      5.761418   0.064099   1.865950
 C      4.633361   0.418757  -0.068638
 H      4.422652   1.495709  -0.038984
 C     -0.810997   1.237454   1.368520
 C     -1.579956   2.408085   1.162278
 H     -1.401431   3.032347   0.271443
 C     -2.566982   2.786581   2.086742
 H     -3.149561   3.706258   1.917295
 C     -2.813421   1.993195   3.224260
 H     -3.588559   2.289806   3.948561
 C     -2.066332   0.820396   3.428250
 H     -2.252911   0.193119   4.314662
 C     -1.070841   0.442510   2.506689
 H     -0.484267  -0.471610   2.685840
 C      1.430872   2.253286  -0.128672
 C      1.928468   2.635959  -1.392878
 H      1.675539   2.045624  -2.285057
 C      2.751861   3.769488  -1.523231
 H      3.131149   4.055191  -2.517250
```

```
 C      3.084919   4.536764  -0.392972
 H      3.727719   5.425398  -0.496203
 C      2.584615   4.169201   0.870050
 H      2.832233   4.769869   1.759800
 C      1.760006   3.038525   1.001432
 H      1.359601   2.775603   1.993092
 C      0.934929  -2.558798  -0.706353
 H      1.555019  -2.256592  -1.573473
 C     -1.431183  -2.534848  -0.088477
 H     -1.656548  -3.605492  -0.311926
 H     -1.112836  -2.528940   0.988950
 C     -2.690171  -1.729460  -0.213349
 C     -3.592579   0.284604  -0.994654
 C     -5.064434  -1.306105   0.162098
 C     -3.917765  -2.138411   0.290404
 H     -3.999279  -3.113268   0.796434
 C      0.973719  -4.097401  -0.648658
 H      2.023461  -4.454302  -0.653145
 H      0.485120  -4.507275   0.258833
 H      0.470261  -4.522837  -1.540820
 C     -1.154902   1.241879  -2.488560
 C      0.780395  -0.263901  -2.682572
 C     -4.884998  -0.039898  -0.508020
 C     -6.002953   0.838851  -0.666368
 C     -6.359513  -1.652393   0.653175
 C     -7.249197   0.473523  -0.176789
 H     -5.859031   1.802972  -1.179707
 C     -7.427242  -0.778285   0.486003
 H     -6.498446  -2.618254   1.164488
 H     -8.109455   1.150092  -0.298450
 H     -8.423822  -1.050765   0.867748
 H     -3.435530   1.240475  -1.517444
---------------------------------------
Mn18/ii
Frequencies, energies and thermodynamic properties:
 Lowest Vibrational Mode (1/cm) =                         16.2559
 2nd Lowest Vibrational Mode (1/cm) =                     19.4818
 E(RB-P86) (a.u.) =                                     -4559.86779571
 Thermal correction to Enthalpy (a.u.) =                  0.675210
 Thermal correction to Gibbs Free Energy (a.u.) =         0.549827
 Total Entropy (cal/Kmol) =                               263.891
 E(RPBE1PBE) (a.u.) =                                   -4559.18489046
Optimised cartesian coordinates (Angstrom):
 Fe3.532590   0.682969  -1.147632
 Mn-0.609826   0.563970   1.199457
 P      0.649009  -0.935784   0.216425
 O     -1.328387  -1.045527   3.583369
 O      1.517069   1.761114   2.837178
 N     -0.505403   1.825486  -0.248520
 N     -2.436817   0.189376   0.316590
 C      1.653848  -0.138685  -1.103309
 C      1.583446   1.275588  -1.466135
 C      2.372051   1.448518  -2.662464
 H      2.543321   2.398299  -3.185024
 C      2.931162   0.177677  -3.039925
 H      3.594430  -0.007335  -3.895734
 C      2.499428  -0.801487  -2.080567
 H      2.758241  -1.868004  -2.092505
 C      4.233265   1.098272   0.745821
 H      3.617853   1.186589   1.650338
 C      4.558002   2.170496  -0.156979
 H      4.237910   3.216542  -0.058423
 C      5.355268   1.627413  -1.227204
 H      5.745735   2.185457  -2.088789
 C      5.525957   0.217566  -0.983227
 H      6.069926  -0.486714  -1.626939
 C      4.830282  -0.110903   0.234225
 H      4.751336  -1.108529   0.685438
 C     -0.375275  -2.182250  -0.706536
 C     -1.033278  -3.190027   0.038898
 H     -0.870600  -3.263444   1.126670
 C     -1.889485  -4.103565  -0.596781
 H     -2.386508  -4.888645  -0.004842
 C     -2.113730  -4.015892  -1.984633
 H     -2.785934  -4.732445  -2.482687
 C     -1.476780  -3.008776  -2.729597
 H     -1.646919  -2.932585  -3.815456
 C     -0.612277  -2.095296  -2.096017
 H     -0.109595  -1.319694  -2.693992
 C      1.828974  -2.038774   1.137082
 C      2.250981  -1.706607   2.442304
 H      1.861749  -0.804984   2.935544
 C      3.173022  -2.521932   3.124206
 H      3.490812  -2.248725   4.142941
 C      3.682137  -3.680689   2.512184
```

```
 H      4.402479  -4.319364   3.047583
 C      3.258766  -4.026072   1.215216
 H      3.644368  -4.937508   0.731213
 C      2.335345  -3.214556   0.534049
 H      1.998043  -3.510236  -0.471696
 C      0.798760   2.353931  -0.707086
 H      1.363952   2.580346   0.218946
 C     -1.469308   1.674682  -1.333184
 H     -1.816766   2.654301  -1.739267
 H     -1.015042   1.142939  -2.210922
 C     -2.652273   0.881682  -0.862300
 C     -3.428923  -0.577408   0.797696
 C     -4.928812   0.022130  -1.052155
 C     -3.860897   0.823178  -1.543650
 C      0.693673   3.674443  -1.493283
 H      1.697831   4.125345  -1.626479
 H      0.248625   3.542419  -2.500737
 H      0.069805   4.387084  -0.916540
 C     -1.035886  -0.405884   2.630674
 C      0.708090   1.255961   2.133461
 H     -1.153814   3.243945   0.811758
 O     -1.412589   4.087661   1.283683
 C     -2.812745   4.278014   1.127674
 H     -3.387301   3.377423   1.461227
 H     -3.087407   4.444267   0.054362
 H     -3.988328   1.399699  -2.473200
 C     -3.251118   5.485987   1.948309
 H     -4.341009   5.664992   1.841299
 H     -3.027724   5.330653   3.024822
 H     -2.715255   6.400660   1.617578
 C     -4.694788  -0.708482   0.170615
 C     -5.732904  -1.529035   0.713727
 C     -6.956061  -1.621112   0.064577
 H     -5.546583  -2.082057   1.648134
 C     -6.198691  -0.093226  -1.694567
 C     -7.188434  -0.899354  -1.144932
 H     -7.755642  -2.253035   0.481415
 H     -6.380340   0.463259  -2.627687
 H     -8.165719  -0.985126  -1.645566
 H     -3.230724  -1.118662   1.735385
---------------------------------------
Mn18/iii
Frequencies, energies and thermodynamic properties:
 Lowest Vibrational Mode (1/cm) =                          20.5041
 2nd Lowest Vibrational Mode (1/cm) =                      24.1272
 E(RB-P86) (a.u.) =                                     -4561.04091250
 Thermal correction to Enthalpy (a.u.) =                   0.691334
 Thermal correction to Gibbs Free Energy (a.u.) =          0.567490
 Total Entropy (cal/Kmol) =                                260.653
 E(RPBE1PBE) (a.u.) =                                   -4560.35482993
Optimised cartesian coordinates (Angstrom):
 Fe3.540472   0.713950  -1.103993
 Mn-0.640539   0.548466   1.401584
 P      0.613350  -0.891604   0.166178
 O     -1.043860  -1.518844   3.482020
 O      1.605487   1.672558   2.939557
 N     -0.509220   1.953936  -0.139770
 N     -2.396244   0.134634   0.392256
 C      1.639737  -0.061656  -1.100140
 C      1.602271   1.370207  -1.382213
 C      2.393444   1.583616  -2.571423
 H      2.587581   2.554137  -3.045314
 C      2.924246   0.322573  -3.016067
 H      3.583931   0.168057  -3.880624
 C      2.472059  -0.695288  -2.108306
 H      2.706424  -1.765470  -2.173934
 C      4.271591   1.010797   0.801399
 H      3.670823   1.059931   1.718222
 C      4.603406   2.126346  -0.043923
 H      4.302875   3.170333   0.117357
 C      5.377295   1.630383  -1.153491
 H      5.766450   2.228967  -1.988053
 C      5.526641   0.206271  -0.990563
 H      6.050299  -0.469808  -1.679758
 C      4.840638  -0.178100   0.215781
 H      4.750965  -1.197528   0.613003
 C     -0.420937  -2.092856  -0.809724
 C     -1.123523  -3.094044  -0.096807
 H     -1.005319  -3.177912   0.995759
 C     -1.964752  -3.994244  -0.771029
 H     -2.498079  -4.772481  -0.202251
 C     -2.126231  -3.902384  -2.166753
 H     -2.786186  -4.608369  -2.695307
 C     -1.440601  -2.905102  -2.880955
 H     -1.558941  -2.826126  -3.973429
```

```
 C     -0.592847  -2.004318  -2.207850
 H     -0.053152  -1.236668  -2.783253
 C      1.786067  -2.064711   1.017342
 C      2.280170  -1.788450   2.310298
 H      1.948716  -0.891039   2.850264
 C      3.204009  -2.654123   2.924209
 H      3.576401  -2.422513   3.934681
 C      3.645984  -3.808987   2.255627
 H      4.367823  -4.487098   2.737820
 C      3.153180  -4.098258   0.969805
 H      3.486051  -5.004926   0.439900
 C      2.226824  -3.236597   0.357620
 H      1.835893  -3.489358  -0.640203
 C      0.823159   2.419590  -0.583444
 H      1.392596   2.616953   0.348379
 C     -1.365894   1.583476  -1.264746
 H     -1.693755   2.463829  -1.868557
 H     -0.841199   0.912927  -1.999332
 C     -2.577744   0.835526  -0.787082
 C     -3.397923  -0.623484   0.854599
 C     -4.866535   0.004752  -1.008184
 C     -3.781719   0.796608  -1.479475
 C      0.752531   3.764919  -1.339930
 H      1.767187   4.186911  -1.491835
 H      0.278679   3.665688  -2.338680
 H      0.164771   4.488450  -0.740078
 C     -0.896282  -0.685817   2.658472
 C      0.746676   1.196019   2.283890
 H     -1.742787   1.369712   2.446731
 H     -1.475652   2.006941   1.983867
 H     -1.156542   3.232306   0.601448
 O     -1.528883   4.011041   1.198037
 C     -2.857293   4.310067   0.811554
 H     -2.911140   4.627391  -0.262540
 H     -3.525654   3.413606   0.898705
 H     -3.892039   1.382200  -2.405341
 C     -3.413099   5.430612   1.687443
 H     -2.786702   6.343316   1.597177
 H     -4.451675   5.691982   1.394724
 H     -3.419832   5.128694   2.756297
 C     -4.658203  -0.741248   0.208071
 C     -5.709934  -1.555595   0.729981
 C     -6.927062  -1.626797   0.065192
 H     -5.541144  -2.120560   1.660602
 C     -6.130115  -0.087760  -1.666615
 C     -7.135753  -0.888777  -1.137859
 H     -7.738818  -2.254043   0.465115
 H     -6.294240   0.480986  -2.595561
 H     -8.107856  -0.957198  -1.651203
 H     -3.220388  -1.169520   1.794199
--------------------------------------
Mn18/iv
Frequencies, energies and thermodynamic properties:
 Lowest Vibrational Mode (1/cm) =                         17.9850
 2nd Lowest Vibrational Mode (1/cm) =                     21.3116
 E(RB-P86) (a.u.) =                                      -4561.07044148
 Thermal correction to Enthalpy (a.u.) =                  0.695919
 Thermal correction to Gibbs Free Energy (a.u.) =         0.571681
 Total Entropy (cal/Kmol) =                               261.482
 E(RPBE1PBE) (a.u.) =                                    -4560.38247784
Optimised cartesian coordinates (Angstrom):
 Fe3.573790   0.615862  -1.149545
 Mn-0.680860   0.676901   1.225079
 P      0.639692  -0.893867   0.233135
 O     -1.264486  -0.967621   3.591592
 O      1.380181   2.124717   2.747751
 N     -0.500555   1.922871  -0.518835
 N     -2.401956   0.165892   0.232404
 C      1.698657  -0.214235  -1.110258
 C      1.645433   1.169411  -1.584201
 C      2.476445   1.249167  -2.764478
 H      2.669273   2.152911  -3.355805
 C      3.049129  -0.044680  -3.017759
 H      3.746309  -0.291935  -3.829524
 C      2.580149  -0.945448  -2.002179
 H      2.840450  -2.008218  -1.916706
 C      4.194135   1.185911   0.729699
 H      3.538720   1.341500   1.596496
 C      4.552368   2.184629  -0.242748
 H      4.225875   3.233325  -0.241859
 C      5.396124   1.563046  -1.231260
 H      5.819625   2.052590  -2.118494
 C      5.562646   0.179218  -0.866866
 H      6.135980  -0.570720  -1.428208
 C      4.818188  -0.055412   0.343034
```

```
 H      4.724771  -1.013741   0.870029
 C     -0.314867  -2.244332  -0.626711
 C     -1.049606  -3.141475   0.186931
 H     -0.977685  -3.068582   1.284635
 C     -1.863384  -4.130803  -0.389016
 H     -2.419155  -4.826167   0.260340
 C     -1.971678  -4.232300  -1.789467
 H     -2.611051  -5.007166  -2.241386
 C     -1.260128  -3.336567  -2.605746
 H     -1.337798  -3.406653  -3.702670
 C     -0.437418  -2.348897  -2.029713
 H      0.124232  -1.666169  -2.686087
 C      1.818619  -1.912175   1.259380
 C      2.240666  -1.445869   2.523040
 H      1.853622  -0.493249   2.912134
 C      3.157994  -2.187138   3.290623
 H      3.476076  -1.807760   4.274835
 C      3.662719  -3.407543   2.807797
 H      4.378876  -3.988871   3.410143
 C      3.239938  -3.886749   1.553935
 H      3.622501  -4.846189   1.170343
 C      2.321624  -3.147480   0.788053
 H      1.985201  -3.545675  -0.182080
 C      0.875612   2.329466  -0.965251
 H      1.385885   2.623531  -0.026412
 C     -1.349816   1.383307  -1.601653
 H     -1.638568   2.154379  -2.348256
 H     -0.760303   0.614132  -2.149273
 C     -2.573915   0.723415  -1.025612
 C     -3.418691  -0.535904   0.753051
 C     -4.867919  -0.102075  -1.183604
 C     -3.766669   0.624353  -1.725300
 C      0.835707   3.569168  -1.875141
 H      1.859123   3.960286  -2.041377
 H      0.390472   3.354091  -2.867995
 H      0.246769   4.373718  -1.389721
 C     -1.021372  -0.312297   2.633556
 C      0.593358   1.511981   2.105659
 H     -1.624222   1.806088   1.856692
 H     -1.638416   3.237202   1.522598
 H     -0.951229   2.792940  -0.162122
 O     -1.674425   4.109302   1.019508
 C     -3.049455   4.491269   0.910707
 H     -3.092732   5.295388   0.144198
 H     -3.669618   3.646954   0.523829
 H     -3.857796   1.102390  -2.713093
 C     -3.622991   4.997828   2.233629
 H     -3.025470   5.849413   2.620646
 H     -4.671991   5.339130   2.104589
 H     -3.619222   4.196963   3.003345
 C     -4.673012  -0.714876   0.106143
 C     -5.736675  -1.462471   0.698436
 C     -6.123394  -0.253833  -1.841934
 C     -6.949124  -1.595385   0.032113
 H     -5.581944  -1.927321   1.685278
 C     -7.142683  -0.988190  -1.242934
 H     -6.272940   0.214817  -2.827691
 H     -7.768605  -2.171838   0.489098
 H     -8.110246  -1.103577  -1.756325
 H     -3.260067  -0.973588   1.750521
---------------------------------------
Mn18/v
Frequencies, energies and thermodynamic properties:
 Lowest Vibrational Mode (1/cm) =                         17.4123
 2nd Lowest Vibrational Mode (1/cm) =                     23.0311
 E(RB-P86) (a.u.) =                                     -4406.13221597
 Thermal correction to Enthalpy (a.u.) =                  0.611247
 Thermal correction to Gibbs Free Energy (a.u.) =         0.501006
 Total Entropy (cal/Kmol) =                               232.022
 E(RPBE1PBE) (a.u.) =                                   -4405.43967607
Optimised cartesian coordinates (Angstrom):
 Fe3.428169  -1.023974   0.722072
 Mn-0.698263  -0.211412  -1.734935
 P      0.359529   0.706509   0.059503
 O     -1.465959   2.347272  -2.960348
 O      1.552534  -0.440534  -3.615817
 N     -0.397081  -2.175448  -0.919245
 N     -2.507370  -0.503670  -0.825334
 C      1.460052  -0.483225   0.935712
 C      1.578914  -1.903625   0.600616
 C      2.370570  -2.524010   1.639648
 H      2.663593  -3.580374   1.682166
 C      2.752859  -1.519675   2.593861
 H      3.381062  -1.680693   3.480196
 C      2.202144  -0.265179   2.163800
```

```
 H      2.319284   0.697004   2.678605
 C      4.187333  -0.472753  -1.111784
 H      3.592915  -0.230239  -2.002610
 C      4.657443  -1.782875  -0.744987
 H      4.489943  -2.710631  -1.308598
 C      5.367371  -1.669269   0.503517
 H      5.828965  -2.495147   1.061193
 C      5.339188  -0.286562   0.906695
 H      5.776134   0.125976   1.825984
 C      4.608346   0.452869  -0.089125
 H      4.390392   1.528364  -0.066402
 C     -0.790309   1.279284   1.410673
 C     -1.615132   2.397008   1.132190
 H     -1.498376   2.938484   0.178884
 C     -2.576323   2.829758   2.060440
 H     -3.200777   3.707860   1.829828
 C     -2.744781   2.143127   3.278479
 H     -3.499892   2.481075   4.005866
 C     -1.944752   1.021681   3.556971
 H     -2.068713   0.476010   4.506295
 C     -0.974585   0.591757   2.630658
 H     -0.347377  -0.280145   2.873554
 C      1.423277   2.224231  -0.150339
 C      1.935857   2.549227  -1.424635
 H      1.684817   1.918005  -2.289378
 C      2.768851   3.670034  -1.597645
 H      3.160024   3.908843  -2.599563
 C      3.096440   4.484923  -0.499327
 H      3.746192   5.364164  -0.635231
 C      2.581502   4.175904   0.773535
 H      2.825188   4.813376   1.638608
 C      1.747756   3.057006   0.946021
 H      1.336659   2.839496   1.944420
 C      1.005646  -2.622611  -0.614814
 H      1.582801  -2.312403  -1.508521
 C     -1.362192  -2.412589   0.175709
 H     -1.561189  -3.491559   0.350834
 H     -0.908508  -2.013550   1.110287
 C     -2.645895  -1.672610  -0.090415
 C     -3.597822   0.259652  -0.994682
 C     -5.040361  -1.310351   0.229197
 C     -3.864429  -2.096522   0.414618
 H     -3.920647  -3.043977   0.973129
 C      1.108623  -4.152717  -0.500649
 H      2.170970  -4.465526  -0.465465
 H      0.610158  -4.545757   0.408666
 H      0.652338  -4.637234  -1.388488
 C     -1.145324   1.322267  -2.454451
 C      0.686006  -0.329509  -2.812626
 H     -0.703414  -2.712641  -1.741966
 H     -1.388533  -0.982489  -2.937361
 C     -4.888216  -0.075296  -0.497803
 C     -6.027967   0.759958  -0.709511
 C     -6.327735  -1.674109   0.720634
 C     -7.271193   0.380606  -0.216667
 H     -5.907124   1.702456  -1.267172
 C     -7.421602  -0.841445   0.500995
 H     -6.443201  -2.618508   1.276151
 H     -8.148612   1.025587  -0.380835
 H     -8.414103  -1.126559   0.884051
 H     -3.468525   1.185486  -1.575840
---------------------------------------
Mn18/vi_R
Frequencies, energies and thermodynamic properties:
 Lowest Vibrational Mode (1/cm) =                          14.5303
 2nd Lowest Vibrational Mode (1/cm) =                      19.9281
 E(RB-P86) (a.u.) =                                       -4828.84292179
 Thermal correction to Enthalpy (a.u.) =                   0.763808
 Thermal correction to Gibbs Free Energy (a.u.) =          0.631467
 Total Entropy (cal/Kmol) =                                278.535
 E(RPBE1PBE) (a.u.) =                                     -4828.14660486
Optimised cartesian coordinates (Angstrom):
 Fe3.897733   0.528344  -1.236465
 Mn-0.599657   0.390284   0.390544
 P      1.175790  -0.896730   0.597523
 O     -1.445727   0.195354   3.223137
 O      0.766612   2.899333   1.094663
 N     -0.396209   0.576253  -1.553344
 N     -1.956559  -1.037611  -0.215197
 C      2.296105  -0.689806  -0.843958
 C      2.018411   0.161552  -1.998751
 C      3.034014  -0.117806  -2.986196
 H      3.127058   0.368192  -3.965647
 C      3.932767  -1.111822  -2.463182
 H      4.819801  -1.511878  -2.972665
```

```
 C      3.489278  -1.462447  -1.142174
 H      3.966651  -2.194420  -0.478356
 C      3.986423   2.125685   0.064038
 H      3.158947   2.489489   0.686345
 C      4.278639   2.553325  -1.278238
 H      3.717855   3.302102  -1.853744
 C      5.420924   1.809540  -1.745060
 H      5.880018   1.887912  -2.739679
 C      5.836894   0.923306  -0.687745
 H      6.669425   0.208586  -0.735999
 C      4.948789   1.115626   0.429441
 H      4.985234   0.577089   1.385318
 C      0.728958  -2.700821   0.550275
 C      0.125123  -3.268024   1.698267
 H     -0.005766  -2.659551   2.608065
 C     -0.303948  -4.605057   1.691882
 H     -0.763327  -5.035066   2.596173
 C     -0.149122  -5.392658   0.534543
 H     -0.485985  -6.441387   0.529950
 C      0.436341  -4.833244  -0.613962
 H      0.561657  -5.442000  -1.523616
 C      0.873243  -3.494458  -0.608572
 H      1.343624  -3.074219  -1.510651
 C      2.286070  -0.840422   2.089183
 C      2.283591   0.277623   2.950502
 H      1.600464   1.117807   2.764400
 C      3.154639   0.331392   4.054315
 H      3.137994   1.210982   4.717185
 C      4.037219  -0.731775   4.313125
 H      4.717348  -0.689477   5.178598
 C      4.039997  -1.855443   3.465907
 H      4.720771  -2.698210   3.665677
 C      3.167801  -1.912415   2.365426
 H      3.167243  -2.808493   1.725410
 C      0.847777   1.143467  -2.127072
 H      1.080706   2.025831  -1.497926
 C     -0.932856  -0.526089  -2.353592
 H     -1.356747  -0.181598  -3.326086
 H     -0.129459  -1.259361  -2.625316
 C     -1.992830  -1.255187  -1.581478
 C     -2.838737  -1.687376   0.560402
 C     -3.874025  -2.797120  -1.369643
 C     -2.926447  -2.099466  -2.168794
 H     -2.926019  -2.231460  -3.262038
 C      0.684382   1.661237  -3.569545
 H      1.584165   2.231400  -3.877129
 H      0.538951   0.843387  -4.304660
 H     -0.183995   2.347931  -3.628912
 C     -1.109399   0.273013   2.090891
 C      0.271612   1.866149   0.794177
 H     -1.545646   1.715408  -1.774727
 H     -2.257744   1.784713   0.151217
 C     -2.905300   2.411433  -0.568886
 C     -3.028347   3.792621   0.060354
 C     -4.360321   1.868645  -0.477470
 C     -4.217056   3.886305   0.817500
 C     -2.149121   4.881264  -0.041487
 C     -4.985971   2.577085   0.748881
 H     -4.877193   2.187137  -1.409339
 C     -4.531418   5.078192   1.492049
 C     -2.465992   6.077163   0.633055
 H     -1.232518   4.797914  -0.647007
 H     -6.083990   2.720192   0.667732
 C     -3.648372   6.173566   1.395484
 H     -5.460115   5.164255   2.080502
 H     -1.790593   6.945136   0.560463
 H     -3.888521   7.116229   1.913607
 O     -2.334704   2.380811  -1.839073
 H     -4.406744   0.762234  -0.427319
 H     -4.813098   1.984487   1.676822
 C     -3.819925  -2.580274   0.056011
 C     -4.748231  -3.253656   0.910303
 C     -4.860119  -3.684958  -1.897055
 C     -5.694122  -4.111756   0.366934
 H     -4.700324  -3.081438   1.997290
 C     -5.748915  -4.326992  -1.043145
 H     -4.904340  -3.852468  -2.984775
 H     -6.409348  -4.631483   1.023202
 H     -6.506980  -5.011347  -1.455533
 H     -2.786547  -1.496829   1.643148
---------------------------------------
Mn18/vi_S
Frequencies, energies and thermodynamic properties:
 Lowest Vibrational Mode (1/cm) =                        12.4254
 2nd Lowest Vibrational Mode (1/cm) =                    17.9602
```

```
E(RB-P86) (a.u.) =                                          -4828.84320848
Thermal correction to Enthalpy (a.u.) =                      0.763804
Thermal correction to Gibbs Free Energy (a.u.) =             0.631426
Total Entropy (cal/Kmol) =                                   278.614
E(RPBE1PBE) (a.u.) =                                        -4828.14743144
```

Optimised cartesian coordinates (Angstrom):

```
Fe-4.083369  -0.706198  -1.185752
Mn0.379603  -0.561767   0.526382
P     -1.334267   0.822766   0.521467
O      1.157116  -0.056536   3.340191
O     -1.112225  -2.904656   1.493038
N      0.209693  -0.964037  -1.388428
N      1.831623   0.705727  -0.206058
C     -2.436758   0.486188  -0.909069
C     -2.175298  -0.512300  -1.943474
C     -3.160245  -0.317016  -2.980878
H     -3.256246  -0.916460  -3.895076
C     -4.025056   0.769280  -2.604965
H     -4.884875   1.138420  -3.180195
C     -3.590859   1.262866  -1.327153
H     -4.048732   2.090484  -0.770349
C     -4.263145  -2.133726   0.289763
H     -3.461279  -2.459559   0.964280
C     -4.559567  -2.702050  -0.998335
H     -4.027707  -3.537399  -1.473312
C     -5.659672  -1.966717  -1.568271
H     -6.110198  -2.140127  -2.554759
C     -6.045562  -0.944437  -0.628860
H     -6.842265  -0.202652  -0.774662
C     -5.181099  -1.045115   0.518424
H     -5.202528  -0.396907   1.404106
C     -0.795183   2.582568   0.257648
C     -0.160970   3.250707   1.332792
H     -0.059119   2.748283   2.308688
C      0.335846   4.553760   1.169371
H      0.818498   5.064257   2.017947
C      0.219630   5.204945  -0.074182
H      0.610292   6.226888  -0.202019
C     -0.396050   4.543787  -1.150446
H     -0.491511   5.045597  -2.126572
C     -0.901205   3.239240  -0.987779
H     -1.394451   2.739527  -1.835422
C     -2.473658   1.009325   1.980163
C     -2.557480   0.001996   2.965174
H     -1.924001  -0.893157   2.896081
C     -3.451988   0.130129   4.043701
H     -3.503293  -0.664831   4.804475
C     -4.271950   1.266884   4.153237
H     -4.970561   1.366981   4.999012
C     -4.188212   2.281282   3.181533
H     -4.819417   3.180439   3.263544
C     -3.292417   2.156675   2.105748
H     -3.223396   2.969013   1.365561
C     -1.044181  -1.547148  -1.923089
H     -1.326951  -2.337772  -1.199333
C      0.802917   0.023203  -2.292774
H      1.214778  -0.438630  -3.220633
H      0.037133   0.761954  -2.646363
C      1.892311   0.775606  -1.586769
C      2.742602   1.382055   0.510493
C      3.848739   2.234367  -1.508820
C      2.873589   1.505682  -2.245057
H      2.889026   1.524449  -3.345918
C     -0.869922  -2.241255  -3.288082
H     -1.783949  -2.812149  -3.548591
H     -0.676535  -1.524623  -4.112407
H     -0.027332  -2.960371  -3.240128
C      0.850580  -0.259283   2.215101
C     -0.569746  -1.934209   1.081872
H      1.338125  -2.145047  -1.462609
H      2.000227  -2.022409   0.481198
C      2.547524  -2.846728  -0.112163
C      2.282594  -4.190286   0.626338
C      3.336319  -4.245877   1.759547
O      2.139753  -2.798090  -1.442949
C      4.035309  -2.629359   0.121333
C      4.485816  -3.424448   1.198883
C      4.917533  -1.790049  -0.576007
C      5.832769  -3.375365   1.595495
C      6.269191  -1.744202  -0.179800
H      4.554654  -1.191235  -1.427208
C      6.722034  -2.529742   0.900134
H      6.196832  -3.996794   2.430625
H      6.979019  -1.096859  -0.719872
H      7.782675  -2.489996   1.197089
```

```
 H      3.634902  -5.277802   2.040034
 H      2.943158  -3.768964   2.687029
 H      2.472394  -5.000562  -0.111675
 H      1.236868  -4.288546   0.978545
 C      3.773385   2.166558  -0.069537
 C      4.727856   2.876431   0.723875
 C      4.881569   3.015068  -2.111014
 C      5.719151   3.628203   0.108552
 H      4.663429   2.817206   1.821967
 C      5.794646   3.696639  -1.315213
 H      4.942343   3.069230  -3.209488
 H      6.454546   4.176292   0.717758
 H      6.588540   4.298315  -1.785126
 H      2.671952   1.307005   1.606290
---------------------------------------
Mn18/viii
Frequencies, energies and thermodynamic properties:
 Lowest Vibrational Mode (1/cm) =                         19.9237
 2nd Lowest Vibrational Mode (1/cm) =                     25.4343
 E(RB-P86) (a.u.) =                                      -4406.09668693
 Thermal correction to Enthalpy (a.u.) =                  0.606623
 Thermal correction to Gibbs Free Energy (a.u.) =         0.496087
 Total Entropy (cal/Kmol) =                               232.642
 E(RPBE1PBE) (a.u.) =                                    -4405.40543197
Optimised cartesian coordinates (Angstrom):
 Fe3.433571  -1.006809   0.738948
 Mn-0.659570  -0.230735  -1.793676
 P      0.343427   0.644742   0.033479
 O     -1.349698   2.457909  -2.834644
 O      1.759274  -0.269648  -3.471651
 N     -0.370530  -2.109782  -1.011514
 N     -2.488412  -0.511674  -0.865953
 C      1.439222  -0.540263   0.900052
 C      1.601483  -1.940086   0.520266
 C      2.380494  -2.569063   1.560468
 H      2.700001  -3.619050   1.574377
 C      2.709110  -1.588104   2.561232
 H      3.312261  -1.760592   3.463069
 C      2.140215  -0.332022   2.155539
 H      2.214778   0.613953   2.707056
 C      4.250687  -0.331335  -1.030289
 H      3.686599  -0.035480  -1.923819
 C      4.718005  -1.659303  -0.734985
 H      4.577138  -2.548116  -1.364674
 C      5.381440  -1.624824   0.543909
 H      5.831211  -2.482798   1.061507
 C      5.326891  -0.271517   1.036880
 H      5.729203   0.081774   1.995917
 C      4.625143   0.527904   0.066067
 H      4.399006   1.598826   0.150564
 C     -0.873992   1.163416   1.343280
 C     -1.672704   2.304938   1.092133
 H     -1.525275   2.887106   0.167899
 C     -2.648153   2.712405   2.016849
 H     -3.255668   3.607871   1.809927
 C     -2.849123   1.979814   3.202496
 H     -3.613867   2.299702   3.927945
 C     -2.068883   0.838342   3.454139
 H     -2.218373   0.258927   4.379246
 C     -1.087357   0.430400   2.530235
 H     -0.476932  -0.460003   2.745413
 C      1.364401   2.199172  -0.111091
 C      1.908519   2.597428  -1.351025
 H      1.705930   2.011397  -2.257827
 C      2.718029   3.744751  -1.443475
 H      3.131975   4.040095  -2.420694
 C      2.993786   4.512065  -0.298438
 H      3.626231   5.411003  -0.371948
 C      2.448622   4.129478   0.941178
 H      2.650559   4.728336   1.843594
 C      1.636330   2.986235   1.033283
 H      1.200730   2.714003   2.007188
 C      0.982932  -2.587612  -0.728649
 H      1.605670  -2.281857  -1.595559
 C     -1.313939  -2.439965   0.036441
 H     -1.547901  -3.534038   0.090103
 H     -0.946919  -2.188265   1.076926
 C     -2.601484  -1.687048  -0.144330
 C     -3.572286   0.259485  -1.020179
 C     -4.985392  -1.300558   0.240766
 C     -3.816110  -2.096256   0.393584
 C      1.073297  -4.129324  -0.657416
 H      2.130083  -4.467566  -0.636967
 H      0.573781  -4.542581   0.243628
 H      0.588658  -4.568450  -1.553195
```

```
 C     -1.085866   1.375608  -2.437987
 C      0.825600  -0.243055  -2.746677
 H     -1.275608  -1.463660  -2.884498
 H     -1.573723  -0.784615  -3.233915
 H     -3.868273  -3.046357   0.948016
 C     -4.851053  -0.066381  -0.493772
 C     -5.991054   0.775246  -0.680558
 C     -6.265312  -1.654236   0.766845
 C     -7.221456   0.404608  -0.154913
 H     -5.878333   1.714948  -1.244522
 C     -7.356756  -0.816260   0.571835
 H     -6.372788  -2.596062   1.328035
 H     -8.100581   1.052093  -0.297436
 H     -8.340675  -1.094357   0.981556
 H     -3.447629   1.187680  -1.599358
--------------------------------------
Mn18/ix
Frequencies, energies and thermodynamic properties:
 Lowest Vibrational Mode (1/cm) =                         19.9805
 2nd Lowest Vibrational Mode (1/cm) =                     24.8844
 E(RB-P86) (a.u.) =                                     -4559.88529100
 Thermal correction to Enthalpy (a.u.) =                  0.675845
 Thermal correction to Gibbs Free Energy (a.u.) =         0.556720
 Total Entropy (cal/Kmol) =                               250.721
 E(RPBE1PBE) (a.u.) =                                   -4559.19790679
Optimised cartesian coordinates (Angstrom):
 Fe-3.588640  -0.420666  -1.181887
 Mn0.670757  -0.944928   1.111253
 P     -0.503096   0.763526   0.209161
 O      1.413570   0.622838   3.487162
 O     -1.598988  -2.087869   2.591271
 N      0.344582  -2.078045  -0.663922
 N      2.406224  -0.489836   0.055844
 C     -1.635402   0.211999  -1.139349
 C     -1.727748  -1.152086  -1.661215
 C     -2.575739  -1.108065  -2.830075
 H     -2.863358  -1.968038  -3.447802
 C     -3.016937   0.244819  -3.032821
 H     -3.693730   0.589848  -3.825959
 C     -2.449098   1.058138  -1.994080
 H     -2.600789   2.138023  -1.872078
 C     -4.263035  -0.965422   0.688360
 H     -3.629305  -1.202937   1.552353
 C     -4.716903  -1.904049  -0.303572
 H     -4.495066  -2.979589  -0.324211
 C     -5.494368  -1.182498  -1.278415
 H     -5.963385  -1.609642  -2.174943
 C     -5.523169   0.203669  -0.886319
 H     -6.018408   1.017739  -1.432448
 C     -4.759883   0.339156   0.327117
 H     -4.570912   1.273178   0.871927
 C      0.556048   2.042146  -0.640342
 C      1.337628   2.895701   0.175514
 H      1.250910   2.835628   1.272867
 C      2.218990   3.826420  -0.398347
 H      2.811905   4.489275   0.252068
 C      2.346547   3.911109  -1.798410
 H      3.038626   4.640246  -2.248735
 C      1.585852   3.058513  -2.616362
 H      1.677606   3.116798  -3.712831
 C      0.695796   2.129858  -2.042338
 H      0.095937   1.481411  -2.699499
 C     -1.570740   1.868443   1.267735
 C     -2.006402   1.438788   2.539776
 H     -1.695934   0.458553   2.926526
 C     -2.842690   2.253869   3.325005
 H     -3.171082   1.900858   4.315538
 C     -3.253116   3.512440   2.851903
 H     -3.906342   4.150901   3.467691
 C     -2.815408   3.955836   1.590070
 H     -3.122628   4.944562   1.213340
 C     -1.977176   3.143389   0.807186
 H     -1.626133   3.516083  -0.167637
 C     -1.043122  -2.394430  -1.100440
 H     -1.563815  -2.688930  -0.166767
 C      1.260816  -1.682662  -1.746772
 H      1.506628  -2.524328  -2.430377
 H      0.752057  -0.911325  -2.368954
 C      2.526491  -1.087429  -1.183210
 C      3.470589   0.129916   0.573116
 C      4.873178  -0.435667  -1.360454
 C      3.722728  -1.088800  -1.888188
 C     -1.106996  -3.594375  -2.061129
 H     -2.158425  -3.908843  -2.216052
 H     -0.673305  -3.365569  -3.055916
```

```
 H     -0.560531  -4.457785  -1.629694
 C      1.120612  -0.023209   2.536921
 C     -0.715364  -1.597640   1.972320
 H      0.770537  -2.865237  -0.107422
 H      3.778879  -1.592851  -2.865630
 O      1.705661  -2.679471   1.380408
 C      1.739040  -3.432929   2.555263
 C      2.729620  -2.913539   3.610498
 H      2.040203  -4.484160   2.296243
 H      0.729583  -3.532165   3.041169
 H      2.762646  -3.586171   4.496108
 H      3.755522  -2.852723   3.186652
 H      2.447128  -1.899201   3.960941
 C      4.733209   0.206723  -0.077792
 C      5.851912   0.879129   0.502894
 C      6.135577  -0.386348  -2.024679
 C      7.067443   0.912086  -0.168459
 H      5.735963   1.366315   1.484206
 C      7.207878   0.276010  -1.437329
 H      6.246662  -0.876742  -3.004769
 H      7.930525   1.430277   0.277685
 H      8.179442   0.311786  -1.954974
 H      3.346990   0.589503   1.566073
---------------------------------------
Mn18/x
Frequencies, energies and thermodynamic properties:
 Lowest Vibrational Mode (1/cm) =                        12.9560
 2nd Lowest Vibrational Mode (1/cm) =                    22.4578
 E(RB-P86) (a.u.) =                                    -4714.82702316
 Thermal correction to Enthalpy (a.u.) =                 0.759957
 Thermal correction to Gibbs Free Energy (a.u.) =        0.626846
 Total Entropy (cal/Kmol) =                              280.155
 E(RPBE1PBE) (a.u.) =                                  -4714.14794215
Optimised cartesian coordinates (Angstrom):
 Fe3.731502   0.688068  -1.162028
 Mn-0.622429   0.537331   1.071103
 P      0.853576  -0.946387   0.201225
 O     -1.028711  -1.236397   3.382269
 O      1.357490   2.116078   2.568379
 N     -0.444342   1.728508  -0.716022
 N     -2.225296  -0.189193  -0.028138
 C      1.908897  -0.253655  -1.136142
 C      1.785419   1.100925  -1.671393
 C      2.642972   1.183202  -2.831414
 H      2.795618   2.071968  -3.456332
 C      3.300809  -0.082085  -3.012103
 H      4.034858  -0.320212  -3.793502
 C      2.860212  -0.966717  -1.969741
 H      3.182445  -2.006641  -1.832093
 C      4.272083   1.372524   0.704453
 H      3.590699   1.521842   1.551885
 C      4.589232   2.351579  -0.301364
 H      4.197753   3.376575  -0.351107
 C      5.492102   1.744762  -1.245752
 H      5.903972   2.223443  -2.144286
 C      5.735987   0.390010  -0.820573
 H      6.366640  -0.344589  -1.339040
 C      4.980465   0.158167   0.382950
 H      4.934356  -0.782213   0.947223
 C      0.027358  -2.399105  -0.625856
 C     -0.629524  -3.343309   0.200122
 H     -0.578225  -3.243822   1.296605
 C     -1.341509  -4.415000  -0.362984
 H     -1.838707  -5.145085   0.295526
 C     -1.421320  -4.555551  -1.761895
 H     -1.980685  -5.395468  -2.203493
 C     -0.782427  -3.617301  -2.590200
 H     -0.836716  -3.718803  -3.685961
 C     -0.062141  -2.545889  -2.027145
 H      0.445928  -1.830752  -2.692324
 C      2.064711  -1.833480   1.308989
 C      2.418916  -1.298838   2.566395
 H      1.958950  -0.365823   2.919534
 C      3.365607  -1.947493   3.380702
 H      3.627642  -1.514847   4.359307
 C      3.970512  -3.141671   2.951391
 H      4.710210  -3.649905   3.590169
 C      3.617784  -3.688655   1.703819
 H      4.078803  -4.628658   1.360789
 C      2.669091  -3.043213   0.892023
 H      2.389601  -3.496711  -0.071626
 C      0.915448   2.221840  -1.121884
 H      1.366978   2.580000  -0.175263
 C     -1.205861   1.109898  -1.822547
 H     -1.533128   1.851420  -2.582886
```

```
 H     -0.538723   0.391536  -2.349793
 C     -2.392655   0.354260  -1.286747
 C     -3.201428  -0.949053   0.475300
 C     -4.611391  -0.639965  -1.511031
 C     -3.553264   0.164466  -2.024260
 C      0.826851   3.430424  -2.069732
 H      1.826646   3.889508  -2.204054
 H      0.443285   3.156055  -3.073760
 H      0.156678   4.195248  -1.628398
 C     -0.874877  -0.512015   2.458066
 C      0.602428   1.449113   1.948014
 H     -1.973373   3.200445   0.889009
 H     -0.972730   2.591468  -0.426590
 O     -1.867398   4.013382   0.218835
 C     -3.134720   4.289632  -0.360387
 H     -2.968433   4.727371  -1.372824
 H     -3.719069   3.348818  -0.517127
 H     -3.651249   0.632605  -3.016053
 C     -3.962249   5.268474   0.477820
 H     -3.405322   6.216841   0.632137
 H     -4.925634   5.510020  -0.020758
 H     -4.192597   4.842787   1.477707
 O     -2.030043   1.950146   1.617723
 C     -2.343024   2.234572   2.961820
 C     -3.549920   1.447108   3.489798
 H     -2.574190   3.328721   3.053914
 H     -1.471703   2.058472   3.643165
 H     -3.807894   1.765314   4.523911
 H     -4.438952   1.614890   2.845350
 H     -3.342645   0.357413   3.512721
 C     -4.417480  -1.227375  -0.209209
 C     -5.441274  -2.047105   0.356423
 C     -5.830744  -0.890144  -2.208586
 C     -6.617151  -2.274636  -0.347500
 H     -5.285263  -2.491371   1.352402
 C     -6.810914  -1.692858  -1.634885
 H     -5.982285  -0.441788  -3.203253
 H     -7.407166  -2.906355   0.087506
 H     -7.749706  -1.883639  -2.178405
 H     -3.039946  -1.369237   1.479750
---------------------------------------
Mn18/TS-i
Frequencies, energies and thermodynamic properties:
 Lowest Vibrational Mode (1/cm) =-732.7303
 2nd Lowest Vibrational Mode (1/cm) =                      19.0146
 E(RB-P86) (a.u.) =                                      -4561.03895122
 Thermal correction to Enthalpy (a.u.) =                   0.687582
 Thermal correction to Gibbs Free Energy (a.u.) =         0.565423
 Total Entropy (cal/Kmol) =                                257.106
 E(RPBE1PBE) (a.u.) =                                    -4560.35024642
Optimised cartesian coordinates (Angstrom):
 Fe3.543475   0.673517  -1.118623
 Mn-0.647150   0.588361   1.371671
 P      0.601393  -0.889157   0.175984
 O     -1.102039  -1.403518   3.506489
 O      1.592255   1.722977   2.912236
 N     -0.491526   1.971836  -0.204946
 N     -2.399367   0.176133   0.358110
 C      1.638403  -0.089934  -1.105604
 C      1.614331   1.336611  -1.419131
 C      2.410587   1.520245  -2.609885
 H      2.613426   2.479182  -3.103187
 C      2.932414   0.246140  -3.025713
 H      3.594100   0.068936  -3.884271
 C      2.468771  -0.748855  -2.098845
 H      2.695433  -1.821829  -2.141349
 C      4.266685   1.011577   0.782477
 H      3.661228   1.091317   1.694202
 C      4.616747   2.100755  -0.089701
 H      4.327660   3.152077   0.042274
 C      5.391725   1.567160  -1.180754
 H      5.792515   2.139110  -2.028359
 C      5.523497   0.146350  -0.979782
 H      6.042965  -0.553573  -1.647987
 C      4.825698  -0.198498   0.231607
 H      4.721486  -1.206228   0.654218
 C     -0.435203  -2.101092  -0.784129
 C     -1.148297  -3.083894  -0.055977
 H     -1.032645  -3.150467   1.038110
 C     -1.996490  -3.987691  -0.716483
 H     -2.537430  -4.751694  -0.135707
 C     -2.155589  -3.917387  -2.113761
 H     -2.821197  -4.626037  -2.631533
 C     -1.460295  -2.938032  -2.843211
 H     -1.576631  -2.875864  -3.936991
```

```
 C     -0.604931  -2.034212  -2.183830
 H     -0.056661  -1.282349  -2.771934
 C      1.763930  -2.052854   1.052602
 C      2.249254  -1.755688   2.344252
 H      1.918412  -0.846423   2.864493
 C      3.163588  -2.614957   2.981023
 H      3.529509  -2.367057   3.989998
 C      3.604359  -3.784105   2.336885
 H      4.318739  -4.457184   2.836919
 C      3.119954  -4.094182   1.052661
 H      3.451997  -5.012167   0.542067
 C      2.203135  -3.238875   0.417628
 H      1.818311  -3.507733  -0.578414
 C      0.855198   2.415418  -0.648309
 H      1.414345   2.616245   0.288344
 C     -1.358681   1.591295  -1.326273
 H     -1.671614   2.464522  -1.944264
 H     -0.825337   0.904895  -2.032256
 C     -2.577000   0.864370  -0.829512
 C     -3.408564  -0.567074   0.828503
 C     -4.874646   0.057620  -1.038101
 C     -3.781744   0.832801  -1.519600
 C      0.800982   3.749804  -1.420349
 H      1.821056   4.156969  -1.573487
 H      0.327415   3.645361  -2.418407
 H      0.223721   4.488981  -0.829346
 C     -0.929510  -0.602622   2.656114
 C      0.733936   1.242488   2.258030
 H     -1.727760   1.489056   2.338461
 H     -1.482857   2.198221   1.919229
 H     -1.034769   2.985040   0.454142
 O     -1.472179   3.775025   1.241853
 C     -2.797508   4.154256   0.955609
 H     -2.935111   4.376833  -0.136106
 H     -3.526257   3.327758   1.183525
 H     -3.887029   1.409416  -2.451626
 C     -3.201236   5.391370   1.762405
 H     -2.527558   6.244016   1.531302
 H     -4.244450   5.701141   1.537350
 H     -3.128391   5.190235   2.852597
 C     -4.671059  -0.678425   0.184733
 C     -5.730086  -1.476463   0.716625
 C     -6.949439  -1.541178   0.054943
 H     -5.565464  -2.034020   1.652407
 C     -6.140030  -0.027641  -1.693151
 C     -7.153134  -0.812931  -1.154570
 H     -7.766789  -2.155884   0.462871
 H     -6.300071   0.533713  -2.627261
 H     -8.126982  -0.875963  -1.665239
 H     -3.236352  -1.103359   1.774514
---------------------------------------
Mn18/TS-ii_si
Frequencies, energies and thermodynamic properties:
 Lowest Vibrational Mode (1/cm) =-258.2972
 2nd Lowest Vibrational Mode (1/cm) =                      17.8210
 E(RB-P86) (a.u.) =                                     -4828.83880081
 Thermal correction to Enthalpy (a.u.) =                 0.760598
 Thermal correction to Gibbs Free Energy (a.u.) =        0.631366
 Total Entropy (cal/Kmol) =                              271.991
 E(RPBE1PBE) (a.u.) =                                   -4828.13679652
Optimised cartesian coordinates (Angstrom):
 Fe3.896942   0.021576  -1.208685
 Mn-0.650124   0.568378   0.361806
 P      0.974003  -0.988349   0.597209
 O     -1.513993   0.577318   3.175055
 O      0.992640   2.946725   0.907047
 N     -0.351925   0.624617  -1.720334
 N     -2.113821  -0.732827  -0.235374
 C      2.152165  -0.985529  -0.816669
 C      2.013982  -0.152312  -2.012131
 C      3.014349  -0.593314  -2.957573
 H      3.192887  -0.164494  -3.951514
 C      3.771244  -1.663818  -2.368456
 H      4.618019  -2.185619  -2.834137
 C      3.251217  -1.904488  -1.051557
 H      3.619216  -2.659219  -0.344810
 C      4.133423   1.661267   0.015611
 H      3.333699   2.154398   0.583053
 C      4.525164   1.978686  -1.332340
 H      4.081782   2.757432  -1.967450
 C      5.584734   1.079781  -1.712800
 H      6.086193   1.049301  -2.689421
 C      5.850156   0.207830  -0.597001
 H      6.589662  -0.603856  -0.574952
 C      4.952292   0.564838   0.470420
```

```
 H      4.886233   0.076964   1.451463
 C      0.336916  -2.738521   0.616346
 C     -0.405962  -3.150780   1.749683
 H     -0.521196  -2.467686   2.607403
 C     -0.991344  -4.426456   1.798099
 H     -1.557158  -4.733466   2.692268
 C     -0.858888  -5.308382   0.708147
 H     -1.319316  -6.308396   0.745338
 C     -0.137173  -4.903086  -0.427512
 H     -0.027462  -5.585323  -1.285764
 C      0.457094  -3.626924  -0.474761
 H      1.031882  -3.332937  -1.366623
 C      2.069814  -1.005506   2.104915
 C      2.213838   0.157667   2.891198
 H      1.658546   1.069114   2.628513
 C      3.067789   0.165105   4.009575
 H      3.167306   1.081982   4.612126
 C      3.786269  -0.991714   4.359796
 H      4.452699  -0.986388   5.236969
 C      3.641611  -2.159884   3.588447
 H      4.193090  -3.074279   3.859867
 C      2.786572  -2.168540   2.472640
 H      2.668308  -3.096567   1.891644
 C      1.010993   0.973130  -2.244065
 H      1.318342   1.846561  -1.635307
 C     -0.947941  -0.563180  -2.368123
 H     -1.225971  -0.375620  -3.427527
 H     -0.188309  -1.377176  -2.376431
 C     -2.145010  -1.032412  -1.588593
 C     -3.098959  -1.214735   0.538618
 C     -4.236224  -2.275075  -1.363945
 C     -3.173752  -1.763044  -2.164123
 H     -3.160194  -1.957354  -3.247887
 C      0.964554   1.433837  -3.711282
 H      1.940004   1.866769  -4.009786
 H      0.734383   0.605200  -4.411597
 H      0.196214   2.223819  -3.833350
 C     -1.151732   0.559475   2.046247
 C      0.379653   1.958129   0.681281
 H     -0.988181   1.439179  -1.956679
 H     -1.765973   1.757273   0.074547
 C     -2.648546   2.753710  -0.935960
 C     -2.348832   3.994491  -0.134775
 C     -4.015416   2.256407  -0.402247
 C     -3.241897   4.090260   0.955577
 C     -1.400582   4.996329  -0.396976
 C     -4.155738   2.877881   1.004434
 H     -4.780134   2.680711  -1.092977
 C     -3.184717   5.204116   1.809381
 C     -1.347496   6.111980   0.457629
 H     -0.724178   4.903432  -1.261688
 H     -5.200138   3.137792   1.274696
 C     -2.232619   6.212214   1.553185
 H     -3.876939   5.297789   2.662421
 H     -0.617502   6.916187   0.271430
 H     -2.183142   7.094113   2.212275
 O     -2.224149   2.539739  -2.107698
 H     -4.118823   1.155420  -0.435247
 H     -3.789467   2.169690   1.782219
 C     -4.183292  -1.993420   0.048797
 C     -5.216039  -2.485572   0.905084
 C     -5.324661  -3.039471  -1.878716
 C     -6.262697  -3.229872   0.375087
 H     -5.169971  -2.266158   1.983733
 C     -6.316739  -3.506921  -1.022564
 H     -5.366957  -3.254414  -2.958305
 H     -7.058435  -3.609334   1.034939
 H     -7.154545  -4.097746  -1.425098
 H     -3.053113  -0.970114   1.610729
---------------------------------------
Mn18/TS-ii_re
Frequencies, energies and thermodynamic properties:
 Lowest Vibrational Mode (1/cm) =-254.9322
 2nd Lowest Vibrational Mode (1/cm) =                       15.7251
 E(RB-P86) (a.u.) =                                       -4828.83934315
 Thermal correction to Enthalpy (a.u.) =                    0.760568
 Thermal correction to Gibbs Free Energy (a.u.) =           0.631036
 Total Entropy (cal/Kmol) =                                 272.623
 E(RPBE1PBE) (a.u.) =                                     -4828.13944807
Optimised cartesian coordinates (Angstrom):
 Fe-4.116333  -0.428780  -1.134824
 Mn0.435540  -0.744098   0.505230
 P     -1.197055   0.823231   0.520767
 O      1.264292  -0.364332   3.304025
 O     -1.197400  -3.033616   1.364880
```

N	0.137888	-1.087795	-1.548215
N	1.891275	0.471571	-0.267743
C	-2.372039	0.620537	-0.881568
C	-2.229745	-0.371113	-1.948368
C	-3.226393	-0.066568	-2.949672
H	-3.401983	-0.630765	-3.874116
C	-3.984047	1.076744	-2.519294
H	-4.828402	1.528813	-3.056770
C	-3.468525	1.499001	-1.247043
H	-3.836728	2.345612	-0.653692
C	-4.358179	-1.884828	0.302947
H	-3.559924	-2.298540	0.932617
C	-4.753058	-2.382502	-0.988415
H	-4.314527	-3.243675	-1.510430
C	-5.809228	-1.539616	-1.487924
H	-6.311515	-1.641026	-2.459303
C	-6.069733	-0.521912	-0.502072
H	-6.806304	0.287914	-0.590795
C	-5.171599	-0.732569	0.603369
H	-5.102879	-0.114976	1.508186
C	-0.570052	2.563501	0.300192
C	0.160492	3.136074	1.370033
H	0.273998	2.580419	2.315448
C	0.735569	4.411147	1.244178
H	1.291699	4.844757	2.090709
C	0.604958	5.131404	0.041090
H	1.056989	6.131014	-0.058158
C	-0.104136	4.565423	-1.032055
H	-0.212259	5.120392	-1.977776
C	-0.687858	3.289900	-0.905080
H	-1.253052	2.869379	-1.751177
C	-2.296612	1.040817	2.010270
C	-2.432847	-0.002989	2.950453
H	-1.868214	-0.936636	2.817961
C	-3.289947	0.137326	4.057574
H	-3.383210	-0.687703	4.781650
C	-4.019597	1.324955	4.242291
H	-4.688643	1.435528	5.110466
C	-3.882925	2.375801	3.316175
H	-4.443509	3.313838	3.456681
C	-3.024652	2.237129	2.211506
H	-2.913762	3.076395	1.507099
C	-1.223731	-1.514479	-2.015198
H	-1.529028	-2.290082	-1.285208
C	0.725190	0.001498	-2.357373
H	1.017760	-0.336356	-3.374841
H	-0.046056	0.792746	-2.494637
C	1.907423	0.601743	-1.647019
C	2.868470	1.065317	0.432874
C	3.951874	1.937231	-1.592053
C	2.907168	1.292548	-2.315690
H	2.881609	1.354630	-3.414813
C	-1.173624	-2.184514	-3.399036
H	-2.148116	-2.656832	-3.634318
H	-0.940736	-1.466974	-4.212065
H	-0.404545	-2.983237	-3.400985
C	0.923739	-0.501069	2.177098
C	-0.589767	-2.077340	1.010703
H	0.776771	-1.925484	-1.670448
H	1.585627	-1.928726	0.373090
C	2.411337	-3.106566	-0.480938
C	3.755436	-2.647381	0.019387
C	1.998189	-4.250136	0.479668
C	4.008407	-3.202388	1.294502
C	4.712074	-1.858621	-0.640823
C	2.815697	-4.014728	1.768313
C	5.237336	-2.960056	1.929631
C	5.943186	-1.620721	-0.003158
H	4.495727	-1.453195	-1.642377
C	6.200932	-2.166516	1.273213
H	5.454412	-3.389920	2.921464
H	6.714933	-1.013011	-0.502108
H	7.171869	-1.976889	1.758912
O	2.027745	-3.014328	-1.682257
H	3.112288	-4.950287	2.286060
H	2.225503	-3.416613	2.499604
H	0.904990	-4.318807	0.629385
H	2.328235	-5.189489	-0.021153
C	3.920106	1.819528	-0.156167
C	4.937452	2.447889	0.625623
C	5.004550	2.679817	-2.204210
C	5.948697	3.168231	0.001652
H	4.907413	2.352948	1.722747
C	5.981825	3.283498	-1.419139
H	5.030881	2.769576	-3.301878

```
 H      6.732122   3.653779   0.604245
 H      6.791421   3.857578  -1.896734
 H      2.839008   0.945277   1.526447
---------------------------------------
Mn18/TS-iii
Frequencies, energies and thermodynamic properties:
 Lowest Vibrational Mode (1/cm) =-597.9180
 2nd Lowest Vibrational Mode (1/cm) =                     20.1070
 E(RB-P86) (a.u.) =                                     -4406.09398889
 Thermal correction to Enthalpy (a.u.) =                  0.605585
 Thermal correction to Gibbs Free Energy (a.u.) =         0.496031
 Total Entropy (cal/Kmol) =                               230.575
 E(RPBE1PBE) (a.u.) =                                   -4405.39963279
Optimised cartesian coordinates (Angstrom):
 Fe3.426275  -0.992956   0.736777
 Mn-0.647053  -0.275603  -1.773128
 P      0.340070   0.675596   0.037604
 O     -1.434313   2.267956  -3.060055
 O      1.767239  -0.304310  -3.455820
 N     -0.385429  -2.182041  -0.956562
 N     -2.482981  -0.530273  -0.845819
 C      1.434234  -0.518721   0.901500
 C      1.595455  -1.928456   0.541579
 C      2.378375  -2.539960   1.590389
 H      2.697621  -3.589481   1.620447
 C      2.712402  -1.544580   2.573383
 H      3.321187  -1.703697   3.473746
 C      2.141542  -0.295950   2.151021
 H      2.220559   0.658675   2.686777
 C      4.240953  -0.332663  -1.039663
 H      3.675488  -0.040450  -1.933353
 C      4.703274  -1.660884  -0.736826
 H      4.557063  -2.553142  -1.360403
 C      5.371113  -1.620730   0.539396
 H      5.818572  -2.477179   1.061482
 C      5.323914  -0.263996   1.023484
 H      5.730237   0.093717   1.979164
 C      4.622698   0.532014   0.049550
 H      4.401115   1.604361   0.127918
 C     -0.861215   1.213135   1.354939
 C     -1.669661   2.343469   1.084274
 H     -1.532703   2.904733   0.145313
 C     -2.640781   2.766418   2.006636
 H     -3.255429   3.653189   1.783780
 C     -2.829083   2.059598   3.209887
 H     -3.590786   2.390945   3.933381
 C     -2.040681   0.928054   3.480700
 H     -2.180710   0.368033   4.419181
 C     -1.063412   0.505198   2.559009
 H     -0.447739  -0.377894   2.789486
 C      1.368638   2.221242  -0.129464
 C      1.886936   2.606387  -1.384539
 H      1.663754   2.010031  -2.279996
 C      2.695679   3.751706  -1.505263
 H      3.090038   4.036932  -2.493544
 C      2.994766   4.530370  -0.373743
 H      3.626326   5.427892  -0.469101
 C      2.473749   4.161686   0.880554
 H      2.693851   4.770317   1.772146
 C      1.662935   3.020046   1.001046
 H      1.245645   2.758514   1.985928
 C      0.981558  -2.629722  -0.680653
 H      1.581939  -2.340460  -1.568404
 C     -1.328437  -2.449669   0.112199
 H     -1.565605  -3.536419   0.227572
 H     -0.936589  -2.133731   1.121228
 C     -2.610823  -1.692382  -0.103713
 C     -3.562146   0.241808  -1.022313
 C     -4.995348  -1.287811   0.254626
 C     -3.831198  -2.088454   0.428462
 C      1.089779  -4.166104  -0.561767
 H      2.150030  -4.491437  -0.531195
 H      0.593924  -4.555462   0.351685
 H      0.611535  -4.638732  -1.443544
 C     -1.119838   1.251393  -2.546027
 C      0.837150  -0.286446  -2.725156
 H     -1.016729  -1.797501  -2.384151
 H     -1.361318  -1.215372  -3.007397
 H     -3.893231  -3.030111   0.995902
 C     -4.847086  -0.067280  -0.498276
 C     -5.979430   0.778757  -0.707100
 C     -6.280625  -1.625440   0.776511
 C     -7.216559   0.424471  -0.184593
 H     -5.856489   1.708567  -1.285085
 C     -7.365501  -0.783266   0.559917
```

```
 H     -6.398553  -2.557516   1.351646
 H     -8.090155   1.075451  -0.344166
 H     -8.354286  -1.048600   0.966322
 H     -3.430952   1.156438  -1.620942
---------------------------------------
Mn19/i
Frequencies, energies and thermodynamic properties:
 Lowest Vibrational Mode (1/cm) =                         17.6313
 2nd Lowest Vibrational Mode (1/cm) =                     21.1693
 E(RB-P86) (a.u.) =                                      -4519.14896017
 Thermal correction to Enthalpy (a.u.) =                  0.691600
 Thermal correction to Gibbs Free Energy (a.u.) =         0.566473
 Total Entropy (cal/Kmol) =                               263.351
 E(RPBE1PBE) (a.u.) =                                    -4518.45474537
Optimised cartesian coordinates (Angstrom):
 Fe-2.421403  -1.796499  -1.547997
 Mn0.724574  -1.102912   1.960434
 P      0.035362   0.101723   0.255501
 O      0.836587   1.083909   3.962839
 O     -1.924692  -1.779001   3.033650
 N      0.948497  -2.691456   0.940701
 N      2.771883  -1.038346   1.711633
 C     -0.574555  -1.030466  -1.072903
 C     -0.550404  -2.488782  -1.014231
 C     -0.927444  -2.978008  -2.319458
 H     -1.027097  -4.032977  -2.605885
 C     -1.186486  -1.853167  -3.179503
 H     -1.511576  -1.903025  -4.227782
 C     -0.976901  -0.653515  -2.415097
 H     -1.094717   0.372772  -2.786227
 C     -3.726452  -1.699716   0.043331
 H     -3.436523  -1.595310   1.097127
 C     -3.888437  -2.941646  -0.665926
 H     -3.754843  -3.947572  -0.245402
 C     -4.233249  -2.635045  -2.031272
 H     -4.405396  -3.365062  -2.833694
 C     -4.287493  -1.200981  -2.164415
 H     -4.507074  -0.647233  -3.087273
 C     -3.971636  -0.622440  -0.883800
 H     -3.901059   0.449031  -0.655003
 C      1.422043   0.992169  -0.581055
 C      1.929116   2.179158   0.002482
 H      1.439838   2.599700   0.896832
 C      3.036906   2.848319  -0.526970
 H      3.377725   3.771053  -0.037601
 C      3.718849   2.348811  -1.681488
 C      3.217386   1.141185  -2.255919
 H      3.698562   0.706982  -3.143071
 C      2.102152   0.487873  -1.712907
 H      1.747857  -0.434254  -2.199754
 C     -1.238918   1.425303   0.423781
 C     -2.090697   1.488010   1.549514
 H     -1.977122   0.756780   2.362944
 C     -3.090910   2.461034   1.666072
 H     -3.718935   2.461759   2.567383
 C     -3.292771   3.440596   0.644959
 C     -2.422623   3.380729  -0.489043
 H     -2.518203   4.113942  -1.301678
 C     -1.427610   2.401879  -0.584432
 H     -0.769398   2.411151  -1.468105
 C     -0.174395  -3.320077   0.222751
 H     -1.035252  -3.275580   0.919468
 C      2.237093  -2.924800   0.317603
 H      2.576120  -3.986062   0.406801
 H      2.212701  -2.727777  -0.788498
 C      3.274806  -2.029630   0.920623
 C      3.650393  -0.164524   2.279368
 H      3.213033   0.624559   2.907785
 C      5.031598  -0.252650   2.097130
 H      5.690081   0.480201   2.586547
 C      5.552138  -1.284064   1.290285
 C      4.655939  -2.181911   0.697843
 H      5.011445  -3.004606   0.058664
 C      0.041547  -4.806504  -0.119679
 H     -0.904167  -5.259517  -0.480323
 H      0.806751  -4.959868  -0.908120
 H      0.356699  -5.362690   0.786991
 C      0.783597   0.212087   3.158902
 C     -0.882400  -1.489734   2.540527
 H      6.636645  -1.382940   1.129659
 N      4.811120   3.005026  -2.213624
 C      5.482920   2.468137  -3.388249
 C      5.295412   4.234434  -1.601896
 H      4.801696   2.403154  -4.267281
 H      6.325206   3.129370  -3.661567
```

```
 H      5.894773   1.449079  -3.206914
 H      4.521295   5.035357  -1.600495
 H      5.620531   4.079543  -0.547798
 H      6.165517   4.607261  -2.172212
 N     -4.273656   4.404422   0.751708
 C     -5.143087   4.435260   1.919825
 C     -4.451894   5.387751  -0.307729
 H     -4.571775   4.590628   2.863107
 H     -5.860998   5.269133   1.817797
 H     -5.727614   3.493813   2.031623
 H     -3.538493   6.005528  -0.463537
 H     -4.707566   4.912744  -1.282266
 H     -5.278243   6.069607  -0.036650
---------------------------------------
Mn19/ii
Frequencies, energies and thermodynamic properties:
 Lowest Vibrational Mode (1/cm) =                          15.1728
 2nd Lowest Vibrational Mode (1/cm) =                      19.1662
 E(RB-P86) (a.u.) =                                      -4674.08432926
 Thermal correction to Enthalpy (a.u.) =                   0.776231
 Thermal correction to Gibbs Free Energy (a.u.) =          0.635678
 Total Entropy (cal/Kmol) =                                295.819
 E(RPBE1PBE) (a.u.) =                                    -4673.39872920
Optimised cartesian coordinates (Angstrom):
 Fe2.085035  -1.901241   2.007972
 Mn-1.103264  -0.862722  -1.378592
 P      0.258280   0.333063  -0.128594
 O     -0.676026   0.632867  -3.900470
 O      0.905693  -2.773983  -2.346927
 N     -1.753442  -1.939858   0.084915
 N     -2.897914   0.100322  -1.033557
 C      0.615852  -0.587548   1.430615
 C      0.031580  -1.871388   1.807707
 C      0.410596  -2.129097   3.177104
 H      0.150327  -3.023837   3.757244
 C      1.219119  -1.035987   3.649214
 H      1.675092  -0.953315   4.645289
 C      1.353788  -0.088428   2.576420
 H      1.912486   0.855528   2.618434
 C      3.051633  -2.727099   0.386439
 H      2.663024  -2.753728  -0.639780
 C      2.824515  -3.724319   1.398934
 H      2.241586  -4.647225   1.276855
 C      3.474942  -3.288808   2.608975
 H      3.471812  -3.819095   3.570832
 C      4.107641  -2.021666   2.342082
 H      4.670861  -1.417262   3.065919
 C      3.844139  -1.672897   0.969726
 H      4.166017  -0.755510   0.459606
 C     -0.554997   1.873843   0.483202
 C     -0.709444   2.964771  -0.407579
 H     -0.274980   2.908333  -1.419555
 C     -1.396420   4.125810  -0.041418
 H     -1.474743   4.943226  -0.771303
 C     -1.989262   4.254975   1.254830
 C     -1.846429   3.146689   2.144438
 H     -2.279025   3.185893   3.153534
 C     -1.148984   1.993099   1.760394
 H     -1.057021   1.171571   2.487986
 C      1.880134   0.958219  -0.743084
 C      2.518023   0.384544  -1.866223
 H      2.033205  -0.437193  -2.412755
 C      3.767454   0.830791  -2.313507
 H      4.210699   0.348692  -3.195472
 C      4.454904   1.893980  -1.649741
 C      3.803205   2.477452  -0.517759
 H      4.273655   3.307132   0.027483
 C      2.553046   2.019339  -0.089026
 H      2.082932   2.516457   0.774670
 C     -0.830264  -2.758970   0.900784
 H     -0.149566  -3.242183   0.171325
 C     -2.863923  -1.394760   0.853988
 H     -3.590998  -2.175012   1.184884
 H     -2.508126  -0.911890   1.802933
 C     -3.583692  -0.352420   0.054933
 C     -3.472847   1.067955  -1.800236
 C     -4.732622   1.603086  -1.524099
 C     -5.446251   1.129653  -0.405849
 C     -4.859637   0.138253   0.389789
 C     -1.526549  -3.895282   1.673832
 H     -0.775723  -4.592969   2.096927
 H     -2.150803  -3.525380   2.513063
 H     -2.169734  -4.464628   0.972534
 C     -0.841350   0.036109  -2.889266
 C      0.135315  -1.988549  -1.900470
```

H   -2.490904 -3.220237 -1.048030
O   -2.854643 -4.010055 -1.548562
C   -4.244220 -3.806892 -1.762687
H   -4.443927 -2.816339 -2.244046
H   -4.813324 -3.804970 -0.797292
H   -5.378376 -0.264534   1.272982
H   -5.147821   2.379686 -2.183174
H   -2.891124   1.410931 -2.667831
C   -4.784309 -4.917377 -2.657534
H   -5.872319 -4.791571 -2.836169
H   -4.267269 -4.915190 -3.640214
H   -4.622311 -5.911481 -2.189796
H   -6.443503   1.527575 -0.163103
N   5.683569   2.343587 -2.085241
C   6.355789   3.429184 -1.384305
C   6.311446   1.738532 -3.252100
H   6.556970   3.176805 -0.318563
H   7.326392   3.632188 -1.871975
H   5.762026   4.371442 -1.401350
H   6.509491   0.652498 -3.105002
H   5.685348   1.845949 -4.166795
H   7.279243   2.236024 -3.444545
N   -2.667153   5.398294   1.626525
C   -3.266267   5.493164   2.950237
C   -2.799763   6.507464   0.692163
H   -2.507813   5.396852   3.760138
H   -3.754545   6.478451   3.059905
H   -4.038978   4.708599   3.118647
H   -1.810335   6.913390   0.381554
H   -3.348968   6.215390 -0.231901
H   -3.363369   7.325709   1.175997

Mn19/iii
Frequencies, energies and thermodynamic properties:
Lowest Vibrational Mode (1/cm) =   19.6110
2nd Lowest Vibrational Mode (1/cm) =   21.2372
E(RB-P86) (a.u.) =   -4675.25680003
Thermal correction to Enthalpy (a.u.) =   0.792413
Thermal correction to Gibbs Free Energy (a.u.) =   0.653798
Total Entropy (cal/Kmol) =   291.740
E(RPBE1PBE) (a.u.) =   -4674.56819091
Optimised cartesian coordinates (Angstrom):
Fe2.023492 -2.007534   1.951873
Mn-1.137445 -0.852088 -1.577553
P   0.222389   0.281743 -0.134896
O   -0.310504   0.921771 -3.797111
O   0.869551 -2.837789 -2.409090
N   -1.865888 -1.984217   0.022714
N   -2.823569   0.245033 -1.085664
C   0.570062 -0.662567   1.399763
C   -0.031154 -1.945368   1.745908
C   0.343299 -2.232091   3.111810
H   0.070648 -3.133410   3.675534
C   1.168403 -1.161689   3.606149
H   1.626014 -1.106358   4.603341
C   1.319316 -0.195829   2.552399
H   1.892654   0.738322   2.611343
C   2.999990 -2.809924   0.323157
H   2.629515 -2.802097 -0.709662
C   2.734760 -3.829080   1.303611
H   2.134591 -4.735473   1.146679
C   3.373421 -3.439879   2.535522
H   3.342523 -3.995296   3.482606
C   4.036969 -2.179610   2.313574
H   4.600305 -1.607135   3.062845
C   3.804092 -1.788477   0.947230
H   4.154449 -0.865625   0.466633
C   -0.525899   1.852446   0.495048
C   -0.675828   2.939267 -0.401266
H   -0.302119   2.851224 -1.434927
C   -1.277336   4.140519 -0.013112
H   -1.356630   4.950921 -0.750643
C   -1.781364   4.318583   1.313949
C   -1.639300   3.216923   2.211502
H   -2.003915   3.292269   3.245076
C   -1.028942   2.022472   1.804021
H   -0.934678   1.208329   2.539422
C   1.871706   0.874415 -0.727006
C   2.534740   0.281122 -1.825112
H   2.057153 -0.537051 -2.382249
C   3.804951   0.701192 -2.238515
H   4.264862   0.202923 -3.102779
C   4.491989   1.756985 -1.563573
C   3.817619   2.359458 -0.455516
H   4.286494   3.184119   0.098654

```
 C      2.546621   1.927530  -0.061860
 H      2.061523   2.439743   0.784348
 C     -0.924236  -2.788633   0.831287
 H     -0.263557  -3.293783   0.096396
 C     -2.778977  -1.203245   0.852668
 H     -3.540183  -1.831346   1.377206
 H     -2.248423  -0.652254   1.677400
 C     -3.483082  -0.159671   0.035478
 C     -3.371898   1.228406  -1.844760
 C     -4.591698   1.836752  -1.532474
 C     -5.284967   1.413810  -0.384381
 C     -4.719716   0.403982   0.404676
 C     -1.635435  -3.911840   1.618950
 H     -0.898275  -4.612952   2.061524
 H     -2.260331  -3.517671   2.447364
 H     -2.285254  -4.485379   0.927900
 C     -0.652436   0.208139  -2.919368
 C      0.102698  -2.022544  -2.026853
 H     -2.199233  -1.410243  -2.828849
 H     -2.274482  -2.030257  -2.282341
 H     -2.729381  -3.078071  -0.784421
 O     -3.206214  -3.784376  -1.398692
 C     -4.604557  -3.572296  -1.372301
 H     -5.013050  -3.643373  -0.330362
 H     -4.875298  -2.546217  -1.736577
 H     -5.226735   0.040246   1.311063
 H     -4.988891   2.625502  -2.188024
 H     -2.806290   1.528132  -2.739152
 C     -5.302676  -4.609682  -2.249156
 H     -5.078433  -5.636907  -1.891014
 H     -6.404142  -4.470507  -2.238702
 H     -4.953462  -4.534359  -3.300811
 H     -6.252081   1.863684  -0.111569
 N     -2.376368   5.500464   1.707166
 C     -2.499836   6.605670   0.767184
 C     -2.877476   5.647818   3.066186
 H     -3.109782   6.332567  -0.123851
 H     -2.996321   7.456335   1.268328
 H     -1.508561   6.957857   0.400994
 H     -3.667637   4.899870   3.304832
 H     -2.069981   5.536787   3.825527
 H     -3.317891   6.654343   3.185476
 N      5.740699   2.181576  -1.966390
 C      6.391606   1.555677  -3.109266
 C      6.413548   3.256910  -1.250599
 H      5.792447   1.662996  -4.041787
 H      7.371420   2.037338  -3.279968
 H      6.569729   0.468283  -2.946698
 H      5.837884   4.209770  -1.286603
 H      6.581141   3.004773  -0.178923
 H      7.400523   3.440599  -1.712370
---------------------------------------
Mn19/iv
Frequencies, energies and thermodynamic properties:
 Lowest Vibrational Mode (1/cm) =                        13.1176
 2nd Lowest Vibrational Mode (1/cm) =                    18.5105
 E(RB-P86) (a.u.) =                                     -4675.28352932
 Thermal correction to Enthalpy (a.u.) =                 0.796995
 Thermal correction to Gibbs Free Energy (a.u.) =        0.656773
 Total Entropy (cal/Kmol) =                              295.122
 E(RPBE1PBE) (a.u.) =                                   -4674.59336214
Optimised cartesian coordinates (Angstrom):
 Fe-2.187921  -1.754689  -2.061786
 Mn1.166678  -1.004012   1.391318
 P     -0.200027   0.312861   0.103795
 O      0.656690   0.497052   3.865832
 O     -0.794958  -3.024211   2.242809
 N      1.782712  -2.066039  -0.379536
 N      2.895425   0.002099   0.920641
 C     -0.667576  -0.494390  -1.489974
 C     -0.135319  -1.768122  -1.971331
 C     -0.588989  -1.937111  -3.334151
 H     -0.379906  -2.798677  -3.980665
 C     -1.398174  -0.805134  -3.696077
 H     -1.906570  -0.659193  -4.658755
 C     -1.454440   0.079312  -2.565252
 H     -1.997937   1.031886  -2.519741
 C     -3.070665  -2.689510  -0.452149
 H     -2.618950  -2.808655   0.541548
 C     -2.934622  -3.605120  -1.554642
 H     -2.370098  -4.547327  -1.546137
 C     -3.650331  -3.058885  -2.679727
 H     -3.721873  -3.508027  -3.679483
 C     -4.232519  -1.806000  -2.270186
 H     -4.824764  -1.132625  -2.904377
```

```
 C     -3.872515  -1.575980  -0.895216
 H     -4.136309  -0.697272  -0.292382
 C      0.572625   1.899877  -0.470846
 C      0.853784   2.892961   0.499877
 H      0.548476   2.731455   1.547365
 C      1.501528   4.089041   0.172485
 H      1.681508   4.824672   0.968586
 C      1.927875   4.354984  -1.166585
 C      1.660689   3.345728  -2.139903
 H      1.963923   3.489216  -3.186197
 C      1.001690   2.156809  -1.791891
 H      0.809005   1.419141  -2.586943
 C     -1.802461   0.924405   0.801258
 C     -2.425190   0.264145   1.884132
 H     -1.942149  -0.614311   2.336698
 C     -3.655679   0.688874   2.401440
 H     -4.088582   0.136014   3.246380
 C     -4.336575   1.820599   1.855413
 C     -3.701664   2.490894   0.763245
 H     -4.169799   3.373337   0.305486
 C     -2.470910   2.050179   0.262974
 H     -2.011523   2.612600  -0.565902
 C      0.730713  -2.756069  -1.199899
 H      0.094328  -3.262403  -0.446902
 C      2.706444  -1.223273  -1.167462
 H      3.383267  -1.816028  -1.820686
 H      2.097812  -0.577956  -1.840108
 C      3.504091  -0.329879  -0.255067
 C      3.536938   0.874572   1.742466
 C      4.789492   1.423814   1.445800
 C      5.427972   1.061973   0.247684
 C      4.767892   0.169654  -0.611213
 C      1.334864  -3.847361  -2.100550
 H      0.532773  -4.463871  -2.552993
 H      1.944879  -3.427593  -2.926411
 H      1.973801  -4.523317  -1.496519
 C      0.854370  -0.104217   2.861173
 C     -0.044120  -2.187678   1.858077
 H      2.184712  -1.930491   2.219560
 H      2.628155  -3.259936   1.852698
 H      2.341310  -2.827543   0.061281
 O      3.028121  -4.050262   1.365931
 C      4.427006  -4.075856   1.662350
 H      4.898463  -4.747285   0.911673
 H      4.884727  -3.066963   1.520264
 H      5.222604  -0.143795  -1.563222
 H      5.254928   2.121986   2.157291
 H      3.019789   1.126416   2.679579
 C      4.717312  -4.585763   3.073993
 H      4.281552  -5.595607   3.223821
 H      5.811370  -4.648644   3.255215
 H      4.284622  -3.907819   3.840121
 H      6.418490   1.465100  -0.012726
 N     -5.545294   2.252050   2.363473
 C     -6.212628   3.407678   1.781015
 C     -6.165734   1.541727   3.472881
 H     -6.466280   3.250207   0.707665
 H     -7.154726   3.598311   2.326501
 H     -5.589418   4.328328   1.847431
 H     -6.387707   0.479837   3.219695
 H     -5.523190   1.542727   4.382645
 H     -7.120355   2.033948   3.733068
 N      2.568689   5.533210  -1.500516
 C      2.819123   6.542907  -0.482560
 C      2.990677   5.768750  -2.873092
 H      3.469179   6.161425   0.337922
 H      3.330966   7.406900  -0.944184
 H      1.876933   6.914432  -0.018324
 H      3.716526   5.000978  -3.226750
 H      2.131850   5.769178  -3.583140
 H      3.484195   6.755477  -2.939366
---------------------------------------
Mn19/v
Frequencies, energies and thermodynamic properties:
 Lowest Vibrational Mode (1/cm) =                        18.8243
 2nd Lowest Vibrational Mode (1/cm) =                    21.8165
 E(RB-P86) (a.u.) =                                     -4520.34473208
 Thermal correction to Enthalpy (a.u.) =                 0.712469
 Thermal correction to Gibbs Free Energy (a.u.) =        0.586924
 Total Entropy (cal/Kmol) =                              264.232
 E(RPBE1PBE) (a.u.) =                                   -4519.65064033
Optimised cartesian coordinates (Angstrom):
 Fe-2.472962  -1.656070  -1.604840
 Mn0.676727  -1.171436   2.072916
 P      0.077050   0.068438   0.242745
```

O	0.757365	1.095921	3.939435
O	-1.977605	-1.905076	3.103609
N	0.863145	-2.915540	0.825415
N	2.690239	-1.155156	1.691830
C	-0.591961	-0.977656	-1.127627
C	-0.637993	-2.439876	-1.122739
C	-1.043632	-2.863412	-2.445232
H	-1.192757	-3.899788	-2.773234
C	-1.261703	-1.697546	-3.257305
H	-1.602214	-1.695550	-4.301572
C	-0.990971	-0.539490	-2.451577
H	-1.069537	0.504942	-2.779851
C	-3.751639	-1.572540	0.006772
H	-3.443121	-1.520940	1.059349
C	-3.970826	-2.778496	-0.748645
H	-3.870708	-3.805047	-0.370802
C	-4.323304	-2.404381	-2.094818
H	-4.533024	-3.094627	-2.923032
C	-4.325421	-0.965451	-2.169855
H	-4.535862	-0.367163	-3.066600
C	-3.969998	-0.451017	-0.872863
H	-3.855772	0.606677	-0.602177
C	1.476979	0.957725	-0.592391
C	2.084718	2.034478	0.099668
H	1.670417	2.359943	1.068726
C	3.196356	2.713334	-0.411186
H	3.615447	3.547705	0.168088
C	3.784064	2.333229	-1.658352
C	3.184649	1.234444	-2.344234
H	3.591453	0.891800	-3.305757
C	2.064122	0.573045	-1.818088
H	1.634024	-0.259409	-2.397528
C	-1.158580	1.436102	0.425315
C	-2.050387	1.464540	1.520735
H	-1.995140	0.676091	2.285923
C	-3.017799	2.467787	1.662625
H	-3.681643	2.436869	2.537529
C	-3.140921	3.517381	0.700591
C	-2.231094	3.492030	-0.402585
H	-2.266812	4.277890	-1.169771
C	-1.271145	2.480292	-0.523604
H	-0.578890	2.516399	-1.380362
C	-0.335397	-3.372705	0.043546
H	-1.168462	-3.305627	0.771623
C	2.113076	-2.839836	0.038766
H	2.495848	-3.838566	-0.263753
H	1.886534	-2.284920	-0.898994
C	3.164126	-2.076792	0.801718
C	3.601770	-0.383404	2.343424
H	3.193837	0.353559	3.050056
C	4.983298	-0.508224	2.158171
H	5.663919	0.147117	2.721931
C	5.470020	-1.474715	1.262027
C	4.536612	-2.268486	0.576676
H	4.862902	-3.038341	-0.139124
C	-0.213086	-4.843177	-0.390120
H	-1.178209	-5.207043	-0.795092
H	0.559395	-4.993539	-1.171639
H	0.039637	-5.482219	0.481131
C	0.716713	0.188084	3.172874
C	-0.933369	-1.583181	2.635117
H	0.998317	-3.599741	1.582039
H	1.100827	-2.165897	3.240545
H	6.550385	-1.608413	1.100109
N	4.881780	2.998422	-2.172300
C	5.464183	4.117099	-1.446022
C	5.456389	2.581996	-3.442486
H	5.837029	3.817984	-0.439542
H	6.321494	4.516439	-2.018040
H	4.736545	4.948136	-1.299977
H	5.818303	1.528455	-3.415108
H	4.726809	2.662883	-4.280922
H	6.319605	3.229167	-3.682586
N	-4.086989	4.513960	0.833881
C	-4.190058	5.563191	-0.170200
C	-4.998648	4.507599	1.969087
H	-4.427610	5.156761	-1.179957
H	-4.999811	6.260163	0.112570
H	-3.249273	6.152915	-0.258340
H	-5.630377	3.590282	1.995525
H	-4.457870	4.570726	2.940691
H	-5.672695	5.380699	1.900454

Mn19/vi_R

Frequencies, energies and thermodynamic properties:

```
 Lowest Vibrational Mode (1/cm) =                         12.1772
 2nd Lowest Vibrational Mode (1/cm) =                     17.5921
 E(RB-P86) (a.u.) =                                      -4943.05966315
 Thermal correction to Enthalpy (a.u.) =                  0.865080
 Thermal correction to Gibbs Free Energy (a.u.) =         0.716614
 Total Entropy (cal/Kmol) =                               312.474
 E(RPBE1PBE) (a.u.) =                                    -4942.36027521
Optimised cartesian coordinates (Angstrom):
 Fe-1.799728  -2.265413  -2.434252
 Mn1.237042   0.175142   0.343542
 P     -0.953313   0.184989   0.046241
 O      1.111935   0.429138   3.291788
 O      1.494841  -2.729293   0.720255
 N      1.641769   0.177178  -1.577123
 N      1.611520   2.186935   0.083913
 C     -1.353914  -0.420155  -1.648358
 C     -0.375820  -0.791190  -2.666802
 C     -1.100878  -1.004520  -3.897832
 H     -0.663143  -1.314289  -4.855428
 C     -2.501876  -0.781472  -3.657462
 H     -3.308540  -0.888831  -4.395400
 C     -2.663791  -0.428859  -2.273451
 H     -3.614485  -0.197768  -1.775863
 C     -1.182071  -3.805399  -1.211218
 H     -0.423165  -3.727524  -0.422010
 C     -0.932270  -4.127250  -2.591434
 H      0.047900  -4.345178  -3.036703
 C     -2.190228  -4.090001  -3.293441
 H     -2.336422  -4.270228  -4.366993
 C     -3.219018  -3.747763  -2.344064
 H     -4.286808  -3.621423  -2.568342
 C     -2.596269  -3.568868  -1.058006
 H     -3.100530  -3.279051  -0.126879
 C     -1.645728   1.896454   0.053787
 C     -1.788125   2.567289   1.293708
 H     -1.553520   2.036714   2.231530
 C     -2.227993   3.891819   1.370382
 H     -2.328882   4.356029   2.360947
 C     -2.541283   4.635337   0.188311
 C     -2.380664   3.961405  -1.060832
 H     -2.606392   4.478242  -2.003597
 C     -1.943405   2.630841  -1.116538
 H     -1.849211   2.153045  -2.104235
 C     -2.097555  -0.699600   1.191446
 C     -1.649996  -1.739621   2.037099
 H     -0.587495  -2.020804   2.047981
 C     -2.526799  -2.440465   2.873848
 H     -2.118040  -3.235485   3.512221
 C     -3.921876  -2.131170   2.909957
 C     -4.370811  -1.072278   2.059184
 H     -5.429138  -0.777573   2.050432
 C     -3.477593  -0.381913   1.233367
 H     -3.869425   0.439885   0.612801
 C      1.138037  -0.906436  -2.453451
 H      1.325552  -1.843034  -1.890865
 C      1.614599   1.487257  -2.224265
 H      2.381973   1.589652  -3.028307
 H      0.631666   1.673474  -2.731208
 C      1.817088   2.569741  -1.208471
 C      1.741983   3.123970   1.062890
 H      1.565736   2.780583   2.092257
 C      2.087129   4.451387   0.798949
 H      2.181619   5.162974   1.632253
 C      2.312073   4.845868  -0.533903
 C      2.175935   3.888411  -1.546017
 H      2.342794   4.148253  -2.602316
 C      1.902137  -1.030881  -3.786342
 H      1.607214  -1.960346  -4.314155
 H      1.707839  -0.181034  -4.472462
 H      2.992708  -1.086155  -3.593967
 C      1.163101   0.328707   2.112052
 C      1.334373  -1.567924   0.538041
 H      3.263645  -0.067166  -1.446136
 H      3.445015   0.023702   0.601595
 C      4.470057  -0.008286   0.082850
 C      5.255036  -1.133806   0.743637
 C      5.261500   1.256269   0.525061
 C      6.074577  -0.625664   1.775853
 C      5.240793  -2.503964   0.442023
 C      5.908533   0.881503   1.881010
 H      6.049403   1.423534  -0.242086
 C      6.884223  -1.494808   2.526432
 C      6.055122  -3.375065   1.192936
 H      4.606978  -2.883915  -0.375154
 H      6.862892   1.412501   2.081292
```

```
 C      6.869699  -2.873363   2.228945
 H      7.532450  -1.107456   3.330192
 H      6.061526  -4.453839   0.966896
 H      7.506929  -3.563533   2.805482
 O      4.281748  -0.141306  -1.293576
 H      4.631361   2.167455   0.566761
 H      5.225515   1.131663   2.725512
 H      2.590966   5.882923  -0.775834
 N     -2.978600   5.942513   0.254454
 C     -3.132109   6.598604   1.545647
 C     -3.283823   6.672471  -0.967993
 H     -2.170236   6.656586   2.104202
 H     -3.494346   7.630788   1.388978
 H     -3.868414   6.074688   2.196598
 H     -2.396083   6.763402  -1.634585
 H     -4.096000   6.185543  -1.554372
 H     -3.619007   7.693304  -0.709540
 N     -4.792539  -2.814180   3.732339
 C     -6.208096  -2.470944   3.745890
 C     -4.300588  -3.884928   4.588763
 H     -6.681530  -2.607288   2.747070
 H     -6.734908  -3.127010   4.462269
 H     -6.380436  -1.416399   4.059701
 H     -3.838126  -4.710697   4.001857
 H     -3.540261  -3.522505   5.317300
 H     -5.144297  -4.308925   5.162753
---------------------------------------
Mn19/vi_S
Frequencies, energies and thermodynamic properties:
 Lowest Vibrational Mode (1/cm) =                          14.5237
 2nd Lowest Vibrational Mode (1/cm) =                      17.5223
 E(RB-P86) (a.u.) =                                      -4943.06004541
 Thermal correction to Enthalpy (a.u.) =                   0.865302
 Thermal correction to Gibbs Free Energy (a.u.) =          0.717837
 Total Entropy (cal/Kmol) =                                310.367
 E(RPBE1PBE) (a.u.) =                                    -4942.36058318
Optimised cartesian coordinates (Angstrom):
 Fe-2.741870  -1.835273  -2.165652
 Mn1.071598  -0.528899   0.407070
 P     -0.923707   0.345047   0.029665
 O      1.101213   0.102688   3.300650
 O      0.120853  -3.214976   1.127381
 N      1.408397  -0.909989  -1.488232
 N      2.253233   1.098050  -0.059694
 C     -1.568368  -0.248964  -1.591964
 C     -0.845080  -1.106802  -2.525829
 C     -1.616894  -1.156704  -3.745700
 H     -1.360688  -1.732178  -4.644447
 C     -2.801140  -0.356361  -3.580187
 H     -3.594997  -0.216308  -4.326496
 C     -2.780181   0.198000  -2.254144
 H     -3.545395   0.854005  -1.819333
 C     -2.782200  -3.331464  -0.748935
 H     -2.041005  -3.473962   0.047975
 C     -2.715039  -3.893071  -2.072254
 H     -1.919503  -4.545027  -2.457668
 C     -3.862174  -3.431266  -2.812192
 H     -4.091850  -3.665577  -3.860397
 C     -4.640750  -2.585647  -1.942955
 H     -5.567663  -2.062286  -2.213810
 C     -3.972536  -2.521382  -0.668947
 H     -4.294381  -1.939388   0.204529
 C     -0.844284   2.175362  -0.199552
 C     -0.657530   2.995878   0.940571
 H     -0.632924   2.538844   1.943861
 C     -0.508373   4.381697   0.836545
 H     -0.377102   4.967251   1.756789
 C     -0.523932   5.032211  -0.438307
 C     -0.695036   4.200205  -1.586675
 H     -0.716371   4.640486  -2.592930
 C     -0.849091   2.812731  -1.461066
 H     -0.992259   2.218060  -2.376779
 C     -2.310988   0.162933   1.231563
 C     -2.321980  -0.858197   2.208402
 H     -1.473557  -1.552589   2.287160
 C     -3.394695  -1.022949   3.092796
 H     -3.342899  -1.832273   3.833707
 C     -4.532110  -0.158385   3.046126
 C     -4.515043   0.878817   2.061024
 H     -5.353905   1.583863   1.982309
 C     -3.431648   1.028572   1.189076
 H     -3.456817   1.856287   0.462289
 C      0.496995  -1.794843  -2.251033
 H      0.300992  -2.654704  -1.579155
 C      1.894183   0.220940  -2.276665
```

```
 H      2.603884  -0.083475  -3.082241
 H      1.053935   0.741638  -2.806719
 C      2.560198   1.221345  -1.382312
 C      2.792869   1.990853   0.814356
 H      2.523031   1.861001   1.872160
 C      3.648798   3.019284   0.413720
 H      4.056748   3.709432   1.166650
 C      3.974360   3.143316  -0.950332
 C      3.422727   2.228259  -1.855125
 H      3.649049   2.283491  -2.930687
 C      1.126053  -2.372186  -3.534368
 H      0.475362  -3.162115  -3.961045
 H      1.278290  -1.604066  -4.320263
 H      2.105955  -2.834731  -3.298834
 C      1.092433  -0.150116   2.142960
 C      0.444131  -2.119135   0.804086
 H      2.819632  -1.741908  -1.295147
 H      3.055336  -1.521385   0.741617
 C      3.894699  -2.151382   0.274514
 C      3.850250  -3.553393   0.948696
 C      4.657260  -3.400047   2.261509
 O      3.751073  -2.146382  -1.114047
 H      4.649944   3.939478  -1.298905
 C      5.209333  -1.581724   0.787418
 C      5.642327  -2.290818   1.930185
 C      5.960753  -0.514307   0.272628
 C      6.835117  -1.925337   2.576586
 C      7.158860  -0.150396   0.919453
 H      5.619957   0.016513  -0.631122
 C      7.591039  -0.850050   2.064875
 H      7.185549  -2.476352   3.465301
 H      7.766131   0.680819   0.525562
 H      8.532799  -0.560358   2.558781
 H      5.151958  -4.337176   2.593060
 H      3.992497  -3.082415   3.097889
 H      4.371621  -4.254151   0.259834
 H      2.817285  -3.925716   1.096639
 N     -5.591599  -0.310379   3.915157
 C     -6.735866   0.587693   3.837994
 C     -5.572992  -1.373286   4.911080
 H     -7.239390   0.539427   2.845832
 H     -7.476088   0.301512   4.606956
 H     -6.449050   1.648447   4.019377
 H     -5.514381  -2.382970   4.444717
 H     -4.712747  -1.275134   5.611386
 H     -6.501880  -1.329578   5.507992
 N     -0.380351   6.399980  -0.550716
 C     -0.209777   7.218668   0.641695
 C     -0.389782   7.029198  -1.863905
 H      0.710140   6.948840   1.208806
 H     -0.125365   8.280003   0.345699
 H     -1.073759   7.127503   1.338362
 H      0.435802   6.657426  -2.512651
 H     -1.347429   6.853975  -2.404996
 H     -0.262485   8.120609  -1.746325
---------------------------------------
Mn19/viii
Frequencies, energies and thermodynamic properties:
 Lowest Vibrational Mode (1/cm) =                        13.6730
 2nd Lowest Vibrational Mode (1/cm) =                    20.7334
 E(RB-P86) (a.u.) =                                    -4520.31243743
 Thermal correction to Enthalpy (a.u.) =                 0.707639
 Thermal correction to Gibbs Free Energy (a.u.) =        0.581149
 Total Entropy (cal/Kmol) =                              266.219
 E(RPBE1PBE) (a.u.) =                                  -4519.61857045
Optimised cartesian coordinates (Angstrom):
 Fe-2.506001  -1.669679  -1.574933
 Mn0.661783  -1.175422   2.098714
 P      0.067311   0.022217   0.263620
 O      0.710409   1.258793   3.788165
 O     -2.079506  -1.745956   3.001893
 N      0.832282  -2.842320   0.913902
 N      2.689299  -1.113297   1.674254
 C     -0.611565  -1.032340  -1.080860
 C     -0.681059  -2.488143  -1.044171
 C     -1.082985  -2.925547  -2.360753
 H     -1.248350  -3.966567  -2.667442
 C     -1.271724  -1.771584  -3.200900
 H     -1.597828  -1.781297  -4.250060
 C     -0.991322  -0.601099  -2.414201
 H     -1.044510   0.437754  -2.764772
 C     -3.822963  -1.525820   0.005711
 H     -3.542549  -1.450580   1.064118
 C     -4.040737  -2.747846  -0.721876
 H     -3.964409  -3.764834  -0.313965
```

```
 C     -4.352820  -2.405291  -2.086782
 H     -4.553004  -3.114679  -2.901170
 C     -4.331329  -0.968408  -2.200443
 H     -4.512016  -0.391667  -3.117677
 C     -4.000888  -0.424558  -0.908443
 H     -3.880350   0.638588  -0.662243
 C      1.496565   0.869733  -0.551482
 C      2.083909   1.984100   0.096344
 H      1.652739   2.356507   1.040436
 C      3.197901   2.644368  -0.431642
 H      3.603235   3.509099   0.111413
 C      3.802912   2.209530  -1.652857
 C      3.217348   1.076866  -2.296018
 H      3.635986   0.694873  -3.237250
 C      2.097630   0.431517  -1.752129
 H      1.678858  -0.431994  -2.292188
 C     -1.131923   1.421340   0.436738
 C     -2.033807   1.504100   1.521407
 H     -2.012607   0.742977   2.313890
 C     -2.975697   2.535339   1.625159
 H     -3.646216   2.546245   2.495282
 C     -3.065789   3.558449   0.631724
 C     -2.147341   3.477756  -0.461649
 H     -2.157043   4.240255  -1.252632
 C     -1.211568   2.440824  -0.543568
 H     -0.512394   2.436279  -1.395022
 C     -0.324623  -3.348923   0.177752
 H     -1.184430  -3.290018   0.878156
 C      2.055728  -2.880253   0.145269
 H      2.454262  -3.916980  -0.010619
 H      1.952183  -2.470879  -0.905066
 C      3.129158  -2.057858   0.796362
 C      3.602085  -0.301922   2.269747
 C      4.975435  -0.399740   2.029403
 C      5.438107  -1.380588   1.132115
 C      4.500288  -2.215373   0.512801
 C     -0.187991  -4.838819  -0.212007
 H     -1.148018  -5.241557  -0.596670
 H      0.579829  -5.002924  -0.996887
 H      0.099446  -5.430639   0.681159
 C      0.697268   0.274073   3.131275
 C     -1.001209  -1.495745   2.580464
 H      1.136262  -2.575673   3.074783
 H      1.255366  -1.969173   3.609189
 H      3.205168   0.447546   2.970027
 H      5.667672   0.281993   2.545048
 H      4.815217  -2.998201  -0.194002
 H      6.513409  -1.491929   0.923335
 N      4.901362   2.856439  -2.183612
 C      5.468589   4.012271  -1.503714
 C      5.489515   2.390622  -3.431122
 H      5.826074   3.762575  -0.478763
 H      6.333645   4.386319  -2.080891
 H      4.735744   4.846049  -1.409975
 H      5.856046   1.341442  -3.355711
 H      4.766540   2.433212  -4.277710
 H      6.351563   3.031859  -3.690111
 N     -3.987853   4.580074   0.726778
 C     -4.908943   4.629506   1.853765
 C     -4.054309   5.603957  -0.306753
 H     -4.374821   4.712710   2.827462
 H     -5.563890   5.513604   1.750880
 H     -5.559741   3.726856   1.903139
 H     -3.098322   6.167631  -0.399819
 H     -4.291302   5.175428  -1.307257
 H     -4.849565   6.327825  -0.052019
---------------------------------------
Mn19/ix
Frequencies, energies and thermodynamic properties:
 Lowest Vibrational Mode (1/cm) =                        15.8518
 2nd Lowest Vibrational Mode (1/cm) =                    22.0037
 E(RB-P86) (a.u.) =                                     -4674.09976711
 Thermal correction to Enthalpy (a.u.) =                 0.776949
 Thermal correction to Gibbs Free Energy (a.u.) =        0.642085
 Total Entropy (cal/Kmol) =                              283.845
 E(RPBE1PBE) (a.u.) =                                   -4673.40997443
Optimised cartesian coordinates (Angstrom):
 Fe-2.547946  -1.213557  -2.079562
 Mn0.868034  -1.419358   1.381824
 P     -0.029452   0.199357   0.065804
 O      0.863083   0.304813   3.763995
 O     -1.707251  -2.557432   2.225590
 N      1.101560  -2.662485  -0.335747
 N      2.821322  -0.951031   0.833123
 C     -0.705861  -0.493521  -1.512306
```

C	-0.608583	-1.884989	-1.950378
C	-1.081227	-1.942688	-3.315184
H	-1.154431	-2.846414	-3.933393
C	-1.478981	-0.622575	-3.722865
H	-1.904238	-0.350161	-4.698285
C	-1.257297	0.268120	-2.617494
H	-1.464902	1.345693	-2.610440
C	-3.710794	-1.723442	-0.456282
H	-3.338039	-1.917880	0.557691
C	-3.851182	-2.700275	-1.504184
H	-3.614052	-3.769992	-1.426408
C	-4.336112	-2.025571	-2.681460
H	-4.528206	-2.488935	-3.658576
C	-4.498030	-0.630368	-2.358972
H	-4.834127	0.155637	-3.048627
C	-4.109266	-0.442804	-0.985242
H	-4.091368	0.509590	-0.439456
C	1.192561	1.450932	-0.551989
C	1.702030	2.402805	0.364938
H	1.314682	2.426438	1.397295
C	2.683194	3.331117	0.001411
H	3.030441	4.051569	0.754799
C	3.229478	3.348231	-1.320515
C	2.726979	2.376851	-2.238166
H	3.106309	2.337961	-3.268564
C	1.737042	1.459633	-1.855029
H	1.374960	0.741761	-2.607977
C	-1.371905	1.301176	0.707645
C	-2.160170	0.935390	1.821981
H	-1.963912	-0.010588	2.345868
C	-3.204074	1.743950	2.289357
H	-3.777678	1.405603	3.163095
C	-3.518533	2.985875	1.656423
C	-2.713478	3.359720	0.534972
H	-2.894882	4.309395	0.013031
C	-1.673347	2.538032	0.086792
H	-1.067930	2.883167	-0.766576
C	-0.087604	-3.063573	-1.134340
H	-0.850619	-3.328201	-0.374285
C	2.287234	-2.297160	-1.126376
H	2.757211	-3.171004	-1.629137
H	1.970159	-1.602424	-1.937837
C	3.303033	-1.586056	-0.268585
C	3.685726	-0.235823	1.593534
C	5.049977	-0.128991	1.299675
C	5.554644	-0.797166	0.171058
C	4.664581	-1.536039	-0.620414
C	0.162430	-4.317161	-1.990897
H	-0.789439	-4.684739	-2.423875
H	0.862482	-4.125527	-2.829636
H	0.580320	-5.131285	-1.363993
C	0.867838	-0.402821	2.810874
C	-0.692048	-2.069648	1.851607
H	1.363516	-3.392585	0.380429
H	5.013389	-2.076915	-1.513235
H	5.702664	0.464468	1.956779
H	3.260556	0.260623	2.477908
H	6.623905	-0.745934	-0.085832
O	1.824241	-3.105815	2.041359
C	1.528546	-3.799385	3.214935
C	2.165374	-3.197355	4.479120
H	1.905528	-4.853916	3.115685
H	0.423088	-3.902867	3.396688
H	1.949971	-3.820191	5.375630
H	3.269052	-3.128519	4.365020
H	1.780844	-2.174017	4.671022
N	-4.542730	3.791056	2.110692
C	-4.834643	5.050622	1.440705
C	-5.337325	3.382728	3.260780
H	-5.110512	4.902387	0.371821
H	-5.686533	5.542440	1.944330
H	-3.970093	5.752312	1.470829
H	-5.865459	2.418124	3.083931
H	-4.716504	3.262857	4.177670
H	-6.101488	4.153663	3.468257
N	4.197340	4.262357	-1.689905
C	4.688778	5.236439	-0.726190
C	4.732886	4.249814	-3.043133
H	5.165047	4.751737	0.156680
H	5.448477	5.876714	-1.210361
H	3.877116	5.899014	-0.347854
H	5.228457	3.282988	-3.290364
H	3.942807	4.426460	-3.808521
H	5.486720	5.051611	-3.144808

```
Mn19/x
Frequencies, energies and thermodynamic properties:
 Lowest Vibrational Mode (1/cm) =                        14.9634
 2nd Lowest Vibrational Mode (1/cm) =                    20.2465
 E(RB-P86) (a.u.) =                                    -4829.04157989
 Thermal correction to Enthalpy (a.u.) =                 0.861085
 Thermal correction to Gibbs Free Energy (a.u.) =        0.713338
 Total Entropy (cal/Kmol) =                              310.961
 E(RPBE1PBE) (a.u.) =                                  -4828.35991950
Optimised cartesian coordinates (Angstrom):
 Fe-2.113767  -2.048247  -2.117330
 Mn1.258212  -0.648125   1.139453
 P     -0.463608   0.326441   0.010678
 O      0.446039   0.787923   3.572802
 O     -0.230591  -3.032949   2.000300
 N      1.940898  -1.528609  -0.707315
 N      2.641996   0.770421   0.517818
 C     -0.853813  -0.515361  -1.584067
 C     -0.102242  -1.631346  -2.151911
 C     -0.601418  -1.851218  -3.490719
 H     -0.260354  -2.629263  -4.185220
 C     -1.652175  -0.905353  -3.752793
 H     -2.244411  -0.840178  -4.675517
 C     -1.815881  -0.087595  -2.582662
 H     -2.541704   0.727106  -2.464267
 C     -2.697100  -3.165005  -0.487779
 H     -2.185469  -3.189658   0.483090
 C     -2.415026  -4.021265  -1.609835
 H     -1.659017  -4.817482  -1.640939
 C     -3.282613  -3.636537  -2.693992
 H     -3.299849  -4.083515  -3.697090
 C     -4.104123  -2.543356  -2.239419
 H     -4.856519  -2.010906  -2.836714
 C     -3.740837  -2.249649  -0.877345
 H     -4.161964  -1.453482  -0.249609
 C     -0.105844   2.062242  -0.536445
 C     -0.033453   3.078035   0.448424
 H     -0.257097   2.835083   1.500507
 C      0.305516   4.396960   0.129232
 H      0.335308   5.141844   0.936316
 C      0.610052   4.776428  -1.215982
 C      0.549285   3.748369  -2.204731
 H      0.770250   3.975639  -3.256662
 C      0.200167   2.433174  -1.864553
 H      0.154013   1.684316  -2.670973
 C     -2.104758   0.541274   0.839805
 C     -2.497609  -0.261312   1.934497
 H     -1.809564  -1.018625   2.335696
 C     -3.755488  -0.129885   2.536067
 H     -3.999487  -0.780750   3.386737
 C     -4.703658   0.830955   2.066990
 C     -4.302389   1.646490   0.962802
 H     -4.981124   2.411966   0.562191
 C     -3.039126   1.500782   0.379120
 H     -2.769804   2.168724  -0.454643
 C      1.005861  -2.421412  -1.471610
 H      0.548407  -3.063463  -0.692344
 C      2.608555  -0.510557  -1.545601
 H      3.364361  -0.949088  -2.232811
 H      1.842158  -0.022879  -2.189454
 C      3.239392   0.550800  -0.683839
 C      3.122656   1.762953   1.304544
 C      4.208545   2.566710   0.937187
 C      4.839457   2.331981  -0.295744
 C      4.345119   1.306322  -1.114333
 C      1.761277  -3.348437  -2.439724
 H      1.077276  -4.118130  -2.849887
 H      2.203531  -2.798863  -3.295938
 H      2.572658  -3.869099  -1.892484
 C      0.783682   0.200345   2.599919
 C      0.323541  -2.054615   1.625893
 H      3.548162  -2.568021   1.141210
 H      2.694293  -2.168195  -0.346776
 O      3.917333  -3.253934   0.417155
 C      5.284391  -2.958871   0.174032
 H      5.524971  -3.242940  -0.877915
 H      5.479760  -1.860189   0.254572
 H      4.806911   1.083956  -2.088159
 H      4.553262   3.358739   1.618188
 H      2.612576   1.913008   2.266763
 C      6.224034  -3.711848   1.120950
 H      6.055775  -4.807101   1.046319
 H      7.289253  -3.507944   0.878202
 H      6.050785  -3.412779   2.176740
 H      5.703575   2.935629  -0.613166
```

```
 O      2.969252  -1.518312   1.932831
 C      3.073433  -1.881952   3.288941
 C      3.700239  -0.792928   4.170594
 H      3.710958  -2.802285   3.368067
 H      2.082569  -2.170638   3.725307
 H      3.836420  -1.156829   5.212898
 H      4.695188  -0.494499   3.776640
 H      3.059635   0.111986   4.208416
 N     -5.942919   0.972266   2.656303
 C     -6.884581   1.961448   2.150592
 C     -6.315619   0.129282   3.783927
 H     -7.156040   1.775917   1.086402
 H     -7.813115   1.919731   2.748370
 H     -6.481670   2.997537   2.219408
 H     -6.311247  -0.952304   3.517924
 H     -5.631435   0.264934   4.652193
 H     -7.336973   0.392327   4.113907
 N      0.946373   6.075980  -1.541279
 C      1.003165   7.097742  -0.505735
 C      1.257062   6.424735  -2.919875
 H      1.759165   6.861336   0.277703
 H      1.281452   8.064798  -0.962715
 H      0.022174   7.232853   0.004262
 H      2.132809   5.856926  -3.309800
 H      0.400065   6.230829  -3.604732
 H      1.499137   7.501477  -2.978449
---------------------------------------
Mn19/TS-i
Frequencies, energies and thermodynamic properties:
 Lowest Vibrational Mode (1/cm) =-739.4014
 2nd Lowest Vibrational Mode (1/cm) =                     16.9236
 E(RB-P86) (a.u.) =                                     -4675.25455352
 Thermal correction to Enthalpy (a.u.) =                 0.788557
 Thermal correction to Gibbs Free Energy (a.u.) =        0.650721
 Total Entropy (cal/Kmol) =                              290.099
 E(RPBE1PBE) (a.u.) =                                   -4674.56378390
Optimised cartesian coordinates (Angstrom):
 Fe2.105447  -1.911257   1.971968
 Mn-1.113429  -0.919306  -1.551229
 P      0.204216   0.288239  -0.128489
 O     -0.400978   0.844475  -3.810752
 O      0.975462  -2.816997  -2.386693
 N     -1.786864  -2.074846   0.073970
 N     -2.845780   0.101212  -1.057791
 C      0.597821  -0.631155   1.413651
 C      0.052388  -1.935110   1.777267
 C      0.442780  -2.193101   3.144314
 H      0.209606  -3.099164   3.717979
 C      1.224418  -1.084373   3.623371
 H      1.683836  -1.000635   4.617647
 C      1.330566  -0.123839   2.559556
 H      1.865845   0.833238   2.606694
 C      3.103511  -2.695931   0.347889
 H      2.725260  -2.722408  -0.681890
 C      2.893100  -3.709687   1.347167
 H      2.333975  -4.645151   1.210411
 C      3.524122  -3.271963   2.566491
 H      3.526207  -3.812318   3.522719
 C      4.128240  -1.987210   2.318156
 H      4.671100  -1.377508   3.052997
 C      3.865911  -1.629259   0.948202
 H      4.169369  -0.699146   0.450103
 C     -0.611024   1.826407   0.498518
 C     -0.818219   2.897615  -0.405273
 H     -0.445223   2.819856  -1.440067
 C     -1.476376   4.070761  -0.023501
 H     -1.598325   4.870589  -0.766721
 C     -1.983423   4.234180   1.304320
 C     -1.784732   3.147347   2.209147
 H     -2.149132   3.212153   3.243527
 C     -1.117238   1.981463   1.808154
 H     -0.979495   1.179574   2.550251
 C      1.822760   0.948917  -0.730637
 C      2.503710   0.378371  -1.829868
 H      2.058889  -0.464159  -2.377743
 C      3.751160   0.851618  -2.254843
 H      4.227071   0.368501  -3.119065
 C      4.394731   1.941751  -1.591590
 C      3.702031   2.520758  -0.482220
 H      4.137640   3.369175   0.063136
 C      2.454379   2.035293  -0.076690
 H      1.952103   2.531150   0.769261
 C     -0.798670  -2.834194   0.883431
 H     -0.124707  -3.307111   0.140030
 C     -2.739249  -1.323879   0.899607
```

H   -3.463311 -1.983029   1.433108
H   -2.212255 -0.750286   1.703842
C   -3.489268 -0.325087   0.064773
C   -3.439828   1.051302 -1.824808
C   -4.688705   1.601941 -1.519375
C   -5.364799   1.153515 -0.371352
C   -4.753097   0.177706   0.426582
C   -1.458883 -3.983188   1.672811
H   -0.690465 -4.652493   2.110230
H   -2.096935 -3.616422   2.503335
H   -2.084636 -4.583680   0.982306
C   -0.693789   0.135561 -2.911765
C   0.173136 -2.036018 -2.005076
H   -2.167483 -1.583038 -2.728889
H   -2.239462 -2.270245 -2.225448
H   -2.434454 -2.949861 -0.639640
O   -2.901150 -3.708914 -1.471301
C   -4.299457 -3.634990 -1.576364
H   -4.793841 -3.603568 -0.567501
H   -4.632311 -2.691501 -2.094799
H   -5.244619 -0.204347   1.333901
H   -5.121455   2.366047 -2.181557
H   -2.888433   1.370288 -2.721204
C   -4.859360 -4.831289 -2.353642
H   -4.591122 -5.783726 -1.848115
H   -5.966529 -4.781968 -2.438666
H   -4.434929 -4.864535 -3.379978
H   -6.353803   1.556825 -0.104615
N   5.619961   2.419590 -2.006416
C   6.248990   3.528522 -1.302023
C   6.290203   1.816214 -3.150280
H   6.437296   3.290304 -0.230588
H   7.222362   3.754778 -1.773653
H   5.629853   4.453709 -1.337830
H   7.245723   2.340880 -3.331766
H   6.518943   0.739233 -2.981999
H   5.679715   1.889778 -4.078703
N   -2.633423   5.388849   1.691266
C   -3.137682   5.521150   3.050661
C   -2.816831   6.478266   0.742624
H   -2.324122   5.450471   3.808297
H   -3.622044   6.507807   3.165684
H   -3.893155   4.740041   3.295580
H   -3.350720   7.308279   1.239951
H   -1.846244   6.874814   0.366660
H   -3.418627   6.168097 -0.141868

Mn19/TS-ii_si
Frequencies, energies and thermodynamic properties:
Lowest Vibrational Mode (1/cm) =-209.2649
2nd Lowest Vibrational Mode (1/cm) =   15.3548
E(RB-P86) (a.u.) =   -4943.05273545
Thermal correction to Enthalpy (a.u.) =   0.861770
Thermal correction to Gibbs Free Energy (a.u.) =   0.716564
Total Entropy (cal/Kmol) =   305.611
E(RPBE1PBE) (a.u.) =   -4942.34811373
Optimised cartesian coordinates (Angstrom):
Fe-1.623446 -2.287136 -2.462104
Mn1.374949   0.210245   0.416262
P   -0.865651   0.194751   0.018695
O   1.177582   0.414843   3.343610
O   1.775017 -2.688593   0.701134
N   1.777182   0.254554 -1.655087
N   1.615350   2.225006   0.133156
C   -1.252248 -0.423837 -1.678065
C   -0.259577 -0.767133 -2.695324
C   -0.973976 -1.008321 -3.929274
H   -0.525309 -1.305307 -4.885571
C   -2.381192 -0.834286 -3.691419
H   -3.181290 -0.974865 -4.430714
C   -2.556974 -0.481700 -2.309822
H   -3.515745 -0.284402 -1.813312
C   -0.958833 -3.791755 -1.222114
H   -0.213017 -3.675578 -0.424987
C   -0.678482 -4.111273 -2.597434
H   0.315351 -4.290673 -3.029438
C   -1.928036 -4.131667 -3.314770
H   -2.053168 -4.324013 -4.388870
C   -2.981862 -3.827715 -2.380159
H   -4.051063 -3.747209 -2.618343
C   -2.383481 -3.614872 -1.087884
H   -2.910807 -3.339715 -0.165181
C   -1.653649   1.873034   0.022215
C   -1.773705   2.551763   1.260149
H   -1.467634   2.045047   2.190815

C	-2.281180	3.852337	1.346877
H	-2.359105	4.321573	2.337314
C	-2.688924	4.565133	0.175704
C	-2.549903	3.886752	-1.072633
H	-2.844751	4.380559	-2.008822
C	-2.046116	2.578878	-1.136729
H	-1.974151	2.098053	-2.125178
C	-2.002396	-0.743220	1.137182
C	-1.522442	-1.785015	1.962158
H	-0.450351	-2.029860	1.962967
C	-2.376025	-2.531196	2.784603
H	-1.942500	-3.326859	3.405839
C	-3.779699	-2.265823	2.827871
C	-4.262024	-1.204728	1.998841
H	-5.329210	-0.943248	1.996557
C	-3.390888	-0.470375	1.186217
H	-3.808011	0.351231	0.581808
C	1.251465	-0.857426	-2.514523
H	1.479520	-1.774865	-1.936317
C	1.528859	1.602265	-2.209204
H	2.141390	1.812372	-3.112616
H	0.462405	1.665690	-2.523554
C	1.773616	2.646723	-1.155735
C	1.737909	3.151766	1.122384
H	1.598830	2.785459	2.149566
C	2.032213	4.497397	0.876946
H	2.118896	5.194030	1.723868
C	2.218819	4.925478	-0.448649
C	2.087237	3.978670	-1.475239
H	2.220141	4.262490	-2.530272
C	2.005024	-0.958504	-3.852227
H	1.734883	-1.896241	-4.377475
H	1.778383	-0.112974	-4.533402
H	3.098461	-0.980256	-3.669024
C	1.236150	0.331635	2.160579
C	1.560031	-1.529457	0.562697
H	2.828006	0.146433	-1.620722
H	3.013659	0.177233	0.583741
C	4.623639	0.255144	-0.079996
C	5.070476	-0.974391	0.661878
C	5.027923	1.443076	0.824468
C	5.440654	-0.630598	1.981547
C	5.207784	-2.286386	0.179418
C	5.186016	0.844371	2.239341
H	6.012845	1.795305	0.439034
C	5.951841	-1.615619	2.842407
C	5.722486	-3.269817	1.042683
H	4.923199	-2.526269	-0.857703
H	5.989558	1.320786	2.838287
C	6.089895	-2.935118	2.364491
H	6.249620	-1.364733	3.873922
H	5.846839	-4.305801	0.688293
H	6.496458	-3.714736	3.028948
O	4.476376	0.328335	-1.329988
H	4.325823	2.295355	0.756479
H	4.241447	0.959745	2.818276
H	2.462238	5.974097	-0.677895
N	-3.192605	5.849663	0.251123
C	-3.315600	6.512051	1.541490
C	-3.584900	6.550518	-0.962489
H	-2.331049	6.633662	2.048890
H	-3.746432	7.519213	1.394691
H	-3.984373	5.954828	2.236467
H	-2.732097	6.679374	-1.667997
H	-4.395480	6.018122	-1.510852
H	-3.960059	7.556064	-0.698566
N	-4.628718	-2.992300	3.637576
C	-6.054096	-2.695068	3.656239
C	-4.103393	-4.065969	4.469371
H	-6.523837	-2.828688	2.655046
H	-6.559792	-3.379878	4.360962
H	-6.260534	-1.652120	3.988155
H	-4.932395	-4.525906	5.037276
H	-3.619254	-4.866207	3.864400
H	-3.350771	-3.697664	5.203128

Mn19/TS-ii_re

Frequencies, energies and thermodynamic properties:

```
 Lowest Vibrational Mode (1/cm) =-218.9857
 2nd Lowest Vibrational Mode (1/cm) =                     13.7734
 E(RB-P86) (a.u.) =                                     -4943.05321087
 Thermal correction to Enthalpy (a.u.) =                  0.861762
 Thermal correction to Gibbs Free Energy (a.u.) =         0.715784
 Total Entropy (cal/Kmol) =                               307.236
 E(RPBE1PBE) (a.u.) =                                   -4942.34938818
```

Optimised cartesian coordinates (Angstrom):

Fe-2.894728	-1.643723	-2.093884	
Mn1.137669	-0.747800	0.445452	
P	-0.787586	0.382839	-0.005452
O	1.227018	-0.067513	3.306119
O	-0.047618	-3.339053	1.164500
N	1.375814	-1.214297	-1.597316
N	2.401148	0.760005	-0.131422
C	-1.547577	-0.173060	-1.594706
C	-0.950642	-1.131994	-2.522865
C	-1.757717	-1.129144	-3.722572
H	-1.592579	-1.751598	-4.610998
C	-2.843006	-0.202268	-3.548291
H	-3.639627	0.000422	-4.276849
C	-2.721667	0.381885	-2.241273
H	-3.398645	1.126946	-1.804056
C	-4.148690	-2.159170	-0.547582
H	-4.386598	-1.524179	0.315807
C	-3.048087	-3.086789	-0.631816
H	-2.302768	-3.281340	0.150338
C	-3.073943	-3.689040	-1.939052
H	-2.359314	-4.429204	-2.323772
C	-4.188701	-3.134425	-2.664278
H	-4.468893	-3.372801	-3.699181
C	-4.854519	-2.191079	-1.802194
H	-5.730938	-1.583967	-2.066236
C	-0.563159	2.201778	-0.285997
C	-0.234138	3.014766	0.826797
H	-0.190511	2.567464	1.834008
C	0.029745	4.382004	0.694320
H	0.268711	4.961310	1.596835
C	-0.005290	5.019997	-0.585432
C	-0.318948	4.195733	-1.708019
H	-0.360780	4.625687	-2.718246
C	-0.588488	2.827846	-1.552139
H	-0.839493	2.242600	-2.450956
C	-2.178818	0.370340	1.213778
C	-2.276655	-0.634203	2.202392
H	-1.499540	-1.409422	2.269498
C	-3.345353	-0.681684	3.106590
H	-3.364167	-1.485743	3.854970
C	-4.388969	0.294072	3.069674
C	-4.284121	1.314264	2.072660
H	-5.048704	2.099961	1.999841
C	-3.206105	1.344203	1.180803
H	-3.159504	2.162999	0.444791
C	0.291160	-1.984732	-2.291417
H	0.032572	-2.789262	-1.574664
C	1.844312	-0.034874	-2.354880
H	2.422360	-0.311916	-3.263146
H	0.955115	0.537127	-2.704619
C	2.662728	0.861308	-1.466655
C	3.057226	1.602678	0.710084
H	2.829260	1.493925	1.779964
C	3.977008	2.559753	0.268054
H	4.470307	3.213768	1.002095
C	4.255688	2.655862	-1.105687
C	3.589365	1.784719	-1.980101
H	3.772318	1.817863	-3.064877
C	0.798199	-2.661559	-3.576853
H	0.042276	-3.374472	-3.962322
H	1.017126	-1.932851	-4.384002
H	1.721332	-3.236132	-3.359420
C	1.179450	-0.328654	2.149370
C	0.373259	-2.271146	0.853651
H	2.206097	-1.866077	-1.521930
H	2.533320	-1.605122	0.649168
C	3.806540	-2.614313	0.031354
C	4.866609	-1.844856	0.768397
C	3.446446	-3.798499	0.959946
C	4.951574	-2.302928	2.103097
C	5.742204	-0.865047	0.271443
C	3.894964	-3.360388	2.371272
C	5.923544	-1.767721	2.963972
C	6.715858	-0.332958	1.135801
H	5.664498	-0.538091	-0.777893
C	6.803219	-0.781084	2.472007
H	6.007915	-2.116593	4.006483
H	7.420371	0.431356	0.770129
H	7.574691	-0.359756	3.136786
O	3.666507	-2.620029	-1.222285
H	4.271890	-4.194057	2.999190
H	3.043305	-2.901577	2.923463
H	2.386141	-4.102224	0.884432
H	4.067285	-4.653597	0.604795

```
 H      4.979992   3.391209  -1.488031
 N     -5.443362   0.259874   3.959244
 C     -5.518949  -0.793958   4.961467
 C     -6.492032   1.267765   3.889090
 H     -4.641092  -0.783617   5.646784
 H     -6.427504  -0.650411   5.574056
 H     -5.574295  -1.807037   4.501556
 H     -7.239141   1.072405   4.679610
 H     -6.094938   2.296788   4.043523
 H     -7.021682   1.255144   2.909154
 N      0.253159   6.369979  -0.726605
 C      0.575091   7.178762   0.440122
 C      0.217137   6.984483  -2.045373
 H      1.499066   6.827645   0.954289
 H      0.741607   8.224618   0.123925
 H     -0.247609   7.177926   1.191150
 H      0.443385   8.062287  -1.952299
 H      0.966502   6.537500  -2.738394
 H     -0.783191   6.887258  -2.526005
---------------------------------------
Mn19/TS-iii
Frequencies, energies and thermodynamic properties:
 Lowest Vibrational Mode (1/cm) =-652.0119
 2nd Lowest Vibrational Mode (1/cm) =                      11.2051
 E(RB-P86) (a.u.) =                                      -4520.30902316
 Thermal correction to Enthalpy (a.u.) =                   0.706648
 Thermal correction to Gibbs Free Energy (a.u.) =          0.581003
 Total Entropy (cal/Kmol) =                                264.442
 E(RPBE1PBE) (a.u.) =                                    -4519.61209082
Optimised cartesian coordinates (Angstrom):
 Fe-2.485931  -1.662992  -1.585229
 Mn0.644674  -1.210104   2.077280
 P      0.070943   0.050581   0.261858
 O      0.734599   1.042174   3.988853
 O     -2.104310  -1.758459   2.966593
 N      0.862146  -2.907677   0.871850
 N      2.678239  -1.130966   1.670712
 C     -0.595929  -1.022551  -1.079808
 C     -0.665111  -2.483252  -1.052942
 C     -1.059483  -2.910047  -2.376277
 H     -1.222038  -3.948592  -2.692017
 C     -1.246247  -1.751891  -3.209035
 H     -1.569531  -1.755770  -4.258974
 C     -0.969856  -0.587562  -2.413629
 H     -1.024197   0.453551  -2.757164
 C     -3.814936  -1.514597  -0.014927
 H     -3.542442  -1.435220   1.045056
 C     -4.026074  -2.739624  -0.739803
 H     -3.952408  -3.755059  -0.327572
 C     -4.328657  -2.402213  -2.107916
 H     -4.521996  -3.114527  -2.921386
 C     -4.307457  -0.965779  -2.226525
 H     -4.481084  -0.392593  -3.147323
 C     -3.986994  -0.417002  -0.934161
 H     -3.868301   0.647155  -0.691428
 C      1.492343   0.909838  -0.557247
 C      2.078627   2.017361   0.103159
 H      1.647311   2.377252   1.052296
 C      3.191118   2.686531  -0.417312
 H      3.595271   3.545170   0.136265
 C      3.796672   2.267391  -1.643580
 C      3.213980   1.140193  -2.298682
 H      3.634333   0.768731  -3.243394
 C      2.095352   0.486485  -1.762059
 H      1.679928  -0.372742  -2.311762
 C     -1.140093   1.438322   0.432754
 C     -2.023510   1.518280   1.532689
 H     -1.982377   0.758318   2.325823
 C     -2.970045   2.543961   1.649993
 H     -3.625954   2.553110   2.531213
 C     -3.082153   3.564457   0.656073
 C     -2.180829   3.487449  -0.451975
 H     -2.207526   4.249064  -1.243448
 C     -1.240752   2.455609  -0.547681
 H     -0.554530   2.454041  -1.409756
 C     -0.312681  -3.385111   0.140393
 H     -1.157776  -3.335611   0.858616
 C      2.085378  -2.880099   0.095543
 H      2.501735  -3.898029  -0.113019
 H      1.943127  -2.417247  -0.922784
 C      3.140053  -2.059373   0.786445
 C      3.576917  -0.324423   2.292014
 C      4.955003  -0.407878   2.068867
 C      5.438441  -1.371740   1.166365
 C      4.515335  -2.206065   0.523000
```

```
 C     -0.186900  -4.864295  -0.287087
 H     -1.149034  -5.250147  -0.682728
 H      0.581653  -5.012454  -1.074204
 H      0.093892  -5.480802   0.591045
 C      0.697608   0.140820   3.223383
 C     -1.022612  -1.520736   2.545585
 H      1.030472  -2.807092   2.442393
 H      1.124668  -2.357087   3.249119
 H      3.166370   0.409032   3.000917
 H      5.633769   0.272346   2.604019
 H      4.846545  -2.978333  -0.187762
 H      6.517355  -1.472717   0.971806
 N      4.893775   2.922899  -2.167388
 C      5.460647   4.069888  -1.472658
 C      5.485839   2.469113  -3.417307
 H      5.822844   3.806706  -0.452587
 H      6.322258   4.454974  -2.047771
 H      4.725857   4.900102  -1.364048
 H      5.856889   1.420829  -3.349855
 H      4.763963   2.515174  -4.264641
 H      6.345403   3.116141  -3.670132
 N     -4.008412   4.581103   0.764288
 C     -4.911373   4.627276   1.905956
 C     -4.096486   5.603645  -0.268905
 H     -5.573738   5.506783   1.811575
 H     -5.555281   3.720481   1.967894
 H     -4.362215   4.716300   2.870765
 H     -3.144823   6.171783  -0.378022
 H     -4.347673   5.173123  -1.265094
 H     -4.890883   6.324026  -0.001870
---------------------------------------
Mn20/i
Frequencies, energies and thermodynamic properties:
 Lowest Vibrational Mode (1/cm) =                         22.3505
 2nd Lowest Vibrational Mode (1/cm) =                     29.9047
 E(RB-P86) (a.u.) =                                     -4362.05424740
 Thermal correction to Enthalpy (a.u.) =                  0.577066
 Thermal correction to Gibbs Free Energy (a.u.) =         0.467333
 Total Entropy (cal/Kmol) =                               230.953
 E(RPBE1PBE) (a.u.) =                                   -4361.37750825
Optimised cartesian coordinates (Angstrom):
 Fe2.794087  -1.232940   1.019437
 Mn-1.011639  -0.620849  -1.776036
 P     -0.142402   0.512570  -0.105026
 O     -1.712442   1.687606  -3.503747
 O      1.443229  -0.886868  -3.366763
 N     -0.883211  -2.300790  -0.896184
 N     -2.973555  -0.813545  -1.162060
 C      0.825777  -0.641293   0.966392
 C      0.929877  -2.084082   0.770778
 C      1.593821  -2.628419   1.931841
 H      1.846661  -3.684665   2.091097
 C      1.905363  -1.552013   2.835478
 H      2.429016  -1.646964   3.796637
 C      1.441459  -0.327176   2.242403
 H      1.530474   0.674188   2.683234
 C      3.761296  -0.894256  -0.768663
 H      3.270446  -0.755909  -1.741019
 C      4.172509  -2.152275  -0.203287
 H      4.060007  -3.138908  -0.672747
 C      4.736631  -1.894615   1.097742
 H      5.125408  -2.649652   1.794283
 C      4.676474  -0.474322   1.334815
 H      5.011022   0.042114   2.244685
 C      4.071304   0.143796   0.183371
 H      3.858150   1.213413   0.057608
 C     -1.437587   1.146997   1.052473
 C     -2.177279   2.298880   0.684962
 H     -1.916622   2.843480  -0.237711
 C     -3.235299   2.770640   1.468302
 H     -3.783575   3.676210   1.160594
 C     -3.618055   2.095185   2.662342
 C     -2.890415   0.928357   3.021364
 H     -3.164317   0.381530   3.938617
 C     -1.826905   0.469316   2.231003
 H     -1.282921  -0.433315   2.550310
 C      0.926471   1.997281  -0.349244
 C      1.562142   2.245124  -1.587714
 H      1.397860   1.562065  -2.433733
 C      2.411808   3.343693  -1.767675
 H      2.889809   3.510429  -2.746836
 C      2.662638   4.256886  -0.705933
 C      2.018527   4.012143   0.540154
 H      2.185063   4.706286   1.380215
 C      1.170171   2.911919   0.705531
```

H   0.676404   2.768906   1.680045
C   0.410306 -2.852079 -0.455402
H   1.116489 -2.654242 -1.286377
C   -2.002727 -2.730118 -0.080102
H   -2.244602 -3.813265 -0.211647
H   -1.791171 -2.618728   1.017956
C   -3.218785 -1.916620 -0.397829
C   -4.024917 -0.005010 -1.475582
H   -3.793152   0.877493 -2.089050
C   -5.332259 -0.267441 -1.062338
H   -6.140216   0.420482 -1.352442
C   -5.587836 -1.413608 -0.283449
C   -4.511977 -2.245594   0.049151
H   -4.658634 -3.152448   0.655702
C   0.408981 -4.375822 -0.228963
H   1.445410 -4.744815 -0.090189
H   -0.175081 -4.677915   0.664469
H   -0.015501 -4.890484 -1.115282
C   -1.424298   0.768324 -2.810504
C   0.488728 -0.766719 -2.668428
H   -6.608118 -1.650620   0.055089
N   -4.627628   2.586217   3.465630
H   -5.289126   3.245328   3.051186
H   -5.045814   1.950916   4.147713
N   3.454415   5.368742 -0.892163
H   4.084554   5.383325 -1.695749
H   3.810698   5.852148 -0.066139

Mn20/ii
Frequencies, energies and thermodynamic properties:
Lowest Vibrational Mode (1/cm) = 17.3037
2nd Lowest Vibrational Mode (1/cm) = 23.0506
E(RB-P86) (a.u.) = -4516.98963643
Thermal correction to Enthalpy (a.u.) = 0.661827
Thermal correction to Gibbs Free Energy (a.u.) = 0.537076
Total Entropy (cal/Kmol) = 262.561
E(RPBE1PBE) (a.u.) = -4516.32149106
Optimised cartesian coordinates (Angstrom):
Fe-2.604158 -1.594860 -1.368756
Mn1.213276 -0.389366   1.213374
P   -0.357952   0.743156   0.168806
O   1.316854   1.308971   3.639449
O   -0.580206 -2.168273   2.711559
N   1.543672 -1.597047 -0.256076
N   2.911939   0.493847   0.437731
C   -1.031430 -0.283529 -1.209364
C   -0.551777 -1.609529 -1.588044
C   -1.203548 -1.963583 -2.826997
H   -1.076737 -2.908257 -3.371307
C   -2.076999 -0.888296 -3.216671
H   -2.724038 -0.871327 -4.104322
C   -1.980342   0.144392 -2.221232
H   -2.525322   1.097272 -2.229198
C   -3.228311 -2.278987   0.471910
H   -2.634819 -2.254963   1.395068
C   -3.241972 -3.346978 -0.492632
H   -2.668386 -4.281573 -0.429305
C   -4.121047 -2.965779 -1.569029
H   -4.332076 -3.556458 -2.470677
C   -4.654182 -1.661391 -1.266633
H   -5.342026 -1.083822 -1.898895
C   -4.100682 -1.235294 -0.007170
H   -4.286697 -0.275418   0.492216
C   0.331086   2.213544 -0.712731
C   0.668828   3.364003   0.043728
H   0.446319   3.392448   1.123300
C   1.278235   4.475244 -0.546375
H   1.518390   5.358754   0.067642
C   1.595317   4.482843 -1.935234
C   1.275236   3.322094 -2.690836
H   1.509550   3.296844 -3.767729
C   0.658502   2.216474 -2.088585
H   0.417200   1.342091 -2.712824
C   -1.817633   1.457119   1.042170
C   -2.223564   0.981996   2.310663
H   -1.647515   0.190338   2.810864
C   -3.357666   1.491760   2.954490
H   -3.644811   1.101558   3.944550
C   -4.143592   2.514594   2.353732
C   -3.732729   2.998173   1.078776
H   -4.314161   3.797025   0.589975
C   -2.595821   2.482003   0.447979
H   -2.300919   2.897048 -0.529058
C   0.464741 -2.449156 -0.803497
H   -0.061057 -2.857109   0.083076

```
 C      2.488725  -1.150823  -1.270557
 H      3.129886  -1.976657  -1.663047
 H      1.961323  -0.743278  -2.173845
 C      3.361898  -0.064251  -0.722431
 C      3.636151   1.507160   0.988490
 C      4.818682   1.986690   0.421806
 C      5.290145   1.404576  -0.771067
 C      4.548843   0.365050  -1.345339
 C      0.977414  -3.659319  -1.607678
 H      0.149504  -4.368482  -1.810684
 H      1.418970  -3.371066  -2.583778
 H      1.746323  -4.188479  -1.008955
 C      1.269430   0.631616   2.667559
 C      0.092648  -1.438603   2.060122
 H      2.479322  -2.824853   0.793944
 O      2.929777  -3.592171   1.256870
 C      4.331012  -3.358373   1.255975
 H      4.577976  -2.352418   1.680152
 H      4.749996  -3.370183   0.216700
 H      4.876994  -0.122645  -2.275886
 H      5.363145   2.805187   0.915304
 H      3.243095   1.934861   1.921948
 C      5.022047  -4.435718   2.085038
 H      6.121416  -4.285049   2.097503
 H      4.655961  -4.418056   3.133193
 H      4.814691  -5.444339   1.669084
 H      6.221289   1.756754  -1.240889
 N      2.148865   5.595860  -2.532300
 H      2.596407   5.479726  -3.443103
 H      2.594830   6.291152  -1.931496
 N     -5.228875   3.056426   3.004904
 H     -5.633079   2.538863   3.786952
 H     -5.901937   3.597518   2.459924
---------------------------------------
Mn20/iii
Frequencies, energies and thermodynamic properties:
 Lowest Vibrational Mode (1/cm) =                        17.0453
 2nd Lowest Vibrational Mode (1/cm) =                    25.6264
 E(RB-P86) (a.u.) =                                     -4518.16197089
 Thermal correction to Enthalpy (a.u.) =                 0.677762
 Thermal correction to Gibbs Free Energy (a.u.) =        0.553718
 Total Entropy (cal/Kmol) =                              261.073
 E(RPBE1PBE) (a.u.) =                                   -4517.49081327
Optimised cartesian coordinates (Angstrom):
 Fe-2.578475  -1.669892  -1.323898
 Mn1.239245  -0.367964   1.421849
 P     -0.326742   0.698562   0.145514
 O      0.916462   1.653710   3.558889
 O     -0.646941  -2.147641   2.813775
 N      1.603657  -1.688692  -0.157277
 N      2.840531   0.578169   0.511488
 C     -0.994032  -0.365657  -1.193056
 C     -0.521100  -1.709481  -1.505659
 C     -1.156628  -2.103867  -2.741877
 H     -1.030901  -3.069696  -3.247684
 C     -2.016688  -1.039581  -3.187039
 H     -2.651067  -1.053877  -4.083807
 C     -1.928212   0.030617  -2.231666
 H     -2.466019   0.986092  -2.283332
 C     -3.255214  -2.273005   0.527673
 H     -2.690924  -2.206652   1.466443
 C     -3.237001  -3.383254  -0.387333
 H     -2.662516  -4.311412  -0.265360
 C     -4.084446  -3.053839  -1.505399
 H     -4.266394  -3.684794  -2.385867
 C     -4.630008  -1.739239  -1.277701
 H     -5.300551  -1.193656  -1.955344
 C     -4.115570  -1.254950  -0.022823
 H     -4.319906  -0.275275   0.428666
 C      0.347443   2.160608  -0.768297
 C      0.692874   3.318326  -0.026745
 H      0.507715   3.348044   1.059708
 C      1.252295   4.442802  -0.641701
 H      1.500497   5.330553  -0.037147
 C      1.508745   4.457848  -2.042317
 C      1.171241   3.294378  -2.785630
 H      1.352539   3.275429  -3.872833
 C      0.606180   2.175149  -2.157688
 H      0.348169   1.300530  -2.774929
 C     -1.812216   1.436688   0.966322
 C     -2.275919   0.983360   2.222863
 H     -1.729389   0.197466   2.762632
 C     -3.434545   1.506454   2.810307
 H     -3.764151   1.131608   3.793034
 C     -4.190893   2.521727   2.161765
```

```
 C     -3.724453   2.982804   0.898034
 H     -4.282260   3.774650   0.371602
 C     -2.562868   2.453947   0.325084
 H     -2.226531   2.853068  -0.644992
 C      0.493923  -2.511220  -0.686146
 H     -0.035256  -2.900507   0.208299
 C      2.369451  -1.044753  -1.220936
 H      2.991186  -1.762899  -1.809715
 H      1.711281  -0.546143  -1.984977
 C      3.256948   0.030060  -0.664121
 C      3.562365   1.595558   1.047828
 C      4.722472   2.099309   0.451338
 C      5.165691   1.529120  -0.755330
 C      4.421086   0.482814  -1.314603
 C      0.997019  -3.747544  -1.464728
 H      0.161551  -4.443369  -1.685397
 H      1.469407  -3.475723  -2.431703
 H      1.742183  -4.287931  -0.846914
 C      1.059163   0.842033   2.712034
 C      0.064119  -1.417512   2.213760
 H      2.496984  -0.862671   2.505412
 H      2.444095  -1.535321   2.021690
 H      2.562654  -2.747185   0.587098
 O      3.121455  -3.413984   1.175741
 C      4.495905  -3.281095   0.867639
 H      4.699332  -3.501281  -0.212996
 H      4.859578  -2.233789   1.039204
 H      4.730752   0.005213  -2.256444
 H      5.268114   2.922919   0.934659
 H      3.190954   2.011603   1.995760
 C      5.316255  -4.237814   1.730145
 H      4.997942  -5.287974   1.558297
 H      6.399298  -4.160798   1.498202
 H      5.176002  -4.013918   2.808971
 H      6.079200   1.894287  -1.249544
 N      2.107523   5.545308  -2.644380
 H      2.034259   5.630400  -3.659675
 H      2.085854   6.437972  -2.148227
 N     -5.300581   3.077978   2.758157
 H     -5.947839   3.610541   2.174830
 H     -5.741506   2.571583   3.527646
---------------------------------------
Mn20/iv
Frequencies, energies and thermodynamic properties:
 Lowest Vibrational Mode (1/cm) =                       17.5173
 2nd Lowest Vibrational Mode (1/cm) =                   23.1560
 E(RB-P86) (a.u.) =                                    -4518.18893518
 Thermal correction to Enthalpy (a.u.) =                0.682463
 Thermal correction to Gibbs Free Energy (a.u.) =       0.558402
 Total Entropy (cal/Kmol) =                             261.109
 E(RPBE1PBE) (a.u.) =                                  -4517.51656560
Optimised cartesian coordinates (Angstrom):
 Fe-2.695728  -1.501274  -1.364142
 Mn1.312833  -0.500578   1.219683
 P     -0.317761   0.711361   0.159484
 O      1.326406   1.203536   3.614061
 O     -0.384601  -2.440866   2.637094
 N      1.544622  -1.708599  -0.549543
 N      2.884346   0.454727   0.303228
 C     -1.101099  -0.212091  -1.234633
 C     -0.671693  -1.525794  -1.712659
 C     -1.403427  -1.801376  -2.929267
 H     -1.328602  -2.714306  -3.533331
 C     -2.281260  -0.695919  -3.201995
 H     -2.983899  -0.624599  -4.043235
 C     -2.103778   0.278574  -2.161318
 H     -2.633801   1.236229  -2.079380
 C     -3.205078  -2.276773   0.473728
 H     -2.550923  -2.303790   1.355142
 C     -3.291039  -3.292614  -0.542720
 H     -2.723111  -4.232523  -0.566411
 C     -4.235951  -2.850831  -1.536811
 H     -4.510273  -3.391665  -2.452561
 C     -4.737367  -1.562185  -1.131568
 H     -5.460159  -0.948337  -1.685753
 C     -4.099117  -1.205866   0.108930
 H     -4.243065  -0.272778   0.668955
 C      0.295033   2.252157  -0.676796
 C      0.754891   3.312366   0.144676
 H      0.670252   3.227894   1.241033
 C      1.303761   4.478994  -0.398809
 H      1.641146   5.288519   0.269343
 C      1.438635   4.635772  -1.807047
 C      0.991601   3.569790  -2.632179
 H      1.079516   3.660101  -3.727530
```

```
 C      0.435397   2.408072  -2.074311
 H      0.089124   1.614407  -2.755068
 C     -1.744019   1.378085   1.136275
 C     -2.100866   0.809207   2.380394
 H     -1.510426  -0.024223   2.788084
 C     -3.198054   1.278057   3.114556
 H     -3.446356   0.813805   4.083121
 C     -3.995456   2.352677   2.632813
 C     -3.632101   2.934220   1.385459
 H     -4.221709   3.778122   0.990739
 C     -2.531938   2.456359   0.663734
 H     -2.270956   2.949046  -0.286742
 C      0.346998  -2.454594  -1.064607
 H     -0.109173  -2.889962  -0.153056
 C      2.270851  -0.943955  -1.585245
 H      2.801799  -1.596326  -2.312380
 H      1.526258  -0.357215  -2.168793
 C      3.232036   0.020984  -0.943327
 C      3.674740   1.391001   0.891970
 C      4.827151   1.907352   0.288763
 C      5.199625   1.441749  -0.983394
 C      4.383573   0.482424  -1.602517
 C      0.753614  -3.622910  -1.979264
 H     -0.121666  -4.266338  -2.198356
 H      1.171175  -3.279034  -2.947666
 H      1.511406  -4.253069  -1.470816
 C      1.312835   0.519743   2.643450
 C      0.251589  -1.639513   2.033472
 H      2.500409  -1.359535   1.878050
 H      2.861411  -2.725982   1.539282
 H      2.190968  -2.436386  -0.176981
 O      3.146547  -3.558529   1.043144
 C      4.576568  -3.587969   1.033089
 H      4.873552  -4.318867   0.249399
 H      4.994817  -2.599837   0.723278
 H      4.628622   0.086546  -2.599802
 H      5.424257   2.661488   0.822659
 H      3.367052   1.724218   1.893583
 C      5.163163  -4.002565   2.382469
 H      4.767780  -4.991527   2.695162
 H      6.270385  -4.072170   2.328154
 H      4.907599  -3.264923   3.172609
 H      6.105878   1.816715  -1.482914
 N     -5.103073   2.790626   3.328778
 H     -5.478913   3.712174   3.098905
 H     -5.174543   2.544692   4.317652
 N      2.032856   5.762660  -2.345974
 H      2.068687   6.599658  -1.760665
 H      1.853027   5.962162  -3.332019
--------------------------------------
Mn20/v
Frequencies, energies and thermodynamic properties:
 Lowest Vibrational Mode (1/cm) =                        18.6978
 2nd Lowest Vibrational Mode (1/cm) =                    29.9357
 E(RB-P86) (a.u.) =                                    -4363.25013405
 Thermal correction to Enthalpy (a.u.) =                 0.597970
 Thermal correction to Gibbs Free Energy (a.u.) =        0.487594
 Total Entropy (cal/Kmol) =                              232.306
 E(RPBE1PBE) (a.u.) =                                  -4362.57379729
Optimised cartesian coordinates (Angstrom):
 Fe2.807514  -1.122813   1.069254
 Mn-1.035319  -0.675008  -1.889743
 P     -0.189702   0.476619  -0.100301
 O     -1.674863   1.700682  -3.495378
 O      1.416406  -1.064454  -3.466591
 N     -0.821741  -2.521290  -0.803877
 N     -2.924958  -0.887906  -1.125874
 C      0.820492  -0.598527   1.014213
 C      0.985004  -2.044590   0.863918
 C      1.671480  -2.525697   2.042932
 H      1.966132  -3.564288   2.238842
 C      1.945236  -1.409458   2.906185
 H      2.479789  -1.453484   3.864698
 C      1.430121  -0.225636   2.276529
 H      1.485042   0.793207   2.681084
 C      3.746005  -0.796596  -0.735239
 H      3.242413  -0.697829  -1.706147
 C      4.203003  -2.027214  -0.143954
 H      4.118532  -3.028238  -0.588220
 C      4.772214  -1.719605   1.143618
 H      5.191123  -2.444648   1.854352
 C      4.669870  -0.296737   1.346196
 H      4.996189   0.252302   2.239734
 C      4.033694   0.273587   0.187358
 H      3.783859   1.332097   0.038725
```

```
 C     -1.464739   1.159599   1.066362
 C     -2.270444   2.234298   0.612466
 H     -2.073077   2.682974  -0.375540
 C     -3.312310   2.752470   1.389906
 H     -3.911972   3.596150   1.009945
 C     -3.613629   2.202960   2.667659
 C     -2.822891   1.111901   3.115838
 H     -3.032735   0.659885   4.099402
 C     -1.774912   0.607570   2.329780
 H     -1.181138  -0.231837   2.725126
 C      0.870866   1.969719  -0.383434
 C      1.517072   2.168447  -1.624826
 H      1.366865   1.441507  -2.436619
 C      2.352174   3.270737  -1.849934
 H      2.838484   3.397215  -2.831242
 C      2.576945   4.237926  -0.831750
 C      1.918799   4.045866   0.415670
 H      2.060997   4.785073   1.221295
 C      1.086628   2.939814   0.625766
 H      0.577743   2.839927   1.598020
 C      0.542278  -2.910676  -0.308920
 H      1.207355  -2.700302  -1.170144
 C     -1.893452  -2.637644   0.208246
 H     -2.125002  -3.691361   0.475331
 H     -1.532128  -2.143391   1.137718
 C     -3.134236  -1.923283  -0.259531
 C     -4.007890  -0.157678  -1.506418
 H     -3.811221   0.673063  -2.199284
 C     -5.309377  -0.434167  -1.072385
 H     -6.139928   0.195868  -1.424229
 C     -5.526596  -1.517165  -0.204114
 C     -4.413554  -2.269233   0.203989
 H     -4.526052  -3.125726   0.886176
 C      0.636196  -4.414966  -0.003518
 H      1.689389  -4.705781   0.180887
 H      0.044636  -4.705457   0.888517
 H      0.278072  -5.007870  -0.870436
 C     -1.406844   0.749906  -2.834130
 C      0.460814  -0.882556  -2.783266
 H     -1.043262  -3.153824  -1.584809
 H     -1.607002  -1.611482  -3.041418
 H     -6.538119  -1.771164   0.147488
 N      3.432027   5.302775  -1.031626
 H      3.356314   6.100751  -0.398315
 H      3.660639   5.555908  -1.994621
 N     -4.606801   2.747348   3.463227
 H     -5.321164   3.311391   2.998302
 H     -4.977741   2.161879   4.214380
----------------------------------------
Mn20/vi_R
Frequencies, energies and thermodynamic properties:
 Lowest Vibrational Mode (1/cm) =                          13.9750
 2nd Lowest Vibrational Mode (1/cm) =                      19.6672
 E(RB-P86) (a.u.) =                                      -4785.96487947
 Thermal correction to Enthalpy (a.u.) =                   0.750504
 Thermal correction to Gibbs Free Energy (a.u.) =          0.617811
 Total Entropy (cal/Kmol) =                                279.275
 E(RPBE1PBE) (a.u.) =                                    -4785.28304022
Optimised cartesian coordinates (Angstrom):
 Fe-2.693039  -2.139185  -1.537910
 Mn1.000518   0.263851   0.325369
 P     -1.196249   0.505281   0.367841
 O      1.405065   0.929337   3.182984
 O      1.023183  -2.571310   1.103694
 N      1.067413  -0.042937  -1.611845
 N      1.524280   2.160280  -0.294705
 C     -1.939546  -0.272776  -1.128349
 C     -1.191364  -0.897665  -2.215251
 C     -2.133771  -1.187524  -3.270907
 H     -1.899133  -1.677354  -4.224584
 C     -3.444069  -0.762927  -2.854755
 H     -4.371560  -0.870749  -3.433471
 C     -3.331608  -0.206642  -1.534116
 H     -4.155845   0.205318  -0.937667
 C     -2.047273  -3.545756  -0.177483
 H     -1.174683  -3.440424   0.479834
 C     -2.036751  -4.091668  -1.508974
 H     -1.158440  -4.484007  -2.039188
 C     -3.375995  -4.011701  -2.034945
 H     -3.696473  -4.328017  -3.036742
 C     -4.215533  -3.418430  -1.024901
 H     -5.288121  -3.203283  -1.123253
 C     -3.394515  -3.127873   0.122056
 H     -3.725797  -2.649674   1.053172
 C     -1.697786   2.276632   0.211162
```

```
 C     -1.545262   3.124667   1.337076
 H     -1.201242   2.702738   2.295960
 C     -1.830969   4.491293   1.268025
 H     -1.712035   5.121891   2.164293
 C     -2.273340   5.084325   0.050657
 C     -2.413821   4.237565  -1.082465
 H     -2.758457   4.666424  -2.037731
 C     -2.128817   2.867388  -0.999166
 H     -2.264663   2.245117  -1.897387
 C     -2.217786  -0.060194   1.797179
 C     -1.743195  -1.027372   2.713108
 H     -0.729838  -1.438677   2.603538
 C     -2.536880  -1.484876   3.771945
 H     -2.134043  -2.235230   4.471579
 C     -3.856850  -0.990281   3.965183
 C     -4.333448  -0.009776   3.049084
 H     -5.346756   0.404400   3.178633
 C     -3.528317   0.441572   1.997967
 H     -3.926905   1.218000   1.325648
 C      0.318272  -1.165791  -2.223656
 H      0.507646  -2.034123  -1.560926
 C      1.058802   1.161296  -2.439905
 H      1.681078   1.058612  -3.360750
 H      0.026195   1.397601  -2.808401
 C      1.540845   2.337228  -1.646497
 C      1.913481   3.197476   0.496556
 H      1.884037   3.020436   1.581172
 C      2.337552   4.424636  -0.018199
 H      2.643708   5.225105   0.671141
 C      2.367552   4.605132  -1.414339
 C      1.965116   3.544076  -2.233970
 H      1.972212   3.637046  -3.330498
 C      0.826943  -1.559845  -3.624367
 H      0.356232  -2.508805  -3.951986
 H      0.601756  -0.792374  -4.393130
 H      1.923596  -1.721077  -3.598038
 C      1.245074   0.663774   2.039227
 C      0.953549  -1.435112   0.769478
 H      2.657754  -0.461647  -1.697047
 H      3.191788  -0.120974   0.261555
 C      4.105998  -0.342632  -0.398975
 C      4.882615  -1.453390   0.295946
 C      5.076300   0.867934  -0.281520
 C      5.908190  -0.910215   1.101343
 C      4.690144  -2.840617   0.211289
 C      5.904134   0.606328   1.000536
 H      5.735775   0.835451  -1.176682
 C      6.747803  -1.758909   1.842292
 C      5.534282  -3.691745   0.952181
 H      3.895495  -3.250764  -0.432366
 H      6.924050   1.044116   0.969630
 C      6.554662  -3.153744   1.763252
 H      7.555297  -1.345190   2.469230
 H      5.401843  -4.784425   0.894002
 H      7.212442  -3.829669   2.333541
 O      3.674731  -0.636251  -1.693242
 H      4.551202   1.844280  -0.283611
 H      5.399227   1.046219   1.891532
 H      2.701653   5.557896  -1.852939
 N     -2.510336   6.439910  -0.034859
 H     -3.058898   6.781920  -0.825652
 H     -2.656871   6.957899   0.833222
 N     -4.656588  -1.467213   4.979015
 H     -5.482986  -0.927780   5.241158
 H     -4.214156  -1.970529   5.749513
---------------------------------------
Mn20/vi_S
Frequencies, energies and thermodynamic properties:
 Lowest Vibrational Mode (1/cm) =                         14.9818
 2nd Lowest Vibrational Mode (1/cm) =                     18.6505
 E(RB-P86) (a.u.) =                                     -4785.96519277
 Thermal correction to Enthalpy (a.u.) =                  0.750524
 Thermal correction to Gibbs Free Energy (a.u.) =         0.617647
 Total Entropy (cal/Kmol) =                               279.663
 E(RPBE1PBE) (a.u.) =                                   -4785.28310863
Optimised cartesian coordinates (Angstrom):
 Fe-3.303395  -1.668881  -1.346063
 Mn0.881515  -0.224529   0.461881
 P     -1.127717   0.678537   0.282718
 O      1.363108   0.721371   3.227084
 O     -0.008005  -2.773288   1.623200
 N      0.918022  -0.825767  -1.405959
 N      2.017554   1.291426  -0.358909
 C     -2.027313  -0.070408  -1.140805
 C     -1.471170  -1.051479  -2.067769
```

C	-2.421493	-1.209119	-3.143937
H	-2.316484	-1.889286	-3.998855
C	-3.551965	-0.354310	-2.895398
H	-4.448179	-0.270104	-3.525071
C	-3.318327	0.342485	-1.660048
H	-3.995916	1.069176	-1.193423
C	-3.154394	-3.003972	0.217358
H	-2.302472	-3.087778	0.904339
C	-3.301808	-3.704579	-1.030979
H	-2.587356	-4.421491	-1.457642
C	-4.539811	-3.283048	-1.636175
H	-4.931813	-3.618380	-2.605847
C	-5.159538	-2.322864	-0.758202
H	-6.106692	-1.798174	-0.942661
C	-4.302623	-2.147915	0.385797
H	-4.475837	-1.466347	1.228878
C	-1.040261	2.467037	-0.172266
C	-0.654855	3.399423	0.824034
H	-0.485794	3.055339	1.857901
C	-0.491791	4.756600	0.532348
H	-0.201857	5.458019	1.331743
C	-0.693221	5.249248	-0.789273
C	-1.068873	4.313653	-1.791479
H	-1.237662	4.664534	-2.822775
C	-1.234301	2.955589	-1.485099
H	-1.539939	2.267597	-2.288668
C	-2.321216	0.694522	1.690243
C	-2.210503	-0.214469	2.767772
H	-1.380146	-0.934024	2.797764
C	-3.144578	-0.231616	3.810612
H	-3.025678	-0.950598	4.637547
C	-4.243550	0.672162	3.824827
C	-4.354231	1.591371	2.742731
H	-5.188984	2.311186	2.727701
C	-3.411858	1.599924	1.709006
H	-3.523632	2.340167	0.900735
C	-0.116884	-1.754101	-1.918374
H	-0.226001	-2.526630	-1.130633
C	1.303022	0.191732	-2.382287
H	1.873488	-0.227261	-3.244917
H	0.403576	0.684605	-2.836464
C	2.121323	1.256398	-1.717820
C	2.706002	2.251414	0.316878
H	2.598311	2.249210	1.410974
C	3.514711	3.194006	-0.322006
H	4.049393	3.945109	0.277649
C	3.630231	3.154758	-1.724533
C	2.925191	2.169352	-2.426091
H	2.985724	2.097318	-3.522541
C	0.298235	-2.492989	-3.205954
H	-0.425978	-3.299873	-3.438047
H	0.345918	-1.822776	-4.088755
H	1.292429	-2.963771	-3.066282
C	1.173739	0.343126	2.120302
C	0.286234	-1.734303	1.129550
H	2.318615	-1.691492	-1.328143
H	2.868359	-1.251540	0.608567
C	3.611977	-1.964507	0.099955
C	3.644055	-3.274506	0.939367
C	4.647766	-2.999357	2.086044
O	3.253900	-2.117793	-1.240342
H	4.263053	3.879890	-2.258851
C	5.001768	-1.390008	0.334268
C	5.592282	-1.976458	1.476042
C	5.685746	-0.420295	-0.414622
C	6.878235	-1.584546	1.884616
C	6.976814	-0.029851	-0.006012
H	5.219933	0.014275	-1.314003
C	7.567268	-0.606848	1.137083
H	7.351112	-2.040746	2.770342
H	7.532297	0.725377	-0.585584
H	8.579989	-0.298128	1.443455
H	5.168265	-3.909637	2.451191
H	4.127351	-2.559408	2.968082
H	4.038215	-4.070986	0.270322
H	2.639159	-3.586629	1.286211
N	-5.135885	0.694614	4.872459
H	-6.028648	1.172597	4.741471
H	-5.171427	-0.111686	5.498028
N	-0.484444	6.577907	-1.091352
H	-0.874321	6.941042	-1.962714
H	-0.473155	7.250320	-0.322621

Mn20/viii
Frequencies, energies and thermodynamic properties:

```
 Lowest Vibrational Mode (1/cm) =                        18.0184
 2nd Lowest Vibrational Mode (1/cm) =                    25.0784
 E(RB-P86) (a.u.) =                                     -4363.21770420
 Thermal correction to Enthalpy (a.u.) =                 0.593073
 Thermal correction to Gibbs Free Energy (a.u.) =        0.482072
 Total Entropy (cal/Kmol) =                              233.621
 E(RPBE1PBE) (a.u.) =                                   -4362.54147135
Optimised cartesian coordinates (Angstrom):
 Fe2.828263  -1.123742   1.058478
 Mn-1.012003  -0.678620  -1.918110
 P     -0.183763   0.425429  -0.116850
 O     -1.604964   1.864515  -3.319557
 O      1.544374  -0.880252  -3.360646
 N     -0.797196  -2.444950  -0.896134
 N     -2.916202  -0.869575  -1.120647
 C      0.826280  -0.654487   0.975001
 C      1.018431  -2.087065   0.783852
 C      1.697002  -2.583193   1.958366
 H      2.010024  -3.621822   2.126785
 C      1.934601  -1.486668   2.860732
 H      2.450675  -1.545502   3.828911
 C      1.408299  -0.293406   2.255300
 H      1.433389   0.713721   2.691202
 C      3.806216  -0.713377  -0.710516
 H      3.326644  -0.583253  -1.689237
 C      4.266086  -1.959677  -0.157960
 H      4.206231  -2.942851  -0.644023
 C      4.797077  -1.696608   1.156042
 H      5.209895  -2.443713   1.847356
 C      4.668031  -0.284078   1.413051
 H      4.965587   0.233086   2.335327
 C      4.052548   0.323441   0.261459
 H      3.792672   1.383967   0.147513
 C     -1.502103   1.056319   1.020131
 C     -2.286536   2.159063   0.599090
 H     -2.068145   2.649074  -0.364116
 C     -3.334773   2.654117   1.381440
 H     -3.919937   3.519422   1.029141
 C     -3.659316   2.054561   2.631440
 C     -2.885018   0.937442   3.046392
 H     -3.112856   0.448782   4.007975
 C     -1.832915   0.454246   2.255001
 H     -1.253192  -0.408588   2.618269
 C      0.834864   1.951827  -0.366826
 C      1.501015   2.212637  -1.586303
 H      1.392569   1.519840  -2.432391
 C      2.310558   3.343257  -1.754282
 H      2.810472   3.517127  -2.721184
 C      2.492895   4.277282  -0.697424
 C      1.816679   4.022095   0.529076
 H      1.925633   4.733026   1.364615
 C      1.008363   2.890288   0.681312
 H      0.485073   2.743372   1.639519
 C      0.520384  -2.879065  -0.434746
 H      1.223283  -2.667431  -1.267914
 C     -1.844114  -2.684189   0.070848
 H     -2.110079  -3.767998   0.183496
 H     -1.580366  -2.361160   1.123250
 C     -3.092013  -1.929195  -0.282411
 C     -3.995141  -0.110335  -1.445792
 C     -5.281289  -0.374566  -0.966694
 C     -5.472662  -1.475663  -0.110916
 C     -4.362695  -2.257540   0.230338
 C      0.600357  -4.401760  -0.179893
 H      1.648846  -4.725040  -0.012744
 H      0.010521  -4.714384   0.707145
 H      0.209919  -4.946592  -1.063831
 C     -1.376881   0.837159  -2.777540
 C      0.548547  -0.780398  -2.727439
 H     -1.539240  -2.038054  -2.926096
 H     -1.815896  -1.406621  -3.364177
 H     -3.809642   0.736094  -2.123153
 H     -6.117690   0.272815  -1.268664
 H     -4.463920  -3.129283   0.894732
 H     -6.472794  -1.719300   0.279809
 N      3.321514   5.368759  -0.841457
 H      3.576341   5.659373  -1.786817
 H      3.227524   6.136601  -0.174708
 N     -4.657465   2.572901   3.431945
 H     -5.035328   1.975697   4.169656
 H     -5.351016   3.182128   2.994325
---------------------------------------
Mn20/ix
Frequencies, energies and thermodynamic properties:
 Lowest Vibrational Mode (1/cm) =                        20.9050
```

```
 2nd Lowest Vibrational Mode (1/cm) =                    27.0019
 E(RB-P86) (a.u.) =                                    -4517.00496991
 Thermal correction to Enthalpy (a.u.) =                 0.662267
 Thermal correction to Gibbs Free Energy (a.u.) =        0.542684
 Total Entropy (cal/Kmol) =                              251.684
 E(RPBE1PBE) (a.u.) =                                  -4516.33297843
Optimised cartesian coordinates (Angstrom):
 Fe-2.997318  -0.832747  -1.335636
 Mn1.211295  -1.013471   1.110226
 P     -0.032396   0.598927   0.106033
 O      1.791211   0.692494   3.434312
 O     -1.033752  -2.219395   2.576027
 N      1.004483  -2.232738  -0.627432
 N      2.937777  -0.494826   0.071483
 C     -1.082185  -0.086812  -1.255614
 C     -1.081872  -1.472319  -1.723321
 C     -1.891846  -1.524895  -2.919301
 H     -2.110368  -2.423809  -3.509507
 C     -2.400238  -0.207982  -3.191833
 H     -3.067725   0.066391  -4.019876
 C     -1.912141   0.675874  -2.169675
 H     -2.126067   1.749625  -2.094684
 C     -3.692190  -1.374425   0.527123
 H     -3.066205  -1.570172   1.407279
 C     -4.085270  -2.345921  -0.459612
 H     -3.820636  -3.412008  -0.458018
 C     -4.868394  -1.671109  -1.463396
 H     -5.300077  -2.130560  -2.362797
 C     -4.961535  -0.281276  -1.094235
 H     -5.476275   0.503814  -1.664367
 C     -4.232470  -0.096870   0.133782
 H     -4.088175   0.851834   0.667007
 C      0.965892   1.889147  -0.779596
 C      1.674041   2.837970  -0.000368
 H      1.559870   2.836956   1.096450
 C      2.511735   3.792282  -0.587324
 H      3.039534   4.522713   0.047900
 C      2.696319   3.832952  -1.998365
 C      1.997176   2.875199  -2.780487
 H      2.115507   2.882688  -3.876568
 C      1.154735   1.927639  -2.179366
 H      0.621440   1.214433  -2.827423
 C     -1.182151   1.661363   1.096659
 C     -1.634672   1.264211   2.375999
 H     -1.281765   0.320454   2.814599
 C     -2.533002   2.046037   3.113723
 H     -2.860498   1.706750   4.110127
 C     -3.024636   3.277651   2.600208
 C     -2.562220   3.686184   1.317319
 H     -2.912008   4.643209   0.896307
 C     -1.662373   2.896133   0.593286
 H     -1.311071   3.262241  -0.384575
 C     -0.347522  -2.648836  -1.088710
 H     -0.879151  -2.932977  -0.157928
 C      1.930236  -1.831982  -1.698623
 H      2.268271  -2.689775  -2.320782
 H      1.394737  -1.138399  -2.386818
 C      3.121974  -1.104832  -1.129555
 C      3.959689   0.228985   0.589335
 C      5.194607   0.371713  -0.053697
 C      5.393689  -0.268674  -1.288648
 C      4.339814  -1.018099  -1.829132
 C     -0.313039  -3.890035  -1.997225
 H     -1.339800  -4.269141  -2.172256
 H      0.141600  -3.678835  -2.986623
 H      0.264810  -4.702895  -1.511730
 C      1.564124  -0.009206   2.504160
 C     -0.158457  -1.705700   1.961010
 H      1.461301  -2.964685  -0.020079
 H      4.449186  -1.539189  -2.792383
 H      5.987009   0.971303   0.418060
 H      3.775418   0.702900   1.564413
 H      6.354859  -0.188345  -1.819306
 O      2.349840  -2.676629   1.469239
 C      2.363529  -3.399888   2.662296
 C      3.238105  -2.781579   3.766170
 H      2.761875  -4.430364   2.453691
 H      1.334624  -3.567425   3.085782
 H      3.263145  -3.429207   4.670584
 H      4.282449  -2.650404   3.408204
 H      2.856502  -1.784123   4.067790
 N     -3.942856   4.030721   3.300490
 H     -4.064867   5.008976   3.033315
 H     -4.046908   3.852552   4.300868
 N      3.564238   4.738804  -2.578387
```

```
 H      3.813549   5.567617  -2.035247
 H      3.459156   4.919275  -3.578644
---------------------------------------
Mn20/x
Frequencies, energies and thermodynamic properties:
 Lowest Vibrational Mode (1/cm) =                         13.1464
 2nd Lowest Vibrational Mode (1/cm) =                     22.0004
 E(RB-P86) (a.u.) =                                      -4671.94688544
 Thermal correction to Enthalpy (a.u.) =                  0.746481
 Thermal correction to Gibbs Free Energy (a.u.) =         0.613471
 Total Entropy (cal/Kmol) =                               279.943
 E(RPBE1PBE) (a.u.) =                                    -4671.28266454
Optimised cartesian coordinates (Angstrom):
 Fe-2.792174  -1.833020  -1.249994
 Mn1.231669  -0.205962   1.008822
 P     -0.634774   0.710463   0.079519
 O      0.998765   1.654696   3.273358
 O     -0.162085  -2.299214   2.532022
 N      1.484874  -1.416191  -0.756490
 N      2.528951   0.981105  -0.096973
 C     -1.379417  -0.339914  -1.242155
 C     -0.811170  -1.588983  -1.742054
 C     -1.581416  -1.980550  -2.900859
 H     -1.424573  -2.884964  -3.502035
 C     -2.618084  -1.007888  -3.116390
 H     -3.381387  -1.044438  -3.905339
 C     -2.503304  -0.002617  -2.096131
 H     -3.151427   0.875948  -1.983848
 C     -3.074210  -2.652096   0.619366
 H     -2.367622  -2.587686   1.457008
 C     -3.094448  -3.679508  -0.388389
 H     -2.412614  -4.538494  -0.449208
 C     -4.151915  -3.372332  -1.317655
 H     -4.413256  -3.951994  -2.213210
 C     -4.787902  -2.155337  -0.881023
 H     -5.618375  -1.644694  -1.386861
 C     -4.120598  -1.708239   0.313833
 H     -4.346520  -0.796153   0.881365
 C     -0.316219   2.307385  -0.810963
 C      0.000392   3.452769  -0.037918
 H     -0.017423   3.390941   1.062810
 C      0.324015   4.675422  -0.635293
 H      0.554462   5.549067  -0.003645
 C      0.362860   4.809190  -2.052224
 C      0.055019   3.660230  -2.829100
 H      0.070213   3.730977  -3.929185
 C     -0.274954   2.441551  -2.217156
 H     -0.522870   1.583243  -2.861158
 C     -2.063313   1.183336   1.159825
 C     -2.258622   0.595080   2.430656
 H     -1.535310  -0.137757   2.814693
 C     -3.363513   0.918791   3.228340
 H     -3.481074   0.443462   4.215927
 C     -4.335554   1.858604   2.786774
 C     -4.136994   2.459403   1.511592
 H     -4.864537   3.201961   1.144607
 C     -3.026553   2.128465   0.727055
 H     -2.899157   2.633566  -0.243634
 C      0.376318  -2.343870  -1.163612
 H      0.058900  -2.817794  -0.213205
 C      2.015118  -0.598709  -1.868382
 H      2.593064  -1.198854  -2.604325
 H      1.157950  -0.157394  -2.425388
 C      2.859060   0.529003  -1.336177
 C      3.208156   2.040221   0.405037
 C      4.235201   2.685785  -0.293874
 C      4.590638   2.211683  -1.567540
 C      3.890990   1.115055  -2.091416
 C      0.880652  -3.466589  -2.086960
 H      0.098065  -4.240469  -2.217788
 H      1.159445  -3.094718  -3.094235
 H      1.766145  -3.949864  -1.627128
 C      1.103418   0.896913   2.367638
 C      0.349421  -1.439156   1.897297
 H      3.384467  -2.273780   0.896318
 H      2.266221  -2.047950  -0.444092
 O      3.568125  -3.088737   0.238106
 C      4.865766  -2.934744  -0.316511
 H      4.871533  -3.394438  -1.333259
 H      5.116191  -1.853778  -0.459492
 H      4.134902   0.706828  -3.083843
 H      4.749412   3.543047   0.165038
 H      2.911946   2.378247   1.408300
 C      5.950944  -3.598300   0.537027
 H      5.728508  -4.677046   0.679646
```

```
 H      6.949903  -3.511256   0.057760
 H      6.011767  -3.128125   1.541711
 H      5.401059   2.686174  -2.141923
 O      3.023515  -1.074109   1.598347
 C      3.381332  -1.229538   2.951224
 C      4.227566  -0.072083   3.499276
 H      3.974335  -2.176094   3.061536
 H      2.486000  -1.363511   3.611405
 H      4.553595  -0.279116   4.542445
 H      5.135864   0.077790   2.877341
 H      3.654425   0.877767   3.505522
 N     -5.447444   2.149322   3.547196
 H     -5.973146   2.996979   3.327678
 H     -5.427822   1.910690   4.540083
 N      0.735263   5.999525  -2.645821
 H      0.674292   6.849809  -2.082558
 H      0.490716   6.136502  -3.628334
---------------------------------------
Mn20/TS-i
Frequencies, energies and thermodynamic properties:
 Lowest Vibrational Mode (1/cm) =-736.6930
 2nd Lowest Vibrational Mode (1/cm) =                     22.9677
 E(RB-P86) (a.u.) =                                      -4518.15984223
 Thermal correction to Enthalpy (a.u.) =                  0.674067
 Thermal correction to Gibbs Free Energy (a.u.) =         0.552552
 Total Entropy (cal/Kmol) =                               255.750
 E(RPBE1PBE) (a.u.) =                                    -4517.48654792
Optimised cartesian coordinates (Angstrom):
 Fe-2.639322  -1.566501  -1.334965
 Mn1.242951  -0.437300   1.390803
 P     -0.295373   0.705513   0.147439
 O      1.047572   1.544761   3.572108
 O     -0.692404  -2.157171   2.791123
 N      1.542983  -1.760866  -0.218062
 N      2.870247   0.452704   0.472808
 C     -1.010239  -0.320220  -1.200409
 C     -0.590301  -1.676968  -1.537109
 C     -1.248912  -2.031578  -2.773295
 H     -1.162296  -2.994294  -3.292971
 C     -2.072333  -0.930252  -3.195707
 H     -2.714398  -0.909211  -4.086738
 C     -1.936372   0.122601  -2.227041
 H     -2.439407   1.097530  -2.261403
 C     -3.319465  -2.173239   0.513851
 H     -2.744216  -2.143244   1.447955
 C     -3.352392  -3.268604  -0.418750
 H     -2.812131  -4.219557  -0.317679
 C     -4.198694  -2.889994  -1.521873
 H     -4.412993  -3.499322  -2.410221
 C     -4.692136  -1.559959  -1.267708
 H     -5.348323  -0.978714  -1.929507
 C     -4.146897  -1.115365  -0.011317
 H     -4.309603  -0.135882   0.457216
 C      0.422596   2.150970  -0.759822
 C      0.822882   3.285268  -0.009494
 H      0.651647   3.309669   1.079421
 C      1.426111   4.390914  -0.617288
 H      1.716564   5.260722  -0.005509
 C      1.671998   4.409742  -2.019803
 C      1.285715   3.266723  -2.770882
 H      1.463047   3.249544  -3.858734
 C      0.675614   2.167150  -2.150316
 H      0.381828   1.308547  -2.774141
 C     -1.747879   1.485107   0.986859
 C     -2.213242   1.036013   2.244381
 H     -1.688505   0.226217   2.770338
 C     -3.346535   1.593287   2.849463
 H     -3.678847   1.220436   3.832040
 C     -4.073804   2.640583   2.218756
 C     -3.605373   3.097356   0.954111
 H     -4.140835   3.913286   0.441441
 C     -2.469553   2.533733   0.363116
 H     -2.130033   2.930130  -0.607004
 C      0.390129  -2.537430  -0.743233
 H     -0.138453  -2.908452   0.158593
 C      2.333407  -1.130622  -1.281425
 H      2.915436  -1.867158  -1.882996
 H      1.673514  -0.602773  -2.015976
 C      3.265224  -0.101345  -0.707553
 C      3.633389   1.436069   1.015381
 C      4.813248   1.896084   0.421695
 C      5.234749   1.315826  -0.787339
 C      4.447551   0.304815  -1.354203
 C      0.841867  -3.784244  -1.531258
 H     -0.020657  -4.447796  -1.744865
```

```
 H      1.315267  -3.526128  -2.501162
 H      1.570659  -4.354982  -0.921007
 C      1.134352   0.749119   2.702803
 C      0.040816  -1.449746   2.190261
 H      2.489078  -1.044916   2.397018
 H      2.442217  -1.776208   1.955634
 H      2.296171  -2.599682   0.435514
 O      2.902676  -3.298097   1.226946
 C      4.294757  -3.268790   1.042088
 H      4.573913  -3.350747  -0.043401
 H      4.743333  -2.294461   1.387020
 H      4.737967  -0.179374  -2.298738
 H      5.391699   2.693394   0.910947
 H      3.280577   1.859217   1.967076
 C      4.978111  -4.407841   1.806038
 H      4.592916  -5.392038   1.463212
 H      6.079885  -4.394900   1.659994
 H      4.772984  -4.327626   2.895085
 H      6.163526   1.645191  -1.278076
 N      2.219953   5.517957  -2.632109
 H      2.618331   5.400768  -3.565387
 H      2.718234   6.190636  -2.046749
 N     -5.156777   3.229058   2.832518
 H     -5.605371   2.732219   3.603725
 H     -5.791475   3.789885   2.262094
---------------------------------------
Mn20/TS-ii_si
Frequencies, energies and thermodynamic properties:
 Lowest Vibrational Mode (1/cm) =-211.5966
 2nd Lowest Vibrational Mode (1/cm) =                     13.1119
 E(RB-P86) (a.u.) =                                     -4785.95807836
 Thermal correction to Enthalpy (a.u.) =                  0.747244
 Thermal correction to Gibbs Free Energy (a.u.) =         0.617492
 Total Entropy (cal/Kmol) =                               273.086
 E(RPBE1PBE) (a.u.) =                                   -4785.27102197
Optimised cartesian coordinates (Angstrom):
 Fe-2.697114  -2.035612  -1.536664
 Mn1.170985   0.214149   0.329995
 P     -1.078119   0.555585   0.351816
 O      1.621068   0.803323   3.176165
 O      1.164313  -2.653579   0.977969
 N      1.146527  -0.070147  -1.759509
 N      1.655216   2.101796  -0.300533
 C     -1.892735  -0.188590  -1.129315
 C     -1.192174  -0.826652  -2.243329
 C     -2.171048  -1.082663  -3.276360
 H     -1.977142  -1.569660  -4.240301
 C     -3.456719  -0.629963  -2.819223
 H     -4.402216  -0.712675  -3.372090
 C     -3.291840  -0.086405  -1.499650
 H     -4.088165   0.339602  -0.875893
 C     -2.028001  -3.479318  -0.228519
 H     -1.121660  -3.407259   0.386771
 C     -2.098245  -4.005109  -1.566779
 H     -1.258730  -4.413837  -2.145284
 C     -3.458810  -3.880692  -2.024544
 H     -3.836701  -4.172246  -3.013749
 C     -4.230579  -3.280089  -0.966289
 H     -5.300030  -3.033459  -1.008767
 C     -3.346814  -3.029814   0.142537
 H     -3.617845  -2.556372   1.095240
 C     -1.584907   2.336442   0.235109
 C     -1.347606   3.170303   1.356924
 H     -0.941291   2.733799   2.284710
 C     -1.623753   4.541394   1.325171
 H     -1.437207   5.158145   2.219793
 C     -2.142205   5.155248   0.150046
 C     -2.361890   4.325802  -0.981994
 H     -2.760549   4.770451  -1.908729
 C     -2.088496   2.950287  -0.934211
 H     -2.287414   2.345005  -1.832635
 C     -2.093854  -0.015404   1.790495
 C     -1.618225  -1.018065   2.666327
 H     -0.620457  -1.452337   2.508567
 C     -2.393206  -1.487547   3.734631
 H     -1.990422  -2.268752   4.399985
 C     -3.692912  -0.964909   3.981159
 C     -4.172556   0.047948   3.103152
 H     -5.172880   0.479810   3.271512
 C     -3.385956   0.509076   2.041279
 H     -3.786456   1.308520   1.397473
 C      0.291974  -1.165362  -2.326970
 H      0.491275  -2.031689  -1.665126
 C      1.002083   1.221178  -2.463955
 H      1.444540   1.204165  -3.483524
```

```
 H     -0.083374   1.436207  -2.589571
 C      1.610935   2.326549  -1.646536
 C      2.115194   3.106084   0.494651
 H      2.133371   2.899846   1.574350
 C      2.554993   4.336487  -0.005567
 H      2.916682   5.104985   0.693500
 C      2.531733   4.557902  -1.393274
 C      2.052063   3.531151  -2.219966
 H      2.009651   3.654516  -3.312737
 C      0.732443  -1.562161  -3.746796
 H      0.220582  -2.492352  -4.064439
 H      0.504360  -0.778324  -4.497707
 H      1.823755  -1.758227  -3.761438
 C      1.417139   0.569048   2.030301
 C      1.109471  -1.499638   0.705614
 H      2.152450  -0.365626  -1.896957
 H      2.784108  -0.093663   0.203942
 C      4.213377  -0.417148  -0.735985
 C      4.609206  -1.583890   0.126473
 C      4.972200   0.796188  -0.148530
 C      5.289671  -1.122058   1.275571
 C      4.442634  -2.955484  -0.126184
 C      5.322640   0.396199   1.301430
 H      5.899583   0.897013  -0.758860
 C      5.809061  -2.045400   2.197686
 C      4.966237  -3.877593   0.797337
 H      3.918656  -3.289600  -1.036040
 H      6.295148   0.798895   1.652778
 C      5.642795  -3.423779   1.950844
 H      6.346277  -1.703339   3.097709
 H      4.855544  -4.959678   0.620873
 H      6.052306  -4.157464   2.664011
 O      3.825227  -0.504302  -1.932512
 H      4.411295   1.744787  -0.246114
 H      4.545718   0.769956   2.006700
 H      2.880422   5.509514  -1.822522
 N     -4.437697  -1.381498   5.064056
 H     -5.440043  -1.185193   5.062285
 H     -4.182975  -2.264129   5.510583
 N     -2.466981   6.498780   0.130400
 H     -2.561718   6.947892  -0.782555
 H     -2.040551   7.097799   0.839965
----------------------------------------
Mn20/TS-ii_re
Frequencies, energies and thermodynamic properties:
 Lowest Vibrational Mode (1/cm) =-217.8841
 2nd Lowest Vibrational Mode (1/cm) =                      13.6434
 E(RB-P86) (a.u.) =                                      -4785.95863471
 Thermal correction to Enthalpy (a.u.) =                   0.747385
 Thermal correction to Gibbs Free Energy (a.u.) =          0.617369
 Total Entropy (cal/Kmol) =                                273.643
 E(RPBE1PBE) (a.u.) =                                    -4785.27252621
Optimised cartesian coordinates (Angstrom):
 Fe-3.427969  -1.443949  -1.238201
 Mn0.995439  -0.421219   0.467546
 P     -0.964168   0.713569   0.233263
 O      1.587314   0.547820   3.181740
 O     -0.077341  -2.887130   1.652595
 N      0.870617  -1.105664  -1.523453
 N      2.156333   0.980924  -0.477322
 C     -1.993350   0.025235  -1.137192
 C     -1.578855  -1.045217  -2.042880
 C     -2.579468  -1.141027  -3.082156
 H     -2.578306  -1.857904  -3.912890
 C     -3.605008  -0.165724  -2.829058
 H     -4.511621  -0.014125  -3.430325
 C     -3.253158   0.547667  -1.632539
 H     -3.835604   1.355727  -1.171427
 C     -4.418886  -1.743690   0.537805
 H     -4.507341  -1.006660   1.346559
 C     -3.355217  -2.705604   0.388225
 H     -2.494610  -2.830473   1.058167
 C     -3.598140  -3.452451  -0.818118
 H     -2.961789  -4.251133  -1.222691
 C     -4.810802  -2.953697  -1.415472
 H     -5.257500  -3.301241  -2.356769
 C     -5.319142  -1.899262  -0.575108
 H     -6.221082  -1.301941  -0.765056
 C     -0.763389   2.489135  -0.265171
 C     -0.222200   3.388335   0.688113
 H     -0.005364   3.033795   1.709648
 C      0.034581   4.726730   0.372805
 H      0.445861   5.401495   1.141597
 C     -0.223629   5.232285  -0.932772
 C     -0.753132   4.330185  -1.893804
```

```
 H     -0.967650   4.690700  -2.913362
 C     -1.012926   2.991560  -1.562407
 H     -1.437186   2.332741  -2.336339
 C     -2.125368   0.868826   1.666717
 C     -2.062202  -0.029050   2.756825
 H     -1.294991  -0.816621   2.770427
 C     -2.959042   0.054420   3.829680
 H     -2.879162  -0.659834   4.665582
 C     -3.970067   1.054392   3.862090
 C     -4.028792   1.966721   2.770791
 H     -4.792028   2.762310   2.771918
 C     -3.124581   1.872411   1.706626
 H     -3.189580   2.612100   0.892580
 C     -0.328573  -1.909776  -1.932525
 H     -0.471312  -2.623343  -1.096848
 C      1.215647  -0.027553  -2.473934
 H      1.626537  -0.416959  -3.430741
 H      0.286413   0.530287  -2.729976
 C      2.184332   0.933153  -1.840608
 C      2.955170   1.889743   0.142743
 H      2.914813   1.900955   1.241241
 C      3.792440   2.769845  -0.551364
 H      4.410046   3.484702   0.012053
 C      3.831207   2.711771  -1.954559
 C      3.017013   1.770912  -2.602061
 H      3.010521   1.683871  -3.699155
 C     -0.061273  -2.738000  -3.201157
 H     -0.884847  -3.459740  -3.371577
 H      0.029432  -2.108319  -4.109807
 H      0.874863  -3.319696  -3.079072
 C      1.337335   0.170331   2.084780
 C      0.296425  -1.870555   1.161871
 H      1.693593  -1.770749  -1.521846
 H      2.393433  -1.295733   0.518134
 C      3.534177  -2.391448  -0.194802
 C      4.709308  -1.581141   0.276888
 C      3.323026  -3.470058   0.894425
 C      5.010441  -1.909267   1.618628
 C      5.499566  -0.677102  -0.452484
 C      4.003431  -2.908107   2.161591
 C      6.117070  -1.318744   2.250155
 C      6.608781  -0.089776   0.182389
 H      5.252178  -0.450395  -1.501986
 C      6.912687  -0.408310   1.524041
 H      6.369886  -1.566831   3.294287
 H      7.250167   0.616699  -0.368733
 H      7.788111   0.054998   2.007359
 O      3.186718  -2.516444  -1.400980
 H      4.471736  -3.685932   2.799592
 H      3.259220  -2.376161   2.797118
 H      2.262305  -3.752901   1.025305
 H      3.868660  -4.370986   0.529322
 H      4.484738   3.382622  -2.532804
 N     -4.883652   1.115056   4.893094
 H     -5.409814   1.981470   5.018732
 H     -4.656753   0.631860   5.763910
 N      0.080269   6.539428  -1.263933
 H      0.181544   7.208514  -0.498268
 H     -0.364876   6.931666  -2.095884
---------------------------------------
Mn20/TS-iii
Frequencies, energies and thermodynamic properties:
 Lowest Vibrational Mode (1/cm) =-651.4945
 2nd Lowest Vibrational Mode (1/cm) =                      21.4157
 E(RB-P86) (a.u.) =                                      -4363.21430012
 Thermal correction to Enthalpy (a.u.) =                   0.592083
 Thermal correction to Gibbs Free Energy (a.u.) =          0.482088
 Total Entropy (cal/Kmol) =                                231.502
 E(RPBE1PBE) (a.u.) =                                    -4362.53511318
Optimised cartesian coordinates (Angstrom):
 Fe-2.810007  -1.113355  -1.067467
 Mn0.992394  -0.721378   1.900857
 P      0.190137   0.455420   0.117724
 O      1.650571   1.643873   3.546202
 O     -1.570182  -0.913877   3.331200
 N      0.818562  -2.518484   0.841105
 N      2.905892  -0.884579   1.112822
 C     -0.810113  -0.638354  -0.976832
 C     -1.002718  -2.077994  -0.803176
 C     -1.677927  -2.557964  -1.987468
 H     -1.989380  -3.594302  -2.170879
 C     -1.915951  -1.451425  -2.875443
 H     -2.432338  -1.498968  -3.843930
 C     -1.391051  -0.266664  -2.254621
 H     -1.418427   0.745972  -2.677318
```

```
 C     -3.792864  -0.695256   0.696926
 H     -3.314850  -0.552947   1.674420
 C     -4.241902  -1.950419   0.155143
 H     -4.174788  -2.928553   0.650329
 C     -4.774190  -1.703426  -1.161231
 H     -5.180206  -2.459947  -1.846290
 C     -4.656377  -0.292271  -1.430765
 H     -4.956671   0.213894  -2.358221
 C     -4.046802   0.330580  -0.284220
 H     -3.794456   1.394006  -0.180492
 C      1.497671   1.108227  -1.021587
 C      2.280898   2.205180  -0.583292
 H      2.062825   2.678413   0.388624
 C      3.326914   2.716453  -1.358643
 H      3.910592   3.576937  -0.992218
 C      3.651126   2.139246  -2.619028
 C      2.879251   1.027179  -3.051210
 H      3.107461   0.554724  -4.020814
 C      1.829370   0.528006  -2.266421
 H      1.252501  -0.331004  -2.643527
 C     -0.843905   1.968382   0.378913
 C     -1.487033   2.218633   1.612864
 H     -1.354589   1.521830   2.452546
 C     -2.302052   3.342139   1.802200
 H     -2.784431   3.507865   2.779412
 C     -2.511703   4.279507   0.753414
 C     -1.856481   4.036080  -0.486990
 H     -1.986024   4.751008  -1.316170
 C     -1.043033   2.911055  -0.660661
 H     -0.535556   2.773492  -1.628803
 C     -0.513371  -2.920445   0.384966
 H     -1.197971  -2.726324   1.237026
 C      1.866670  -2.681356  -0.146006
 H      2.143435  -3.750772  -0.327371
 H      1.571388  -2.288336  -1.160855
 C      3.103249  -1.923425   0.253063
 C      3.974687  -0.126386   1.469032
 C      5.269246  -0.367967   0.998096
 C      5.481445  -1.447223   0.122072
 C      4.382077  -2.232111  -0.248643
 C     -0.607438  -4.432412   0.082679
 H     -1.658181  -4.740636  -0.096235
 H     -0.017470  -4.721381  -0.812079
 H     -0.224866  -5.007883   0.950127
 C      1.386529   0.697409   2.887409
 C     -0.573068  -0.821788   2.698144
 H      1.299107  -2.317828   2.336000
 H      1.590231  -1.820397   3.063886
 H      3.776128   0.700272   2.166383
 H      6.095441   0.280679   1.324423
 H      4.499569  -3.089411  -0.928733
 H      6.488181  -1.673976  -0.261596
 N      4.647001   2.674003  -3.412899
 H      5.027107   2.086578  -4.157438
 H      5.342124   3.272639  -2.963064
 N     -3.346318   5.363816   0.918204
 H     -3.583871   5.648586   1.869862
 H     -3.269733   6.135767   0.253903
---------------------------------------
Mn21/i
Frequencies, energies and thermodynamic properties:
 Lowest Vibrational Mode (1/cm) =                         17.7572
 2nd Lowest Vibrational Mode (1/cm) =                     21.5831
 E(RB-P86) (a.u.) =                                     -4480.28550103
 Thermal correction to Enthalpy (a.u.) =                  0.610032
 Thermal correction to Gibbs Free Energy (a.u.) =         0.493980
 Total Entropy (cal/Kmol) =                               244.251
 E(RPBE1PBE) (a.u.) =                                   -4479.61872087
Optimised cartesian coordinates (Angstrom):
 Fe2.661726  -1.506428   1.291847
 Mn-0.815715  -0.819954  -1.887516
 P     -0.094596   0.289259  -0.139821
 O     -1.292663   1.503298  -3.671887
 O      1.784844  -1.139853  -3.216855
 N     -0.809280  -2.502776  -1.006661
 N     -2.831484  -0.974133  -1.476157
 C      0.724711  -0.877768   1.032262
 C      0.821276  -2.322613   0.842380
 C      1.345192  -2.881783   2.065927
 H      1.559067  -3.943032   2.246972
 C      1.577946  -1.814013   3.002462
 H      1.992957  -1.921232   4.013959
 C      1.204574  -0.578591   2.369402
 H      1.264563   0.418066   2.825328
 C      3.816102  -1.148240  -0.376722
```

```
 H      3.432704  -0.967737  -1.389487
 C      4.130655  -2.434521   0.186905
 H      4.036664  -3.403073  -0.322494
 C      4.566444  -2.232918   1.545642
 H      4.859213  -3.020005   2.253531
 C      4.523864  -0.819058   1.820861
 H      4.778852  -0.340148   2.775862
 C      4.057953  -0.148603   0.634462
 H      3.893133   0.931082   0.523309
 C     -1.487499   0.975865   0.873637
 C     -2.125833   2.160511   0.443339
 H     -1.738120   2.704377  -0.433668
 C     -3.249365   2.676716   1.110158
 H     -3.708037   3.607516   0.747853
 C     -3.773795   1.992796   2.232450
 C     -3.153975   0.795650   2.662553
 H     -3.570563   0.271753   3.536781
 C     -2.030425   0.297151   1.991036
 H     -1.561177  -0.630153   2.354302
 C      1.037704   1.749097  -0.266170
 C      1.775746   1.988792  -1.442540
 H      1.660084   1.321463  -2.308540
 C      2.671018   3.069369  -1.543959
 H      3.223406   3.220700  -2.481878
 C      2.841449   3.944817  -0.447752
 C      2.097402   3.720859   0.737672
 H      2.228327   4.416006   1.581362
 C      1.209797   2.644090   0.820813
 H      0.628903   2.506979   1.746395
 C      0.421370  -3.078168  -0.435254
 H      1.214299  -2.889462  -1.186014
 C     -2.017037  -2.921059  -0.321201
 H     -2.263498  -3.996501  -0.498939
 H     -1.920604  -2.834128   0.794749
 C     -3.176489  -2.076964  -0.750974
 C     -3.827668  -0.137708  -1.883362
 H     -3.516674   0.743504  -2.462586
 C     -5.174941  -0.371624  -1.603048
 H     -5.934001   0.338187  -1.963712
 C     -5.532606  -1.517520  -0.865177
 C     -4.514852  -2.378352  -0.437377
 H     -4.742017  -3.285914   0.142621
 C      0.371115  -4.602223  -0.218114
 H      1.381478  -4.988409   0.025981
 H     -0.307953  -4.897809   0.607733
 H      0.033837  -5.106560  -1.146762
 C     -1.094447   0.578268  -2.956415
 C      0.766076  -0.998913  -2.622643
 H     -6.586467  -1.731994  -0.630537
 O     -4.854846   2.402606   2.951393
 C     -5.518560   3.605269   2.566191
 H     -6.351231   3.741835   3.280988
 H     -4.841174   4.485613   2.625231
 H     -5.932219   3.534776   1.536063
 O      3.674264   5.019337  -0.437616
 C      4.449881   5.297141  -1.603388
 H      5.046365   6.198494  -1.370142
 H      5.137930   4.458052  -1.847044
 H      3.804747   5.505582  -2.484906
---------------------------------------
Mn21/ii
Frequencies, energies and thermodynamic properties:
 Lowest Vibrational Mode (1/cm) =                          18.1237
 2nd Lowest Vibrational Mode (1/cm) =                      20.8667
 E(RB-P86) (a.u.) =                                      -4635.22035687
 Thermal correction to Enthalpy (a.u.) =                   0.694725
 Thermal correction to Gibbs Free Energy (a.u.) =          0.563971
 Total Entropy (cal/Kmol) =                                275.196
 E(RPBE1PBE) (a.u.) =                                    -4634.56217412
Optimised cartesian coordinates (Angstrom):
 Fe-2.265216  -1.992967  -1.591650
 Mn1.256574  -0.540372   1.272782
 P     -0.327951   0.476408   0.145101
 O      1.068814   1.102423   3.733008
 O     -0.483436  -2.489749   2.615334
 N      1.777591  -1.684562  -0.187105
 N      2.925591   0.489642   0.628592
 C     -0.824751  -0.562152  -1.293946
 C     -0.210404  -1.835342  -1.662731
 C     -0.740329  -2.208041  -2.952761
 H     -0.497711  -3.125221  -3.504675
 C     -1.670681  -1.198020  -3.383132
 H     -2.252507  -1.211088  -4.314791
 C     -1.732096  -0.186693  -2.363483
 H     -2.351679   0.718860  -2.394041
```

```
 C     -2.969987  -2.756557   0.188202
 H     -2.460960  -2.687069   1.158210
 C     -2.797807  -3.808998  -0.777808
 H     -2.141669  -4.683220  -0.669992
 C     -3.617636  -3.503332  -1.922690
 H     -3.693147  -4.101095  -2.840963
 C     -4.300504  -2.261545  -1.661693
 H     -4.987590  -1.747300  -2.347127
 C     -3.898438  -1.798002  -0.358824
 H     -4.223830  -0.868992   0.127277
 C      0.290523   2.029329  -0.655638
 C      0.468426   3.180756   0.144017
 H      0.168291   3.163665   1.204620
 C      1.016697   4.363287  -0.378899
 H      1.127235   5.239086   0.275864
 C      1.419741   4.409365  -1.734823
 C      1.262123   3.257417  -2.541288
 H      1.578526   3.304684  -3.594773
 C      0.707398   2.087517  -2.007238
 H      0.584209   1.210662  -2.661268
 C     -1.907753   1.049768   0.921431
 C     -2.352101   0.513642   2.153514
 H     -1.745227  -0.235732   2.680879
 C     -3.564460   0.918680   2.722484
 H     -3.906358   0.503108   3.682838
 C     -4.375068   1.881760   2.073999
 C     -3.941113   2.433304   0.845796
 H     -4.539530   3.191057   0.320879
 C     -2.719524   2.017718   0.289012
 H     -2.393636   2.477168  -0.657476
 C      0.811098  -2.609245  -0.819538
 H      0.257581  -3.071715   0.022187
 C      2.757555  -1.153498  -1.124507
 H      3.490038  -1.922355  -1.469841
 H      2.269746  -0.781096  -2.064238
 C      3.496996  -0.007892  -0.505503
 C      3.523325   1.550734   1.238679
 C      4.695159   2.137873   0.757655
 C      5.291738   1.618523  -0.407768
 C      4.681498   0.530255  -1.042242
 C      1.472617  -3.761519  -1.599081
 H      0.718883  -4.528454  -1.869447
 H      1.958706  -3.423561  -2.537264
 H      2.235741  -4.240742  -0.952754
 C      1.138444   0.447468   2.748263
 C      0.170818  -1.695595   2.024925
 H      2.731710  -2.848622   0.933116
 O      3.208004  -3.578910   1.426319
 C      4.586290  -3.241416   1.506271
 H      4.731635  -2.213716   1.924702
 H      5.068044  -3.237912   0.494594
 H      5.110118   0.088473  -1.954562
 H      5.134384   2.990738   1.295702
 H      3.035547   1.927839   2.148901
 C      5.302066  -4.251530   2.396451
 H      6.384507  -4.018913   2.471529
 H      4.872413  -4.245390   3.420303
 H      5.195757  -5.278922   1.988603
 H      6.217775   2.056478  -0.810653
 O     -5.531692   2.216068   2.704684
 C     -6.389550   3.183616   2.099862
 H     -7.256736   3.293619   2.776967
 H     -5.884639   4.168608   1.992266
 H     -6.747246   2.847239   1.101984
 O      1.961003   5.499708  -2.342923
 C      2.139981   6.692613  -1.580607
 H      2.585073   7.435288  -2.268441
 H      1.171697   7.085133  -1.199188
 H      2.829779   6.532350  -0.723035
--------------------------------------
Mn21/iii
Frequencies, energies and thermodynamic properties:
 Lowest Vibrational Mode (1/cm) =                        18.5324
 2nd Lowest Vibrational Mode (1/cm) =                    23.9624
 E(RB-P86) (a.u.) =                                     -4636.39304121
 Thermal correction to Enthalpy (a.u.) =                 0.710725
 Thermal correction to Gibbs Free Energy (a.u.) =        0.580693
 Total Entropy (cal/Kmol) =                              273.676
 E(RPBE1PBE) (a.u.) =                                   -4635.73193942
Optimised cartesian coordinates (Angstrom):
 Fe-2.373222  -1.784664  -1.674770
 Mn1.143679  -0.607024   1.498042
 P     -0.250088   0.495998   0.074799
 O      0.640231   1.405229   3.609231
 O     -0.920655  -2.355393   2.659543
```

N	1.645558	-1.930722	-0.039081
N	2.852599	0.307548	0.770818
C	-0.783756	-0.527202	-1.348335
C	-0.312149	-1.881470	-1.619013
C	-0.809447	-2.246676	-2.925134
H	-0.651586	-3.211397	-3.423748
C	-1.583611	-1.155747	-3.454809
H	-2.109515	-1.145019	-4.419132
C	-1.579525	-0.095897	-2.484309
H	-2.081636	0.874367	-2.589576
C	-3.283119	-2.361138	0.082553
H	-2.837675	-2.295060	1.083235
C	-3.168976	-3.479602	-0.815135
H	-2.627947	-4.414471	-0.616193
C	-3.866774	-3.148165	-2.031555
H	-3.947542	-3.784159	-2.923326
C	-4.415781	-1.823948	-1.882901
H	-4.988907	-1.274791	-2.642061
C	-4.052797	-1.335613	-0.577729
H	-4.299501	-0.349622	-0.163010
C	0.546839	1.968668	-0.725867
C	0.808547	3.106621	0.069753
H	0.490369	3.124346	1.124934
C	1.460052	4.236773	-0.451298
H	1.635060	5.103558	0.201359
C	1.880423	4.242742	-1.802247
C	1.633895	3.104608	-2.605995
H	1.962601	3.120136	-3.656623
C	0.978574	1.987393	-2.073001
H	0.790083	1.121607	-2.726162
C	-1.811859	1.256837	0.730301
C	-2.401420	0.814214	1.931375
H	-1.922367	0.023588	2.524938
C	-3.608917	1.357922	2.406532
H	-4.028071	0.983649	3.350925
C	-4.258427	2.375233	1.672043
C	-3.674117	2.834037	0.465249
H	-4.181630	3.634129	-0.095457
C	-2.471659	2.285899	0.009941
H	-2.031074	2.678447	-0.919784
C	0.579448	-2.717785	-0.696601
H	-0.059075	-3.096980	0.128336
C	2.547903	-1.309583	-1.004721
H	3.218412	-2.044951	-1.513010
H	1.997877	-0.801529	-1.844565
C	3.387684	-0.251773	-0.350226
C	3.528119	1.314308	1.382913
C	4.756071	1.795510	0.918286
C	5.321324	1.212475	-0.229841
C	4.625620	0.177175	-0.866670
C	1.131993	-3.963570	-1.424563
H	0.307144	-4.631097	-1.748241
H	1.721964	-3.699702	-2.326969
H	1.782882	-4.532122	-0.730332
C	0.854978	0.597368	2.774609
C	-0.132019	-1.637704	2.149900
H	2.270281	-1.134988	2.702615
H	2.245705	-1.809247	2.218960
H	2.479177	-3.021680	0.807476
O	2.942773	-3.701844	1.458311
C	4.347640	-3.623319	1.305839
H	4.660379	-3.846421	0.252497
H	4.731132	-2.592429	1.526513
H	5.029853	-0.309498	-1.767042
H	5.259221	2.611145	1.457859
H	3.061775	1.739835	2.283483
C	5.027184	-4.616924	2.245396
H	4.689824	-5.652465	2.027759
H	6.131644	-4.581516	2.137961
H	4.774327	-4.393758	3.303631
H	6.290746	1.559371	-0.619601
O	2.518344	5.280692	-2.408266
C	2.792416	6.455912	-1.646778
H	3.308272	7.154655	-2.331175
H	1.858023	6.932622	-1.277078
H	3.456108	6.238764	-0.781121
O	-5.422036	2.973496	2.040051
C	-6.055321	2.556222	3.249597
H	-6.971745	3.167416	3.345329
H	-6.336650	1.480876	3.214518
H	-5.405396	2.733266	4.134390

Mn21/iv

Frequencies, energies and thermodynamic properties:

Lowest Vibrational Mode (1/cm) = 17.1985

```
 2nd Lowest Vibrational Mode (1/cm) =                      20.9036
 E(RB-P86) (a.u.) =                                      -4636.42089344
 Thermal correction to Enthalpy (a.u.) =                   0.715515
 Thermal correction to Gibbs Free Energy (a.u.) =          0.585286
 Total Entropy (cal/Kmol) =                                274.090
 E(RPBE1PBE) (a.u.) =                                    -4635.75812431
Optimised cartesian coordinates (Angstrom):
 Fe-2.528634  -1.553027  -1.740473
 Mn1.181685  -0.757116   1.316961
 P     -0.230429   0.525148   0.060440
 O      1.014878   0.966862   3.691888
 O     -0.794111  -2.573881   2.522321
 N      1.557417  -2.003537  -0.398253
 N      2.908985   0.092505   0.594913
 C     -0.890631  -0.359993  -1.415992
 C     -0.482557  -1.702500  -1.830125
 C     -1.069098  -1.947594  -3.128829
 H     -0.973548  -2.870551  -3.714306
 C     -1.837732  -0.796041  -3.515409
 H     -2.422764  -0.693486  -4.439259
 C     -1.736164   0.178800  -2.465317
 H     -2.214137   1.166804  -2.457844
 C     -3.322836  -2.256698   0.024487
 H     -2.797007  -2.294040   0.987541
 C     -3.322247  -3.292294  -0.975240
 H     -2.805961  -4.259136  -0.903460
 C     -4.098975  -2.830245  -2.097335
 H     -4.272909  -3.379737  -3.032233
 C     -4.583248  -1.508819  -1.788864
 H     -5.190939  -0.874646  -2.448259
 C     -4.102006  -1.152808  -0.479367
 H     -4.275653  -0.200495   0.038084
 C      0.566830   2.028521  -0.692894
 C      0.967643   3.067839   0.177318
 H      0.736097   2.999207   1.253166
 C      1.651443   4.202964  -0.290121
 H      1.933906   4.990638   0.422551
 C      1.969008   4.313465  -1.664171
 C      1.588672   3.273364  -2.543943
 H      1.839873   3.368060  -3.611833
 C      0.899813   2.151677  -2.062571
 H      0.605556   1.366728  -2.776495
 C     -1.732784   1.288796   0.843832
 C     -2.266109   0.760232   2.036372
 H     -1.778148  -0.098017   2.520376
 C     -3.421801   1.299030   2.631860
 H     -3.800361   0.855517   3.563449
 C     -4.071665   2.401070   2.031991
 C     -3.541736   2.947782   0.836913
 H     -4.049288   3.812995   0.382792
 C     -2.391179   2.400815   0.260139
 H     -1.989425   2.859210  -0.657259
 C      0.389599  -2.682698  -1.055558
 H     -0.201797  -3.079154  -0.206329
 C      2.453801  -1.301705  -1.341390
 H      3.032748  -1.998315  -1.985913
 H      1.825896  -0.685989  -2.024049
 C      3.381602  -0.380402  -0.594875
 C      3.672342   0.994076   1.267590
 C      4.916713   1.437294   0.805330
 C      5.414744   0.931601  -0.407043
 C      4.628616   0.007699  -1.112492
 C      0.834171  -3.883972  -1.907582
 H     -0.045250  -4.475661  -2.230954
 H      1.388606  -3.577313  -2.817999
 H      1.482701  -4.552980  -1.306203
 C      1.073931   0.276907   2.728116
 C     -0.039192  -1.821292   1.999420
 H      2.222437  -1.672365   2.124068
 H      2.545868  -3.068646   1.846789
 H      2.105593  -2.764027   0.058087
 O      2.838024  -3.919787   1.390268
 C      4.255489  -4.032150   1.548850
 H      4.596609  -4.793654   0.813962
 H      4.765132  -3.076017   1.278201
 H      4.970928  -0.416215  -2.068746
 H      5.485509   2.166360   1.401255
 H      3.263938   1.359789   2.220711
 C      4.654640  -4.454630   2.962543
 H      4.167935  -5.413429   3.238052
 H      5.754568  -4.588375   3.039638
 H      4.352713  -3.689022   3.708506
 H      6.394421   1.249074  -0.795424
 O      2.628977   5.365921  -2.224087
 C      3.034433   6.444254  -1.383395
```

```
 H      3.541384   7.175522  -2.040162
 H      2.163612   6.936341  -0.896460
 H      3.746183   6.108297  -0.597245
 O     -5.187924   3.003962   2.523776
 C     -5.763263   2.500891   3.728947
 H     -6.647762   3.131362   3.936234
 H     -6.088282   1.443004   3.617951
 H     -5.055352   2.574927   4.583591
---------------------------------------
Mn21/v
Frequencies, energies and thermodynamic properties:
 Lowest Vibrational Mode (1/cm) =                         20.5877
 2nd Lowest Vibrational Mode (1/cm) =                     24.9896
 E(RB-P86) (a.u.) =                                     -4481.48224923
 Thermal correction to Enthalpy (a.u.) =                  0.630860
 Thermal correction to Gibbs Free Energy (a.u.) =         0.514660
 Total Entropy (cal/Kmol) =                               244.563
 E(RPBE1PBE) (a.u.) =                                   -4480.81543358
Optimised cartesian coordinates (Angstrom):
 Fe2.689774  -1.390652   1.333975
 Mn-0.810712  -0.852046  -2.002196
 P     -0.137865   0.258318  -0.126142
 O     -1.244895   1.549746  -3.638255
 O      1.782233  -1.265089  -3.327308
 N     -0.742818  -2.712544  -0.924543
 N     -2.775816  -1.045274  -1.449850
 C      0.729383  -0.836721   1.080932
 C      0.887782  -2.283946   0.928626
 C      1.439921  -2.788761   2.166480
 H      1.697571  -3.833982   2.378291
 C      1.637918  -1.687551   3.068515
 H      2.068594  -1.751340   4.076834
 C      1.208827  -0.487937   2.405458
 H      1.235886   0.523907   2.829797
 C      3.809286  -1.022610  -0.354176
 H      3.406771  -0.873913  -1.364864
 C      4.176259  -2.287210   0.227730
 H      4.111125  -3.267646  -0.263136
 C      4.623068  -2.045724   1.576026
 H      4.951778  -2.809257   2.293811
 C      4.535476  -0.629659   1.826304
 H      4.785339  -0.124941   2.769226
 C      4.030513   0.002721   0.635421
 H      3.825972   1.073497   0.507422
 C     -1.522478   0.964136   0.899260
 C     -2.256094   2.044937   0.358669
 H     -1.946311   2.488435  -0.602204
 C     -3.374465   2.584775   1.016500
 H     -3.906932   3.432510   0.562547
 C     -3.800164   2.029236   2.245787
 C     -3.087077   0.935821   2.789850
 H     -3.426159   0.509320   3.746773
 C     -1.968311   0.415102   2.124867
 H     -1.427351  -0.427603   2.582916
 C      0.973901   1.740951  -0.270505
 C      1.743226   1.941572  -1.433961
 H      1.665976   1.227729  -2.267141
 C      2.620701   3.034604  -1.560832
 H      3.199807   3.152309  -2.487554
 C      2.739179   3.965063  -0.503911
 C      1.963827   3.781268   0.667855
 H      2.055048   4.518281   1.480864
 C      1.095506   2.690175   0.775626
 H      0.489320   2.583480   1.689100
 C      0.555873  -3.130508  -0.294341
 H      1.310431  -2.923709  -1.079200
 C     -1.916310  -2.819887  -0.030738
 H     -2.188807  -3.871919   0.201728
 H     -1.647418  -2.337539   0.935663
 C     -3.090681  -2.084527  -0.621176
 C     -3.800803  -0.297463  -1.940083
 H     -3.519598   0.534994  -2.600967
 C     -5.145160  -0.559033  -1.651823
 H     -5.924517   0.084672  -2.086208
 C     -5.468968  -1.644746  -0.821163
 C     -4.417030  -2.415737  -0.301667
 H     -4.614204  -3.275495   0.356687
 C      0.593266  -4.638969   0.001847
 H      1.618026  -4.950059   0.286260
 H     -0.089047  -4.927469   0.827176
 H      0.313962  -5.216770  -0.903352
 C     -1.061033   0.588901  -2.964009
 C      0.765869  -1.076004  -2.741826
 H     -0.892215  -3.335706  -1.729717
 H     -1.268706  -1.761653  -3.220427
```

```
 H     -6.515834  -1.886847  -0.582995
 O     -4.869379   2.471181   2.966541
 C     -5.623853   3.572022   2.464390
 H     -6.428025   3.758398   3.200437
 H     -5.000709   4.488606   2.367975
 H     -6.082127   3.341214   1.477269
 O      3.549725   5.058168  -0.520700
 C      4.354142   5.296327  -1.674859
 H      4.924967   6.220799  -1.468941
 H      5.066621   4.462001  -1.857758
 H      3.732408   5.448001  -2.584546
---------------------------------------
Mn21/vi_R
Frequencies, energies and thermodynamic properties:
 Lowest Vibrational Mode (1/cm) =                         12.7728
 2nd Lowest Vibrational Mode (1/cm) =                     15.7148
 E(RB-P86) (a.u.) =                                      -4904.19571906
 Thermal correction to Enthalpy (a.u.) =                  0.783471
 Thermal correction to Gibbs Free Energy (a.u.) =         0.644544
 Total Entropy (cal/Kmol) =                               292.396
 E(RPBE1PBE) (a.u.) =                                    -4903.52391141
Optimised cartesian coordinates (Angstrom):
 Fe-2.272530  -2.267788  -1.999842
 Mn1.102139   0.232445   0.293971
 P     -1.095036   0.347741   0.144768
 O      1.219041   0.782311   3.202494
 O      1.214791  -2.633054   0.946308
 N      1.355462   0.022920  -1.641626
 N      1.563927   2.181783  -0.194362
 C     -1.666889  -0.388578  -1.441851
 C     -0.790257  -0.918638  -2.483268
 C     -1.617788  -1.203853  -3.631885
 H     -1.272415  -1.632759  -4.581157
 C     -2.981557  -0.870000  -3.318392
 H     -3.846515  -0.997732  -3.983280
 C     -3.019635  -0.375007  -1.969762
 H     -3.916831  -0.036450  -1.435859
 C     -1.675508  -3.704197  -0.648337
 H     -0.877395  -3.586513   0.095741
 C     -1.505472  -4.179699  -1.995781
 H     -0.558511  -4.494112  -2.454873
 C     -2.790870  -4.147892  -2.646112
 H     -2.994606  -4.429224  -3.688026
 C     -3.757099  -3.654962  -1.697533
 H     -4.826379  -3.494828  -1.890683
 C     -3.068042  -3.377726  -0.463808
 H     -3.516940  -2.969674   0.451158
 C     -1.689328   2.099884   0.038496
 C     -1.699279   2.881015   1.215987
 H     -1.425512   2.422508   2.180390
 C     -2.057291   4.238778   1.196040
 H     -2.061538   4.806777   2.136911
 C     -2.403660   4.854305  -0.030410
 C     -2.383274   4.085847  -1.218495
 H     -2.656262   4.574278  -2.166651
 C     -2.030438   2.730959  -1.181481
 H     -2.036968   2.154173  -2.119038
 C     -2.211701  -0.343010   1.450849
 C     -1.754013  -1.307078   2.371221
 H     -0.708633  -1.644478   2.343131
 C     -2.607347  -1.864268   3.340979
 H     -2.205742  -2.610261   4.040821
 C     -3.958405  -1.455004   3.406132
 C     -4.429275  -0.479825   2.491700
 H     -5.480251  -0.158730   2.558084
 C     -3.567359   0.066747   1.536854
 H     -3.955354   0.839038   0.854407
 C      0.726876  -1.107269  -2.365938
 H      0.905348  -1.994882  -1.726124
 C      1.351405   1.263586  -2.414749
 H      2.060549   1.238813  -3.276122
 H      0.345385   1.460330  -2.869448
 C      1.689900   2.424336  -1.530142
 C      1.819511   3.199324   0.673412
 H      1.705460   2.969027   1.742271
 C      2.213638   4.471413   0.252682
 H      2.410012   5.253088   1.001032
 C      2.356207   4.720467  -1.125771
 C      2.092220   3.679677  -2.023762
 H      2.191205   3.825315  -3.110015
 C      1.379280  -1.407302  -3.729715
 H      0.994566  -2.363541  -4.138277
 H      1.180033  -0.617729  -4.483053
 H      2.476580  -1.511370  -3.609606
 C      1.174277   0.563375   2.039527
```

```
 C      1.112874  -1.487900   0.657385
 H      2.963690  -0.303748  -1.603293
 H      3.284315  -0.041070   0.410502
 C      4.270431  -0.192768  -0.163768
 C      5.013768  -1.316038   0.546870
 C      5.173136   1.041319   0.122639
 C      5.929734  -0.787620   1.483255
 C      4.884419  -2.701275   0.365858
 C      5.876721   0.731283   1.466722
 H      5.920814   1.086209  -0.699594
 C      6.721885  -1.650645   2.259198
 C      5.681019  -3.566447   1.142043
 H      4.175690  -3.098325  -0.378356
 H      6.876778   1.203519   1.564978
 C      6.591938  -3.043914   2.083224
 H      7.444769  -1.248551   2.988559
 H      5.597661  -4.657543   1.010104
 H      7.213943  -3.730410   2.680382
 O      3.984897  -0.422499  -1.509261
 H      4.611626   1.997205   0.121351
 H      5.268088   1.100798   2.324176
 H      2.670521   5.710370  -1.490485
 O     -2.764723   6.159174  -0.164402
 C     -2.806924   6.982904   1.000188
 H     -3.121358   7.986264   0.657871
 H     -3.542932   6.605171   1.743571
 H     -1.808863   7.060453   1.484710
 O     -4.866633  -1.922828   4.302255
 C     -4.450715  -2.902743   5.253716
 H     -5.341017  -3.135255   5.866775
 H     -4.097959  -3.832616   4.756197
 H     -3.644589  -2.515402   5.914574
---------------------------------------
Mn21/vi_S
Frequencies, energies and thermodynamic properties:
 Lowest Vibrational Mode (1/cm) =                        14.0069
 2nd Lowest Vibrational Mode (1/cm) =                    17.0727
 E(RB-P86) (a.u.) =                                     -4904.19600608
 Thermal correction to Enthalpy (a.u.) =                 0.783536
 Thermal correction to Gibbs Free Energy (a.u.) =        0.644803
 Total Entropy (cal/Kmol) =                              291.990
 E(RPBE1PBE) (a.u.) =                                   -4903.52436389
Optimised cartesian coordinates (Angstrom):
 Fe-3.105578  -1.739909  -1.731653
 Mn0.953450  -0.397467   0.417603
 P     -1.017835   0.532943   0.086188
 O      1.237072   0.531409   3.216448
 O     -0.071459  -2.935800   1.490433
 N      1.126142  -0.999924  -1.441333
 N      2.158786   1.108758  -0.312194
 C     -1.814158  -0.177591  -1.413118
 C     -1.205091  -1.170533  -2.294524
 C     -2.063871  -1.300012  -3.448130
 H     -1.903821  -1.980803  -4.293883
 C     -3.189015  -0.417011  -3.293138
 H     -4.026228  -0.308843  -3.995939
 C     -3.045183   0.270702  -2.039465
 H     -3.742568   1.013662  -1.631692
 C     -3.136752  -3.058839  -0.147915
 H     -2.362195  -3.147972   0.624619
 C     -3.164752  -3.772113  -1.397317
 H     -2.420008  -4.502794  -1.740595
 C     -4.328098  -3.342301  -2.130919
 H     -4.622709  -3.683806  -3.132343
 C     -5.021194  -2.363747  -1.331943
 H     -5.936905  -1.829214  -1.618377
 C     -4.283924  -2.185793  -0.107863
 H     -4.536494  -1.492844   0.705243
 C     -0.874228   2.335161  -0.321796
 C     -0.585239   3.241194   0.723488
 H     -0.517840   2.877313   1.761977
 C     -0.384622   4.609140   0.477757
 H     -0.171861   5.280621   1.321361
 C     -0.456418   5.101178  -0.847294
 C     -0.729832   4.200894  -1.904220
 H     -0.784764   4.594690  -2.930864
 C     -0.933939   2.840163  -1.642589
 H     -1.159082   2.165269  -2.482574
 C     -2.332143   0.553378   1.390883
 C     -2.300269  -0.344043   2.476987
 H     -1.469055  -1.055289   2.580828
 C     -3.316958  -0.361377   3.449137
 H     -3.248820  -1.075986   4.281169
 C     -4.401579   0.538795   3.347153
 C     -4.441233   1.452404   2.264248
```

```
 H     -5.284141   2.157690   2.200076
 C     -3.420890   1.459390   1.309160
 H     -3.466972   2.195204   0.491110
 C      0.115146  -1.905828  -2.036197
 H     -0.075558  -2.674891  -1.260802
 C      1.614082   0.003704  -2.385877
 H      2.262207  -0.431359  -3.183287
 H      0.769260   0.497609  -2.933558
 C      2.375408   1.068771  -1.657614
 C      2.789978   2.070563   0.415400
 H      2.592510   2.072371   1.496832
 C      3.649766   3.010197  -0.157472
 H      4.133461   3.762976   0.481993
 C      3.881654   2.965703  -1.545336
 C      3.237059   1.978105  -2.299555
 H      3.388318   1.901962  -3.386909
 C      0.615722  -2.654899  -3.286644
 H     -0.106180  -3.444854  -3.576739
 H      0.749894  -1.986549  -4.161808
 H      1.584247  -3.148678  -3.068351
 C      1.127443   0.160682   2.097182
 C      0.282472  -1.901753   1.029293
 H      2.499001  -1.892901  -1.248850
 H      2.893357  -1.425481   0.717824
 C      3.674913  -2.147054   0.279320
 C      3.641936  -3.440163   1.143914
 C      4.551913  -3.139826   2.360365
 O      3.419831  -2.325340  -1.080503
 H      4.557106   3.688718  -2.027743
 C      5.041077  -1.564032   0.610478
 C      5.540093  -2.126148   1.806815
 C      5.780759  -0.607723  -0.101729
 C      6.789391  -1.722702   2.307503
 C      7.035255  -0.206080   0.399272
 H      5.386787  -0.192247  -1.043441
 C      7.534123  -0.758429   1.596931
 H      7.191530  -2.159829   3.236700
 H      7.634077   0.538629  -0.149905
 H      8.519205  -0.440980   1.975858
 H      5.043386  -4.041438   2.782630
 H      3.963210  -2.684654   3.190145
 H      4.088393  -4.248526   0.523809
 H      2.613197  -3.748475   1.416284
 O     -0.279748   6.403922  -1.197387
 C     -0.006822   7.359150  -0.172858
 H      0.095600   8.336189  -0.680470
 H     -0.837000   7.418048   0.565024
 H      0.939614   7.127228   0.363259
 O     -5.432760   0.611260   4.229309
 C     -5.446551  -0.280491   5.344268
 H     -6.364761  -0.047649   5.914570
 H     -5.479766  -1.343121   5.018126
 H     -4.561874  -0.128998   6.000805
---------------------------------------
Mn21/viii
Frequencies, energies and thermodynamic properties:
 Lowest Vibrational Mode (1/cm) =                         21.6040
 2nd Lowest Vibrational Mode (1/cm) =                     26.6023
 E(RB-P86) (a.u.) =                                     -4481.44904466
 Thermal correction to Enthalpy (a.u.) =                  0.626071
 Thermal correction to Gibbs Free Energy (a.u.) =         0.509757
 Total Entropy (cal/Kmol) =                               244.804
 E(RPBE1PBE) (a.u.) =                                   -4480.78250660
Optimised cartesian coordinates (Angstrom):
 Fe2.641368  -1.406084   1.375705
 Mn-0.845249  -1.032602  -2.009575
 P     -0.102901   0.210445  -0.270210
 O     -1.053436   1.349655  -3.760657
 O      1.814177  -1.612195  -3.128055
 N     -0.899563  -2.662201  -0.764676
 N     -2.830743  -0.971887  -1.417131
 C      0.709306  -0.788631   1.036829
 C      0.780154  -2.245249   1.040187
 C      1.301064  -2.638927   2.328034
 H      1.498505  -3.669195   2.651382
 C      1.561050  -1.458463   3.110203
 H      1.981976  -1.434443   4.124716
 C      1.206934  -0.313526   2.316045
 H      1.288799   0.735600   2.628179
 C      3.817086  -1.304704  -0.317077
 H      3.449154  -1.254614  -1.349880
 C      4.094847  -2.509715   0.417792
 H      3.980799  -3.535633   0.042473
 C      4.524708  -2.136275   1.741781
 H      4.792499  -2.827015   2.552684
```

```
 C      4.515517  -0.697233   1.822993
 H      4.776308  -0.099821   2.707060
 C      4.075013  -0.182893   0.552061
 H      3.939148   0.874987   0.292262
 C     -1.468768   1.075098   0.643132
 C     -2.124809   2.159588   0.005266
 H     -1.785807   2.501231  -0.986343
 C     -3.194351   2.818669   0.616629
 H     -3.696737   3.665604   0.124245
 C     -3.655860   2.403990   1.891281
 C     -3.021818   1.318082   2.533541
 H     -3.353563   0.971047   3.522208
 C     -1.941190   0.667776   1.906261
 H     -1.456744  -0.169712   2.431340
 C      1.060024   1.627056  -0.574636
 C      1.867159   1.682298  -1.735770
 H      1.792221   0.896330  -2.499875
 C      2.776883   2.726416  -1.937408
 H      3.400247   2.767056  -2.843890
 C      2.908698   3.758809  -0.977746
 C      2.104595   3.723423   0.185102
 H      2.172153   4.513038   0.946639
 C      1.193408   2.669290   0.369767
 H      0.564672   2.676069   1.273922
 C      0.319786  -3.144111  -0.117947
 H      1.114151  -3.105837  -0.892855
 C     -2.052933  -2.685302   0.106270
 H     -2.426510  -3.719944   0.323838
 H     -1.864721  -2.246267   1.132533
 C     -3.185310  -1.890506  -0.475432
 C     -3.798645  -0.187232  -1.959543
 C     -5.145321  -0.285829  -1.599234
 C     -5.520386  -1.239143  -0.633994
 C     -4.525765  -2.047393  -0.070757
 C      0.224162  -4.621181   0.327891
 H      1.216748  -5.010619   0.635804
 H     -0.468728  -4.761791   1.183731
 H     -0.140347  -5.240172  -0.517357
 C     -0.980120   0.384800  -3.079988
 C      0.774088  -1.358312  -2.623456
 H     -1.388345  -2.464468  -2.897889
 H     -1.555353  -1.875906  -3.439391
 H     -3.470281   0.540759  -2.715780
 H     -5.885290   0.373829  -2.075731
 H     -4.772313  -2.808583   0.685072
 H     -6.572802  -1.350099  -0.330199
 O      3.814658   4.732244  -1.261634
 C      3.992508   5.797317  -0.328332
 H      4.767939   6.458432  -0.757649
 H      3.055631   6.379671  -0.186993
 H      4.339991   5.422820   0.659557
 O     -4.701740   3.106165   2.406736
 C     -5.208503   2.736064   3.687928
 H     -6.041022   3.430950   3.904503
 H     -5.595136   1.693111   3.691004
 H     -4.434486   2.837499   4.480365
---------------------------------------
Mn21/ix
Frequencies, energies and thermodynamic properties:
 Lowest Vibrational Mode (1/cm) =                        19.2186
 2nd Lowest Vibrational Mode (1/cm) =                    25.3513
 E(RB-P86) (a.u.) =                                     -4635.23664746
 Thermal correction to Enthalpy (a.u.) =                 0.695316
 Thermal correction to Gibbs Free Energy (a.u.) =        0.570063
 Total Entropy (cal/Kmol) =                              263.616
 E(RPBE1PBE) (a.u.) =                                   -4634.57435235
Optimised cartesian coordinates (Angstrom):
 Fe-2.830342  -1.047242  -1.698858
 Mn0.982289  -1.142368   1.330749
 P     -0.016093   0.393120  -0.000541
 O      1.342045   0.781709   3.526343
 O     -1.505201  -2.079002   2.587438
 N      0.939070  -2.544611  -0.275204
 N      2.861049  -0.821145   0.492434
 C     -0.906116  -0.384296  -1.421611
 C     -0.923786  -1.813193  -1.735795
 C     -1.558991  -1.962563  -3.025219
 H     -1.746443  -2.911209  -3.543851
 C     -1.943605  -0.663590  -3.505669
 H     -2.470456  -0.454607  -4.446438
 C     -1.551711   0.307571  -2.522529
 H     -1.708881   1.391148  -2.595690
 C     -3.807988  -1.330829   0.092132
 H     -3.324428  -1.440694   1.071414
 C     -4.110372  -2.401145  -0.821437
```

```
 H     -3.905614  -3.467751  -0.657311
 C     -4.707355  -1.826885  -2.000278
 H     -5.031809  -2.377430  -2.893511
 C     -4.776655  -0.399880  -1.813328
 H     -5.163033   0.327327  -2.540050
 C     -4.218582  -0.092436  -0.521975
 H     -4.103152   0.909352  -0.088305
 C      1.168746   1.544633  -0.855895
 C      1.797510   2.547880  -0.084749
 H      1.523014   2.676159   0.975316
 C      2.768227   3.402821  -0.633273
 H      3.223506   4.175600   0.002007
 C      3.146368   3.255685  -1.988556
 C      2.534862   2.246077  -2.768185
 H      2.834676   2.140025  -3.822337
 C      1.563067   1.406549  -2.207438
 H      1.095746   0.639960  -2.844873
 C     -1.232340   1.613276   0.695057
 C     -1.865513   1.383409   1.932966
 H     -1.625437   0.483119   2.515149
 C     -2.814809   2.280604   2.456716
 H     -3.279662   2.060376   3.427868
 C     -3.150284   3.448179   1.735462
 C     -2.514588   3.697575   0.493726
 H     -2.772845   4.616022  -0.055847
 C     -1.571152   2.797033  -0.009612
 H     -1.076711   3.030215  -0.965515
 C     -0.360628  -2.943608  -0.880531
 H     -1.032648  -3.093535  -0.011283
 C      2.030301  -2.323061  -1.237577
 H      2.399147  -3.267167  -1.695380
 H      1.640880  -1.701513  -2.076208
 C      3.172534  -1.580897  -0.591093
 C      3.840981  -0.078194   1.062262
 C      5.158056  -0.064231   0.589039
 C      5.487456  -0.859509  -0.521798
 C      4.477297  -1.628199  -1.115824
 C     -0.281398  -4.280767  -1.637142
 H     -1.296810  -4.628366  -1.913989
 H      0.315583  -4.206496  -2.568914
 H      0.172677  -5.058955  -0.990251
 C      1.203006  -0.005958   2.650068
 C     -0.527541  -1.675990   2.050618
 H      1.255641  -3.224336   0.466608
 H      4.687996  -2.266746  -1.987113
 H      5.911088   0.557657   1.095107
 H      3.552063   0.519950   1.938689
 H      6.514924  -0.882647  -0.916280
 O      1.953442  -2.799541   2.021351
 C      1.811883  -3.347890   3.296883
 C      2.656499  -2.652942   4.378507
 H      2.128175  -4.425860   3.261105
 H      0.743343  -3.370862   3.647895
 H      2.556655  -3.167571   5.359909
 H      3.731762  -2.656443   4.096287
 H      2.344606  -1.596268   4.511950
 O      4.075097   4.025968  -2.620378
 C      4.726675   5.056864  -1.880599
 H      5.428107   5.545580  -2.582132
 H      4.003255   5.813586  -1.504486
 H      5.300478   4.645947  -1.020774
 O     -4.047830   4.382931   2.148000
 C     -4.716925   4.187520   3.393522
 H     -5.391035   5.054588   3.521878
 H     -5.321098   3.253760   3.392072
 H     -4.000217   4.154869   4.243295
---------------------------------------
Mn21/x
Frequencies, energies and thermodynamic properties:
 Lowest Vibrational Mode (1/cm) =                        15.4064
 2nd Lowest Vibrational Mode (1/cm) =                    21.6628
 E(RB-P86) (a.u.) =                                     -4790.17828548
 Thermal correction to Enthalpy (a.u.) =                 0.779397
 Thermal correction to Gibbs Free Energy (a.u.) =        0.640065
 Total Entropy (cal/Kmol) =                              293.249
 E(RPBE1PBE) (a.u.) =                                   -4789.52404942
Optimised cartesian coordinates (Angstrom):
 Fe-2.523960  -1.930920  -1.707759
 Mn1.209417  -0.421045   1.077079
 P     -0.537393   0.525679  -0.019911
 O      0.715313   1.357790   3.366051
 O     -0.346526  -2.566597   2.351260
 N      1.664644  -1.574282  -0.687006
 N      2.620205   0.801344   0.164350
 C     -1.120399  -0.454333  -1.466472
```

C	-0.498374	-1.687158	-1.943947
C	-1.121101	-2.022743	-3.204311
H	-0.895245	-2.902929	-3.819173
C	-2.119740	-1.033400	-3.505022
H	-2.780025	-1.031100	-4.382645
C	-2.128812	-0.071363	-2.438173
H	-2.782877	0.807469	-2.372882
C	-3.056268	-2.781763	0.091073
H	-2.477574	-2.720231	1.021735
C	-2.914586	-3.794327	-0.921868
H	-2.215993	-4.641437	-0.894998
C	-3.833259	-3.489690	-1.988989
H	-3.953409	-4.059803	-2.919958
C	-4.545861	-2.289055	-1.633117
H	-5.304185	-1.783861	-2.246263
C	-4.064391	-1.849416	-0.349275
H	-4.389113	-0.950965	0.191410
C	-0.126219	2.170348	-0.785375
C	0.067290	3.276763	0.072219
H	-0.096140	3.165230	1.156667
C	0.458518	4.533452	-0.419300
H	0.587490	5.367999	0.284151
C	0.681934	4.705687	-1.805601
C	0.503954	3.603000	-2.673657
H	0.677734	3.747104	-3.751297
C	0.106171	2.358175	-2.168293
H	-0.039366	1.524787	-2.872950
C	-2.094314	0.953539	0.899290
C	-2.421062	0.327447	2.118898
H	-1.736951	-0.409576	2.561573
C	-3.617800	0.615194	2.800798
H	-3.827606	0.104114	3.750763
C	-4.524403	1.555660	2.262421
C	-4.205632	2.198587	1.040540
H	-4.912593	2.938069	0.633637
C	-3.010221	1.903319	0.378558
H	-2.779420	2.435614	-0.557352
C	0.605753	-2.475598	-1.255234
H	0.173372	-2.978207	-0.367149
C	2.329817	-0.728543	-1.700997
H	2.998313	-1.310609	-2.371768
H	1.549853	-0.274530	-2.353090
C	3.096935	0.385498	-1.039259
C	3.228749	1.847958	0.772423
C	4.327181	2.515708	0.217858
C	4.833142	2.078868	-1.017643
C	4.206962	0.995845	-1.650809
C	1.206354	-3.572180	-2.151869
H	0.436291	-4.327900	-2.405508
H	1.610398	-3.169246	-3.103174
H	2.023242	-4.087000	-1.607358
C	0.925526	0.633577	2.452044
C	0.234782	-1.685694	1.814311
H	3.365804	-2.488756	1.143436
H	2.399572	-2.223630	-0.304960
O	3.617818	-3.284320	0.487463
C	4.970994	-3.129867	0.085433
H	5.088565	-3.577537	-0.929662
H	5.241167	-2.048869	-0.015719
H	4.568536	0.616671	-2.618485
H	4.778967	3.360654	0.758021
H	2.815045	2.156557	1.743083
C	5.949636	-3.810034	1.047539
H	5.708053	-4.889165	1.149915
H	6.996671	-3.722523	0.685382
H	5.898572	-3.352781	2.058673
H	5.702844	2.571421	-1.478939
O	2.920759	-1.306627	1.837457
C	3.123959	-1.502789	3.217414
C	3.917397	-0.372331	3.886952
H	3.689587	-2.460540	3.366493
H	2.158960	-1.640481	3.769400
H	4.121602	-0.610519	4.954124
H	4.891614	-0.220485	3.375096
H	3.359033	0.585870	3.854676
O	1.063346	5.875757	-2.388227
C	1.256019	7.022037	-1.561181
H	1.556619	7.844238	-2.236888
H	0.320411	7.309893	-1.032837
H	2.059981	6.856922	-0.810403
O	-5.703462	1.912550	2.837995
C	-6.076974	1.300486	4.072262
H	-7.056847	1.732698	4.347459
H	-6.181571	0.198191	3.967928
H	-5.342815	1.521019	4.878003

```
---------------------------------------
Mn21/TS-i
Frequencies, energies and thermodynamic properties:
 Lowest Vibrational Mode (1/cm) =-751.8612
 2nd Lowest Vibrational Mode (1/cm) =                    18.7479
 E(RB-P86) (a.u.) =                                    -4636.39080395
 Thermal correction to Enthalpy (a.u.) =                 0.706980
 Thermal correction to Gibbs Free Energy (a.u.) =        0.578681
 Total Entropy (cal/Kmol) =                              270.027
 E(RPBE1PBE) (a.u.) =                                  -4635.72702857
Optimised cartesian coordinates (Angstrom):
 Fe-2.249892  -1.873073  -1.691566
 Mn1.250580  -0.657334   1.478231
 P     -0.284047   0.442721   0.203650
 O      0.667026   1.070671   3.801923
 O     -0.605645  -2.684042   2.534014
 N      1.818452  -1.782098  -0.206766
 N      2.850971   0.466404   0.801545
 C     -0.777962  -0.497800  -1.292868
 C     -0.191683  -1.768266  -1.710068
 C     -0.702485  -2.057516  -3.029920
 H     -0.472941  -2.950660  -3.624766
 C     -1.597499  -1.003351  -3.426004
 H     -2.160765  -0.955044  -4.367737
 C     -1.656014  -0.044557  -2.357257
 H     -2.253274   0.876336  -2.351758
 C     -3.024144  -2.716005   0.023179
 H     -2.543654  -2.715378   1.009853
 C     -2.849761  -3.717281  -0.995179
 H     -2.218126  -4.612573  -0.919039
 C     -3.628671  -3.326955  -2.142760
 H     -3.691288  -3.869576  -3.095597
 C     -4.288228  -2.084256  -1.830892
 H     -4.942064  -1.514738  -2.505034
 C     -3.912474  -1.704309  -0.493839
 H     -4.229348  -0.796214   0.035238
 C      0.353988   2.043009  -0.486415
 C      0.569803   3.120696   0.411470
 H      0.306980   3.011326   1.476279
 C      1.101032   4.334941  -0.030026
 H      1.259061   5.172790   0.666453
 C      1.450475   4.510056  -1.392771
 C      1.253816   3.443002  -2.296378
 H      1.512277   3.544715  -3.359679
 C      0.710624   2.227234  -1.836970
 H      0.554241   1.416519  -2.565231
 C     -1.882287   1.001455   0.964635
 C     -2.401311   0.385591   2.128321
 H     -1.844040  -0.420817   2.624502
 C     -3.628936   0.780882   2.671004
 H     -4.026982   0.301647   3.578590
 C     -4.382378   1.814640   2.064277
 C     -3.875130   2.445712   0.904593
 H     -4.427361   3.258803   0.412833
 C     -2.638755   2.038644   0.374764
 H     -2.256031   2.559826  -0.516660
 C      0.799509  -2.607017  -0.906019
 H      0.234577  -3.112001  -0.095652
 C      2.630713  -0.981984  -1.130009
 H      3.336934  -1.600371  -1.731883
 H      1.988877  -0.452812  -1.879993
 C      3.398086   0.071302  -0.382076
 C      3.462277   1.457532   1.500418
 C      4.634128   2.083056   1.062839
 C      5.211132   1.669033  -0.150281
 C      4.581877   0.649778  -0.876896
 C      1.441152  -3.723053  -1.755584
 H      0.671788  -4.438597  -2.110333
 H      1.969400  -3.327846  -2.647819
 H      2.166961  -4.282311  -1.131524
 C      0.910988   0.377443   2.877141
 C      0.098130  -1.855387   2.069570
 H      2.460286  -1.245017   2.534746
 H      2.532004  -1.936321   2.031487
 H      2.595649  -2.614083   0.445486
 O      3.186413  -3.317200   1.232584
 C      4.583220  -3.162319   1.178293
 H      4.962353  -3.191747   0.121460
 H      4.909774  -2.163021   1.581355
 H      4.998028   0.291892  -1.830588
 H      5.085655   2.878005   1.674181
 H      2.990955   1.747991   2.450525
 C      5.289445  -4.259181   1.981002
 H      5.023091  -5.262840   1.586080
 H      6.394528  -4.149820   1.936806
```

```
 H      4.981058  -4.224625   3.047825
 H      6.138419   2.131844  -0.521497
 O     -5.559015   2.133205   2.665475
 C     -6.361240   3.169567   2.099706
 H     -7.257159   3.252328   2.742410
 H     -5.826543   4.144650   2.093576
 H     -6.678642   2.922393   1.062919
 O      1.960834   5.725984  -1.728009
 C      2.329636   5.962809  -3.085856
 H      2.713699   6.998975  -3.126143
 H      3.128379   5.265497  -3.421819
 H      1.457345   5.871326  -3.769905
---------------------------------------
Mn21/TS-ii_si
Frequencies, energies and thermodynamic properties:
 Lowest Vibrational Mode (1/cm) =-229.7629
 2nd Lowest Vibrational Mode (1/cm) =                     9.0870
 E(RB-P86) (a.u.) =                                      -4904.18959648
 Thermal correction to Enthalpy (a.u.) =                  0.780120
 Thermal correction to Gibbs Free Energy (a.u.) =         0.643442
 Total Entropy (cal/Kmol) =                               287.662
 E(RPBE1PBE) (a.u.) =                                    -4903.51204627
Optimised cartesian coordinates (Angstrom):
 Fe-2.275118  -2.202482  -2.019096
 Mn1.252560   0.184132   0.325975
 P     -0.996635   0.391027   0.121073
 O      1.379950   0.706017   3.218740
 O      1.344443  -2.699506   0.894092
 N      1.461862  -0.030978  -1.759198
 N      1.684338   2.118338  -0.197984
 C     -1.619094  -0.336267  -1.456401
 C     -0.768341  -0.891760  -2.507326
 C     -1.615333  -1.150099  -3.651502
 H     -1.291783  -1.584247  -4.605786
 C     -2.964955  -0.781636  -3.322606
 H     -3.838768  -0.883872  -3.980048
 C     -2.974267  -0.288256  -1.972781
 H     -3.856061   0.071730  -1.427439
 C     -1.631399  -4.152491  -2.158796
 H     -0.779434  -4.505496  -2.755442
 C     -2.992017  -4.031219  -2.619222
 H     -3.360180  -4.274014  -3.625008
 C     -3.783484  -3.514625  -1.530354
 H     -4.858536  -3.291834  -1.561085
 C     -2.913185  -3.308855  -0.404427
 H     -3.204607  -2.899800   0.571299
 C     -1.580437  -3.704002  -0.791722
 H     -0.681724  -3.647221  -0.163289
 C     -1.594846   2.148720   0.021701
 C     -1.525242   2.936587   1.193050
 H     -1.194534   2.479157   2.140232
 C     -1.876071   4.297033   1.191850
 H     -1.816585   4.867532   2.129455
 C     -2.295512   4.911462  -0.011511
 C     -2.353495   4.138917  -1.194993
 H     -2.681228   4.625741  -2.126645
 C     -2.007652   2.781005  -1.174950
 H     -2.077886   2.204455  -2.110359
 C     -2.122503  -0.284132   1.433919
 C     -1.664713  -1.259232   2.342337
 H     -0.625190  -1.612394   2.287784
 C     -2.510032  -1.810016   3.323429
 H     -2.108834  -2.566883   4.011871
 C     -3.853193  -1.380362   3.413632
 C     -4.324255  -0.393496   2.512123
 H     -5.369295  -0.057511   2.597656
 C     -3.469782   0.144784   1.545286
 H     -3.856916   0.926196   0.872562
 C      0.732864  -1.150774  -2.442724
 H      0.909771  -2.027139  -1.787982
 C      1.318941   1.272609  -2.441813
 H      1.871230   1.312163  -3.405417
 H      0.243433   1.430472  -2.683805
 C      1.768166   2.383993  -1.534353
 C      1.993742   3.120919   0.668789
 H      1.912106   2.880308   1.738358
 C      2.404962   4.391431   0.251511
 H      2.641900   5.156611   1.005393
 C      2.514117   4.656557  -1.124294
 C      2.190617   3.631192  -2.024892
 H      2.256320   3.787933  -3.112145
 C      1.339827  -1.476787  -3.818449
 H      0.914830  -2.420520  -4.214386
 H      1.150282  -0.680684  -4.567080
 H      2.435608  -1.616908  -3.722907
```

```
 C      1.306008   0.497513   2.053039
 C      1.252829  -1.542691   0.648111
 H      2.493416  -0.265068  -1.798536
 H      2.889096  -0.031661   0.354281
 C      4.403071  -0.220533  -0.415551
 C      4.782195  -1.406729   0.430499
 C      5.024687   1.000620   0.304296
 C      5.304489  -0.966502   1.667295
 C      4.732569  -2.770446   0.098332
 C      5.238631   0.547809   1.765166
 H      6.005363   1.177919  -0.194945
 C      5.780490  -1.904680   2.597915
 C      5.212677  -3.707266   1.030823
 H      4.332132  -3.087362  -0.878020
 H      6.139748   0.987081   2.240975
 C      5.730645  -3.275598   2.271559
 H      6.195261  -1.579133   3.566231
 H      5.191850  -4.783205   0.793674
 H      6.107708  -4.020278   2.991192
 O      4.160365  -0.269211  -1.653523
 H      4.425289   1.922961   0.186498
 H      4.367456   0.839830   2.394912
 H      2.845083   5.641167  -1.488126
 O     -2.656676   6.219732  -0.127223
 C     -2.617322   7.045794   1.034841
 H     -2.945032   8.051303   0.711426
 H     -3.306728   6.676252   1.825808
 H     -1.589688   7.116508   1.454762
 O     -4.754089  -1.839573   4.323302
 C     -4.336557  -2.831259   5.261048
 H     -5.218950  -3.053817   5.889190
 H     -4.007383  -3.763832   4.752256
 H     -3.512305  -2.461184   5.909539
---------------------------------------
Mn21/TS-ii_re
Frequencies, energies and thermodynamic properties:
 Lowest Vibrational Mode (1/cm) =-236.2285
 2nd Lowest Vibrational Mode (1/cm) =                      12.2602
 E(RB-P86) (a.u.) =                                      -4904.19032319
 Thermal correction to Enthalpy (a.u.) =                   0.780249
 Thermal correction to Gibbs Free Energy (a.u.) =          0.644773
 Total Entropy (cal/Kmol) =                                285.134
 E(RPBE1PBE) (a.u.) =                                    -4903.51395041
Optimised cartesian coordinates (Angstrom):
 Fe-3.118026  -1.639484  -1.684559
 Mn1.100351  -0.589485   0.467309
 P     -0.879237   0.474010   0.153165
 O      1.420913   0.180781   3.289333
 O      0.044032  -3.182158   1.362539
 N      1.172263  -1.124188  -1.569692
 N      2.266659   0.928929  -0.268933
 C     -1.763400  -0.135088  -1.345615
 C     -1.226536  -1.112966  -2.291633
 C     -2.130237  -1.159435  -3.418906
 H     -2.028289  -1.806700  -4.298969
 C     -3.214182  -0.245046  -3.182872
 H     -4.072155  -0.078480  -3.848035
 C     -2.997574   0.381452  -1.908452
 H     -3.651672   1.128306  -1.440407
 C     -4.249122  -2.118687  -0.035805
 H     -4.445345  -1.454877   0.816024
 C     -3.134592  -3.025180  -0.159667
 H     -2.333557  -3.173773   0.576172
 C     -3.235560  -3.680756  -1.437023
 H     -2.530758  -4.419909  -1.841180
 C     -4.410956  -3.181151  -2.103804
 H     -4.755469  -3.467632  -3.106537
 C     -5.038692  -2.217757  -1.235743
 H     -5.945819  -1.641284  -1.461748
 C     -0.721171   2.296375  -0.176126
 C     -0.306838   3.128546   0.896552
 H     -0.161298   2.696478   1.900296
 C     -0.086508   4.495743   0.710948
 H      0.225055   5.140250   1.547514
 C     -0.257172   5.080773  -0.568963
 C     -0.652972   4.264827  -1.650795
 H     -0.794774   4.685036  -2.656474
 C     -0.879388   2.889198  -1.444761
 H     -1.201332   2.279326  -2.303157
 C     -2.168124   0.471510   1.490306
 C     -2.149949  -0.510446   2.508846
 H     -1.352577  -1.266835   2.526749
 C     -3.136379  -0.544010   3.500982
 H     -3.117807  -1.308003   4.293495
 C     -4.178763   0.414115   3.508055
```

```
 C     -4.206834   1.408447   2.502568
 H     -4.994550   2.174775   2.484018
 C     -3.206439   1.428483   1.514962
 H     -3.238429   2.224952   0.754762
 C      0.046196  -1.937489  -2.138675
 H     -0.135203  -2.717874  -1.373227
 C      1.551184   0.032677  -2.408518
 H      2.066873  -0.271233  -3.345150
 H      0.623948   0.567584  -2.715922
 C      2.410688   0.986146  -1.624424
 C      2.963281   1.822246   0.482988
 H      2.831896   1.746911   1.571762
 C      3.808333   2.790261  -0.070187
 H      4.339152   3.487117   0.594946
 C      3.965900   2.841894  -1.465235
 C      3.258943   1.917343  -2.247796
 H      3.348885   1.914715  -3.344652
 C      0.452225  -2.654312  -3.437873
 H     -0.322567  -3.390507  -3.730997
 H      0.590008  -1.952881  -4.286016
 H      1.399430  -3.208886  -3.280635
 C      1.281425  -0.115937   2.149582
 C      0.413154  -2.117178   0.986253
 H      2.020402  -1.758333  -1.543336
 H      2.532885  -1.406016   0.572003
 C      3.764111  -2.382806  -0.101425
 C      4.860860  -1.554836   0.510435
 C      3.531330  -3.543025   0.896906
 C      5.077905  -1.958637   1.847640
 C      5.656749  -0.569480  -0.097068
 C      4.084894  -3.036371   2.246499
 C      6.104145  -1.362840   2.598770
 C      6.685236   0.023303   0.657411
 H      5.476493  -0.284728  -1.146136
 C      6.904588  -0.370662   1.995483
 H      6.291357  -1.669107   3.641269
 H      7.329553   0.793290   0.203361
 H      7.717605   0.098120   2.573272
 O      3.522496  -2.447347  -1.339042
 H      4.542843  -3.832882   2.868713
 H      3.269504  -2.582604   2.854790
 H      2.481061  -3.886938   0.926334
 H      4.151186  -4.388265   0.517682
 H      4.629362   3.584354  -1.934329
 O     -5.088680   0.305349   4.513202
 C     -6.162827   1.243042   4.570956
 H     -6.769413   0.964545   5.452577
 H     -5.792580   2.283994   4.698601
 H     -6.800041   1.193476   3.660696
 O     -0.017524   6.418955  -0.652750
 C     -0.170502   7.062202  -1.916397
 H      0.071875   8.128748  -1.752945
 H      0.524916   6.643998  -2.677359
 H     -1.213121   6.980579  -2.295635
---------------------------------------
Mn21/TS-iii
Frequencies, energies and thermodynamic properties:
 Lowest Vibrational Mode (1/cm) =-649.3891
 2nd Lowest Vibrational Mode (1/cm) =                      18.8542
 E(RB-P86) (a.u.) =                                      -4481.44575440
 Thermal correction to Enthalpy (a.u.) =                   0.625059
 Thermal correction to Gibbs Free Energy (a.u.) =          0.508981
 Total Entropy (cal/Kmol) =                                244.307
 E(RPBE1PBE) (a.u.) =                                    -4480.77627764
Optimised cartesian coordinates (Angstrom):
 Fe2.701082  -1.384959   1.323316
 Mn-0.767411  -0.900241  -2.014528
 P     -0.135123   0.237399  -0.146723
 O     -1.216037   1.492504  -3.691450
 O      1.927970  -1.119882  -3.172012
 N     -0.734719  -2.707241  -0.963435
 N     -2.756740  -1.041593  -1.437919
 C      0.730894  -0.874329   1.035297
 C      0.914650  -2.316134   0.865532
 C      1.456392  -2.818765   2.107062
 H      1.729635  -3.862230   2.309547
 C      1.621275  -1.725656   3.027320
 H      2.035091  -1.792061   4.042722
 C      1.183813  -0.525093   2.370688
 H      1.185066   0.481506   2.808297
 C      3.870817  -0.969181  -0.324197
 H      3.502492  -0.807446  -1.345169
 C      4.236085  -2.238919   0.245403
 H      4.200800  -3.210236  -0.266172
 C      4.633275  -2.017105   1.612777
```

```
 H      4.949710  -2.789334   2.326866
 C      4.515785  -0.606990   1.886750
 H      4.727418  -0.117081   2.846760
 C      4.041499   0.040561   0.691134
 H      3.826863   1.110885   0.575374
 C     -1.547241   0.907940   0.856936
 C     -2.259248   2.017109   0.347708
 H     -1.932846   2.494060  -0.591243
 C     -3.377693   2.546345   1.013340
 H     -3.895298   3.416229   0.585139
 C     -3.820327   1.953394   2.219039
 C     -3.125092   0.832812   2.731654
 H     -3.477631   0.378512   3.670609
 C     -2.008576   0.320503   2.058220
 H     -1.482510  -0.546709   2.486300
 C      0.943568   1.743129  -0.274774
 C      1.702763   1.996129  -1.434854
 H      1.644101   1.310578  -2.291708
 C      2.552431   3.113499  -1.533675
 H      3.122641   3.271540  -2.459773
 C      2.654524   4.015534  -0.451064
 C      1.889564   3.778938   0.718284
 H      1.966608   4.493692   1.552208
 C      1.047864   2.665659   0.797880
 H      0.448485   2.520541   1.710247
 C      0.534814  -3.138833  -0.375685
 H      1.307457  -2.950802  -1.150187
 C     -1.885328  -2.868637  -0.097768
 H     -2.195771  -3.936200   0.031885
 H     -1.695940  -2.500047   0.950903
 C     -3.060302  -2.084007  -0.614406
 C     -3.769697  -0.263747  -1.900072
 C     -5.110365  -0.487647  -1.571391
 C     -5.430856  -1.568379  -0.730765
 C     -4.389773  -2.374150  -0.252650
 C      0.569482  -4.654662  -0.080963
 H      1.591304  -4.984773   0.198090
 H     -0.111954  -4.939418   0.747636
 H      0.264603  -5.214891  -0.988199
 C     -1.035589   0.535470  -3.021012
 C      0.872271  -1.019162  -2.645489
 H     -1.051027  -2.483581  -2.503712
 H     -1.253777  -1.972476  -3.249823
 H     -3.485847   0.564217  -2.565613
 H     -5.887181   0.176125  -1.978452
 H     -4.592367  -3.233803   0.403993
 H     -6.475891  -1.780451  -0.456917
 O     -4.888591   2.383338   2.945573
 C     -5.625786   3.511460   2.477644
 H     -6.432377   3.681682   3.214779
 H     -4.989798   4.422135   2.418280
 H     -6.079766   3.320935   1.480189
 O      3.437920   5.126963  -0.440162
 C      4.229694   5.418410  -1.591467
 H      4.778601   6.350332  -1.361490
 H      4.961094   4.607369  -1.801536
 H      3.598604   5.580638  -2.492718
---------------------------------------
Mn22/i
Frequencies, energies and thermodynamic properties:
 Lowest Vibrational Mode (1/cm) =                         21.8616
 2nd Lowest Vibrational Mode (1/cm) =                     23.6364
 E(RB-P86) (a.u.) =                                     -4329.97364669
 Thermal correction to Enthalpy (a.u.) =                  0.598505
 Thermal correction to Gibbs Free Energy (a.u.) =         0.484725
 Total Entropy (cal/Kmol) =                               239.471
 E(RPBE1PBE) (a.u.) =                                   -4329.28877985
Optimised cartesian coordinates (Angstrom):
 Fe2.790332  -1.222966   1.037999
 Mn-1.011230  -0.651354  -1.770910
 P     -0.141974   0.492587  -0.117769
 O     -1.718875   1.626720  -3.537417
 O      1.447213  -0.932234  -3.355628
 N     -0.878091  -2.319932  -0.875240
 N     -2.970338  -0.838113  -1.150385
 C      0.823705  -0.636281   0.974809
 C      0.930590  -2.081690   0.793138
 C      1.590583  -2.612365   1.962139
 H      1.844767  -3.666391   2.132847
 C      1.897446  -1.526669   2.855790
 H      2.418198  -1.611156   3.819372
 C      1.434744  -0.307926   2.250153
 H      1.521623   0.696777   2.683456
 C      3.763937  -0.876967  -0.745428
 H      3.278028  -0.725559  -1.718237
```

```
 C      4.161807  -2.144211  -0.191720
 H      4.039514  -3.125079  -0.670634
 C      4.726449  -1.904824   1.112348
 H      5.105738  -2.670493   1.802397
 C      4.680238  -0.486607   1.363199
 H      5.018355   0.017592   2.278524
 C      4.083045   0.148754   0.217130
 H      3.884894   1.222322   0.101732
 C     -1.447538   1.161498   1.021201
 C     -2.171959   2.311083   0.622061
 H     -1.901037   2.834363  -0.309715
 C     -3.230110   2.798612   1.401151
 H     -3.770664   3.702060   1.072545
 C     -3.619588   2.151775   2.599488
 C     -2.905167   0.997274   2.981651
 H     -3.185112   0.469802   3.908722
 C     -1.836073   0.506051   2.208333
 H     -1.294310  -0.390650   2.546607
 C      0.938965   1.981474  -0.363215
 C      1.569922   2.213691  -1.603233
 H      1.402411   1.525016  -2.443673
 C      2.423123   3.317085  -1.779032
 H      2.905489   3.473554  -2.758094
 C      2.671889   4.228606  -0.729427
 C      2.030732   3.993714   0.509228
 H      2.201190   4.688344   1.348581
 C      1.175840   2.895483   0.690404
 H      0.680568   2.756916   1.664392
 C      0.416853  -2.863231  -0.427058
 H      1.124185  -2.670287  -1.258110
 C     -1.999584  -2.751730  -0.062600
 H     -2.239630  -3.834342  -0.200178
 H     -1.789931  -2.645833   1.036046
 C     -3.215030  -1.937777  -0.381194
 C     -4.020970  -0.028786  -1.464516
 H     -3.789939   0.850841  -2.082229
 C     -5.327505  -0.287779  -1.046821
 H     -6.135131   0.400244  -1.337388
 C     -5.582641  -1.430585  -0.263117
 C     -4.507589  -2.263180   0.070016
 H     -4.654177  -3.167308   0.680518
 C      0.420040  -4.384513  -0.186712
 H      1.457519  -4.747705  -0.041388
 H     -0.165772  -4.680541   0.707477
 H      0.000881  -4.908692  -1.069869
 C     -1.428912   0.721041  -2.829207
 C      0.491731  -0.806001  -2.661990
 H     -6.602360  -1.664493   0.079116
 C      3.562756   5.432952  -0.928829
 H      4.276670   5.280527  -1.762711
 H      2.959337   6.335039  -1.173144
 H      4.139264   5.670485  -0.011672
 C     -4.760919   2.689433   3.431980
 H     -4.541649   3.714591   3.800706
 H     -5.695895   2.760383   2.836226
 H     -4.963860   2.047849   4.312103
 ---------------------------------------
Mn22/ii
Frequencies, energies and thermodynamic properties:
 Lowest Vibrational Mode (1/cm) =                       17.5179
 2nd Lowest Vibrational Mode (1/cm) =                   23.0117
 E(RB-P86) (a.u.) =                                    -4484.90856385
 Thermal correction to Enthalpy (a.u.) =                0.683227
 Thermal correction to Gibbs Free Energy (a.u.) =       0.554327
 Total Entropy (cal/Kmol) =                             271.293
 E(RPBE1PBE) (a.u.) =                                  -4484.23254388
Optimised cartesian coordinates (Angstrom):
 Fe-2.587967  -1.604006  -1.378193
 Mn1.224912  -0.392221   1.208620
 P     -0.351195   0.723333   0.169318
 O      1.329191   1.293776   3.644835
 O     -0.561396  -2.183808   2.703015
 N      1.556781  -1.594715  -0.260775
 N      2.912899   0.504170   0.429250
 C     -1.022065  -0.289188  -1.214806
 C     -0.536535  -1.613888  -1.594414
 C     -1.183707  -1.966408  -2.835606
 H     -1.052688  -2.909564  -3.381346
 C     -2.059647  -0.893459  -3.225251
 H     -2.704640  -0.877120  -4.114261
 C     -1.970355   0.138098  -2.228440
 H     -2.519448   1.088410  -2.238229
 C     -3.229303  -2.267237   0.464585
 H     -2.652191  -2.218089   1.396939
 C     -3.208775  -3.351939  -0.480643
```

```
 H     -2.619566  -4.274610  -0.391783
 C     -4.077077  -3.005588  -1.577094
 H     -4.262461  -3.615389  -2.471550
 C     -4.637835  -1.705962  -1.306710
 H     -5.325429  -1.152050  -1.959951
 C     -4.112171  -1.247780  -0.046672
 H     -4.326683  -0.283809   0.432790
 C      0.327968   2.223076  -0.688391
 C      0.638659   3.364199   0.090780
 H      0.407278   3.372914   1.168616
 C      1.233224   4.490785  -0.493375
 H      1.454768   5.370299   0.133997
 C      1.555929   4.519878  -1.872214
 C      1.257970   3.373892  -2.638488
 H      1.496698   3.365512  -3.714963
 C      0.654003   2.241040  -2.060542
 H      0.423486   1.371664  -2.695286
 C     -1.834598   1.428030   1.033356
 C     -2.226941   0.953783   2.301988
 H     -1.637494   0.176338   2.808235
 C     -3.376547   1.461084   2.933463
 H     -3.661565   1.071614   3.924709
 C     -4.169542   2.458583   2.326162
 C     -3.765359   2.935907   1.056966
 H     -4.359278   3.719237   0.557249
 C     -2.617813   2.436684   0.423087
 H     -2.324426   2.847664  -0.555717
 C      0.481258  -2.451304  -0.808899
 H     -0.044246  -2.860937   0.076974
 C      2.504742  -1.150213  -1.273577
 H      3.153000  -1.975909  -1.653998
 H      1.979988  -0.755632  -2.183905
 C      3.367633  -0.053780  -0.729186
 C      3.627269   1.526778   0.976072
 C      4.804498   2.015667   0.406678
 C      5.280932   1.433682  -0.784183
 C      4.550082   0.384570  -1.354110
 C      0.998861  -3.659382  -1.612483
 H      0.172936  -4.370350  -1.816782
 H      1.441418  -3.369596  -2.587567
 H      1.767687  -4.186851  -1.012244
 C      1.282455   0.622413   2.670215
 C      0.108954  -1.451011   2.055097
 H      2.498399  -2.828709   0.797778
 O      2.945820  -3.597007   1.258645
 C      4.347639  -3.363088   1.265052
 H      4.592099  -2.358756   1.694091
 H      4.770812  -3.371269   0.227602
 H      4.882431  -0.103224  -2.283047
 H      5.340983   2.841456   0.896628
 H      3.231197   1.954286   1.908240
 C      5.034142  -4.443661   2.093405
 H      6.133423  -4.293215   2.111384
 H      4.663303  -4.429544   3.139910
 H      4.828517  -5.450654   1.672769
 H      6.208028   1.793622  -1.256015
 C      2.199163   5.740346  -2.489461
 H      1.567457   6.643308  -2.348462
 H      3.178887   5.961756  -2.014278
 H      2.369560   5.608329  -3.576121
 C     -5.392089   3.022104   3.012782
 H     -5.706342   2.394671   3.870081
 H     -5.192847   4.044638   3.401790
 H     -6.247578   3.109095   2.311327
--------------------------------------
Mn22/iii
Frequencies, energies and thermodynamic properties:
 Lowest Vibrational Mode (1/cm) =                        17.7905
 2nd Lowest Vibrational Mode (1/cm) =                    25.2278
 E(RB-P86) (a.u.) =                                     -4486.08107206
 Thermal correction to Enthalpy (a.u.) =                 0.699211
 Thermal correction to Gibbs Free Energy (a.u.) =        0.570999
 Total Entropy (cal/Kmol) =                              269.846
 E(RPBE1PBE) (a.u.) =                                   -4485.40185773
Optimised cartesian coordinates (Angstrom):
 Fe-2.568977  -1.675203  -1.331266
 Mn1.246199  -0.368563   1.417486
 P     -0.321526   0.681215   0.144336
 O      0.918820   1.650559   3.557063
 O     -0.633173  -2.157070   2.809356
 N      1.610706  -1.690349  -0.159217
 N      2.844602   0.579335   0.505265
 C     -0.989450  -0.369922  -1.198163
 C     -0.512638  -1.713531  -1.510133
 C     -1.144127  -2.106442  -2.748284
```

```
 H     -1.015771  -3.071542  -3.254613
 C     -2.005043  -1.043564  -3.194455
 H     -2.637293  -1.058401  -4.092584
 C     -1.922406   0.026347  -2.238787
 H     -2.462523   0.980232  -2.293164
 C     -3.259403  -2.262160   0.520593
 H     -2.708171  -2.176863   1.465513
 C     -3.215601  -3.384491  -0.378283
 H     -2.629745  -4.302657  -0.236769
 C     -4.054094  -3.082206  -1.510414
 H     -4.216409  -3.727410  -2.384293
 C     -4.619931  -1.772305  -1.307786
 H     -5.289544  -1.245362  -2.000819
 C     -4.126894  -1.263467  -0.054148
 H     -4.353729  -0.281579   0.381275
 C      0.347535   2.167220  -0.750094
 C      0.673918   3.315626   0.011782
 H      0.483198   3.328710   1.097357
 C      1.227896   4.448818  -0.599426
 H      1.463724   5.332389   0.016822
 C      1.489979   4.479293  -1.990503
 C      1.173464   3.327739  -2.740793
 H      1.363838   3.319712  -3.826817
 C      0.611415   2.187979  -2.134716
 H      0.366122   1.314707  -2.758555
 C     -1.826708   1.417057   0.954637
 C     -2.282090   0.962499   2.209618
 H     -1.728143   0.185168   2.753624
 C     -3.453528   1.487560   2.783565
 H     -3.785359   1.111332   3.765317
 C     -4.209934   2.484534   2.130675
 C     -3.745165   2.941318   0.875181
 H     -4.309201   3.722399   0.338706
 C     -2.574553   2.424675   0.299753
 H     -2.236255   2.821019  -0.670350
 C      0.501577  -2.513863  -0.687980
 H     -0.029044  -2.901685   0.206162
 C      2.380065  -1.050654  -1.222926
 H      3.005337  -1.771375  -1.804433
 H      1.724983  -0.558434  -1.993924
 C      3.263843   0.028268  -0.668101
 C      3.563335   1.600226   1.039678
 C      4.722499   2.105036   0.442527
 C      5.168382   1.532152  -0.761884
 C      4.427580   0.481891  -1.318540
 C      1.005617  -3.751232  -1.463771
 H      0.170055  -4.446712  -1.684829
 H      1.480240  -3.481131  -2.430039
 H      1.748788  -4.291521  -0.843545
 C      1.065039   0.840591   2.710200
 C      0.075169  -1.424630   2.211177
 H      2.502983  -0.860328   2.499982
 H      2.451597  -1.534332   2.017397
 H      2.570003  -2.751014   0.594194
 O      3.125073  -3.409310   1.191980
 C      4.499667  -3.293156   0.874510
 H      4.694622  -3.535401  -0.202727
 H      4.872150  -2.245866   1.024669
 H      4.739573   0.002067  -2.258432
 H      5.265616   2.931346   0.923989
 H      3.190709   2.017995   1.986304
 C      5.316026  -4.240488   1.750704
 H      4.988184  -5.290892   1.599825
 H      6.398360  -4.176763   1.511777
 H      5.183354  -3.995390   2.825837
 H      6.081430   1.898169  -1.256246
 C      2.089579   5.706597  -2.637501
 H      2.210084   5.575743  -3.730923
 H      1.455141   6.602407  -2.466813
 H      3.088080   5.939077  -2.209053
 C     -5.454416   3.068672   2.757990
 H     -5.843687   2.425377   3.571777
 H     -5.244961   4.069210   3.196180
 H     -6.259567   3.210512   2.008000
---------------------------------------
Mn22/iv
Frequencies, energies and thermodynamic properties:
 Lowest Vibrational Mode (1/cm) =                          17.5595
 2nd Lowest Vibrational Mode (1/cm) =                      21.8058
 E(RB-P86) (a.u.) =                                     -4486.10930693
 Thermal correction to Enthalpy (a.u.) =                   0.703873
 Thermal correction to Gibbs Free Energy (a.u.) =          0.575273
 Total Entropy (cal/Kmol) =                                270.661
 E(RPBE1PBE) (a.u.) =                                   -4485.42832329
Optimised cartesian coordinates (Angstrom):
```

Fe	-2.685562	-1.509742	-1.372996
Mn	1.312342	-0.490136	1.220076
P	-0.315730	0.697824	0.152040
O	1.322964	1.228038	3.605205
O	-0.380597	-2.426947	2.648163
N	1.551306	-1.717390	-0.533201
N	2.888960	0.455338	0.300577
C	-1.093855	-0.221679	-1.242116
C	-0.660191	-1.539237	-1.708204
C	-1.382820	-1.820873	-2.928394
H	-1.304025	-2.737475	-3.526183
C	-2.258190	-0.717177	-3.214650
H	-2.954477	-0.651041	-4.061447
C	-2.089459	0.264303	-2.179616
H	-2.620099	1.222480	-2.110141
C	-3.217227	-2.256567	0.470573
H	-2.578192	-2.262161	1.363322
C	-3.276514	-3.291908	-0.527636
H	-2.698670	-4.225996	-0.524474
C	-4.209292	-2.878666	-1.544898
H	-4.462435	-3.439062	-2.454903
C	-4.730181	-1.588073	-1.172491
H	-5.449790	-0.992245	-1.749900
C	-4.116013	-1.201816	0.071148
H	-4.282765	-0.261124	0.611667
C	0.294467	2.254074	-0.669089
C	0.728834	3.311463	0.168343
H	0.627050	3.219888	1.262436
C	1.279602	4.480882	-0.373933
H	1.598626	5.291095	0.303075
C	1.436742	4.639776	-1.772215
C	1.018171	3.577804	-2.600247
H	1.126509	3.669207	-3.693944
C	0.456463	2.403594	-2.061998
H	0.129155	1.605630	-2.746704
C	-1.762377	1.367558	1.115451
C	-2.114962	0.801258	2.358248
H	-1.520871	-0.026206	2.771914
C	-3.227426	1.274939	3.075938
H	-3.481141	0.810900	4.043536
C	-4.023160	2.332825	2.584438
C	-3.661248	2.901301	1.340839
H	-4.259818	3.731185	0.929277
C	-2.550055	2.433797	0.621640
H	-2.289928	2.915144	-0.334415
C	0.353496	-2.464736	-1.047201
H	-0.108152	-2.890749	-0.134024
C	2.286687	-0.967815	-1.573754
H	2.822938	-1.631411	-2.286490
H	1.547707	-0.389983	-2.173256
C	3.243954	0.005301	-0.938107
C	3.676136	1.398343	0.882852
C	4.832444	1.906502	0.280108
C	5.212144	1.424700	-0.983723
C	4.399712	0.457977	-1.595911
C	0.760870	-3.642061	-1.949591
H	-0.115223	-4.285080	-2.166397
H	1.183505	-3.308021	-2.919171
H	1.514713	-4.269495	-1.432108
C	1.310630	0.540233	2.638511
C	0.253728	-1.627985	2.041406
H	2.492941	-1.341956	1.892156
H	2.867482	-2.720675	1.571497
H	2.193234	-2.443785	-0.149391
O	3.147846	-3.552848	1.075654
C	4.578271	-3.589450	1.062009
H	4.867886	-4.331368	0.286169
H	5.000297	-2.607381	0.738756
H	4.650951	0.049381	-2.586452
H	5.426916	2.666650	0.808296
H	3.363166	1.743882	1.878590
C	5.166407	-3.990998	2.414391
H	4.767268	-4.974283	2.739839
H	6.273054	-4.066559	2.357407
H	4.917118	-3.242762	3.196544
H	6.121725	1.792798	-1.482255
C	-5.199051	2.864788	3.371093
H	-5.570706	2.121916	4.104709
H	-4.914719	3.776942	3.940840
H	-6.039166	3.151086	2.705812
C	2.035517	5.904244	-2.345071
H	2.071068	5.875417	-3.452064
H	1.450724	6.799887	-2.044686
H	3.071054	6.063354	-1.974736

```
Mn22/v
Frequencies, energies and thermodynamic properties:
 Lowest Vibrational Mode (1/cm) =                         21.5599
 2nd Lowest Vibrational Mode (1/cm) =                     27.1238
 E(RB-P86) (a.u.) =                                     -4331.17082007
 Thermal correction to Enthalpy (a.u.) =                  0.619429
 Thermal correction to Gibbs Free Energy (a.u.) =         0.505249
 Total Entropy (cal/Kmol) =                               240.311
 E(RPBE1PBE) (a.u.) =                                   -4330.48575176
Optimised cartesian coordinates (Angstrom):
 Fe-2.802081  -1.128219  -1.081287
 Mn1.041229  -0.684534   1.879076
 P      0.184247   0.462273   0.106189
 O      1.681666   1.687748   3.490683
 O     -1.400530  -1.091247   3.467336
 N      0.827311  -2.529504   0.793862
 N      2.932015  -0.896838   1.114024
 C     -0.819543  -0.598957  -1.020268
 C     -0.978988  -2.046824  -0.874560
 C     -1.659248  -2.525145  -2.057778
 H     -1.949758  -3.563917  -2.258335
 C     -1.934386  -1.407135  -2.917928
 H     -2.465806  -1.449933  -3.878129
 C     -1.426450  -0.223059  -2.283645
 H     -1.484376   0.796110  -2.686735
 C     -3.746051  -0.786310   0.717152
 H     -3.245781  -0.666501   1.687290
 C     -4.186701  -2.031936   0.145735
 H     -4.089024  -3.024474   0.605959
 C     -4.759068  -1.752737  -1.146673
 H     -5.167549  -2.494537  -1.846029
 C     -4.675465  -0.332339  -1.372348
 H     -5.008231   0.197929  -2.274728
 C     -4.047404   0.265119  -0.222836
 H     -3.815553   1.329947  -0.091496
 C      1.464402   1.176944  -1.043160
 C      2.252151   2.252379  -0.562204
 H      2.037771   2.686560   0.428518
 C      3.298417   2.780726  -1.331240
 H      3.886502   3.625329  -0.934370
 C      3.616618   2.248779  -2.604456
 C      2.841802   1.166694  -3.070714
 H      3.063774   0.727957  -4.057966
 C      1.783616   0.637308  -2.306567
 H      1.195035  -0.198420  -2.716511
 C     -0.892800   1.955486   0.388950
 C     -1.538698   2.136422   1.629867
 H     -1.385275   1.404090   2.435783
 C     -2.385936   3.237042   1.847241
 H     -2.880724   3.351099   2.826169
 C     -2.613377   4.199489   0.838786
 C     -1.957330   4.017113  -0.400724
 H     -2.111232   4.752067  -1.208545
 C     -1.108746   2.920543  -0.621944
 H     -0.601705   2.822791  -1.594987
 C     -0.537745  -2.915620   0.297069
 H     -1.202980  -2.705557   1.158197
 C      1.899144  -2.646891  -0.218604
 H      2.129737  -3.700924  -0.484587
 H      1.537777  -2.154064  -1.148902
 C      3.140420  -1.933044   0.248653
 C      4.014742  -0.166840   1.494991
 H      3.818702   0.664012   2.187825
 C      5.316152  -0.444273   1.061169
 H      6.147038   0.185371   1.412733
 C      5.532551  -1.527740   0.193558
 C      4.419615  -2.279814  -0.214473
 H      4.532064  -3.136629  -0.896168
 C     -0.634077  -4.418989  -0.011145
 H     -1.687621  -4.707064  -0.197579
 H     -0.041697  -4.709204  -0.902654
 H     -0.278734  -5.013948   0.855418
 C      1.414026   0.739220   2.827753
 C     -0.449957  -0.902088   2.780760
 H      1.046923  -3.165516   1.572658
 H      1.612787  -1.615962   3.030735
 H      6.543991  -1.782252  -0.157823
 C      4.748879   2.825626  -3.423665
 H      4.866629   2.295732  -4.389574
 H      4.580951   3.901916  -3.643012
 H      5.715051   2.760584  -2.878725
 C     -3.496137   5.401473   1.084998
 H     -4.255874   5.198120   1.866143
 H     -2.894517   6.270425   1.432442
 H     -4.019559   5.720456   0.160874
```

```
--------------------------------------
Mn22/vi_R
Frequencies, energies and thermodynamic properties:
 Lowest Vibrational Mode (1/cm) =                        14.1923
 2nd Lowest Vibrational Mode (1/cm) =                    18.7747
 E(RB-P86) (a.u.) =                                    -4753.88368387
 Thermal correction to Enthalpy (a.u.) =                 0.771907
 Thermal correction to Gibbs Free Energy (a.u.) =        0.635227
 Total Entropy (cal/Kmol) =                              287.668
 E(RPBE1PBE) (a.u.) =                                  -4753.19395173
Optimised cartesian coordinates (Angstrom):
 Fe-2.690996  -2.132211  -1.544215
 Mn1.011492   0.260114   0.318241
 P     -1.178266   0.501124   0.350111
 O      1.421980   0.927927   3.175414
 O      1.021367  -2.574758   1.102361
 N      1.079095  -0.055010  -1.616766
 N      1.533694   2.153122  -0.307064
 C     -1.931801  -0.269983  -1.139319
 C     -1.182340  -0.901775  -2.222657
 C     -2.123297  -1.188592  -3.279745
 H     -1.888437  -1.682324  -4.231229
 C     -3.432512  -0.756687  -2.868708
 H     -4.358711  -0.861715  -3.449780
 C     -3.323026  -0.197849  -1.549333
 H     -4.148499   0.219707  -0.958828
 C     -2.067593  -3.534494  -0.168637
 H     -1.207121  -3.428375   0.504235
 C     -2.031812  -4.083878  -1.498002
 H     -1.143122  -4.475370  -2.011171
 C     -3.361151  -4.007715  -2.048523
 H     -3.662334  -4.326282  -3.055516
 C     -4.220179  -3.413493  -1.055856
 H     -5.291062  -3.200422  -1.174568
 C     -3.421021  -3.118256   0.105140
 H     -3.772551  -2.641481   1.029476
 C     -1.677162   2.284147   0.213014
 C     -1.526672   3.113747   1.350963
 H     -1.183129   2.681057   2.304946
 C     -1.815613   4.483279   1.282561
 H     -1.699325   5.104948   2.185958
 C     -2.251335   5.083100   0.075818
 C     -2.383713   4.253516  -1.057090
 H     -2.720370   4.690606  -2.011780
 C     -2.102298   2.875402  -0.994688
 H     -2.232091   2.258912  -1.897454
 C     -2.224047  -0.061352   1.777438
 C     -1.748771  -1.019994   2.696008
 H     -0.733980  -1.429035   2.594493
 C     -2.564243  -1.472154   3.748457
 H     -2.167127  -2.223165   4.451129
 C     -3.876954  -0.983336   3.923991
 C     -4.345530  -0.017457   3.002687
 H     -5.364825   0.388514   3.113136
 C     -3.535488   0.441145   1.952959
 H     -3.929960   1.208896   1.268953
 C      0.326093  -1.177133  -2.226177
 H      0.509708  -2.043908  -1.559856
 C      1.081485   1.145038  -2.451570
 H      1.714955   1.034966  -3.363702
 H      0.054299   1.380028  -2.835230
 C      1.556968   2.324571  -1.659602
 C      1.916852   3.194207   0.482437
 H      1.882852   3.021825   1.567595
 C      2.341130   4.419934  -0.035208
 H      2.642435   5.223571   0.652506
 C      2.378005   4.594759  -1.431843
 C      1.982290   3.529782  -2.249488
 H      1.995049   3.618430  -3.346269
 C      0.836424  -1.578179  -3.624040
 H      0.362249  -2.526230  -3.948990
 H      0.616579  -0.812462  -4.395963
 H      1.932166  -1.744532  -3.594361
 C      1.259932   0.662816   2.033051
 C      0.958416  -1.440094   0.766410
 H      2.667459  -0.480698  -1.698211
 H      3.183139  -0.128095   0.261396
 C      4.104195  -0.355940  -0.390312
 C      4.869682  -1.465289   0.318567
 C      5.075200   0.853316  -0.268023
 C      5.888430  -0.920649   1.131479
 C      4.674004  -2.852335   0.238473
 C      5.889648   0.595336   1.023264
 H      5.743251   0.815801  -1.156534
 C      6.718481  -1.768034   1.884627
```

```
 C      5.508565  -3.701985   0.991659
 H      3.884862  -3.263513  -0.411219
 H      6.911068   1.029958   0.999744
 C      6.522326  -3.162638   1.810166
 H      7.520872  -1.353495   2.517489
 H      5.373942  -4.794568   0.937348
 H      7.172656  -3.837568   2.390067
 O      3.683963  -0.654274  -1.686279
 H      4.552152   1.830640  -0.279570
 H      5.377699   1.041085   1.907251
 H      2.712506   5.546400  -1.872485
 C     -2.563210   6.560708   0.016572
 H     -3.395076   6.823342   0.704953
 H     -1.688599   7.169706   0.329875
 H     -2.853413   6.877627  -1.004478
 C     -4.744258  -1.447292   5.071110
 H     -4.372917  -2.395309   5.508164
 H     -4.761303  -0.690184   5.885694
 H     -5.795981  -1.596762   4.751351
---------------------------------------
Mn22/vi_S
Frequencies, energies and thermodynamic properties:
 Lowest Vibrational Mode (1/cm) =                          13.8635
 2nd Lowest Vibrational Mode (1/cm) =                      17.8563
 E(RB-P86) (a.u.) =                                     -4753.88396932
 Thermal correction to Enthalpy (a.u.) =                   0.771913
 Thermal correction to Gibbs Free Energy (a.u.) =          0.634337
 Total Entropy (cal/Kmol) =                                289.552
 E(RPBE1PBE) (a.u.) =                                   -4753.19421250
Optimised cartesian coordinates (Angstrom):
 Fe-3.306033  -1.663829  -1.340788
 Mn0.891291  -0.238707   0.456949
 P     -1.107910   0.666043   0.260709
 O      1.382706   0.731873   3.212854
 O     -0.023359  -2.767617   1.645137
 N      0.923152  -0.865523  -1.401974
 N      2.028052   1.263177  -0.383487
 C     -2.016780  -0.077894  -1.153778
 C     -1.466704  -1.077524  -2.066016
 C     -2.414696  -1.237815  -3.143197
 H     -2.313993  -1.929938  -3.988847
 C     -3.536801  -0.368185  -2.910014
 H     -4.429743  -0.282822  -3.543967
 C     -3.302037   0.342342  -1.683141
 H     -3.974998   1.081868  -1.230590
 C     -3.198620  -2.947218   0.268767
 H     -2.370564  -3.003493   0.986766
 C     -3.296890  -3.688844  -0.960292
 H     -2.561661  -4.411674  -1.338877
 C     -4.516997  -3.299451  -1.620432
 H     -4.872160  -3.669246  -2.591705
 C     -5.175017  -2.317913  -0.795830
 H     -6.119844  -1.808934  -1.029368
 C     -4.359463  -2.097508   0.370343
 H     -4.571064  -1.392970   1.185196
 C     -1.009986   2.465572  -0.185589
 C     -0.627890   3.385785   0.820993
 H     -0.458855   3.033565   1.851867
 C     -0.467034   4.745904   0.524926
 H     -0.178429   5.442630   1.329535
 C     -0.666218   5.240932  -0.787082
 C     -1.031681   4.316226  -1.787296
 H     -1.193829   4.670489  -2.818812
 C     -1.201819   2.949136  -1.496410
 H     -1.502292   2.261605  -2.301947
 C     -2.325694   0.703459   1.661734
 C     -2.211421  -0.185255   2.750607
 H     -1.376160  -0.897579   2.799069
 C     -3.163403  -0.179369   3.785393
 H     -3.049700  -0.886113   4.623873
 C     -4.257442   0.712677   3.773243
 C     -4.362695   1.606165   2.681434
 H     -5.202626   2.319619   2.642874
 C     -3.414251   1.608050   1.647643
 H     -3.520793   2.332655   0.825100
 C     -0.120006  -1.791656  -1.902815
 H     -0.238739  -2.551226  -1.104043
 C      1.319605   0.133554  -2.393293
 H      1.896486  -0.304013  -3.242126
 H      0.425984   0.619338  -2.865906
 C      2.135815   1.207643  -1.741539
 C      2.714484   2.233304   0.280133
 H      2.604574   2.247584   1.373836
 C      3.524703   3.166286  -0.370618
 H      4.057674   3.926167   0.219337
```

```
 C      3.644161   3.105983  -1.771989
 C      2.941838   2.109875  -2.460850
 H      3.005602   2.021473  -3.555859
 C      0.290712  -2.553218  -3.178240
 H     -0.441497  -3.355668  -3.400039
 H      0.347714  -1.896449  -4.070446
 H      1.279311  -3.032694  -3.029133
 C      1.190351   0.344869   2.110809
 C      0.282865  -1.738508   1.141620
 H      2.317123  -1.739751  -1.311206
 H      2.851926  -1.257302   0.617991
 C      3.601981  -1.979759   0.128132
 C      3.631937  -3.270005   0.997348
 C      4.624688  -2.964032   2.145718
 O      3.252504  -2.161821  -1.209802
 H      4.278274   3.823221  -2.315282
 C      4.986852  -1.393581   0.360443
 C      5.569688  -1.950830   1.520569
 C      5.672883  -0.438827  -0.405514
 C      6.850423  -1.543782   1.930689
 C      6.958809  -0.033563   0.004648
 H      5.212841  -0.027701  -1.318741
 C      7.541581  -0.580891   1.166172
 H      7.317677  -1.977054   2.830750
 H      7.516287   0.709996  -0.587888
 H      8.550324  -0.260644   1.473776
 H      5.146182  -3.863465   2.535346
 H      4.094945  -2.506914   3.013304
 H      4.035071  -4.079655   0.349904
 H      2.625668  -3.579105   1.342734
 C     -0.492169   6.710779  -1.092531
 H     -1.202604   7.330216  -0.504054
 H      0.528265   7.059258  -0.825349
 H     -0.658304   6.928571  -2.165898
 C     -5.266378   0.738781   4.897862
 H     -5.218477  -0.181736   5.512619
 H     -5.082192   1.601439   5.575230
 H     -6.301099   0.849818   4.513239
---------------------------------------
Mn22/viii
Frequencies, energies and thermodynamic properties:
 Lowest Vibrational Mode (1/cm) =                          22.0369
 2nd Lowest Vibrational Mode (1/cm) =                      26.5712
 E(RB-P86) (a.u.) =                                      -4331.13716467
 Thermal correction to Enthalpy (a.u.) =                   0.614507
 Thermal correction to Gibbs Free Energy (a.u.) =          0.499727
 Total Entropy (cal/Kmol) =                                241.575
 E(RPBE1PBE) (a.u.) =                                    -4330.45281560
Optimised cartesian coordinates (Angstrom):
 Fe2.823360  -1.116334   1.072569
 Mn-1.014706  -0.709929  -1.909347
 P     -0.182499   0.406366  -0.127538
 O     -1.601268   1.813154  -3.350370
 O      1.543471  -0.937200  -3.346744
 N     -0.801425  -2.461779  -0.863657
 N     -2.919302  -0.888870  -1.111761
 C      0.824498  -0.647537   0.984876
 C      1.014717  -2.083578   0.811849
 C      1.691724  -2.563766   1.993260
 H      2.003729  -3.600254   2.175485
 C      1.930701  -1.455890   2.880845
 H      2.446812  -1.502492   3.849545
 C      1.406963  -0.269435   2.260836
 H      1.433976   0.742242   2.685578
 C      3.802007  -0.698511  -0.694357
 H      3.323315  -0.549755  -1.670669
 C      4.244760  -1.958387  -0.159544
 H      4.167913  -2.934125  -0.657920
 C      4.783637  -1.720314   1.155796
 H      5.186448  -2.482530   1.836339
 C      4.676840  -0.309619   1.431540
 H      4.984770   0.190833   2.359543
 C      4.066981   0.322034   0.290023
 H      3.826737   1.388622   0.190713
 C     -1.508839   1.074817   0.991125
 C     -2.275670   2.176145   0.538942
 H     -2.045168   2.644987  -0.431704
 C     -3.323384   2.688774   1.316244
 H     -3.898432   3.552984   0.943792
 C     -3.656898   2.117594   2.568186
 C     -2.897798   1.012275   3.005659
 H     -3.132339   0.544521   3.976301
 C     -1.840546   0.495527   2.232586
 H     -1.264552  -0.361721   2.613852
 C      0.849307   1.935477  -0.382531
```

```
 C      1.510416   2.176738  -1.604611
 H      1.397159   1.476374  -2.443439
 C      2.329624   3.307763  -1.769632
 H      2.834686   3.468671  -2.736412
 C      2.516292   4.239507  -0.725946
 C      1.846146   3.996177   0.495805
 H      1.966431   4.705584   1.331516
 C      1.024110   2.871708   0.664545
 H      0.504582   2.729198   1.625031
 C      0.515522  -2.891476  -0.396019
 H      1.219198  -2.692930  -1.231742
 C     -1.850373  -2.689524   0.104363
 H     -2.117222  -3.771793   0.226513
 H     -1.587803  -2.357365   1.154179
 C     -3.096827  -1.937258  -0.259728
 C     -3.997307  -0.133294  -1.449197
 C     -5.283885  -0.389932  -0.967596
 C     -5.476807  -1.478624  -0.096503
 C     -4.368237  -2.257142   0.256310
 C      0.592435  -4.410365  -0.119793
 H      1.640472  -4.732883   0.051006
 H      0.002631  -4.709236   0.771897
 H      0.200493  -4.966417  -0.995910
 C     -1.377483   0.793982  -2.793282
 C      0.547726  -0.827175  -2.717244
 H     -1.538684  -2.081639  -2.893910
 H     -1.812233  -1.456764  -3.344234
 H     -3.810923   0.703318  -2.138324
 H     -6.119443   0.253816  -1.279318
 H     -4.470976  -3.119466   0.932557
 H     -6.477403  -1.715487   0.297047
 C      3.373549   5.470225  -0.910976
 H      4.056115   5.367399  -1.777770
 H      2.743210   6.369056  -1.089594
 H      3.982937   5.681275  -0.008263
 C     -4.787941   2.680753   3.397861
 H     -4.924706   2.116146   4.341169
 H     -4.603872   3.744765   3.659734
 H     -5.748390   2.653366   2.840056
---------------------------------------
Mn22/ix
Frequencies, energies and thermodynamic properties:
 Lowest Vibrational Mode (1/cm) =                          23.3939
 2nd Lowest Vibrational Mode (1/cm) =                      28.4113
 E(RB-P86) (a.u.) =                                      -4484.92491105
 Thermal correction to Enthalpy (a.u.) =                   0.683752
 Thermal correction to Gibbs Free Energy (a.u.) =          0.560539
 Total Entropy (cal/Kmol) =                                259.324
 E(RPBE1PBE) (a.u.) =                                    -4484.24444557
Optimised cartesian coordinates (Angstrom):
 Fe-2.989276  -0.850644  -1.338446
 Mn1.212812  -1.005784   1.117997
 P     -0.032614   0.582721   0.097331
 O      1.784310   0.725759   3.426200
 O     -1.033360  -2.204204   2.589475
 N      1.010030  -2.245000  -0.605716
 N      2.942179  -0.498306   0.076513
 C     -1.077285  -0.103865  -1.262000
 C     -1.074208  -1.495243  -1.714735
 C     -1.879355  -1.559306  -2.912853
 H     -2.095782  -2.464295  -3.494291
 C     -2.387353  -0.245850  -3.200955
 H     -3.051995   0.019404  -4.034106
 C     -1.904403   0.649716  -2.187129
 H     -2.119713   1.723982  -2.126631
 C     -3.694730  -1.351531   0.531858
 H     -3.076285  -1.516483   1.423489
 C     -4.065556  -2.354441  -0.431470
 H     -3.786254  -3.416226  -0.398642
 C     -4.847929  -1.717816  -1.460183
 H     -5.264053  -2.207191  -2.351019
 C     -4.963094  -0.320013  -1.130127
 H     -5.482583   0.442215  -1.726274
 C     -4.248366  -0.092385   0.099011
 H     -4.125853   0.872356   0.608270
 C      0.971430   1.884507  -0.778206
 C      1.661333   2.834467   0.014255
 H      1.535866   2.829200   1.109612
 C      2.499875   3.790424  -0.575304
 H      3.016381   4.523867   0.066366
 C      2.697910   3.830131  -1.976931
 C      2.020112   2.872361  -2.759201
 H      2.152548   2.875822  -3.853988
 C      1.170016   1.914222  -2.173620
 H      0.648191   1.194322  -2.823160
```

```
 C     -1.201350   1.660545   1.067730
 C     -1.651165   1.272693   2.346777
 H     -1.294656   0.336262   2.797252
 C     -2.564073   2.068528   3.062068
 H     -2.898768   1.738033   4.059303
 C     -3.057037   3.279888   2.531589
 C     -2.595927   3.669252   1.251914
 H     -2.955725   4.613060   0.809111
 C     -1.682522   2.880941   0.535929
 H     -1.332253   3.230468  -0.447915
 C     -0.341543  -2.665329  -1.066114
 H     -0.875603  -2.939616  -0.133794
 C      1.939420  -1.858094  -1.679416
 H      2.278821  -2.724369  -2.288590
 H      1.406189  -1.174302  -2.379062
 C      3.129731  -1.124307  -1.115781
 C      3.962967   0.230669   0.589418
 C      5.200370   0.363388  -0.050896
 C      5.402874  -0.293053  -1.276760
 C      4.350267  -1.048125  -1.811728
 C     -0.305292  -3.915730  -1.961514
 H     -1.331788  -4.296375  -2.134404
 H      0.151515  -3.715065  -2.952043
 H      0.271166  -4.723420  -1.466004
 C      1.562031   0.013902   2.503900
 C     -0.158027  -1.694528   1.973302
 H      1.464234  -2.971496   0.009496
 H      4.462785  -1.581774  -2.767656
 H      5.991971   0.967593   0.416196
 H      3.776278   0.717015   1.557858
 H      6.366150  -0.220947  -1.804719
 O      2.349050  -2.657452   1.501195
 C      2.364356  -3.367538   2.703159
 C      3.239765  -2.737016   3.798869
 H      2.763167  -4.399102   2.503323
 H      1.336031  -3.531622   3.128414
 H      3.266257  -3.375686   4.709461
 H      4.283517  -2.608776   3.438440
 H      2.857729  -1.737158   4.091767
 C      3.604825   4.866168  -2.600891
 H      3.662865   4.750333  -3.701184
 H      3.248535   5.896154  -2.384282
 H      4.636455   4.795945  -2.194472
 C     -4.019443   4.148919   3.307902
 H     -4.465102   3.603512   4.163159
 H     -3.504391   5.046225   3.715665
 H     -4.842940   4.519678   2.663022
---------------------------------------
Mn22/x
Frequencies, energies and thermodynamic properties:
 Lowest Vibrational Mode (1/cm) =                         11.7643
 2nd Lowest Vibrational Mode (1/cm) =                     19.9135
 E(RB-P86) (a.u.) =                                      -4639.86655799
 Thermal correction to Enthalpy (a.u.) =                  0.767929
 Thermal correction to Gibbs Free Energy (a.u.) =         0.630057
 Total Entropy (cal/Kmol) =                               290.176
 E(RPBE1PBE) (a.u.) =                                    -4639.19399965
Optimised cartesian coordinates (Angstrom):
 Fe-2.781944  -1.834699  -1.261332
 Mn1.236251  -0.203729   1.006697
 P     -0.626052   0.695222   0.073968
 O      0.991404   1.662472   3.266509
 O     -0.152288  -2.300242   2.531509
 N      1.494417  -1.424034  -0.750636
 N      2.534345   0.979750  -0.103197
 C     -1.370604  -0.344319  -1.250271
 C     -0.799165  -1.595295  -1.744399
 C     -1.563021  -1.986914  -2.906869
 H     -1.403681  -2.892466  -3.505488
 C     -2.597772  -1.014339  -3.130019
 H     -3.356524  -1.051916  -3.923157
 C     -2.489737  -0.007070  -2.111540
 H     -3.138956   0.871481  -2.007097
 C     -3.087477  -2.630997   0.614234
 H     -2.397865  -2.547166   1.464116
 C     -3.076033  -3.672896  -0.378378
 H     -2.381735  -4.523184  -0.414480
 C     -4.120118  -3.393293  -1.330848
 H     -4.356833  -3.988810  -2.222774
 C     -4.779779  -2.178820  -0.923944
 H     -5.607321  -1.686645  -1.452279
 C     -4.140499  -1.705538   0.276071
 H     -4.392625  -0.789985   0.826616
 C     -0.311776   2.311557  -0.797044
 C     -0.014850   3.447410  -0.004271
```

```
 H     -0.040552   3.371716   1.095189
 C      0.303342   4.676310  -0.598695
 H      0.520005   5.545952   0.044051
 C      0.352778   4.821474  -2.006380
 C      0.069377   3.683859  -2.790012
 H      0.097882   3.762892  -3.889539
 C     -0.258458   2.448185  -2.199306
 H     -0.490000   1.590637  -2.849878
 C     -2.075429   1.165603   1.145181
 C     -2.263513   0.580107   2.414233
 H     -1.535039  -0.144613   2.802970
 C     -3.385221   0.904095   3.198205
 H     -3.505715   0.428778   4.185715
 C     -4.356234   1.824379   2.748235
 C     -4.159618   2.413718   1.477308
 H     -4.896346   3.140349   1.095925
 C     -3.040055   2.097290   0.692988
 H     -2.913449   2.592895  -0.282251
 C      0.384503  -2.350554  -1.158179
 H      0.062103  -2.819729  -0.207153
 C      2.033694  -0.615844  -1.865243
 H      2.618615  -1.222630  -2.589951
 H      1.181385  -0.181704  -2.435333
 C      2.872329   0.517683  -1.336677
 C      3.208097   2.044754   0.393937
 C      4.237788   2.686442  -0.304516
 C      4.601610   2.201984  -1.571853
 C      3.907547   1.099476  -2.090622
 C      0.889019  -3.478352  -2.074969
 H      0.104568  -4.250353  -2.205286
 H      1.172815  -3.111537  -3.082628
 H      1.771079  -3.962549  -1.609674
 C      1.102807   0.903906   2.363426
 C      0.357038  -1.439741   1.897826
 H      3.383518  -2.278130   0.914681
 H      2.271914  -2.057333  -0.430503
 O      3.563364  -3.096097   0.264044
 C      4.864088  -2.953930  -0.288482
 H      4.870313  -3.428653  -1.298017
 H      5.117913  -1.875989  -0.446530
 H      4.158237   0.683467  -3.078070
 H      4.747547   3.548585   0.150094
 H      2.905787   2.390777   1.392627
 C      5.943911  -3.608470   0.578268
 H      5.717636  -4.684323   0.735537
 H      6.944562  -3.531305   0.100978
 H      6.003116  -3.123978   1.576196
 H      5.414530   2.673123  -2.145415
 O      3.020898  -1.064057   1.606269
 C      3.379854  -1.204781   2.961341
 C      4.231459  -0.044541   3.494217
 H      3.968924  -2.152335   3.080859
 H      2.484821  -1.327987   3.623610
 H      4.558205  -0.241179   4.539080
 H      5.139341   0.094356   2.869260
 H      3.662716   0.907980   3.490921
 C      0.697545   6.151961  -2.635435
 H      0.691560   6.095545  -3.741847
 H     -0.022410   6.940841  -2.329132
 H      1.703327   6.499729  -2.316143
 C     -5.549045   2.193474   3.599616
 H     -5.700610   1.472426   4.427080
 H     -5.415987   3.199654   4.054279
 H     -6.480385   2.235923   2.997905
--------------------------------------
Mn22/TS-i
Frequencies, energies and thermodynamic properties:
 Lowest Vibrational Mode (1/cm) =-743.1474
 2nd Lowest Vibrational Mode (1/cm) =                      22.2115
 E(RB-P86) (a.u.) =                                       -4486.07896508
 Thermal correction to Enthalpy (a.u.) =                   0.695524
 Thermal correction to Gibbs Free Energy (a.u.) =          0.569858
 Total Entropy (cal/Kmol) =                                264.485
 E(RPBE1PBE) (a.u.) =                                     -4485.39718041
Optimised cartesian coordinates (Angstrom):
 Fe-2.633570  -1.572588  -1.339147
 Mn1.249483  -0.440238   1.385514
 P     -0.289596   0.687495   0.145060
 O      1.051680   1.538105   3.570791
 O     -0.679970  -2.167861   2.786281
 N      1.548877  -1.764525  -0.221890
 N      2.876577   0.448917   0.467915
 C     -1.008681  -0.325034  -1.204660
 C     -0.585261  -1.681585  -1.541474
 C     -1.241979  -2.034834  -2.778508
```

```
 H     -1.153524  -2.996842  -3.298990
 C     -2.067226  -0.935029  -3.200579
 H     -2.708700  -0.914638  -4.091932
 C     -1.935580   0.117638  -2.231627
 H     -2.441210   1.091042  -2.267726
 C     -3.322988  -2.167557   0.510371
 H     -2.757361  -2.122176   1.449651
 C     -3.334828  -3.273206  -0.410132
 H     -2.783824  -4.216191  -0.294019
 C     -4.175584  -2.916835  -1.524590
 H     -4.374008  -3.538075  -2.408297
 C     -4.686939  -1.590223  -1.289723
 H     -5.343910  -1.024132  -1.963671
 C     -4.158176  -1.125219  -0.033783
 H     -4.340699  -0.143684   0.422766
 C      0.426095   2.154570  -0.744824
 C      0.812733   3.278620   0.025398
 H      0.635521   3.287797   1.113342
 C      1.410041   4.392742  -0.579988
 H      1.692198   5.257855   0.042961
 C      1.657997   4.427145  -1.973601
 C      1.281872   3.299425  -2.732397
 H      1.459903   3.294948  -3.820530
 C      0.675436   2.179387  -2.132182
 H      0.383208   1.326049  -2.763433
 C     -1.759716   1.469560   0.974870
 C     -2.218477   1.017879   2.229794
 H     -1.688485   0.213478   2.758175
 C     -3.363076   1.579864   2.822373
 H     -3.698677   1.204924   3.803340
 C     -4.088095   2.612238   2.188906
 C     -3.619780   3.065678   0.933593
 H     -4.159704   3.873494   0.412113
 C     -2.475678   2.511953   0.339379
 H     -2.132903   2.906351  -0.629987
 C      0.396191  -2.540978  -0.746871
 H     -0.133085  -2.911956   0.154567
 C      2.340388  -1.136090  -1.285357
 H      2.923024  -1.873711  -1.884923
 H      1.682587  -0.609681  -2.023337
 C      3.272145  -0.106469  -0.711819
 C      3.639855   1.432245   1.010841
 C      4.820110   1.891277   0.417440
 C      5.241893   1.310170  -0.791082
 C      4.455083   0.298830  -1.357734
 C      0.847148  -3.787881  -1.534994
 H     -0.016122  -4.449931  -1.750005
 H      1.322439  -3.530030  -2.503964
 H      1.573755  -4.361074  -0.924573
 C      1.141110   0.744773   2.700461
 C      0.050777  -1.458537   2.186479
 H      2.489692  -1.049879   2.391158
 H      2.455090  -1.786744   1.951549
 H      2.307854  -2.608743   0.443985
 O      2.906607  -3.285866   1.241739
 C      4.299799  -3.270260   1.044988
 H      4.565446  -3.375772  -0.040895
 H      4.755566  -2.292540   1.366983
 H      4.745973  -0.185964  -2.301766
 H      5.398580   2.688629   0.906511
 H      3.287178   1.855935   1.962298
 C      4.978538  -4.399406   1.825942
 H      4.582991  -5.386825   1.505260
 H      6.078957  -4.397920   1.670678
 H      4.783275  -4.295992   2.914736
 H      6.170878   1.639166  -1.281584
 C     -5.302136   3.235826   2.837486
 H     -5.722673   2.585519   3.629955
 H     -5.042814   4.208854   3.309800
 H     -6.099223   3.443141   2.094323
 C      2.304220   5.633914  -2.614255
 H      2.416251   5.505845  -3.708912
 H      1.705981   6.552913  -2.435924
 H      3.312161   5.824061  -2.187147
---------------------------------------
Mn22/TS-ii_si
Frequencies, energies and thermodynamic properties:
 Lowest Vibrational Mode (1/cm) =-236.9715
 2nd Lowest Vibrational Mode (1/cm) =                      13.1505
 E(RB-P86) (a.u.) =                                     -4753.87814373
 Thermal correction to Enthalpy (a.u.) =                  0.768612
 Thermal correction to Gibbs Free Energy (a.u.) =         0.634661
 Total Entropy (cal/Kmol) =                               281.925
 E(RPBE1PBE) (a.u.) =                                   -4753.18276713
Optimised cartesian coordinates (Angstrom):
```

Fe-2.699055	-2.023568	-1.546033	
Mn1.175593	0.208890	0.324288	
P	-1.062738	0.553078	0.331594
O	1.616988	0.810001	3.170418
O	1.144698	-2.655002	0.991329
N	1.161089	-0.089757	-1.760959
N	1.673456	2.091402	-0.316269
C	-1.883894	-0.183389	-1.144395
C	-1.181033	-0.832105	-2.251844
C	-2.156433	-1.083464	-3.288628
H	-1.961196	-1.576228	-4.249248
C	-3.440827	-0.618547	-2.840921
H	-4.383730	-0.696138	-3.398729
C	-3.280702	-0.070882	-1.522823
H	-4.078464	0.364045	-0.907295
C	-2.071059	-3.453034	-0.202216
H	-1.186514	-3.374396	0.443000
C	-2.092987	-3.991766	-1.536682
H	-1.232352	-4.402710	-2.081528
C	-3.436892	-3.876426	-2.042813
H	-3.778714	-4.177875	-3.042066
C	-4.247119	-3.268597	-1.018213
H	-5.315100	-3.025940	-1.100483
C	-3.403551	-3.004462	0.118339
H	-3.711962	-2.526050	1.056989
C	-1.554510	2.345151	0.230091
C	-1.317437	3.160319	1.364492
H	-0.924248	2.709549	2.290838
C	-1.585321	4.535773	1.331550
H	-1.400876	5.142892	2.233687
C	-2.085067	5.159720	0.162717
C	-2.303187	4.346528	-0.968575
H	-2.691393	4.801224	-1.895238
C	-2.043993	2.962513	-0.939597
H	-2.244393	2.362254	-1.840724
C	-2.106887	-0.009187	1.766192
C	-1.639350	-1.008539	2.644794
H	-0.641129	-1.445094	2.498688
C	-2.439960	-1.465414	3.706590
H	-2.049395	-2.249707	4.376049
C	-3.730484	-0.939193	3.933443
C	-4.191516	0.067326	3.053051
H	-5.193761	0.502201	3.203590
C	-3.395299	0.529194	1.993433
H	-3.782486	1.328040	1.341446
C	0.301102	-1.182398	-2.325794
H	0.490234	-2.046042	-1.657531
C	1.033062	1.196665	-2.478092
H	1.487261	1.166754	-3.492001
H	-0.049171	1.418366	-2.619628
C	1.640948	2.305138	-1.664181
C	2.131865	3.099009	0.475424
H	2.141077	2.901013	1.556741
C	2.581455	4.323365	-0.030741
H	2.941297	5.095577	0.665064
C	2.570176	4.533648	-1.420228
C	2.092436	3.503282	-2.243178
H	2.059429	3.618755	-3.337067
C	0.745233	-1.591410	-3.740826
H	0.228146	-2.519977	-4.054583
H	0.526336	-0.810854	-4.497768
H	1.835080	-1.795532	-3.749046
C	1.418407	0.572192	2.025398
C	1.101853	-1.503649	0.710872
H	2.167516	-0.393420	-1.890049
H	2.791590	-0.110040	0.202515
C	4.197833	-0.437212	-0.698927
C	4.586517	-1.608320	0.163728
C	4.969819	0.768454	-0.109720
C	5.265552	-1.151816	1.315538
C	4.413377	-2.978356	-0.091637
C	5.309609	0.366244	1.342090
H	5.900565	0.855395	-0.716723
C	5.776629	-2.079350	2.238239
C	4.928701	-3.904796	0.832366
H	3.891056	-3.308286	-1.003974
H	6.283549	0.761490	1.697897
C	5.603442	-3.456389	1.988967
H	6.313026	-1.741506	3.140332
H	4.812826	-4.985976	0.653752
H	6.006355	-4.193328	2.702530
O	3.828726	-0.524750	-1.903524
H	4.422327	1.724442	-0.210737
H	4.532799	0.746654	2.043916
H	2.926736	5.480292	-1.853897

```
 C     -2.371601   6.643850   0.139889
 H     -2.734187   6.975501  -0.853087
 H     -3.141799   6.916839   0.892953
 H     -1.463435   7.233962   0.387486
 C     -4.578666  -1.406466   5.093837
 H     -4.263504  -2.405510   5.455383
 H     -4.496683  -0.704008   5.952494
 H     -5.652589  -1.455011   4.820598
---------------------------------------
Mn22/TS-ii_re
Frequencies, energies and thermodynamic properties:
 Lowest Vibrational Mode (1/cm) =-237.6053
 2nd Lowest Vibrational Mode (1/cm) =                      12.0621
 E(RB-P86) (a.u.) =                                      -4753.87865646
 Thermal correction to Enthalpy (a.u.) =                   0.768744
 Thermal correction to Gibbs Free Energy (a.u.) =          0.634777
 Total Entropy (cal/Kmol) =                                281.956
 E(RPBE1PBE) (a.u.) =                                    -4753.18426226
Optimised cartesian coordinates (Angstrom):
 Fe-3.429662  -1.432519  -1.243400
 Mn0.994607  -0.428811   0.465011
 P     -0.952023   0.704239   0.215881
 O      1.589973   0.556445   3.173568
 O     -0.105207  -2.874254   1.668930
 N      0.873154  -1.134190  -1.516373
 N      2.169413   0.956130  -0.489877
 C     -1.986022   0.023970  -1.149128
 C     -1.574887  -1.057407  -2.044667
 C     -2.571371  -1.151652  -3.087420
 H     -2.572549  -1.874956  -3.912423
 C     -3.589925  -0.166266  -2.846890
 H     -4.492354  -0.012044  -3.453572
 C     -3.239383   0.553940  -1.654559
 H     -3.818428   1.370605  -1.204746
 C     -4.451445  -1.706582   0.519153
 H     -4.558597  -0.958924   1.315718
 C     -3.383965  -2.669181   0.403213
 H     -2.535601  -2.784260   1.090176
 C     -3.604913  -3.435006  -0.795198
 H     -2.960313  -4.238585  -1.176233
 C     -4.807283  -2.947481  -1.421392
 H     -5.236329  -3.309755  -2.365277
 C     -5.331647  -1.880938  -0.606892
 H     -6.230704  -1.287946  -0.822099
 C     -0.734588   2.486618  -0.273560
 C     -0.202405   3.375336   0.693067
 H     -0.000307   3.015262   1.715438
 C      0.063816   4.713394   0.371636
 H      0.467458   5.384640   1.148139
 C     -0.172909   5.217798  -0.930219
 C     -0.687217   4.324586  -1.892688
 H     -0.883373   4.685742  -2.916037
 C     -0.964556   2.981035  -1.574097
 H     -1.380455   2.321917  -2.351933
 C     -2.134074   0.880921   1.642735
 C     -2.075640  -0.007838   2.735929
 H     -1.310925  -0.797096   2.761047
 C     -2.992372   0.095372   3.797381
 H     -2.924607  -0.615001   4.637971
 C     -3.994130   1.090056   3.809219
 C     -4.042839   1.984005   2.713734
 H     -4.810167   2.775925   2.693271
 C     -3.128677   1.887206   1.653385
 H     -3.186898   2.613883   0.827723
 C     -0.331967  -1.931461  -1.922508
 H     -0.483649  -2.636644  -1.081374
 C      1.234609  -0.072437  -2.479551
 H      1.651949  -0.479875  -3.425828
 H      0.311971   0.487402  -2.754619
 C      2.204499   0.891006  -1.852208
 C      2.970263   1.867143   0.124335
 H      2.924544   1.891603   1.222347
 C      3.816629   2.733659  -0.575701
 H      4.435900   3.451132  -0.017584
 C      3.862334   2.658253  -1.977783
 C      3.046362   1.714753  -2.618884
 H      3.045837   1.614596  -3.714813
 C     -0.068165  -2.772700  -3.183098
 H     -0.897466  -3.488764  -3.349278
 H      0.030414  -2.151767  -4.096844
 H      0.862422  -3.361473  -3.053246
 C      1.339708   0.173502   2.079724
 C      0.281235  -1.867447   1.170710
 H      1.691215  -1.807908  -1.501904
 H      2.389006  -1.315489   0.526347
```

```
 C      3.506416  -2.403059  -0.145127
 C      4.689027  -1.593637   0.313989
 C      3.296486  -3.467679   0.959618
 C      4.987112  -1.901190   1.661081
 C      5.485895  -0.708187  -0.430364
 C      3.974897  -2.886536   2.219079
 C      6.097492  -1.307580   2.283225
 C      6.599000  -0.117851   0.195013
 H      5.240999  -0.498596  -1.484017
 C      6.899678  -0.415188   1.542185
 H      6.348322  -1.539957   3.331426
 H      7.245921   0.574116  -0.367890
 H      7.778071   0.050166   2.018079
 O      3.168954  -2.553666  -1.353139
 H      4.438776  -3.655088   2.871360
 H      3.231126  -2.341497   2.844007
 H      2.236797  -3.752712   1.093578
 H      3.845589  -4.371240   0.606893
 H      4.523237   3.318014  -2.560350
 C      0.123908   6.661408  -1.266532
 H     -0.159923   6.905452  -2.309286
 H     -0.422032   7.353971  -0.591006
 H      1.205201   6.888117  -1.146220
 C     -4.963006   1.220274   4.961888
 H     -4.985121   0.303563   5.583912
 H     -4.678654   2.065982   5.625914
 H     -5.993547   1.426298   4.606030
---------------------------------------
Mn22/TS-iii
Frequencies, energies and thermodynamic properties:
 Lowest Vibrational Mode (1/cm) =-635.2931
 2nd Lowest Vibrational Mode (1/cm) =                      18.4050
 E(RB-P86) (a.u.) =                                       -4331.13397333
 Thermal correction to Enthalpy (a.u.) =                   0.613544
 Thermal correction to Gibbs Free Energy (a.u.) =          0.499461
 Total Entropy (cal/Kmol) =                                240.109
 E(RPBE1PBE) (a.u.) =                                     -4330.44652652
Optimised cartesian coordinates (Angstrom):
 Fe2.809313  -1.107073   1.077502
 Mn-0.995476  -0.749468  -1.889627
 P     -0.187788   0.437951  -0.126078
 O     -1.650084   1.594857  -3.567813
 O      1.567064  -0.965735  -3.318169
 N     -0.823359  -2.530690  -0.807713
 N     -2.911127  -0.903155  -1.104794
 C      0.812579  -0.630150   0.985602
 C      1.001261  -2.073082   0.829470
 C      1.676141  -2.537984   2.019338
 H      1.985661  -3.572397   2.215854
 C      1.918332  -1.420768   2.892310
 H      2.436226  -1.457002   3.860361
 C      1.396546  -0.242239   2.257960
 H      1.427804   0.774811   2.669319
 C      3.793147  -0.695935  -0.688211
 H      3.316410  -0.544884  -1.664900
 C      4.227187  -1.958501  -0.152072
 H      4.145278  -2.933935  -0.650223
 C      4.766018  -1.723060   1.163549
 H      5.163012  -2.487313   1.845209
 C      4.667436  -0.311639   1.438458
 H      4.976529   0.187160   2.366943
 C      4.063131   0.323208   0.295817
 H      3.828973   1.391121   0.196014
 C     -1.502729   1.124392   0.996088
 C     -2.275787   2.213258   0.524143
 H     -2.050240   2.662532  -0.457154
 C     -3.322729   2.738750   1.293962
 H     -3.902217   3.592817   0.905250
 C     -3.650502   2.192589   2.558525
 C     -2.886198   1.098940   3.015881
 H     -3.116378   0.650037   3.996466
 C     -1.829737   0.569855   2.250022
 H     -1.250977  -0.278507   2.647067
 C      0.856725   1.955203  -0.391588
 C      1.499860   2.184190  -1.625588
 H      1.368430   1.477292  -2.456574
 C      2.322721   3.309509  -1.811214
 H      2.814107   3.461075  -2.786534
 C      2.529276   4.247802  -0.777141
 C      1.875317   4.017591   0.456066
 H      2.011046   4.733268   1.284059
 C      1.050472   2.898558   0.645594
 H      0.543194   2.766189   1.614143
 C      0.507880  -2.930072  -0.346886
 H      1.192686  -2.749995  -1.201860
```

```
 C     -1.871642  -2.679592   0.181726
 H     -2.146425  -3.746487   0.378478
 H     -1.577737  -2.271449   1.191170
 C     -3.108762  -1.929880  -0.230590
 C     -3.980383  -0.153007  -1.476837
 C     -5.275735  -0.390701  -1.006447
 C     -5.487974  -1.456568  -0.114241
 C     -4.388366  -2.233500   0.271949
 C      0.597553  -4.437810  -0.023663
 H      1.647845  -4.746687   0.156354
 H      0.009234  -4.712368   0.876615
 H      0.210746  -5.023626  -0.882152
 C     -1.388533   0.657618  -2.896715
 C      0.571049  -0.864094  -2.686776
 H     -1.304971  -2.345859  -2.313878
 H     -1.591073  -1.854857  -3.044310
 H     -3.781831   0.663201  -2.186325
 H     -6.102432   0.250680  -1.345447
 H     -4.506276  -3.080496   0.964663
 H     -6.495338  -1.679407   0.269945
 C     -4.781315   2.768476   3.379843
 H     -4.914831   2.221114   4.333743
 H     -4.599270   3.837550   3.621862
 H     -5.742938   2.728649   2.824819
 C      3.390444   5.472504  -0.983148
 H      4.054054   5.363309  -1.863748
 H      2.762561   6.375240  -1.150208
 H      4.020179   5.681350  -0.093928
---------------------------------------
Mn23/i
Frequencies, energies and thermodynamic properties:
 Lowest Vibrational Mode (1/cm) =                          23.4666
 2nd Lowest Vibrational Mode (1/cm) =                      29.1672
 E(RB-P86) (a.u.) =                                      -4449.72493819
 Thermal correction to Enthalpy (a.u.) =                   0.527859
 Thermal correction to Gibbs Free Energy (a.u.) =          0.420413
 Total Entropy (cal/Kmol) =                                226.141
 E(RPBE1PBE) (a.u.) =                                    -4449.10631943
Optimised cartesian coordinates (Angstrom):
 Fe2.811094  -1.194522   0.999084
 Mn-1.026635  -0.631615  -1.760023
 P     -0.148047   0.499330  -0.108183
 O     -1.780881   1.655688  -3.495838
 O      1.416349  -0.869084  -3.376483
 N     -0.866051  -2.307894  -0.884771
 N     -2.975339  -0.842472  -1.114385
 C      0.839971  -0.627046   0.964534
 C      0.958564  -2.069797   0.765721
 C      1.635866  -2.606148   1.921894
 H      1.902418  -3.659299   2.078226
 C      1.942218  -1.527390   2.823867
 H      2.474416  -1.617252   3.780616
 C      1.461885  -0.306294   2.237073
 H      1.544124   0.693569   2.682312
 C      3.761346  -0.802208  -0.787463
 H      3.263766  -0.627824  -1.750427
 C      4.167896  -2.082338  -0.271335
 H      4.040207  -3.050848  -0.773365
 C      4.748956  -1.875382   1.030917
 H      5.137652  -2.657853   1.696383
 C      4.704297  -0.464269   1.318592
 H      5.053721   0.017024   2.241916
 C      4.091575   0.199044   0.196848
 H      3.894101   1.275427   0.111688
 C     -1.444867   1.152075   1.052910
 C     -2.182166   2.297281   0.665721
 H     -1.926440   2.828981  -0.265078
 C     -3.239647   2.777418   1.452553
 H     -3.811076   3.672538   1.164382
 C     -3.570790   2.089538   2.630544
 C     -2.873988   0.944231   3.035654
 H     -3.161560   0.430452   3.965274
 C     -1.810632   0.479842   2.240217
 H     -1.254305  -0.412829   2.563916
 C      0.914387   2.003935  -0.350301
 C      1.515461   2.256837  -1.602668
 H      1.336601   1.577446  -2.447920
 C      2.350218   3.371206  -1.793329
 H      2.820117   3.576257  -2.766822
 C      2.576774   4.237559  -0.715254
 C      1.988494   4.023265   0.540091
 H      2.179667   4.730542   1.361021
 C      1.155708   2.906322   0.713036
 H      0.678702   2.750020   1.692922
 C      0.439591  -2.842923  -0.457659
```

```
 H      1.135515  -2.632660  -1.294050
 C     -1.973895  -2.762335  -0.065605
 H     -2.206901  -3.844175  -0.219920
 H     -1.750805  -2.675369   1.031941
 C     -3.199770  -1.953095  -0.354934
 C     -4.036708  -0.037616  -1.403091
 H     -3.822375   0.850467  -2.014688
 C     -5.334431  -0.311031  -0.967622
 H     -6.151528   0.374256  -1.237286
 C     -5.568655  -1.464588  -0.193322
 C     -4.482690  -2.293290   0.112712
 H     -4.613159  -3.205739   0.714377
 C      0.461073  -4.366618  -0.235220
 H      1.503825  -4.720510  -0.105897
 H     -0.111592  -4.679074   0.661832
 H      0.037541  -4.884610  -1.119871
 C     -1.472675   0.746672  -2.800542
 C      0.467203  -0.762439  -2.671762
 H     -6.581033  -1.709768   0.162487
 F      3.373461   5.313748  -0.891439
 F     -4.590222   2.545815   3.392459
---------------------------------------
Mn23/ii
Frequencies, energies and thermodynamic properties:
 Lowest Vibrational Mode (1/cm) =                          16.9718
 2nd Lowest Vibrational Mode (1/cm) =                      23.6846
 E(RB-P86) (a.u.) =                                       -4604.65966354
 Thermal correction to Enthalpy (a.u.) =                   0.612620
 Thermal correction to Gibbs Free Energy (a.u.) =          0.489841
 Total Entropy (cal/Kmol) =                                258.410
 E(RPBE1PBE) (a.u.) =                                     -4604.04983945
Optimised cartesian coordinates (Angstrom):
 Fe-2.615711  -1.588523  -1.337688
 Mn1.235156  -0.377914   1.192480
 P     -0.348900   0.733431   0.168880
 O      1.384772   1.322985   3.616650
 O     -0.540643  -2.149762   2.723612
 N      1.541212  -1.592225  -0.271452
 N      2.914184   0.507558   0.380977
 C     -1.044075  -0.279581  -1.200847
 C     -0.568204  -1.608823  -1.578798
 C     -1.231068  -1.964582  -2.810523
 H     -1.110779  -2.911303  -3.352462
 C     -2.107369  -0.890408  -3.195491
 H     -2.762985  -0.876293  -4.076661
 C     -2.003112   0.146276  -2.205561
 H     -2.549067   1.098412  -2.216043
 C     -3.244004  -2.230659   0.517381
 H     -2.662981  -2.168001   1.446462
 C     -3.223299  -3.327455  -0.413555
 H     -2.628849  -4.245815  -0.315833
 C     -4.099061  -2.999858  -1.509661
 H     -4.285747  -3.622073  -2.395198
 C     -4.664694  -1.699596  -1.253686
 H     -5.358625  -1.157722  -1.910240
 C     -4.134518  -1.222346  -0.002695
 H     -4.355115  -0.254369   0.465540
 C      0.321793   2.230123  -0.704501
 C      0.649402   3.369413   0.070029
 H      0.433037   3.382850   1.150486
 C      1.245652   4.495453  -0.516382
 H      1.494568   5.388675   0.076010
 C      1.530554   4.469439  -1.890674
 C      1.233655   3.354142  -2.684049
 H      1.470320   3.369673  -3.758526
 C      0.628456   2.235429  -2.083271
 H      0.382299   1.364060  -2.708704
 C     -1.817046   1.453944   1.048811
 C     -2.185732   0.989923   2.330403
 H     -1.590435   0.213734   2.831078
 C     -3.315240   1.504960   2.988959
 H     -3.607182   1.150328   3.988689
 C     -4.075173   2.495819   2.352834
 C     -3.732897   2.987723   1.084453
 H     -4.345728   3.774408   0.619447
 C     -2.600072   2.464623   0.441395
 H     -2.320171   2.866001  -0.544814
 C      0.454125  -2.446873  -0.799654
 H     -0.063171  -2.846329   0.095588
 C      2.480970  -1.164175  -1.298878
 H      3.123896  -1.997197  -1.672282
 H      1.949285  -0.783190  -2.210865
 C      3.351428  -0.062154  -0.778408
 C      3.636784   1.535512   0.906796
 C      4.804607   2.019182   0.314204
```

```
 C      5.262706   1.425483  -0.878023
 C      4.523975   0.370226  -1.425818
 C      0.954609  -3.663925  -1.600196
 H      0.121850  -4.371126  -1.789055
 H      1.387310  -3.384299  -2.582570
 H      1.727095  -4.191755  -1.005064
 C      1.320253   0.645861   2.647640
 C      0.126477  -1.426839   2.062249
 H      2.486498  -2.827573   0.791085
 O      2.930224  -3.596198   1.253557
 C      4.333840  -3.371494   1.253065
 H      4.586451  -2.366976   1.676771
 H      4.752307  -3.387002   0.213865
 H      4.842275  -0.126854  -2.354713
 H      5.348146   2.850035   0.787476
 H      3.255722   1.971923   1.841085
 C      5.016359  -4.453049   2.083282
 H      6.116628  -4.309613   2.096323
 H      4.649738  -4.432064   3.131146
 H      4.802636  -5.460423   1.667680
 H      6.181962   1.781261  -1.367948
 F      2.106828   5.550327  -2.461662
 F     -5.159181   2.998872   2.981076
---------------------------------------
Mn23/iii
Frequencies, energies and thermodynamic properties:
 Lowest Vibrational Mode (1/cm) =                          20.6104
 2nd Lowest Vibrational Mode (1/cm) =                      26.8151
 E(RB-P86) (a.u.) =                                      -4605.83210279
 Thermal correction to Enthalpy (a.u.) =                   0.628657
 Thermal correction to Gibbs Free Energy (a.u.) =          0.506865
 Total Entropy (cal/Kmol) =                                256.331
 E(RPBE1PBE) (a.u.) =                                    -4605.21903138
Optimised cartesian coordinates (Angstrom):
 Fe-2.604523  -1.656404  -1.286836
 Mn1.252006  -0.354104   1.405202
 P     -0.320186   0.692689   0.142322
 O      0.966917   1.686954   3.530383
 O     -0.627145  -2.113383   2.834946
 N      1.587533  -1.691308  -0.163564
 N      2.847769   0.573060   0.467092
 C     -1.016362  -0.360850  -1.181638
 C     -0.551310  -1.709835  -1.489585
 C     -1.199184  -2.106445  -2.717794
 H     -1.083221  -3.075942  -3.218604
 C     -2.058688  -1.041463  -3.161315
 H     -2.701617  -1.058783  -4.051713
 C     -1.959398   0.034527  -2.214194
 H     -2.494649   0.991101  -2.270752
 C     -3.280560  -2.231429   0.574575
 H     -2.721850  -2.144210   1.514938
 C     -3.248133  -3.358504  -0.318576
 H     -2.663545  -4.277490  -0.177441
 C     -4.095541  -3.059743  -1.444830
 H     -4.266813  -3.708834  -2.314052
 C     -4.655607  -1.747179  -1.244650
 H     -5.329691  -1.221869  -1.934504
 C     -4.150066  -1.233311   0.001800
 H     -4.373845  -0.249489   0.434046
 C      0.350660   2.165660  -0.775254
 C      0.702659   3.314182  -0.025366
 H      0.526966   3.340131   1.062090
 C      1.266794   4.439004  -0.645515
 H      1.535365   5.337870  -0.070613
 C      1.491305   4.404269  -2.030281
 C      1.164957   3.281550  -2.801324
 H      1.352452   3.289889  -3.885496
 C      0.594339   2.163888  -2.165844
 H      0.326368   1.287149  -2.774676
 C     -1.807120   1.456005   0.963602
 C     -2.246870   1.014150   2.230718
 H     -1.694512   0.233319   2.770699
 C     -3.397120   1.555731   2.829364
 H     -3.741401   1.216793   3.817801
 C     -4.109475   2.551218   2.147557
 C     -3.698889   3.020689   0.891357
 H     -4.275435   3.810765   0.387360
 C     -2.545380   2.471542   0.309562
 H     -2.214462   2.856452  -0.667164
 C      0.466594  -2.511088  -0.672841
 H     -0.056686  -2.887780   0.230321
 C      2.351499  -1.069048  -1.241393
 H      2.966757  -1.800801  -1.819611
 H      1.693032  -0.582652  -2.013309
 C      3.247764   0.010153  -0.707404
```

```
 C      3.580306   1.593350   0.984070
 C      4.733219   2.087402   0.366376
 C      5.158351   1.503324  -0.840143
 C      4.404467   0.452582  -1.377787
 C      0.952454  -3.758339  -1.444094
 H      0.109257  -4.449413  -1.649212
 H      1.417711  -3.500101  -2.418096
 H      1.698676  -4.298958  -0.827895
 C      1.097367   0.867616   2.690385
 C      0.081821  -1.394400   2.222071
 H      2.517320  -0.848465   2.477695
 H      2.455889  -1.525862   2.001781
 H      2.548801  -2.755013   0.590649
 O      3.104267  -3.408926   1.190706
 C      4.476865  -3.307286   0.857993
 H      4.659332  -3.570648  -0.216354
 H      4.856909  -2.259830   0.985343
 H      4.700579  -0.036251  -2.318140
 H      5.287766   2.914122   0.833851
 H      3.224519   2.019739   1.933320
 C      5.295339  -4.243639   1.743748
 H      4.959100  -5.294375   1.615562
 H      6.375580  -4.191676   1.492978
 H      5.175013  -3.977716   2.815361
 H      6.065780   1.860977  -1.350639
 F      2.035557   5.483524  -2.634309
 F     -5.212997   3.079976   2.718113
---------------------------------------
Mn23/iv
Frequencies, energies and thermodynamic properties:
 Lowest Vibrational Mode (1/cm) =                          17.8504
 2nd Lowest Vibrational Mode (1/cm) =                      22.5196
 E(RB-P86) (a.u.) =                                       -4605.86082047
 Thermal correction to Enthalpy (a.u.) =                   0.633250
 Thermal correction to Gibbs Free Energy (a.u.) =          0.511025
 Total Entropy (cal/Kmol) =                                257.245
 E(RPBE1PBE) (a.u.) =                                     -4605.24597814
Optimised cartesian coordinates (Angstrom):
 Fe-2.707906  -1.503747  -1.327472
 Mn1.322549  -0.469417   1.206037
 P     -0.316669   0.707224   0.152944
 O      1.371577   1.265397   3.579021
 O     -0.352482  -2.393713   2.671984
 N      1.538451  -1.710462  -0.539364
 N      2.889482   0.467676   0.258996
 C     -1.115786  -0.216007  -1.224978
 C     -0.687536  -1.536856  -1.688277
 C     -1.425012  -1.825037  -2.897777
 H     -1.354240  -2.745380  -3.490709
 C     -2.303949  -0.723294  -3.179879
 H     -3.010842  -0.662467  -4.018174
 C     -2.123289   0.264675  -2.153139
 H     -2.653973   1.222969  -2.084792
 C     -3.220648  -2.235059   0.527894
 H     -2.574405  -2.230654   1.415420
 C     -3.284400  -3.280271  -0.459501
 H     -2.702729  -4.211921  -0.452142
 C     -4.227394  -2.880892  -1.472682
 H     -4.485408  -3.451015  -2.375188
 C     -4.750394  -1.588895  -1.108870
 H     -5.477428  -1.001685  -1.685723
 C     -4.127188  -1.188013   0.125547
 H     -4.296675  -0.243470   0.658115
 C      0.280399   2.262098  -0.684423
 C      0.729018   3.319726   0.144882
 H      0.643517   3.233186   1.240343
 C      1.277454   4.489917  -0.401534
 H      1.618265   5.318287   0.237656
 C      1.394002   4.593225  -1.796229
 C      0.976553   3.562389  -2.646981
 H      1.082389   3.676738  -3.736323
 C      0.420505   2.398990  -2.082972
 H      0.079998   1.596928  -2.755678
 C     -1.749329   1.388156   1.132020
 C     -2.076748   0.830230   2.387174
 H     -1.474103   0.007034   2.796124
 C     -3.170549   1.305867   3.130752
 H     -3.429287   0.876260   4.110139
 C     -3.937425   2.355516   2.607288
 C     -3.636927   2.941167   1.368667
 H     -4.254323   3.770894   0.992829
 C     -2.539321   2.454244   0.640319
 H     -2.293072   2.928069  -0.322593
 C      0.333299  -2.459455  -1.033895
 H     -0.117619  -2.879171  -0.112533
```

```
 C      2.263301  -0.972044  -1.595300
 H      2.792296  -1.643609  -2.305840
 H      1.518269  -0.401810  -2.194627
 C      3.227368   0.007936  -0.980779
 C      3.684570   1.414902   0.823466
 C      4.831321   1.919844   0.200121
 C      5.192560   1.429474  -1.065749
 C      4.372841   0.457123  -1.658763
 C      0.727832  -3.642981  -1.933828
 H     -0.151728  -4.286317  -2.134904
 H      1.138223  -3.315869  -2.910973
 H      1.487479  -4.267862  -1.421860
 C      1.343874   0.570944   2.617876
 C      0.275100  -1.601204   2.050659
 H      2.510043  -1.316697   1.868593
 H      2.878028  -2.706138   1.557991
 H      2.183115  -2.435482  -0.156942
 O      3.147301  -3.539379   1.059541
 C      4.577589  -3.583892   1.026329
 H      4.851675  -4.329328   0.248342
 H      5.000197  -2.604967   0.694729
 H      4.610398   0.041302  -2.649651
 H      5.432826   2.684173   0.714053
 H      3.386485   1.766939   1.821498
 C      5.181794  -3.985558   2.371407
 H      4.781654  -4.965802   2.704632
 H      6.287071  -4.067572   2.299233
 H      4.947755  -3.234057   3.155136
 H      6.094089   1.795110  -1.580417
 F     -4.986981   2.823615   3.318095
 F      1.924351   5.717175  -2.330478
---------------------------------------
Mn23/v
Frequencies, energies and thermodynamic properties:
 Lowest Vibrational Mode (1/cm) =                          22.6824
 2nd Lowest Vibrational Mode (1/cm) =                      29.8928
 E(RB-P86) (a.u.) =                                      -4450.92247830
 Thermal correction to Enthalpy (a.u.) =                   0.548821
 Thermal correction to Gibbs Free Energy (a.u.) =          0.441018
 Total Entropy (cal/Kmol) =                                226.890
 E(RPBE1PBE) (a.u.) =                                    -4450.30358091
Optimised cartesian coordinates (Angstrom):
 Fe-2.826152  -1.100063  -1.037552
 Mn1.055050  -0.662412   1.865843
 P      0.189148   0.470318   0.094661
 O      1.737398   1.716847   3.450318
 O     -1.370566  -1.031238   3.487880
 N      0.814028  -2.515946   0.802343
 N      2.936722  -0.899768   1.082996
 C     -0.840278  -0.586748  -1.009974
 C     -1.008696  -2.032422  -0.849428
 C     -1.709063  -2.514571  -2.019107
 H     -2.011233  -3.552399  -2.206662
 C     -1.987958  -1.401763  -2.884601
 H     -2.533662  -1.448296  -3.836517
 C     -1.462480  -0.216056  -2.267946
 H     -1.518634   0.799559  -2.680286
 C     -3.742438  -0.726732   0.769013
 H     -3.228224  -0.590812   1.729626
 C     -4.191916  -1.981837   0.226317
 H     -4.086761  -2.966104   0.702344
 C     -4.784197  -1.725267  -1.061623
 H     -5.202544  -2.479462  -1.741541
 C     -4.704060  -0.309282  -1.313739
 H     -5.051035   0.205030  -2.219954
 C     -4.058412   0.307958  -0.184627
 H     -3.828514   1.375577  -0.075788
 C      1.462038   1.164047  -1.078495
 C      2.271863   2.227726  -0.608695
 H      2.079001   2.667654   0.383402
 C      3.318953   2.743947  -1.387260
 H      3.944070   3.576734  -1.031294
 C      3.569711   2.173724  -2.644793
 C      2.802556   1.110019  -3.135280
 H      3.026887   0.686042  -4.125727
 C      1.750069   0.610513  -2.345546
 H      1.140206  -0.216341  -2.740854
 C     -0.865025   1.981915   0.374242
 C     -1.488345   2.179303   1.625673
 H     -1.331992   1.451083   2.434429
 C     -2.313915   3.292769   1.858541
 H     -2.801266   3.453258   2.831951
 C     -2.507836   4.217159   0.823113
 C     -1.897099   4.059799  -0.429777
 H     -2.063166   4.811194  -1.216420
```

```
 C     -1.074652   2.941575  -0.644023
 H     -0.580332   2.828880  -1.621467
 C     -0.559976  -2.894923   0.324251
 H     -1.213275  -2.671866   1.191165
 C      1.873550  -2.653807  -0.220739
 H      2.092676  -3.712809  -0.476155
 H      1.505406  -2.170491  -1.153379
 C      3.125868  -1.944760   0.224045
 C      4.029828  -0.176199   1.445919
 H      3.849516   0.660785   2.135644
 C      5.323401  -0.467107   0.997703
 H      6.163656   0.158198   1.334300
 C      5.519948  -1.558236   0.135128
 C      4.396359  -2.304986  -0.252691
 H      4.493667  -3.168012  -0.928820
 C     -0.672202  -4.399880   0.029971
 H     -1.730215  -4.680845  -0.141233
 H     -0.092964  -4.702386  -0.866013
 H     -0.311346  -4.990314   0.897296
 C      1.453018   0.766006   2.798535
 C     -0.426899  -0.858972   2.788110
 H      1.036338  -3.146979   1.584492
 H      1.630232  -1.584882   3.020822
 H      6.524741  -1.823109  -0.227443
 F     -3.294821   5.294460   1.039811
 F      4.579826   2.663979  -3.400016
---------------------------------------
Mn23/vi_R
Frequencies, energies and thermodynamic properties:
 Lowest Vibrational Mode (1/cm) =                        14.1307
 2nd Lowest Vibrational Mode (1/cm) =                    19.1875
 E(RB-P86) (a.u.) =                                     -4873.63469373
 Thermal correction to Enthalpy (a.u.) =                 0.701242
 Thermal correction to Gibbs Free Energy (a.u.) =        0.570735
 Total Entropy (cal/Kmol) =                              274.677
 E(RPBE1PBE) (a.u.) =                                   -4873.01112388
Optimised cartesian coordinates (Angstrom):
 Fe-2.720403  -2.130111  -1.492877
 Mn1.008900   0.263082   0.310631
 P     -1.175995   0.510979   0.361484
 O      1.457802   0.948882   3.158222
 O      1.011906  -2.566129   1.116614
 N      1.054582  -0.066456  -1.622174
 N      1.529150   2.150200  -0.333938
 C     -1.952393  -0.268299  -1.109913
 C     -1.216466  -0.909466  -2.197654
 C     -2.170278  -1.200109  -3.241785
 H     -1.947735  -1.701146  -4.192359
 C     -3.473591  -0.762178  -2.818903
 H     -4.406527  -0.868949  -3.388663
 C     -3.348570  -0.195282  -1.504424
 H     -4.167425   0.228016  -0.908722
 C     -2.095664  -3.523747  -0.108325
 H     -1.235012  -3.415306   0.563901
 C     -2.059535  -4.081429  -1.434025
 H     -1.170484  -4.475434  -1.944561
 C     -3.388726  -4.009562  -1.985124
 H     -3.689401  -4.334244  -2.990272
 C     -4.248294  -3.409482  -0.996655
 H     -5.319250  -3.197794  -1.116770
 C     -3.449389  -3.106380   0.162433
 H     -3.803560  -2.626632   1.084058
 C     -1.673249   2.295658   0.216502
 C     -1.503047   3.131999   1.346517
 H     -1.151201   2.706622   2.300274
 C     -1.781345   4.505016   1.278341
 H     -1.660955   5.160499   2.153849
 C     -2.219272   5.044913   0.058883
 C     -2.385262   4.249901  -1.082083
 H     -2.731659   4.707397  -2.020906
 C     -2.109504   2.873197  -0.996200
 H     -2.253574   2.245876  -1.888807
 C     -2.209599  -0.030291   1.807652
 C     -1.720521  -0.980649   2.730303
 H     -0.708569  -1.393187   2.618438
 C     -2.511805  -1.420600   3.804998
 H     -2.135377  -2.158395   4.529122
 C     -3.803563  -0.897574   3.951663
 C     -4.319389   0.054278   3.059727
 H     -5.333960   0.452183   3.211138
 C     -3.514635   0.485731   1.993564
 H     -3.913430   1.248560   1.307270
 C      0.291035  -1.190002  -2.216087
 H      0.478827  -2.053187  -1.546298
 C      1.055277   1.126723  -2.466908
```

```
 H      1.684294   1.006973  -3.380849
 H      0.027210   1.360357  -2.848911
 C      1.538021   2.311933  -1.687852
 C      1.921164   3.196771   0.444121
 H      1.900360   3.031985   1.530795
 C      2.338742   4.419070  -0.086759
 H      2.647201   5.227645   0.591893
 C      2.359789   4.584169  -1.484862
 C      1.956198   3.513249  -2.290696
 H      1.957105   3.594276  -3.388127
 C      0.784476  -1.601609  -3.616796
 H      0.304145  -2.550519  -3.929841
 H      0.558047  -0.840516  -4.391310
 H      1.879962  -1.770980  -3.598253
 C      1.280731   0.677003   2.020176
 C      0.953354  -1.434424   0.771513
 H      2.641567  -0.499891  -1.717516
 H      3.172579  -0.132179   0.234341
 C      4.088407  -0.368818  -0.422445
 C      4.854223  -1.475647   0.289741
 C      5.064359   0.837771  -0.316050
 C      5.880476  -0.928571   1.091464
 C      4.652731  -2.862474   0.221392
 C      5.886769   0.586497   0.971495
 H      5.726033   0.791045  -1.208852
 C      6.712488  -1.773379   1.845336
 C      5.489312  -3.709506   0.975242
 H      3.857716  -3.275527  -0.419893
 H      6.909648   1.016897   0.937335
 C      6.510587  -3.167728   1.782745
 H      7.520765  -1.357139   2.469502
 H      5.350371  -4.801951   0.930059
 H      7.162374  -3.840697   2.363274
 O      3.657234  -0.675268  -1.712782
 H      4.544841   1.816899  -0.331507
 H      5.382829   1.041097   1.855557
 H      2.688397   5.533016  -1.935776
 F     -2.487638   6.367178  -0.014299
 F     -4.568378  -1.311694   4.984179
---------------------------------------
Mn23/vi_S
Frequencies, energies and thermodynamic properties:
 Lowest Vibrational Mode (1/cm) =                          11.7664
 2nd Lowest Vibrational Mode (1/cm) =                      17.3672
 E(RB-P86) (a.u.) =                                      -4873.63498054
 Thermal correction to Enthalpy (a.u.) =                   0.701278
 Thermal correction to Gibbs Free Energy (a.u.) =          0.570232
 Total Entropy (cal/Kmol) =                                275.809
 E(RPBE1PBE) (a.u.) =                                    -4873.01157110
Optimised cartesian coordinates (Angstrom):
 Fe-3.331314  -1.650325  -1.295025
 Mn0.888064  -0.237243   0.458193
 P     -1.104733   0.674135   0.266641
 O      1.411043   0.757445   3.200129
 O     -0.037724  -2.746876   1.679776
 N      0.902156  -0.885265  -1.393374
 N      2.022832   1.251270  -0.408137
 C     -2.030784  -0.073321  -1.132847
 C     -1.493597  -1.086294  -2.038784
 C     -2.449685  -1.249404  -3.108118
 H     -2.359843  -1.950630  -3.947397
 C     -3.563858  -0.369421  -2.876376
 H     -4.460234  -0.283706  -3.505312
 C     -3.316780   0.351455  -1.658056
 H     -3.982516   1.100308  -1.210155
 C     -3.242378  -2.893031   0.347749
 H     -2.427958  -2.926396   1.082458
 C     -3.310824  -3.665530  -0.863978
 H     -2.561990  -4.390453  -1.210434
 C     -4.521554  -3.303997  -1.556196
 H     -4.854830  -3.700875  -2.524483
 C     -5.203736  -2.308451  -0.768973
 H     -6.148550  -1.814429  -1.032540
 C     -4.412270  -2.051779   0.406404
 H     -4.648465  -1.331058   1.199927
 C     -1.002620   2.471568  -0.193817
 C     -0.612648   3.395975   0.805600
 H     -0.445349   3.053206   1.839482
 C     -0.438381   4.754812   0.505241
 H     -0.144716   5.481489   1.277471
 C     -0.642583   5.185521  -0.815070
 C     -1.015328   4.296748  -1.831214
 H     -1.168085   4.670330  -2.854829
 C     -1.193542   2.938105  -1.513081
 H     -1.500537   2.242169  -2.308348
```

```
 C     -2.312707   0.736013   1.677182
 C     -2.184955  -0.139036   2.777885
 H     -1.349766  -0.850950   2.827089
 C     -3.116788  -0.121209   3.829544
 H     -3.020822  -0.798275   4.691388
 C     -4.183069   0.786208   3.772351
 C     -4.336851   1.676483   2.699297
 H     -5.179797   2.383591   2.691955
 C     -3.395449   1.647855   1.658471
 H     -3.506659   2.361544   0.827655
 C     -0.151421  -1.809170  -1.876635
 H     -0.270777  -2.558565  -1.068478
 C      1.300935   0.097497  -2.400021
 H      1.876655  -0.355474  -3.241507
 H      0.408832   0.577138  -2.881532
 C      2.120616   1.179731  -1.766171
 C      2.714225   2.229077   0.239128
 H      2.613815   2.255533   1.333516
 C      3.518243   3.155283  -0.428644
 H      4.055153   3.922265   0.148357
 C      3.626413   3.079215  -1.830130
 C      2.920194   2.074391  -2.501982
 H      2.975593   1.973520  -3.596344
 C      0.244395  -2.588051  -3.146146
 H     -0.495918  -3.386796  -3.353867
 H      0.300583  -1.941611  -4.045869
 H      1.229944  -3.074155  -2.998553
 C      1.206472   0.361204   2.104060
 C      0.275104  -1.727009   1.162881
 H      2.289961  -1.770369  -1.301021
 H      2.841231  -1.252917   0.613258
 C      3.587150  -1.985242   0.130296
 C      3.626489  -3.256502   1.026496
 C      4.629435  -2.924640   2.158710
 O      3.223812  -2.194679  -1.199560
 H      4.255015   3.791141  -2.386600
 C      4.973446  -1.393060   0.336501
 C      5.567526  -1.924415   1.502990
 C      5.651319  -0.455336  -0.457193
 C      6.851798  -1.507718   1.891762
 C      6.940826  -0.040548  -0.068420
 H      5.182177  -0.065118  -1.374953
 C      7.534888  -0.561702   1.099433
 H      7.328023  -1.920789   2.796584
 H      7.492257   0.689794  -0.682629
 H      8.546287  -0.234219   1.390214
 H      5.155493  -3.814942   2.562827
 H      4.107159  -2.449341   3.021017
 H      4.024599  -4.079461   0.392899
 H      2.623853  -3.559365   1.387631
 F     -0.474877   6.493053  -1.111532
 F     -5.080035   0.812788   4.780907
---------------------------------------
Mn23/viii
Frequencies, energies and thermodynamic properties:
 Lowest Vibrational Mode (1/cm) =                        22.0326
 2nd Lowest Vibrational Mode (1/cm) =                    29.4254
 E(RB-P86) (a.u.) =                                    -4450.88843895
 Thermal correction to Enthalpy (a.u.) =                 0.543874
 Thermal correction to Gibbs Free Energy (a.u.) =        0.435306
 Total Entropy (cal/Kmol) =                              228.500
 E(RPBE1PBE) (a.u.) =                                  -4450.27028870
Optimised cartesian coordinates (Angstrom):
 Fe2.847967  -1.091684   1.026333
 Mn-1.027937  -0.682096  -1.899882
 P     -0.185946   0.414113  -0.115643
 O     -1.649259   1.853024  -3.305242
 O      1.517773  -0.868214  -3.365711
 N     -0.791844  -2.441329  -0.873968
 N     -2.924387  -0.885198  -1.088362
 C      0.846385  -0.638062   0.972262
 C      1.044187  -2.071207   0.781412
 C      1.741042  -2.558001   1.948193
 H      2.063527  -3.593787   2.115230
 C      1.984544  -1.457769   2.843861
 H      2.514872  -1.510486   3.804466
 C      1.444122  -0.268340   2.244197
 H      1.470367   0.738309   2.680929
 C      3.798915  -0.622888  -0.743045
 H      3.306302  -0.443768  -1.707093
 C      4.246177  -1.899785  -0.254788
 H      4.157553  -2.859320  -0.781782
 C      4.807305  -1.704027   1.058057
 H      5.218121  -2.488127   1.708205
 C      4.709826  -0.302389   1.379135
```

```
 H      5.034694   0.168368   2.316794
 C      4.083530   0.365903   0.267776
 H      3.849112   1.436467   0.205549
 C     -1.505931   1.055476   1.028970
 C     -2.295533   2.146619   0.592211
 H     -2.086581   2.626269  -0.377561
 C     -3.344587   2.642736   1.380633
 H     -3.957517   3.495431   1.052170
 C     -3.610844   2.027560   2.613537
 C     -2.858167   0.938768   3.071331
 H     -3.094405   0.481817   4.044030
 C     -1.805981   0.456708   2.271578
 H     -1.207701  -0.392240   2.635540
 C      0.821833   1.962591  -0.360609
 C      1.464523   2.223591  -1.590525
 H      1.351974   1.529440  -2.434285
 C      2.261600   3.367854  -1.765395
 H      2.761566   3.576821  -2.722923
 C      2.411275   4.257675  -0.693493
 C      1.782204   4.036149   0.540393
 H      1.912439   4.760573   1.358287
 C      0.986776   2.889924   0.696247
 H      0.477933   2.729444   1.659083
 C      0.532910  -2.869941  -0.427622
 H      1.224849  -2.660355  -1.270379
 C     -1.828545  -2.686502   0.103013
 H     -2.086172  -3.771803   0.215973
 H     -1.556759  -2.364738   1.153794
 C     -3.084246  -1.939901  -0.240672
 C     -4.012373  -0.137376  -1.411388
 C     -5.291297  -0.406679  -0.916747
 C     -5.465523  -1.500233  -0.047851
 C     -4.347109  -2.271831   0.288538
 C      0.619818  -4.390848  -0.166681
 H      1.671491  -4.709998  -0.013152
 H      0.043504  -4.700472   0.730082
 H      0.218719  -4.940483  -1.042649
 C     -1.412419   0.828591  -2.764135
 C      0.527565  -0.777228  -2.725246
 H     -1.550193  -2.047802  -2.895865
 H     -1.831476  -1.420763  -3.337476
 H     -3.840868   0.703488  -2.099216
 H     -6.135477   0.231423  -1.216510
 H     -4.435495  -3.138221   0.961573
 H     -6.459583  -1.746427   0.356314
 F      3.171351   5.362293  -0.854666
 F     -4.620316   2.499907   3.378331
---------------------------------------
Mn23/ix
Frequencies, energies and thermodynamic properties:
 Lowest Vibrational Mode (1/cm) =                          24.1311
 2nd Lowest Vibrational Mode (1/cm) =                      30.3686
 E(RB-P86) (a.u.) =                                      -4604.67626873
 Thermal correction to Enthalpy (a.u.) =                   0.613124
 Thermal correction to Gibbs Free Energy (a.u.) =          0.496015
 Total Entropy (cal/Kmol) =                                246.477
 E(RPBE1PBE) (a.u.) =                                    -4604.06190259
Optimised cartesian coordinates (Angstrom):
 Fe-3.013474  -0.836624  -1.284921
 Mn1.226429  -0.994476   1.099229
 P     -0.031319   0.590577   0.097681
 O      1.840913   0.737215   3.396781
 O     -1.000919  -2.182008   2.608225
 N      0.995215  -2.234753  -0.619743
 N      2.940823  -0.490158   0.029317
 C     -1.101699  -0.090511  -1.242187
 C     -1.106183  -1.482225  -1.695471
 C     -1.931702  -1.545099  -2.879443
 H     -2.158515  -2.449737  -3.457370
 C     -2.444971  -0.231759  -3.158016
 H     -3.124279   0.033726  -3.979098
 C     -1.945240   0.663851  -2.152478
 H     -2.160564   1.738024  -2.089475
 C     -3.691458  -1.319859   0.600807
 H     -3.062342  -1.468758   1.487715
 C     -4.064228  -2.338769  -0.344514
 H     -3.774335  -3.397280  -0.300817
 C     -4.864990  -1.723474  -1.371856
 H     -5.286847  -2.228636  -2.251058
 C     -4.989546  -0.322579  -1.059315
 H     -5.523712   0.426626  -1.658915
 C     -4.262083  -0.071892   0.157799
 H     -4.146068   0.900910   0.652740
 C      0.959340   1.893095  -0.795929
 C      1.666382   2.837758  -0.012630
```

```
 H      1.561552   2.832176   1.084472
 C      2.501281   3.795187  -0.608033
 H      3.045696   4.537793  -0.005532
 C      2.640785   3.793807  -2.004516
 C      1.968567   2.866267  -2.809867
 H      2.100184   2.894545  -3.901987
 C      1.128440   1.917986  -2.197323
 H      0.589721   1.199779  -2.834097
 C     -1.178308   1.678019   1.085764
 C     -1.600347   1.292406   2.376978
 H     -1.237368   0.355737   2.821078
 C     -2.489491   2.088578   3.119450
 H     -2.818668   1.792967   4.126852
 C     -2.954962   3.285037   2.558341
 C     -2.550211   3.706436   1.283192
 H     -2.925921   4.657492   0.876858
 C     -1.659270   2.900048   0.557244
 H     -1.326390   3.244570  -0.433852
 C     -0.363885  -2.653414  -1.059488
 H     -0.883686  -2.928066  -0.119281
 C      1.909631  -1.853235  -1.708364
 H      2.240767  -2.723202  -2.316701
 H      1.366391  -1.175020  -2.405717
 C      3.107978  -1.116448  -1.165834
 C      3.970154   0.239163   0.524677
 C      5.195748   0.373335  -0.137525
 C      5.376815  -0.282317  -1.367128
 C      4.315893  -1.038677  -1.883326
 C     -0.342646  -3.902899  -1.956575
 H     -1.371915  -4.283011  -2.113075
 H      0.098190  -3.701441  -2.954126
 H      0.241381  -4.711172  -1.471071
 C      1.602051   0.025046   2.479458
 C     -0.132861  -1.678930   1.977219
 H      1.456982  -2.961275  -0.010201
 H      4.412027  -1.572114  -2.841123
 H      5.994974   0.978042   0.315661
 H      3.800937   0.724472   1.496862
 H      6.330305  -0.208726  -1.912317
 O      2.364690  -2.644817   1.467546
 C      2.394047  -3.357232   2.668672
 C      3.282016  -2.728852   3.755055
 H      2.790587  -4.387855   2.461056
 H      1.370722  -3.522272   3.104857
 H      3.319420  -3.369868   4.663548
 H      4.321398  -2.599409   3.382827
 H      2.903324  -1.729965   4.055438
 F     -3.805860   4.058835   3.266799
 F      3.445354   4.712341  -2.585536
---------------------------------------
Mn23/x
Frequencies, energies and thermodynamic properties:
 Lowest Vibrational Mode (1/cm) =                         11.6478
 2nd Lowest Vibrational Mode (1/cm) =                     20.8281
 E(RB-P86) (a.u.) =                                     -4759.61776308
 Thermal correction to Enthalpy (a.u.) =                  0.697340
 Thermal correction to Gibbs Free Energy (a.u.) =         0.565717
 Total Entropy (cal/Kmol) =                               277.022
 E(RPBE1PBE) (a.u.) =                                   -4759.01132047
Optimised cartesian coordinates (Angstrom):
 Fe-2.810869  -1.828494  -1.203768
 Mn1.239502  -0.184954   0.995049
 P     -0.627650   0.704442   0.072353
 O      1.031212   1.719924   3.226615
 O     -0.141123  -2.249457   2.570461
 N      1.472353  -1.435043  -0.744771
 N      2.529620   0.975418  -0.150651
 C     -1.393771  -0.345690  -1.229154
 C     -0.832914  -1.606575  -1.711332
 C     -1.610315  -2.009917  -2.860475
 H     -1.461343  -2.924417  -3.447986
 C     -2.643091  -1.036108  -3.086450
 H     -3.410282  -1.081506  -3.870933
 C     -2.520835  -0.015119  -2.083457
 H     -3.165930   0.867328  -1.986217
 C     -3.103700  -2.595448   0.686422
 H     -2.408541  -2.500773   1.530631
 C     -3.100889  -3.652623  -0.289742
 H     -2.407967  -4.504287  -0.317482
 C     -4.151358  -3.386593  -1.238867
 H     -4.394886  -3.995536  -2.119771
 C     -4.806572  -2.165089  -0.846650
 H     -5.637337  -1.680148  -1.376513
 C     -4.157938  -1.674014   0.341047
 H     -4.407746  -0.750721   0.879198
```

```
 C     -0.318335   2.310830  -0.822028
 C     -0.005263   3.452002  -0.043548
 H     -0.020271   3.390383   1.056575
 C      0.319045   4.676015  -0.647612
 H      0.554143   5.568281  -0.048157
 C      0.344173   4.751673  -2.048700
 C      0.051892   3.641446  -2.850426
 H      0.078481   3.736538  -3.946338
 C     -0.279350   2.423290  -2.228927
 H     -0.524908   1.557599  -2.862681
 C     -2.061796   1.201604   1.154516
 C     -2.228558   0.637894   2.438367
 H     -1.497201  -0.083767   2.826441
 C     -3.327785   0.979236   3.244824
 H     -3.459794   0.544522   4.246856
 C     -4.265870   1.897764   2.755338
 C     -4.129709   2.484656   1.488797
 H     -4.879264   3.210193   1.138849
 C     -3.023395   2.134781   0.698344
 H     -2.908004   2.612785  -0.286516
 C      0.351793  -2.360458  -1.125289
 H      0.036020  -2.811083  -0.163236
 C      2.005854  -0.650557  -1.878922
 H      2.584118  -1.273625  -2.595038
 H      1.150841  -0.226956  -2.452876
 C      2.851588   0.491155  -1.379862
 C      3.211484   2.047686   0.319392
 C      4.232774   2.675732  -0.403259
 C      4.579552   2.168827  -1.666555
 C      3.877822   1.058319  -2.156902
 C      0.838246  -3.506989  -2.028297
 H      0.046468  -4.274690  -2.137917
 H      1.114835  -3.159089  -3.044595
 H      1.720951  -3.990656  -1.563791
 C      1.128913   0.945804   2.335691
 C      0.366164  -1.403230   1.916987
 H      3.375047  -2.274298   0.912445
 H      2.249103  -2.068238  -0.422005
 O      3.541983  -3.101954   0.273288
 C      4.838339  -2.978024  -0.294723
 H      4.830679  -3.470974  -1.295333
 H      5.097222  -1.904808  -0.474751
 H      4.115462   0.624615  -3.139899
 H      4.749295   3.544911   0.029744
 H      2.923218   2.411299   1.316017
 C      5.922361  -3.624011   0.572959
 H      5.690995  -4.695435   0.751436
 H      6.918595  -3.561646   0.084453
 H      5.994611  -3.122298   1.561456
 H      5.385372   2.628877  -2.258790
 O      3.023979  -1.043085   1.587569
 C      3.401547  -1.159209   2.940289
 C      4.273083   0.002807   3.435324
 H      3.982207  -2.110354   3.070744
 H      2.515211  -1.258916   3.617815
 H      4.613815  -0.175247   4.479004
 H      5.172530   0.118877   2.793799
 H      3.714427   0.961207   3.420535
 F     -5.322456   2.235178   3.525893
 F      0.657820   5.927364  -2.637779
----------------------------------------
Mn23/TS-i
Frequencies, energies and thermodynamic properties:
 Lowest Vibrational Mode (1/cm) =-745.0927
 2nd Lowest Vibrational Mode (1/cm) =                     23.7453
 E(RB-P86) (a.u.) =                                     -4605.82997642
 Thermal correction to Enthalpy (a.u.) =                  0.624940
 Thermal correction to Gibbs Free Energy (a.u.) =         0.505500
 Total Entropy (cal/Kmol) =                               251.383
 E(RPBE1PBE) (a.u.) =                                   -4605.21411142
Optimised cartesian coordinates (Angstrom):
 Fe-2.663842  -1.557703  -1.295132
 Mn1.257902  -0.423653   1.372079
 P     -0.288659   0.697291   0.142512
 O      1.100424   1.574443   3.542894
 O     -0.665005  -2.126894   2.811941
 N      1.529975  -1.763429  -0.226727
 N      2.879142   0.447972   0.427322
 C     -1.033222  -0.317227  -1.189550
 C     -0.619402  -1.678265  -1.522037
 C     -1.292301  -2.036116  -2.748794
 H     -1.214925  -3.002071  -3.263611
 C     -2.117967  -0.935766  -3.168291
 H     -2.770341  -0.918767  -4.051702
 C     -1.971000   0.122983  -2.208316
```

```
 H     -2.473427   1.098014  -2.246674
 C     -3.339104  -2.131051   0.566802
 H     -2.767826  -2.074248   1.502022
 C     -3.355175  -3.248043  -0.339588
 H     -2.801216  -4.188236  -0.215467
 C     -4.204356  -2.907441  -1.452429
 H     -4.407080  -3.539972  -2.327048
 C     -4.716764  -1.579094  -1.231038
 H     -5.379633  -1.022821  -1.907313
 C     -4.180144  -1.097354   0.015166
 H     -4.365137  -0.111328   0.460544
 C      0.422764   2.155553  -0.768660
 C      0.829182   3.279652  -0.009095
 H      0.666571   3.298848   1.080563
 C      1.431384   4.388664  -0.621825
 H      1.741869   5.268839  -0.039156
 C      1.639930   4.362296  -2.009312
 C      1.260749   3.263201  -2.790054
 H      1.437134   3.277452  -3.876022
 C      0.651914   2.161621  -2.161790
 H      0.341556   1.304988  -2.779113
 C     -1.741789   1.501289   0.984588
 C     -2.179874   1.062773   2.253449
 H     -1.647564   0.258052   2.778364
 C     -3.302806   1.637971   2.872341
 H     -3.645831   1.301795   3.862158
 C     -3.988812   2.664122   2.209200
 C     -3.578724   3.131067   0.951844
 H     -4.134070   3.945459   0.462984
 C     -2.452791   2.547812   0.349575
 H     -2.121398   2.930371  -0.627950
 C      0.367029  -2.537012  -0.732381
 H     -0.154154  -2.898362   0.177636
 C      2.314297  -1.151251  -1.304719
 H      2.888531  -1.898607  -1.900325
 H      1.652462  -0.632239  -2.044581
 C      3.256145  -0.118756  -0.753123
 C      3.654061   1.432484   0.951778
 C      4.826669   1.883134   0.337288
 C      5.228364   1.291579  -0.873000
 C      4.430576   0.277943  -1.419612
 C      0.800952  -3.792221  -1.516810
 H     -0.068952  -4.450232  -1.716804
 H      1.266464  -3.544436  -2.493111
 H      1.530844  -4.366287  -0.911221
 C      1.174625   0.772797   2.679046
 C      0.063934  -1.429120   2.197118
 H      2.505496  -1.034814   2.366271
 H      2.467795  -1.779174   1.936356
 H      2.293577  -2.610432   0.444289
 O      2.892914  -3.273897   1.243828
 C      4.283942  -3.279123   1.022559
 H      4.526850  -3.413212  -0.064956
 H      4.754803  -2.299434   1.313469
 H      4.706121  -0.215308  -2.363839
 H      5.414979   2.682192   0.811560
 H      3.317632   1.864173   1.905525
 C      4.962058  -4.397744   1.818176
 H      4.549498  -5.387423   1.527542
 H      6.059398  -4.413073   1.643730
 H      4.787427  -4.266441   2.907410
 H      6.150610   1.614125  -1.380161
 F      2.220903   5.426274  -2.606190
 F     -5.065615   3.225468   2.799288
---------------------------------------
Mn23/TS-ii_si
Frequencies, energies and thermodynamic properties:
 Lowest Vibrational Mode (1/cm) =-246.2637
 2nd Lowest Vibrational Mode (1/cm) =                     13.9729
 E(RB-P86) (a.u.) =                                     -4873.62953154
 Thermal correction to Enthalpy (a.u.) =                  0.697962
 Thermal correction to Gibbs Free Energy (a.u.) =         0.570593
 Total Entropy (cal/Kmol) =                               268.071
 E(RPBE1PBE) (a.u.) =                                   -4873.00035320
Optimised cartesian coordinates (Angstrom):
 Fe-2.730854  -2.017831  -1.492604
 Mn1.172755   0.211818   0.313484
 P     -1.059520   0.563228   0.343004
 O      1.654128   0.833455   3.149226
 O      1.135526  -2.646193   1.005959
 N      1.134173  -0.103344  -1.768070
 N      1.670246   2.088381  -0.347866
 C     -1.905064  -0.179001  -1.114359
 C     -1.217458  -0.838003  -2.225838
 C     -2.206335  -1.092060  -3.248804
```

H	-2.024659	-1.592364	-4.208127
C	-3.483565	-0.619658	-2.788964
H	-4.433329	-0.697822	-3.334812
C	-3.306507	-0.064051	-1.476421
H	-4.096532	0.377510	-0.855567
C	-2.100962	-3.437810	-0.139133
H	-1.215589	-3.356638	0.504559
C	-2.124031	-3.986231	-1.469430
H	-1.263666	-4.400824	-2.011871
C	-3.468211	-3.874843	-1.975305
H	-3.810553	-4.183221	-2.972220
C	-4.277827	-3.259626	-0.954849
H	-5.345888	-3.017809	-1.037931
C	-3.433341	-2.986993	0.178967
H	-3.743652	-2.504388	1.114654
C	-1.547026	2.357998	0.236834
C	-1.286688	3.177336	1.362876
H	-0.883391	2.731449	2.286681
C	-1.541840	4.556578	1.332309
H	-1.351095	5.196217	2.207154
C	-2.047568	5.123325	0.152252
C	-2.302985	4.347736	-0.985281
H	-2.700637	4.824772	-1.893693
C	-2.049973	2.964375	-0.935257
H	-2.266949	2.355096	-1.825921
C	-2.089290	0.021170	1.797267
C	-1.607717	-0.971044	2.678883
H	-0.613185	-1.411298	2.521653
C	-2.382776	-1.416888	3.763287
H	-2.011892	-2.188187	4.454777
C	-3.651089	-0.855333	3.962803
C	-4.158846	0.138942	3.113648
H	-5.154635	0.565753	3.306151
C	-3.369933	0.573862	2.036482
H	-3.761249	1.368852	1.383045
C	0.262156	-1.195993	-2.314407
H	0.454486	-2.055252	-1.641490
C	1.006182	1.177028	-2.496171
H	1.454698	1.135649	-3.512120
H	-0.075926	1.400957	-2.635176
C	1.622954	2.291278	-1.696874
C	2.139222	3.101125	0.430958
H	2.162072	2.911094	1.513496
C	2.583307	4.321399	-0.089594
H	2.951873	5.098575	0.595981
C	2.555545	4.521094	-1.480363
C	2.068323	3.484856	-2.290043
H	2.023113	3.592065	-3.384316
C	0.687299	-1.618324	-3.731250
H	0.161634	-2.546527	-4.031455
H	0.463589	-0.842574	-4.491667
H	1.775808	-1.828504	-3.750749
C	1.440218	0.587772	2.009127
C	1.096571	-1.497717	0.715041
H	2.138563	-0.413184	-1.905283
H	2.787526	-0.114191	0.172816
C	4.171422	-0.451593	-0.728860
C	4.564125	-1.622325	0.133624
C	4.960323	0.750611	-0.153834
C	5.258687	-1.165668	1.275995
C	4.380410	-2.992123	-0.115055
C	5.312970	0.352202	1.295942
H	5.884770	0.826282	-0.771713
C	5.774836	-2.092937	2.196183
C	4.900984	-3.918350	0.806262
H	3.846223	-3.322186	-1.020453
H	6.293400	0.742333	1.639335
C	5.591033	-3.469792	1.953712
H	6.323473	-1.755054	3.090860
H	4.777196	-4.999451	0.632596
H	5.997835	-4.206547	2.665246
O	3.794059	-0.542888	-1.931544
H	4.420283	1.710999	-0.253214
H	4.546734	0.741010	2.004747
H	2.907156	5.464357	-1.925246
F	-2.293595	6.452593	0.114339
F	-4.400551	-1.273858	5.006004

Mn23/TS-ii_re

Frequencies, energies and thermodynamic properties:

Lowest Vibrational Mode (1/cm) =-246.5739

2nd Lowest Vibrational Mode (1/cm) =	10.9749
E(RB-P86) (a.u.) =	-4873.63006481
Thermal correction to Enthalpy (a.u.) =	0.698097
Thermal correction to Gibbs Free Energy (a.u.) =	0.570545

Total Entropy (cal/Kmol) = 268.456
E(RPBE1PBE) (a.u.) = -4873.00193404
Optimised cartesian coordinates (Angstrom):

Fe-3.449873	-1.424446	-1.196761	
Mn0.989310	-0.424599	0.469062	
P	-0.950446	0.710940	0.221720
O	1.614328	0.583160	3.163081
O	-0.117197	-2.849463	1.708826
N	0.852175	-1.153447	-1.501454
N	2.160973	0.946689	-0.510259
C	-2.000164	0.025175	-1.126294
C	-1.599477	-1.066702	-2.014506
C	-2.603166	-1.165036	-3.049704
H	-2.613589	-1.896107	-3.867689
C	-3.615614	-0.172939	-2.811664
H	-4.521347	-0.020342	-3.413718
C	-3.254854	0.556593	-1.628184
H	-3.828283	1.380095	-1.183533
C	-4.471340	-1.678314	0.568780
H	-4.581379	-0.923096	1.357555
C	-3.404244	-2.642671	0.463952
H	-2.556229	-2.752012	1.152268
C	-3.625919	-3.421961	-0.725430
H	-2.981724	-4.230079	-1.097359
C	-4.828079	-2.941161	-1.356897
H	-5.257048	-3.313738	-2.296749
C	-5.351883	-1.865128	-0.554921
H	-6.250709	-1.274269	-0.776640
C	-0.729782	2.489422	-0.286814
C	-0.186463	3.383122	0.668690
H	0.018705	3.034403	1.693889
C	0.091786	4.718042	0.338793
H	0.504550	5.419407	1.079439
C	-0.161149	5.156513	-0.970128
C	-0.682973	4.297872	-1.945422
H	-0.869747	4.675227	-2.962091
C	-0.964862	2.964254	-1.595838
H	-1.389177	2.295873	-2.360500
C	-2.119826	0.914577	1.657063
C	-2.050810	0.036399	2.760486
H	-1.290482	-0.756689	2.785269
C	-2.945483	0.152906	3.838151
H	-2.895241	-0.527247	4.701577
C	-3.914205	1.165011	3.805015
C	-4.006734	2.061891	2.730501
H	-4.772857	2.851659	2.742311
C	-3.103343	1.932024	1.663366
H	-3.163960	2.649072	0.830113
C	-0.360932	-1.947143	-1.891241
H	-0.511530	-2.642297	-1.041657
C	1.216970	-0.108957	-2.482324
H	1.635666	-0.534219	-3.420053
H	0.295480	0.446043	-2.770780
C	2.187154	0.865134	-1.871740
C	2.964584	1.865779	0.088224
H	2.927509	1.901980	1.186232
C	3.803589	2.726458	-0.627573
H	4.425235	3.451561	-0.082184
C	3.838800	2.635548	-2.029002
C	3.021405	1.682473	-2.653621
H	3.013791	1.569801	-3.748278
C	-0.111378	-2.803436	-3.144447
H	-0.946969	-3.515066	-3.297434
H	-0.014358	-2.192948	-4.065323
H	0.815586	-3.397724	-3.014260
C	1.352681	0.191803	2.075389
C	0.273509	-1.852267	1.195835
H	1.665422	-1.834316	-1.481025
H	2.383303	-1.314778	0.528028
C	3.481852	-2.409269	-0.124453
C	4.674556	-1.590150	0.291105
C	3.293746	-3.445180	1.011758
C	4.996210	-1.861127	1.640562
C	5.459335	-0.727562	-0.491837
C	3.993418	-2.829825	2.243049
C	6.118312	-1.252538	2.226205
C	6.584158	-0.122101	0.097138
H	5.196216	-0.547552	-1.546646
C	6.908144	-0.382335	1.446572
H	6.387666	-1.456482	3.275712
H	7.222101	0.552531	-0.496208
H	7.795462	0.094476	1.893680
O	3.126700	-2.595004	-1.323330
H	4.468279	-3.580380	2.908226
H	3.260811	-2.267179	2.865568

```
 H      2.237369  -3.729094   1.171692
 H      3.838913  -4.356409   0.673100
 H      4.493340   3.290761  -2.623719
 F     -4.774695   1.287907   4.839201
 F      0.107142   6.441226  -1.296263
---------------------------------------
Mn23/TS-iii
Frequencies, energies and thermodynamic properties:
 Lowest Vibrational Mode (1/cm) =-631.4994
 2nd Lowest Vibrational Mode (1/cm) =                     23.7918
 E(RB-P86) (a.u.) =                                     -4450.88527785
 Thermal correction to Enthalpy (a.u.) =                  0.542934
 Thermal correction to Gibbs Free Energy (a.u.) =         0.435511
 Total Entropy (cal/Kmol) =                               226.091
 E(RPBE1PBE) (a.u.) =                                   -4450.26405106
Optimised cartesian coordinates (Angstrom):
 Fe-2.831466  -1.083498  -1.034025
 Mn1.010359  -0.721762   1.881252
 P      0.191884   0.446723   0.114893
 O      1.703735   1.633975   3.528319
 O     -1.538949  -0.897672   3.339350
 N      0.812606  -2.512251   0.821870
 N      2.916368  -0.899605   1.077707
 C     -0.831970  -0.621067  -0.972870
 C     -1.028546  -2.061215  -0.798422
 C     -1.721186  -2.533555  -1.974799
 H     -2.040862  -3.567616  -2.156054
 C     -1.966521  -1.424314  -2.856872
 H     -2.496952  -1.467385  -3.817760
 C     -1.429206  -0.242274  -2.242419
 H     -1.458738   0.769750  -2.666278
 C     -3.790955  -0.625789   0.733735
 H     -3.302045  -0.448120   1.699768
 C     -4.230159  -1.903212   0.238995
 H     -4.139074  -2.864255   0.762798
 C     -4.787998  -1.705147  -1.074697
 H     -5.192678  -2.488686  -1.729335
 C     -4.696127  -0.301891  -1.390135
 H     -5.019526   0.170583  -2.327418
 C     -4.076989   0.365217  -0.274147
 H     -3.847249   1.436530  -0.207459
 C      1.499032   1.109430  -1.033100
 C      2.289870   2.192835  -0.579026
 H      2.082721   2.655086   0.399788
 C      3.337142   2.703795  -1.360476
 H      3.950265   3.550766  -1.017754
 C      3.601733   2.110716  -2.604436
 C      2.849108   1.029256  -3.079342
 H      3.084225   0.588785  -4.059919
 C      1.798769   0.532597  -2.286105
 H      1.201842  -0.311497  -2.663704
 C     -0.831425   1.981305   0.372632
 C     -1.452874   2.229124   1.616149
 H     -1.318381   1.528662   2.451729
 C     -2.255178   3.366152   1.813618
 H     -2.739281   3.564868   2.781445
 C     -2.429846   4.262425   0.750919
 C     -1.820631   4.054873  -0.495440
 H     -1.969915   4.784846  -1.305135
 C     -1.020364   2.915367  -0.674134
 H     -0.526386   2.765881  -1.646517
 C     -0.526466  -2.908438   0.381439
 H     -1.200021  -2.714278   1.242209
 C      1.849373  -2.683733  -0.175954
 H      2.114914  -3.755448  -0.358169
 H      1.547432  -2.291066  -1.189251
 C      3.095982  -1.936402   0.211583
 C      3.995435  -0.153407   1.429423
 C      5.282767  -0.403893   0.944147
 C      5.476186  -1.478999   0.058765
 C      4.366860  -2.252717  -0.305133
 C     -0.629656  -4.418855   0.076025
 H     -1.684073  -4.722398  -0.087946
 H     -0.053998  -4.707121  -0.828083
 H     -0.236644  -4.997746   0.936340
 C      1.427066   0.692524   2.870059
 C     -0.548657  -0.814594   2.697019
 H      1.312790  -2.314351   2.322913
 H      1.609664  -1.816545   3.043218
 H      3.811967   0.670289   2.134316
 H      6.118014   0.235032   1.266306
 H      4.470254  -3.107094  -0.991041
 H      6.476825  -1.711514  -0.337016
 F     -3.194879   5.360470   0.933412
 F      4.610133   2.596653  -3.362573
```

```
--------------------------------------
Mn24/i
Frequencies, energies and thermodynamic properties:
 Lowest Vibrational Mode (1/cm) =                        21.3276
 2nd Lowest Vibrational Mode (1/cm) =                    23.9322
 E(RB-P86) (a.u.) =                                    -5170.40232464
 Thermal correction to Enthalpy (a.u.) =                 0.525798
 Thermal correction to Gibbs Free Energy (a.u.) =        0.415529
 Total Entropy (cal/Kmol) =                              232.081
 E(RPBE1PBE) (a.u.) =                                  -5169.65823047
Optimised cartesian coordinates (Angstrom):
 Fe-2.644460  -1.505771  -1.287721
 Mn0.856938  -0.876683   1.869805
 P      0.076764   0.288054   0.195253
 O      1.289103   1.349889   3.785581
 O     -1.714009  -1.346446   3.214259
 N      0.890508  -2.508028   0.901303
 N      2.871183  -0.943318   1.427265
 C     -0.726252  -0.829082  -1.028618
 C     -0.773270  -2.285165  -0.913897
 C     -1.300026  -2.794479  -2.157354
 H     -1.483539  -3.850887  -2.391623
 C     -1.584052  -1.686794  -3.031138
 H     -2.013860  -1.753754  -4.039802
 C     -1.240361  -0.474309  -2.339885
 H     -1.341461   0.541985  -2.742066
 C     -3.782361  -1.235281   0.408587
 H     -3.389554  -1.057050   1.417938
 C     -4.045985  -2.524344  -0.173743
 H     -3.895942  -3.496277   0.315330
 C     -4.516851  -2.318989  -1.519944
 H     -4.784142  -3.106144  -2.237651
 C     -4.547577  -0.900136  -1.768711
 H     -4.843867  -0.416515  -2.709229
 C     -4.091381  -0.230048  -0.578475
 H     -3.981749   0.854495  -0.449696
 C      1.440259   1.080863  -0.789997
 C      2.062835   2.240813  -0.270424
 H      1.683660   2.703827   0.655051
 C      3.162202   2.825000  -0.916702
 H      3.634293   3.732825  -0.512989
 C      3.660270   2.231509  -2.092386
 C      3.072388   1.070318  -2.620810
 H      3.472642   0.617040  -3.539697
 C      1.965314   0.501298  -1.965599
 H      1.502078  -0.400888  -2.393267
 C     -1.096080   1.716178   0.399871
 C     -1.832477   1.867068   1.594129
 H     -1.700079   1.153165   2.419470
 C     -2.744840   2.923875   1.755022
 H     -3.313752   3.035361   2.689681
 C     -2.919450   3.844354   0.707160
 C     -2.191686   3.723783  -0.490827
 H     -2.331319   4.456535  -1.299182
 C     -1.283251   2.662986  -0.634328
 H     -0.704511   2.589243  -1.568114
 C     -0.329185  -3.094014   0.315974
 H     -1.116618  -2.972685   1.086334
 C      2.103326  -2.855853   0.185426
 H      2.383739  -3.929882   0.312748
 H      1.991607  -2.723182  -0.924389
 C      3.240752  -1.996804   0.643381
 C      3.845614  -0.097124   1.865639
 H      3.515251   0.743298   2.492686
 C      5.195781  -0.273848   1.558712
 H      5.936906   0.440493   1.946540
 C      5.579237  -1.369045   0.759688
 C      4.583720  -2.239234   0.299907
 H      4.831740  -3.109018  -0.327363
 C     -0.231237  -4.601306   0.015991
 H     -1.231446  -5.006770  -0.237952
 H      0.446615  -4.828737  -0.832011
 H      0.133459  -5.143423   0.912320
 C      1.109360   0.465203   3.018123
 C     -0.709026  -1.145225   2.616215
 H      6.636183  -1.537868   0.502866
 Cl-4.052780   5.171132   0.899397
 Cl5.038537   2.954163  -2.907853
--------------------------------------
Mn24/ii
Frequencies, energies and thermodynamic properties:
 Lowest Vibrational Mode (1/cm) =                        15.2876
 2nd Lowest Vibrational Mode (1/cm) =                    19.0248
 E(RB-P86) (a.u.) =                                    -5325.33695813
 Thermal correction to Enthalpy (a.u.) =                 0.610515
```

```
 Thermal correction to Gibbs Free Energy (a.u.) =         0.484542
 Total Entropy (cal/Kmol) =                               265.131
 E(RPBE1PBE) (a.u.) =                                    -5324.60154792
Optimised cartesian coordinates (Angstrom):
 Fe-2.326348  -1.805388  -1.693421
 Mn1.186930  -0.597292   1.293376
 P     -0.311467   0.511326   0.148853
 O      1.022297   0.990429   3.792417
 O     -0.688475  -2.482817   2.544641
 N      1.686396  -1.732119  -0.178857
 N      2.920607   0.362311   0.717175
 C     -0.826167  -0.456656  -1.327982
 C     -0.265808  -1.752085  -1.708615
 C     -0.777657  -2.068968  -3.020065
 H     -0.566490  -2.985376  -3.585836
 C     -1.644501  -1.004931  -3.451835
 H     -2.200916  -0.969353  -4.398181
 C     -1.685309  -0.013216  -2.412424
 H     -2.259780   0.921576  -2.442230
 C     -3.121203  -2.577770   0.045134
 H     -2.640333  -2.560849   1.031776
 C     -2.973491  -3.612526  -0.943444
 H     -2.364676  -4.520880  -0.840931
 C     -3.742684  -3.236732  -2.102257
 H     -3.819476  -3.805694  -3.038480
 C     -4.369551  -1.968853  -1.828013
 H     -5.008877  -1.402773  -2.518657
 C     -3.983808  -1.559640  -0.502468
 H     -4.280743  -0.629052  -0.001740
 C      0.403175   2.062598  -0.585098
 C      0.618629   3.173382   0.265215
 H      0.294654   3.135286   1.317925
 C      1.238977   4.338657  -0.208671
 H      1.394147   5.202205   0.454696
 C      1.666501   4.390564  -1.549263
 C      1.478977   3.296976  -2.410683
 H      1.817785   3.351817  -3.455715
 C      0.847929   2.138349  -1.922904
 H      0.691188   1.291752  -2.608459
 C     -1.888457   1.156827   0.890389
 C     -2.381280   0.629341   2.103060
 H     -1.824964  -0.155234   2.634930
 C     -3.588936   1.089281   2.655433
 H     -3.965289   0.673998   3.601724
 C     -4.311347   2.092778   1.986980
 C     -3.838900   2.643040   0.781431
 H     -4.409130   3.434368   0.273062
 C     -2.629683   2.173966   0.244371
 H     -2.259115   2.625690  -0.688804
 C      0.692435  -2.594142  -0.856413
 H      0.093873  -3.045951  -0.040193
 C      2.716643  -1.230922  -1.078330
 H      3.418626  -2.028689  -1.420877
 H      2.273405  -0.815957  -2.022289
 C      3.495347  -0.137633  -0.414263
 C      3.555834   1.375840   1.369049
 C      4.767775   1.913313   0.931540
 C      5.366192   1.392657  -0.232187
 C      4.718502   0.352365  -0.908533
 C      1.316281  -3.761960  -1.643382
 H      0.531578  -4.481364  -1.952998
 H      1.847393  -3.430048  -2.558917
 H      2.032500  -4.295518  -0.986123
 C      1.083401   0.358799   2.793324
 C      0.023276  -1.713711   1.991071
 H      2.556691  -2.978483   0.943408
 O      2.973052  -3.744520   1.433274
 C      4.372585  -3.511543   1.525933
 H      4.590828  -2.504525   1.962309
 H      4.858920  -3.527752   0.516778
 H      5.147516  -0.088565  -1.820990
 H      5.235958   2.729022   1.501724
 H      3.065984   1.754984   2.277240
 C      5.004558  -4.587103   2.402566
 H      6.101035  -4.438478   2.485989
 H      4.571231  -4.564536   3.424618
 H      4.822579  -5.596694   1.977384
 H      6.322904   1.792527  -0.601515
 Cl-5.818127   2.676710   2.670921
 Cl2.446442   5.844285  -2.151025
---------------------------------------
Mn24/iii
Frequencies, energies and thermodynamic properties:
 Lowest Vibrational Mode (1/cm) =                         18.5177
 2nd Lowest Vibrational Mode (1/cm) =                     23.9144
```

```
 E(RB-P86) (a.u.) =                                       -5326.50943381
 Thermal correction to Enthalpy (a.u.) =                   0.626621
 Thermal correction to Gibbs Free Energy (a.u.) =          0.502185
 Total Entropy (cal/Kmol) =                                261.898
 E(RPBE1PBE) (a.u.) =                                     -5325.77091629
Optimised cartesian coordinates (Angstrom):
 Fe-2.292790  -1.893744  -1.636751
 Mn1.201289  -0.577659   1.503251
 P     -0.280611   0.465445   0.138242
 O      0.599668   1.326929   3.688393
 O     -0.725516  -2.495032   2.635999
 N      1.760784  -1.809327  -0.088331
 N      2.840037   0.466499   0.788704
 C     -0.788434  -0.539035  -1.302292
 C     -0.229084  -1.847484  -1.628702
 C     -0.729879  -2.202232  -2.935798
 H     -0.516872  -3.136382  -3.470690
 C     -1.591312  -1.152457  -3.410770
 H     -2.139713  -1.147867  -4.362418
 C     -1.641029  -0.127621  -2.404896
 H     -2.213681   0.806625  -2.467350
 C     -3.130106  -2.599838   0.111283
 H     -2.678809  -2.540299   1.109742
 C     -2.947752  -3.673351  -0.828484
 H     -2.335620  -4.571605  -0.671655
 C     -3.686891  -3.351243  -2.022538
 H     -3.733435  -3.958460  -2.936470
 C     -4.329770  -2.077921  -1.818287
 H     -4.953600  -1.546020  -2.549114
 C     -3.983716  -1.611390  -0.500896
 H     -4.301335  -0.663997  -0.046612
 C      0.406919   2.017389  -0.625968
 C      0.633405   3.132233   0.216069
 H      0.355413   3.087906   1.281422
 C      1.201490   4.313970  -0.282535
 H      1.366823   5.178908   0.376471
 C      1.561258   4.380471  -1.641608
 C      1.357609   3.285174  -2.497181
 H      1.641634   3.351239  -3.557730
 C      0.781473   2.109440  -1.983285
 H      0.614017   1.261440  -2.664591
 C     -1.879329   1.119964   0.837947
 C     -2.425339   0.588999   2.026206
 H     -1.898701  -0.201421   2.578029
 C     -3.652021   1.051091   2.532613
 H     -4.066756   0.631652   3.460843
 C     -4.343421   2.060868   1.841849
 C     -3.820630   2.613685   0.658856
 H     -4.366697   3.409094   0.130736
 C     -2.592302   2.142602   0.168912
 H     -2.184863   2.597512  -0.747035
 C      0.739799  -2.647986  -0.753407
 H      0.147038  -3.099543   0.069079
 C      2.598309  -1.093417  -1.046985
 H      3.303026  -1.763600  -1.596879
 H      1.998303  -0.591714  -1.855836
 C      3.383586  -0.010679  -0.365860
 C      3.468333   1.483351   1.434042
 C      4.654327   2.058448   0.967495
 C      5.226507   1.563538  -0.217888
 C      4.580861   0.516330  -0.887007
 C      1.363268  -3.825941  -1.534621
 H      0.580536  -4.539922  -1.863421
 H      1.912282  -3.491707  -2.439472
 H      2.067998  -4.368866  -0.873268
 C      0.856235   0.562639   2.826126
 C      0.005680  -1.712315   2.138699
 H      2.369890  -1.078784   2.677789
 H      2.393779  -1.729071   2.162302
 H      2.693118  -2.869305   0.707051
 O      3.215529  -3.530566   1.327534
 C      4.608494  -3.358672   1.139615
 H      4.901329  -3.521619   0.069950
 H      4.934250  -2.315273   1.390972
 H      4.993742   0.095493  -1.816101
 H      5.120109   2.877926   1.534023
 H      2.998734   1.838717   2.362882
 C      5.375019  -4.343554   2.019180
 H      5.095358  -5.388961   1.769238
 H      6.471389  -4.234945   1.882492
 H      5.141382  -4.177508   3.092208
 H      6.163653   1.986715  -0.611067
 Cl2.274360   5.854870  -2.275414
 Cl-5.874172   2.648048   2.468224
---------------------------------------
```

```
Mn24/iv
Frequencies, energies and thermodynamic properties:
 Lowest Vibrational Mode (1/cm) =                          17.7597
 2nd Lowest Vibrational Mode (1/cm) =                      21.7074
 E(RB-P86) (a.u.) =                                      -5326.53848998
 Thermal correction to Enthalpy (a.u.) =                   0.631346
 Thermal correction to Gibbs Free Energy (a.u.) =          0.506735
 Total Entropy (cal/Kmol) =                                262.267
 E(RPBE1PBE) (a.u.) =                                    -5325.79814670
Optimised cartesian coordinates (Angstrom):
 Fe-2.435988  -1.681170  -1.716734
 Mn1.258546  -0.704020   1.311193
 P     -0.266341   0.492772   0.122489
 O      1.014327   0.932465   3.741667
 O     -0.548393  -2.701886   2.495572
 N      1.692030  -1.875658  -0.440585
 N      2.910251   0.285468   0.586942
 C     -0.887807  -0.380931  -1.372876
 C     -0.387240  -1.676738  -1.834367
 C     -0.970505  -1.921085  -3.134261
 H     -0.815779  -2.815165  -3.750933
 C     -1.827040  -0.818139  -3.474299
 H     -2.430118  -0.729767  -4.387835
 C     -1.785756   0.127546  -2.394234
 H     -2.335665   1.076492  -2.351966
 C     -3.157085  -2.502616   0.029488
 H     -2.625136  -2.531470   0.989478
 C     -3.081271  -3.502070  -1.003222
 H     -2.487488  -4.425288  -0.966103
 C     -3.901688  -3.068923  -2.105313
 H     -4.037328  -3.600239  -3.056827
 C     -4.488214  -1.801611  -1.751933
 H     -5.150320  -1.197984  -2.387044
 C     -4.026755  -1.449714  -0.434422
 H     -4.277817  -0.533008   0.114289
 C      0.399447   2.087357  -0.577216
 C      0.731024   3.116636   0.337275
 H      0.517684   2.989059   1.411144
 C      1.323821   4.312257  -0.095461
 H      1.567930   5.109963   0.621582
 C      1.608240   4.479775  -1.463759
 C      1.306057   3.469960  -2.391901
 H      1.533326   3.613211  -3.458662
 C      0.703690   2.280561  -1.942124
 H      0.457628   1.503188  -2.681852
 C     -1.815242   1.119000   0.951692
 C     -2.281511   0.506371   2.134336
 H     -1.721314  -0.328499   2.578814
 C     -3.461782   0.940743   2.761773
 H     -3.817181   0.457353   3.683657
 C     -4.184036   2.007579   2.199857
 C     -3.738727   2.643658   1.026867
 H     -4.309040   3.482828   0.601873
 C     -2.556574   2.196866   0.414519
 H     -2.206501   2.713433  -0.492680
 C      0.562220  -2.615418  -1.099831
 H      0.012400  -3.073564  -0.253570
 C      2.523586  -1.090450  -1.377557
 H      3.139742  -1.729856  -2.045970
 H      1.844576  -0.505056  -2.037719
 C      3.396390  -0.124974  -0.620482
 C      3.619390   1.220794   1.272641
 C      4.823093   1.760451   0.805042
 C      5.336409   1.319860  -0.426027
 C      4.605964   0.360870  -1.144229
 C      1.073203  -3.760576  -1.990598
 H      0.230724  -4.402657  -2.316136
 H      1.590472  -3.393719  -2.900619
 H      1.775502  -4.398872  -1.416961
 C      1.104422   0.278693   2.756492
 C      0.136284  -1.879139   1.984047
 H      2.372173  -1.561398   2.077731
 H      2.801367  -2.931411   1.761362
 H      2.298642  -2.608534  -0.013202
 O      3.144499  -3.739887   1.268063
 C      4.570255  -3.752495   1.395394
 H      4.948432  -4.466802   0.632033
 H      5.002673  -2.754598   1.142228
 H      4.963122  -0.015332  -2.114894
 H      5.349060   2.512607   1.411418
 H      3.202028   1.533492   2.240594
 C      5.029556  -4.183033   2.787990
 H      4.620390  -5.182196   3.045252
 H      6.137701  -4.237451   2.839058
 H      4.689320  -3.462273   3.561628
```

```
 H      6.285958   1.713823  -0.819038
 Cl-5.657687   2.562387   2.978272
 Cl2.353549   5.972814  -2.016445
---------------------------------------
Mn24/v
Frequencies, energies and thermodynamic properties:
 Lowest Vibrational Mode (1/cm) =                        22.1250
 2nd Lowest Vibrational Mode (1/cm) =                    25.3504
 E(RB-P86) (a.u.) =                                    -5171.60018321
 Thermal correction to Enthalpy (a.u.) =                 0.546793
 Thermal correction to Gibbs Free Energy (a.u.) =        0.436293
 Total Entropy (cal/Kmol) =                              232.565
 E(RPBE1PBE) (a.u.) =                                  -5170.85572822
Optimised cartesian coordinates (Angstrom):
 Fe-2.668603  -1.393349  -1.337136
 Mn0.857184  -0.915034   1.978819
 P      0.121244   0.259193   0.180380
 O      1.238920   1.412645   3.732118
 O     -1.700834  -1.486789   3.313180
 N      0.833280  -2.719234   0.810083
 N      2.823949  -1.023335   1.403572
 C     -0.727638  -0.784994  -1.077253
 C     -0.832550  -2.243876  -1.003063
 C     -1.381772  -2.697401  -2.261562
 H     -1.606029  -3.737469  -2.528926
 C     -1.630566  -1.555724  -3.098197
 H     -2.072162  -1.579049  -4.103439
 C     -1.236769  -0.379362  -2.374766
 H     -1.305862   0.652415  -2.742681
 C     -3.782472  -1.146972   0.376955
 H     -3.375360  -1.023854   1.389151
 C     -4.100445  -2.401199  -0.253523
 H     -3.986003  -3.397132   0.195264
 C     -4.574119  -2.124352  -1.585517
 H     -4.876324  -2.871564  -2.331466
 C     -4.552859  -0.696907  -1.777318
 H     -4.836970  -0.165217  -2.695313
 C     -4.061722  -0.092494  -0.566501
 H     -3.908310   0.980858  -0.396711
 C      1.471907   1.078351  -0.811124
 C      2.173547   2.145995  -0.199466
 H      1.857551   2.516356   0.789542
 C      3.268488   2.755385  -0.830282
 H      3.799470   3.591174  -0.351037
 C      3.685906   2.280070  -2.087802
 C      3.021831   1.210908  -2.710969
 H      3.358445   0.848100  -3.693468
 C      1.919479   0.617541  -2.068339
 H      1.398666  -0.209922  -2.574508
 C     -1.035697   1.703898   0.413813
 C     -1.802440   1.804814   1.594182
 H     -1.701515   1.041893   2.379573
 C     -2.702750   2.866475   1.788448
 H     -3.296171   2.937069   2.711916
 C     -2.833452   3.845289   0.788093
 C     -2.074897   3.776544  -0.394550
 H     -2.180828   4.554027  -1.165511
 C     -1.179884   2.708581  -0.570778
 H     -0.577145   2.673840  -1.491706
 C     -0.458016  -3.144770   0.168206
 H     -1.210778  -3.007903   0.970087
 C      2.001240  -2.747816  -0.097569
 H      2.301447  -3.779048  -0.382484
 H      1.708694  -2.229157  -1.037926
 C      3.159599  -2.008428   0.519005
 C      3.831759  -0.274123   1.926067
 H      3.534023   0.513067   2.633522
 C      5.180055  -0.480998   1.612824
 H      5.945113   0.160524   2.074820
 C      5.525867  -1.510484   0.721910
 C      4.491642  -2.283325   0.170955
 H      4.706970  -3.100880  -0.533708
 C     -0.446947  -4.634454  -0.212559
 H     -1.463100  -4.964569  -0.505803
 H      0.236326  -4.851702  -1.058543
 H     -0.138715  -5.252193   0.655971
 C      1.076245   0.482819   3.012294
 C     -0.700846  -1.232003   2.726522
 H      1.007531  -3.379362   1.580319
 H      1.350295  -1.865572   3.147682
 H      6.576741  -1.708740   0.461979
 Cl-3.951432   5.180264   1.021706
 Cl5.059302   3.033751  -2.886060
---------------------------------------
Mn24/vi_R
```

```
Frequencies, energies and thermodynamic properties:
 Lowest Vibrational Mode (1/cm) =                       13.5874
 2nd Lowest Vibrational Mode (1/cm) =                   18.5869
 E(RB-P86) (a.u.) =                                    -5594.31196265
 Thermal correction to Enthalpy (a.u.) =                0.699217
 Thermal correction to Gibbs Free Energy (a.u.) =       0.566096
 Total Entropy (cal/Kmol) =                             280.176
 E(RPBE1PBE) (a.u.) =                                  -5593.56290063
Optimised cartesian coordinates (Angstrom):
 Fe-2.237960  -2.280550  -1.982212
 Mn1.123992   0.230918   0.319068
 P     -1.068074   0.317125   0.180676
 O      1.265792   0.734899   3.235739
 O      1.270964  -2.643550   0.928729
 N      1.361712   0.052122  -1.621032
 N      1.559247   2.191923  -0.143816
 C     -1.654461  -0.402854  -1.403107
 C     -0.778912  -0.905443  -2.460023
 C     -1.613139  -1.183393  -3.604989
 H     -1.270797  -1.593230  -4.563649
 C     -2.978421  -0.873750  -3.274525
 H     -3.847298  -1.003945  -3.933631
 C     -3.012924  -0.400596  -1.918238
 H     -3.911166  -0.083239  -1.373232
 C     -1.627639  -3.728879  -0.648164
 H     -0.839178  -3.612727   0.106276
 C     -1.435122  -4.178415  -2.001160
 H     -0.477798  -4.467388  -2.455323
 C     -2.713945  -4.159340  -2.664322
 H     -2.901369  -4.425870  -3.713097
 C     -3.698879  -3.700144  -1.718355
 H     -4.768812  -3.556434  -1.920214
 C     -3.027881  -3.430973  -0.473095
 H     -3.496004  -3.050329   0.443904
 C     -1.687000   2.069175   0.115482
 C     -1.674943   2.828877   1.309544
 H     -1.371410   2.360557   2.259821
 C     -2.050699   4.180086   1.312067
 H     -2.048476   4.762071   2.245398
 C     -2.429761   4.787824   0.099906
 C     -2.436667   4.060966  -1.102118
 H     -2.735735   4.548531  -2.041655
 C     -2.064740   2.704504  -1.087059
 H     -2.087757   2.138715  -2.030814
 C     -2.180179  -0.399188   1.487880
 C     -1.702071  -1.373832   2.389358
 H     -0.655763  -1.706015   2.345903
 C     -2.546599  -1.942023   3.358270
 H     -2.164587  -2.699642   4.058007
 C     -3.887651  -1.527216   3.428025
 C     -4.389014  -0.550791   2.548046
 H     -5.438051  -0.227878   2.618929
 C     -3.531271   0.008561   1.587904
 H     -3.929149   0.786693   0.918353
 C      0.741638  -1.075197  -2.357857
 H      0.936916  -1.970096  -1.733456
 C      1.338286   1.303907  -2.376209
 H      2.043251   1.298983  -3.241117
 H      0.328140   1.494558  -2.824180
 C      1.669008   2.455734  -1.477098
 C      1.811454   3.199413   0.736906
 H      1.712990   2.952061   1.803462
 C      2.184531   4.482607   0.331250
 H      2.378575   5.255415   1.089283
 C      2.309098   4.754032  -1.044606
 C      2.049921   3.723459  -1.955594
 H      2.136272   3.886573  -3.040401
 C      1.386436  -1.345473  -3.731204
 H      1.011524  -2.301040  -4.150136
 H      1.169704  -0.547833  -4.470964
 H      2.485959  -1.436248  -3.621772
 C      1.211534   0.535551   2.070778
 C      1.157024  -1.496499   0.656914
 H      2.975113  -0.256401  -1.601164
 H      3.294419  -0.015993   0.414384
 C      4.282570  -0.150097  -0.163536
 C      5.035969  -1.276183   0.531398
 C      5.172053   1.088913   0.141147
 C      5.946215  -0.752027   1.475641
 C      4.919879  -2.659787   0.330328
 C      5.878561   0.766320   1.480684
 H      5.919111   1.153377  -0.680298
 C      6.746365  -1.618359   2.239595
 C      5.724542  -3.528089   1.094507
 H      4.215392  -3.052920  -0.419922
```

```
 H      6.874035   1.246678   1.585396
 C      6.629875  -3.010091   2.043587
 H      7.464999  -1.219794   2.975017
 H      5.651948  -4.617928   0.946985
 H      7.258186  -3.698981   2.631282
 O      3.997282  -0.362734  -1.511247
 H      4.600942   2.038942   0.153568
 H      5.266665   1.117799   2.343336
 H      2.606382   5.753397  -1.397484
 Cl-4.949458  -2.227517   4.637040
 Cl-2.899729   6.479817   0.092623
---------------------------------------
Mn24/vi_S
Frequencies, energies and thermodynamic properties:
 Lowest Vibrational Mode (1/cm) =                        14.4195
 2nd Lowest Vibrational Mode (1/cm) =                    17.0665
 E(RB-P86) (a.u.) =                                    -5594.31221402
 Thermal correction to Enthalpy (a.u.) =                 0.699228
 Thermal correction to Gibbs Free Energy (a.u.) =        0.565920
 Total Entropy (cal/Kmol) =                              280.569
 E(RPBE1PBE) (a.u.) =                                  -5593.56340838
Optimised cartesian coordinates (Angstrom):
 Fe-3.067661  -1.783951  -1.712813
 Mn0.978857  -0.400624   0.436214
 P     -1.008957   0.485794   0.131984
 O      1.269738   0.470173   3.253953
 O      0.008062  -2.979891   1.463424
 N      1.148235  -0.962085  -1.435663
 N      2.145595   1.145392  -0.271167
 C     -1.810323  -0.202526  -1.369818
 C     -1.185502  -1.163087  -2.277173
 C     -2.049318  -1.283604  -3.427472
 H     -1.881275  -1.941719  -4.289362
 C     -3.192145  -0.429284  -3.245012
 H     -4.036511  -0.324070  -3.939494
 C     -3.056151   0.233052  -1.977152
 H     -3.767669   0.951281  -1.550049
 C     -3.077762  -3.122291  -0.144572
 H     -2.313046  -3.196745   0.639084
 C     -3.062233  -3.821355  -1.401816
 H     -2.287408  -4.521717  -1.741453
 C     -4.227823  -3.422691  -2.148823
 H     -4.494442  -3.761652  -3.158838
 C     -4.966343  -2.477767  -1.350342
 H     -5.895236  -1.971482  -1.645266
 C     -4.254634  -2.289169  -0.112905
 H     -4.546875  -1.617471   0.704582
 C     -0.905304   2.307012  -0.227223
 C     -0.616079   3.185551   0.844193
 H     -0.527464   2.795421   1.871122
 C     -0.442711   4.560181   0.626928
 H     -0.227566   5.238798   1.465378
 C     -0.544274   5.064665  -0.683616
 C     -0.816190   4.213018  -1.767094
 H     -0.893348   4.621187  -2.785584
 C     -0.995156   2.837760  -1.532155
 H     -1.222753   2.180312  -2.384979
 C     -2.324687   0.461735   1.446290
 C     -2.263980  -0.459177   2.513683
 H     -1.421700  -1.159668   2.596533
 C     -3.272789  -0.504489   3.490988
 H     -3.215729  -1.224469   4.320432
 C     -4.356426   0.385940   3.400510
 C     -4.438197   1.320760   2.352320
 H     -5.286564   2.018697   2.297838
 C     -3.420527   1.354604   1.386104
 H     -3.484915   2.104006   0.582116
 C      0.151171  -1.876415  -2.042248
 H     -0.018835  -2.664514  -1.281244
 C      1.609336   0.069999  -2.363285
 H      2.262268  -0.336576  -3.171472
 H      0.751142   0.554688  -2.898181
 C      2.351918   1.137166  -1.618902
 C      2.761786   2.105926   0.471161
 H      2.575182   2.081038   1.554206
 C      3.594928   3.076278  -0.089550
 H      4.066989   3.826661   0.561232
 C      3.815391   3.065329  -1.479807
 C      3.187365   2.078597  -2.248900
 H      3.331250   2.027785  -3.338676
 C      0.658076  -2.589521  -3.310744
 H     -0.050257  -3.387356  -3.612222
 H      0.773343  -1.900831  -4.172618
 H      1.637235  -3.068853  -3.108648
 C      1.158042   0.123903   2.128260
```

C   0.341680 -1.932156   1.021346
H   2.539446 -1.832543 -1.271619
H   2.927290 -1.385032   0.699439
C   3.725440 -2.082241   0.247808
C   3.725068 -3.386871   1.095173
C   4.628535 -3.079644   2.314673
O   3.470022 -2.246222 -1.113003
H   4.469829   3.813404 -1.952780
C   5.077061 -1.470022   0.584726
C   5.590869 -2.035046   1.773364
C   5.792483 -0.487806 -0.116830
C   6.830935 -1.608155   2.277454
C   7.037879 -0.062864   0.387484
H   5.387502 -0.070793 -1.053155
C   7.551340 -0.617833   1.577761
H   7.244850 -2.047065   3.200580
H   7.618228   0.702260 -0.153335
H   8.529177 -0.281755   1.959325
H   5.142492 -3.974285   2.724686
H   4.029595 -2.649961   3.150657
H   4.190975 -4.175431   0.464091
H   2.704860 -3.724828   1.364185
Cl-5.619571   0.340142   4.617814
Cl-0.327007   6.784227 -0.965719

Mn24/viii
Frequencies, energies and thermodynamic properties:
Lowest Vibrational Mode (1/cm) =   20.7953
2nd Lowest Vibrational Mode (1/cm) =   26.0572
E(RB-P86) (a.u.) =   -5171.56578396
Thermal correction to Enthalpy (a.u.) =   0.541827
Thermal correction to Gibbs Free Energy (a.u.) =   0.430618
Total Entropy (cal/Kmol) =   234.059
E(RPBE1PBE) (a.u.) =   -5170.82237586
Optimised cartesian coordinates (Angstrom):
Fe-2.693500 -1.385331 -1.321225
Mn0.832818 -0.939060   2.011000
P   0.115173   0.203698   0.205503
O   1.166395   1.547072   3.591112
O   -1.833505 -1.331429   3.195647
N   0.798795 -2.651300   0.884765
N   2.809583 -1.003349   1.391131
C   -0.736733 -0.835817 -1.037782
C   -0.873878 -2.285295 -0.936981
C   -1.419591 -2.743007 -2.192594
H   -1.665444 -3.783242 -2.441926
C   -1.631022 -1.609978 -3.054926
H   -2.056648 -1.637998 -4.067243
C   -1.221242 -0.428557 -2.346030
H   -1.258236   0.597202 -2.734768
C   -3.839892 -1.036410   0.357023
H   -3.455134 -0.862090   1.369751
C   -4.155601 -2.320158 -0.209761
H   -4.057620 -3.290413   0.295435
C   -4.597652 -2.112807 -1.565584
H   -4.892608 -2.897139 -2.275567
C   -4.558243 -0.697528 -1.834957
H   -4.819777 -0.214842 -2.786245
C   -4.086976 -0.031811 -0.648084
H   -3.929244   1.048328 -0.532331
C   1.508167   0.977927 -0.756828
C   2.178223   2.087820 -0.189551
H   1.837928   2.508402   0.770506
C   3.274748   2.680059 -0.833722
H   3.784900   3.547916 -0.390519
C   3.718315   2.147375 -2.058646
C   3.079626   1.038529 -2.638073
H   3.436332   0.632931 -3.596295
C   1.977988   0.459822 -1.982199
H   1.476708 -0.402027 -2.448538
C   -0.998866   1.684339   0.417431
C   -1.774147   1.848378   1.585139
H   -1.709476   1.118268   2.403348
C   -2.645695   2.941072   1.732602
H   -3.243476   3.059670   2.648164
C   -2.742423   3.886398   0.697481
C   -1.976238   3.753862 -0.474678
H   -2.053822   4.504530 -1.274902
C   -1.108533   2.657852 -0.603479
H   -0.499537   2.576868 -1.516922
C   -0.447192 -3.120494   0.280477
H   -1.232959 -2.990037   1.054175
C   1.943950 -2.798760   0.015175
H   2.272117 -3.863396 -0.111505
H   1.765508 -2.445887 -1.045466

```
 C      3.113791  -2.004974   0.519045
 C      3.815091  -0.216734   1.857820
 C      5.150534  -0.394106   1.486689
 C      5.473365  -1.432399   0.592837
 C      4.440461  -2.243646   0.108921
 C     -0.423828  -4.627816  -0.060101
 H     -1.435436  -4.989737  -0.337789
 H      0.256973  -4.860671  -0.905335
 H     -0.084802  -5.202516   0.825886
 C      1.043466   0.541805   2.980988
 C     -0.790589  -1.156674   2.666435
 H      1.328473  -2.327018   2.990143
 H      1.527521  -1.710949   3.487997
 H      3.527541   0.578775   2.560699
 H      5.922463   0.271467   1.899949
 H      4.645636  -3.069494  -0.589093
 H      6.515431  -1.605814   0.282804
 Cl5.090743   2.881203  -2.873579
 Cl-3.824768   5.257405   0.872992
---------------------------------------
Mn24/ix
Frequencies, energies and thermodynamic properties:
 Lowest Vibrational Mode (1/cm) =                         19.8099
 2nd Lowest Vibrational Mode (1/cm) =                     23.2237
 E(RB-P86) (a.u.) =                                      -5325.35378879
 Thermal correction to Enthalpy (a.u.) =                  0.611177
 Thermal correction to Gibbs Free Energy (a.u.) =         0.490979
 Total Entropy (cal/Kmol) =                               252.979
 E(RPBE1PBE) (a.u.) =                                    -5324.61385345
Optimised cartesian coordinates (Angstrom):
 Fe-2.802382  -1.064863  -1.697600
 Mn1.047939  -1.185030   1.275504
 P     -0.037323   0.382008   0.069650
 O      1.335384   0.588782   3.605110
 O     -1.381287  -2.331568   2.471944
 N      1.061239  -2.458670  -0.434890
 N      2.908538  -0.716146   0.460975
 C     -0.914610  -0.318985  -1.392735
 C     -0.858157  -1.718310  -1.818558
 C     -1.504281  -1.800041  -3.108117
 H     -1.647824  -2.713906  -3.698118
 C     -1.968549  -0.491309  -3.478987
 H     -2.522204  -0.239361  -4.393347
 C     -1.616650   0.420808  -2.426847
 H     -1.836005   1.495946  -2.413406
 C     -3.748946  -1.518985   0.076865
 H     -3.256655  -1.653582   1.048511
 C     -3.981345  -2.552726  -0.896744
 H     -3.702661  -3.610216  -0.794838
 C     -4.622900  -1.954164  -2.039235
 H     -4.912666  -2.473302  -2.962615
 C     -4.789719  -0.548724  -1.770010
 H     -5.229739   0.190628  -2.452543
 C     -4.247352  -0.278620  -0.463864
 H     -4.204680   0.701711   0.027969
 C      1.085652   1.654522  -0.704188
 C      1.692775   2.608597   0.147353
 H      1.438421   2.632524   1.219525
 C      2.617236   3.540700  -0.347045
 H      3.076406   4.284676   0.320525
 C      2.955556   3.511435  -1.713170
 C      2.379661   2.567234  -2.578835
 H      2.651641   2.555681  -3.644645
 C      1.447826   1.645108  -2.068125
 H      0.991132   0.920458  -2.759533
 C     -1.300789   1.503732   0.859873
 C     -1.916041   1.146817   2.078857
 H     -1.641269   0.211818   2.585761
 C     -2.889976   1.969195   2.670998
 H     -3.361716   1.681805   3.622091
 C     -3.252462   3.169570   2.036502
 C     -2.649541   3.556795   0.826063
 H     -2.934621   4.503255   0.343486
 C     -1.676790   2.723959   0.250489
 H     -1.196912   3.048394  -0.685689
 C     -0.221881  -2.879680  -1.061308
 H     -0.874053  -3.135498  -0.201980
 C      2.129082  -2.104818  -1.384250
 H      2.536321  -2.988555  -1.922497
 H      1.700830  -1.435709  -2.165444
 C      3.243014  -1.367925  -0.684395
 C      3.862218   0.012477   1.090915
 C      5.174145   0.120904   0.615800
 C      5.526990  -0.560781  -0.561270
 C      4.544870  -1.316600  -1.215720
```

```
 C     -0.078539  -4.145856  -1.923081
 H     -1.076861  -4.526674  -2.217508
 H      0.500911  -3.964670  -2.851173
 H      0.426086  -4.946007  -1.344044
 C      1.226615  -0.139596   2.676234
 C     -0.429504  -1.844804   1.960980
 H      1.420193  -3.177800   0.247951
 H      4.775336  -1.870030  -2.138637
 H      5.905188   0.726477   1.171359
 H      3.556452   0.519383   2.017700
 H      6.551667  -0.507684  -0.959999
 O      2.095600  -2.837254   1.839119
 C      1.971018  -3.500881   3.061865
 C      2.762192  -2.857556   4.212960
 H      2.348335  -4.551671   2.936298
 H      0.901895  -3.613549   3.391365
 H      2.678463  -3.460958   5.143849
 H      3.839494  -2.777933   3.950685
 H      2.390379  -1.835330   4.432887
 Cl-4.465147   4.206426   2.769513
 Cl4.114622   4.672799  -2.342237
---------------------------------------
Mn24/x
Frequencies, energies and thermodynamic properties:
 Lowest Vibrational Mode (1/cm) =                         6.8722
 2nd Lowest Vibrational Mode (1/cm) =                     20.2044
 E(RB-P86) (a.u.) =                                      -5480.29518625
 Thermal correction to Enthalpy (a.u.) =                  0.695294
 Thermal correction to Gibbs Free Energy (a.u.) =         0.560799
 Total Entropy (cal/Kmol) =                               283.069
 E(RPBE1PBE) (a.u.) =                                    -5479.56333468
Optimised cartesian coordinates (Angstrom):
 Fe-2.454063  -2.008911  -1.679707
 Mn1.254069  -0.391943   1.076898
 P     -0.553454   0.486160   0.039436
 O      0.710871   1.295926   3.423961
 O     -0.163803  -2.651711   2.315149
 N      1.731196  -1.473312  -0.726651
 N      2.591311   0.920373   0.175849
 C     -1.123401  -0.470697  -1.422931
 C     -0.448992  -1.657075  -1.947105
 C     -1.078857  -1.986532  -3.204973
 H     -0.821566  -2.836384  -3.849286
 C     -2.131806  -1.041559  -3.458185
 H     -2.808790  -1.048552  -4.322849
 C     -2.170106  -0.111898  -2.363993
 H     -2.867200   0.729774  -2.263436
 C     -2.902409  -2.954891   0.095018
 H     -2.306955  -2.903033   1.015678
 C     -2.735828  -3.919040  -0.960086
 H     -1.996230  -4.730694  -0.982324
 C     -3.692921  -3.619168  -1.994006
 H     -3.806197  -4.157370  -2.944571
 C     -4.454446  -2.470152  -1.575788
 H     -5.250413  -1.979604  -2.151881
 C     -3.964770  -2.057237  -0.286507
 H     -4.323421  -1.199292   0.296361
 C     -0.242786   2.182464  -0.670531
 C     -0.078379   3.260188   0.232746
 H     -0.206243   3.098967   1.315356
 C      0.239867   4.548340  -0.222501
 H      0.356353   5.381408   0.486278
 C      0.412049   4.764240  -1.602712
 C      0.267370   3.711358  -2.520888
 H      0.402598   3.893487  -3.597227
 C     -0.059945   2.427243  -2.048404
 H     -0.187727   1.614889  -2.780141
 C     -2.118934   0.816770   0.998908
 C     -2.387321   0.133392   2.204082
 H     -1.663070  -0.586981   2.607796
 C     -3.580982   0.350385   2.913534
 H     -3.778721  -0.186965   3.852562
 C     -4.520594   1.267084   2.411917
 C     -4.277101   1.969307   1.217703
 H     -5.016102   2.690625   0.838934
 C     -3.077834   1.742109   0.523989
 H     -2.890109   2.312637  -0.398582
 C      0.705758  -2.409169  -1.301535
 H      0.315251  -2.956759  -0.420540
 C      2.335005  -0.568210  -1.728025
 H      3.016638  -1.098716  -2.427508
 H      1.521587  -0.134835  -2.352863
 C      3.062384   0.561731  -1.048342
 C      3.164914   1.974440   0.804826
 C      4.220329   2.708068   0.250145
```

```
 C      4.719538   2.332084  -1.007831
 C      4.130774   1.240428  -1.661720
 C      1.340881  -3.449227  -2.240349
 H      0.603289  -4.234184  -2.501344
 H      1.706172  -3.000395  -3.186669
 H      2.191995  -3.938499  -1.725700
 C      0.943662   0.609667   2.487558
 C      0.359718  -1.727200   1.794623
 H      3.500215  -2.362221   1.050994
 H      2.503871  -2.097205  -0.376625
 O      3.775392  -3.121667   0.367445
 C      5.115460  -2.894177  -0.046103
 H      5.238676  -3.308013  -1.074524
 H      5.333751  -1.799529  -0.119724
 H      4.489830   0.906967  -2.646962
 H      4.644446   3.556212   0.807352
 H      2.758687   2.234190   1.792818
 C      6.136083  -3.554860   0.885011
 H      5.944745  -4.646166   0.960470
 H      7.173014  -3.409964   0.512521
 H      6.078242  -3.127748   1.908852
 H      5.556115   2.877732  -1.470550
 O      3.010699  -1.213810   1.787422
 C      3.246754  -1.436869   3.159149
 C      3.995885  -0.287921   3.847214
 H      3.860547  -2.369303   3.270788
 H      2.299530  -1.637427   3.722028
 H      4.230243  -0.546040   4.903394
 H      4.952365  -0.074441   3.324208
 H      3.391565   0.642476   3.852560
 Cl-6.013338   1.548355   3.292032
 Cl0.812856   6.373350  -2.183026
---------------------------------------
Mn24/TS-i
Frequencies, energies and thermodynamic properties:
 Lowest Vibrational Mode (1/cm) =-749.5996
 2nd Lowest Vibrational Mode (1/cm) =                      17.5618
 E(RB-P86) (a.u.) =                                       -5326.50726973
 Thermal correction to Enthalpy (a.u.) =                   0.622891
 Thermal correction to Gibbs Free Energy (a.u.) =          0.499951
 Total Entropy (cal/Kmol) =                                258.749
 E(RPBE1PBE) (a.u.) =                                     -5325.76598596
Optimised cartesian coordinates (Angstrom):
 Fe-2.379127  -1.772934  -1.654356
 Mn1.196740  -0.662935   1.465571
 P     -0.249748   0.470828   0.135006
 O      0.735613   1.207559   3.705936
 O     -0.801789  -2.501577   2.605690
 N      1.684780  -1.903396  -0.161186
 N      2.876259   0.308265   0.747430
 C     -0.815119  -0.490101  -1.317643
 C     -0.319115  -1.817659  -1.672633
 C     -0.849019  -2.130063  -2.979137
 H     -0.683834  -3.064034  -3.530902
 C     -1.667450  -1.035410  -3.426984
 H     -2.225881  -0.991758  -4.371694
 C     -1.659251  -0.025356  -2.405327
 H     -2.191089   0.933869  -2.446972
 C     -3.236312  -2.434267   0.100878
 H     -2.779915  -2.382294   1.097395
 C     -3.094257  -3.521523  -0.830159
 H     -2.513894  -4.439495  -0.666747
 C     -3.824933  -3.183911  -2.024999
 H     -3.895317  -3.796977  -2.933454
 C     -4.421692  -1.886927  -1.830315
 H     -5.027864  -1.339510  -2.564487
 C     -4.055735  -1.421426  -0.517808
 H     -4.338064  -0.459198  -0.071443
 C      0.499440   1.999249  -0.618907
 C      0.784986   3.091579   0.234824
 H      0.512272   3.046994   1.301583
 C      1.405533   4.251061  -0.253399
 H      1.615898   5.098960   0.414861
 C      1.759876   4.317217  -1.613978
 C      1.499010   3.243381  -2.481086
 H      1.779367   3.308662  -3.542677
 C      0.869928   2.090498  -1.977486
 H      0.656619   1.261111  -2.668854
 C     -1.812907   1.185775   0.854572
 C     -2.368499   0.666861   2.043684
 H     -1.869543  -0.150388   2.582152
 C     -3.569035   1.175899   2.567587
 H     -3.991838   0.765359   3.496157
 C     -4.223489   2.221306   1.893983
 C     -3.689338   2.763731   0.711181
```

```
 H     -4.205903   3.587423   0.196916
 C     -2.487551   2.245277   0.203566
 H     -2.069473   2.691758  -0.711748
 C      0.612225  -2.685378  -0.827811
 H      0.012713  -3.114882   0.000856
 C      2.556160  -1.209729  -1.115902
 H      3.219079  -1.905712  -1.680957
 H      1.958583  -0.674943  -1.898154
 C      3.394650  -0.178725  -0.414828
 C      3.556126   1.284485   1.403108
 C      4.768687   1.805552   0.940611
 C      5.314624   1.297499  -0.250977
 C      4.615957   0.292192  -0.931917
 C      1.175688  -3.878993  -1.625875
 H      0.358881  -4.554646  -1.951415
 H      1.730456  -3.560839  -2.532682
 H      1.860807  -4.459982  -0.976227
 C      0.931544   0.457224   2.815639
 C     -0.039254  -1.749374   2.107279
 H      2.352580  -1.291141   2.554742
 H      2.390146  -2.013811   2.087737
 H      2.402875  -2.764606   0.545880
 O      2.931315  -3.451376   1.372647
 C      4.338091  -3.416877   1.300438
 H      4.697270  -3.549555   0.245563
 H      4.747841  -2.423056   1.631916
 H      5.005854  -0.137254  -1.866977
 H      5.275123   2.593957   1.516299
 H      3.107526   1.650048   2.338175
 C      4.957909  -4.512114   2.172188
 H      4.603319  -5.514029   1.848871
 H      6.067430  -4.499608   2.112668
 H      4.667594  -4.378656   3.236157
 H      6.271419   1.677724  -0.640519
 Cl-5.721344   2.866846   2.542055
 Cl2.538445   5.763806  -2.234716
---------------------------------------
Mn24/TS-ii_si
Frequencies, energies and thermodynamic properties:
 Lowest Vibrational Mode (1/cm) =-251.2407
 2nd Lowest Vibrational Mode (1/cm) =                     13.1351
 E(RB-P86) (a.u.) =                                     -5594.30702201
 Thermal correction to Enthalpy (a.u.) =                 0.695885
 Thermal correction to Gibbs Free Energy (a.u.) =        0.565474
 Total Entropy (cal/Kmol) =                              274.474
 E(RPBE1PBE) (a.u.) =                                   -5593.55237885
Optimised cartesian coordinates (Angstrom):
 Fe-2.198959  -2.226432  -2.000554
 Mn1.267410   0.201466   0.352886
 P     -0.975722   0.357516   0.154008
 O      1.389741   0.664003   3.256896
 O      1.416310  -2.689641   0.870966
 N      1.474942   0.033029  -1.735688
 N      1.665417   2.154823  -0.133034
 C     -1.595258  -0.352917  -1.425721
 C     -0.737581  -0.869217  -2.493701
 C     -1.588208  -1.137243  -3.630930
 H     -1.262862  -1.550869  -4.593581
 C     -2.944954  -0.813160  -3.284354
 H     -3.822140  -0.936819  -3.933423
 C     -2.957187  -0.337409  -1.929338
 H     -3.845070  -0.011955  -1.372237
 C     -1.582460  -3.677194  -0.673638
 H     -0.782302  -3.564701   0.069209
 C     -1.412057  -4.129756  -2.029030
 H     -0.463504  -4.427059  -2.496115
 C     -2.699699  -4.102613  -2.674285
 H     -2.903437  -4.368556  -3.720159
 C     -3.667939  -3.635528  -1.715374
 H     -4.739401  -3.483801  -1.902618
 C     -2.978036  -3.369769  -0.479927
 H     -3.430679  -2.983993   0.442561
 C     -1.609482   2.108811   0.101701
 C     -1.529334   2.871813   1.291825
 H     -1.174879   2.402631   2.224129
 C     -1.901633   4.224043   1.315741
 H     -1.845744   4.805756   2.247671
 C     -2.345722   4.833182   0.126900
 C     -2.420216   4.105721  -1.072206
 H     -2.769313   4.593482  -1.994340
 C     -2.051630   2.747864  -1.076724
 H     -2.129941   2.184290  -2.019120
 C     -2.100118  -0.363332   1.453845
 C     -1.626980  -1.366974   2.325740
 H     -0.586516  -1.713796   2.254255
```

```
 C     -2.466768  -1.943940   3.293805
 H     -2.088627  -2.725046   3.969554
 C     -3.798820  -1.506781   3.393997
 C     -4.295484  -0.501223   2.544848
 H     -5.337569  -0.161970   2.639389
 C     -3.441808   0.064833   1.584378
 H     -3.835169   0.864587   0.937832
 C      0.770915  -1.088214  -2.442741
 H      0.973737  -1.974810  -1.809604
 C      1.303852   1.347035  -2.391532
 H      1.850585   1.415354  -3.356536
 H      0.224660   1.489946  -2.626648
 C      1.738455   2.447804  -1.464220
 C      1.964167   3.143863   0.752733
 H      1.893167   2.880644   1.817729
 C      2.352618   4.428949   0.359061
 H      2.581378   5.182553   1.126899
 C      2.449986   4.722987  -1.011637
 C      2.138525   3.711244  -1.931443
 H      2.196941   3.890869  -3.015513
 C      1.377303  -1.366302  -3.829013
 H      0.976474  -2.313157  -4.242310
 H      1.159386  -0.560025  -4.558736
 H      2.477198  -1.477338  -3.743646
 C      1.320396   0.480330   2.087815
 C      1.303753  -1.532190   0.642736
 H      2.513333  -0.177352  -1.780635
 H      2.911308   0.013182   0.368867
 C      4.399364  -0.135887  -0.383624
 C      4.800844  -1.340619   0.427608
 C      5.014281   1.071758   0.366526
 C      5.321942  -0.928154   1.674206
 C      4.769224  -2.694611   0.056684
 C      5.238102   0.582065   1.813957
 H      5.991278   1.269163  -0.132016
 C      5.814930  -1.885546   2.576074
 C      5.266628  -3.650682   0.960218
 H      4.369697  -2.989143  -0.927021
 H      6.135819   1.018884   2.298359
 C      5.783083  -3.247144   2.210961
 H      6.229368  -1.581735   3.551550
 H      5.260336  -4.719581   0.692441
 H      6.173534  -4.006533   2.907710
 O      4.166181  -0.155581  -1.626172
 H      4.407893   1.992601   0.275991
 H      4.366463   0.847198   2.454831
 H      2.763264   5.719900  -1.357081
 Cl-2.811104   6.527742   0.145001
 Cl-4.855809  -2.216893   4.603264
---------------------------------------
Mn24/TS-ii_re
Frequencies, energies and thermodynamic properties:
 Lowest Vibrational Mode (1/cm) =-250.0491
 2nd Lowest Vibrational Mode (1/cm) =                      11.4967
 E(RB-P86) (a.u.) =                                      -5594.30755320
 Thermal correction to Enthalpy (a.u.) =                   0.695965
 Thermal correction to Gibbs Free Energy (a.u.) =          0.565568
 Total Entropy (cal/Kmol) =                                274.444
 E(RPBE1PBE) (a.u.) =                                    -5593.55392203
Optimised cartesian coordinates (Angstrom):
 Fe-3.209959  -1.575880  -1.625867
 Mn1.057355  -0.593277   0.456739
 P     -0.871025   0.524311   0.094641
 O      1.433290   0.301416   3.235790
 O     -0.115130  -3.083130   1.490807
 N      1.099909  -1.236254  -1.547257
 N      2.287959   0.833065  -0.358713
 C     -1.795468  -0.115260  -1.361386
 C     -1.301259  -1.157583  -2.262013
 C     -2.210465  -1.217770  -3.383642
 H     -2.138801  -1.908917  -4.232645
 C     -3.255303  -0.250166  -3.188518
 H     -4.108435  -0.080165  -3.858837
 C     -3.010083   0.425658  -1.945309
 H     -3.632624   1.220043  -1.514000
 C     -4.370714  -1.934997   0.033783
 H     -4.552400  -1.226721   0.852269
 C     -3.290152  -2.887426  -0.037202
 H     -2.502393  -3.034889   0.713087
 C     -3.406640  -3.598005  -1.283136
 H     -2.727222  -4.381063  -1.645870
 C     -4.556862  -3.086447  -1.983375
 H     -4.903619  -3.405807  -2.975261
 C     -5.154148  -2.060397  -1.167530
 H     -6.036687  -1.461215  -1.428694
```

```
 C     -0.636673   2.325670  -0.320205
 C     -0.192351   3.187128   0.711983
 H     -0.071984   2.802212   1.737758
 C      0.094056   4.537100   0.460213
 H      0.428828   5.201036   1.270832
 C     -0.048309   5.034877  -0.848737
 C     -0.472073   4.200191  -1.895816
 H     -0.579824   4.601801  -2.914238
 C     -0.763382   2.850788  -1.624449
 H     -1.110040   2.210745  -2.450278
 C     -2.159649   0.655814   1.434215
 C     -2.169607  -0.264580   2.503883
 H     -1.403952  -1.050978   2.561627
 C     -3.150127  -0.200714   3.508737
 H     -3.148915  -0.921973   4.339132
 C     -4.133018   0.801952   3.444800
 C     -4.142885   1.738564   2.395246
 H     -4.912668   2.523598   2.361231
 C     -3.154770   1.660310   1.400692
 H     -3.160396   2.410083   0.594368
 C     -0.062532  -2.026616  -2.074730
 H     -0.272069  -2.763485  -1.274211
 C      1.530354  -0.145305  -2.447677
 H      2.033730  -0.524265  -3.363256
 H      0.628153   0.410648  -2.790118
 C      2.430290   0.811887  -1.715318
 C      3.024945   1.733213   0.344966
 H      2.894767   1.720685   1.436385
 C      3.908874   2.633629  -0.259163
 H      4.471766   3.340541   0.367756
 C      4.063321   2.605300  -1.655136
 C      3.315342   1.672162  -2.387503
 H      3.401591   1.608275  -3.482696
 C      0.305972  -2.818631  -3.340965
 H     -0.500735  -3.534227  -3.596323
 H      0.468082  -2.163792  -4.221375
 H      1.229739  -3.405035  -3.161641
 C      1.272732  -0.044232   2.113845
 C      0.303598  -2.060409   1.057100
 H      1.920238  -1.906541  -1.486393
 H      2.454841  -1.469054   0.597937
 C      3.623318  -2.508962  -0.001804
 C      4.759012  -1.696825   0.562372
 C      3.352797  -3.606008   1.058148
 C      4.962224  -2.034416   1.919855
 C      5.595033  -0.782437  -0.099441
 C      3.927508  -3.047505   2.377985
 C      6.014838  -1.442011   2.636445
 C      6.649908  -0.193087   0.620564
 H      5.425980  -0.549685  -1.163120
 C      6.855343  -0.520253   1.978594
 H      6.191710  -1.697781   3.694233
 H      7.325790   0.521409   0.123744
 H      7.689135  -0.055178   2.529113
 O      3.383222  -2.638720  -1.236438
 H      4.354751  -3.825916   3.043435
 H      3.131840  -2.529314   2.960445
 H      2.291676  -3.912324   1.106706
 H      3.942633  -4.490491   0.723600
 H      4.756029   3.293267  -2.163325
 Cl0.310564   6.723825  -1.175674
 Cl-5.359515   0.894717   4.698107
--------------------------------------
Mn24/TS-iii
Frequencies, energies and thermodynamic properties:
 Lowest Vibrational Mode (1/cm) =-633.7487
 2nd Lowest Vibrational Mode (1/cm) =                      20.8360
 E(RB-P86) (a.u.) =                                      -5171.56266322
 Thermal correction to Enthalpy (a.u.) =                  0.540829
 Thermal correction to Gibbs Free Energy (a.u.) =         0.430421
 Total Entropy (cal/Kmol) =                               232.374
 E(RPBE1PBE) (a.u.) =                                    -5170.81606873
Optimised cartesian coordinates (Angstrom):
 Fe-2.681530  -1.375522  -1.326611
 Mn0.814537  -0.979650   1.988090
 P      0.119974   0.236151   0.202941
 O      1.207478   1.315705   3.810544
 O     -1.859278  -1.353902   3.157991
 N      0.825780  -2.717647   0.829108
 N      2.801931  -1.023842   1.387643
 C     -0.727837  -0.817510  -1.037204
 C     -0.861501  -2.273192  -0.953374
 C     -1.404020  -2.715205  -2.217104
 H     -1.646126  -3.752642  -2.480541
 C     -1.620069  -1.573649  -3.064996
```

```
 H     -2.047412  -1.591493  -4.076691
 C     -1.214041  -0.400732  -2.341886
 H     -1.256670   0.629769  -2.717323
 C     -3.836624  -1.033021   0.347358
 H     -3.456857  -0.854727   1.361082
 C     -4.140136  -2.320160  -0.218858
 H     -4.036742  -3.288898   0.288158
 C     -4.579361  -2.118074  -1.576178
 H     -4.865404  -2.905547  -2.286314
 C     -4.549912  -0.702920  -1.847351
 H     -4.811298  -0.223687  -2.800406
 C     -4.088027  -0.031945  -0.659789
 H     -3.938705   1.049519  -0.545157
 C      1.503201   1.022574  -0.764612
 C      2.186672   2.112587  -0.174656
 H      1.857106   2.510237   0.799051
 C      3.282204   2.714066  -0.811998
 H      3.802221   3.566363  -0.350399
 C      3.712673   2.210201  -2.053694
 C      3.061775   1.120654  -2.655785
 H      3.408542   0.736746  -3.626542
 C      1.961233   0.532920  -2.006059
 H      1.451895  -0.314353  -2.490226
 C     -1.003557   1.706989   0.421267
 C     -1.766145   1.859426   1.598961
 H     -1.687924   1.122382   2.410086
 C     -2.640232   2.947654   1.764018
 H     -3.228705   3.057632   2.686673
 C     -2.750791   3.900436   0.737015
 C     -1.995991   3.780215  -0.443979
 H     -2.084355   4.537388  -1.236944
 C     -1.126177   2.688094  -0.590716
 H     -0.525482   2.616327  -1.510516
 C     -0.436493  -3.156403   0.230648
 H     -1.204612  -3.043253   1.024098
 C      1.968374  -2.784558  -0.059780
 H      2.307063  -3.831000  -0.265439
 H      1.753630  -2.351447  -1.078758
 C      3.126427  -2.001867   0.495870
 C      3.797098  -0.249328   1.892517
 C      5.140008  -0.412749   1.538694
 C      5.482132  -1.426339   0.626169
 C      4.459731  -2.229242   0.104936
 C     -0.423685  -4.651350  -0.156990
 H     -1.437679  -4.998462  -0.443564
 H      0.255183  -4.860729  -1.009707
 H     -0.087717  -5.255455   0.710100
 C      1.050474   0.398663   3.082527
 C     -0.814018  -1.190371   2.628653
 H      1.154805  -2.575941   2.385169
 H      1.345634  -2.102938   3.155394
 H      3.497220   0.524587   2.613734
 H      5.901829   0.245095   1.981981
 H      4.680106  -3.038199  -0.607866
 H      6.529905  -1.589624   0.330400
 Cl-3.836035   5.266377   0.934145
 Cl5.084705   2.954548  -2.860104
---------------------------------------
Mn25/i
Frequencies, energies and thermodynamic properties:
 Lowest Vibrational Mode (1/cm) =                        12.9392
 2nd Lowest Vibrational Mode (1/cm) =                    13.4647
 E(RB-P86) (a.u.) =                                     -4924.98606827
 Thermal correction to Enthalpy (a.u.) =                 0.558197
 Thermal correction to Gibbs Free Energy (a.u.) =        0.433644
 Total Entropy (cal/Kmol) =                              262.145
 E(RPBE1PBE) (a.u.) =                                   -4924.48348614
Optimised cartesian coordinates (Angstrom):
 Fe-2.361006  -1.981059  -1.632301
 Mn0.670507  -1.304136   1.976270
 P      0.004279  -0.100852   0.284918
 O      0.689783   0.825415   4.045965
 O     -1.998795  -2.043642   2.960589
 N      0.946499  -2.864919   0.934546
 N      2.721832  -1.201338   1.787853
 C     -0.550445  -1.187661  -1.091457
 C     -0.495719  -2.648028  -1.061624
 C     -0.820144  -3.113384  -2.388415
 H     -0.888573  -4.163207  -2.700912
 C     -1.078928  -1.975865  -3.230971
 H     -1.372186  -2.009861  -4.288974
 C     -0.922266  -0.787849  -2.437525
 H     -1.051837   0.241845  -2.794727
 C     -3.724523  -1.914733  -0.088065
 H     -3.480934  -1.785273   0.974451
```

```
 C     -3.811202  -3.172092  -0.781946
 H     -3.650159  -4.164979  -0.340867
 C     -4.120292  -2.900376  -2.162705
 H     -4.231638  -3.648721  -2.958747
 C     -4.228015  -1.472523  -2.321704
 H     -4.437477  -0.942203  -3.260343
 C     -3.980730  -0.862531  -1.040943
 H     -3.973446   0.214463  -0.829409
 C      1.409682   0.859226  -0.469247
 C      1.859337   2.028935   0.188954
 H      1.328487   2.399108   1.080654
 C      2.978085   2.729733  -0.280031
 H      3.312869   3.640564   0.239008
 C      3.679309   2.260481  -1.410969
 C      3.254244   1.087655  -2.060981
 H      3.802999   0.714180  -2.938320
 C      2.126499   0.392193  -1.592119
 H      1.798845  -0.516509  -2.119101
 C     -1.299421   1.223743   0.413992
 C     -2.185469   1.240107   1.511452
 H     -2.097112   0.485000   2.304928
 C     -3.193226   2.213895   1.604936
 H     -3.879908   2.211079   2.464367
 C     -3.323379   3.190034   0.599431
 C     -2.439902   3.189195  -0.498161
 H     -2.537138   3.949976  -1.287153
 C     -1.434361   2.215053  -0.585702
 H     -0.743541   2.240302  -1.442263
 C     -0.143956  -3.498285   0.170114
 H     -1.026359  -3.480455   0.840260
 C      2.261098  -3.083310   0.360942
 H      2.605723  -4.140384   0.470413
 H      2.275586  -2.894756  -0.746184
 C      3.264135  -2.174712   1.000960
 C      3.567247  -0.317592   2.389725
 H      3.099422   0.455708   3.015632
 C      4.954118  -0.378087   2.243805
 H      5.585144   0.361184   2.758787
 C      5.515112  -1.390144   1.440126
 C      4.653370  -2.298517   0.814184
 H      5.041960  -3.106848   0.176157
 C      0.106795  -4.973456  -0.193423
 H     -0.820570  -5.432183  -0.591894
 H      0.898492  -5.099859  -0.959866
 H      0.400680  -5.542321   0.712238
 C      0.675426  -0.020819   3.217537
 C     -0.948350  -1.732198   2.506315
 H      6.605117  -1.466308   1.307407
 C     -4.370782   4.274914   0.722873
 F     -3.876463   5.380471   1.347067
 F     -4.825130   4.683021  -0.490203
 F     -5.446631   3.867296   1.442805
 C      4.853999   3.047842  -1.944586
 F      5.580868   3.615483  -0.946292
 F      5.701026   2.277823  -2.674249
 F      4.451280   4.065763  -2.757358
---------------------------------------
Mn25/ii
Frequencies, energies and thermodynamic properties:
 Lowest Vibrational Mode (1/cm) =                        14.3534
 2nd Lowest Vibrational Mode (1/cm) =                    15.6189
 E(RB-P86) (a.u.) =                                     -5079.92043439
 Thermal correction to Enthalpy (a.u.) =                 0.643003
 Thermal correction to Gibbs Free Energy (a.u.) =        0.504078
 Total Entropy (cal/Kmol) =                              292.391
 E(RPBE1PBE) (a.u.) =                                   -5079.42678022
Optimised cartesian coordinates (Angstrom):
 Fe1.931720  -2.034755   2.126714
 Mn-1.126481  -1.039351  -1.388547
 P      0.181724   0.143761  -0.100678
 O     -0.630125   0.419100  -3.922973
 O      0.925291  -2.951729  -2.269167
 N     -1.819223  -2.112370   0.048107
 N     -2.925650  -0.072292  -1.102514
 C      0.483851  -0.736299   1.483544
 C     -0.108801  -2.024755   1.840862
 C      0.209771  -2.268894   3.226832
 H     -0.069344  -3.162003   3.800329
 C      0.989561  -1.168406   3.727518
 H      1.400642  -1.077528   4.741965
 C      1.168674  -0.224917   2.658543
 H      1.719688   0.721851   2.726352
 C      2.993046  -2.844728   0.556122
 H      2.671820  -2.859810  -0.493336
 C      2.699117  -3.854324   1.537891
```

```
 H      2.119211  -4.771229   1.366996
 C      3.275531  -3.439828   2.791458
 H      3.208389  -3.982756   3.743713
 C      3.929805  -2.173294   2.583173
 H      4.449958  -1.582550   3.349065
 C      3.753353  -1.803335   1.202792
 H      4.120702  -0.883926   0.728851
 C     -0.643882   1.725152   0.429458
 C     -0.728404   2.785646  -0.504220
 H     -0.243262   2.694747  -1.489255
 C     -1.426052   3.959860  -0.192528
 H     -1.480019   4.777610  -0.926945
 C     -2.066922   4.086560   1.057952
 C     -2.004558   3.032213   1.986811
 H     -2.508640   3.124314   2.960273
 C     -1.296471   1.859090   1.673694
 H     -1.246301   1.048656   2.416310
 C      1.849188   0.777547  -0.635662
 C      2.516256   0.191789  -1.731894
 H      2.048625  -0.630103  -2.291192
 C      3.787091   0.644443  -2.122468
 H      4.297170   0.174174  -2.976192
 C      4.405313   1.696416  -1.421133
 C      3.746057   2.295121  -0.329487
 H      4.224228   3.118366   0.222007
 C      2.476203   1.840222   0.055613
 H      1.969478   2.331976   0.899925
 C     -0.922548  -2.924241   0.900834
 H     -0.206540  -3.400430   0.201451
 C     -2.975948  -1.593163   0.765474
 H     -3.714676  -2.388455   1.026724
 H     -2.681946  -1.142533   1.750155
 C     -3.654900  -0.533095  -0.045896
 C     -3.463869   0.909825  -1.878130
 C     -4.727802   1.452765  -1.640408
 C     -5.485330   0.972123  -0.554799
 C     -4.937951  -0.035225   0.248234
 C     -1.638008  -4.067271   1.644155
 H     -0.896864  -4.753444   2.101251
 H     -2.304862  -3.705251   2.453215
 H     -2.239457  -4.647420   0.915380
 C     -0.823500  -0.159380  -2.909317
 C      0.134168  -2.170350  -1.860902
 H     -2.488906  -3.408902  -1.157379
 O     -2.802033  -4.199222  -1.683297
 C     -4.192763  -4.048459  -1.939102
 H     -4.414751  -3.065411  -2.425392
 H     -4.788486  -4.070703  -0.990681
 H     -5.492125  -0.444145   1.106615
 H     -5.111806   2.241415  -2.303870
 H     -2.849850   1.258144  -2.720792
 C     -4.661402  -5.177512  -2.850184
 H     -5.747085  -5.091978  -3.062875
 H     -4.114376  -5.154555  -3.816155
 H     -4.477196  -6.165272  -2.377413
 H     -6.486569   1.377206  -0.342730
 C      5.749242   2.224822  -1.873437
 F      5.611719   3.241663  -2.769582
 F      6.478365   2.713643  -0.837486
 F      6.496210   1.267485  -2.479615
 C     -2.777374   5.374310   1.408943
 F     -3.389874   5.926673   0.329000
 F     -3.724984   5.194842   2.364064
 F     -1.911970   6.313001   1.886421
---------------------------------------
Mn25/iii
Frequencies, energies and thermodynamic properties:
 Lowest Vibrational Mode (1/cm) =                        11.6477
 2nd Lowest Vibrational Mode (1/cm) =                    12.0779
 E(RB-P86) (a.u.) =                                    -5081.09298244
 Thermal correction to Enthalpy (a.u.) =                 0.659250
 Thermal correction to Gibbs Free Energy (a.u.) =        0.521079
 Total Entropy (cal/Kmol) =                              290.804
 E(RPBE1PBE) (a.u.) =                                  -5080.59614538
Optimised cartesian coordinates (Angstrom):
 Fe1.865011  -2.142604   2.063952
 Mn-1.170175  -1.021002  -1.587019
 P      0.142365   0.094324  -0.114571
 O     -0.258595   0.723868  -3.798510
 O      0.851310  -3.030305  -2.329561
 N     -1.949692  -2.140037  -0.006773
 N     -2.867214   0.083828  -1.159272
 C      0.438307  -0.805429   1.447239
 C     -0.178542  -2.086496   1.781068
 C      0.140431  -2.355626   3.163246
```

```
 H     -0.154641  -3.250809   3.725040
 C      0.946512  -1.281531   3.678742
 H      1.364122  -1.216590   4.692494
 C      1.143416  -0.325814   2.624350
 H      1.715394   0.607507   2.703316
 C      2.919823  -2.951539   0.486983
 H      2.609671  -2.940983  -0.565502
 C      2.590424  -3.970833   1.446720
 H      1.988405  -4.868806   1.253573
 C      3.165313  -3.593796   2.712759
 H      3.074812  -4.151622   3.654430
 C      3.854183  -2.340947   2.533516
 H      4.382154  -1.778008   3.314847
 C      3.700565  -1.941681   1.158729
 H      4.097156  -1.024521   0.704480
 C     -0.604876   1.710558   0.435343
 C     -0.678968   2.766031  -0.504495
 H     -0.253443   2.641179  -1.512941
 C     -1.285582   3.983884  -0.167904
 H     -1.333718   4.796201  -0.908465
 C     -1.840041   4.161908   1.116002
 C     -1.784781   3.114816   2.053874
 H     -2.221387   3.246543   3.055071
 C     -1.170577   1.897425   1.714077
 H     -1.125328   1.093282   2.463730
 C      1.837107   0.681199  -0.635908
 C      2.517927   0.068545  -1.708834
 H      2.047523  -0.745661  -2.275887
 C      3.810600   0.482287  -2.070019
 H      4.328219  -0.009611  -2.906777
 C      4.441118   1.521102  -1.360998
 C      3.770828   2.145814  -0.291057
 H      4.257877   2.958248   0.268688
 C      2.478460   1.730970   0.062507
 H      1.966131   2.243935   0.890370
 C     -1.036411  -2.937863   0.840144
 H     -0.347873  -3.446519   0.133969
 C     -2.898408  -1.360422   0.783802
 H     -3.680555  -1.990678   1.272831
 H     -2.405917  -0.813017   1.634581
 C     -3.568108  -0.316293  -0.061483
 C     -3.384546   1.066589  -1.941537
 C     -4.611682   1.680588  -1.673554
 C     -5.346449   1.263642  -0.549364
 C     -4.814847   0.253071   0.261479
 C     -1.773278  -4.057162   1.608289
 H     -1.050629  -4.751636   2.083632
 H     -2.431552  -3.659777   2.408604
 H     -2.392668  -4.639570   0.897149
 C     -0.637843   0.023178  -2.927911
 C      0.076247  -2.209079  -1.985730
 H     -2.188498  -1.586315  -2.866247
 H     -2.283622  -2.205914  -2.321716
 H     -2.781305  -3.248173  -0.853124
 O     -3.221610  -3.942074  -1.499783
 C     -4.625523  -3.754195  -1.514501
 H     -5.065058  -3.869304  -0.489888
 H     -4.900710  -2.720765  -1.852118
 H     -5.355553  -0.106136   1.149909
 H     -4.982231   2.469038  -2.344705
 H     -2.788038   1.360848  -2.817344
 C     -5.273247  -4.771920  -2.450173
 H     -5.041574  -5.806882  -2.120609
 H     -6.376773  -4.652656  -2.471265
 H     -4.891754  -4.652691  -3.486382
 H     -6.320264   1.718876  -0.312016
 C     -2.446823   5.493459   1.498204
 F     -3.019812   6.115700   0.435640
 F     -3.400796   5.364784   2.455642
 F     -1.509267   6.350118   1.992747
 C      5.811289   2.007592  -1.779990
 F      5.727334   3.036968  -2.668306
 F      6.533643   2.462061  -0.723721
 F      6.538637   1.031285  -2.379446
---------------------------------------
Mn25/iv
Frequencies, energies and thermodynamic properties:
 Lowest Vibrational Mode (1/cm) =                         11.0311
 2nd Lowest Vibrational Mode (1/cm) =                     13.6377
 E(RB-P86) (a.u.) =                                     -5081.12252121
 Thermal correction to Enthalpy (a.u.) =                  0.663693
 Thermal correction to Gibbs Free Energy (a.u.) =         0.524305
 Total Entropy (cal/Kmol) =                               293.366
 E(RPBE1PBE) (a.u.) =                                   -5080.62372417
Optimised cartesian coordinates (Angstrom):
```

```
Fe2.025172  -1.912449   2.183012
Mn-1.161778  -1.140327  -1.419685
P      0.137695   0.131448  -0.062263
O     -0.558169   0.397514  -3.852110
O      0.836017  -3.149082  -2.215214
N     -1.850921  -2.245940   0.292701
N     -2.925404  -0.161512  -1.014885
C      0.531648  -0.661605   1.548358
C     -0.017226  -1.947239   1.983569
C      0.360824  -2.124863   3.367176
H      0.124644  -2.995478   3.991421
C      1.138710  -0.991408   3.786558
H      1.591188  -0.852946   4.777556
C      1.252717  -0.092028   2.672887
H      1.789240   0.865444   2.674688
C      3.035024  -2.761954   0.600889
H      2.671791  -2.818157  -0.433488
C      2.795503  -3.740654   1.628238
H      2.224096  -4.671433   1.512496
C      3.415933  -3.275250   2.842030
H      3.394671  -3.785236   3.814396
C      4.043115  -2.008741   2.563687
H      4.584754  -1.384645   3.287003
C      3.806144  -1.689434   1.179920
H      4.140237  -0.782148   0.660530
C     -0.653861   1.741817   0.447317
C     -0.850165   2.721590  -0.556709
H     -0.466113   2.549968  -1.575239
C     -1.527039   3.915631  -0.274934
H     -1.666243   4.667633  -1.066282
C     -2.038153   4.146584   1.019406
C     -1.867272   3.173558   2.021026
H     -2.269565   3.344669   3.030691
C     -1.179838   1.981406   1.735162
H     -1.044284   1.239683   2.536898
C      1.791248   0.760823  -0.663329
C      2.448105   0.108990  -1.728124
H      1.979235  -0.756886  -2.216130
C      3.705172   0.548483  -2.174489
H      4.206454   0.025479  -3.002448
C      4.321894   1.655170  -1.561290
C      3.673595   2.320652  -0.502027
H      4.150487   3.186357  -0.018279
C      2.417201   1.877359  -0.062303
H      1.919282   2.419943   0.755836
C     -0.829311  -2.934549   1.153945
H     -0.148213  -3.418954   0.426132
C     -2.830230  -1.437645   1.050440
H     -3.534177  -2.059157   1.645041
H     -2.271948  -0.808206   1.779655
C     -3.588401  -0.527865   0.120453
C     -3.533734   0.722977  -1.848934
C     -4.804526   1.254685  -1.602033
C     -5.497063   0.859820  -0.445487
C     -4.872940  -0.047258   0.424384
C     -1.460149  -4.049166   2.005825
H     -0.671640  -4.659886   2.488735
H     -2.118479  -3.652832   2.805608
H     -2.055545  -4.725299   1.359287
C     -0.794264  -0.216039  -2.865755
C      0.068954  -2.320118  -1.854098
H     -2.119396  -2.056752  -2.314974
H     -2.579260  -3.425998  -2.006317
H     -2.373202  -3.009006  -0.190506
O     -2.980437  -4.215799  -1.528732
C     -4.364739  -4.284649  -1.887569
H     -4.845696  -4.968538  -1.154909
H     -4.857908  -3.289968  -1.769006
H     -5.371793  -0.386862   1.344650
H     -5.241180   1.964700  -2.319824
H     -2.975166   0.999572  -2.754644
C     -4.574844  -4.807337  -3.308077
H     -4.102452  -5.803844  -3.433641
H     -5.657282  -4.904086  -3.537136
H     -4.129787  -4.118667  -4.057362
H     -6.502835   1.248735  -0.225364
C      5.649205   2.168167  -2.073826
F      6.391063   1.185982  -2.646194
F      5.483042   3.135375  -3.019397
F      6.399090   2.717739  -1.083390
C     -2.722478   5.456043   1.336042
F     -1.826237   6.424792   1.679833
F     -3.421051   5.936011   0.273680
F     -3.591121   5.344360   2.373961
---------------------------------------
```

Mn25/v
Frequencies, energies and thermodynamic properties:

Lowest Vibrational Mode (1/cm) =	11.1447
2nd Lowest Vibrational Mode (1/cm) =	12.3121
E(RB-P86) (a.u.) =	-4926.18443701
Thermal correction to Enthalpy (a.u.) =	0.579200
Thermal correction to Gibbs Free Energy (a.u.) =	0.454292
Total Entropy (cal/Kmol) =	262.892
E(RPBE1PBE) (a.u.) =	-4925.68147629

Optimised cartesian coordinates (Angstrom):

```
Fe-2.389277  -1.857949  -1.695450
Mn0.635268  -1.340692   2.086134
P      0.045531  -0.124450   0.268051
O      0.632190   0.910186   3.976762
O     -2.023039  -2.146840   3.052823
N      0.878707  -3.082727   0.851599
N      2.661701  -1.296621   1.762556
C     -0.549140  -1.146403  -1.141960
C     -0.562485  -2.611080  -1.146353
C     -0.901833  -3.030189  -2.487866
H     -1.015429  -4.066517  -2.829278
C     -1.111394  -1.863736  -3.300511
H     -1.408535  -1.861567  -4.357706
C     -0.902033  -0.704814  -2.479173
H     -0.988860   0.338459  -2.808721
C     -3.742195  -1.774984  -0.145130
H     -3.489083  -1.682766   0.919252
C     -3.884095  -3.009825  -0.870732
H     -3.763808  -4.019884  -0.456401
C     -4.188478  -2.689914  -2.241818
H     -4.333484  -3.412202  -3.056240
C     -4.238728  -1.255619  -2.363270
H     -4.429762  -0.692923  -3.286800
C     -3.960641  -0.689323  -1.069234
H     -3.908052   0.380700  -0.831035
C      1.447084   0.846775  -0.487693
C      1.962585   1.937076   0.255593
H      1.479432   2.234871   1.200209
C      3.082433   2.650103  -0.192665
H      3.465009   3.498289   0.394924
C      3.723805   2.272931  -1.391132
C      3.236620   1.177448  -2.127785
H      3.737287   0.872912  -3.059135
C      2.107597   0.471667  -1.678025
H      1.734441  -0.372934  -2.277147
C     -1.241154   1.223076   0.412982
C     -2.152853   1.204636   1.489073
H     -2.091908   0.409036   2.245045
C     -3.147633   2.189348   1.606596
H     -3.854402   2.157762   2.449007
C     -3.239395   3.213799   0.645989
C     -2.330081   3.249028  -0.430257
H     -2.397047   4.046966  -1.185237
C     -1.338007   2.263208  -0.540264
H     -0.627229   2.316138  -1.379175
C     -0.289781  -3.546587   0.025743
H     -1.147733  -3.490492   0.725038
C      2.154948  -3.003169   0.107296
H      2.557155  -4.002191  -0.166158
H      1.954528  -2.468000  -0.847835
C      3.172358  -2.217786   0.893056
C      3.541942  -0.506890   2.434652
H      3.104453   0.226624   3.126908
C      4.929781  -0.610345   2.286226
H      5.584855   0.059136   2.863221
C      5.454343  -1.574263   1.409524
C      4.553303  -2.388533   0.705715
H      4.911523  -3.157801   0.004864
C     -0.138991  -5.013005  -0.411018
H     -1.086381  -5.382735  -0.850775
H      0.661523  -5.151999  -1.165774
H      0.088680  -5.654403   0.465179
C      0.626425   0.012291   3.201082
C     -0.978117  -1.796607   2.613325
H      0.990945  -3.770098   1.609436
H      1.035712  -2.315518   3.269205
H      6.540556  -1.691018   1.276586
C     -4.271352   4.309675   0.792140
F     -3.756345   5.405247   1.418167
F     -4.735047   4.734115  -0.412684
F     -5.344453   3.910962   1.521118
C      4.897096   3.074185  -1.904336
F      4.494153   4.127935  -2.671218
F      5.641576   3.594906  -0.893715
F      5.729506   2.328890  -2.677028
```

```
---------------------------------------
Mn25/vi_R
Frequencies, energies and thermodynamic properties:
 Lowest Vibrational Mode (1/cm) =                        11.4762
 2nd Lowest Vibrational Mode (1/cm) =                    11.9140
 E(RB-P86) (a.u.) =                                   -5348.89538893
 Thermal correction to Enthalpy (a.u.) =                 0.731607
 Thermal correction to Gibbs Free Energy (a.u.) =        0.584182
 Total Entropy (cal/Kmol) =                              310.283
 E(RPBE1PBE) (a.u.) =                                 -5348.38805048
Optimised cartesian coordinates (Angstrom):
 Fe1.397852  -2.318511   2.661386
 Mn-1.374866   0.169915  -0.343693
 P      0.773754   0.090494   0.099492
 O     -1.083621   0.346027  -3.288016
 O     -1.714745  -2.734484  -0.673892
 N     -1.887651   0.229292   1.548262
 N     -1.669699   2.199410  -0.145792
 C      1.074975  -0.480575   1.816465
 C      0.020223  -0.791403   2.780038
 C      0.661089  -0.999739   4.056461
 H      0.154441  -1.270767   4.991299
 C      2.081237  -0.834994   3.899025
 H      2.836071  -0.954604   4.687803
 C      2.344916  -0.523254   2.521559
 H      3.335281  -0.339202   2.085981
 C      0.863582  -3.854234   1.393639
 H      0.218011  -3.762151   0.510962
 C      0.417835  -4.130229   2.733229
 H     -0.622771  -4.286034   3.047987
 C      1.572369  -4.140288   3.594780
 H      1.565686  -4.300193   4.681263
 C      2.733955  -3.872925   2.785394
 H      3.767921  -3.795267   3.147295
 C      2.296542  -3.693136   1.425291
 H      2.938700  -3.460345   0.566042
 C      1.548930   1.782595   0.058051
 C      1.784345   2.382375  -1.202178
 H      1.575711   1.823282  -2.128337
 C      2.285087   3.687880  -1.290929
 H      2.467065   4.139979  -2.277534
 C      2.545513   4.422646  -0.115238
 C      2.298797   3.843095   1.142250
 H      2.494304   4.415229   2.061243
 C      1.803838   2.530282   1.227268
 H      1.630019   2.086543   2.219056
 C      1.981966  -0.866514  -0.948270
 C      1.534857  -1.893584  -1.805194
 H      0.464347  -2.126686  -1.882099
 C      2.448130  -2.637761  -2.570093
 H      2.083972  -3.438652  -3.230393
 C      3.824946  -2.359018  -2.489346
 C      4.284063  -1.330904  -1.642492
 H      5.359405  -1.108018  -1.575025
 C      3.367498  -0.589182  -0.882991
 H      3.743826   0.221844  -0.241277
 C     -1.480953  -0.854266   2.474948
 H     -1.669387  -1.794734   1.919546
 C     -1.853219   1.551510   2.171448
 H     -2.673865   1.699056   2.912922
 H     -0.905783   1.708698   2.750118
 C     -1.937363   2.619208   1.123763
 C     -1.691909   3.120124  -1.149034
 H     -1.470951   2.747532  -2.159349
 C     -1.985164   4.468030  -0.931519
 H     -1.990974   5.165312  -1.781931
 C     -2.272975   4.901259   0.376778
 C     -2.250400   3.960634   1.412995
 H     -2.470289   4.251160   2.451339
 C     -2.329444  -0.921017   3.759459
 H     -2.102775  -1.849633   4.321067
 H     -2.144940  -0.064548   4.439739
 H     -3.407267  -0.940478   3.500565
 C     -1.201516   0.278683  -2.113218
 C     -1.529902  -1.577296  -0.506705
 H     -3.508166   0.048937   1.327962
 H     -3.526470   0.089943  -0.725260
 C     -4.592180   0.106980  -0.286094
 C     -5.356199  -1.017898  -0.971190
 C     -5.300306   1.378609  -0.834261
 C     -6.070365  -0.522078  -2.084381
 C     -5.412801  -2.375722  -0.622471
 C     -5.846359   0.974544  -2.225641
 H     -6.142035   1.597518  -0.141065
 C     -6.844664  -1.392632  -2.869814
```

```
 C     -6.191938  -3.247735  -1.408620
 H     -4.862111  -2.745035   0.257384
 H     -6.763228   1.526576  -2.521005
 C     -6.900967  -2.759030  -2.525370
 H     -7.411334  -1.015158  -3.737282
 H     -6.253868  -4.316704  -1.147423
 H     -7.511558  -3.449825  -3.129264
 O     -4.513533   0.018628   1.102737
 H     -4.638605   2.267742  -0.853649
 H     -5.089170   1.173682  -3.018841
 H     -2.512939   5.955839   0.581353
 C      3.133529   5.812356  -0.214432
 F      4.488338   5.776105  -0.358175
 F      2.648477   6.493219  -1.285634
 F      2.877213   6.558797   0.889751
 C      4.808361  -3.119774  -3.352069
 F      5.033324  -2.483153  -4.535167
 F      6.016214  -3.254947  -2.746192
 F      4.364635  -4.365670  -3.655580
---------------------------------------
Mn25/vi_S
Frequencies, energies and thermodynamic properties:
 Lowest Vibrational Mode (1/cm) =                          12.0645
 2nd Lowest Vibrational Mode (1/cm) =                      12.6836
 E(RB-P86) (a.u.) =                                      -5348.89564171
 Thermal correction to Enthalpy (a.u.) =                   0.731687
 Thermal correction to Gibbs Free Energy (a.u.) =          0.584487
 Total Entropy (cal/Kmol) =                                309.810
 E(RPBE1PBE) (a.u.) =                                    -5348.38837600
Optimised cartesian coordinates (Angstrom):
 Fe-2.519141  -1.972994  -2.344263
 Mn1.174702  -0.663086   0.398989
 P     -0.779730   0.199457  -0.103869
 O      1.072317   0.005324   3.284734
 O      0.153443  -3.328774   1.108402
 N      1.599596  -1.084102  -1.469063
 N      2.380067   0.951094  -0.040295
 C     -1.364862  -0.391860  -1.738549
 C     -0.600463  -1.273144  -2.619757
 C     -1.307479  -1.331276  -3.876783
 H     -1.011178  -1.923237  -4.751952
 C     -2.490038  -0.517567  -3.786238
 H     -3.241982  -0.382150  -4.575214
 C     -2.537340   0.057186  -2.470292
 H     -3.320109   0.727512  -2.092799
 C     -2.692205  -3.417134  -0.882756
 H     -2.034845  -3.528305  -0.011112
 C     -2.488482  -4.026805  -2.169635
 H     -1.653233  -4.683457  -2.447830
 C     -3.557580  -3.604728  -3.038003
 H     -3.677356  -3.878778  -4.094699
 C     -4.425360  -2.735234  -2.285291
 H     -5.323103  -2.231568  -2.668008
 C     -3.889564  -2.616133  -0.954107
 H     -4.310614  -2.011480  -0.140462
 C     -0.684464   2.047076  -0.312693
 C     -0.554026   2.845092   0.849273
 H     -0.576140   2.377430   1.846624
 C     -0.397478   4.233513   0.749594
 H     -0.301005   4.841601   1.661718
 C     -0.354764   4.847283  -0.520107
 C     -0.465072   4.062509  -1.681631
 H     -0.425445   4.536479  -2.673451
 C     -0.629099   2.670101  -1.577259
 H     -0.728862   2.072126  -2.495409
 C     -2.260005   0.058398   1.020008
 C     -2.316769  -0.941859   2.012855
 H     -1.477197  -1.636257   2.151531
 C     -3.446743  -1.070792   2.836904
 H     -3.477651  -1.859289   3.603362
 C     -4.536097  -0.193939   2.679680
 C     -4.488980   0.814045   1.696685
 H     -5.337288   1.503014   1.569266
 C     -3.357026   0.939863   0.877917
 H     -3.331793   1.744562   0.127482
 C      0.716828  -1.972126  -2.262962
 H      0.474809  -2.817442  -1.587878
 C      2.146990   0.021310  -2.254541
 H      2.908850  -0.313771  -2.997461
 H      1.351584   0.527504  -2.861608
 C      2.758820   1.044172  -1.346897
 C      2.870295   1.865111   0.841621
 H      2.546956   1.758249   1.886817
 C      3.742755   2.887797   0.463380
 H      4.107367   3.596666   1.220970
```

```
 C      4.140532   2.981857  -0.883586
 C      3.643239   2.043086  -1.795178
 H      3.927929   2.074033  -2.857650
 C      1.404213  -2.578965  -3.501411
 H      0.765987  -3.368518  -3.946768
 H      1.606786  -1.826736  -4.291029
 H      2.364107  -3.049761  -3.207616
 C      1.118536  -0.259770   2.133216
 C      0.511276  -2.245956   0.787776
 H      2.986239  -1.932987  -1.185795
 H      3.092063  -1.627829   0.846963
 C      3.964323  -2.273250   0.457957
 C      3.884136  -3.641125   1.194819
 C      4.601920  -3.415869   2.548239
 O      3.902312  -2.331062  -0.933410
 H      4.830411   3.773456  -1.213760
 C      5.238648  -1.666225   1.025476
 C      5.599168  -2.314498   2.227882
 C      6.016028  -0.619386   0.507164
 C      6.744980  -1.906919   2.931482
 C      7.167169  -0.213802   1.211601
 H      5.732652  -0.137192  -0.442422
 C      7.526669  -0.851885   2.416603
 H      7.039605  -2.410098   3.867476
 H      7.794815   0.601608   0.816834
 H      8.432327  -0.529708   2.955749
 H      5.080220  -4.330990   2.956152
 H      3.881585  -3.065159   3.323032
 H      4.454661  -4.368521   0.576327
 H      2.846750  -4.017393   1.292457
 C     -0.235834   6.351672  -0.617821
 F     -1.439209   6.963779  -0.431805
 F      0.603834   6.855216   0.324839
 F      0.226237   6.754656  -1.828285
 C     -5.731775  -0.294189   3.601825
 F     -6.879050   0.098485   2.990787
 F     -5.924248  -1.559887   4.052084
 F     -5.583580   0.495843   4.701632
---------------------------------------
Mn25/viii
Frequencies, energies and thermodynamic properties:
 Lowest Vibrational Mode (1/cm) =                         10.0944
 2nd Lowest Vibrational Mode (1/cm) =                     13.3828
 E(RB-P86) (a.u.) =                                     -4926.14943077
 Thermal correction to Enthalpy (a.u.) =                  0.574267
 Thermal correction to Gibbs Free Energy (a.u.) =         0.448795
 Total Entropy (cal/Kmol) =                               264.077
 E(RPBE1PBE) (a.u.) =                                   -4925.64759651
Optimised cartesian coordinates (Angstrom):
 Fe-2.435879  -1.850062  -1.665175
 Mn0.612383  -1.380493   2.110168
 P      0.036055  -0.183260   0.292534
 O      0.566108   1.018082   3.852504
 O     -2.143078  -2.022563   2.924895
 N      0.840613  -3.018338   0.900025
 N      2.648901  -1.275154   1.741837
 C     -0.574712  -1.194685  -1.104101
 C     -0.619661  -2.653833  -1.095549
 C     -0.969015  -3.069164  -2.432904
 H     -1.106710  -4.106195  -2.764901
 C     -1.150700  -1.902727  -3.256372
 H     -1.442694  -1.897434  -4.315299
 C     -0.918916  -0.741588  -2.441689
 H     -0.979369   0.300708  -2.780158
 C     -3.809577  -1.692951  -0.134603
 H     -3.572416  -1.569018   0.929613
 C     -3.960307  -2.949338  -0.818162
 H     -3.860702  -3.945601  -0.366792
 C     -4.238193  -2.672480  -2.204693
 H     -4.384139  -3.420275  -2.995688
 C     -4.263139  -1.241921  -2.376609
 H     -4.433118  -0.708978  -3.321765
 C     -3.994879  -0.635643  -1.098294
 H     -3.930173   0.441108  -0.895083
 C      1.475342   0.750525  -0.435967
 C      1.977934   1.865006   0.276619
 H      1.488307   2.192820   1.207464
 C      3.096458   2.568938  -0.189672
 H      3.474418   3.435269   0.373645
 C      3.740115   2.159711  -1.375540
 C      3.259419   1.043111  -2.083636
 H      3.762862   0.715845  -3.005529
 C      2.134031   0.343670  -1.615085
 H      1.764498  -0.521817  -2.185362
 C     -1.207084   1.204889   0.433868
```

```
 C     -2.129911   1.241142   1.500235
 H     -2.111323   0.466759   2.278635
 C     -3.091967   2.260897   1.584757
 H     -3.806382   2.270803   2.421258
 C     -3.142136   3.264774   0.600141
 C     -2.222867   3.244826  -0.467114
 H     -2.256344   4.026204  -1.241063
 C     -1.262038   2.225812  -0.543819
 H     -0.542548   2.239033  -1.376453
 C     -0.285843  -3.531048   0.121323
 H     -1.167297  -3.498982   0.795830
 C      2.089865  -3.033626   0.172596
 H      2.507386  -4.063845   0.027840
 H      2.014876  -2.624891  -0.880368
 C      3.129006  -2.199241   0.862935
 C      3.530277  -0.455377   2.373426
 C      4.910435  -0.522860   2.165385
 C      5.414218  -1.481099   1.265862
 C      4.509591  -2.326080   0.612492
 C     -0.111440  -5.010353  -0.290315
 H     -1.053502  -5.421762  -0.708180
 H      0.680606  -5.146681  -1.055916
 H      0.161067  -5.612266   0.600509
 C      0.593573   0.047598   3.178430
 C     -1.059004  -1.746628   2.543090
 H      1.083166  -2.790685   3.066029
 H      1.173543  -2.195475   3.618354
 H      3.102442   0.274741   3.075826
 H      5.576013   0.164565   2.707653
 H      4.857537  -3.092589  -0.096512
 H      6.496204  -1.567476   1.081822
 C      4.912623   2.951914  -1.908374
 F      5.743223   2.191129  -2.666368
 F      4.504634   3.987948  -2.694602
 F      5.657509   3.493071  -0.909508
 C     -4.141076   4.395049   0.716131
 F     -5.267024   4.016011   1.372533
 F     -3.624281   5.452805   1.401905
 F     -4.519299   4.866362  -0.499967
---------------------------------------
Mn25/ix
Frequencies, energies and thermodynamic properties:
 Lowest Vibrational Mode (1/cm) =                         10.0837
 2nd Lowest Vibrational Mode (1/cm) =                     14.4817
 E(RB-P86) (a.u.) =                                     -5079.93773270
 Thermal correction to Enthalpy (a.u.) =                  0.643699
 Thermal correction to Gibbs Free Energy (a.u.) =         0.509938
 Total Entropy (cal/Kmol) =                               281.523
 E(RPBE1PBE) (a.u.) =                                   -5079.43935527
Optimised cartesian coordinates (Angstrom):
 Fe-2.436189  -1.448982  -2.174347
 Mn0.821115  -1.544836   1.443724
 P     -0.027767   0.000276   0.043046
 O      0.710035   0.267993   3.758860
 O     -1.784731  -2.669821   2.213446
 N      1.120684  -2.854404  -0.212624
 N      2.798870  -1.099091   0.955857
 C     -0.627026  -0.714467  -1.545743
 C     -0.511018  -2.122565  -1.929741
 C     -0.912701  -2.222498  -3.313673
 H     -0.958374  -3.146160  -3.904023
 C     -1.285684  -0.917170  -3.785314
 H     -1.660500  -0.677814  -4.789394
 C     -1.119842   0.012057  -2.703119
 H     -1.323629   1.089230  -2.749741
 C     -3.691039  -1.865245  -0.593488
 H     -3.382983  -1.987158   0.452833
 C     -3.755513  -2.913839  -1.576761
 H     -3.509568  -3.971237  -1.410250
 C     -4.174888  -2.330289  -2.825126
 H     -4.298516  -2.862989  -3.777482
 C     -4.372701  -0.919314  -2.612407
 H     -4.674777  -0.188626  -3.374569
 C     -4.071509  -0.630598  -1.234390
 H     -4.110194   0.357874  -0.758775
 C      1.216980   1.272071  -0.520222
 C      1.645284   2.240119   0.419978
 H      1.191239   2.273527   1.423416
 C      2.644338   3.166523   0.093264
 H      2.962814   3.914823   0.834590
 C      3.246972   3.131434  -1.181556
 C      2.842648   2.163567  -2.118940
 H      3.313626   2.127316  -3.112595
 C      1.834093   1.241850  -1.788822
 H      1.521336   0.501741  -2.540839
```

```
 C     -1.415269   1.133557   0.575288
 C     -2.234790   0.793393   1.671786
 H     -2.050801  -0.130911   2.235373
 C     -3.300646   1.622605   2.058922
 H     -3.932468   1.339003   2.913540
 C     -3.559347   2.811639   1.352842
 C     -2.743426   3.169061   0.260932
 H     -2.939214   4.097787  -0.295936
 C     -1.679035   2.338244  -0.118208
 H     -1.042868   2.645135  -0.962146
 C     -0.036601  -3.274938  -1.049913
 H     -0.835369  -3.505799  -0.316336
 C      2.344998  -2.531608  -0.963708
 H      2.831529  -3.431757  -1.398806
 H      2.070466  -1.880121  -1.824867
 C      3.324460  -1.783222  -0.095079
 C      3.631972  -0.354056   1.722591
 C      5.007713  -0.262096   1.482880
 C      5.557136  -0.978717   0.406321
 C      4.699926  -1.750421  -0.389842
 C      0.240452  -4.559641  -1.849204
 H     -0.694506  -4.935138  -2.310910
 H      0.979240  -4.403348  -2.661417
 H      0.621926  -5.353516  -1.175165
 C      0.760336  -0.475172   2.837350
 C     -0.756930  -2.190908   1.871633
 H      1.343420  -3.563285   0.535178
 H      5.085293  -2.329904  -1.242372
 H      5.633815   0.357860   2.141201
 H      3.172161   0.179814   2.566946
 H      6.636378  -0.939740   0.193107
 O      1.737722  -3.187596   2.212312
 C      1.415192  -3.814201   3.418596
 C      2.032404  -3.148875   4.659917
 H      1.788849  -4.872804   3.379055
 H      0.306429  -3.903779   3.581963
 H      1.800409  -3.726768   5.581750
 H      3.137823  -3.088225   4.561011
 H      1.646621  -2.117363   4.796500
 C      4.287259   4.163885  -1.550540
 F      3.716846   5.309968  -2.019944
 F      5.050962   4.520717  -0.484737
 F      5.127952   3.721849  -2.520909
 C     -4.672149   3.736662   1.793645
 F     -4.212681   4.715750   2.622325
 F     -5.258012   4.362984   0.739876
 F     -5.648785   3.078903   2.468159
---------------------------------------
Mn25/x
Frequencies, energies and thermodynamic properties:
 Lowest Vibrational Mode (1/cm) =                         8.0650
 2nd Lowest Vibrational Mode (1/cm) =                     13.3797
 E(RB-P86) (a.u.) =                                      -5234.87895167
 Thermal correction to Enthalpy (a.u.) =                  0.727840
 Thermal correction to Gibbs Free Energy (a.u.) =         0.580372
 Total Entropy (cal/Kmol) =                               310.371
 E(RPBE1PBE) (a.u.) =                                    -5234.38872378
Optimised cartesian coordinates (Angstrom):
 Fe-1.874709  -2.168888  -2.274635
 Mn1.321196  -0.768437   1.160675
 P     -0.347076   0.164671  -0.041311
 O      0.369970   0.646689   3.558454
 O     -0.168072  -3.182594   1.940775
 N      2.099436  -1.647956  -0.645821
 N      2.718367   0.666588   0.600905
 C     -0.658113  -0.637930  -1.663264
 C      0.132615  -1.746596  -2.196313
 C     -0.289015  -1.953216  -3.562490
 H      0.094153  -2.723453  -4.243262
 C     -1.327668  -1.010425  -3.875681
 H     -1.866693  -0.939973  -4.829830
 C     -1.565253  -0.203720  -2.711509
 H     -2.300531   0.607269  -2.634161
 C     -2.560190  -3.294369  -0.690178
 H     -2.125030  -3.314244   0.317292
 C     -2.180816  -4.147298  -1.785068
 H     -1.410808  -4.929945  -1.757089
 C     -2.970783  -3.779430  -2.931934
 H     -2.903551  -4.227249  -3.932409
 C     -3.842144  -2.699928  -2.544419
 H     -4.556313  -2.181428  -3.197972
 C     -3.587316  -2.397576  -1.160159
 H     -4.078019  -1.612966  -0.570150
 C     -0.006192   1.938794  -0.510627
 C     -0.043027   2.916782   0.512557
```

```
 H     -0.340575   2.635791   1.535381
 C      0.292181   4.250569   0.243959
 H      0.254807   4.998667   1.050120
 C      0.685681   4.629348  -1.056322
 C      0.741094   3.664364  -2.078250
 H      1.052580   3.952655  -3.093256
 C      0.396079   2.329474  -1.805520
 H      0.430337   1.593603  -2.623056
 C     -2.054445   0.351691   0.695541
 C     -2.468290  -0.464755   1.769010
 H     -1.781012  -1.204048   2.201289
 C     -3.764219  -0.354441   2.298879
 H     -4.071161  -1.003054   3.132799
 C     -4.667129   0.581792   1.761474
 C     -4.265322   1.408758   0.694339
 H     -4.966061   2.142654   0.269047
 C     -2.968333   1.295607   0.171868
 H     -2.669046   1.962730  -0.650605
 C      1.204605  -2.538145  -1.461534
 H      0.707150  -3.182589  -0.709547
 C      2.820595  -0.637099  -1.448661
 H      3.624462  -1.083092  -2.073188
 H      2.101925  -0.165210  -2.156304
 C      3.386468   0.440543  -0.561772
 C      3.139048   1.675261   1.401825
 C      4.232953   2.489990   1.086880
 C      4.937030   2.248469  -0.104306
 C      4.505321   1.206099  -0.936704
 C      2.010040  -3.460837  -2.392124
 H      1.348951  -4.229245  -2.840191
 H      2.496366  -2.907780  -3.221542
 H      2.791267  -3.983170  -1.804331
 C      0.767202   0.070928   2.603645
 C      0.387590  -2.195763   1.600583
 H      3.595730  -2.703991   1.282141
 H      2.827240  -2.293591  -0.243430
 O      3.980315  -3.395746   0.582713
 C      5.365560  -3.133835   0.402149
 H      5.647645  -3.446834  -0.630477
 H      5.579492  -2.038080   0.468301
 H      5.026010   0.978708  -1.879003
 H      4.526758   3.295396   1.775834
 H      2.573553   1.829734   2.331826
 C      6.240012  -3.885212   1.409827
 H      6.053043  -4.978185   1.349056
 H      7.319669  -3.707428   1.215803
 H      6.021929  -3.559905   2.449374
 H      5.809782   2.860295  -0.379347
 O      2.984818  -1.618744   2.035574
 C      3.039998  -1.951003   3.404547
 C      3.647152  -0.846482   4.279466
 H      3.666079  -2.874099   3.523959
 H      2.032992  -2.222425   3.812814
 H      3.748188  -1.187996   5.333064
 H      4.655884  -0.564306   3.909791
 H      3.013448   0.064095   4.278359
 C      0.997536   6.077781  -1.356050
 F     -0.130007   6.786892  -1.646453
 F      1.585092   6.700451  -0.300884
 F      1.828827   6.214261  -2.420806
 C     -6.045285   0.740137   2.365178
 F     -6.049301   1.656681   3.373206
 F     -6.956600   1.161488   1.450965
 F     -6.507360  -0.422286   2.892578
---------------------------------------
Mn25/TS-i
Frequencies, energies and thermodynamic properties:
 Lowest Vibrational Mode (1/cm) =-744.0144
 2nd Lowest Vibrational Mode (1/cm) =                     14.5001
 E(RB-P86) (a.u.) =                                      -5081.09076877
 Thermal correction to Enthalpy (a.u.) =                  0.655228
 Thermal correction to Gibbs Free Energy (a.u.) =         0.518057
 Total Entropy (cal/Kmol) =                               288.702
 E(RPBE1PBE) (a.u.) =                                    -5080.59089624
Optimised cartesian coordinates (Angstrom):
 Fe2.022820  -2.004940   2.076887
 Mn-1.095624  -1.103139  -1.564693
 P      0.140297   0.106173  -0.101467
 O     -0.359092   0.681361  -3.801863
 O      1.064153  -2.952329  -2.334244
 N     -1.786063  -2.289438   0.028809
 N     -2.871331  -0.136938  -1.124293
 C      0.505901  -0.772536   1.460996
 C     -0.016307  -2.094758   1.800311
 C      0.322416  -2.338107   3.182679
```

H	0.093233	-3.250922	3.746794
C	1.049989	-1.207746	3.693705
H	1.464317	-1.111379	4.706228
C	1.175422	-0.242704	2.637062
H	1.678807	0.729559	2.713057
C	3.129428	-2.734980	0.497108
H	2.817681	-2.744394	-0.554955
C	2.874564	-3.776561	1.455749
H	2.337539	-4.714780	1.262064
C	3.424022	-3.362222	2.721291
H	3.374571	-3.926405	3.662195
C	4.022668	-2.063925	2.543107
H	4.511120	-1.466499	3.324512
C	3.838265	-1.673891	1.169491
H	4.167943	-0.730023	0.716539
C	-0.722136	1.660830	0.457404
C	-0.879065	2.708806	-0.481110
H	-0.440735	2.620647	-1.487990
C	-1.578621	3.875092	-0.143105
H	-1.679033	4.687099	-0.878892
C	-2.147522	4.007002	1.140367
C	-2.006021	2.968496	2.078442
H	-2.439827	3.070216	3.084283
C	-1.297423	1.803586	1.737569
H	-1.181547	1.009347	2.490204
C	1.785988	0.816485	-0.625237
C	2.501381	0.259335	-1.705859
H	2.085987	-0.584413	-2.272814
C	3.758438	0.766660	-2.073377
H	4.304387	0.317138	-2.915984
C	4.317344	1.844959	-1.363061
C	3.611337	2.414947	-0.285467
H	4.042398	3.257911	0.275047
C	2.354797	1.906177	0.074688
H	1.812477	2.376212	0.908987
C	-0.802212	-3.017872	0.870306
H	-0.083271	-3.462840	0.152133
C	-2.793955	-1.579268	0.823177
H	-3.518689	-2.267403	1.317737
H	-2.321873	-1.002167	1.659500
C	-3.541373	-0.592736	-0.028732
C	-3.465241	0.802791	-1.904908
C	-4.738542	1.315552	-1.637864
C	-5.440543	0.838067	-0.517491
C	-4.830565	-0.128554	0.292535
C	-1.447286	-4.193763	1.631760
H	-0.670556	-4.831724	2.100093
H	-2.133489	-3.854735	2.435048
H	-2.018741	-4.820958	0.918426
C	-0.663958	-0.034630	-2.914266
C	0.230383	-2.193738	-1.982046
H	-2.080569	-1.796852	-2.772875
H	-2.173450	-2.505245	-2.289167
H	-2.386601	-3.198604	-0.740735
O	-2.778702	-3.917682	-1.605177
C	-4.183651	-3.917437	-1.727093
H	-4.679370	-4.036592	-0.727864
H	-4.564984	-2.941748	-2.135787
H	-5.342526	-0.532805	1.178607
H	-5.169478	2.073460	-2.308158
H	-2.893828	1.144368	-2.780111
C	-4.651678	-5.047018	-2.647639
H	-4.322298	-6.031923	-2.253448
H	-5.758777	-5.062078	-2.740671
H	-4.221269	-4.929368	-3.664977
H	-6.448636	1.212017	-0.281587
C	5.646177	2.430146	-1.789024
F	6.338825	2.937044	-0.736755
F	6.439997	1.509037	-2.391698
F	5.482872	3.449691	-2.677679
C	-2.947466	5.243193	1.485147
F	-2.973078	5.481515	2.821241
F	-2.449035	6.355001	0.884396
F	-4.244794	5.131706	1.082027

Mn25/TS-ii_si

Frequencies, energies and thermodynamic properties:

Lowest Vibrational Mode (1/cm) =-260.9126	
2nd Lowest Vibrational Mode (1/cm) =	11.1705
E(RB-P86) (a.u.) =	-5348.89106949
Thermal correction to Enthalpy (a.u.) =	0.728305
Thermal correction to Gibbs Free Energy (a.u.) =	0.584019
Total Entropy (cal/Kmol) =	303.674
E(RPBE1PBE) (a.u.) =	-5348.37806984

Optimised cartesian coordinates (Angstrom):

```
Fe1.380263  -2.238346   2.662381
Mn-1.507543   0.141932  -0.414229
P      0.684820   0.157949   0.104348
O     -1.172051   0.294617  -3.334497
O     -1.825443  -2.771866  -0.660981
N     -2.029299   0.208434   1.622922
N     -1.800544   2.159117  -0.180705
C      1.013778  -0.405257   1.822540
C     -0.028992  -0.748968   2.791179
C      0.623008  -0.943787   4.065927
H      0.129117  -1.230159   5.002745
C      2.036658  -0.742795   3.902241
H      2.796895  -0.848064   4.687693
C      2.284620  -0.418151   2.525641
H      3.267404  -0.208707   2.084361
C      0.871138  -3.780645   1.393997
H      0.215525  -3.698191   0.517622
C      0.443286  -4.071322   2.736749
H     -0.590689  -4.252203   3.059979
C      1.605141  -4.059446   3.588071
H      1.611494  -4.222856   4.674028
C      2.753177  -3.763920   2.769619
H      3.788261  -3.664351   3.122806
C      2.300467  -3.589129   1.414059
H      2.930479  -3.341294   0.550059
C      1.452465   1.856183   0.060316
C      1.626045   2.468002  -1.205194
H      1.385971   1.910000  -2.124639
C      2.104546   3.780684  -1.309528
H      2.237864   4.239111  -2.301089
C      2.404284   4.515339  -0.143114
C      2.218790   3.925935   1.120508
H      2.444853   4.496474   2.033595
C      1.746580   2.605733   1.219509
H      1.622310   2.157643   2.217013
C      1.923324  -0.789466  -0.922251
C      1.494736  -1.849928  -1.747858
H      0.427094  -2.100861  -1.813211
C      2.419817  -2.599788  -2.492190
H      2.069208  -3.422270  -3.133308
C      3.791805  -2.292905  -2.423041
C      4.231518  -1.224711  -1.616772
H      5.300858  -0.969595  -1.572028
C      3.301909  -0.478219  -0.877318
H      3.661954   0.365035  -0.268457
C     -1.526332  -0.876330   2.530914
H     -1.703040  -1.809124   1.959463
C     -1.853588   1.569815   2.172483
H     -2.536567   1.775003   3.024638
H     -0.816471   1.664065   2.567232
C     -2.045904   2.594921   1.089346
C     -1.875525   3.067832  -1.191042
H     -1.669638   2.689041  -2.202307
C     -2.203922   4.412006  -0.984825
H     -2.248451   5.095052  -1.845757
C     -2.478336   4.855203   0.320156
C     -2.398031   3.926578   1.367870
H     -2.601176   4.224311   2.407618
C     -2.347984  -0.971545   3.827924
H     -2.085004  -1.894088   4.382690
H     -2.177492  -0.109881   4.505027
H     -3.428939  -1.022709   3.586572
C     -1.287415   0.235316  -2.156683
C     -1.650083  -1.607080  -0.536269
H     -3.077321   0.071156   1.527467
H     -3.143039   0.070113  -0.664607
C     -4.719309   0.103712  -0.145818
C     -5.103997  -1.142453  -0.901939
C     -5.117140   1.273570  -1.080645
C     -5.406441  -0.816654  -2.242852
C     -5.241358  -2.452516  -0.415576
C     -5.174889   0.662798  -2.497798
H     -6.134422   1.587383  -0.751253
C     -5.848003  -1.819154  -3.122172
C     -5.686813  -3.453528  -1.297247
H     -5.011296  -2.677413   0.638240
H     -5.954593   1.113942  -3.145779
C     -5.985332  -3.137308  -2.640669
H     -6.093340  -1.582595  -4.170711
H     -5.810326  -4.488827  -0.940643
H     -6.337740  -3.930587  -3.319704
O     -4.675918   0.188989   1.115736
H     -4.454161   2.153449  -0.980680
H     -4.202271   0.801072  -3.022784
H     -2.750421   5.903176   0.517610
```

```
 C      2.967574   5.912884  -0.259844
 F      2.434877   6.589800  -1.311179
 F      2.740650   6.653782   0.855015
 F      4.317385   5.898830  -0.452251
 C      4.796633  -3.141127  -3.170171
 F      5.904919  -2.436843  -3.515350
 F      5.221497  -4.193029  -2.413997
 F      4.277485  -3.664968  -4.309780
---------------------------------------
Mn25/TS-ii_re
Frequencies, energies and thermodynamic properties:
 Lowest Vibrational Mode (1/cm) =-262.6659
 2nd Lowest Vibrational Mode (1/cm) =                     8.4665
 E(RB-P86) (a.u.) =                                    -5348.89143983
 Thermal correction to Enthalpy (a.u.) =                 0.728356
 Thermal correction to Gibbs Free Energy (a.u.) =        0.583937
 Total Entropy (cal/Kmol) =                              303.957
 E(RPBE1PBE) (a.u.) =                                  -5348.37963038
Optimised cartesian coordinates (Angstrom):
 Fe-2.700781  -1.764629  -2.297812
 Mn1.187009  -0.894514   0.463430
 P     -0.670580   0.232233  -0.134321
 O      1.132176  -0.142149   3.309150
 O     -0.115425  -3.430127   1.184425
 N      1.542305  -1.446597  -1.535222
 N      2.515905   0.570385  -0.087415
 C     -1.352435  -0.323642  -1.748212
 C     -0.719377  -1.325003  -2.608813
 C     -1.441372  -1.327249  -3.860494
 H     -1.234654  -1.976922  -4.720003
 C     -2.507558  -0.366388  -3.785232
 H     -3.245569  -0.160925  -4.572051
 C     -2.462771   0.249212  -2.488653
 H     -3.148064   1.025100  -2.124264
 C     -4.134761  -2.181737  -0.884448
 H     -4.477619  -1.497884  -0.097287
 C     -3.040188  -3.114897  -0.778865
 H     -2.396287  -3.266408   0.096977
 C     -2.915612  -3.797300  -2.039516
 H     -2.166228  -4.560409  -2.289293
 C     -3.930448  -3.287514  -2.925495
 H     -4.085661  -3.588381  -3.970194
 C     -4.685678  -2.290932  -2.210232
 H     -5.518695  -1.700280  -2.614298
 C     -0.389791   2.055853  -0.399710
 C     -0.134937   2.856750   0.740194
 H     -0.185655   2.413679   1.747852
 C      0.180428   4.215496   0.608203
 H      0.371539   4.824231   1.504826
 C      0.263302   4.797508  -0.674035
 C      0.030667   4.008879  -1.815419
 H      0.100492   4.455847  -2.818268
 C     -0.293567   2.648258  -1.677160
 H     -0.486293   2.051583  -2.581555
 C     -2.159159   0.291217   0.991067
 C     -2.323413  -0.680656   2.000010
 H     -1.564830  -1.462795   2.140818
 C     -3.454219  -0.668286   2.833110
 H     -3.569504  -1.436670   3.611876
 C     -4.437427   0.325126   2.669379
 C     -4.282001   1.307261   1.670971
 H     -5.047073   2.086848   1.537754
 C     -3.149349   1.290282   0.843597
 H     -3.037759   2.075616   0.080526
 C      0.477771  -2.207105  -2.271984
 H      0.149066  -2.979878  -1.549026
 C      2.106085  -0.319963  -2.309233
 H      2.752708  -0.661630  -3.146062
 H      1.268001   0.250040  -2.770951
 C      2.870475   0.606947  -1.404166
 C      3.122678   1.442965   0.760579
 H      2.822526   1.383498   1.816349
 C      4.081335   2.371447   0.341135
 H      4.530559   3.053638   1.077581
 C      4.454691   2.402179  -1.012758
 C      3.841854   1.497879  -1.891963
 H      4.100779   1.480214  -2.961384
 C      1.040403  -2.941173  -3.501119
 H      0.285466  -3.641454  -3.910588
 H      1.333933  -2.247013  -4.314756
 H      1.927425  -3.538287  -3.208087
 C      1.145118  -0.428698   2.160001
 C      0.359815  -2.389193   0.870445
 H      2.344271  -2.124541  -1.376291
 H      2.555780  -1.776283   0.775624
```

```
 C      3.793936  -2.786808   0.327473
 C      4.838503  -1.990801   1.064967
 C      3.385632  -3.912573   1.311285
 C      4.851575  -2.362068   2.428596
 C      5.757939  -1.064099   0.547156
 C      3.765502  -3.385391   2.712142
 C      5.794401  -1.789876   3.298269
 C      6.702745  -0.495166   1.420238
 H      5.737332  -0.807526  -0.524225
 C      6.717457  -0.855013   2.785485
 H      5.823550  -2.071719   4.363769
 H      7.441202   0.228450   1.039216
 H      7.466762  -0.405878   3.457344
 O      3.734997  -2.887202  -0.932605
 H      4.096428  -4.179346   3.413100
 H      2.893765  -2.880602   3.187715
 H      2.330869  -4.225292   1.202743
 H      4.024175  -4.785155   1.041308
 H      5.211731   3.113607  -1.376257
 C      0.553286   6.274386  -0.811657
 F     -0.579130   7.024508  -0.692792
 F      1.411154   6.715847   0.145513
 F      1.098904   6.581151  -2.016538
 C     -5.630607   0.377622   3.597809
 F     -5.401096   1.193496   4.664715
 F     -6.738910   0.855271   2.974278
 F     -5.942889  -0.844102   4.099788
---------------------------------------
Mn25/TS-iii
Frequencies, energies and thermodynamic properties:
 Lowest Vibrational Mode (1/cm) =-619.0055
 2nd Lowest Vibrational Mode (1/cm) =                      13.7055
 E(RB-P86) (a.u.) =                                       -4926.14656172
 Thermal correction to Enthalpy (a.u.) =                   0.573350
 Thermal correction to Gibbs Free Energy (a.u.) =          0.449542
 Total Entropy (cal/Kmol) =                                260.575
 E(RPBE1PBE) (a.u.) =                                     -4925.64162745
Optimised cartesian coordinates (Angstrom):
 Fe-2.407706  -1.797828  -1.679247
 Mn0.633452  -1.444885   2.063817
 P      0.059711  -0.143530   0.300693
 O      0.635445   0.718934   4.080169
 O     -2.123085  -2.090272   2.867468
 N      0.906573  -3.081162   0.796880
 N      2.673654  -1.309411   1.705180
 C     -0.547877  -1.144812  -1.109778
 C     -0.586363  -2.608257  -1.144219
 C     -0.939789  -2.982973  -2.493505
 H     -1.074024  -4.009736  -2.856756
 C     -1.132465  -1.794799  -3.280579
 H     -1.432007  -1.760522  -4.336733
 C     -0.901280  -0.658470  -2.433243
 H     -0.970176   0.393048  -2.739949
 C     -3.781859  -1.669032  -0.145600
 H     -3.545304  -1.553774   0.919539
 C     -3.920137  -2.920269  -0.841540
 H     -3.810301  -3.919845  -0.399971
 C     -4.202541  -2.632497  -2.224723
 H     -4.341308  -3.373641  -3.023224
 C     -4.242064  -1.200607  -2.382187
 H     -4.418104  -0.660109  -3.321908
 C     -3.979036  -0.604213  -1.098167
 H     -3.928442   0.471218  -0.883856
 C      1.481135   0.821521  -0.421292
 C      1.983344   1.916797   0.321239
 H      1.497322   2.214681   1.264233
 C      3.096258   2.639788  -0.129392
 H      3.472847   3.490939   0.457505
 C      3.736492   2.268618  -1.329562
 C      3.257191   1.170647  -2.067572
 H      3.757858   0.872189  -3.000752
 C      2.137384   0.452725  -1.614453
 H      1.770369  -0.398137  -2.208190
 C     -1.202840   1.220874   0.472812
 C     -2.109314   1.221378   1.553736
 H     -2.055669   0.439228   2.323017
 C     -3.095827   2.214951   1.664394
 H     -3.793558   2.201701   2.514788
 C     -3.185466   3.228322   0.692354
 C     -2.273242   3.252399  -0.380952
 H     -2.326274   4.052668  -1.134214
 C     -1.288514   2.258948  -0.484213
 H     -0.573417   2.307080  -1.319377
 C     -0.240565  -3.552457   0.018530
 H     -1.103314  -3.552119   0.716939
```

```
 C      2.149684  -3.011804   0.055573
 H      2.586687  -4.017149  -0.168709
 H      2.028403  -2.525690  -0.954984
 C      3.172282  -2.195524   0.797671
 C      3.542133  -0.507891   2.375093
 C      4.925408  -0.553811   2.175901
 C      5.446577  -1.473225   1.248358
 C      4.555261  -2.303434   0.557163
 C     -0.071726  -5.008240  -0.468390
 H     -1.017172  -5.397546  -0.898856
 H      0.715055  -5.105241  -1.245145
 H      0.205724  -5.654632   0.388825
 C      0.635607  -0.144910   3.274978
 C     -1.038680  -1.815078   2.484613
 H      1.041541  -3.040083   2.392885
 H      1.107145  -2.615640   3.208602
 H      3.103106   0.189082   3.103360
 H      5.578767   0.120330   2.748830
 H      4.917112  -3.041853  -0.174170
 H      6.530854  -1.543395   1.071366
 C     -4.291039   4.257713   0.767149
 F     -4.691063   4.488757   2.043268
 F     -3.915715   5.450068   0.236952
 F     -5.395091   3.853347   0.077136
 C      4.903279   3.079972  -1.845254
 F      5.638635   3.614360  -0.835800
 F      5.744755   2.337934  -2.610318
 F      4.489115   4.122886  -2.619420
---------------------------------------
Mn26/i
Frequencies, energies and thermodynamic properties:
 Lowest Vibrational Mode (1/cm) =                          21.1301
 2nd Lowest Vibrational Mode (1/cm) =                      23.7755
 E(RB-P86) (a.u.) =                                     -4435.75595184
 Thermal correction to Enthalpy (a.u.) =                   0.543090
 Thermal correction to Gibbs Free Energy (a.u.) =          0.430375
 Total Entropy (cal/Kmol) =                                237.230
 E(RPBE1PBE) (a.u.) =                                   -4435.07197598
Optimised cartesian coordinates (Angstrom):
 Fe2.693405  -1.405448   1.210945
 Mn-0.911318  -0.771995  -1.829869
 P     -0.089506   0.365745  -0.165338
 O     -1.439489   1.471269  -3.703904
 O      1.623601  -1.179758  -3.262561
 N     -0.886407  -2.421259  -0.896852
 N     -2.908972  -0.881418  -1.326005
 C      0.760875  -0.753474   1.018844
 C      0.825965  -2.206939   0.873164
 C      1.392377  -2.731340   2.092212
 H      1.597430  -3.789075   2.300884
 C      1.684007  -1.636761   2.979558
 H      2.142057  -1.717029   3.974619
 C      1.305164  -0.415849   2.323012
 H      1.402033   0.592891   2.744598
 C      3.786237  -1.078690  -0.505649
 H      3.369477  -0.876561  -1.500769
 C      4.074410  -2.380923   0.033558
 H      3.918944  -3.340108  -0.478339
 C      4.577690  -2.209088   1.372480
 H      4.868455  -3.013725   2.061006
 C      4.603723  -0.797746   1.660275
 H      4.919887  -0.338080   2.606246
 C      4.112451  -0.098636   0.501233
 H      3.995162   0.988515   0.405946
 C     -1.440118   1.132483   0.860182
 C     -2.078160   2.300364   0.375674
 H     -1.712677   2.788727  -0.541770
 C     -3.173021   2.852831   1.048302
 H     -3.653702   3.767196   0.669930
 C     -3.667286   2.228110   2.223226
 C     -3.047124   1.049007   2.704079
 H     -3.427951   0.561380   3.613767
 C     -1.944469   0.511141   2.024950
 H     -1.464016  -0.396884   2.419327
 C      1.060120   1.818754  -0.373128
 C      1.753034   2.002230  -1.589023
 H      1.602005   1.301438  -2.421850
 C      2.640256   3.074891  -1.756107
 H      3.174691   3.208095  -2.708212
 C      2.843766   3.992425  -0.696437
 C      2.145027   3.822327   0.525008
 H      2.294866   4.536878   1.347781
 C      1.261442   2.746222   0.676434
 H      0.714054   2.641494   1.625521
 C      0.360198  -2.998364  -0.360245
```

```
 H      1.122491  -2.846692  -1.150168
 C     -2.074415  -2.816343  -0.163685
 H     -2.338159  -3.889761  -0.325665
 H     -1.935838  -2.724345   0.946874
 C     -3.237853  -1.962150  -0.561434
 C     -3.908495  -0.037552  -1.709005
 H     -3.610842   0.824745  -2.322551
 C     -5.245373  -0.243211  -1.364195
 H     -6.008681   0.470461  -1.707521
 C     -5.587129  -1.366359  -0.585475
 C     -4.565489  -2.234397  -0.182716
 H     -4.781155  -3.125063   0.426869
 C      0.297192  -4.512749  -0.089790
 H      1.311562  -4.904259   0.126715
 H     -0.351641  -4.770165   0.772092
 H     -0.082915  -5.042781  -0.986813
 C     -1.222219   0.582373  -2.953154
 C      0.634998  -1.004600  -2.632315
 H     -6.632728  -1.558151  -0.300060
 C     -4.791501   2.787818   2.919143
 N     -5.712240   3.246929   3.488460
 C      3.749257   5.096255  -0.860369
 N      4.490203   5.998856  -0.993624
---------------------------------------
Mn26/ii
Frequencies, energies and thermodynamic properties:
 Lowest Vibrational Mode (1/cm) =                          14.3289
 2nd Lowest Vibrational Mode (1/cm) =                      19.8762
 E(RB-P86) (a.u.) =                                       -4590.69011530
 Thermal correction to Enthalpy (a.u.) =                   0.627871
 Thermal correction to Gibbs Free Energy (a.u.) =          0.499589
 Total Entropy (cal/Kmol) =                                269.992
 E(RPBE1PBE) (a.u.) =                                     -4590.01507405
Optimised cartesian coordinates (Angstrom):
 Fe-2.432870  -1.716756  -1.591760
 Mn1.184734  -0.511620   1.266100
 P     -0.333086   0.589275   0.152757
 O      1.122877   1.102921   3.754266
 O     -0.680310  -2.352331   2.599990
 N      1.622683  -1.675321  -0.200116
 N      2.912605   0.414668   0.623483
 C     -0.907334  -0.384118  -1.293656
 C     -0.374670  -1.693140  -1.670827
 C     -0.929311  -2.020918  -2.961678
 H     -0.747419  -2.948050  -3.519930
 C     -1.795156  -0.952051  -3.383131
 H     -2.379091  -0.923292  -4.312876
 C     -1.793509   0.055457  -2.358584
 H     -2.357688   0.996549  -2.386426
 C     -3.215299  -2.398373   0.190465
 H     -2.733481  -2.323173   1.173720
 C     -3.058753  -3.485633  -0.738128
 H     -2.440058  -4.380164  -0.585750
 C     -3.834308  -3.184086  -1.914066
 H     -3.906658  -3.805820  -2.816393
 C     -4.473818  -1.909281  -1.710587
 H     -5.120810  -1.390303  -2.430381
 C     -4.089460  -1.421500  -0.411384
 H     -4.398601  -0.468310   0.036729
 C      0.383128   2.127395  -0.613422
 C      0.627559   3.246220   0.219637
 H      0.319611   3.226124   1.277238
 C      1.256330   4.390802  -0.281796
 H      1.431182   5.258976   0.370715
 C      1.672147   4.429802  -1.638227
 C      1.446941   3.308796  -2.473571
 H      1.767567   3.335374  -3.525537
 C      0.807287   2.171147  -1.960303
 H      0.623539   1.315103  -2.626584
 C     -1.884952   1.274795   0.924572
 C     -2.348950   0.773341   2.159736
 H     -1.788596  -0.011703   2.686013
 C     -3.528489   1.264937   2.736734
 H     -3.880030   0.867240   3.700076
 C     -4.265263   2.280156   2.079214
 C     -3.802610   2.796148   0.842892
 H     -4.367846   3.589464   0.331970
 C     -2.621754   2.296331   0.279675
 H     -2.267968   2.723313  -0.671037
 C      0.598144  -2.535089  -0.834673
 H      0.018536  -2.966889   0.005672
 C      2.639257  -1.210680  -1.134216
 H      3.321385  -2.027611  -1.471163
 H      2.181043  -0.814425  -2.078790
 C      3.448552  -0.113759  -0.514152
```

```
 C      3.577585   1.433030   1.237233
 C      4.781775   1.948163   0.753927
 C      5.339975   1.398510  -0.416348
 C      4.661922   0.352624  -1.053098
 C      1.183657  -3.722049  -1.622024
 H      0.380774  -4.435355  -1.896935
 H      1.691207  -3.410248  -2.557703
 H      1.912268  -4.255142  -0.978201
 C      1.143186   0.462624   2.760410
 C      0.026792  -1.603578   2.015946
 H      2.510482  -2.927837   0.917979
 O      2.930304  -3.697229   1.397039
 C      4.331514  -3.466137   1.476276
 H      4.554760  -2.462287   1.917062
 H      4.806735  -3.476077   0.461872
 H      5.059176  -0.110733  -1.968781
 H      5.275273   2.769161   1.294309
 H      3.119575   1.834796   2.152260
 C      4.971169  -4.548176   2.339008
 H      6.068578  -4.401040   2.411691
 H      4.548784  -4.531762   3.365715
 H      4.783771  -5.554740   1.909099
 H      6.289506   1.780599  -0.821201
 C     -5.473096   2.791992   2.666248
 N     -6.461029   3.211310   3.145294
 C      2.319037   5.600356  -2.161486
 N      2.848416   6.558498  -2.590459
---------------------------------------
Mn26/iii
Frequencies, energies and thermodynamic properties:
 Lowest Vibrational Mode (1/cm) =                        19.1532
 2nd Lowest Vibrational Mode (1/cm) =                    23.8508
 E(RB-P86) (a.u.) =                                     -4591.86234535
 Thermal correction to Enthalpy (a.u.) =                 0.644013
 Thermal correction to Gibbs Free Energy (a.u.) =        0.517150
 Total Entropy (cal/Kmol) =                              267.006
 E(RPBE1PBE) (a.u.) =                                   -4591.18419200
Optimised cartesian coordinates (Angstrom):
 Fe-2.408266  -1.797537  -1.537865
 Mn1.200083  -0.494234   1.473461
 P     -0.300837   0.543132   0.135615
 O      0.697250   1.455418   3.644771
 O     -0.729369  -2.357338   2.691169
 N      1.688600  -1.761315  -0.111352
 N      2.839283   0.504226   0.697153
 C     -0.873243  -0.465567  -1.272962
 C     -0.345945  -1.789735  -1.593203
 C     -0.891139  -2.155051  -2.879016
 H     -0.710607  -3.101125  -3.404672
 C     -1.748508  -1.098325  -3.345520
 H     -2.324926  -1.099684  -4.280371
 C     -1.752145  -0.056424  -2.356442
 H     -2.311363   0.885886  -2.419146
 C     -3.223375  -2.429200   0.249582
 H     -2.759221  -2.332084   1.239046
 C     -3.056596  -3.540870  -0.647265
 H     -2.444567  -4.433638  -0.461662
 C     -3.811126  -3.265971  -1.843280
 H     -3.871204  -3.910036  -2.730740
 C     -4.447856  -1.983407  -1.683311
 H     -5.080364  -1.480355  -2.426900
 C     -4.082553  -1.463904  -0.391200
 H     -4.395336  -0.498741   0.027675
 C      0.393632   2.077714  -0.663573
 C      0.657529   3.198048   0.161345
 H      0.400168   3.171106   1.232079
 C      1.237770   4.357337  -0.365189
 H      1.430707   5.224592   0.283309
 C      1.579156   4.411795  -1.740811
 C      1.329188   3.291974  -2.570461
 H      1.590335   3.330585  -3.638309
 C      0.741633   2.138473  -2.030412
 H      0.541012   1.282761  -2.692251
 C     -1.872223   1.245572   0.864368
 C     -2.389453   0.749330   2.080501
 H     -1.859740  -0.039278   2.631268
 C     -3.587115   1.248633   2.611431
 H     -3.976753   0.853662   3.561085
 C     -4.292691   2.266698   1.925496
 C     -3.779694   2.776386   0.706875
 H     -4.320129   3.571140   0.171959
 C     -2.580202   2.269527   0.191190
 H     -2.190077   2.693499  -0.746371
 C      0.634463  -2.592512  -0.732624
 H      0.057792  -3.019690   0.113847
```

```
 C      2.514748  -1.080401  -1.104729
 H      3.190395  -1.775396  -1.659963
 H      1.904410  -0.582021  -1.908058
 C      3.340451  -0.002944  -0.463991
 C      3.506712   1.517058   1.309040
 C      4.690686   2.059427   0.800302
 C      5.218609   1.534324  -0.392607
 C      4.533167   0.490534  -1.026553
 C      1.214394  -3.794123  -1.510855
 H      0.409676  -4.498214  -1.805956
 H      1.744511  -3.484948  -2.435579
 H      1.926495  -4.339905  -0.859869
 C      0.915918   0.673507   2.788809
 C      0.002013  -1.597824   2.161477
 H      2.388291  -1.001752   2.623570
 H      2.386834  -1.660924   2.118434
 H      2.623199  -2.828204   0.681877
 O      3.145978  -3.482115   1.306966
 C      4.537702  -3.347261   1.077884
 H      4.797938  -3.553766   0.007550
 H      4.891046  -2.303184   1.284061
 H      4.911244   0.046420  -1.959567
 H      5.189290   2.877249   1.340610
 H      3.071808   1.896245   2.245323
 C      5.305365  -4.317773   1.971919
 H      4.997014  -5.364665   1.766096
 H      6.399739  -4.238391   1.803280
 H      5.103905  -4.109373   3.044040
 H      6.152767   1.931592  -0.818469
 C     -5.518935   2.786266   2.465653
 N     -6.521722   3.211626   2.906970
 C      2.175820   5.597507  -2.289940
 N      2.664306   6.567583  -2.739669
----------------------------------------
Mn26/iv
Frequencies, energies and thermodynamic properties:
 Lowest Vibrational Mode (1/cm) =                          18.7263
 2nd Lowest Vibrational Mode (1/cm) =                      20.8920
 E(RB-P86) (a.u.) =                                      -4591.89272173
 Thermal correction to Enthalpy (a.u.) =                   0.648678
 Thermal correction to Gibbs Free Energy (a.u.) =          0.521496
 Total Entropy (cal/Kmol) =                                267.679
 E(RPBE1PBE) (a.u.) =                                    -4591.21242220
Optimised cartesian coordinates (Angstrom):
 Fe-2.539859  -1.602348  -1.606494
 Mn1.256358  -0.601560   1.287594
 P     -0.294759   0.572019   0.127234
 O      1.111972   1.088310   3.690382
 O     -0.528220  -2.557365   2.573076
 N      1.622543  -1.822362  -0.443380
 N      2.902402   0.343618   0.493986
 C     -0.970532  -0.315067  -1.331760
 C     -0.495683  -1.626183  -1.779666
 C     -1.115880  -1.887809  -3.058522
 H     -0.987737  -2.795440  -3.661207
 C     -1.968837  -0.782090  -3.397952
 H     -2.594816  -0.705047  -4.296892
 C     -1.889732   0.184549  -2.339164
 H     -2.427583   1.140621  -2.303280
 C     -3.232908  -2.367803   0.176353
 H     -2.685509  -2.370862   1.127956
 C     -3.178473  -3.398666  -0.826021
 H     -2.586779  -4.322248  -0.770854
 C     -4.016557  -2.996701  -1.926356
 H     -4.169713  -3.556213  -2.858798
 C     -4.592843  -1.717109  -1.602420
 H     -5.264030  -1.130962  -2.244226
 C     -4.107440  -1.326499  -0.304662
 H     -4.350161  -0.393569   0.219871
 C      0.368335   2.150851  -0.609835
 C      0.723515   3.193898   0.281983
 H      0.518627   3.090283   1.359671
 C      1.329208   4.366034  -0.182524
 H      1.588922   5.172336   0.519446
 C      1.611789   4.514221  -1.565438
 C      1.277440   3.470500  -2.462741
 H      1.493365   3.580276  -3.535939
 C      0.662956   2.304632  -1.983329
 H      0.395130   1.513115  -2.699578
 C     -1.815674   1.234905   0.989942
 C     -2.247432   0.650829   2.200323
 H     -1.681165  -0.181244   2.641453
 C     -3.397290   1.114717   2.854657
 H     -3.723256   0.651090   3.797396
 C     -4.138315   2.187874   2.301617
```

```
 C     -3.708900   2.787301   1.091071
 H     -4.277892   3.624745   0.660802
 C     -2.557070   2.312450   0.450691
 H     -2.229478   2.801742  -0.479226
 C      0.463343  -2.560971  -1.052247
 H     -0.066869  -2.992648  -0.179944
 C      2.440419  -1.075589  -1.423353
 H      3.029261  -1.743053  -2.088824
 H      1.753204  -0.499255  -2.083067
 C      3.347716  -0.104051  -0.716001
 C      3.644765   1.283360   1.137212
 C      4.842212   1.793143   0.622506
 C      5.312988   1.315516  -0.611654
 C      4.548742   0.350636  -1.285361
 C      0.932438  -3.732496  -1.931408
 H      0.072289  -4.370332  -2.216377
 H      1.426415  -3.393135  -2.864595
 H      1.643611  -4.366759  -1.364425
 C      1.161767   0.414844   2.716869
 C      0.145666  -1.752511   2.022968
 H      2.371661  -1.459371   2.045162
 H      2.793948  -2.852427   1.751569
 H      2.230035  -2.554426  -0.014739
 O      3.104836  -3.668579   1.253717
 C      4.534663  -3.705982   1.327773
 H      4.867941  -4.447988   0.570197
 H      4.975713  -2.724223   1.030788
 H      4.872761  -0.054378  -2.255991
 H      5.396584   2.551083   1.195357
 H      3.260651   1.624974   2.109102
 C      5.038240  -4.108637   2.713025
 H      4.620290  -5.092161   3.012754
 H      6.146238  -4.183856   2.723280
 H      4.742222  -3.360725   3.479057
 H      6.256453   1.685491  -1.040894
 C     -5.316200   2.671766   2.967371
 N     -6.279976   3.067915   3.511305
 C      2.233462   5.713278  -2.051923
 N      2.742226   6.695481  -2.451064
---------------------------------------
Mn26/v
Frequencies, energies and thermodynamic properties:
 Lowest Vibrational Mode (1/cm) =                          21.1940
 2nd Lowest Vibrational Mode (1/cm) =                      25.1577
 E(RB-P86) (a.u.) =                                     -4436.95481038
 Thermal correction to Enthalpy (a.u.) =                   0.564130
 Thermal correction to Gibbs Free Energy (a.u.) =          0.451266
 Total Entropy (cal/Kmol) =                                237.541
 E(RPBE1PBE) (a.u.) =                                   -4436.27023738
Optimised cartesian coordinates (Angstrom):
 Fe-2.712237  -1.313275  -1.250871
 Mn0.926736  -0.787323   1.935743
 P      0.130190   0.341926   0.145436
 O      1.392165   1.578576   3.617854
 O     -1.584683  -1.307055   3.376420
 N      0.842502  -2.622592   0.823563
 N      2.875068  -0.941242   1.307040
 C     -0.761397  -0.716289  -1.064609
 C     -0.875446  -2.172463  -0.948953
 C     -1.463415  -2.651897  -2.179610
 H     -1.703478  -3.696312  -2.413919
 C     -1.727468  -1.529903  -3.037589
 H     -2.198178  -1.575113  -4.028669
 C     -1.304552  -0.338406  -2.357258
 H     -1.375485   0.683398  -2.751711
 C     -3.778184  -1.004486   0.483982
 H     -3.345670  -0.846020   1.480504
 C     -4.113841  -2.279940  -0.091806
 H     -3.986842  -3.259180   0.389094
 C     -4.624527  -2.050657  -1.418914
 H     -4.946890  -2.824005  -2.128836
 C     -4.608319  -0.631336  -1.662805
 H     -4.917695  -0.132725  -2.591060
 C     -4.083649   0.015731  -0.488757
 H     -3.929896   1.095008  -0.362503
 C      1.458975   1.137266  -0.892009
 C      2.174633   2.219271  -0.319486
 H      1.870306   2.622158   0.659970
 C      3.265700   2.794101  -0.978943
 H      3.803862   3.640922  -0.527606
 C      3.682449   2.280090  -2.234880
 C      2.986514   1.186974  -2.807287
 H      3.304190   0.784572  -3.780814
 C      1.888679   0.626945  -2.138227
 H      1.351785  -0.210771  -2.608865
```

```
 C     -1.011869   1.803552   0.383427
 C     -1.737395   1.932100   1.587486
 H     -1.614699   1.183120   2.382621
 C     -2.618549   3.003882   1.787493
 H     -3.179002   3.092443   2.729912
 C     -2.783035   3.979172   0.773099
 C     -2.051307   3.865544  -0.435614
 H     -2.171134   4.624277  -1.223171
 C     -1.174740   2.788257  -0.619142
 H     -0.602404   2.725869  -1.557327
 C     -0.475067  -3.046596   0.234094
 H     -1.199509  -2.876755   1.055404
 C      1.981117  -2.694112  -0.118907
 H      2.257173  -3.737313  -0.383015
 H      1.666513  -2.198759  -1.064698
 C      3.168590  -1.955879   0.441070
 C      3.908851  -0.194093   1.779142
 H      3.644675   0.616139   2.473718
 C      5.243796  -0.430607   1.431343
 H      6.032099   0.210801   1.852458
 C      5.546607  -1.489155   0.559284
 C      4.485219  -2.260871   0.061097
 H      4.667316  -3.100858  -0.626259
 C     -0.497297  -4.545968  -0.105071
 H     -1.527000  -4.867587  -0.357525
 H      0.155834  -4.797463  -0.965208
 H     -0.171811  -5.144203   0.770755
 C      1.196406   0.634860   2.926807
 C     -0.606451  -1.073793   2.748225
 H      1.031449  -3.265998   1.604609
 H      1.434429  -1.706051   3.119589
 H      6.586022  -1.710925   0.273656
 C      4.802381   2.862112  -2.918313
 N      5.720057   3.339564  -3.477899
 C     -3.682261   5.082459   0.970085
 N     -4.418454   5.984744   1.130366
----------------------------------------
Mn26/vi_R
Frequencies, energies and thermodynamic properties:
 Lowest Vibrational Mode (1/cm) =                        12.6750
 2nd Lowest Vibrational Mode (1/cm) =                    18.2089
 E(RB-P86) (a.u.) =                                     -4859.66487125
 Thermal correction to Enthalpy (a.u.) =                 0.716450
 Thermal correction to Gibbs Free Energy (a.u.) =        0.580870
 Total Entropy (cal/Kmol) =                              285.351
 E(RPBE1PBE) (a.u.) =                                   -4858.97611527
Optimised cartesian coordinates (Angstrom):
 Fe-2.435215  -2.208097  -1.832598
 Mn1.074023   0.241219   0.314940
 P     -1.111933   0.385115   0.235316
 O      1.332248   0.803049   3.214145
 O      1.145652  -2.623231   0.986516
 N      1.249975   0.006435  -1.624883
 N      1.556220   2.175246  -0.209094
 C     -1.774819  -0.341041  -1.311297
 C     -0.945389  -0.895811  -2.380486
 C     -1.820340  -1.166797  -3.495867
 H     -1.518930  -1.607764  -4.454345
 C     -3.164530  -0.803811  -3.135392
 H     -4.055110  -0.917530  -3.767888
 C     -3.146895  -0.302588  -1.789201
 H     -4.019696   0.056847  -1.229367
 C     -1.851314  -3.645497  -0.474251
 H     -1.052894  -3.537421   0.270810
 C     -1.683058  -4.127272  -1.819088
 H     -0.737795  -4.450334  -2.275444
 C     -2.966688  -4.087296  -2.471585
 H     -3.170313  -4.369329  -3.513204
 C     -3.930567  -3.582824  -1.527243
 H     -4.998014  -3.414698  -1.722979
 C     -3.241612  -3.306591  -0.293480
 H     -3.693716  -2.899044   0.619924
 C     -1.674124   2.159220   0.167192
 C     -1.603443   2.930380   1.352696
 H     -1.294638   2.460840   2.300232
 C     -1.925314   4.291692   1.343642
 H     -1.877191   4.879677   2.272046
 C     -2.312800   4.915976   0.129725
 C     -2.370299   4.155801  -1.063453
 H     -2.670487   4.636364  -2.006363
 C     -2.053011   2.789700  -1.038495
 H     -2.119187   2.209140  -1.970780
 C     -2.216456  -0.266847   1.589989
 C     -1.738476  -1.230104   2.504044
 H     -0.703521  -1.591998   2.439681
```

```
 C     -2.571047  -1.743408   3.508666
 H     -2.186322  -2.492893   4.215708
 C     -3.908640  -1.291086   3.615294
 C     -4.395586  -0.317517   2.707777
 H     -5.431622   0.042468   2.790890
 C     -3.551201   0.187644   1.711144
 H     -3.942502   0.957747   1.029270
 C      0.570685  -1.116363  -2.315435
 H      0.752342  -2.001875  -1.673856
 C      1.251135   1.238777  -2.412209
 H      1.937108   1.186887  -3.290595
 H      0.238804   1.450334  -2.845386
 C      1.639247   2.401840  -1.551272
 C      1.861624   3.196200   0.638909
 H      1.785060   2.978810   1.713703
 C      2.261958   4.456779   0.190720
 H      2.499145   5.241927   0.923296
 C      2.358456   4.689587  -1.194375
 C      2.045196   3.644958  -2.071681
 H      2.108119   3.778169  -3.162101
 C      1.166707  -1.442370  -3.698464
 H      0.745811  -2.393040  -4.083263
 H      0.958602  -0.654823  -4.451303
 H      2.264750  -1.571306  -3.616558
 C      1.230440   0.581938   2.057365
 C      1.063311  -1.480154   0.692327
 H      2.853457  -0.360355  -1.642496
 H      3.228256  -0.075713   0.355912
 C      4.198355  -0.257161  -0.241454
 C      4.932492  -1.385592   0.469758
 C      5.130765   0.962340   0.007357
 C      5.877553  -0.861969   1.379547
 C      4.771421  -2.770332   0.311653
 C      5.855177   0.657208   1.341305
 H      5.861871   0.979730  -0.830563
 C      6.667827  -1.729610   2.152192
 C      5.566372  -3.639921   1.084426
 H      4.040428  -3.163968  -0.412534
 H      6.866443   1.109942   1.411637
 C      6.506279  -3.122222   1.999447
 H      7.413242  -1.331801   2.860841
 H      5.458878  -4.730860   0.970291
 H      7.126309  -3.812501   2.594234
 O      3.872611  -0.498880  -1.574528
 H      4.588972   1.929402   0.002763
 H      5.272190   1.051819   2.205309
 H      2.675999   5.670276  -1.580328
 C     -4.767257  -1.810906   4.643900
 N     -5.470056  -2.235780   5.484610
 C     -2.644141   6.313423   0.111563
 N     -2.914981   7.457307   0.096880
---------------------------------------
Mn26/vi_S
Frequencies, energies and thermodynamic properties:
 Lowest Vibrational Mode (1/cm) =                        14.2477
 2nd Lowest Vibrational Mode (1/cm) =                    16.6963
 E(RB-P86) (a.u.) =                                     -4859.66512057
 Thermal correction to Enthalpy (a.u.) =                 0.716546
 Thermal correction to Gibbs Free Energy (a.u.) =        0.580748
 Total Entropy (cal/Kmol) =                              285.810
 E(RPBE1PBE) (a.u.) =                                   -4858.97659013
Optimised cartesian coordinates (Angstrom):
 Fe-3.182528  -1.715570  -1.577697
 Mn0.930549  -0.342037   0.447227
 P     -1.050205   0.553208   0.170028
 O      1.310135   0.568996   3.242879
 O     -0.049646  -2.889152   1.546094
 N      1.047663  -0.943157  -1.414213
 N      2.101403   1.172616  -0.317103
 C     -1.900150  -0.143928  -1.296502
 C     -1.307547  -1.130441  -2.198849
 C     -2.199382  -1.261063  -3.325931
 H     -2.059644  -1.937480  -4.178545
 C     -3.327294  -0.389483  -3.133782
 H     -4.186217  -0.287387  -3.810510
 C     -3.155330   0.295549  -1.882760
 H     -3.849003   1.030778  -1.455326
 C     -3.186353  -3.014702   0.023947
 H     -2.416557  -3.075349   0.803696
 C     -3.183397  -3.745099  -1.215177
 H     -2.414055  -4.457027  -1.542913
 C     -4.352745  -3.360166  -1.963143
 H     -4.627900  -3.722620  -2.962605
 C     -5.081122  -2.392121  -1.183603
 H     -6.009827  -1.889066  -1.484364
```

```
 C     -4.359365  -2.175567   0.043295
 H     -4.646470  -1.484879   0.846615
 C     -0.927667   2.371396  -0.212868
 C     -0.609348   3.259133   0.843506
 H     -0.514665   2.881423   1.874111
 C     -0.414767   4.622986   0.600855
 H     -0.177862   5.306089   1.429863
 C     -0.521433   5.125413  -0.721902
 C     -0.823848   4.241976  -1.786287
 H     -0.907714   4.628296  -2.812867
 C     -1.024469   2.878009  -1.527720
 H     -1.275593   2.207497  -2.363168
 C     -2.342857   0.577224   1.514671
 C     -2.262642  -0.318342   2.602433
 H     -1.425558  -1.025169   2.679484
 C     -3.244731  -0.324127   3.603100
 H     -3.170247  -1.026255   4.446440
 C     -4.331871   0.580229   3.529091
 C     -4.416898   1.489643   2.445330
 H     -5.255522   2.199068   2.387303
 C     -3.426859   1.485062   1.454778
 H     -3.500573   2.212464   0.632156
 C      0.025542  -1.856040  -1.980406
 H     -0.137139  -2.627157  -1.200714
 C      1.508818   0.058915  -2.374373
 H      2.143915  -0.378458  -3.180525
 H      0.650212   0.541068  -2.910694
 C      2.278633   1.133093  -1.668571
 C      2.745361   2.140494   0.391693
 H      2.583192   2.140310   1.478894
 C      3.577482   3.088440  -0.207394
 H      4.072901   3.846041   0.417067
 C      3.767169   3.045748  -1.601533
 C      3.111274   2.050843  -2.335982
 H      3.231201   1.975500  -3.427200
 C      0.494292  -2.599648  -3.245852
 H     -0.230711  -3.394059  -3.514675
 H      0.598047  -1.929471  -4.123558
 H      1.471621  -3.087827  -3.056751
 C      1.163019   0.208434   2.126843
 C      0.288409  -1.855956   1.076882
 H      2.434550  -1.834909  -1.264249
 H      2.869772  -1.350603   0.686717
 C      3.650918  -2.065611   0.232371
 C      3.654868  -3.353811   1.104330
 C      4.589199  -3.034318   2.297062
 O      3.363590  -2.252324  -1.119308
 H      4.419847   3.775727  -2.104194
 C      5.015645  -1.461634   0.527707
 C      5.549772  -2.010285   1.715033
 C      5.725023  -0.499803  -0.207493
 C      6.805069  -1.587430   2.183589
 C      6.985487  -0.078922   0.261390
 H      5.303744  -0.095513  -1.142185
 C      7.519580  -0.617606   1.450083
 H      7.234950  -2.013779   3.105265
 H      7.561352   0.670092  -0.306060
 H      8.508978  -0.284928   1.803764
 H      5.102942  -3.927079   2.711337
 H      4.014268  -2.583195   3.138555
 H      4.097329  -4.159291   0.477832
 H      2.637472  -3.675176   1.402652
 C     -5.340911   0.581874   4.552460
 N     -6.166275   0.583277   5.389132
 C     -0.322765   6.524268  -0.979936
 N     -0.159853   7.669269  -1.190854
---------------------------------------
Mn26/viii
Frequencies, energies and thermodynamic properties:
 Lowest Vibrational Mode (1/cm) =                       19.3938
 2nd Lowest Vibrational Mode (1/cm) =                   26.2094
 E(RB-P86) (a.u.) =                                    -4436.91904312
 Thermal correction to Enthalpy (a.u.) =                0.559149
 Thermal correction to Gibbs Free Energy (a.u.) =       0.445567
 Total Entropy (cal/Kmol) =                             239.053
 E(RPBE1PBE) (a.u.) =                                  -4436.23595703
Optimised cartesian coordinates (Angstrom):
 Fe-2.737243  -1.282883  -1.246054
 Mn0.895143  -0.842670   1.972454
 P      0.132731   0.277992   0.178491
 O      1.314406   1.655530   3.514097
 O     -1.740463  -1.182646   3.241495
 N      0.800321  -2.568526   0.873427
 N      2.851325  -0.946716   1.295299
 C     -0.767061  -0.757204  -1.029243
```

```
 C     -0.921294  -2.203737  -0.902222
 C     -1.508149  -2.670620  -2.135186
 H     -1.775270  -3.710647  -2.362151
 C     -1.729633  -1.547332  -3.007299
 H     -2.185192  -1.584002  -4.006106
 C     -1.284567  -0.361093  -2.328979
 H     -1.319300   0.658408  -2.733857
 C     -3.836221  -0.887168   0.454469
 H     -3.426122  -0.691356   1.453210
 C     -4.173703  -2.182076  -0.072198
 H     -4.066530  -3.140372   0.453433
 C     -4.651362  -2.004918  -1.419987
 H     -4.968683  -2.804564  -2.102625
 C     -4.612089  -0.597048  -1.724857
 H     -4.896960  -0.135995  -2.680088
 C     -4.105383   0.094365  -0.567801
 H     -3.943303   1.176734  -0.483091
 C      1.510045   1.032694  -0.826084
 C      2.200118   2.145012  -0.286259
 H      1.881691   2.581314   0.673822
 C      3.286808   2.713087  -0.960200
 H      3.810086   3.582540  -0.535641
 C      3.714965   2.162639  -2.195448
 C      3.039050   1.041719  -2.735852
 H      3.367513   0.612315  -3.693904
 C      1.947618   0.486127  -2.051941
 H      1.425323  -0.377247  -2.490916
 C     -0.958438   1.781067   0.397244
 C     -1.689176   1.973434   1.589443
 H     -1.604468   1.254772   2.415403
 C     -2.536103   3.079902   1.745912
 H     -3.098269   3.217264   2.681228
 C     -2.663477   4.024460   0.698815
 C     -1.928509   3.845739  -0.499693
 H     -2.017976   4.579342  -1.314385
 C     -1.084566   2.736849  -0.639015
 H     -0.507939   2.628483  -1.569912
 C     -0.470600  -3.027259   0.314508
 H     -1.230516  -2.872513   1.109158
 C      1.917495  -2.749526  -0.025895
 H      2.224199  -3.821405  -0.142444
 H      1.713907  -2.413041  -1.087292
 C      3.113846  -1.966945   0.431018
 C      3.882357  -0.169250   1.720321
 C      5.203207  -0.374073   1.312990
 C      5.483025  -1.431319   0.426926
 C      4.423927  -2.233471  -0.013447
 C     -0.480448  -4.539518  -0.002749
 H     -1.505665  -4.889352  -0.243180
 H      0.170160  -4.796156  -0.864625
 H     -0.123072  -5.105219   0.881716
 C      1.158493   0.646428   2.919570
 C     -0.711868  -1.030007   2.680648
 H      1.395726  -2.225474   2.951886
 H      1.618507  -1.607417   3.437504
 H      3.628711   0.641106   2.419212
 H      5.997413   0.285011   1.692984
 H      4.595584  -3.073479  -0.703490
 H      6.512618  -1.626449   0.089499
 C      4.829442   2.739518  -2.894167
 N      5.741844   3.211915  -3.465547
 C     -3.527529   5.162443   0.852480
 N     -4.234385   6.093128   0.977344
---------------------------------------
Mn26/ix
Frequencies, energies and thermodynamic properties:
 Lowest Vibrational Mode (1/cm) =                         21.1612
 2nd Lowest Vibrational Mode (1/cm) =                     25.4879
 E(RB-P86) (a.u.) =                                     -4590.70758644
 Thermal correction to Enthalpy (a.u.) =                  0.628528
 Thermal correction to Gibbs Free Energy (a.u.) =         0.506276
 Total Entropy (cal/Kmol) =                               257.301
 E(RPBE1PBE) (a.u.) =                                   -4590.02772988
Optimised cartesian coordinates (Angstrom):
 Fe-2.871057  -1.002757  -1.571638
 Mn1.098753  -1.078406   1.245176
 P     -0.044058   0.456706   0.065154
 O      1.484788   0.737656   3.528762
 O     -1.277599  -2.209647   2.558904
 N      1.036651  -2.387306  -0.437103
 N      2.923901  -0.629940   0.341414
 C     -0.974258  -0.257674  -1.353214
 C     -0.935989  -1.665564  -1.755106
 C     -1.629555  -1.767334  -3.017898
 H     -1.797352  -2.691165  -3.585392
```

```
 C     -2.104640  -0.464436  -3.394805
 H     -2.691806  -0.227704  -4.292049
 C     -1.712611   0.466300  -2.374049
 H     -1.929237   1.542060  -2.374492
 C     -3.758084  -1.403446   0.246528
 H     -3.236125  -1.503268   1.206775
 C     -4.013418  -2.470687  -0.683854
 H     -3.723297  -3.522163  -0.555534
 C     -4.694965  -1.915154  -1.824686
 H     -5.008941  -2.466935  -2.720747
 C     -4.863449  -0.502607  -1.598000
 H     -5.330039   0.210486  -2.290646
 C     -4.282111  -0.185018  -0.319660
 H     -4.234716   0.811874   0.137138
 C      1.046938   1.730063  -0.754384
 C      1.666601   2.702470   0.068823
 H      1.432911   2.744578   1.144727
 C      2.575156   3.623688  -0.462668
 H      3.040944   4.380737   0.185496
 C      2.898487   3.579089  -1.843793
 C      2.296209   2.600747  -2.671947
 H      2.541980   2.562834  -3.743613
 C      1.380539   1.689320  -2.126351
 H      0.909332   0.948343  -2.789620
 C     -1.281921   1.590725   0.889689
 C     -1.835384   1.252855   2.143650
 H     -1.530868   0.328932   2.652919
 C     -2.780677   2.083006   2.762666
 H     -3.202519   1.807169   3.740336
 C     -3.188666   3.282134   2.129390
 C     -2.632697   3.636591   0.874765
 H     -2.940326   4.570358   0.381321
 C     -1.687902   2.797355   0.271082
 H     -1.253900   3.101449  -0.693356
 C     -0.273428  -2.815007  -1.002179
 H     -0.890676  -3.051065  -0.111931
 C      2.066213  -2.059148  -1.437551
 H      2.449427  -2.957414  -1.968917
 H      1.607267  -1.409805  -2.217916
 C      3.209018  -1.308206  -0.802028
 C      3.903439   0.111245   0.914542
 C      5.193971   0.207407   0.381696
 C      5.496174  -0.501159  -0.793490
 C      4.487292  -1.270588  -1.388644
 C     -0.170115  -4.097703  -1.844460
 H     -1.181208  -4.479586  -2.089706
 H      0.370931  -3.936607  -2.798975
 H      0.355124  -4.888732  -1.271440
 C      1.338022  -0.007063   2.619627
 C     -0.348972  -1.729276   2.003990
 H      1.420307  -3.095687   0.242863
 H      4.678762  -1.845160  -2.307480
 H      5.948230   0.824185   0.891951
 H      3.637992   0.638466   1.842415
 H      6.503032  -0.458310  -1.236289
 O      2.162762  -2.714832   1.800822
 C      2.097891  -3.351128   3.043337
 C      2.946697  -2.684474   4.137702
 H      2.466802  -4.404722   2.920128
 H      1.046264  -3.454429   3.426660
 H      2.911456  -3.268367   5.083846
 H      4.008695  -2.611344   3.817921
 H      2.586514  -1.657741   4.355778
 C     -4.155363   4.139257   2.758396
 N     -4.946333   4.840530   3.272341
 C      3.830808   4.519877  -2.398156
 N      4.594276   5.290401  -2.852073
---------------------------------------
Mn26/x
Frequencies, energies and thermodynamic properties:
 Lowest Vibrational Mode (1/cm) =                        13.8451
 2nd Lowest Vibrational Mode (1/cm) =                    20.6976
 E(RB-P86) (a.u.) =                                     -4745.64873612
 Thermal correction to Enthalpy (a.u.) =                 0.712740
 Thermal correction to Gibbs Free Energy (a.u.) =        0.576632
 Total Entropy (cal/Kmol) =                              286.463
 E(RPBE1PBE) (a.u.) =                                   -4744.97699384
Optimised cartesian coordinates (Angstrom):
 Fe-2.595622  -1.927440  -1.542409
 Mn1.226923  -0.314243   1.059340
 P     -0.586876   0.564395   0.048357
 O      0.791629   1.454499   3.370286
 O     -0.207216  -2.501376   2.406710
 N      1.626055  -1.464361  -0.718838
 N      2.572632   0.934361   0.080306
```

```
 C     -1.227348  -0.413713  -1.365255
 C     -0.592464  -1.629459  -1.873505
 C     -1.262900  -1.976898  -3.104917
 H     -1.041842  -2.849394  -3.732100
 C     -2.301984  -1.016277  -3.355857
 H     -3.002251  -1.031728  -4.201550
 C     -2.292109  -0.056812  -2.287420
 H     -2.968458   0.802374  -2.193823
 C     -3.041344  -2.788722   0.276658
 H     -2.444151  -2.698219   1.193046
 C     -2.879837  -3.801249  -0.732490
 H     -2.142144  -4.614753  -0.718590
 C     -3.838622  -3.547581  -1.776850
 H     -3.954762  -4.128792  -2.701298
 C     -4.596382  -2.378502  -1.411116
 H     -5.392866  -1.913744  -2.007398
 C     -4.102743  -1.907149  -0.143552
 H     -4.463561  -1.024925   0.400668
 C     -0.253662   2.236208  -0.712376
 C     -0.039824   3.332084   0.159781
 H     -0.151120   3.202652   1.248098
 C      0.307511   4.592310  -0.338366
 H      0.460929   5.437444   0.348902
 C      0.464074   4.779751  -1.736078
 C      0.265237   3.687479  -2.615050
 H      0.382379   3.827856  -3.699844
 C     -0.090781   2.431705  -2.101728
 H     -0.260126   1.600936  -2.803193
 C     -2.121413   0.968725   1.038049
 C     -2.371031   0.327718   2.270723
 H     -1.654038  -0.399892   2.673560
 C     -3.534272   0.601844   3.003863
 H     -3.714776   0.095178   3.963284
 C     -4.475872   1.536833   2.509337
 C     -4.231982   2.192291   1.276724
 H     -4.956467   2.924207   0.890123
 C     -3.063797   1.908942   0.557928
 H     -2.884607   2.443009  -0.387339
 C      0.562020  -2.390503  -1.237480
 H      0.183425  -2.902883  -0.330409
 C      2.228995  -0.610506  -1.765029
 H      2.880063  -1.183587  -2.459765
 H      1.412941  -0.179276  -2.387899
 C      3.001517   0.522747  -1.142925
 C      3.188677   1.994048   0.657967
 C      4.246165   2.681626   0.050895
 C      4.701798   2.250873  -1.205985
 C      4.069411   1.153156  -1.806677
 C      1.145259  -3.473213  -2.161539
 H      0.382190  -4.247346  -2.377421
 H      1.494015  -3.062263  -3.130975
 H      1.998670  -3.967373  -1.655462
 C      0.982171   0.737199   2.448851
 C      0.322292  -1.607028   1.843968
 H      3.415362  -2.354333   1.037116
 H      2.390384  -2.098817  -0.368408
 O      3.648779  -3.137955   0.370572
 C      4.985170  -2.963877  -0.082081
 H      5.068935  -3.412963  -1.099343
 H      5.233156  -1.879178  -0.195362
 H      4.393798   0.778088  -2.788943
 H      4.705765   3.536924   0.567467
 H      2.816613   2.297017   1.647043
 C      6.007981  -3.626649   0.844704
 H      5.786540  -4.708875   0.958254
 H      7.039067  -3.524414   0.443149
 H      5.988331  -3.167118   1.855850
 H      5.538644   2.759357  -1.708738
 O      2.972935  -1.160653   1.748001
 C      3.251277  -1.334227   3.119648
 C      4.064098  -0.184660   3.729359
 H      3.836113  -2.282551   3.248885
 H      2.318933  -1.477584   3.722929
 H      4.330977  -0.407096   4.785833
 H      5.005617  -0.026714   3.161577
 H      3.492136   0.765962   3.718168
 C     -5.669278   1.825269   3.256158
 N     -6.645689   2.061612   3.866244
 C      0.821843   6.069540  -2.256219
 N      1.115042   7.125576  -2.681932
---------------------------------------
```

Mn26/TS-i

Frequencies, energies and thermodynamic properties:

Lowest Vibrational Mode (1/cm) =-732.7994

2nd Lowest Vibrational Mode (1/cm) =   18.6211

```
 E(RB-P86) (a.u.) =                                        -4591.86026184
 Thermal correction to Enthalpy (a.u.) =                    0.640226
 Thermal correction to Gibbs Free Energy (a.u.) =           0.514788
 Total Entropy (cal/Kmol) =                                 264.006
 E(RPBE1PBE) (a.u.) =                                      -4591.17880676
Optimised cartesian coordinates (Angstrom):
 Fe-2.492924  -1.674979  -1.553385
 Mn1.199135  -0.579316   1.433844
 P     -0.267755   0.550546   0.133780
 O      0.842389   1.329932   3.661469
 O     -0.794552  -2.367504   2.661163
 N      1.613690  -1.852324  -0.187004
 N      2.877942   0.345145   0.654339
 C     -0.901889  -0.411729  -1.285021
 C     -0.436245  -1.751714  -1.637362
 C     -1.011640  -2.070893  -2.922492
 H     -0.877769  -3.013784  -3.467378
 C     -1.828515  -0.970346  -3.358137
 H     -2.416273  -0.930629  -4.284975
 C     -1.774833   0.052322  -2.350854
 H     -2.294622   1.018208  -2.389752
 C     -3.312323  -2.296212   0.235304
 H     -2.833354  -2.229338   1.220249
 C     -3.199178  -3.402358  -0.676810
 H     -2.620440  -4.320523  -0.509086
 C     -3.955851  -3.083628  -1.860406
 H     -4.050856  -3.713775  -2.754760
 C     -4.539936  -1.779313  -1.677809
 H     -5.160152  -1.242670  -2.408114
 C     -4.140037  -1.290337  -0.384006
 H     -4.409675  -0.318753   0.049652
 C      0.485978   2.060695  -0.658252
 C      0.815711   3.156038   0.176839
 H      0.569571   3.128098   1.250218
 C      1.446871   4.291677  -0.342505
 H      1.689709   5.140232   0.313949
 C      1.775384   4.346651  -1.721332
 C      1.461264   3.250804  -2.561128
 H      1.712414   3.289657  -3.631382
 C      0.822295   2.121659  -2.028194
 H      0.570707   1.286531  -2.698870
 C     -1.798615   1.313040   0.887290
 C     -2.324151   0.821053   2.101650
 H     -1.824244   0.000117   2.632877
 C     -3.491867   1.365512   2.654438
 H     -3.888913   0.973379   3.602203
 C     -4.157707   2.425725   1.992965
 C     -3.635481   2.931616   0.776679
 H     -4.145067   3.758752   0.260881
 C     -2.466241   2.379052   0.238821
 H     -2.067367   2.799617  -0.696592
 C      0.507930  -2.623418  -0.809921
 H     -0.073068  -3.031890   0.042262
 C      2.470213  -1.190911  -1.176746
 H      3.100475  -1.908704  -1.751869
 H      1.862380  -0.653286  -1.949562
 C      3.352078  -0.170146  -0.514861
 C      3.598400   1.313637   1.277564
 C      4.809207   1.799573   0.774205
 C      5.309567   1.262521  -0.424658
 C      4.569187   0.264703  -1.071795
 C      1.024455  -3.836571  -1.610255
 H      0.185497  -4.500370  -1.902004
 H      1.556450  -3.539307  -2.537449
 H      1.718396  -4.423990  -0.976023
 C      0.997656   0.564700   2.776588
 C     -0.035163  -1.636561   2.129275
 H      2.367079  -1.218123   2.498871
 H      2.388803  -1.953224   2.044475
 H      2.342884  -2.725917   0.524538
 O      2.871980  -3.391077   1.349452
 C      4.278500  -3.397014   1.230225
 H      4.594452  -3.606950   0.174999
 H      4.720240  -2.394760   1.482452
 H      4.923118  -0.186882  -2.010658
 H      5.349805   2.583351   1.324534
 H      3.185227   1.701989   2.219762
 C      4.898016  -4.449953   2.151373
 H      4.507059  -5.460234   1.905877
 H      6.004569  -4.472041   2.054461
 H      4.648635  -4.239788   3.213237
 H      6.263762   1.614629  -0.845665
 C     -5.353153   2.991117   2.555630
 N     -6.330844   3.453777   3.015370
 C      2.423178   5.508329  -2.263612
```

```
 N      2.953159   6.458800  -2.708270
---------------------------------------
Mn26/TS-ii_si
Frequencies, energies and thermodynamic properties:
 Lowest Vibrational Mode (1/cm) =-271.8079
 2nd Lowest Vibrational Mode (1/cm) =                    12.6448
 E(RB-P86) (a.u.) =                                    -4859.66087699
 Thermal correction to Enthalpy (a.u.) =                 0.713105
 Thermal correction to Gibbs Free Energy (a.u.) =        0.580244
 Total Entropy (cal/Kmol) =                              279.631
 E(RPBE1PBE) (a.u.) =                                  -4858.96639633
Optimised cartesian coordinates (Angstrom):
 Fe-2.479545  -2.073830  -1.847183
 Mn1.225101   0.170169   0.327355
 P     -1.004145   0.443516   0.216790
 O      1.500743   0.675388   3.214744
 O      1.216195  -2.717051   0.891615
 N      1.344409  -0.059278  -1.758941
 N      1.714337   2.087063  -0.219803
 C     -1.733734  -0.239619  -1.323514
 C     -0.950896  -0.825785  -2.413875
 C     -1.859141  -1.048323  -3.515368
 H     -1.598131  -1.494850  -4.482805
 C     -3.177450  -0.629620  -3.125378
 H     -4.084004  -0.702067  -3.740805
 C     -3.109708  -0.138123  -1.778029
 H     -3.953875   0.254193  -1.196764
 C     -1.913471  -3.549448  -0.524548
 H     -1.083103  -3.484220   0.190373
 C     -1.818302  -4.025120  -1.879115
 H     -0.906945  -4.386963  -2.373809
 C     -3.122227  -3.920732  -2.481920
 H     -3.377290  -4.182217  -3.517552
 C     -4.025770  -3.382847  -1.497632
 H     -5.090870  -3.163928  -1.651293
 C     -3.279300  -3.150473  -0.288799
 H     -3.677798  -2.731232   0.643888
 C     -1.529019   2.231390   0.179921
 C     -1.376569   2.989050   1.367780
 H     -1.043338   2.498190   2.296284
 C     -1.649541   4.360768   1.388096
 H     -1.537885   4.934989   2.319768
 C     -2.069871   5.014148   0.200522
 C     -2.209303   4.269106  -0.995856
 H     -2.536297   4.770263  -1.919047
 C     -1.940297   2.892674  -0.998981
 H     -2.072430   2.327817  -1.934155
 C     -2.121352  -0.192359   1.573373
 C     -1.666719  -1.202559   2.448039
 H     -0.650154  -1.605823   2.343286
 C     -2.497103  -1.708640   3.458065
 H     -2.130480  -2.495653   4.133399
 C     -3.810023  -1.200063   3.611779
 C     -4.273593  -0.179381   2.744431
 H     -5.290271   0.223314   2.863710
 C     -3.431013   0.316315   1.741291
 H     -3.803238   1.121605   1.089882
 C      0.540316  -1.145801  -2.412680
 H      0.706401  -2.030124  -1.765854
 C      1.244218   1.245575  -2.447436
 H      1.778169   1.252528  -3.421866
 H      0.172687   1.449631  -2.672539
 C      1.763180   2.342632  -1.559685
 C      2.091277   3.079256   0.632012
 H      2.039188   2.845842   1.704973
 C      2.535219   4.331725   0.194564
 H      2.826665   5.090243   0.935763
 C      2.606896   4.586592  -1.185465
 C      2.215289   3.571498  -2.070011
 H      2.250660   3.722045  -3.159399
 C      1.073592  -1.495315  -3.812539
 H      0.592759  -2.420467  -4.187737
 H      0.884773  -0.691721  -4.553217
 H      2.165509  -1.681429  -3.763702
 C      1.370053   0.477637   2.054417
 C      1.169436  -1.559029   0.649806
 H      2.365565  -0.344137  -1.832301
 H      2.858925  -0.117629   0.283312
 C      4.279630  -0.379184  -0.495678
 C      4.654552  -1.566357   0.355775
 C      4.999719   0.823897   0.165432
 C      5.256332  -1.126274   1.555658
 C      4.531506  -2.932143   0.054117
 C      5.263374   0.391190   1.624083
 H      5.961431   0.936119  -0.386089
```

```
 C      5.739570  -2.066900   2.480283
 C      5.019253  -3.871657   0.980032
 H      4.069610  -3.249164  -0.894624
 H      6.206049   0.797533   2.045500
 C      5.616548  -3.440031   2.184499
 H      6.216678  -1.741686   3.419529
 H      4.942132  -4.949767   0.765612
 H      5.998529  -4.186868   2.899260
 O      4.003189  -0.445783  -1.729915
 H      4.446193   1.775417   0.056178
 H      4.439831   0.736856   2.289691
 H      2.961996   5.556644  -1.565136
 C     -4.666542  -1.710668   4.646515
 N     -5.367712  -2.128078   5.492490
 C     -2.351186   6.422123   0.211753
 N     -2.581433   7.575173   0.221022
---------------------------------------
Mn26/TS-ii_re
Frequencies, energies and thermodynamic properties:
 Lowest Vibrational Mode (1/cm) =-267.3279
 2nd Lowest Vibrational Mode (1/cm) =                     7.4042
 E(RB-P86) (a.u.) =                                      -4859.66138370
 Thermal correction to Enthalpy (a.u.) =                  0.713282
 Thermal correction to Gibbs Free Energy (a.u.) =         0.580105
 Total Entropy (cal/Kmol) =                               280.293
 E(RPBE1PBE) (a.u.) =                                    -4858.96790034
Optimised cartesian coordinates (Angstrom):
 Fe-3.325778  -1.480730  -1.488724
 Mn1.014368  -0.533744   0.456041
 P     -0.903942   0.596074   0.133276
 O      1.492448   0.399408   3.207755
 O     -0.178619  -2.980899   1.567748
 N      0.990569  -1.216409  -1.532476
 N      2.251757   0.852261  -0.420152
 C     -1.882513  -0.041694  -1.283134
 C     -1.428827  -1.105493  -2.181386
 C     -2.367758  -1.163415  -3.277928
 H     -2.330251  -1.866894  -4.118879
 C     -3.390702  -0.175787  -3.069340
 H     -4.258015  -0.000455  -3.719638
 C     -3.103415   0.512614  -1.842497
 H     -3.702243   1.323636  -1.408594
 C     -4.464497  -1.798093   0.194444
 H     -4.629801  -1.074560   1.003009
 C     -3.394466  -2.762678   0.124731
 H     -2.597413  -2.906360   0.865776
 C     -3.537328  -3.494702  -1.105625
 H     -2.871204  -4.290919  -1.464178
 C     -4.692861  -2.984435  -1.797567
 H     -5.057378  -3.317937  -2.778314
 C     -5.267408  -1.937530  -0.992255
 H     -6.147788  -1.334291  -1.251100
 C     -0.644160   2.389599  -0.303065
 C     -0.160957   3.252144   0.712150
 H     -0.034891   2.876725   1.740353
 C      0.156988   4.585676   0.434258
 H      0.520735   5.247823   1.233945
 C      0.012248   5.084785  -0.886264
 C     -0.454048   4.226295  -1.911531
 H     -0.569236   4.608635  -2.936637
 C     -0.777058   2.893918  -1.616181
 H     -1.155064   2.247798  -2.422804
 C     -2.155602   0.775066   1.508902
 C     -2.145195  -0.126066   2.595273
 H     -1.390712  -0.923279   2.642570
 C     -3.090269  -0.023245   3.625703
 H     -3.070975  -0.731556   4.467001
 C     -4.068794   0.999958   3.585081
 C     -4.082840   1.915556   2.503212
 H     -4.836771   2.715953   2.470295
 C     -3.130878   1.799782   1.482203
 H     -3.146720   2.531450   0.660155
 C     -0.200423  -1.993657  -2.014367
 H     -0.400544  -2.714028  -1.196633
 C      1.420305  -0.154542  -2.467514
 H      1.894434  -0.564993  -3.384942
 H      0.521609   0.411259  -2.802909
 C      2.357055   0.799900  -1.779310
 C      3.024354   1.751703   0.245366
 H      2.924303   1.763750   1.339923
 C      3.908077   2.622465  -0.401000
 H      4.501080   3.330814   0.195690
 C      4.024063   2.562326  -1.799611
 C      3.239767   1.629009  -2.492501
 H      3.295659   1.540953  -3.587951
```

```
 C      0.117861  -2.811991  -3.277272
 H     -0.708318  -3.516836  -3.497462
 H      0.266026  -2.174385  -4.172573
 H      1.035815  -3.412104  -3.114496
 C      1.291556   0.039461   2.098077
 C      0.249781  -1.976707   1.104420
 H      1.801458  -1.901837  -1.478897
 H      2.398683  -1.442518   0.573898
 C      3.515991  -2.486298  -0.016777
 C      4.682645  -1.690393   0.510148
 C      3.258657  -3.570079   1.061789
 C      4.911993  -2.015228   1.866486
 C      5.520235  -0.801588  -0.183319
 C      3.871152  -3.004606   2.361340
 C      5.993342  -1.435332   2.549857
 C      6.604179  -0.225056   0.503255
 H      5.330254  -0.579025  -1.245637
 C      6.835824  -0.539105   1.860110
 H      6.191033  -1.681854   3.606142
 H      7.282146   0.468938  -0.019091
 H      7.691964  -0.084360   2.384305
 O      3.254213  -2.635364  -1.247026
 H      4.300504  -3.781712   3.026941
 H      3.097411  -2.466543   2.955275
 H      2.196184  -3.865422   1.139286
 H      3.831436  -4.463488   0.721980
 H      4.715611   3.226284  -2.340225
 C     -5.038429   1.114326   4.639498
 N     -5.831786   1.208371   5.501632
 C      0.337707   6.451669  -1.181735
 N      0.604417   7.570987  -1.423788
---------------------------------------
Mn26/TS-iii
Frequencies, energies and thermodynamic properties:
 Lowest Vibrational Mode (1/cm) =-616.1858
 2nd Lowest Vibrational Mode (1/cm) =                     19.0333
 E(RB-P86) (a.u.) =                                    -4436.91614687
 Thermal correction to Enthalpy (a.u.) =                  0.558170
 Thermal correction to Gibbs Free Energy (a.u.) =         0.445329
 Total Entropy (cal/Kmol) =                               237.493
 E(RPBE1PBE) (a.u.) =                                  -4436.22987169
Optimised cartesian coordinates (Angstrom):
 Fe-2.725790  -1.276318  -1.248477
 Mn0.878524  -0.878170   1.949660
 P      0.136535   0.311737   0.173568
 O      1.361509   1.438483   3.724658
 O     -1.763131  -1.200001   3.207389
 N      0.826743  -2.631446   0.820816
 N      2.846303  -0.963445   1.290901
 C     -0.758646  -0.740259  -1.028708
 C     -0.908839  -2.193056  -0.915916
 C     -1.493253  -2.646915  -2.156074
 H     -1.756181  -3.685047  -2.395368
 C     -1.720508  -1.516436  -3.015459
 H     -2.178355  -1.544848  -4.013361
 C     -1.279114  -0.336942  -2.325066
 H     -1.320062   0.686944  -2.718198
 C     -3.830983  -0.882716   0.448733
 H     -3.424105  -0.678957   1.446998
 C     -4.155480  -2.183345  -0.072527
 H     -4.040562  -3.138122   0.457845
 C     -4.633121  -2.016807  -1.421465
 H     -4.941786  -2.822440  -2.101012
 C     -4.606343  -0.610056  -1.732744
 H     -4.893569  -0.156172  -2.690683
 C     -4.107856   0.091370  -0.578162
 H     -3.955624   1.175541  -0.498329
 C      1.502009   1.079930  -0.836311
 C      2.203866   2.174158  -0.274143
 H      1.893578   2.589091   0.698234
 C      3.290916   2.750851  -0.939788
 H      3.822778   3.605965  -0.497084
 C      3.709305   2.227267  -2.190147
 C      3.022372   1.124162  -2.753251
 H      3.342847   0.715384  -3.723019
 C      1.930921   0.559864  -2.076874
 H      1.401741  -0.290193  -2.533479
 C     -0.965481   1.803984   0.399897
 C     -1.683183   1.984203   1.602006
 H     -1.583857   1.259063   2.420955
 C     -2.533555   3.085381   1.776102
 H     -3.086087   3.213582   2.718473
 C     -2.676187   4.037149   0.737395
 C     -1.952830   3.871450  -0.470129
 H     -2.054178   4.611494  -1.277625
```

```
 C     -1.105862   2.767290  -0.627443
 H     -0.538024   2.668524  -1.564889
 C     -0.459572  -3.062370   0.269427
 H     -1.201292  -2.924448   1.083722
 C      1.940954  -2.734348  -0.099982
 H      2.255674  -3.790093  -0.294748
 H      1.703048  -2.318048  -1.120991
 C      3.127699  -1.961462   0.406467
 C      3.868499  -0.197394   1.753345
 C      5.197127  -0.388717   1.361767
 C      5.494853  -1.422323   0.456172
 C      4.444659  -2.216893  -0.020578
 C     -0.479768  -4.563417  -0.092473
 H     -1.507187  -4.900068  -0.340916
 H      0.168715  -4.797225  -0.962237
 H     -0.125274  -5.157247   0.774242
 C      1.169484   0.513983   3.016195
 C     -0.733172  -1.058388   2.645036
 H      1.208362  -2.468459   2.372928
 H      1.426663  -1.983685   3.124398
 H      3.603516   0.592450   2.470881
 H      5.982584   0.263228   1.771106
 H      4.630516  -3.040898  -0.725870
 H      6.530356  -1.607553   0.131659
 C     -3.543634   5.170064   0.908559
 N     -4.253264   6.096651   1.047539
 C      4.824913   2.812039  -2.880139
 N      5.738366   3.290963  -3.444533
---------------------------------------
Mn27/i
Frequencies, energies and thermodynamic properties:
 Lowest Vibrational Mode (1/cm) =                        19.2284
 2nd Lowest Vibrational Mode (1/cm) =                    21.1774
 E(RB-P86) (a.u.) =                                    -4660.15684309
 Thermal correction to Enthalpy (a.u.) =                 0.551888
 Thermal correction to Gibbs Free Energy (a.u.) =        0.434534
 Total Entropy (cal/Kmol) =                              246.992
 E(RPBE1PBE) (a.u.) =                                  -4659.49948110
Optimised cartesian coordinates (Angstrom):
 Fe-2.537685  -1.742861  -1.410306
 Mn0.780858  -0.981835   1.920175
 P      0.019672   0.147767   0.222644
 O      1.015805   1.237816   3.882094
 O     -1.826635  -1.578523   3.140599
 N      0.925190  -2.599888   0.946945
 N      2.818192  -0.973220   1.593769
 C     -0.670131  -0.981280  -1.049787
 C     -0.655111  -2.439501  -0.946818
 C     -1.091047  -2.959607  -2.219938
 H     -1.212616  -4.020934  -2.471262
 C     -1.381356  -1.857881  -3.098920
 H     -1.755657  -1.935521  -4.128579
 C     -1.132659  -0.636779  -2.383325
 H     -1.260898   0.377121  -2.783839
 C     -3.783769  -1.559597   0.222021
 H     -3.463134  -1.381548   1.256737
 C     -3.949257  -2.848743  -0.394355
 H     -3.778836  -3.820993   0.087186
 C     -4.351830  -2.642172  -1.762051
 H     -4.537071  -3.428316  -2.506134
 C     -4.438884  -1.222645  -1.990722
 H     -4.703960  -0.737229  -2.939491
 C     -4.085480  -0.552808  -0.766225
 H     -4.042984   0.533434  -0.615367
 C      1.411320   1.004007  -0.663181
 C      1.948777   2.189782  -0.101684
 H      1.478543   2.644492   0.784692
 C      3.077078   2.800375  -0.660116
 H      3.497548   3.725906  -0.243998
 C      3.683222   2.204950  -1.781608
 C      3.185542   1.021783  -2.350255
 H      3.686234   0.590091  -3.227614
 C      2.048909   0.424828  -1.785711
 H      1.645972  -0.493706  -2.237967
 C     -1.218541   1.532703   0.369556
 C     -2.032917   1.630311   1.519641
 H     -1.927192   0.902890   2.336292
 C     -2.985911   2.652900   1.638956
 H     -3.625921   2.741918   2.527140
 C     -3.111963   3.581946   0.594956
 C     -2.312624   3.518787  -0.557522
 H     -2.439196   4.269440  -1.349429
 C     -1.364652   2.491635  -0.662175
 H     -0.725427   2.450370  -1.557061
 C     -0.237675  -3.236453   0.300023
```

```
 H     -1.066667  -3.153338   1.030726
 C      2.191956  -2.911973   0.312186
 H      2.497606  -3.974427   0.471468
 H      2.144697  -2.796503  -0.803637
 C      3.268817  -2.011196   0.832226
 C      3.734250  -0.092447   2.086380
 H      3.339528   0.733831   2.694608
 C      5.105005  -0.219268   1.855064
 H      5.796763   0.520624   2.283810
 C      5.571845  -1.298073   1.078803
 C      4.636613  -2.203520   0.564126
 H      4.950868  -3.062251  -0.048292
 C     -0.059248  -4.735771   0.000031
 H     -1.027346  -5.180727  -0.306183
 H      0.669921  -4.928858  -0.812946
 H      0.281093  -5.267555   0.911811
 C      0.916927   0.358865   3.096967
 C     -0.805902  -1.327075   2.593955
 H      6.646879  -1.427560   0.881352
 N      4.875030   2.841790  -2.376441
 N     -4.115712   4.667233   0.714353
 O     -4.804962   4.703799   1.739197
 O     -4.206266   5.472581  -0.218560
 O      5.295340   3.883051  -1.855190
 O      5.390195   2.300676  -3.363576
---------------------------------------
Mn27/ii
Frequencies, energies and thermodynamic properties:
 Lowest Vibrational Mode (1/cm) =                        17.6080
 2nd Lowest Vibrational Mode (1/cm) =                    19.7107
 E(RB-P86) (a.u.) =                                    -4815.09064502
 Thermal correction to Enthalpy (a.u.) =                 0.636650
 Thermal correction to Gibbs Free Energy (a.u.) =        0.503839
 Total Entropy (cal/Kmol) =                              279.525
 E(RPBE1PBE) (a.u.) =                                  -4814.44246874
Optimised cartesian coordinates (Angstrom):
 Fe-2.176518  -1.865044  -1.907641
 Mn1.160268  -0.763246   1.322984
 P     -0.262714   0.367022   0.118650
 O      0.902062   0.771195   3.850151
 O     -0.808003  -2.639252   2.444355
 N      1.722116  -1.892526  -0.121634
 N      2.932725   0.181112   0.848332
 C     -0.685099  -0.552577  -1.412047
 C     -0.122688  -1.853985  -1.773829
 C     -0.541510  -2.135678  -3.125258
 H     -0.306740  -3.045931  -3.691495
 C     -1.354159  -1.047283  -3.598900
 H     -1.838517  -0.984299  -4.582573
 C     -1.454416  -0.072799  -2.547890
 H     -2.006715   0.873930  -2.604589
 C     -3.123670  -2.626335  -0.242093
 H     -2.732955  -2.606972   0.783520
 C     -2.892541  -3.666804  -1.208033
 H     -2.297151  -4.575569  -1.047473
 C     -3.555608  -3.295791  -2.431829
 H     -3.550045  -3.869240  -3.368354
 C     -4.200650  -2.025149  -2.221107
 H     -4.774967  -1.461598  -2.968477
 C     -3.932109  -1.609233  -0.869107
 H     -4.273912  -0.676539  -0.402388
 C      0.511027   1.939656  -0.503178
 C      0.640256   3.029989   0.393255
 H      0.204968   2.970860   1.403425
 C      1.317632   4.194073   0.012884
 H      1.416651   5.051768   0.691905
 C      1.884542   4.256003  -1.272650
 C      1.787572   3.187658  -2.177476
 H      2.242513   3.277278  -3.173321
 C      1.098770   2.029858  -1.786264
 H      1.006070   1.195800  -2.497670
 C     -1.886557   1.012229   0.768868
 C     -2.446447   0.471528   1.947353
 H     -1.922645  -0.320927   2.498924
 C     -3.678449   0.934616   2.433921
 H     -4.125849   0.523972   3.349150
 C     -4.342747   1.949672   1.729686
 C     -3.809902   2.515389   0.560494
 H     -4.358735   3.313348   0.042220
 C     -2.577977   2.043403   0.087463
 H     -2.150856   2.498727  -0.818757
 C      0.755275  -2.728163  -0.868683
 H      0.093076  -3.169254  -0.097199
 C      2.833590  -1.429523  -0.941559
 H      3.545918  -2.249124  -1.200997
```

```
 H      2.481247  -1.036054  -1.931206
 C      3.574700  -0.334021  -0.239158
 C      3.531006   1.198272   1.529147
 C      4.771370   1.724430   1.163733
 C      5.439349   1.187572   0.046200
 C      4.829990   0.143587  -0.659231
 C      1.400808  -3.905210  -1.622257
 H      0.619944  -4.599623  -1.992316
 H      2.003101  -3.580445  -2.495103
 H      2.054386  -4.463980  -0.922264
 C      0.998750   0.165461   2.840002
 C     -0.054137  -1.875532   1.945473
 H      2.470688  -3.180219   1.080056
 O      2.810156  -3.969988   1.586395
 C      4.203789  -3.794360   1.812695
 H      4.416702  -2.810800   2.301472
 H      4.778386  -3.798291   0.851254
 H      5.313753  -0.309400  -1.537700
 H      5.207303   2.543568   1.754105
 H      2.987457   1.589441   2.400909
 C      4.712500  -4.920873   2.704894
 H      5.800890  -4.816808   2.893974
 H      4.186856  -4.914292   3.682872
 H      4.535911  -5.908583   2.229201
 H      6.419982   1.578172  -0.265725
 N     -5.645125   2.445566   2.238743
 N      2.603579   5.481168  -1.682374
 O      3.094218   5.511939  -2.817618
 O      2.675648   6.408426  -0.866621
 O     -6.208346   3.341333   1.600889
 O     -6.091966   1.933890   3.270497
---------------------------------------
Mn27/iii
Frequencies, energies and thermodynamic properties:
 Lowest Vibrational Mode (1/cm) =                        18.2438
 2nd Lowest Vibrational Mode (1/cm) =                    20.9639
 E(RB-P86) (a.u.) =                                    -4816.26268531
 Thermal correction to Enthalpy (a.u.) =                 0.652802
 Thermal correction to Gibbs Free Energy (a.u.) =        0.520929
 Total Entropy (cal/Kmol) =                              277.549
 E(RPBE1PBE) (a.u.) =                                  -4815.61162532
Optimised cartesian coordinates (Angstrom):
 Fe2.128438  -1.988764   1.832057
 Mn-1.178956  -0.745159  -1.538540
 P      0.233419   0.313116  -0.123306
 O     -0.453871   1.101241  -3.737485
 O      0.799447  -2.699002  -2.512704
 N     -1.826537  -1.937880   0.045538
 N     -2.853086   0.315949  -0.942366
 C      0.654778  -0.640717   1.372986
 C      0.070855  -1.939669   1.698897
 C      0.488941  -2.257786   3.043463
 H      0.240048  -3.176334   3.589581
 C      1.323958  -1.197795   3.541462
 H      1.813177  -1.168727   4.524299
 C      1.441097  -0.200735   2.514043
 H      2.011558   0.733789   2.589510
 C      3.079708  -2.727951   0.156863
 H      2.704386  -2.675187  -0.872891
 C      2.812859  -3.787652   1.091582
 H      2.199127  -4.677912   0.899761
 C      3.468075  -3.463096   2.332708
 H      3.437415  -4.059977   3.254003
 C      4.143953  -2.202048   2.163226
 H      4.721008  -1.671303   2.932209
 C      3.901917  -1.745239   0.819585
 H      4.271414  -0.809496   0.380918
 C     -0.485554   1.899378   0.539863
 C     -0.636919   2.987687  -0.355329
 H     -0.276664   2.907171  -1.393053
 C     -1.236128   4.181924   0.062618
 H     -1.353650   5.036811  -0.616965
 C     -1.696787   4.277528   1.386692
 C     -1.570921   3.215387   2.294477
 H     -1.940629   3.332134   3.322246
 C     -0.963721   2.025840   1.863377
 H     -0.850334   1.195418   2.576002
 C      1.880258   0.945204  -0.743350
 C      2.476860   0.391420  -1.897704
 H      1.968546  -0.400205  -2.463761
 C      3.729009   0.839059  -2.345471
 H      4.202090   0.417599  -3.242690
 C      4.379932   1.851711  -1.626087
 C      3.813760   2.428796  -0.478704
 H      4.352694   3.223929   0.054075
```

```
 C      2.561233   1.972676  -0.045865
 H      2.110224   2.438082   0.843254
 C     -0.846140  -2.760777   0.787489
 H     -0.206477  -3.229424   0.011145
 C     -2.723006  -1.206266   0.936129
 H     -3.459671  -1.868466   1.452420
 H     -2.176088  -0.689494   1.772728
 C     -3.465161  -0.137865   0.187220
 C     -3.439939   1.319767  -1.645058
 C     -4.650956   1.904066  -1.262097
 C     -5.293525   1.433328  -0.103212
 C     -4.690566   0.400067   0.624880
 C     -1.514360  -3.920711   1.557636
 H     -0.752281  -4.625890   1.947879
 H     -2.115483  -3.566218   2.420473
 H     -2.178798  -4.479703   0.868716
 C     -0.759860   0.361230  -2.871420
 C      0.045019  -1.902584  -2.079172
 H     -2.279313  -1.272758  -2.763763
 H     -2.330523  -1.915535  -2.239961
 H     -2.706473  -3.023753  -0.788332
 O     -3.181763  -3.691428  -1.435290
 C     -4.586358  -3.533747  -1.332140
 H     -4.942066  -3.715724  -0.285139
 H     -4.904463  -2.489424  -1.587870
 H     -5.158834  -0.001319   1.536050
 H     -5.080950   2.711583  -1.872330
 H     -2.915274   1.655930  -2.551245
 C     -5.286014  -4.511887  -2.272285
 H     -5.010850  -5.558136  -2.021281
 H     -6.389747  -4.416387  -2.201550
 H     -4.988849  -4.326398  -3.326155
 H     -6.252055   1.864239   0.224482
 N     -2.332704   5.536767   1.836779
 N      5.703116   2.332446  -2.094970
 O     -2.733102   5.595588   3.004926
 O     -2.428116   6.458946   1.018929
 O      6.252596   3.227818  -1.445001
 O      6.179522   1.809205  -3.107354
---------------------------------------
Mn27/iv
Frequencies, energies and thermodynamic properties:
 Lowest Vibrational Mode (1/cm) =                         18.0480
 2nd Lowest Vibrational Mode (1/cm) =                     19.8644
 E(RB-P86) (a.u.) =                                     -4816.29360242
 Thermal correction to Enthalpy (a.u.) =                  0.657358
 Thermal correction to Gibbs Free Energy (a.u.) =         0.525124
 Total Entropy (cal/Kmol) =                               278.310
 E(RPBE1PBE) (a.u.) =                                   -4815.64024054
Optimised cartesian coordinates (Angstrom):
 Fe-2.297591  -1.760536  -1.917372
 Mn1.195822  -0.854010   1.364178
 P     -0.232570   0.352979   0.087852
 O      0.814499   0.775735   3.782965
 O     -0.711170  -2.835237   2.414868
 N      1.728392  -2.033285  -0.352065
 N      2.923937   0.091623   0.771989
 C     -0.759986  -0.490157  -1.454321
 C     -0.246377  -1.791442  -1.887177
 C     -0.736864  -2.015727  -3.227865
 H     -0.551864  -2.907928  -3.838681
 C     -1.548529  -0.896861  -3.620969
 H     -2.081711  -0.792977  -4.575297
 C     -1.573073   0.041240  -2.534304
 H     -2.109109   0.999029  -2.526967
 C     -3.178045  -2.551486  -0.230836
 H     -2.737700  -2.568155   0.774598
 C     -3.012920  -3.568566  -1.235048
 H     -2.429289  -4.492713  -1.127808
 C     -3.727309  -3.152257  -2.414296
 H     -3.777020  -3.699071  -3.365332
 C     -4.337969  -1.877470  -2.137870
 H     -4.936458  -1.283017  -2.840970
 C     -3.997293  -1.504124  -0.789842
 H     -4.299062  -0.579121  -0.282208
 C      0.515167   1.942296  -0.528262
 C      0.788358   2.953352   0.429442
 H      0.469176   2.821688   1.475672
 C      1.457681   4.127280   0.067154
 H      1.663617   4.922433   0.796533
 C      1.875400   4.283829  -1.266989
 C      1.636745   3.295670  -2.235506
 H      1.974982   3.456552  -3.268240
 C      0.957306   2.127640  -1.859405
 H      0.753787   1.361863  -2.623105
```

```
 C     -1.830021   1.002071   0.810194
 C     -2.388358   0.382010   1.950048
 H     -1.875583  -0.467768   2.421634
 C     -3.599146   0.835446   2.494620
 H     -4.045097   0.362287   3.379947
 C     -4.245426   1.923748   1.888760
 C     -3.713504   2.569783   0.761345
 H     -4.247471   3.422266   0.319953
 C     -2.502623   2.104568   0.230087
 H     -2.075301   2.619647  -0.643486
 C      0.633972  -2.749474  -1.093617
 H      0.018891  -3.201443  -0.290241
 C      2.643402  -1.265243  -1.223925
 H      3.288796  -1.917338  -1.850941
 H      2.028265  -0.660414  -1.927794
 C      3.485003  -0.327466  -0.399612
 C      3.607301   1.001787   1.515350
 C      4.856651   1.509004   1.140340
 C      5.445217   1.060118  -0.053315
 C      4.742774   0.126163  -0.829963
 C      1.186008  -3.899477  -1.952797
 H      0.357300  -4.524679  -2.340363
 H      1.772148  -3.538058  -2.822175
 H      1.834883  -4.552530  -1.334702
 C      0.960702   0.127381   2.802820
 C      0.013978  -2.019479   1.955184
 H      2.211404  -1.744096   2.216430
 H      2.676946  -3.130109   1.922692
 H      2.287821  -2.780258   0.115461
 O      3.036014  -3.927785   1.429212
 C      4.452563  -3.968845   1.638632
 H      4.856674  -4.691738   0.897369
 H      4.918681  -2.980461   1.409910
 H      5.159741  -0.255790  -1.773980
 H      5.358268   2.242100   1.789094
 H      3.130182   1.320732   2.453071
 C      4.821721  -4.407431   3.054818
 H      4.378673  -5.398004   3.287865
 H      5.923750  -4.483705   3.169153
 H      4.452615  -3.679184   3.807984
 H      6.431775   1.428385  -0.373222
 N     -5.525353   2.409147   2.458476
 N      2.581632   5.518953  -1.658936
 O     -5.972492   1.824833   3.451186
 O     -6.073518   3.370109   1.907668
 O      2.775097   6.376991  -0.787679
 O      2.943114   5.631445  -2.837711
---------------------------------------
Mn27/v
Frequencies, energies and thermodynamic properties:
 Lowest Vibrational Mode (1/cm) =                          18.8507
 2nd Lowest Vibrational Mode (1/cm) =                      21.3106
 E(RB-P86) (a.u.) =                                      -4661.35595954
 Thermal correction to Enthalpy (a.u.) =                   0.572755
 Thermal correction to Gibbs Free Energy (a.u.) =          0.454637
 Total Entropy (cal/Kmol) =                                248.601
 E(RPBE1PBE) (a.u.) =                                    -4660.69798175
Optimised cartesian coordinates (Angstrom):
 Fe-2.552180  -1.640195  -1.468382
 Mn0.779435  -1.000590   2.029693
 P      0.062092   0.127190   0.206690
 O      0.986442   1.348655   3.785892
 O     -1.806572  -1.683753   3.255698
 N      0.877184  -2.815478   0.886137
 N      2.778258  -1.051664   1.568892
 C     -0.661874  -0.946112  -1.095627
 C     -0.704714  -2.408669  -1.019503
 C     -1.153440  -2.887411  -2.307591
 H     -1.313993  -3.936865  -2.583563
 C     -1.401433  -1.760097  -3.163310
 H     -1.779008  -1.805723  -4.193428
 C     -1.107511  -0.565062  -2.424121
 H     -1.198626   0.461278  -2.802338
 C     -3.786901  -1.444987   0.168828
 H     -3.457451  -1.299532   1.206016
 C     -4.001177  -2.714820  -0.473326
 H     -3.867297  -3.702537  -0.012143
 C     -4.400018  -2.465539  -1.834624
 H     -4.614803  -3.229020  -2.594146
 C     -4.436399  -1.039729  -2.033660
 H     -4.685399  -0.525440  -2.971447
 C     -4.055668  -0.408224  -0.797419
 H     -3.972493   0.672412  -0.625603
 C      1.435223   1.008123  -0.686632
 C      2.037286   2.117009  -0.035626
```

H   1.617126   2.494111   0.910549
C   3.161589   2.748334 -0.577569
H   3.627328   3.615438 -0.089780
C   3.704813   2.253020 -1.777700
C   3.145199   1.146432 -2.437544
H   3.594719   0.791281 -3.374931
C   2.013189   0.529006 -1.886657
H   1.564685 -0.326361 -2.414321
C   -1.171704   1.520676   0.377965
C   -2.000371   1.580497   1.520332
H   -1.905484   0.818680   2.306737
C   -2.949351   2.602928   1.668161
H   -3.600449   2.661463   2.550879
C   -3.056977   3.573111   0.659717
C   -2.242358   3.549590 -0.483852
H   -2.354354   4.331369 -1.247396
C   -1.299030   2.521043 -0.615725
H   -0.648354   2.509675 -1.503361
C   -0.363054 -3.288848   0.177262
H   -1.162435 -3.172956   0.936132
C   2.096195 -2.819364   0.046765
H   2.443905 -3.844567 -0.202783
H   1.842939 -2.325761 -0.917972
C   3.193585 -2.035289   0.717308
C   3.730047 -0.264887   2.139130
H   3.368566   0.518897   2.819952
C   5.099691 -0.431222   1.904154
H   5.816818   0.239052   2.400685
C   5.526956 -1.457880   1.046303
C   4.551143 -2.269890   0.447631
H   4.832019 -3.087156 -0.233622
C   -0.280606 -4.779428 -0.189687
H   -1.267582 -5.145559 -0.534956
H   0.455044 -4.979221 -0.994952
H   -0.000220 -5.379969   0.699953
C   0.895722   0.412492   3.064759
C   -0.797064 -1.384700   2.712637
H   1.027099 -3.465507   1.670326
H   1.216708 -1.917767   3.240939
H   6.596512 -1.624616   0.847976
N   4.889158   2.911924 -2.356390
N   -4.056011   4.658237   0.808128
O   -4.759420   4.659223   1.824219
O   -4.130381   5.500364 -0.093504
O   5.360426   3.888786 -1.757609
O   5.351737   2.454865 -3.410967

Mn27/vi_R
Frequencies, energies and thermodynamic properties:
Lowest Vibrational Mode (1/cm) =   11.8749
2nd Lowest Vibrational Mode (1/cm) =   17.1896
E(RB-P86) (a.u.) =   -5084.06530224
Thermal correction to Enthalpy (a.u.) =   0.725332
Thermal correction to Gibbs Free Energy (a.u.) =   0.584549
Total Entropy (cal/Kmol) =   296.303
E(RPBE1PBE) (a.u.) =   -5083.40370898
Optimised cartesian coordinates (Angstrom):
Fe-1.986010 -2.278140 -2.277945
Mn1.209901   0.174660   0.313265
P   -0.964682   0.219570   0.059502
O   1.210060   0.557664   3.253610
O   1.369775 -2.722139   0.811891
N   1.559308   0.069368 -1.611491
N   1.635707   2.157781 -0.051724
C   -1.461406 -0.425836 -1.581085
C   -0.519810 -0.871006 -2.607962
C   -1.284394 -1.099911 -3.810395
H   -0.883306 -1.460719 -4.765872
C   -2.669631 -0.818753 -3.544983
H   -3.497665 -0.925125 -4.258508
C   -2.788978 -0.411538 -2.172603
H   -3.721843 -0.128527 -1.668538
C   -1.459041 -3.780573 -0.966622
H   -0.739271 -3.692588 -0.142812
C   -1.144026 -4.159832 -2.317770
H   -0.145932 -4.409679 -2.701900
C   -2.362313 -4.129733 -3.085660
H   -2.454764 -4.346700 -4.158187
C   -3.432899 -3.734222 -2.206667
H   -4.484771 -3.599210 -2.491840
C   -2.875145 -3.515091 -0.897459
H   -3.429660 -3.193043 -0.006733
C   -1.604635   1.967152   0.064138
C   -1.685595   2.649649   1.303550
H   -1.452026   2.123115   2.242495

```
 C     -2.065057   3.995867   1.356464
 H     -2.142853   4.536241   2.309595
 C     -2.349434   4.664218   0.152799
 C     -2.264016   4.021074  -1.091150
 H     -2.497251   4.579282  -2.008050
 C     -1.889826   2.669172  -1.129225
 H     -1.839064   2.155350  -2.100678
 C     -2.142083  -0.573862   1.269938
 C     -1.688001  -1.568265   2.163862
 H     -0.632461  -1.871024   2.169862
 C     -2.573692  -2.186049   3.059928
 H     -2.234235  -2.959944   3.761560
 C     -3.920485  -1.794519   3.054260
 C     -4.403258  -0.803234   2.185366
 H     -5.464140  -0.520322   2.217141
 C     -3.506101  -0.193762   1.297819
 H     -3.879216   0.597379   0.630162
 C      0.993955  -1.034172  -2.424879
 H      1.160099  -1.950514  -1.823843
 C      1.581215   1.345908  -2.324163
 H      2.354698   1.374587  -3.127782
 H      0.609835   1.541267  -2.848638
 C      1.826176   2.470676  -1.365303
 C      1.808373   3.137917   0.878115
 H      1.648325   2.851164   1.927133
 C      2.178200   4.441761   0.541529
 H      2.305940   5.190780   1.336532
 C      2.385494   4.764181  -0.813149
 C      2.209421   3.761856  -1.773979
 H      2.362549   3.965238  -2.844458
 C      1.718630  -1.247612  -3.767645
 H      1.374200  -2.187618  -4.243693
 H      1.539620  -0.422569  -4.486989
 H      2.810565  -1.338407  -3.598897
 C      1.210544   0.408598   2.081391
 C      1.255954  -1.567219   0.583563
 H      3.182209  -0.237629  -1.509962
 H      3.384524  -0.040622   0.523430
 C      4.405331  -0.140002  -0.001896
 C      5.149267  -1.259187   0.713363
 C      5.245281   1.115205   0.369348
 C      5.993243  -0.728876   1.714094
 C      5.079926  -2.641513   0.483866
 C      5.886690   0.786930   1.739830
 H      6.033391   1.211871  -0.409454
 C      6.772856  -1.588244   2.506557
 C      5.864309  -3.502592   1.276713
 H      4.427594  -3.038926  -0.310020
 H      6.862527   1.289739   1.905656
 C      6.703052  -2.978744   2.282094
 H      7.440026  -1.184800   3.286411
 H      5.828219  -4.591105   1.107763
 H      7.316156  -3.661899   2.892082
 O      4.195667  -0.337038  -1.366424
 H      4.650691   2.050732   0.365833
 H      5.220690   1.108341   2.573623
 H      2.682416   5.781239  -1.111398
 N     -2.751346   6.087913   0.199389
 N     -4.862813  -2.441990   4.000257
 O     -4.413069  -3.307441   4.758143
 O     -6.043317  -2.078720   3.974650
 O     -2.818027   6.632288   1.307604
 O     -2.997515   6.654113  -0.871920
---------------------------------------
Mn27/vi_S
Frequencies, energies and thermodynamic properties:
 Lowest Vibrational Mode (1/cm) =                         14.2400
 2nd Lowest Vibrational Mode (1/cm) =                     17.3289
 E(RB-P86) (a.u.) =                                     -5084.06557697
 Thermal correction to Enthalpy (a.u.) =                  0.725485
 Thermal correction to Gibbs Free Energy (a.u.) =         0.585646
 Total Entropy (cal/Kmol) =                               294.315
 E(RPBE1PBE) (a.u.) =                                   -5083.40431630
Optimised cartesian coordinates (Angstrom):
 Fe-2.893449  -1.837609  -1.976581
 Mn1.035921  -0.514293   0.422659
 P     -0.931067   0.365507   0.032019
 O      1.197391   0.289167   3.272518
 O      0.005791  -3.111822   1.345891
 N      1.303385  -1.047513  -1.442464
 N      2.239257   1.040843  -0.196377
 C     -1.653436  -0.272380  -1.525703
 C     -0.980317  -1.217729  -2.416173
 C     -1.774891  -1.304616  -3.617694
 H     -1.559494  -1.943739  -4.483221
```

```
 C     -2.921975  -0.447636  -3.483193
 H     -3.722832  -0.320771  -4.223884
 C     -2.859947   0.184915  -2.194924
 H     -3.593102   0.898313  -1.797556
 C     -3.030334  -3.189131  -0.424689
 H     -2.339483  -3.266259   0.424598
 C     -2.894556  -3.877993  -1.679980
 H     -2.084849  -4.567820  -1.953114
 C     -3.991887  -3.484726  -2.526159
 H     -4.162096  -3.817623  -3.558694
 C     -4.809232  -2.553536  -1.791203
 H     -5.712886  -2.054035  -2.165186
 C     -4.214244  -2.367934  -0.493218
 H     -4.590646  -1.708718   0.299516
 C     -0.799468   2.199694  -0.255243
 C     -0.591203   3.042704   0.864847
 H     -0.592417   2.622100   1.882930
 C     -0.384598   4.417040   0.698436
 H     -0.232105   5.085528   1.556553
 C     -0.371117   4.944123  -0.604792
 C     -0.559408   4.134121  -1.734697
 H     -0.543987   4.586473  -2.735603
 C     -0.773576   2.759251  -1.553181
 H     -0.939111   2.122869  -2.435202
 C     -2.326301   0.316917   1.269700
 C     -2.316959  -0.622598   2.323992
 H     -1.478645  -1.322586   2.438733
 C     -3.374846  -0.678988   3.244162
 H     -3.380130  -1.403423   4.069767
 C     -4.442413   0.219103   3.099993
 C     -4.479522   1.170811   2.068746
 H     -5.330676   1.861160   1.994661
 C     -3.414679   1.216295   1.158753
 H     -3.433188   1.975211   0.362207
 C      0.336234  -1.945325  -2.118708
 H      0.118021  -2.743459  -1.381138
 C      1.833547  -0.012781  -2.329499
 H      2.541908  -0.420245  -3.088935
 H      1.019300   0.475833  -2.925625
 C      2.525843   1.047865  -1.529495
 C      2.806968   1.994654   0.592231
 H      2.557487   1.957203   1.662109
 C      3.668328   2.973584   0.092463
 H      4.098272   3.717890   0.778373
 C      3.971184   2.978574  -1.282188
 C      3.394229   1.998360  -2.098080
 H      3.602877   1.959876  -3.177792
 C      0.908079  -2.642116  -3.368151
 H      0.211175  -3.428662  -3.721161
 H      1.076727  -1.940925  -4.210832
 H      1.870347  -3.133638  -3.119786
 C      1.136594  -0.027450   2.135793
 C      0.367692  -2.058761   0.944908
 H      2.685335  -1.937890  -1.213832
 H      2.966658  -1.504990   0.776167
 C      3.791389  -2.194495   0.361598
 C      3.754560  -3.506153   1.196722
 C      4.593949  -3.205348   2.462763
 O      3.606106  -2.347829  -1.012161
 H      4.649666   3.733938  -1.707134
 C      5.119818  -1.577286   0.772086
 C      5.575794  -2.150186   1.980315
 C      5.863158  -0.584021   0.116693
 C      6.785713  -1.720267   2.550748
 C      7.078247  -0.156041   0.687685
 H      5.503480  -0.160945  -0.835235
 C      7.534086  -0.719062   1.897526
 H      7.154953  -2.165078   3.489815
 H      7.680229   0.617710   0.184144
 H      8.488863  -0.380334   2.331500
 H      5.092221  -4.101090   2.889260
 H      3.951422  -2.787373   3.271826
 H      4.255458  -4.286994   0.583173
 H      2.723898  -3.851516   1.410965
 N     -5.562959   0.167758   4.071562
 N     -0.151648   6.396115  -0.790269
 O     -5.504691  -0.675050   4.972651
 O     -6.489993   0.970662   3.922826
 O      0.012981   7.088310   0.220969
 O     -0.144364   6.836243  -1.945737
---------------------------------------
Mn27/viii
Frequencies, energies and thermodynamic properties:
 Lowest Vibrational Mode (1/cm) =                          19.4463
 2nd Lowest Vibrational Mode (1/cm) =                      23.0087
```

```
 E(RB-P86) (a.u.) =                                        -4661.31958543
 Thermal correction to Enthalpy (a.u.) =                    0.567839
 Thermal correction to Gibbs Free Energy (a.u.) =           0.449235
 Total Entropy (cal/Kmol) =                                 249.621
 E(RPBE1PBE) (a.u.) =                                      -4660.66337356
Optimised cartesian coordinates (Angstrom):
 Fe-2.598557  -1.583441  -1.463288
 Mn0.732437  -1.093919   2.063717
 P      0.072880   0.055065   0.240604
 O      0.901135   1.370893   3.704367
 O     -1.986294  -1.591838   3.081004
 N      0.812999  -2.777148   0.902635
 N      2.742653  -1.090213   1.562908
 C     -0.679941  -0.976383  -1.065124
 C     -0.773846  -2.432098  -0.996438
 C     -1.233732  -2.881071  -2.288447
 H     -1.430558  -3.923471  -2.569754
 C     -1.436413  -1.739166  -3.140519
 H     -1.805916  -1.761211  -4.174644
 C     -1.107446  -0.558349  -2.390468
 H     -1.157880   0.472245  -2.764479
 C     -3.860289  -1.324660   0.149701
 H     -3.550582  -1.166552   1.190448
 C     -4.091174  -2.601854  -0.469303
 H     -3.987421  -3.581890   0.015320
 C     -4.455837  -2.372095  -1.844080
 H     -4.674643  -3.146091  -2.591813
 C     -4.453867  -0.949605  -2.073517
 H     -4.673376  -0.449867  -3.026510
 C     -4.082606  -0.301438  -0.842647
 H     -3.978715   0.780527  -0.689992
 C      1.493801   0.897612  -0.619570
 C      2.092621   2.013231   0.017808
 H      1.679379   2.399582   0.962954
 C      3.208442   2.645696  -0.542698
 H      3.678063   3.516520  -0.065574
 C      3.734454   2.144943  -1.746121
 C      3.171910   1.035052  -2.395560
 H      3.610794   0.676918  -3.336698
 C      2.050570   0.413817  -1.824760
 H      1.598748  -0.449502  -2.335730
 C     -1.098461   1.503211   0.411217
 C     -1.934806   1.620615   1.543366
 H     -1.887944   0.876894   2.349685
 C     -2.840598   2.685592   1.661824
 H     -3.494820   2.788375   2.538084
 C     -2.900319   3.637607   0.633662
 C     -2.078895   3.556483  -0.501783
 H     -2.151063   4.325742  -1.282572
 C     -1.177113   2.488432  -0.603337
 H     -0.519917   2.436030  -1.484199
 C     -0.382926  -3.277684   0.225933
 H     -1.211918  -3.189960   0.959370
 C      2.008612  -2.871605   0.095518
 H      2.370823  -3.923531  -0.038543
 H      1.878480  -2.495741  -0.964107
 C      3.124980  -2.061224   0.686759
 C      3.696893  -0.291390   2.109928
 C      5.056283  -0.425379   1.815421
 C      5.459180  -1.430560   0.916542
 C      4.479395  -2.255014   0.351250
 C     -0.288381  -4.775614  -0.141095
 H     -1.271373  -5.165628  -0.476950
 H      0.441978  -4.967651  -0.954456
 H      0.024808  -5.356357   0.750292
 C      0.843769   0.375766   3.071117
 C     -0.921575  -1.375134   2.618409
 H      1.194606  -2.479441   3.034953
 H      1.347468  -1.864696   3.555956
 H      3.347497   0.477370   2.814438
 H      5.784042   0.248031   2.291224
 H      4.748111  -3.056595  -0.353321
 H      6.522074  -1.569077   0.665503
 N      4.912958   2.807682  -2.345013
 N     -3.854657   4.767226   0.752167
 O     -4.560835   4.820326   1.764365
 O     -3.889539   5.589590  -0.169223
 O      5.383219   3.790887  -1.758995
 O      5.364039   2.342801  -3.399296
---------------------------------------
Mn27/ix
Frequencies, energies and thermodynamic properties:
 Lowest Vibrational Mode (1/cm) =                           18.4527
 2nd Lowest Vibrational Mode (1/cm) =                       22.1944
 E(RB-P86) (a.u.) =                                        -4815.10849218
```

```
 Thermal correction to Enthalpy (a.u.) =                  0.637281
 Thermal correction to Gibbs Free Energy (a.u.) =         0.509938
 Total Entropy (cal/Kmol) =                               268.016
 E(RPBE1PBE) (a.u.) =                                    -4814.45538878
Optimised cartesian coordinates (Angstrom):
 Fe-2.659521  -1.254496  -1.904524
 Mn0.940931  -1.281082   1.380546
 P     -0.052713   0.233473   0.046257
 O      1.051428   0.560085   3.675046
 O     -1.577737  -2.392652   2.417868
 N      1.078229  -2.617075  -0.275434
 N      2.864622  -0.848097   0.699261
 C     -0.805019  -0.500391  -1.462623
 C     -0.715702  -1.913117  -1.837963
 C     -1.246306  -2.032570  -3.175825
 H     -1.341079  -2.963737  -3.748156
 C     -1.672689  -0.735979  -3.625376
 H     -2.143894  -0.512310  -4.591689
 C     -1.412791   0.208759  -2.575835
 H     -1.629122   1.283766  -2.617443
 C     -3.763228  -1.654192  -0.208581
 H     -3.364403  -1.754033   0.809032
 C     -3.902866  -2.721790  -1.162642
 H     -3.631607  -3.773380  -0.999034
 C     -4.439274  -2.166747  -2.378620
 H     -4.640914  -2.718900  -3.306196
 C     -4.634271  -0.754212  -2.175026
 H     -5.012349  -0.041440  -2.919969
 C     -4.214017  -0.436045  -0.835387
 H     -4.224915   0.560903  -0.376632
 C      1.139616   1.485520  -0.650252
 C      1.660880   2.469142   0.228513
 H      1.293124   2.534473   1.264925
 C      2.640481   3.371285  -0.200976
 H      3.043419   4.145797   0.465639
 C      3.116480   3.274812  -1.521085
 C      2.633420   2.302546  -2.410972
 H      3.027281   2.262335  -3.435690
 C      1.645263   1.410620  -1.968832
 H      1.252632   0.660686  -2.671845
 C     -1.374572   1.383975   0.698362
 C     -2.084597   1.059885   1.875938
 H     -1.855193   0.138386   2.426914
 C     -3.093048   1.904324   2.364222
 H     -3.650854   1.663695   3.279320
 C     -3.384108   3.083873   1.662723
 C     -2.692609   3.441883   0.494494
 H     -2.945102   4.377427  -0.022842
 C     -1.686504   2.588672   0.021443
 H     -1.130648   2.879775  -0.882374
 C     -0.153644  -3.051048  -0.991929
 H     -0.876765  -3.272616  -0.181163
 C      2.227168  -2.311281  -1.144573
 H      2.668821  -3.221021  -1.606282
 H      1.873370  -1.675789  -1.988428
 C      3.285690  -1.550869  -0.385977
 C      3.767754  -0.092219   1.370502
 C      5.114045  -0.007520   0.997733
 C      5.556517  -0.743584  -0.114371
 C      4.626456  -1.526560  -0.811528
 C      0.048988  -4.347114  -1.794744
 H     -0.925019  -4.731054  -2.158189
 H      0.705557  -4.202461  -2.676636
 H      0.495612  -5.129856  -1.148388
 C      1.014525  -0.192372   2.762270
 C     -0.590340  -1.919914   1.969956
 H      1.369143  -3.317683   0.455565
 H      4.927654  -2.121302  -1.687049
 H      5.801232   0.621810   1.581990
 H      3.391803   0.456425   2.246269
 H      6.610300  -0.710969  -0.430824
 O      1.911977  -2.911376   2.086128
 C      1.718160  -3.514657   3.332628
 C      2.480667  -2.840385   4.484159
 H      2.068950  -4.579094   3.266697
 H      0.633623  -3.582840   3.618511
 H      2.343585  -3.399520   5.435882
 H      3.569064  -2.800665   4.262667
 H      2.128130  -1.800501   4.645176
 N     -4.447200   3.982141   2.173279
 N      4.149836   4.222443  -1.983962
 O     -5.035013   3.648936   3.207972
 O     -4.685896   5.013105   1.534746
 O      4.557342   5.071919  -1.180955
 O      4.553144   4.116646  -3.149626
```

```
---------------------------------------
Mn27/x
Frequencies, energies and thermodynamic properties:
 Lowest Vibrational Mode (1/cm) =                         14.1057
 2nd Lowest Vibrational Mode (1/cm) =                     20.1636
 E(RB-P86) (a.u.) =                                     -4970.04935694
 Thermal correction to Enthalpy (a.u.) =                  0.721553
 Thermal correction to Gibbs Free Energy (a.u.) =         0.580573
 Total Entropy (cal/Kmol) =                               296.717
 E(RPBE1PBE) (a.u.) =                                   -4969.40458473
Optimised cartesian coordinates (Angstrom):
 Fe-2.232653  -2.074202  -1.953096
 Mn1.260670  -0.528739   1.117643
 P     -0.487633   0.355220   0.001747
 O      0.540893   1.053178   3.492788
 O     -0.203603  -2.854156   2.170655
 N      1.870500  -1.540979  -0.683651
 N      2.639718   0.830060   0.356692
 C     -0.950617  -0.534071  -1.532172
 C     -0.220412  -1.685839  -2.061101
 C     -0.754175  -1.960165  -3.375025
 H     -0.438764  -2.774437  -4.039007
 C     -1.802043  -1.018109  -3.657768
 H     -2.415760  -0.992177  -4.567952
 C     -1.934871  -0.143080  -2.527099
 H     -2.650767   0.684011  -2.439353
 C     -2.820256  -3.087286  -0.256085
 H     -2.322500  -3.047396   0.721337
 C     -2.516943  -4.013531  -1.314096
 H     -1.750767  -4.799794  -1.283934
 C     -3.377005  -3.713973  -2.429751
 H     -3.375754  -4.226194  -3.401080
 C     -4.216016  -2.603400  -2.059564
 H     -4.968200  -2.122221  -2.698770
 C     -3.871043  -2.213549  -0.717482
 H     -4.322579  -1.389546  -0.150341
 C     -0.144244   2.086619  -0.598883
 C     -0.071278   3.124681   0.364604
 H     -0.302456   2.916611   1.421255
 C      0.286696   4.425953  -0.005741
 H      0.336044   5.241282   0.728701
 C      0.590323   4.685617  -1.353866
 C      0.539640   3.678533  -2.330046
 H      0.778498   3.922600  -3.374144
 C      0.171314   2.380617  -1.945555
 H      0.111905   1.595760  -2.714448
 C     -2.122689   0.635708   0.865832
 C     -2.459062  -0.105260   2.020472
 H     -1.755180  -0.838783   2.435116
 C     -3.694678   0.078266   2.659249
 H     -3.966593  -0.490953   3.558413
 C     -4.593962   1.016555   2.131559
 C     -4.289937   1.775536   0.990508
 H     -5.018521   2.504957   0.611421
 C     -3.050246   1.582041   0.365884
 H     -2.804303   2.191042  -0.516853
 C      0.894613  -2.456525  -1.368896
 H      0.448985  -3.044012  -0.541434
 C      2.551523  -0.605457  -1.604876
 H      3.297229  -1.114738  -2.252508
 H      1.793948  -0.165024  -2.291856
 C      3.205998   0.514079  -0.838761
 C      3.145027   1.875878   1.054743
 C      4.225003   2.641863   0.600584
 C      4.822860   2.309404  -0.626180
 C      4.304116   1.227795  -1.351886
 C      1.602152  -3.451630  -2.304395
 H      0.891148  -4.229221  -2.647862
 H      2.027325  -2.961168  -3.203754
 H      2.419569  -3.957098  -1.752406
 C      0.845754   0.412331   2.546737
 C      0.338789  -1.904848   1.723706
 H      3.486986  -2.531987   1.185171
 H      2.612114  -2.180907  -0.297635
 O      3.792902  -3.273645   0.504641
 C      5.169932  -3.078077   0.207962
 H      5.360957  -3.464164  -0.820504
 H      5.426355  -1.989846   0.188925
 H      4.740896   0.929422  -2.316695
 H      4.590483   3.480670   1.210834
 H      2.662176   2.101679   2.016181
 C      6.089681  -3.801877   1.194935
 H      5.860220  -4.888159   1.216663
 H      7.157272  -3.679176   0.912130
 H      5.961854  -3.406152   2.225013
```

H   5.681273   2.881480 -1.010183
O   2.958986 -1.372631   1.911721
C   3.124357 -1.608077   3.292738
C   3.845389 -0.470071   4.026432
H   3.725064 -2.545694   3.425631
H   2.148536 -1.807338   3.804417
H   4.024563 -0.739066   5.090511
H   4.828250 -0.259128   3.554115
H   3.248249   0.464763   4.010325
N   -5.901711   1.217433   2.801884
N   0.970734   6.056343 -1.754238
O   -6.142530   0.542146   3.808012
O   -6.676214   2.048086   2.315130
O   1.003946   6.926904 -0.875593
O   1.235889   6.258822 -2.945770

Mn27/TS-i
Frequencies, energies and thermodynamic properties:
Lowest Vibrational Mode (1/cm) =   -724.7134
2nd Lowest Vibrational Mode (1/cm) =   17.2163
E(RB-P86) (a.u.) =   -4816.26061076
Thermal correction to Enthalpy (a.u.) =   0.648915
Thermal correction to Gibbs Free Energy (a.u.) =   0.518271
Total Entropy (cal/Kmol) =   274.963
E(RPBE1PBE) (a.u.) =   -4815.60622512
Optimised cartesian coordinates (Angstrom):
Fe2.233310 -1.860308   1.849191
Mn-1.157995 -0.829633 -1.502952
P   0.208393   0.319871 -0.115574
O   -0.580774   1.001434 -3.750241
O   0.909482 -2.684082 -2.487372
N   -1.731890 -2.050366   0.108904
N   -2.883884   0.138179 -0.901170
C   0.687609 -0.595577   1.391395
C   0.177381 -1.921125   1.737576
C   0.623728 -2.203189   3.081255
H   0.429177 -3.127702   3.639163
C   1.405072 -1.094927   3.559886
H   1.901182 -1.029872   4.537460
C   1.458509 -0.103883   2.521639
H   1.979984   0.859939   2.582206
C   3.207764 -2.554382   0.168826
H   2.821342 -2.524462 -0.857802
C   3.004127 -3.623677   1.108744
H   2.436562 -4.545609   0.924370
C   3.651963 -3.261926   2.343323
H   3.659948 -3.856808   3.266372
C   4.259996 -1.968169   2.164810
H   4.814673 -1.405613   2.927602
C   3.983495 -1.528552   0.822057
H   4.300070 -0.575985   0.378240
C   -0.587951   1.872077   0.538400
C   -0.806853   2.939206 -0.368642
H   -0.447574   2.866830 -1.407359
C   -1.472652   4.101509   0.037996
H   -1.642125   4.939957 -0.651042
C   -1.933328   4.186022   1.362975
C   -1.742455   3.143549   2.282266
H   -2.114561   3.250785   3.310221
C   -1.068111   1.986632   1.862500
H   -0.902607   1.173797   2.585215
C   1.817427   1.028997 -0.750530
C   2.433290   0.493148 -1.903181
H   1.960948 -0.329465 -2.456070
C   3.658552   0.997769 -2.364550
H   4.146793   0.590095 -3.260027
C   4.262430   2.049787 -1.660654
C   3.674657   2.611620 -0.516492
H   4.176315   3.438982   0.003465
C   2.449306   2.098262 -0.070055
H   1.979915   2.550944   0.816147
C   -0.694090 -2.810308   0.851098
H   -0.040689 -3.245149   0.067226
C   -2.668248 -1.354851   0.997625
H   -3.360427 -2.050993   1.526407
H   -2.129138 -0.806749   1.813086
C   -3.469338 -0.339120   0.233174
C   -3.528989   1.100748 -1.610413
C   -4.771595   1.617429 -1.230341
C   -5.386194   1.119493 -0.068296
C   -4.723740   0.128374   0.667440
C   -1.291125 -3.997595   1.633863
H   -0.487621 -4.655992   2.021708
H   -1.905255 -3.671519   2.498494
H   -1.926869 -4.600788   0.955089

```
 C     -0.823109   0.268310  -2.858000
 C      0.116638  -1.925943  -2.051903
 H     -2.235552  -1.485878  -2.648292
 H     -2.310103  -2.209779  -2.179787
 H     -2.405182  -2.942739  -0.646129
 O     -2.861455  -3.619083  -1.497002
 C     -4.273913  -3.624023  -1.495097
 H     -4.674847  -3.812833  -0.465408
 H     -4.691510  -2.627570  -1.803360
 H     -5.168092  -0.292735   1.581691
 H     -5.247427   2.394102  -1.846593
 H     -3.026815   1.457780  -2.521142
 C     -4.814313  -4.696540  -2.442642
 H     -4.445058  -5.700846  -2.144883
 H     -5.925028  -4.718479  -2.436860
 H     -4.478420  -4.507591  -3.484421
 H     -6.368202   1.496262   0.256395
 N      5.558171   2.589035  -2.142131
 N     -2.639495   5.411015   1.801298
 O     -3.037985   5.460601   2.970634
 O     -2.791976   6.316349   0.973175
 O      6.068289   3.515170  -1.503205
 O      6.052937   2.080430  -3.153181
---------------------------------------
Mn27/TS-ii_si
Frequencies, energies and thermodynamic properties:
 Lowest Vibrational Mode (1/cm) =        -284.3930
 2nd Lowest Vibrational Mode (1/cm) =                     11.4027
 E(RB-P86) (a.u.) =                                     -5084.06164338
 Thermal correction to Enthalpy (a.u.) =                 0.721859
 Thermal correction to Gibbs Free Energy (a.u.) =        0.583760
 Total Entropy (cal/Kmol) =                              290.653
 E(RPBE1PBE) (a.u.) =                                   -5083.39413024
Optimised cartesian coordinates (Angstrom):
 Fe-1.914872  -2.223385  -2.319304
 Mn1.343718   0.160329   0.364070
 P     -0.875732   0.257749   0.033331
 O      1.278076   0.491391   3.289502
 O      1.521734  -2.749881   0.758873
 N      1.693008   0.080174  -1.707394
 N      1.733985   2.140034  -0.014915
 C     -1.386229  -0.376330  -1.610658
 C     -0.450519  -0.821163  -2.645772
 C     -1.222025  -1.038462  -3.847884
 H     -0.828409  -1.394206  -4.808081
 C     -2.603881  -0.754337  -3.573895
 H     -3.434834  -0.855829  -4.284538
 C     -2.713960  -0.353638  -2.199651
 H     -3.642663  -0.071778  -1.687195
 C     -1.373811  -3.737295  -1.028748
 H     -0.639118  -3.657628  -0.217193
 C     -1.084102  -4.108366  -2.388216
 H     -0.094277  -4.361070  -2.791503
 C     -2.315286  -4.067750  -3.134415
 H     -2.427224  -4.276081  -4.206789
 C     -3.368480  -3.674064  -2.234251
 H     -4.424402  -3.531653  -2.500120
 C     -2.787336  -3.466883  -0.933517
 H     -3.324858  -3.147952  -0.031424
 C     -1.531318   1.999910   0.045717
 C     -1.558771   2.686041   1.287015
 H     -1.290074   2.157750   2.215696
 C     -1.929542   4.033450   1.356504
 H     -1.966365   4.572913   2.312665
 C     -2.259144   4.706009   0.166138
 C     -2.226638   4.061197  -1.080253
 H     -2.495237   4.620223  -1.986981
 C     -1.861050   2.707899  -1.133532
 H     -1.854198   2.196725  -2.107997
 C     -2.067837  -0.548738   1.225001
 C     -1.623326  -1.585307   2.075250
 H     -0.574098  -1.909408   2.049148
 C     -2.508203  -2.217006   2.962050
 H     -2.175670  -3.023727   3.629235
 C     -3.846291  -1.796228   2.992951
 C     -4.319884  -0.763993   2.167524
 H     -5.373933  -0.460397   2.225890
 C     -3.422830  -0.142383   1.288002
 H     -3.788311   0.678762   0.653008
 C      1.055500  -1.019304  -2.507091
 H      1.232852  -1.930548  -1.901966
 C      1.553254   1.416816  -2.324273
 H      2.175135   1.528354  -3.238174
 H      0.495026   1.555297  -2.642659
 C      1.895580   2.486833  -1.325135
```

```
 C      1.946512   3.097373   0.928724
 H      1.806501   2.790219   1.974889
 C      2.333047   4.404589   0.614275
 H      2.489770   5.130954   1.425107
 C      2.521505   4.754933  -0.733381
 C      2.299641   3.775899  -1.712328
 H      2.431533   4.001023  -2.781315
 C      1.753217  -1.226386  -3.862113
 H      1.393136  -2.158203  -4.341530
 H      1.569855  -0.390563  -4.567483
 H      2.846859  -1.328324  -3.711431
 C      1.285152   0.362664   2.112805
 C      1.401444  -1.586553   0.579474
 H      2.737962  -0.117016  -1.687593
 H      2.994135  -0.011120   0.475115
 C      4.488835  -0.102952  -0.148144
 C      4.871286  -1.327999   0.645728
 C      5.042057   1.088129   0.676567
 C      5.310392  -0.948771   1.933648
 C      4.891544  -2.668315   0.228154
 C      5.188576   0.554131   2.118055
 H      6.043222   1.312225   0.241966
 C      5.771983  -1.926943   2.829963
 C      5.357508  -3.645195   1.126269
 H      4.556660  -2.936118  -0.786809
 H      6.047772   0.989920   2.668701
 C      5.791690  -3.275245   2.417908
 H      6.123424  -1.648832   3.837294
 H      5.390924  -4.703857   0.822374
 H      6.158483  -4.050371   3.110145
 O      4.354425  -0.086134  -1.408576
 H      4.430286   2.004779   0.580846
 H      4.276546   0.783112   2.715115
 H      2.836343   5.770875  -1.016171
 N     -2.650815   6.128980   0.229356
 N     -4.787645  -2.456575   3.929712
 O     -5.960203  -2.066735   3.937646
 O     -4.346388  -3.359497   4.648358
 O     -2.675395   6.673296   1.340427
 O     -2.932546   6.699964  -0.831749
---------------------------------------
Mn27/TS-ii_re
Frequencies, energies and thermodynamic properties:
 Lowest Vibrational Mode (1/cm) =         -282.2733
 2nd Lowest Vibrational Mode (1/cm) =                    10.6711
 E(RB-P86) (a.u.) =                                    -5084.06215269
 Thermal correction to Enthalpy (a.u.) =                 0.721962
 Thermal correction to Gibbs Free Energy (a.u.) =        0.584350
 Total Entropy (cal/Kmol) =                              289.628
 E(RPBE1PBE) (a.u.) =                                  -5083.39583940
Optimised cartesian coordinates (Angstrom):
 Fe-3.053194  -1.613272  -1.905837
 Mn1.092878  -0.713557   0.444967
 P     -0.802169   0.407263  -0.009038
 O      1.320883   0.141531   3.254260
 O     -0.175599  -3.197540   1.378417
 N      1.249524  -1.343241  -1.553854
 N      2.390493   0.703212  -0.281827
 C     -1.638025  -0.180683  -1.533113
 C     -1.102118  -1.220805  -2.413652
 C     -1.937243  -1.248927  -3.592102
 H     -1.821688  -1.929785  -4.444413
 C     -2.976869  -0.266770  -3.451442
 H     -3.780967  -0.072912  -4.173690
 C     -2.804047   0.389048  -2.185995
 H     -3.440133   1.189740  -1.787600
 C     -4.346157  -1.970002  -0.348014
 H     -4.595389  -1.264360   0.454610
 C     -3.267417  -2.927288  -0.332529
 H     -2.543490  -3.080199   0.478231
 C     -3.285731  -3.635688  -1.584636
 H     -2.582094  -4.420750  -1.892518
 C     -4.373197  -3.118410  -2.374714
 H     -4.639057  -3.434539  -3.392224
 C     -5.030243  -2.090745  -1.608891
 H     -5.885974  -1.487351  -1.939891
 C     -0.512710   2.215269  -0.344835
 C     -0.145980   3.039778   0.749635
 H     -0.131728   2.627231   1.771064
 C      0.196274   4.382671   0.555934
 H      0.470966   5.034819   1.396078
 C      0.187711   4.900261  -0.751614
 C     -0.155052   4.107703  -1.858151
 H     -0.153581   4.550529  -2.863472
 C     -0.503373   2.765129  -1.647833
```

```
 H     -0.790053   2.148031  -2.512633
 C     -2.177678   0.527753   1.250800
 C     -2.257238  -0.411075   2.303105
 H     -1.503141  -1.204534   2.394293
 C     -3.295183  -0.348953   3.245113
 H     -3.368649  -1.072183   4.068557
 C     -4.254005   0.668280   3.125813
 C     -4.200581   1.622596   2.097424
 H     -4.967671   2.406874   2.041888
 C     -3.156227   1.547733   1.165592
 H     -3.103146   2.305930   0.369807
 C      0.109635  -2.110913  -2.159554
 H     -0.159588  -2.852312  -1.381429
 C      1.756911  -0.257631  -2.420377
 H      2.317387  -0.644623  -3.298439
 H      0.889675   0.310913  -2.826551
 C      2.617883   0.686389  -1.626843
 C      3.089603   1.592149   0.472974
 H      2.891477   1.574985   1.554013
 C      4.017995   2.487154  -0.069245
 H      4.546735   3.185362   0.595869
 C      4.259714   2.463850  -1.452805
 C      3.551433   1.541898  -2.236726
 H      3.706365   1.483109  -3.324561
 C      0.542612  -2.896444  -3.409040
 H     -0.258419  -3.595900  -3.720601
 H      0.768623  -2.235921  -4.270722
 H      1.443861  -3.500104  -3.179876
 C      1.221183  -0.186191   2.121690
 C      0.285454  -2.179384   0.983700
 H      2.056348  -2.028525  -1.445201
 H      2.468762  -1.624573   0.657946
 C      3.630948  -2.635354   0.150222
 C      4.746190  -1.848270   0.790703
 C      3.294903  -3.747516   1.178041
 C      4.864026  -2.203554   2.153522
 C      5.633751  -0.941615   0.188804
 C      3.791898  -3.208911   2.537050
 C      5.881991  -1.635661   2.937078
 C      6.654128  -0.377194   0.975603
 H      5.530888  -0.697053  -0.880682
 C      6.773855  -0.721045   2.339741
 H      5.993050  -1.905778   4.000210
 H      7.369730   0.330427   0.526961
 H      7.581228  -0.275482   2.943196
 O      3.480132  -2.760844  -1.102269
 H      4.169137  -3.998996   3.218728
 H      2.967432  -2.688567   3.076024
 H      2.232735  -4.053339   1.159883
 H      3.902874  -4.627664   0.866161
 H      4.989381   3.147769  -1.912328
 N     -5.353457   0.741580   4.118511
 N      0.548055   6.317020  -0.966228
 O      0.848381   6.993851   0.025109
 O      0.530498   6.749922  -2.125264
 O     -5.375043  -0.105782   5.017328
 O     -6.186091   1.645486   3.989625
---------------------------------------
Mn27/TS-iii
Frequencies, energies and thermodynamic properties:
 Lowest Vibrational Mode (1/cm) =          -607.2198
 2nd Lowest Vibrational Mode (1/cm) =                     19.7277
 E(RB-P86) (a.u.) =                                     -4661.31674448
 Thermal correction to Enthalpy (a.u.) =                  0.566996
 Thermal correction to Gibbs Free Energy (a.u.) =         0.449516
 Total Entropy (cal/Kmol) =                               247.258
 E(RPBE1PBE) (a.u.) =                                   -4660.65741801
Optimised cartesian coordinates (Angstrom):
 Fe-2.581685  -1.585248  -1.466596
 Mn0.718799  -1.119309   2.039285
 P      0.073297   0.088665   0.237427
 O      0.934728   1.159095   3.914202
 O     -2.004823  -1.597751   3.048594
 N      0.847297  -2.834205   0.863658
 N      2.738078  -1.096536   1.556744
 C     -0.668846  -0.964675  -1.062269
 C     -0.755045  -2.425726  -1.002659
 C     -1.208579  -2.866795  -2.300645
 H     -1.398604  -3.907987  -2.590287
 C     -1.417552  -1.721776  -3.145195
 H     -1.787166  -1.740312  -4.179230
 C     -1.096368  -0.545096  -2.387162
 H     -1.154184   0.487820  -2.753703
 C     -3.853838  -1.326751   0.138586
 H     -3.550728  -1.159760   1.179758
```

```
 C     -4.070750  -2.609917  -0.473497
 H     -3.961850  -3.585898   0.018135
 C     -4.430641  -2.391978  -1.851252
 H     -4.639196  -3.172412  -2.595204
 C     -4.439085  -0.971010  -2.089789
 H     -4.657428  -0.479291  -3.047199
 C     -4.079545  -0.311933  -0.861214
 H     -3.985076   0.771802  -0.715020
 C      1.484002   0.941713  -0.628858
 C      2.085954   2.048545   0.021170
 H      1.673581   2.423730   0.971397
 C      3.203125   2.685381  -0.531449
 H      3.674221   3.549791  -0.044193
 C      3.729128   2.196728  -1.740000
 C      3.165294   1.094540  -2.401204
 H      3.604526   0.745187  -3.345524
 C      2.042422   0.469527  -1.838143
 H      1.590519  -0.388065  -2.358784
 C     -1.111258   1.524354   0.405712
 C     -1.936636   1.633289   1.546886
 H     -1.874761   0.886928   2.350055
 C     -2.848355   2.691775   1.677236
 H     -3.494539   2.788194   2.560174
 C     -2.923935   3.646373   0.652337
 C     -2.112578   3.574181  -0.491087
 H     -2.197300   4.345527  -1.268566
 C     -1.205220   2.512123  -0.604974
 H     -0.555907   2.466333  -1.492097
 C     -0.364008  -3.310041   0.192335
 H     -1.177880  -3.234317   0.943368
 C      2.042519  -2.863431   0.044976
 H      2.421407  -3.898650  -0.144950
 H      1.877079  -2.430807  -0.983437
 C      3.142464  -2.053444   0.674596
 C      3.678686  -0.300199   2.128510
 C      5.043913  -0.419994   1.851244
 C      5.467974  -1.410767   0.948135
 C      4.502169  -2.236463   0.359159
 C     -0.277110  -4.797825  -0.211452
 H     -1.262049  -5.175504  -0.554698
 H      0.452030  -4.972033  -1.029746
 H      0.034059  -5.401312   0.665163
 C      0.849587   0.250679   3.165818
 C     -0.937938  -1.393109   2.583737
 H      1.083125  -2.704110   2.450554
 H      1.209024  -2.232613   3.230443
 H      3.315219   0.454520   2.840678
 H      5.758954   0.253478   2.345861
 H      4.787484  -3.029145  -0.348760
 H      6.535419  -1.539385   0.711809
 N      4.909609   2.863153  -2.330934
 N     -3.884270   4.769306   0.783109
 O     -4.581143   4.815219   1.802152
 O     -3.933330   5.594130  -0.135524
 O      5.359544   2.409972  -3.390370
 O      5.382488   3.837557  -1.733253
---------------------------------------
Mn28/i
Frequencies, energies and thermodynamic properties:
 Lowest Vibrational Mode (1/cm) =                        19.9928
 2nd Lowest Vibrational Mode (1/cm) =                    27.1353
 E(RB-P86) (a.u.) =                                     -4212.11702668
 Thermal correction to Enthalpy (a.u.) =                 0.513691
 Thermal correction to Gibbs Free Energy (a.u.) =        0.412795
 Total Entropy (cal/Kmol) =                              212.353
 E(RPBE1PBE) (a.u.) =                                   -4211.43667293
Optimised cartesian coordinates (Angstrom):
 Fe2.926517  -1.108684   0.632736
 Mn-1.201337  -0.391492  -1.619275
 P     -0.181134   0.592454   0.048632
 O     -2.170844   2.050797  -3.000211
 O      1.074453  -0.414995  -3.475483
 N     -0.922437  -2.142267  -0.948470
 N     -3.085434  -0.753852  -0.843181
 C      0.951796  -0.596903   0.883489
 C      1.071084  -2.005904   0.525476
 C      1.888830  -2.640490   1.528519
 H      2.178175  -3.700649   1.533537
 C      2.282498  -1.651797   2.498104
 H      2.924964  -1.823162   3.372551
 C      1.714993  -0.390886   2.102287
 H      1.832725   0.560485   2.636953
 C      3.664434  -0.567253  -1.214505
 H      3.059168  -0.357553  -2.106063
 C      4.172299  -1.856233  -0.826043
```

```
 H      4.027758  -2.798721  -1.371292
 C      4.881292  -1.700714   0.418854
 H      5.368160  -2.502978   0.989460
 C      4.813321  -0.312486   0.798890
 H      5.240462   0.128283   1.709705
 C      4.059437   0.387799  -0.208240
 H      3.810683   1.456955  -0.203771
 C     -1.371914   1.059918   1.398267
 C     -2.184558   2.204164   1.212699
 H     -2.045163   2.837937   0.321608
 C     -3.166102   2.544046   2.157750
 H     -3.783112   3.443890   2.004332
 C     -3.363262   1.737352   3.295538
 H     -4.134266   2.003520   4.035928
 C     -2.572048   0.590437   3.479535
 H     -2.719710  -0.046861   4.366159
 C     -1.581639   0.251749   2.537518
 H     -0.959630  -0.641620   2.701396
 C      0.808732   2.155318  -0.137268
 C      1.274908   2.554076  -1.408491
 H      1.030180   1.954399  -2.296826
 C      2.057289   3.714899  -1.550311
 H      2.412389   4.013155  -2.549563
 C      2.380307   4.493346  -0.424708
 H      2.990745   5.403439  -0.537014
 C      1.911654   4.108988   0.845377
 H      2.152029   4.717633   1.731692
 C      1.128014   2.950881   0.988367
 H      0.751733   2.674076   1.985721
 C      0.406764  -2.680043  -0.674838
 H      1.037720  -2.545608  -1.576324
 C     -1.936461  -2.726208  -0.093530
 H     -2.120172  -3.803272  -0.335872
 H     -1.625101  -2.729597   0.985843
 C     -3.212778  -1.951938  -0.201656
 C     -4.192336   0.032831  -0.955990
 C     -5.442215  -0.342707  -0.460202
 C     -5.576462  -1.585616   0.189281
 C     -4.442595  -2.397004   0.316662
 C     -1.775184   1.079072  -2.448669
 C      0.198446  -0.390681  -2.675050
 H     -4.494809  -3.375862   0.817227
 H     -6.300252   0.333901  -0.586669
 H     -4.056329   0.992871  -1.474176
 H     -6.549221  -1.912477   0.587486
 H      0.366817  -3.777573  -0.476328
---------------------------------------
Mn28/ii
Frequencies, energies and thermodynamic properties:
 Lowest Vibrational Mode (1/cm) =                         12.0023
 2nd Lowest Vibrational Mode (1/cm) =                     15.8197
 E(RB-P86) (a.u.) =                                      -4367.05060113
 Thermal correction to Enthalpy (a.u.) =                  0.598316
 Thermal correction to Gibbs Free Energy (a.u.) =         0.481232
 Total Entropy (cal/Kmol) =                               246.425
 E(RPBE1PBE) (a.u.) =                                    -4366.37983909
Optimised cartesian coordinates (Angstrom):
 Fe-2.938051  -1.215579  -1.089793
 Mn1.278580  -0.431287   0.974620
 P     -0.317398   0.858818   0.203967
 O      1.846728   1.105918   3.443793
 O     -0.454542  -2.157383   2.599357
 N      1.316339  -1.576058  -0.577268
 N      2.939346   0.349297   0.017875
 C     -1.267135  -0.021434  -1.103476
 C     -0.944489  -1.354700  -1.601342
 C     -1.783988  -1.603302  -2.746421
 H     -1.791611  -2.529747  -3.337381
 C     -2.623689  -0.454892  -2.964034
 H     -3.385418  -0.349623  -3.748447
 C     -2.315719   0.519095  -1.951886
 H     -2.787033   1.504774  -1.847000
 C     -3.365056  -1.949569   0.789040
 H     -2.660234  -2.004666   1.628501
 C     -3.565717  -2.967737  -0.207473
 H     -3.045410  -3.933752  -0.257778
 C     -4.549457  -2.487832  -1.144511
 H     -4.907290  -3.021488  -2.035242
 C     -4.958845  -1.171610  -0.724558
 H     -5.684408  -0.527465  -1.239249
 C     -4.225366  -0.837176   0.468526
 H     -4.293369   0.105362   1.027167
 C      0.373443   2.338198  -0.684662
 C      0.902990   3.397579   0.090919
 H      0.832855   3.362187   1.190444
```

```
 C      1.513564   4.499647  -0.529057
 H      1.911694   5.321309   0.087504
 C      1.618798   4.553937  -1.932655
 H      2.099002   5.418130  -2.418422
 C      1.108929   3.499610  -2.709438
 H      1.186660   3.533893  -3.807908
 C      0.488948   2.396769  -2.090950
 H      0.079726   1.585578  -2.712428
 C     -1.602079   1.646197   1.292593
 C     -1.851941   1.141968   2.586946
 H     -1.266071   0.295537   2.971959
 C     -2.852511   1.713095   3.394699
 H     -3.033997   1.308468   4.403037
 C     -3.613075   2.796522   2.920839
 H     -4.394754   3.244216   3.554876
 C     -3.363865   3.312715   1.635583
 H     -3.948075   4.167562   1.259281
 C     -2.362668   2.745538   0.828600
 H     -2.165394   3.174146  -0.166436
 C      0.123441  -2.288453  -1.037864
 H     -0.288833  -2.865992  -0.186251
 C      2.155233  -1.153402  -1.689201
 H      2.639772  -2.018234  -2.206688
 H      1.554386  -0.643144  -2.488331
 C      3.192796  -0.185067  -1.211992
 C      3.815773   1.265228   0.515244
 C      4.960861   1.668299  -0.174803
 C      5.229037   1.109477  -1.439221
 C      4.330867   0.169926  -1.959311
 C      1.614805   0.494131   2.457022
 C      0.188663  -1.441371   1.907961
 H      2.299617  -2.801672   0.418537
 O      2.790028  -3.455198   0.997548
 C      4.132722  -3.546950   0.546376
 H      4.620940  -2.540499   0.494359
 H      4.189596  -3.973772  -0.488518
 H      4.498000  -0.294852  -2.942725
 H      5.634670   2.409383   0.279597
 H      3.580499   1.676727   1.507193
 C      4.924350  -4.439744   1.496045
 H      5.977200  -4.543575   1.161139
 H      4.925888  -4.017458   2.523037
 H      4.474050  -5.453633   1.545664
 H      6.125686   1.402438  -2.006547
 H      0.382222  -3.032689  -1.828391
---------------------------------------
Mn28/iii
Frequencies, energies and thermodynamic properties:
 Lowest Vibrational Mode (1/cm) =                        18.2754
 2nd Lowest Vibrational Mode (1/cm) =                    21.0198
 E(RB-P86) (a.u.) =                                    -4368.22400437
 Thermal correction to Enthalpy (a.u.) =                 0.614439
 Thermal correction to Gibbs Free Energy (a.u.) =        0.498800
 Total Entropy (cal/Kmol) =                              243.384
 E(RPBE1PBE) (a.u.) =                                  -4367.54981907
Optimised cartesian coordinates (Angstrom):
 Fe-2.877944  -1.333363  -1.052047
 Mn1.306171  -0.337211   1.238598
 P     -0.302877   0.843714   0.142206
 O      1.379169   1.681249   3.402840
 O     -0.543532  -1.997646   2.815395
 N      1.400535  -1.669533  -0.359368
 N      2.879126   0.482306   0.164254
 C     -1.206367  -0.137987  -1.111062
 C     -0.863051  -1.507912  -1.469805
 C     -1.657738  -1.859959  -2.620028
 H     -1.640983  -2.835478  -3.125946
 C     -2.492069  -0.740968  -2.971246
 H     -3.224448  -0.711045  -3.789355
 C     -2.226403   0.321344  -2.039008
 H     -2.703331   1.309909  -2.038377
 C     -3.385983  -1.905643   0.862943
 H     -2.721136  -1.881395   1.735434
 C     -3.534137  -3.008569  -0.048487
 H     -3.005938  -3.969819   0.009139
 C     -4.476541  -2.621334  -1.067403
 H     -4.789483  -3.233689  -1.923759
 C     -4.913116  -1.277786  -0.782617
 H     -5.618266  -0.688188  -1.383805
 C     -4.237354  -0.833651   0.408553
 H     -4.337452   0.152387   0.880368
 C      0.386783   2.276000  -0.825946
 C      0.902604   3.378246  -0.102757
 H      0.839781   3.392950   0.997462
 C      1.486068   4.464202  -0.775568
```

```
 H      1.875193   5.317955  -0.198216
 C      1.574098   4.461537  -2.180845
 H      2.032011   5.313224  -2.708389
 C      1.074063   3.366581  -2.906089
 H      1.136663   3.356071  -4.005960
 C      0.483475   2.279009  -2.234098
 H      0.084113   1.435076  -2.817192
 C     -1.645764   1.698465   1.112307
 C     -1.991152   1.266932   2.411117
 H     -1.444507   0.439634   2.884034
 C     -3.040869   1.884879   3.115387
 H     -3.294319   1.535534   4.128854
 C     -3.759308   2.943358   2.532600
 H     -4.579566   3.427799   3.085682
 C     -3.418321   3.385915   1.241232
 H     -3.969449   4.219592   0.777653
 C     -2.367046   2.772338   0.538571
 H     -2.100618   3.144421  -0.462882
 C      0.193908  -2.372799  -0.795842
 H     -0.242326  -2.887748   0.085025
 C      2.106591  -1.092530  -1.497645
 H      2.585172  -1.873791  -2.140707
 H      1.421425  -0.535599  -2.194936
 C      3.135519  -0.100753  -1.040739
 C      3.730130   1.437414   0.619857
 C      4.865152   1.841979  -0.089920
 C      5.142926   1.234106  -1.327021
 C      4.265157   0.251775  -1.803134
 C      1.364360   0.870699   2.544473
 C      0.148039  -1.310620   2.148571
 H      2.622541  -0.944408   2.182777
 H      2.476075  -1.602829   1.698379
 H      2.315248  -2.832577   0.291141
 O      2.865112  -3.533040   0.837533
 C      4.182206  -3.597986   0.325190
 H      4.190501  -3.909966  -0.752156
 H      4.691923  -2.599095   0.356576
 H      4.442939  -0.251245  -2.765479
 H      5.520711   2.618366   0.330751
 H      3.487741   1.884064   1.595211
 C      5.003011  -4.599527   1.134333
 H      4.533916  -5.605601   1.097481
 H      6.037658  -4.683668   0.740650
 H      5.061284  -4.291971   2.200014
 H      6.032064   1.521037  -1.909318
 H      0.463424  -3.182355  -1.520552
---------------------------------------
Mn28/iv
Frequencies, energies and thermodynamic properties:
 Lowest Vibrational Mode (1/cm) =                        11.6143
 2nd Lowest Vibrational Mode (1/cm) =                    25.9910
 E(RB-P86) (a.u.) =                                     -4368.25211524
 Thermal correction to Enthalpy (a.u.) =                 0.619122
 Thermal correction to Gibbs Free Energy (a.u.) =        0.503528
 Total Entropy (cal/Kmol) =                              243.289
 E(RPBE1PBE) (a.u.) =                                   -4367.57570360
Optimised cartesian coordinates (Angstrom):
 Fe-2.961369  -1.207020  -1.094505
 Mn1.358346  -0.471428   1.017848
 P     -0.322210   0.840690   0.205458
 O      1.734776   1.126938   3.456255
 O     -0.253925  -2.405213   2.539837
 N      1.340529  -1.629803  -0.793789
 N      2.873547   0.438286  -0.037330
 C     -1.319438   0.027410  -1.110512
 C     -0.994642  -1.276993  -1.681626
 C     -1.869305  -1.486500  -2.810702
 H     -1.881747  -2.383674  -3.445112
 C     -2.737159  -0.347260  -2.940361
 H     -3.530490  -0.222414  -3.689544
 C     -2.407338   0.583181  -1.895447
 H     -2.891731   1.552911  -1.723242
 C     -3.286373  -2.055135   0.754370
 H     -2.540821  -2.143534   1.555469
 C     -3.515704  -3.018377  -0.290160
 H     -2.981011  -3.968801  -0.421832
 C     -4.551886  -2.506259  -1.150260
 H     -4.941357  -2.994853  -2.053470
 C     -4.965440  -1.226096  -0.634847
 H     -5.726341  -0.568609  -1.076368
 C     -4.182834  -0.945692   0.540470
 H     -4.242416  -0.039181   1.156530
 C      0.268855   2.401457  -0.624843
 C      0.863234   3.392124   0.194743
 H      0.892115   3.252440   1.287973
```

```
 C      1.410204   4.556454  -0.368926
 H      1.858873   5.320760   0.285778
 C      1.388861   4.745509  -1.764380
 H      1.819368   5.657830  -2.206903
 C      0.816230   3.761230  -2.587981
 H      0.794270   3.898901  -3.681048
 C      0.259499   2.596995  -2.023383
 H     -0.199456   1.844348  -2.683232
 C     -1.607041   1.537578   1.364618
 C     -1.836051   0.925839   2.615962
 H     -1.238694   0.052888   2.915646
 C     -2.828268   1.418616   3.483586
 H     -2.992740   0.928761   4.456626
 C     -3.602826   2.532622   3.114250
 H     -4.377537   2.919923   3.795047
 C     -3.376398   3.155324   1.872701
 H     -3.972520   4.033564   1.577107
 C     -2.383466   2.664610   1.006718
 H     -2.205421   3.174610   0.046937
 C      0.064662  -2.258242  -1.225636
 H     -0.292276  -2.861625  -0.368592
 C      1.996480  -0.873208  -1.879783
 H      2.399376  -1.540718  -2.674095
 H      1.228993  -0.230543  -2.367371
 C      3.071067   0.020500  -1.322080
 C      3.767613   1.317355   0.487858
 C      4.878920   1.790695  -0.219205
 C      5.095664   1.341144  -1.532253
 C      4.172047   0.441113  -2.085887
 C      1.573446   0.488942   2.469438
 C      0.344521  -1.602672   1.903009
 H      2.563241  -1.399654   1.520977
 H      2.848544  -2.768101   1.093795
 H      1.972968  -2.412172  -0.516904
 O      3.021452  -3.584691   0.528036
 C      4.433551  -3.674168   0.313555
 H      4.580962  -4.397531  -0.517950
 H      4.848261  -2.697565  -0.034971
 H      4.293290   0.061547  -3.111728
 H      5.566091   2.499049   0.266773
 H      3.581620   1.638398   1.522837
 C      5.185266  -4.146939   1.557409
 H      4.793723  -5.125799   1.904600
 H      6.269207  -4.261796   1.344236
 H      5.078426  -3.419002   2.389575
 H      5.965267   1.683265  -2.113707
 H      0.257431  -2.968351  -2.062472
---------------------------------------
Mn28/v
Frequencies, energies and thermodynamic properties:
 Lowest Vibrational Mode (1/cm) =                          25.1289
 2nd Lowest Vibrational Mode (1/cm) =                      32.2381
 E(RB-P86) (a.u.) =                                       -4213.31362842
 Thermal correction to Enthalpy (a.u.) =                   0.534674
 Thermal correction to Gibbs Free Energy (a.u.) =          0.433717
 Total Entropy (cal/Kmol) =                                212.482
 E(RPBE1PBE) (a.u.) =                                     -4212.63326173
Optimised cartesian coordinates (Angstrom):
 Fe2.935288  -1.040488   0.681881
 Mn-1.244173  -0.427333  -1.718499
 P     -0.212593   0.563985   0.056060
 O     -2.117313   2.073556  -2.990790
 O      1.006523  -0.604319  -3.602773
 N     -0.878701  -2.366460  -0.873125
 N     -3.038852  -0.786482  -0.788178
 C      0.953651  -0.558606   0.937704
 C      1.109930  -1.976663   0.621495
 C      1.946319  -2.561281   1.642755
 H      2.260137  -3.613783   1.679404
 C      2.323471  -1.533030   2.574305
 H      2.978482  -1.661192   3.446528
 C      1.720968  -0.301226   2.143186
 H      1.820437   0.670747   2.643327
 C      3.638023  -0.536104  -1.188134
 H      3.017502  -0.359979  -2.076845
 C      4.172345  -1.806581  -0.773051
 H      4.036018  -2.765727  -1.290739
 C      4.897207  -1.606224   0.455964
 H      5.404658  -2.385222   1.040641
 C      4.813021  -0.209682   0.799267
 H      5.245932   0.262154   1.691551
 C      4.033316   0.451473  -0.214193
 H      3.767174   1.516071  -0.234325
 C     -1.368884   1.104702   1.415479
 C     -2.249634   2.177699   1.132680
```

```
 H     -2.173754   2.707309   0.168587
 C     -3.215437   2.580438   2.069492
 H     -3.884008   3.424500   1.835349
 C     -3.332420   1.906920   3.300938
 H     -4.091296   2.221107   4.035059
 C     -2.475894   0.829327   3.584437
 H     -2.559154   0.294464   4.544320
 C     -1.500704   0.430429   2.649471
 H     -0.828318  -0.406164   2.895949
 C      0.780232   2.124512  -0.187915
 C      1.265415   2.452182  -1.472202
 H      1.035709   1.795919  -2.324155
 C      2.045689   3.606243  -1.670579
 H      2.416081   3.846817  -2.679965
 C      2.347634   4.451558  -0.587969
 H      2.956043   5.356709  -0.743778
 C      1.860323   4.139004   0.694841
 H      2.084659   4.799373   1.547911
 C      1.079029   2.986894   0.892665
 H      0.688669   2.765813   1.898604
 C      0.519679  -2.748785  -0.539507
 H      1.111365  -2.598392  -1.463066
 C     -1.826149  -2.622771   0.232040
 H     -1.980827  -3.709705   0.412004
 H     -1.378010  -2.206253   1.161863
 C     -3.130228  -1.916173  -0.024748
 C     -4.173981  -0.061397  -0.977436
 H     -4.073036   0.844386  -1.592364
 C     -5.415678  -0.431278  -0.447820
 H     -6.294618   0.200774  -0.643685
 C     -5.512699  -1.607353   0.314163
 C     -4.344357  -2.357006   0.524335
 H     -4.363128  -3.284458   1.116617
 C     -1.753452   1.073279  -2.464359
 C      0.139172  -0.509414  -2.797543
 H     -1.161755  -2.934779  -1.681991
 H     -1.918091  -1.245462  -2.897820
 H     -6.475213  -1.934631   0.735682
 H      0.567818  -3.833987  -0.292919
---------------------------------------
Mn28/vi_R
Frequencies, energies and thermodynamic properties:
 Lowest Vibrational Mode (1/cm) =                         13.9380
 2nd Lowest Vibrational Mode (1/cm) =                     18.4353
 E(RB-P86) (a.u.) =                                     -4636.02676580
 Thermal correction to Enthalpy (a.u.) =                  0.687069
 Thermal correction to Gibbs Free Energy (a.u.) =         0.563237
 Total Entropy (cal/Kmol) =                               260.627
 E(RPBE1PBE) (a.u.) =                                   -4635.34136680
Optimised cartesian coordinates (Angstrom):
 Fe-3.093131  -1.853519  -1.239348
 Mn0.917615   0.275146   0.264201
 P     -1.237357   0.674499   0.499681
 O      1.637980   0.883342   3.073151
 O      0.787497  -2.565547   1.012717
 N      0.788081  -0.022993  -1.667526
 N      1.539081   2.127147  -0.405994
 C     -2.191256  -0.039308  -0.899411
 C     -1.588448  -0.700415  -2.051655
 C     -2.635582  -0.926270  -3.016350
 H     -2.505194  -1.420915  -3.988910
 C     -3.874583  -0.426371  -2.481595
 H     -4.856314  -0.473242  -2.972024
 C     -3.609689   0.113641  -1.175453
 H     -4.351504   0.570952  -0.508237
 C     -2.404028  -3.339755   0.012899
 H     -1.457270  -3.320298   0.567619
 C     -2.587472  -3.848285  -1.320353
 H     -1.807769  -4.286354  -1.957956
 C     -3.968257  -3.657073  -1.684973
 H     -4.424041  -3.920080  -2.648976
 C     -4.639289  -3.031314  -0.573968
 H     -5.696463  -2.735235  -0.543297
 C     -3.672854  -2.832492   0.474458
 H     -3.861527  -2.361866   1.448043
 C     -1.611049   2.492975   0.400530
 C     -1.274553   3.303426   1.511396
 H     -0.865139   2.841388   2.424602
 C     -1.463224   4.693997   1.463022
 H     -1.206004   5.310449   2.339105
 C     -1.976951   5.298480   0.299201
 H     -2.124037   6.389482   0.261342
 C     -2.299811   4.502818  -0.813273
 H     -2.702310   4.967345  -1.727597
 C     -2.118788   3.107054  -0.765148
```

```
 H     -2.391142   2.494647  -1.638383
 C     -2.182423   0.190183   2.026301
 C     -1.687741  -0.806132   2.895064
 H     -0.720470  -1.286316   2.692124
 C     -2.427005  -1.198706   4.026059
 H     -2.025390  -1.976556   4.694640
 C     -3.668031  -0.599594   4.304585
 H     -4.244920  -0.906159   5.191508
 C     -4.165273   0.400860   3.449040
 H     -5.132447   0.882762   3.663481
 C     -3.426476   0.796936   2.320953
 H     -3.819464   1.596250   1.673478
 C     -0.112508  -1.040708  -2.220612
 H      0.117265  -2.005992  -1.726939
 C      0.808556   1.179412  -2.496442
 H      1.323704   1.007807  -3.473237
 H     -0.228975   1.507252  -2.767452
 C      1.461941   2.305673  -1.756395
 C      2.080772   3.123888   0.346538
 H      2.125095   2.947754   1.430724
 C      2.568510   4.309935  -0.206995
 H      2.999097   5.078382   0.451489
 C      2.501525   4.490146  -1.601600
 C      1.941946   3.471270  -2.381800
 H      1.868177   3.566323  -3.475602
 C      1.351825   0.641688   1.950559
 C      0.776744  -1.423103   0.700237
 H      2.301834  -0.608735  -1.904215
 H      3.027021  -0.264110  -0.010097
 C      3.859069  -0.584099  -0.740074
 C      4.601684  -1.730136  -0.066439
 C      4.928184   0.546199  -0.755913
 C      5.735862  -1.246818   0.622831
 C      4.290247  -3.098157  -0.074858
 C      5.846040   0.260041   0.457827
 H      5.498307   0.429729  -1.703788
 C      6.567243  -2.136712   1.323807
 C      5.125919  -3.990568   0.625620
 H      3.409974  -3.461591  -0.628683
 H      6.891072   0.607714   0.317130
 C      6.255308  -3.512043   1.321451
 H      7.458693  -1.770807   1.860084
 H      4.900855  -5.069533   0.625988
 H      6.904770  -4.220477   1.860954
 O      3.291777  -0.888093  -1.976181
 H      4.484306   1.562006  -0.751914
 H      5.463722   0.772732   1.370584
 H      2.882322   5.410567  -2.070061
 H      0.080885  -1.189966  -3.309983
---------------------------------------
Mn28/vi_S
Frequencies, energies and thermodynamic properties:
 Lowest Vibrational Mode (1/cm) =                          15.3495
 2nd Lowest Vibrational Mode (1/cm) =                      19.9852
 E(RB-P86) (a.u.) =                                      -4636.02703182
 Thermal correction to Enthalpy (a.u.) =                   0.687126
 Thermal correction to Gibbs Free Energy (a.u.) =          0.564074
 Total Entropy (cal/Kmol) =                                258.984
 E(RPBE1PBE) (a.u.) =                                    -4635.34219426
Optimised cartesian coordinates (Angstrom):
 Fe-3.557677  -1.370816  -1.168100
 Mn0.795905  -0.196082   0.405520
 P     -1.176128   0.789309   0.404922
 O      1.504378   0.567209   3.179265
 O     -0.143760  -2.771725   1.465569
 N      0.689577  -0.700601  -1.485347
 N      1.941799   1.314552  -0.415614
 C     -2.214812   0.171534  -0.979401
 C     -1.759140  -0.779750  -1.987405
 C     -2.780913  -0.856616  -3.001306
 H     -2.742566  -1.499286  -3.891820
 C     -3.862091   0.020852  -2.637898
 H     -4.794854   0.165178  -3.199707
 C     -3.523511   0.651030  -1.390439
 H     -4.144672   1.376387  -0.849496
 C     -3.381357  -2.750970   0.353255
 H     -2.512283  -2.868079   1.012716
 C     -3.572802  -3.414337  -0.908892
 H     -2.878973  -4.125926  -1.376479
 C     -4.821845  -2.961313  -1.466102
 H     -5.244954  -3.263746  -2.433435
 C     -5.403982  -2.018248  -0.545026
 H     -6.348963  -1.477145  -0.688138
 C     -4.512863  -1.885580   0.578266
 H     -4.657946  -1.228741   1.445814
```

```
 C     -1.035175   2.612176   0.067237
 C     -0.544363   3.444995   1.101541
 H     -0.318922   3.016594   2.091896
 C     -0.346685   4.817251   0.879118
 H      0.026540   5.454402   1.696695
 C     -0.622052   5.376459  -0.383938
 H     -0.465928   6.452836  -0.558285
 C     -1.096188   4.554191  -1.420210
 H     -1.314689   4.983390  -2.411083
 C     -1.302718   3.178958  -1.198061
 H     -1.690350   2.550293  -2.014325
 C     -2.296232   0.780571   1.889735
 C     -2.132343  -0.177873   2.912647
 H     -1.316960  -0.912370   2.856548
 C     -3.009402  -0.205696   4.012546
 H     -2.865967  -0.959288   4.803039
 C     -4.059361   0.724610   4.105466
 H     -4.744255   0.703034   4.967939
 C     -4.224736   1.690302   3.095328
 H     -5.038552   2.429661   3.163659
 C     -3.347100   1.722092   1.998095
 H     -3.477052   2.497450   1.227002
 C     -0.422434  -1.512275  -1.991986
 H     -0.485848  -2.430147  -1.373891
 C      1.068502   0.340361  -2.437711
 H      1.555100  -0.081291  -3.350921
 H      0.170658   0.895343  -2.816123
 C      1.973648   1.335384  -1.779221
 C      2.705073   2.215059   0.261589
 H      2.656113   2.168945   1.358844
 C      3.518364   3.151708  -0.380394
 H      4.114849   3.853588   0.220461
 C      3.559362   3.169041  -1.787182
 C      2.778079   2.244486  -2.490865
 H      2.778073   2.218626  -3.590943
 C      1.224232   0.263938   2.070230
 C      0.170663  -1.718877   1.021312
 H      2.019383  -1.658428  -1.540492
 H      2.703153  -1.261266   0.359813
 C      3.394438  -1.988780  -0.207580
 C      3.457426  -3.299744   0.628291
 C      4.539235  -3.043106   1.705920
 O      2.937027  -2.129404  -1.515681
 H      4.193792   3.890905  -2.323912
 C      4.807515  -1.441216  -0.067781
 C      5.460693  -2.038622   1.033339
 C      5.459033  -0.487183  -0.864079
 C      6.778794  -1.672586   1.353816
 C      6.782248  -0.122978  -0.544012
 H      4.942502  -0.045450  -1.731673
 C      7.435640  -0.710131   0.559040
 H      7.300768  -2.137371   2.206843
 H      7.313312   0.619311  -1.161839
 H      8.472647  -0.421769   0.796104
 H      5.065362  -3.963012   2.037066
 H      4.086258  -2.593330   2.619594
 H      3.792881  -4.101920  -0.065455
 H      2.472403  -3.595640   1.039905
 H     -0.222027  -1.849105  -3.037341
---------------------------------------
Mn28/viii
Frequencies, energies and thermodynamic properties:
 Lowest Vibrational Mode (1/cm) =                          27.4434
 2nd Lowest Vibrational Mode (1/cm) =                      29.9672
 E(RB-P86) (a.u.) =                                      -4213.28046669
 Thermal correction to Enthalpy (a.u.) =                   0.529760
 Thermal correction to Gibbs Free Energy (a.u.) =          0.428550
 Total Entropy (cal/Kmol) =                                213.015
 E(RPBE1PBE) (a.u.) =                                    -4212.60115017
Optimised cartesian coordinates (Angstrom):
 Fe2.944604  -1.046324   0.658580
 Mn-1.220315  -0.399887  -1.775393
 P     -0.218603   0.504050   0.041626
 O     -2.013751   2.268727  -2.793772
 O      1.171860  -0.368451  -3.488971
 N     -0.866843  -2.265849  -1.006169
 N     -3.029989  -0.755360  -0.821240
 C      0.941236  -0.638273   0.883423
 C      1.131672  -2.030102   0.497747
 C      1.962392  -2.642011   1.504030
 H      2.300861  -3.687768   1.495056
 C      2.295755  -1.655392   2.498485
 H      2.932133  -1.812368   3.380142
 C      1.676879  -0.414111   2.116955
 H      1.742075   0.531670   2.670201
```

```
 C      3.691754  -0.366182  -1.139691
 H      3.094109  -0.103826  -2.021684
 C      4.214058  -1.672181  -0.838724
 H      4.088849  -2.574944  -1.451698
 C      4.907912  -1.595415   0.422086
 H      5.401393  -2.429128   0.939535
 C      4.816311  -0.238179   0.898474
 H      5.229021   0.142800   1.842365
 C      4.061992   0.521358  -0.064641
 H      3.798500   1.584259   0.011453
 C     -1.429447   0.964629   1.378550
 C     -2.277533   2.075742   1.154982
 H     -2.173047   2.671128   0.233318
 C     -3.248007   2.436362   2.104040
 H     -3.894323   3.309144   1.918760
 C     -3.394969   1.685758   3.286316
 H     -4.155994   1.968824   4.030756
 C     -2.565406   0.573685   3.510640
 H     -2.672391  -0.019353   4.433016
 C     -1.588026   0.213517   2.562856
 H     -0.937493  -0.652972   2.757354
 C      0.738643   2.097312  -0.113849
 C      1.250475   2.516732  -1.360558
 H      1.059685   1.922626  -2.264782
 C      2.013226   3.694828  -1.462448
 H      2.402399   4.006702  -2.444695
 C      2.274179   4.471615  -0.320255
 H      2.869859   5.394702  -0.401237
 C      1.761768   4.066961   0.926257
 H      1.953018   4.672281   1.826692
 C      0.996045   2.892760   1.027997
 H      0.585436   2.602243   2.007492
 C      0.481519  -2.706029  -0.709995
 H      1.116496  -2.550313  -1.608058
 C     -1.791770  -2.644785   0.036204
 H     -1.984277  -3.751206   0.061598
 H     -1.428822  -2.412548   1.083011
 C     -3.094524  -1.914948  -0.107217
 C     -4.156363  -0.006721  -0.950575
 C     -5.382485  -0.378686  -0.391854
 C     -5.458895  -1.580971   0.335454
 C     -4.299375  -2.353146   0.475799
 C     -1.708335   1.194405  -2.405507
 C      0.249507  -0.365490  -2.748908
 H     -1.816546  -1.641210  -2.878460
 H     -2.148176  -0.969304  -3.206608
 H     -4.061950   0.921450  -1.532994
 H     -6.262222   0.266011  -0.533735
 H     -4.311591  -3.300803   1.035351
 H     -6.409513  -1.909747   0.783094
 H      0.501559  -3.809499  -0.510499
---------------------------------------
Mn28/ix
Frequencies, energies and thermodynamic properties:
 Lowest Vibrational Mode (1/cm) =                        27.5092
 2nd Lowest Vibrational Mode (1/cm) =                    29.6714
 E(RB-P86) (a.u.) =                                     -4367.06737259
 Thermal correction to Enthalpy (a.u.) =                 0.599034
 Thermal correction to Gibbs Free Energy (a.u.) =        0.489379
 Total Entropy (cal/Kmol) =                              230.788
 E(RPBE1PBE) (a.u.) =                                   -4366.39167871
Optimised cartesian coordinates (Angstrom):
 Fe-3.161434  -0.451733  -1.144110
 Mn1.340260  -1.096779   0.578470
 P     -0.066595   0.652801   0.301161
 O      2.192431  -0.176753   3.239964
 O     -0.648103  -2.867028   1.824680
 N      0.942322  -1.730540  -1.411828
 N      2.912265  -0.177112  -0.440678
 C     -1.282857   0.380124  -1.060242
 C     -1.296630  -0.783456  -1.943922
 C     -2.258952  -0.526668  -2.987567
 H     -2.509347  -1.219382  -3.803010
 C     -2.850637   0.764327  -2.762402
 H     -3.635205   1.230952  -3.373106
 C     -2.260095   1.322633  -1.576696
 H     -2.502379   2.300369  -1.141259
 C     -3.577487  -1.617668   0.503605
 H     -2.833227  -2.038567   1.191806
 C     -4.064846  -2.246658  -0.695459
 H     -3.762063  -3.230712  -1.078165
 C     -5.001078  -1.348477  -1.321850
 H     -5.532640  -1.525525  -2.266472
 C     -5.093905  -0.163674  -0.507319
 H     -5.709367   0.720020  -0.722918
```

```
 C     -4.212459  -0.328010   0.619089
 H     -4.036567   0.405909   1.416099
 C      0.782713   2.229823  -0.217918
 C      1.563008   2.907825   0.749651
 H      1.592724   2.537472   1.787561
 C      2.296572   4.054253   0.403327
 H      2.890212   4.574839   1.171819
 C      2.276026   4.535246  -0.920185
 H      2.852375   5.433711  -1.192262
 C      1.516137   3.860835  -1.891088
 H      1.492839   4.228975  -2.929364
 C      0.773439   2.715648  -1.543396
 H      0.170818   2.209702  -2.313560
 C     -1.110319   1.283921   1.713012
 C     -1.367928   0.467670   2.835428
 H     -0.932635  -0.539146   2.896390
 C     -2.185841   0.927705   3.884162
 H     -2.374392   0.276043   4.752148
 C     -2.755429   2.211749   3.828091
 H     -3.394144   2.571895   4.650213
 C     -2.496269   3.038097   2.718867
 H     -2.929635   4.049803   2.668690
 C     -1.676415   2.580337   1.672995
 H     -1.466175   3.248543   0.823428
 C     -0.437355  -2.027495  -1.838540
 H     -0.854729  -2.741302  -1.101610
 C      1.715619  -0.969789  -2.404902
 H      1.966837  -1.582053  -3.300365
 H      1.085429  -0.127396  -2.771654
 C      2.959652  -0.388977  -1.783534
 C      3.974492   0.416962   0.155409
 C      5.117858   0.813861  -0.547814
 C      5.176584   0.582226  -1.932458
 C      4.079584  -0.029404  -2.554846
 C      1.856426  -0.561141   2.169763
 C      0.116045  -2.118830   1.314431
 H      1.475453  -2.594519  -1.128715
 H      4.079741  -0.230720  -3.636763
 H      5.949818   1.289900  -0.008288
 H      3.902137   0.568764   1.242178
 H      6.063550   0.870920  -2.517067
 O      2.536732  -2.718735   0.263664
 C      2.798937  -3.728640   1.191239
 C      3.903413  -3.380456   2.203282
 H      3.125587  -4.648772   0.635013
 H      1.884466  -4.045161   1.764062
 H      4.119338  -4.240511   2.875257
 H      4.844378  -3.110954   1.676431
 H      3.608719  -2.517424   2.835710
 H     -0.440790  -2.542843  -2.826711
---------------------------------------
Mn28/x
Frequencies, energies and thermodynamic properties:
 Lowest Vibrational Mode (1/cm) =                         11.5809
 2nd Lowest Vibrational Mode (1/cm) =                     26.4963
 E(RB-P86) (a.u.) =                                     -4522.00897333
 Thermal correction to Enthalpy (a.u.) =                  0.683155
 Thermal correction to Gibbs Free Energy (a.u.) =         0.558974
 Total Entropy (cal/Kmol) =                               261.361
 E(RPBE1PBE) (a.u.) =                                   -4521.34106889
Optimised cartesian coordinates (Angstrom):
 Fe-3.123597  -1.484209  -0.961503
 Mn 1.227406  -0.187595   0.848061
 P     -0.644362   0.869609   0.122775
 O      1.368575   1.672049   3.122493
 O     -0.158258  -2.192647   2.492837
 N      1.209840  -1.412762  -0.921664
 N      2.503979   0.893677  -0.389367
 C     -1.621949  -0.089992  -1.103941
 C     -1.196065  -1.368338  -1.663093
 C     -2.106483  -1.700179  -2.732405
 H     -2.060661  -2.611763  -3.344212
 C     -3.094087  -0.660051  -2.836396
 H     -3.937287  -0.638507  -3.539757
 C     -2.805821   0.329042  -1.833878
 H     -3.378617   1.248314  -1.656965
 C     -3.272169  -2.291262   0.928923
 H     -2.489385  -2.264311   1.697811
 C     -3.433595  -3.314106  -0.070314
 H     -2.800370  -4.203082  -0.193568
 C     -4.556081  -2.955068  -0.898858
 H     -4.924731  -3.518878  -1.766248
 C     -5.090668  -1.710179  -0.408749
 H     -5.938889  -1.159760  -0.837378
 C     -4.296419  -1.297961   0.718902
```

```
 H     -4.433133  -0.380769   1.306240
 C     -0.287010   2.458749  -0.784696
 C      0.184666   3.561465  -0.032042
 H      0.261254   3.485834   1.064912
 C      0.548604   4.759510  -0.668388
 H      0.904347   5.610548  -0.065824
 C      0.460968   4.872947  -2.069261
 H      0.747532   5.812292  -2.568157
 C      0.005026   3.780130  -2.826021
 H     -0.069421   3.858750  -3.922482
 C     -0.367760   2.580644  -2.189005
 H     -0.739768   1.741255  -2.796719
 C     -1.926990   1.454498   1.344272
 C     -2.024647   0.867045   2.624115
 H     -1.321087   0.078920   2.925377
 C     -3.023344   1.276555   3.526753
 H     -3.082198   0.806882   4.521473
 C     -3.937804   2.281053   3.164510
 H     -4.718096   2.602614   3.872493
 C     -3.844313   2.879302   1.894397
 H     -4.550465   3.673072   1.602706
 C     -2.844420   2.473236   0.993736
 H     -2.773437   2.966432   0.011847
 C     -0.005415  -2.203376  -1.246539
 H     -0.235604  -2.811187  -0.349783
 C      1.705437  -0.647307  -2.084033
 H      2.149089  -1.308909  -2.861289
 H      0.842514  -0.134707  -2.566456
 C      2.687180   0.405705  -1.645673
 C      3.307855   1.897718   0.035811
 C      4.318987   2.450486  -0.759322
 C      4.522371   1.937733  -2.051055
 C      3.692942   0.898329  -2.496199
 C      1.324511   0.915458   2.212461
 C      0.348697  -1.363350   1.817856
 H      3.178866  -2.416268   0.536279
 H      1.949748  -2.126439  -0.683650
 O      3.194875  -3.245428  -0.124533
 C      4.424837  -3.242395  -0.834541
 H      4.268642  -3.771697  -1.803918
 H      4.741102  -2.200781  -1.094509
 H      3.814592   0.464256  -3.499929
 H      4.939270   3.266381  -0.360174
 H      3.130087   2.267078   1.055867
 C      5.550853  -3.935559  -0.061925
 H      5.264154  -4.977660   0.193167
 H      6.486786  -3.970118  -0.660052
 H      5.771764  -3.401477   0.886749
 H      5.315495   2.338874  -2.700509
 O      2.990937  -1.184015   1.264394
 C      3.470193  -1.367391   2.576766
 C      4.465118  -0.287056   3.021843
 H      3.986401  -2.361905   2.635609
 H      2.638482  -1.421445   3.325003
 H      4.878449  -0.520330   4.027758
 H      5.313312  -0.218108   2.307826
 H      3.980718   0.709468   3.077552
 H      0.219341  -2.919995  -2.069549
---------------------------------------
Mn28/TS-i
Frequencies, energies and thermodynamic properties:
 Lowest Vibrational Mode (1/cm) =                      -746.0556
 2nd Lowest Vibrational Mode (1/cm) =                     21.4468
 E(RB-P86) (a.u.) =                                    -4368.22177942
 Thermal correction to Enthalpy (a.u.) =                  0.610743
 Thermal correction to Gibbs Free Energy (a.u.) =         0.497518
 Total Entropy (cal/Kmol) =                               238.301
 E(RPBE1PBE) (a.u.) =                                  -4367.54418983
Optimised cartesian coordinates (Angstrom):
 Fe -2.925871  -1.231754  -1.067043
 Mn  1.316543  -0.430277   1.182198
 P     -0.273430   0.837089   0.158259
 O      1.507734   1.482550   3.425393
 O     -0.548798  -2.086446   2.745916
 N      1.346617  -1.720982  -0.473277
 N      2.895154   0.373584   0.108514
 C     -1.225582  -0.077820  -1.113151
 C     -0.927230  -1.443121  -1.529080
 C     -1.750827  -1.731610  -2.677097
 H     -1.769283  -2.687842  -3.218386
 C     -2.559562  -0.578520  -2.971325
 H     -3.305206  -0.499236  -3.773916
 C     -2.247872   0.440867  -2.006405
 H     -2.697602   1.441050  -1.960773
 C     -3.412356  -1.862942   0.834372
```

H -2.729702 -1.891396 1.692968
C -3.611271 -2.925001 -0.115366
H -3.110577 -3.902529 -0.105790
C -4.562272 -2.471907 -1.098333
H -4.909880 -3.041293 -1.970797
C -4.953269 -1.128582 -0.753303
H -5.652165 -0.495937 -1.316801
C -4.241016 -0.750457 0.439316
H -4.302174 0.219239 0.949968
C 0.439530 2.277616 -0.780577
C 1.001380 3.341712 -0.034652
H 0.955434 3.326413 1.066481
C 1.609255 4.427828 -0.685257
H 2.033615 5.251927 -0.089891
C 1.677171 4.463085 -2.091242
H 2.154468 5.314764 -2.601316
C 1.132267 3.405648 -2.839418
H 1.178752 3.424708 -3.939989
C 0.516491 2.318589 -2.189512
H 0.080861 1.505918 -2.790891
C -1.576705 1.697848 1.175180
C -1.911976 1.233448 2.465305
H -1.381613 0.375105 2.900086
C -2.930903 1.857547 3.208354
H -3.177269 1.482125 4.214225
C -3.627696 2.955236 2.673674
H -4.423818 3.444395 3.257054
C -3.295970 3.430755 1.391618
H -3.830170 4.295089 0.965879
C -2.275510 2.810755 0.650195
H -2.014978 3.208291 -0.343024
C 0.105144 -2.372688 -0.913527
H -0.323251 -2.902634 -0.038620
C 2.061289 -1.120630 -1.602786
H 2.502819 -1.891407 -2.279951
H 1.368993 -0.524074 -2.250804
C 3.124813 -0.178227 -1.116648
C 3.779469 1.287913 0.584001
C 4.920755 1.678629 -0.123957
C 5.170936 1.099899 -1.380236
C 4.258686 0.159948 -1.877893
C 1.441232 0.715050 2.530149
C 0.151909 -1.400273 2.086689
H 2.612104 -1.158559 2.019793
H 2.494354 -1.890508 1.575389
H 2.104197 -2.650827 0.096285
O 2.725193 -3.364628 0.823762
C 4.078444 -3.485490 0.449888
H 4.184044 -3.665485 -0.652623
H 4.653123 -2.540085 0.653188
H 4.412773 -0.319049 -2.856402
H 5.602953 2.421801 0.314032
H 3.559989 1.710847 1.575052
C 4.751652 -4.635837 1.202542
H 4.228150 -5.593510 0.995442
H 5.816125 -4.748849 0.904609
H 4.716418 -4.462286 2.299265
H 6.064261 1.376131 -1.961164
H 0.336912 -3.161719 -1.670623

Mn28/TS-ii_si

Frequencies, energies and thermodynamic properties:

Lowest Vibrational Mode (1/cm) = -245.1277
2nd Lowest Vibrational Mode (1/cm) = 12.0552
E(RB-P86) (a.u.) = -4636.02085363
Thermal correction to Enthalpy (a.u.) = 0.683789
Thermal correction to Gibbs Free Energy (a.u.) = 0.562762
Total Entropy (cal/Kmol) = 254.722
E(RPBE1PBE) (a.u.) = -4635.32992610

Optimised cartesian coordinates (Angstrom):

Fe -3.109079 -1.710918 -1.221354
Mn 1.084141 0.205838 0.249841
P -1.107870 0.724713 0.488051
O 1.854715 0.736480 3.039617
O 0.891285 -2.657117 0.892016
N 0.850935 -0.062851 -1.822579
N 1.677526 2.050731 -0.428153
C -2.138855 0.069514 -0.889527
C -1.594715 -0.606000 -2.064725
C -2.676993 -0.783949 -3.002653
H -2.592137 -1.273349 -3.982844
C -3.880186 -0.245802 -2.427693
H -4.876557 -0.255108 -2.889631
C -3.556760 0.274488 -1.127332
H -4.261240 0.751509 -0.434015

```
 C     -2.436109  -3.216905   0.013403
 H     -1.478249  -3.221706   0.549459
 C     -2.657062  -3.716772  -1.317967
 H     -1.900910  -4.173116  -1.971026
 C     -4.039110  -3.490362  -1.656163
 H     -4.519028  -3.738866  -2.612264
 C     -4.673493  -2.851921  -0.531148
 H     -5.722053  -2.529294  -0.480084
 C     -3.683353  -2.680547   0.499743
 H     -3.841936  -2.207077   1.477267
 C     -1.459272   2.553701   0.441268
 C     -1.034995   3.332061   1.545827
 H     -0.584765   2.838925   2.423143
 C     -1.189696   4.727938   1.540849
 H     -0.863648   5.316104   2.413639
 C     -1.756168   5.373904   0.424911
 H     -1.876232   6.468889   0.420244
 C     -2.165726   4.612156  -0.682788
 H     -2.610312   5.107492  -1.560915
 C     -2.019138   3.211324  -0.676020
 H     -2.361355   2.629294  -1.545844
 C     -2.043222   0.233315   2.023837
 C     -1.561788  -0.808006   2.846024
 H     -0.619212  -1.314209   2.594082
 C     -2.281708  -1.211718   3.985735
 H     -1.890472  -2.025934   4.616337
 C     -3.490826  -0.577327   4.321389
 H     -4.052809  -0.891829   5.215186
 C     -3.974743   0.468275   3.513260
 H     -4.916930   0.977411   3.772130
 C     -3.254594   0.873744   2.376100
 H     -3.636678   1.706783   1.765426
 C     -0.161697  -1.031851  -2.312873
 H      0.060346  -1.997400  -1.818749
 C      0.765092   1.237146  -2.518679
 H      1.103021   1.165381  -3.576418
 H     -0.302002   1.554597  -2.547323
 C      1.545144   2.279268  -1.767985
 C      2.291063   3.006999   0.320812
 H      2.379085   2.799067   1.396705
 C      2.800304   4.191777  -0.222096
 H      3.286557   4.923365   0.439888
 C      2.685678   4.414270  -1.604866
 C      2.047940   3.437549  -2.383757
 H      1.928951   3.565140  -3.470254
 C      1.523180   0.528239   1.920288
 C      0.911316  -1.501713   0.628254
 H      1.807795  -0.454146  -2.057690
 H      2.649726  -0.242068  -0.030256
 C      3.927111  -0.650646  -1.063311
 C      4.302106  -1.874047  -0.268766
 C      4.850802   0.471219  -0.529095
 C      5.123652  -1.506151   0.820120
 C      3.995899  -3.218319  -0.536226
 C      5.292726   0.002377   0.874467
 H      5.723297   0.497906  -1.222057
 C      5.646025  -2.497727   1.666808
 C      4.523257  -4.209146   0.310968
 H      3.363737  -3.479298  -1.400170
 H      6.324738   0.309538   1.142721
 C      5.340375  -3.849121   1.404938
 H      6.292712  -2.229654   2.518593
 H      4.305197  -5.272340   0.120299
 H      5.750696  -4.636136   2.058254
 O      3.439547  -0.676425  -2.228512
 H      4.378853   1.471302  -0.557684
 H      4.619091   0.422563   1.655768
 H      3.085163   5.329641  -2.067267
 H     -0.034249  -1.192214  -3.408056
---------------------------------------
Mn28/TS-ii_re
Frequencies, energies and thermodynamic properties:
 Lowest Vibrational Mode (1/cm) =                        -245.2409
 2nd Lowest Vibrational Mode (1/cm) =                      10.1402
 E(RB-P86) (a.u.) =                                      -4636.02133249
 Thermal correction to Enthalpy (a.u.) =                   0.683778
 Thermal correction to Gibbs Free Energy (a.u.) =          0.562630
 Total Entropy (cal/Kmol) =                                254.978
 E(RPBE1PBE) (a.u.) =                                    -4635.33163119
Optimised cartesian coordinates (Angstrom):
 Fe   -3.641052  -1.145898  -1.098679
 Mn    0.905005  -0.379089   0.380100
 P     -1.027333   0.806169   0.390659
 O      1.707756   0.295754   3.130098
 O     -0.175578  -2.922362   1.380731
```

```
 N      0.648265  -0.878807  -1.645534
 N      2.053974   1.075670  -0.505774
 C     -2.176721   0.287735  -0.951480
 C     -1.839792  -0.693028  -1.979883
 C     -2.901563  -0.678968  -2.957220
 H     -2.948529  -1.316399  -3.851091
 C     -3.893220   0.278952  -2.548758
 H     -4.832530   0.499287  -3.073528
 C     -3.456180   0.871457  -1.314183
 H     -3.995209   1.639979  -0.745441
 C     -3.494880  -2.575353   0.377811
 H     -2.597639  -2.786283   0.973869
 C     -3.829143  -3.184389  -0.882557
 H     -3.236187  -3.942778  -1.411462
 C     -5.065394  -2.606146  -1.343861
 H     -5.576608  -2.842432  -2.286751
 C     -5.496829  -1.640187  -0.365853
 H     -6.395156  -1.011950  -0.433155
 C     -4.525995  -1.618871   0.696959
 H     -4.552444  -0.974197   1.584959
 C     -0.799104   2.626589   0.068767
 C     -0.184748   3.400017   1.083760
 H      0.070395   2.933054   2.049294
 C      0.096587   4.760327   0.877236
 H      0.565317   5.348881   1.682147
 C     -0.215687   5.369158  -0.353726
 H      0.006366   6.435753  -0.516553
 C     -0.811169   4.606571  -1.372983
 H     -1.059709   5.073272  -2.339690
 C     -1.101849   3.244259  -1.164535
 H     -1.583590   2.666636  -1.968687
 C     -2.104916   0.857046   1.910934
 C     -1.986903  -0.144057   2.898835
 H     -1.238032  -0.940564   2.784979
 C     -2.824793  -0.136938   4.029339
 H     -2.718364  -0.926157   4.790572
 C     -3.789298   0.873539   4.189886
 H     -4.443415   0.880165   5.076266
 C     -3.908111   1.882016   3.215514
 H     -4.654910   2.683055   3.336169
 C     -3.069579   1.876814   2.087254
 H     -3.161919   2.684001   1.343766
 C     -0.601570  -1.561835  -2.065945
 H     -0.698349  -2.462751  -1.429780
 C      0.981325   0.263136  -2.521380
 H      1.317914  -0.070072  -3.528472
 H      0.059446   0.867225  -2.680836
 C      2.011343   1.141987  -1.868200
 C      2.909520   1.908373   0.144611
 H      2.927453   1.826716   1.240689
 C      3.734614   2.823510  -0.518058
 H      4.400932   3.472630   0.068764
 C      3.698277   2.884221  -1.921007
 C      2.826010   2.021680  -2.600857
 H      2.760251   2.029070  -3.699336
 C      1.372930   0.040432   2.022110
 C      0.202425  -1.873362   0.972367
 H      1.437229  -1.580562  -1.753490
 H      2.283155  -1.283681   0.262919
 C      3.315733  -2.330245  -0.579006
 C      4.552558  -1.573493  -0.176628
 C      3.185110  -3.466621   0.465239
 C      4.964703  -1.978359   1.113414
 C      5.298221  -0.651996  -0.930137
 C      3.985066  -2.983125   1.694357
 C      6.140324  -1.447516   1.668972
 C      6.476444  -0.124368  -0.371787
 H      4.963051  -0.367043  -1.940336
 C      6.891555  -0.519200   0.918783
 H      6.480332  -1.755957   2.671409
 H      7.085091   0.594125  -0.944024
 H      7.819945  -0.102354   1.341787
 O      2.868190  -2.392032  -1.758952
 H      4.488028  -3.801267   2.250178
 H      3.312081  -2.469143   2.418121
 H      2.136630  -3.747714   0.674302
 H      3.684080  -4.349957   0.003531
 H      4.339499   3.586376  -2.475302
 H     -0.502841  -1.918378  -3.116995
--------------------------------------
Mn28/TS-iii
Frequencies, energies and thermodynamic properties:
 Lowest Vibrational Mode (1/cm) =                       -654.7146
 2nd Lowest Vibrational Mode (1/cm) =                    23.2504
 E(RB-P86) (a.u.) =                                     -4213.27706326
```

```
 Thermal correction to Enthalpy (a.u.) =                  0.528742
 Thermal correction to Gibbs Free Energy (a.u.) =         0.428167
 Total Entropy (cal/Kmol) =                               211.676
 E(RPBE1PBE) (a.u.) =                                    -4212.59447046
Optimised cartesian coordinates (Angstrom):
 Fe     2.930079  -1.035684   0.677415
 Mn    -1.204237  -0.460181  -1.753637
 P     -0.222403   0.539193   0.040087
 O     -2.089208   2.043310  -3.054052
 O      1.185055  -0.440340  -3.469284
 N     -0.887602  -2.352546  -0.928503
 N     -3.023722  -0.775442  -0.797238
 C      0.929257  -0.609255   0.893515
 C      1.115607  -2.014203   0.538509
 C      1.941841  -2.605463   1.561734
 H      2.274549  -3.653000   1.576958
 C      2.280038  -1.598772   2.532546
 H      2.916784  -1.738722   3.416641
 C      1.666163  -0.365057   2.122512
 H      1.736668   0.593074   2.653392
 C      3.688315  -0.400766  -1.133486
 H      3.096287  -0.157342  -2.024564
 C      4.204514  -1.701106  -0.798587
 H      4.079585  -2.617865  -1.390486
 C      4.893003  -1.596330   0.462938
 H      5.380866  -2.419020   1.002840
 C      4.803696  -0.227861   0.906209
 H      5.212751   0.174097   1.842938
 C      4.056467   0.510789  -0.078324
 H      3.795958   1.576009  -0.028666
 C     -1.416558   1.041210   1.377486
 C     -2.268649   2.142000   1.119614
 H     -2.170121   2.703913   0.176216
 C     -3.235147   2.534597   2.060017
 H     -3.884050   3.399165   1.847125
 C     -3.375577   1.825566   3.268508
 H     -4.133818   2.133038   4.006067
 C     -2.543679   0.722538   3.526766
 H     -2.646081   0.161097   4.469265
 C     -1.570248   0.330964   2.587405
 H     -0.919193  -0.528915   2.808509
 C      0.750881   2.116745  -0.155335
 C      1.237154   2.506163  -1.421885
 H      1.021696   1.892583  -2.307641
 C      2.005182   3.676529  -1.565703
 H      2.374987   3.964990  -2.562507
 C      2.295211   4.475750  -0.446156
 H      2.894760   5.392904  -0.559642
 C      1.806602   4.101877   0.819584
 H      2.020493   4.725835   1.702053
 C      1.036361   2.935064   0.963310
 H      0.644041   2.668516   1.957118
 C      0.476539  -2.752797  -0.637109
 H      1.087793  -2.614878  -1.553324
 C     -1.811365  -2.649026   0.145899
 H     -2.013657  -3.746703   0.248412
 H     -1.418047  -2.338470   1.155856
 C     -3.106392  -1.908782  -0.043630
 C     -4.143369  -0.025320  -0.963900
 C     -5.377348  -0.366726  -0.400840
 C     -5.470898  -1.540908   0.366665
 C     -4.318849  -2.318622   0.541708
 C     -1.734659   1.043260  -2.533839
 C      0.266148  -0.437164  -2.724040
 H     -1.525043  -2.005438  -2.330010
 H     -1.898521  -1.437346  -2.963432
 H     -4.039205   0.878460  -1.581460
 H     -6.249683   0.280761  -0.572549
 H     -4.344312  -3.247467   1.131336
 H     -6.427647  -1.845995   0.817843
 H      0.523139  -3.846063  -0.397121
---------------------------------------
Mn29/TS-ii_re
Frequencies, energies and thermodynamic properties:
 Lowest Vibrational Mode (1/cm) =                        -217.6187
 2nd Lowest Vibrational Mode (1/cm) =                     10.5129
 E(RB-P86) (a.u.) =                                      -5106.64887574
 Thermal correction to Enthalpy (a.u.) =                  0.766090
 Thermal correction to Gibbs Free Energy (a.u.) =         0.625259
 Total Entropy (cal/Kmol) =                               296.404
 E(RPBE1PBE) (a.u.) =                                    -5106.04349630
Optimised cartesian coordinates (Angstrom):
 Fe    -4.206909   0.003013  -1.360632
 Mn     0.170633  -0.992765   0.463972
 P     -1.361670   0.650777   0.722952
```

O	0.608552	-1.497404	3.329846
O	-1.635378	-3.294984	0.617624
N	0.027711	-0.825369	-1.638582
N	1.767118	0.301376	0.053699
C	-2.437551	0.852519	-0.757725
C	-2.261765	0.123408	-2.014111
C	-3.162097	0.712262	-2.979481
H	-3.292551	0.386751	-4.019091
C	-3.895076	1.773401	-2.344377
H	-4.672807	2.389694	-2.815059
C	-3.459854	1.859216	-0.978169
H	-3.832453	2.568753	-0.228207
C	-4.627273	-1.734724	-0.335876
H	-3.898016	-2.342129	0.215954
C	-4.956599	-1.879408	-1.729422
H	-4.529108	-2.618176	-2.420701
C	-5.928233	-0.870377	-2.066072
H	-6.365829	-0.701095	-3.059067
C	-6.202190	-0.103172	-0.877872
H	-6.885708	0.753597	-0.807116
C	-5.396697	-0.634465	0.190652
H	-5.358056	-0.257811	1.220868
C	-0.610002	2.344954	0.930838
C	0.112709	2.592629	2.123519
H	0.153938	1.820716	2.909290
C	0.779750	3.813090	2.317300
H	1.328849	3.991574	3.255733
C	0.752103	4.802573	1.315149
H	1.277327	5.759044	1.466036
C	0.051600	4.560230	0.121344
H	0.024436	5.326908	-0.669610
C	-0.626132	3.340203	-0.070690
H	-1.181775	3.173829	-1.006462
C	-2.558913	0.624208	2.150941
C	-2.856929	-0.594226	2.798839
H	-2.364741	-1.522201	2.474673
C	-3.783954	-0.634378	3.856463
H	-4.003861	-1.593805	4.351296
C	-4.422908	0.543161	4.283956
H	-5.146238	0.511530	5.114269
C	-4.126106	1.764115	3.650474
H	-4.615442	2.693611	3.982990
C	-3.198183	1.805392	2.595004
H	-2.962277	2.771677	2.122150
C	-1.313829	-1.041134	-2.278861
H	-1.714665	-1.938968	-1.768544
C	0.728845	0.368706	-2.155668
H	1.097996	0.228134	-3.193445
H	0.012436	1.220664	-2.186398
C	1.867453	0.726346	-1.243068
C	2.741955	0.700299	0.879291
C	3.834094	1.490563	0.504430
C	3.983855	1.966275	-0.830339
C	2.917676	1.530020	-1.680591
C	-1.190677	-1.381033	-3.774052
H	-2.166693	-1.721617	-4.173318
H	-0.860865	-0.514587	-4.382892
H	-0.464815	-2.207683	-3.913225
C	0.464366	-1.245473	2.182328
C	-0.951684	-2.324024	0.550026
H	0.628969	-1.654518	-1.905145
H	1.279833	-2.163258	0.151291
N	5.024041	2.753189	-1.247801
C	5.524711	2.738369	-2.622679
H	5.219168	3.645903	-3.188961
H	5.173939	1.843923	-3.164109
H	6.634794	2.707743	-2.595027
C	5.738904	3.655311	-0.344598
H	6.725799	3.246140	-0.033928
H	5.139830	3.869279	0.556727
H	5.915955	4.615679	-0.873604
C	2.158013	-3.166613	-0.937276
C	3.459913	-2.877702	-0.242816
C	1.633792	-4.470906	-0.290186
C	3.593593	-3.710483	0.891183
C	4.478702	-1.990126	-0.626976
C	2.351332	-4.564858	1.074420
C	4.762813	-3.649652	1.666786
C	5.647079	-1.929642	0.153727
H	4.356414	-1.369404	-1.529266
C	5.785473	-2.753902	1.292005
H	4.887061	-4.294671	2.552254
H	6.464060	-1.245449	-0.126210
H	6.709000	-2.701432	1.891034
O	1.861087	-2.772677	-2.098072

```
 H      2.587005  -5.603940   1.384326
 H      1.716528  -4.125800   1.877467
 H      0.530079  -4.514132  -0.237944
 H      1.970674  -5.293319  -0.963050
 F      2.881319   1.959987  -2.966204
 F      4.774267   1.744557   1.438325
 F      2.700194   0.291951   2.151641
---------------------------------------
Mn29/TS-ii_si
Frequencies, energies and thermodynamic properties:
 Lowest Vibrational Mode (1/cm) =                       -222.6418
 2nd Lowest Vibrational Mode (1/cm) =                    13.3171
 E(RB-P86) (a.u.) =                                     -5106.64799510
 Thermal correction to Enthalpy (a.u.) =                 0.765998
 Thermal correction to Gibbs Free Energy (a.u.) =        0.625759
 Total Entropy (cal/Kmol) =                              295.158
 E(RPBE1PBE) (a.u.) =                                   -5106.04062738
Optimised cartesian coordinates (Angstrom):
 Fe     3.950460  -0.511342  -1.420990
 Mn    -0.332376   0.831038   0.418944
 P      1.115145  -0.873507   0.744030
 O     -0.707447   1.474335   3.266544
 O      1.614683   3.022210   0.442555
 N     -0.232145   0.561384  -1.674648
 N     -1.992832  -0.405035   0.098860
 C      2.146034  -1.217051  -0.742236
 C      1.989237  -0.544849  -2.032444
 C      2.831482  -1.238582  -2.980673
 H      2.960179  -0.977071  -4.038406
 C      3.511304  -2.308449  -2.302534
 H      4.240014  -2.995621  -2.753088
 C      3.100157  -2.295490  -0.926326
 H      3.444417  -2.987099  -0.146640
 C      4.497904   1.239381  -0.482400
 H      3.819815   1.907878   0.063760
 C      4.792366   1.308831  -1.889418
 H      4.385770   2.042217  -2.598809
 C      5.695615   0.233336  -2.210383
 H      6.092713  -0.000702  -3.207241
 C      5.962081  -0.499788  -0.999082
 H      6.598832  -1.390340  -0.911453
 C      5.220246   0.119332   0.068329
 H      5.191973  -0.212209   1.114338
 C      0.279365  -2.510000   1.061578
 C     -0.403321  -2.666607   2.292665
 H     -0.362757  -1.864644   3.047770
 C     -1.131707  -3.836106   2.564709
 H     -1.648188  -3.944233   3.531969
 C     -1.205789  -4.864421   1.604953
 H     -1.778447  -5.781046   1.817826
 C     -0.544965  -4.712087   0.374155
 H     -0.596434  -5.510009  -0.384020
 C      0.193119  -3.543261   0.103027
 H      0.716174  -3.447793  -0.861018
 C      2.344468  -0.835291   2.144598
 C      2.719448   0.396828   2.722747
 H      2.267491   1.331807   2.362634
 C      3.672949   0.441912   3.756432
 H      3.952875   1.412314   4.196529
 C      4.262002  -0.744112   4.229455
 H      5.006114  -0.708405   5.041033
 C      3.888618  -1.978244   3.665777
 H      4.338498  -2.913966   4.034583
 C      2.934386  -2.024214   2.634298
 H      2.638883  -2.999405   2.216487
 C      1.110693   0.662340  -2.341190
 H      1.573076   1.557079  -1.879953
 C     -1.014703  -0.606527  -2.130963
 H     -1.401258  -0.481022  -3.164394
 H     -0.349520  -1.499511  -2.147574
 C     -2.148906  -0.867032  -1.180521
 C     -2.968037  -0.722772   0.959804
 C     -4.115063  -1.456329   0.636320
 C     -4.327337  -1.960655  -0.680407
 C     -3.257555  -1.615883  -1.568137
 C      0.986751   0.937252  -3.849581
 H      1.977268   1.190839  -4.276844
 H      0.590313   0.066598  -4.410847
 H      0.316523   1.803707  -4.020601
 C     -0.588111   1.172871   2.128233
 C      0.866101   2.100371   0.426413
 H     -0.778282   1.419411  -1.971101
 H     -1.369226   2.062060   0.058410
 N     -5.421793  -2.695091  -1.049525
 C     -5.955853  -2.688117  -2.412222
```

```
 H     -5.720394  -3.628249  -2.958198
 H     -5.566072  -1.832059  -2.987920
 H     -7.060859  -2.588768  -2.358190
 C     -6.171619  -3.520028  -0.101602
 H     -7.118896  -3.034673   0.222260
 H     -5.563554  -3.750833   0.789455
 H     -6.428803  -4.479434  -0.598076
 C     -2.220750   3.043345  -1.063587
 C     -1.831999   4.308536  -0.348563
 C     -3.609775   2.671156  -0.491653
 C     -2.705123   4.531187   0.740282
 C     -0.820686   5.225273  -0.679141
 C     -3.695237   3.386899   0.873482
 H     -4.350907   3.102381  -1.203891
 C     -2.563416   5.689213   1.522323
 C     -0.683542   6.385073   0.104090
 H     -0.160423   5.031797  -1.539584
 H     -4.717381   3.730905   1.134379
 C     -1.548384   6.612472   1.197118
 H     -3.237347   5.881857   2.373382
 H      0.097044   7.124849  -0.136157
 H     -1.432228   7.527804   1.799830
 O     -1.810432   2.703310  -2.207369
 H     -3.785761   1.579502  -0.460793
 H     -3.367471   2.702323   1.688443
 F     -2.873957  -0.272787   2.216243
 F     -5.039279  -1.633350   1.602668
 F     -3.280680  -2.084155  -2.839979
---------------------------------------
Mn30/TS-ii_re
Frequencies, energies and thermodynamic properties:
 Lowest Vibrational Mode (1/cm) =                         -259.2754
 2nd Lowest Vibrational Mode (1/cm) =                      13.6684
 E(RB-P86) (a.u.) =                                       -5423.77684459
 Thermal correction to Enthalpy (a.u.) =                   0.718147
 Thermal correction to Gibbs Free Energy (a.u.) =          0.575615
 Total Entropy (cal/Kmol) =                                299.982
 E(RPBE1PBE) (a.u.) =                                     -5423.25645729
Optimised cartesian coordinates (Angstrom):
 Fe     4.619876  -0.321966  -1.240368
 Mn     0.301220   1.073598   0.469569
 P      1.614861  -0.762657   0.658966
 O     -0.194979   1.404014   3.352666
 O      2.376085   3.100254   0.905184
 N      0.547341   1.074352  -1.622000
 N     -1.414376   0.042795  -0.100368
 C      2.729386  -0.977330  -0.787619
 C      2.714234  -0.132386  -1.982500
 C      3.580521  -0.755943  -2.957036
 H      3.805777  -0.368464  -3.958412
 C      4.137480  -1.951730  -2.386033
 H      4.853863  -2.625023  -2.875454
 C      3.624898  -2.089258  -1.051449
 H      3.864767  -2.899648  -0.351187
 C      5.209691   1.244101  -0.037673
 H      4.540701   1.885339   0.550550
 C      5.612005   1.476406  -1.399674
 H      5.308577   2.325347  -2.027007
 C      6.464163   0.388098  -1.805249
 H      6.918195   0.258620  -2.796813
 C      6.591016  -0.516405  -0.691189
 H      7.158990  -1.456377  -0.685565
 C      5.813769   0.010038   0.400380
 H      5.686212  -0.453738   1.386994
 C      0.641493  -2.350930   0.689910
 C     -0.162243  -2.601145   1.829002
 H     -0.140405  -1.901537   2.680435
 C     -0.988164  -3.735486   1.887137
 H     -1.599204  -3.919438   2.785232
 C     -1.038869  -4.631457   0.801516
 H     -1.688370  -5.519962   0.846449
 C     -0.257498  -4.383004  -0.339569
 H     -0.290277  -5.077071  -1.194615
 C      0.578651  -3.250725  -0.396581
 H      1.195670  -3.080914  -1.292335
 C      2.727475  -0.996959   2.133427
 C      3.143334   0.118302   2.892539
 H      2.788148   1.125266   2.631802
 C      4.015697  -0.044893   3.984181
 H      4.329289   0.836187   4.566305
 C      4.480980  -1.324863   4.334204
 H      5.161680  -1.452148   5.190902
 C      4.064720  -2.443763   3.589371
 H      4.417153  -3.451764   3.860557
 C      3.191053  -2.282288   2.499862
```

H 2.859765 -3.170007 1.938604
C 1.938825 1.164736 -2.182775
H 2.421940 1.957782 -1.578801
C -0.266493 0.028424 -2.271579
H -0.559276 0.296641 -3.308716
H 0.337440 -0.904624 -2.344920
C -1.488060 -0.256114 -1.450011
C -2.458734 -0.277756 0.654496
C -3.746714 -1.201798 -1.217368
C -2.611602 -0.846343 -2.003213
C 1.939372 1.640857 -3.645273
H 2.971116 1.881097 -3.970304
H 1.534658 0.879488 -4.342855
H 1.334424 2.565226 -3.740034
C -0.038739 1.225506 2.194588
C 1.580438 2.239865 0.719868
H 0.070978 1.998482 -1.841675
H -0.623026 2.421768 0.199628
C -1.271766 3.581245 -0.772591
C -2.657751 3.406927 -0.208137
C -0.658596 4.751704 0.037702
C -2.780285 4.133571 0.997753
C -3.746382 2.714366 -0.763235
C -1.461261 4.795961 1.356471
C -4.010793 4.158691 1.674077
C -4.978128 2.743137 -0.083747
H -3.629628 2.182495 -1.721506
C -5.105717 3.457780 1.127262
H -4.127259 4.724227 2.613272
H -5.851951 2.217737 -0.501233
H -6.077706 3.477084 1.646162
O -0.945754 3.287781 -1.959921
H -1.592631 5.819387 1.764485
H -0.947942 4.199745 2.144954
H 0.436754 4.667269 0.162536
H -0.858245 5.665995 -0.567383
C -3.656290 -0.903279 0.194071
C -4.752958 -1.243829 1.041482
C -4.921335 -1.818779 -1.725865
C -5.890704 -1.851726 0.514893
C -5.976203 -2.139990 -0.872173
F -2.609957 -1.111567 -3.322643
F -5.046238 -2.109693 -3.026896
F -7.079397 -2.721528 -1.345695
F -6.916829 -2.172079 1.306824
F -4.726931 -0.995633 2.355403
F -2.378140 0.008378 1.953788

Mn30/TS-ii_si
Frequencies, energies and thermodynamic properties:
Lowest Vibrational Mode (1/cm) = -274.0694
2nd Lowest Vibrational Mode (1/cm) = 10.0912
E(RB-P86) (a.u.) = -5423.77569927
Thermal correction to Enthalpy (a.u.) = 0.717917
Thermal correction to Gibbs Free Energy (a.u.) = 0.574413
Total Entropy (cal/Kmol) = 302.031
E(RPBE1PBE) (a.u.) = -5423.25244247
Optimised cartesian coordinates (Angstrom):
Fe 4.285824 -1.110165 -1.242283
Mn 0.175117 0.911162 0.359633
P 1.275426 -1.038550 0.704097
O -0.254624 1.538588 3.204241
O 2.485430 2.701414 0.618429
N 0.389190 0.702034 -1.724132
N -1.651491 0.032553 -0.107061
C 2.339822 -1.501328 -0.722578
C 2.402303 -0.767028 -1.987052
C 3.176115 -1.568355 -2.907944
H 3.428292 -1.297561 -3.940777
C 3.601804 -2.766328 -2.237287
H 4.229120 -3.557308 -2.669612
C 3.098152 -2.727617 -0.892876
H 3.254786 -3.496462 -0.125452
C 5.073597 0.484912 -0.201052
H 4.493997 1.257827 0.319960
C 5.485969 0.530459 -1.578988
H 5.280495 1.343578 -2.288173
C 6.195954 -0.688005 -1.872890
H 6.619445 -0.969783 -2.846218
C 6.225179 -1.486008 -0.673861
H 6.675662 -2.482602 -0.573775
C 5.529220 -0.763575 0.358746
H 5.357173 -1.107974 1.386666
C 0.132545 -2.499870 0.875551
C -0.669706 -2.568905 2.040788

H	-0.549917	-1.814238	2.835195
C	-1.616322	-3.594292	2.198395
H	-2.224832	-3.638099	3.115785
C	-1.789849	-4.560005	1.187868
H	-2.533596	-5.363118	1.311010
C	-1.008998	-4.491296	0.021720
H	-1.136578	-5.241744	-0.774806
C	-0.053248	-3.468401	-0.135192
H	0.560644	-3.440019	-1.048610
C	2.373675	-1.270335	2.189800
C	2.919426	-0.148479	2.850375
H	2.674346	0.865982	2.506053
C	3.782393	-0.315102	3.948985
H	4.198284	0.571602	4.453261
C	4.108381	-1.604827	4.404582
H	4.781686	-1.734490	5.266757
C	3.562451	-2.729218	3.758212
H	3.805478	-3.743762	4.112401
C	2.698278	-2.563889	2.661777
H	2.264824	-3.453390	2.178402
C	1.773775	0.586489	-2.297299
H	2.351160	1.369814	-1.768188
C	-0.544776	-0.300508	-2.273135
H	-0.815080	-0.099676	-3.331267
H	-0.052567	-1.299650	-2.256299
C	-1.781771	-0.361678	-1.428675
C	-2.717465	-0.096755	0.675798
C	-4.144368	-0.972325	-1.117295
C	-2.980492	-0.839356	-1.930269
C	1.805635	0.929319	-3.796352
H	2.852718	1.018170	-4.147878
H	1.304208	0.161915	-4.420659
H	1.310092	1.905873	-3.969490
C	-0.126377	1.248289	2.065628
C	1.586791	1.938117	0.504303
H	0.014080	1.650847	-2.023192
H	-0.565549	2.348287	-0.022958
C	-1.137983	3.458054	-1.090876
C	-0.499790	4.618336	-0.369567
C	-2.590603	3.414066	-0.551643
C	-1.322830	5.028199	0.703318
C	0.688603	5.296520	-0.684440
C	-2.540909	4.127540	0.816236
H	-3.194485	4.006439	-1.276946
C	-0.950367	6.133719	1.485643
C	1.056984	6.405301	0.098457
H	1.306271	4.963636	-1.533795
H	-3.467555	4.686220	1.061837
C	0.243286	6.817630	1.176265
H	-1.582441	6.470942	2.323661
H	1.980730	6.960688	-0.130634
H	0.541813	7.690698	1.778902
O	-0.801728	3.058132	-2.244325
H	-3.017725	2.393857	-0.532090
H	-2.385466	3.386994	1.633464
C	-3.995257	-0.583877	0.267530
C	-5.119448	-0.699220	1.139839
C	-5.400333	-1.453598	-1.575117
C	-6.338438	-1.175367	0.662066
C	-6.480289	-1.554043	-0.698506
F	-3.032278	-1.204017	-3.224262
F	-5.580616	-1.821953	-2.849575
F	-7.660573	-2.006111	-1.124709
F	-7.390633	-1.282799	1.476660
F	-5.042479	-0.357189	2.430519
F	-2.582427	0.276365	1.949389
